# APOBEC3B coordinates R-loop to promote replication stress and sensitize cancer cells to ATR/Chk1 inhibitors

**DOI:** 10.1038/s41419-023-05867-0

**Published:** 2023-06-03

**Authors:** Chunyan Zong, Zhe Zhang, Li Gao, Jie He, Yiran Wang, Qian Li, Xiaoting Liu, Jie Yang, Di Chen, Rui Huang, Guopei Zheng, Xiaoliang Jin, Wu Wei, Renbing Jia, Jianfeng Shen

**Affiliations:** 1 Department of Ophthalmology, Ninth People’s Hospital, Shanghai Jiao Tong University School of Medicine, Shanghai, 200025 China; 2 Shanghai Key Laboratory of Orbital Diseases and Ocular Oncology, Shanghai, 200025 China; 3 Institute of Translational Medicine, National Facility for Translational Medicine, Shanghai Jiao Tong University, Shanghai, 200240 China; 4 CAS Key Laboratory of Computational Biology, Shanghai Institute of Nutrition and Health, University of Chinese Academy of Sciences, Chinese Academy of Sciences, Shanghai, 200031 China; 5 Lingang Laboratory, Shanghai, 200031 China

**Keywords:** Cancer therapy, DNA replication, DNA damage response

## Abstract

The cytidine deaminase, Apolipoprotein B mRNA editing enzyme catalytic subunit 3B (APOBEC3B, herein termed A3B), is a critical mutation driver that induces genomic instability in cancer by catalyzing cytosine-to-thymine (C-to-T) conversion and promoting replication stress (RS). However, the detailed function of A3B in RS is not fully determined and it is not known whether the mechanism of A3B action can be exploited for cancer therapy. Here, we conducted an immunoprecipitation-mass spectrometry (IP-MS) study and identified A3B to be a novel binding component of R-loops, which are RNA:DNA hybrid structures. Mechanistically, overexpression of A3B exacerbated RS by promoting R-loop formation and altering the distribution of R-loops in the genome. This was rescued by the R-loop gatekeeper, Ribonuclease H1 (RNASEH1, herein termed RNH1). In addition, a high level of A3B conferred sensitivity to ATR/Chk1 inhibitors (ATRi/Chk1i) in melanoma cells, which was dependent on R-loop status. Together, our results provide novel insights into the mechanistic link between A3B and R-loops in the promotion of RS in cancer. This will inform the development of markers to predict the response of patients to ATRi/Chk1i.

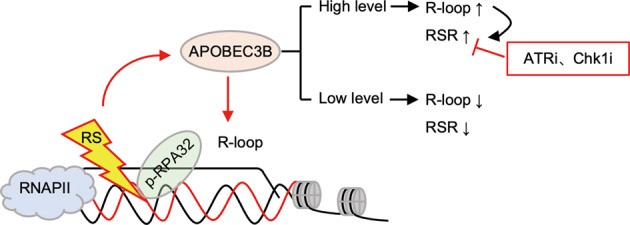

## Introduction

Apolipoprotein B mRNA editing enzyme catalytic subunit 3B (APOBEC3B, herein termed A3B) belongs to the cytidine deaminase APOBEC3 family, which can cause the accumulation of cytosine-to-thymine (C-to-T) mutations in DNA and induce genome instability [1]. Thus, A3B activation usually leads to a mutator phenotype that is commonly observed in cancers [2, 3]. Genomic uracil lesions catalyzed by A3B are responsible for a large proportion of both dispersed and clustered mutations in breast, lung, cervix, head, neck and bladder cancers [4–6]. With defined roles in mutagenesis, A3B is reported to promote both initiation and progression of cancer, and overexpression of A3B is usually associated with poor clinical outcomes of cancer patients [7–9], including primary head/neck mucosal melanomas (MMs) [10]. Recently, emerging evidence has demonstrated that A3B activation was positively correlated with elevated levels of DNA RS, pointing to the additional function of A3B in promoting cancer [3, 11, 12]. RS often occurs in response to various intrinsic or extrinsic stimuli, such as chemotherapy drugs, hypoxia, ultraviolet light and other forms of radiation [13, 14]. The replication stress response (RSR) at stalled replication forks is governed by Ataxia telangiectasia and Rad3-related protein (ATR), a key molecule in modulating DNA damage repair [15]. ATR phosphorylates and activates Checkpoint kinase 1 (Chk1), resulting in the amplification of RSR signaling [16]. The RSR signaling cascades subsequently stabilize the stalled replication forks and promote their restart [17]. In contrast to the well-established mutagenic functions, however, the underlying mechanisms through which A3B exerts the modulation of RS have not yet been characterized. It was noticed that A3B-exacerbated RS might be through replication fork slowing and incomplete replication of DNA as suggested by accumulated FANCD2-flanked ultrafine bridges and 53BP1 foci [3]. But it remains elusive whether these phenomena mediated by A3B were entangled with transcriptional apparatus.

R-loops are intermediates of transcription consisting of a complementary RNA:DNA hybrid plus a displaced DNA strand (ssDNA) [18]. They are involved in various biological processes, such as transcription initiation and termination, telomere homeostasis and class-switching recombination [19, 20]. The co-transcriptionally formed R-loop structures could halt DNA replication if they occurred in the opposite direction to replication fork elongation [21–23], highlighting the necessity to tightly control the formation and distribution of R-loops. Otherwise, unscheduled R-loops interfere with DNA replication and transcription, resulting in transcription elongation defects and genome instability [19, 20, 24]. Among the regulators of R-loop, RNASEH1 is a gatekeeper which cleaves the RNA moiety of the RNA:DNA hybrid to prevent R-loop formation [25]. A recent study has demonstrated the involvement of fork cleavage by endonuclease MUS81, transcriptional reactivation and fork relegation by LIG4/XRCC4 complex to resolve the stalled replication forks by co-transcriptional R-loops [23]. Unexpectedly, we identified A3B to be a novel interacting component of R-loops through an IP-MS, suggesting the functional connectivity between A3B and R-loop modulation. In this study, we aimed to investigate the mechanisms of A3B in modulating RS through its interaction with R-loops, and to explore the therapeutic opportunities by exploiting these mechanisms.

## Results

### A3B is overexpressed in melanoma

**Fig. 1:**
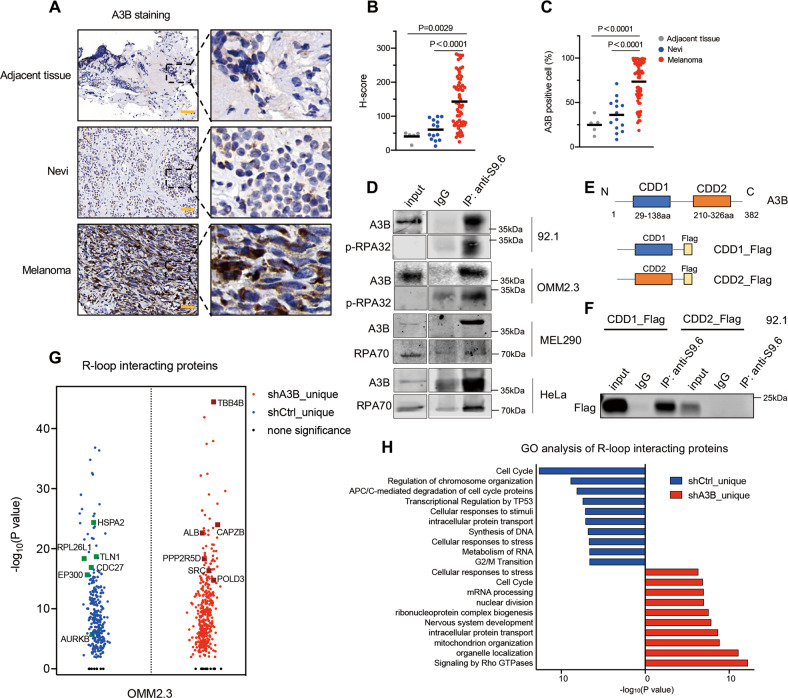
A3B interacts with R-loops and promotes RS. **A** Representative IHC staining of A3B in nevus, ocular melanoma intratumor and peritumor tissues. Scale bar, 50 μm. Images on the right are 5-fold magnification of the selected area. Quantification of A3B H-scores **B** and percentage of A3B positive cells **C** of the indicated tissues. Mean values are indicated by black lines (unpaired t-test). **D** Co-IP of R-loop using anti-S9.6 antibody. Endogenous A3B, p-RPA32 and RPA70 were detected by western blot. Cell lysates (1%) were loaded as input. **E** Schematic representation of full-length A3B (1-382 aa) with two domains named CDD1 (29-138 aa) and CDD2 (210-326 aa) and plasmid CDD1_Flag and CDD2_Flag. CDD, cytidine deaminase domain. **F** Co-IP of R-loop using anti-S9.6 antibody. 92.1 cells were transfected with Flag-tagged CDD1 or CDD2. Flag was detected by western blot. Cell lysates (1%) were loaded as input. **G** Volcano plot of differentially expressed R-loop binding proteins before (blue) or after A3B depletion (red) in OMM2.3 cell as determined by IP-MS. Protein targets (*p* < 0.05) were shown. **H** Bar graph of gene ontology analysis of the proteins in (**G**). blue: R-loop binding proteins before A3B depletion; red: R-loop binding proteins after A3B depletion.

We here used melanoma as a model to elucidate the biological function of A3B. In order to evaluate the expression of A3B, we performed immunohistochemical (IHC) staining in ocular melanoma (*n* = 67), adjacent normal (*n* = 5) and benign nevi tissues (*n* = 14). Concordant with previous reports [10, 26], A3B staining was mainly found in the nuclei. Strong staining was detected in melanoma tissues, while adjacent normal and nevi tissues had only weak or mild immunopositivity (Fig. 1A). Statistical analysis demonstrated that A3B levels were significantly higher in melanoma tissue than in adjacent normal (*P* = 0.0029) and nevi tissues (*P* < 0.0001) (Fig. 1B). Similarly, the percentage of A3B-positive cells was also higher in melanoma tissue (Fig. 1C). We next examined the expression of A3B in a variety of melanoma cell lines. Both qPCR and western blotting revealed that the expression of A3B was dramatically elevated in melanoma cell lines with lines OMM2.3 and 92.1 exhibiting the highest levels (Fig. S1A, B). Also, APOBEC-driven C to T mutation is the main single base substitution in Skin cutaneous Melanoma (SKCM) and Uveal Melanoma (UVM) (Fig. S1C).

### A3B interacts with R-loops and regulates the interactome

Among various cancer types, including UVM and SKCM, we observed a positive correlation between the expression of A3B and RNASEH1, a well-established nuclease that resolves R-loops [27] (Fig. S2). By analyzing the R-loop interactors from previous reports [28, 29], we noticed the involvement of APOBEC family members such as APOBEC3A (A3A), APOBEC3C (A3C) and APOBEC3D (A3D) in a context-dependent manner. This result prompted us to hypothesize that A3B might also be linked to R-loops in our scenario. We tested this by IP-MS analysis with a monoclonal antibody (S9.6) to investigate R-loop interacting components. R-loops interact with Replication protein A (RPA), a key member of the ssDNA-binding complex responsible for DNA replication and damage repair [30]. As expected, we observed this interaction; the phosphorylated form of RPA32 (p-RPA32 S4/S8) and RPA70 were detected in our IP-MS and IP-western blotting assays in multiple cancer cell lines (Fig. 1D and Fig. S3A, Table S1). Surprisingly, A3B was identified as a novel R-loop binding protein (Fig. 1D and Fig. S3A, B). To test if this interaction was restricted to melanoma, we examined the binding of A3B with R-loop in renal cancer cell lines 786 O and 769 P (Fig. S3A). A3B was a two-domain APOBEC3 protein with two cytidine deaminase domains (CDD) [31]. We further noticed that the N- terminal CDD1, which mediates viral RNA binding, was the R-loop binding domain (Fig. 1E, F). We then sought to explore the impact of A3B upon the R-loop interactome using control (shCtrl) and A3B-depleted (shA3B) OMM2.3 and 92.1 cells (Fig. S3C, D). IP-MS analysis of these cells revealed that 270 binding proteins completely lost their interactions with R-loops in the absence of A3B, including cell cycle-related regulatory proteins Cell division cycle 27 (CDC27) and Aurora kinase B (AURKB) (Fig. 1G and Fig. S3E, F). In addition, 301 proteins exhibited impaired interactions with R-loops after A3B-depletion because their protein amounts were markedly reduced (>2-fold change compared with control), including DNA replication-associated protein minichromosomal maintenance complex component 4 (MCM4). In contrast, 367 proteins gained the ability to interact with R-loops when A3B was depleted (Fig. 1G and Fig. S3E, F). Gene ontology analysis of the A3B-dependent R-loop interactome demonstrated the enrichment of proteins involved in regulating the cell cycle and cellular response to stress (Fig. 1H), consistent with previously established functions of A3B [32]. We further compared the R-loop interactome in OMM2.3 with reported R-loop interactors in MCF10A [29] and HeLa [28] cells and found 254 overlapped R-loop interactors (Fig. S3G). The overlapped interactors are mainly involved in RNA metabolism-related functions (Fig. S3H). In addition to RNA metabolism, OMM2.3 specific R-loop interactors also participate in cell cycle and DNA repair (Fig. S3I). These data indicated that A3B might maintain the R-loop interactome to modulate cell cycle, stress response or gene transcription.

### A3B regulates the formation and genomic distribution of R-loops

**Fig. 2:**
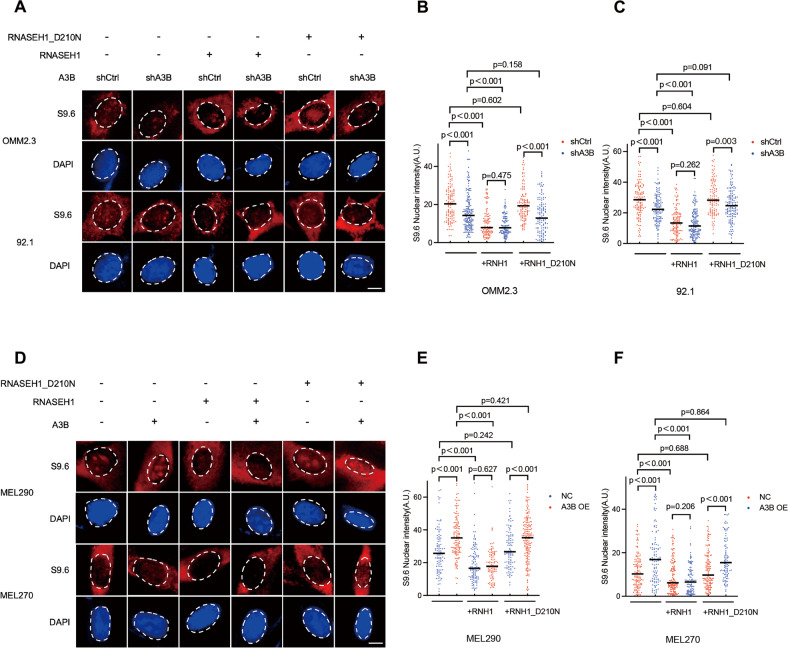
A3B promotes the formation of R-loops in RNASEH1-dependent manner. **A** Representative images of S9.6 staining in OMM2.3 and 92.1 cells in each condition. Scale bar, 4 μm. Quantification of nuclear S9.6 signal intensity of **B** OMM2.3 and **C** 92.1 cells in (**A**). **D** Representative images of S9.6 staining in MEL290 and MEL270 cells in each condition. Scale bar, 4 μm. Quantification of nuclear S9.6 signal intensity of MEL290 **E** and MEL270 **F** cells in (**D**). Data in (**B**, **C**, **E**, **F**) are presented as a scatter plot (n ≥ 100). Median values are indicated by black lines (two-tailed Mann–Whitney U-test). AU, arbitrary units.

To explore the regulatory function of A3B on R-loops, we calculated the number of R-loops by immunofluorescence staining using the S9.6 antibody in control and A3B knock-down cells. S9.6 nuclear signals were significantly reduced in A3B-depleted OMM2.3 (*P* < 0.001) and 92.1 (*P* < 0.001) cells (Fig. 2A–C). In contrast, A3B overexpression in MEL270 (*P* < 0.001) and MEL290 (*P* < 0.001) cells resulted in significant increases in S9.6 staining (Fig. 2D–F). Overexpression of RNASEH1 almost abrogated the formation of R-loops (Fig. 2B, C, E–F and Fig. S4A–F). The catalytically inactive form of RNASEH1, which harbored a D210N mutation in the catalytic domain but retained the binding affinity to R-loops, served as a negative control.

**Fig. 3:**
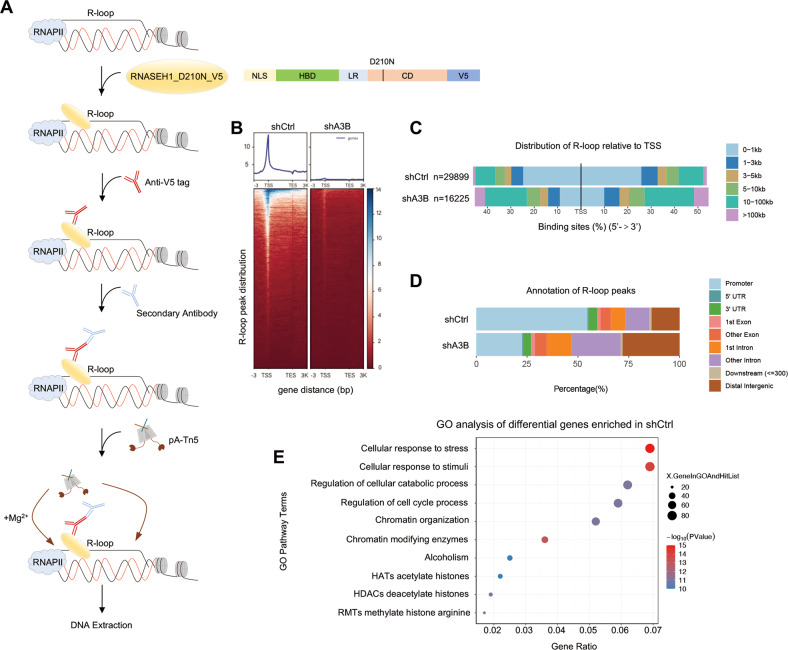
Genome-wide distribution analysis of R-loop and A3B reveals coordinated regulations on RS. **A** Workflow for R-loop mapping with CUT&Tag assay. Catalytically inactive RNASEH1 plasmids with V5 tags were transformed into A3B knockdown and control OMM2.3 cell lines. Schematic diagram of the structure of the tool plasmid ppyCAG_RNASEH1_D210N_V5 is shown on the right. **B** The heatmap presentation of R-loop signals in regions ± 3 Kb from TSS region. **C** The peak distribution of CUT&Tag signals for R-loop relative to TSS. The distribution ranges are marked according to the colors shown in the labels on the right. **D** Annotation of R-loop peaks captured by CUT&Tag assay. Genomic regions are coded on the right. UTR, untranslated region. **E** GO analysis of differential genes between A3B knock down and control cell line captured by CUT&Tag.

We then applied the CUT&Tag approach to characterize the genomic distribution of R-loops in control and A3B-depleted OMM2.3 cells with RNASEH1_D210N_V5 expression (Fig. 3A). We used a V5 antibody to pull-down R-loops, and to harvest the DNA fragments from R-loop regions for sequencing. The R-loop peaks were mainly distributed near transcription start sites (TSS) (Fig. 3B, C). Knockdown of A3B markedly reduced the number of R-loop peaks, confirming our findings in Fig. 2A (Fig. 3B, C). Importantly, A3B depletion redistributed the genome-wide positioning of R-loops. The enrichment of R-loops at TSS was diminished and redirected to more diverse regions across the genome (Fig. 3C). Further annotation revealed that R-loops were preferentially localized at the promoter, intronic and distal intercellular regions of the genome (Fig. 3D), correlating with the highest R-loop signal intensity at promoter regions (Fig. S5A, B). This localization preference of R-loops was impaired by A3B knockdown (Fig. 3D).

We then characterized the function of genes that showing different R-loop peaks after A3B knockdown. The R-loop peaks of a total of 459 genes were depleted by A3B knockdown, most of which function in stress response and cell cycle processes (Fig. 3E). From this observation, we reasoned that A3B may regulate RSR through stress-related, R-loop containing genes.

### A3B coordinates R-loops to regulate the RSR

To further explore the relationship between A3B and R-loop, we performed ChIP-seq analysis of A3B. The profiles of A3B distribution of our study were in agreement with those of previous reports using the breast cancer cell line MCF7 (GSE56979) [33] (Fig. S5C–E). We performed an integrated analysis of R-loop CUT&Tag-seq and A3B ChIP-seq data. A Venn diagram showed that 8,167 (54.7%) out of 14,930 genes co-occurred in A3B and R-loop peak regions (Fig. S5F). It is worth noting that the overall C-to-T mutation rate in R-loop-unique but not R-loop-A3B regions was significantly decreased by A3B perturbation (*P* < 0.001), reflecting the potential involvement of other factors in the promotion of C-to-T conversion in these co-occurring regions and compensation for the effects on DNA sequence in the absence of A3B (Fig. S5G).

**Fig. 4:**
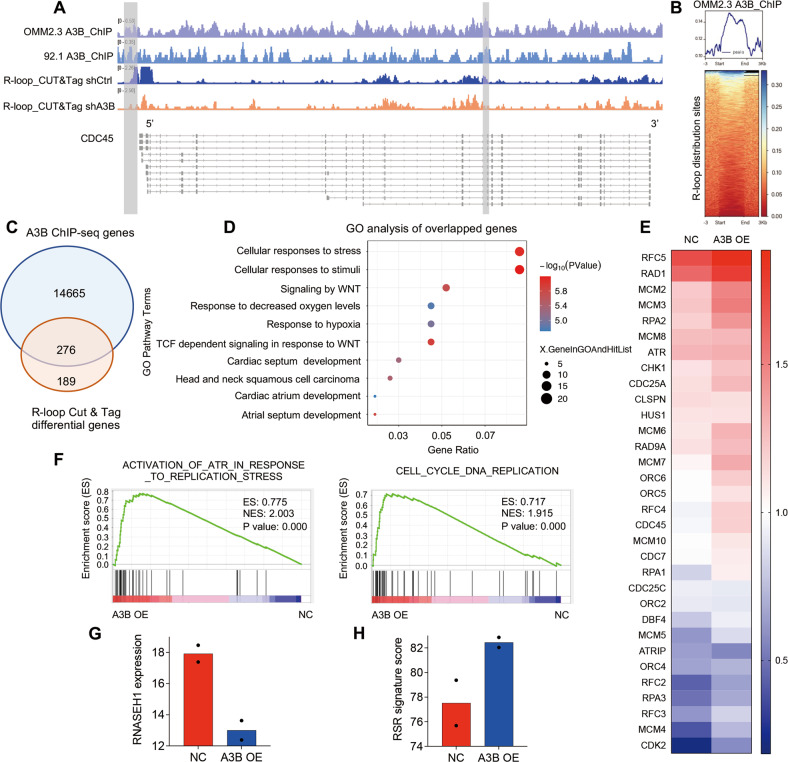
A3B coordinates with R-loops to regulate the expression of RS-associated genes. **A** Genome browser view of R-loop and A3B signals at example loci. **B** The heatmap presentation of ChIP-seq signals for A3B in regions ±3 Kb from R-loop centers. **C** Venn diagram shown genome-wide co-occurrence across A3B target genes (blue) and R-loop-prone differential genes on A3B knock down (orange). **D** GO analysis of overlapping genes in (**C**). **E** Heatmap of gene expression of MEL290 RNA-seq. **F** GSEA pathway enrichment analysis of differential genes obtained by transcriptomic sequencing of stable transfection cell lines of MEL290. NES: normalized enrichment score. **G** Bar charts of RNASEH1 expression based on RNA-seq of A3B overexpression. **H** Bar charts of RSR signature score based on RNA-seq of A3B overexpression.

In fact, we found that both A3B and R-loop peaks occurred at the *Cell division cycle protein 45* (*CDC45)* gene, which is important for initiation of DNA replication [34] (Fig. 4A). In addition, an aggregated distribution of A3B was observed at R-loop center locations (Fig. 4B). Among the 465 genes that R-loops peaks were altered by A3B knockdown, 276 of them were also detected with A3B ChIP-seq binding signals (Fig. 4C). Subsequent gene ontology analysis of the overlapped genes indicated involvement in stress response regulation (Fig. 4D), further validating our results in Fig. 3E.

We also conducted transcriptomic sequencing in control and A3B-depleted melanoma cell lines. In 92.1 cells, 183 differentially expressed genes were detected in response to A3B depletion, among which 27 and 156 were up- and down-regulated, respectively. In contrast, A3B overexpression resulted in elevated expression of 379 genes in MEL290 cells. DNA replication and stress pathways were significantly altered by A3B perturbation (Figs 4E, F and Fig. S5H), possibly through a mechanism involving the regulation of RNASEH1 (Fig. 4G). To further assess the influence of RS by A3B, we employed a set of RSR signature genes as reported previously [35]. The RSR signature score was markedly increased by A3B overexpression, confirming exacerbated RS (Fig. 4H).

### A3B overexpression confers R-loop dependent sensitivity to ATRi/Chk1i in melanoma cells

**Fig. 5:**
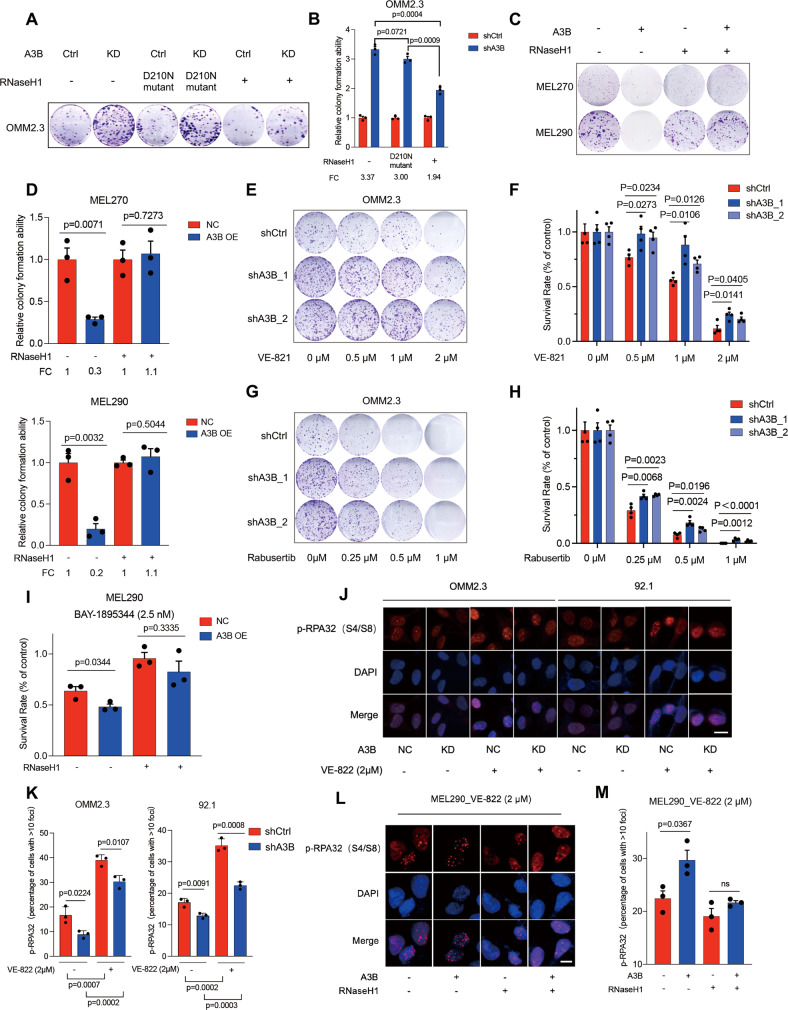
Inhibition of R-loops abrogates A3B-mediated sensitivity to ATR/Chk1 inhibitors. **A** Images of representative colony formation assays of OMM2.3 cells in each condition. **B** Bar chart displayed the quantification of colony formation assay of (**A**) (*n* = 3 biological replicates, mean + SEM, unpaired t-test). FC, fold change. **C** Images of representative colony formation assays of MEL270 and MEL290 with or without RNASEH1 and/or A3B expression. **D** Bar chart displayed the quantification of colony formation assay of MEL270 and MEL290 with or without RNASEH1 and A3B expression (*n* = 3 biological replicates, mean + SEM, unpaired t-test). **E** Images of representative colony formation assays of OMM2.3 cell lines treated with ATRi VE-821. **F** Survival rate of OMM2.3 cell lines treated with VE-821 (*n* = 4 biological replicates, mean + SEM, unpaired t-test). **G** Images of representative colony formation assays of OMM2.3 cell lines treated with Chik1i rabusertib. **H** Survival rate of OMM2.3 cell lines treated with rabusertib (*n* = 4 biological replicates, mean + SEM, unpaired t-test). **I** Survival rate of MEL290 cell lines represented by cell viability treated with 2.5 nM BAY-1895344 for 72 h (*n* = 3 biological replicates, mean + SEM, unpaired t-test). **J** A3B knockdown and control cells were treated with 2 μM ATRi VE-822 or DMSO for 24 h. Representative images of each condition were shown. Scale bar, 20 μm. **K** Percentage of cells with more than 10 p-RPA32 foci in (**J**) was analyzed (*n* = 3 biological replicates, mean + SEM, unpaired t-test). **L** MEL290 cells with A3B and/or RNASEH1 overexpression were treated with 2 μM VE-822 for 24 h. Representative images of each cell lines were shown. Scale bar, 10 μm. **M** Percentage of cells with more than 10 p-RPA32 foci in (**L**) was analyzed (*n* = 3 biological replicates, mean + SEM, unpaired t-test).

We sought to evaluate the functional consequences of A3B on R-loops in melanoma cells. Compared with control cells, the colony area was increased in A3B-depleted cells about 3-fold (Fig. 5A, B and Fig. S6A, B), but significantly reduced in A3B-overexpressing MEL270 (*P* = 0.0071) and MEL290 (*P* = 0.0032) cells (Fig. 5C, D). A3B suppression of colony number was substantially attenuated by the overexpression of RNASEH1 (Fig. 5A–D). These results demonstrated the critical role of R-loop status in A3B-mediated melanoma cell survival. Given the function of A3B and R-loops in RSR activation, we next tested their impact on sensitivity to ATRi/Chk1i, which lead to mitotic catastrophe and tumor suppression [36]. The survival of melanoma cells under of ATRi (VE-821/VE-822) or Chk1i (Rabusertib) treatment was significantly improved by A3B depletion, indicating an essential role of A3B in conferring sensitivity to these drugs (Fig. 5E–H). In contrast, overexpression of A3B resulted in significant reduction of melanoma cell survival under the treatment of VE-822 (Fig. S6C). We also tested the effects of BAY-1895344, a potent and selective ATRi that was currently under clinical evaluation [37]. Intriguingly, the A3B-mediated sensitivity to BAY-1895344 appeared to be R-loop-dependent because the addition of RNASEH1 almost abolished these phenotypes (Fig. 5I). Furthermore, the measurement of p-RPA32 (S4/S8) foci in control and A3B-depleted cells showed that the percentage of p-RPA32 positivity was significantly reduced by A3B ablation under ATRi (VE-822) treatment (OMM2.3: *P* = 0.0107; 92.1: *P* = 0.0008) (Fig. 5J, K). We also noticed that A3B depletion inhibited the baseline level of p-RPA32 foci (OMM2.3: *P* = 0.0224; 92.1: *P* = 0.0091) (Fig. 5J, K). Consistent with the observations in Fig. 5J, K, RNASEH1 substantially inhibited the formation of p-RPA32 foci that was augmented by A3B overexpression (Fig. 5L, M). However, it was unclear whether the A3B-mediated drug sensitivity was specific to ATRi/Chk1i [38, 39]. To address this, we tested a variety of conventional chemotherapy drugs for melanoma treatment, including cisplatin, carboplatin and dacarbazine. We found that the sensitivities to these selected drugs remained unchanged in control and A3B-depleted melanoma cells **(**Fig. S6D–S6K). These results demonstrated that, at least in our scenario, the A3B-mediated drug sensitivity was exclusive to ATRi/Chk1i.

### The association between A3B and R-loops predicts levels of RSR and patient survival

**Fig. 6:**
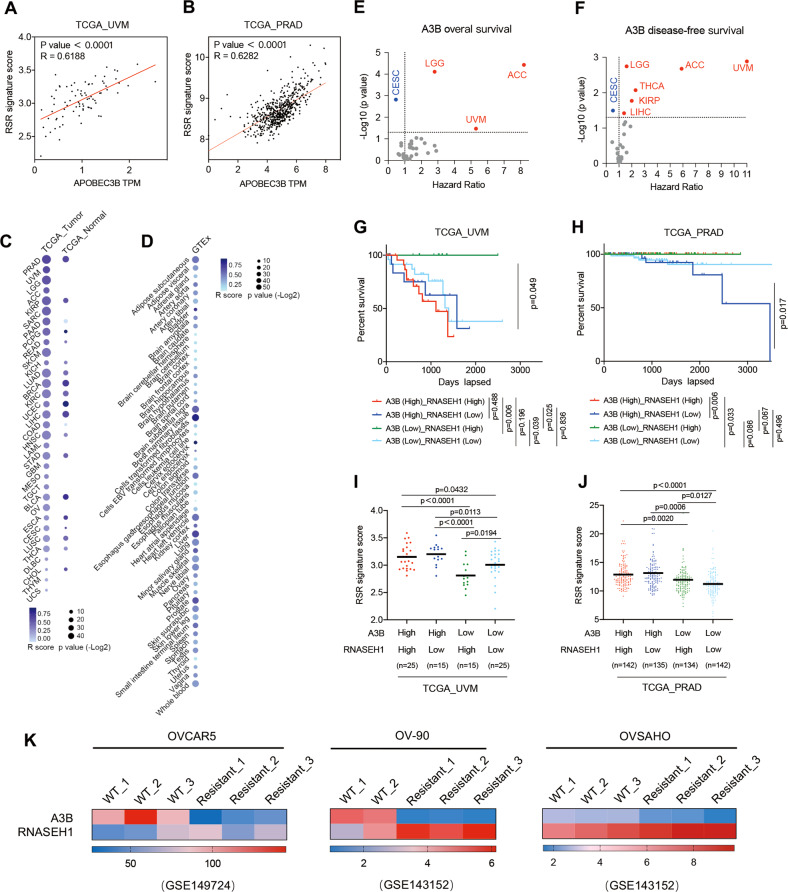
The association of A3B and R-loop predicts RSR levels and patient survival. Pearson correlation of A3B expression and RSR signature score in **A** UVM and **B** PRAD patients. Pearson correlation analysis between A3B and RS in **C** TCGA Cohort and **D** GTEx Cohort. **E** Overall survival and **F** disease-free survival of A3B were examined in TCGA Cohort. Hazard Ratio were calculated based on Cox proportional hazards model. Those with HR > 1 are marked in red, those with HR < 1 are marked in blue. Kaplan-Meier curve of overall survival in **G** UVM and **H** PRAD patients with A3B high/low and RNASEH1 high/low expression (log rank test). RSR signature score in **I** UVM and **J** PRAD patients with A3B high/low and RNASEH1 high/low expression. Mean values are indicated by black lines (unpaired t-test). **K** A3B and RNASEH1 expression of wildtype (WT) and Chk1i resistant cell lines in GSE149724 and GSE143152 datasets.

To explore the clinical relevance of A3B for RSR, we measured RSR signature scores using The Cancer Genome Atlas (TCGA) datasets. Pearson correlation analysis revealed that A3B levels were positively correlated with RSR signature scores in both UVM and prostate adenocarcinoma (PRAD) datasets (Fig. 6A, B). Similar correlations were found in various other cancer types (Fig. 6C). Studies in tumour-adjacent (TCGA_Normal) and normal tissues (GTEx) also exhibited a positive correlation between A3B and RSR, but to a lesser extent (Fig. 6C, D, Table S2), indicating that the association of A3B with RS was not limited to melanoma. In addition, the expression of A3B appeared to be a negative prognostic marker for the prediction of patient survival in a variety of cancer types (Fig. 6E, F, Table S3). The contribution of R-loops to A3B-mediated survival correlation was further assessed by stratifying patients into four groups based on A3B and RNASEH1 levels. The group of patients with low A3B but high RNASEH1 levels exhibited the longest survival durations compared with the other three groups (Fig. 6G, H, Table S4). This indicates that higher R-loop levels are associated with poorer prognosis in UVM and PRAD patients when A3B expression is low. Accordingly, the calculation of RSR signature scores for the four patient clusters demonstrated that the A3B-high but RNASEH1-low group produced the highest RSR score, in contrast to the lowest score produced by the A3B-low but RNASEH1-high group (Fig. 6I, J, Table S5). To further investigate the roles of A3B and R-loops in ATRi/Chk1i sensitivity, we analyzed two sets of data with ChK1i responsiveness (GSE149724 and GSE143152). To our expect, we observed an expression pattern of high A3B but low RNASEH1 in prexasertib (Chk1i) sensitive ovarian cancer cell lines, in contrast to that of prexasertib-resistant cell lines (Fig. 6K). These findings further supported the notion that A3B and R-loop levels were associated with the strength of RSR and could be used to predict patients’ responses to ATRi/Chk1i.

## Discussion

A number of R-loop regulators are related to DNA damage modulation, including RPA [30], BRG1 [40] and BRCA2 [41]. Here, we report the discovery of A3B as a novel binding partner and regulator of R-loops, linking DNA editing family members to the modulation of R-loop. C-to-T mutagenesis is a critical feature of A3B function in cancer, and the kataegic mutational signature attributed to A3B is commonly observed [4, 6]. A3B mutagenesis occurs on the lagging-strand template during DNA replication or at stalled replication forks [42, 43]. However, it is unclear whether and how this mutagenic activity is involved in A3B-mediated RS. We found that A3B interacted with R-loops and was enriched in the proximity of R-loop regions. We, therefore, speculated that such recruitment might affect the C-to-T mutation rate at R-loop-A3B loci. Indeed, A3B depletion led to marked reduction of C-to-T ratio across R-loop loci, in agreement with the active role of A3B to catalyze C-to-T conversion at R-loop regions. Intriguingly, the C-to-T ratio at R-loop-A3B co-occurrent loci was largely retained when A3B was depleted compared with other regions, suggesting the involvement of other C-to-T mutagenic factors to complement the role of A3B at the co-occurrent regions. It seemed that A3B was more likely to function as a “road blocker” instead of “mutator” at R-loop loci to orchestrate the expression of neighboring genes. Though the mechanistic details were unknown, the recruitment of other binding components to R-loop may be involved as indicated by our IP-MS analysis.

Mounting evidence demonstrates that A3B is clinically significant in cancer. A3B overexpression was shown to confer resistance to drugs such as doxorubicin and tamoxifen [44, 45]. But on the other hand, A3B may enhance the sensitivity to immunotherapies. Studies have shown that patients with high levels of A3B are more likely to respond to immune checkpoint blockade [9, 46]. These apparently paradoxical observations highlight the fact that the function of A3B is fine-tuned in different contexts, and A3B alone is not sufficient to predict the overall benefits to patients. Perturbation of the ATR-Chk1 pathway induces deleterious consequences, thus can be exploited to treat cancer in which RS levels are always augmented because of oncogene hyperactivation [47, 48]. Accordingly, ATRi or Chk1i exhibit promising antitumor functions in pre-clinical models and are currently under evaluation in clinical trials [37, 49–51]. Our data revealed that both RS and sensitivity to ATRi elicited by A3B was dependent on R-loop formation because these effects could be largely alleviated by overexpression of RNASEH1. In this regard, A3B together with R-loop status could better define the level of RS and ATRi/ChK1i sensitivity. Recently, CRISPR-based screening has revealed several potential biomarkers, including ATM, ARID1A, POLE3/4, CDK8 and SMG1 [49, 52–55]. Most of them are related to DNA damage and cell cycle functions that ATR is primarily involved in. From the point of RS regulation, our results demonstrated that the combined expression pattern of A3B and RNASEH1 (A3B-high RNASEH1-low) instead of A3B alone may represent a robust metric to predict the therapeutic benefits to ATRi. And the vulnerability to ATRi may not be limited to melanoma but may also be applicable to other cancer types.

Taken together, we have shown mechanistic insights into A3B-coordinated R-loop, which exerts RS-promoting activity in cancer. ATRi sensitivity elicited by A3B expression and R-loop status may be exploited to guide ATRi treatment of cancer patients.

## Materials and methods

### Patient samples

A total of 67 human ocular melanoma tissues, 14 human nevi tissues and 5 adjacent normal tissues were collected for IHC from Shanghai Ninth People’s Hospital, Shanghai Jiao Tong University School of Medicine from 2007 to 2017. Patients’ clinical and demographic features are detailed in Table S6.

### Immunohistochemistry (IHC)

IHC staining of tissue slides was performed by Servicebio (Wuhan, China). Tissues were deparaffinized and rehydrated through an alcohol series, followed by antigen retrieval with sodium citrate buffer. Then tissue sections were blocked with 3% bovine serum albumin (BSA) 30 min at room temperature and then incubated with anti-A3B (abcam, Cambridge, England, ab184990, 1:100) antibody at 4 °C overnight. Finally, the tissues were covered with horseradish peroxidase (HRP) labeled secondary antibody and incubated at room temperature for 50 minutes. All immunostained slides were scanned on 3D Histech Quant Center (3D Histech, Hungary), and computerized image analysis was performed by Halo (Indica labs, USA). Immunostaining for A3B was analyzed in adjacent normal tissues, nevi tissues and melanoma tissues using percentage of positive cells and histochemistry score (H-score). H-score was determined based on the proportion of positive cells and the intensity of nuclear staining as previously described [56]. H-score = ∑(pi×i)= (percentage of cells of weak intensity ×1) + (percentage of cells of moderate intensity ×2)+ (percentage of cells of strong intensity ×3). In the formula, pi represents the percentage of positive cells in the slide; i represents the intensity of A3B staining.

### Cell culture

The adult retinal pigment epithelium cell line ARPE-19 was obtained from the Cell Bank/Stem Cell Bank (Chinese Academy of Sciences). Human cutaneous melanocyte cell line PIG1 was obtained from the Department of Ophthalmology, Peking University Third Hospital. The human UVM cell lines OMM1, OMM2.3, MEL285 and MEL290 and conjunctiva melanoma cell lines CRMM1, CRMM2, and CM2005.1 were kind gifts from Prof. Martine J. Jager (Leiden University Medical Center, Leiden, The Netherlands). The human UVM cell line MUM2B and 92.1 were kindly supplied by Prof. John F. Marshall (Tumor Biology Laboratory, Cancer Research UK Clinical Center, John Vane Science Centre, London, UK). HEK293T human embryonic kidney cell line was purchased from the American Type Culture Collection (Manassas, VA, USA). The cell lines used in this study were authenticated by short tandem repeat (STR) profiling. Human HEK293T, A375 and A2058 cells was cultured in Dulbecco’s modified Eagle’s medium (DMEM; Thermo Fisher Scientific, Massachusetts, USA). Other cells were cultured in RPMI1640 medium (Gibco). All mediums are supplemented with 10% fetal bovine serum (FBS; Thermo Fisher Scientific) and 1% penicillin/streptomycin (P/S; Thermo Fisher Scientific). All cells were cultured in a 37 °C humidified incubator containing 5% CO_2_.

### RNA extraction and quantification

RNA purification was performed using EZ-press RNA purification kit (EZBioscience, CA, USA, B0004DP) according to the manufacturer’s guidelines. Then, cDNA synthesis was achieved using the PrimeScript RT Master Mix (Takara, Kyoto, Japan). Finally, real-time quantitative PCR (RT-qPCR) was carried out using the SYBR Green qPCR Master Mix (Applied Biosystems). mRNA expression values were calculated using the ΔΔCt method and GAPDH gene as a control. A detailed list with primers used in the present study is provided in Table S7. CT values were shown in Supplementary Material 1.

### Western blotting

Total cell lysates were prepared in RIPA lysis buffer (Beyotime, Shanghai, China, P0013B). Protein samples were separated by sodium dodecyl sulfate-polyacrylamide gel electrophoresis (SDS-PAGE) and transferred to polyvinylidene fluoride (PVDF) membranes (Millipore). After blocking with 5% non-fat milk for 1 h at room temperature, the membranes were incubated with anti-p-RPA32 (BETHYL, TX, USA, A300-245A, 1:2000), anti-RPA70 (CST, MA, USA, 2267 S, 1:1000), anti-Flag (ABclonal, Wuhan, China, AE063, 1:1000), anti-A3B (Abcam, ab184990, 1:1,000), anti-GFP (Invitrogen, CA, USA, A-11122, 1:1000), anti-Myc (santa cruz, TX, USA, sc-40, 1:1000) or anti-V5 (Abcam, ab15828, 1:2000) antibodies overnight at 4 °C and then with the appropriate secondary antibodies conjugated to a fluorescent tag (Invitrogen) for 1 h at room temperature. Anti-β-Actin (Proteintech, IL, USA, 66009-1-Ig, 1:20000) antibody served as the loading control. The immunoblots were recorded with the Odyssey infrared imaging system (LI-COR Biosciences, NE, USA). Full and uncropped western blots were shown in Supplementary Material 2.

### Co-immunoprecipitation (co-IP)

Cells were pelleted and washed with PBS, and then lysates were prepared in 500 μL lysis buffer containing 120 mM NaCl, 20 mM Tris-Cl, 2 mM EDTA, 1% NP40 and 5% Glycerol supplemented with 1× protease inhibitor cocktail (Roche, NJ, USA). Anti-S9.6 antibody (Kerafast, MA, USA, ENH001, 1:100) or normal mouse IgG (Santa cruz, sc-2025, 1:10000) was incubated with the cell lysates overnight at 4 °C, after which 30 μL protein A magnetic beads (CST, #73778) were added and incubated for 2 additional hours. Then, the magnetic beads were washed three times with lysis buffer. For IP-MS analysis, 100 μL glycine solution was used for each tube to elute the protein complexes from the beads, while 1 × SDS loading buffer (NCM, Suzhou, China) was used per sample for SDS-PAGE analysis.

### Transfection and virus packaging

Three shRNA sequences targeting A3B were cloned into the pGIPZ-TurboGFP-puro vector. The A3B targeting sequences were: 5’-TAAAGTTGAAAGTGAATGTGTT-3’(shA3B_1); 5’-TTAAAGTTGAAAGTGAATGTGG-3’ (shA3B_2). The full length of human A3B (isoform NM_004900.5), Myc_RNASEH1_EGFP (isoform NM_002936.3) and CDD1/2_Flag were cloned into the pHAGE-puro vector, respectively for overexpression of A3B and RNASEH1. Primers used for cloning are shown in Table S8. PolyJet DNA In Vitro Transfection Reagent (SignaGen, MD, USA) was used for plasmid transfection following the manufacturer’s instructions. After lentiviral packaging with HEK293T cells, cells were infected with lentiviruses and selected by incubation with 2 μg/mL puromycin for 3 days. For transfection of RNASEH1 D210N mutant construct, cells were transfected with ppyCAG_RNASEH1_D210N_V5 plasmid (Addgene, MA, USA, 111904) and selected with 300 μg/mL Hygromycin B (Selleck, TX, USA, S2908) for 3 days.

### Colony formation

A volume of 2 mL of complete medium containing 1000 cells was seeded in each well of a 12-well plate and incubated for about 2 weeks. For drug sensitivity colony formation assay, cells were treated with the indicated drug concentrations every 48 h. Once colonies formed in DMSO control conditions, plates were fixed with 4% paraformaldehyde, stained with crystal violet and the number of colonies was counted. Colony formation efficiency indicated by area of colonies treated relative to area of control colonies was used to measure survival between wells. RSR inhibitors and chemotherapy drugs for drug sensitivity assay were purchased from Selleck: VE-821 (S8007) targeting ATR, VE-822 (S7102) highly selective and potent derivatives of VE-821, Rabusertib (S2626) targeting Chk1, Cisplatin (S1166), Carboplatin (S1215), Dacarbazine (S1221). The quantifications presented were obtained from at least three independent biological replicates.

### Cell viability

To determine cell viability, MEL290 cells were seeded in 96-well plates at a density of 1000 cells per well and treated with 2.5 nM BAY-1895344 (Selleck, S8666) or 2 μM VE-822. After incubation with 10 μL CCK-8 reagent (Dojindo Laboratories, Kumamoto, Japan) per well, the absorbance was measured at a wavelength of 450 nm at the indicated time points. The data were recorded and analyzed. The quantifications presented were obtained from three independent biological replicates.

### Immunofluorescence (IF)

S9.6 IF was performed essentially as described [57]. Briefly, cells were fixed with 100% ice-cold methanol, blocked with 2% BSA for 1 h at room temperature and incubated with S9.6 (Kerafast, ENH001; 1:200) overnight at 4 °C. Then coverslips were washed three times in PBS and incubated with Alexa Fluor 594 secondary antibody (Invitrogen, #A21203; 1:1000) for 1 h at room temperature. Finally, cells were stained with DAPI and mounted in ProLong Gold AntiFade reagent (Invitrogen). For DNA RS assessment by p-RPA32 (S4/S8) immunostaining, cells adhering to a glass slide were fixed with 4% paraformaldehyde (Biosharp, Beijing, China, 70071800) for 15 min, permeabilized with 0.1% Triton X-100 for 15 min and then blocked with 2% BSA solution for 1 h at room temperature. After incubation with primary antibody against p-RPA32 (S4/S8) (BETHYL, A300-245A, 1:2000) overnight, the cells were washed three times with PBS and subsequently incubated with Alexa Fluor 594 secondary antibody (Invitrogen, A11012, 1:1000) for 1 h. The coverslips were then mounted with ProLong Gold mounting medium with DAPI (Invitrogen, P36931) and observed under an inverted fluorescence microscope (Nikon, Tokyo, Japan). Images were acquired with Leica TCS SP8 confocal microscope (Leica, Mannheim, Germany). Nuclear intensity was quantified using FIJI (ImageJ) image processing package. The nuclear mean gray value for S9.6 (>100 cells) was measured for each condition. Percentage of cells with more than 10 p-RPA32 foci was quantified. The results presented were obtained from three independent biological replicates; at least 100 cells were measured per replicate.

### RNA-seq

RNA sequencing was performed by Novogene (Beijing, China). Total RNA was harvested from MEL290 cells overexpressing A3B and/or RNASEH1 and 92.1 cells transfected with A3B knockdown or empty vector using Trizol. The integrity of the RNA was assessed by Agilent 2100 bioanalyzer (Thermo Fisher Scientific). Approximately 1 μg mRNA from each sample was used for RNA sequencing (Illumina HiSeq PE150 platform). Short reads were aligned to the reference human genome GRCh38 using STAR [58]. Gene expression in terms of Fragments Per Kilobase of transcript per Million mapped reads (FPKM) of genes overlapping with gene annotations in Ensembl release 75 was calculated by Cufflinks [59]. Differential gene expression was determined using the Cufflinks Cuffdiff package [59]. Gene Set Enrichment Analysis (GSEA) was performed to interpret the function of regulated genes using the cancer hallmark gene sets, canonical pathways gene sets and GO biological process gene sets.

### ChIP-seq

ChIP assay was performed as previously described, with minor modifications [60]. Briefly, OMM2.3 and 92.1 (1 × 10^7^ cells) were cross-linked for 10 min at room temperature with 1% formaldehyde-containing medium. The reaction was then halted using a 1× glycine solution. Then cells were pelleted before resuspension in sonication buffer (10 mM Tris-HCl pH 8.0, 1 mM EDTA, 0.5 mM EGTA and 1 × protease inhibitor cocktail). The cellular chromatin was then sonicated to lengths between 100 and 600 bp. 10% chromatin for each ChIP reaction was kept as input DNA. The rest chromatin was then incubated with anti-A3B antibody (abcam, ab184990, 1:50) or normal rabbit IgG (CST, 2729, 1:500) at 4 °C overnight. 30 μL magnetic protein A/G beads (CST) were then added for another 1 h of incubation at 4 °C before going through a series of salt washes. Chromatin was then eluted from the magnetic beads in the elution buffer at 65 °C for 15 minutes while vortexing. The supernatant was removed and treated with RNase A followed by Proteinase K. ChIP DNA was then purified using MinElute PCR Purification Kit (QIAGEN, Dusseldorf, Germany, 28006). The ChIP-seq libraries were constructed by Novogene. Pair-end sequencing of the sample was performed on the Illumina platform (Illumina, CA, USA). Library quality was assessed on the Agilent Bioanalyzer 2100 system. Raw data of fastq format was processed using FastQC 0.11.9 software [61]. Clean reads were aligned to the reference genome GRCh38 using Bowtie2 2.2.5 [62] to generate bam files. After removing PCR duplicates with Sambamba 0.8.2 software [63], we used MACS2 2.2.7.1 to identify regions of IP enrichment over the background [64]. A p-value threshold of 0.05 was used for all data sets. Deeptools 3.5.1 was used to transfer deduplicated bam file to BigWig format and to generate heatmaps and density plot figures [65]. ChIPseeker package and ChIPpeakAnno package were used for peak annotation and visualization [66, 67].

### CUT&Tag

CUT&Tag assay was performed using Hyperactive In-Situ ChIP Library Prep Kit for Illumina (Vazyme, Nanjing, China, TD901-TD902) following the manufacturer’s protocol. Briefly, 5 × 10^5^ OMM2.3 cells transfected with V5 tagged RNASEH1_D210N were collected. After binding to concanavalin A–coated magnetic beads (ConA beads), bead-bound cells were incubated with anti-V5 antibody (Abcam, ab15828, 5ug/1:10) or normal rabbit IgG (CST, 2729, 0.5ug/1:100) for 2 h at room temperature. After brief wash with dig-wash buffer, cells were then incubated with goat antirabbit secondary antibody (Abcam, ab6702, 1:100) for 1 h at room temperature. When antibody binding procedures were finished, the bead-bound cells were then mixed with hyperactive pA-Tn5 transposon and tagmentated with tagmentation buffer. Tagmentated DNA was then extracted and amplified to form the sequencing-ready libraries. After the PCR reaction, libraries were purified with the DNA clean beads (Vazyme, N411) and library quality was assessed on the Agilent Bioanalyzer 2100 system. The clustering of the index-coded samples was performed on a cBot Cluster Generation System using TruSeq PE Cluster Kit v3-cBot-HS (Illumina). The library preparations were sequenced on the Illumina Novaseq platform at Novogene and 150 bp paired-end reads were generated. Raw data was first processed using FastQC 0.11.9. Clean data was obtained by removing adapters using Cutadapt 4.1 with default parameters [68]. Paired-end reads were aligned to genome GRCh38 using Bowtie2 2.2.5 as follows: bowtie2 -local -very-sensitive -no-mixed -no-discordant -phred33 -I 10 -X 700. After removing the duplicated reads with Picard 2.27.3 (https://broadinstitute.github.io/picard/), we calculated the read coverage and depth with Samtools 1.15.1 [69]. To ensure that our data were of high quality and reproducibility, we filtered data with minQualityScoreå 2 using Samtools 1.15.1. Then we filtered and kept the mapped read pairs. Bedtools was used to convert files into bed file format and calculate reads coverage at the genome level. Peak calling was performed with SEACR 1.3 [70]. EdgeR package was used to get differential peaks [71]. BigWig file and reads distributions across genes were acquired using Deeptools 3.5.1. Peak annotation was performed with ChIPseeker and ChIPpeakAnno package as previously described. Gene ontology enrichment analysis was implemented by Metascape (www.metascape.org). In Venn diagrams, numbers represent genes co-occurring between conditions.

### TCGA data analysis

Two TCGA data sets (PRAD and UVM) were used to test the correlation between the selected genes and patient survival. Based on the expression levels of A3B and RNASEH1 and their median expression values, respectively, patients were further discretized as four groups: “A3B High and RNASEH1 High”, “A3B High and RNASEH1 Low”, “A3B Low and RNASEH1 High” and “A3B Low and RNASEH1 Low”. The statistical analysis was performed by the R package “survival” and survival curves were fitted by the survfit function. RSR signature score was calculated based on the median expression levels of the RSR signature gene sets (Table S9) [35]. The somatic mutation signatures of 80 UVM and 472 SKCM patients from TCGA were deciphered using the R package SomaticSignatures [72].

### Statistical analysis

All statistical analyses were performed using GraphPad Prism 7.0 (GraphPad Software, CA, USA). Descriptive values are presented as mean + standard error of the mean (SEM) unless stated elsewhere. Differences between two groups were analyzed by unpaired Student’s t-test while differences among multiple groups were analyzed by one-way analysis of variance (ANOVA) with post-hoc intergroup comparisons with Turkey’s test. When data did not meet the normal distribution, the Mann–Whitney U-test was performed. Test details were indicated in the figure legends. Results were considered statistically significant when *p* < 0.05. Pearson correlation analysis was performed on GEPIA (http://gepia.cancer-pku.cn/).

## Supplementary information

Supplementary Figures and Tables

Supplementary Material

checklist

## Data Availability

The data that support the findings of this study are available in Gene Expression Omnibus (GEO) with accession number GSE218751, GSE218753 and GSE218754.
