# Supplementary material for: APOBEC3B coordinates R-loop to promote replication stress and sensitize cancer cells to ATR/Chk1 inhibitors
Source: Cell Death Dis. 2023 Jun 3;14(6):348. doi: 10.1038/s41419-023-05867-0 (PMC10239489; doi:10.1038/s41419-023-05867-0)
Supplement: Supplementary file 2 — Supplementary Material [file 41419_2023_5867_MOESM2_ESM.pdf]

Supplementary Material 1. CT value of RT-PCR.

CT value Fig. S1a

		A3B	
ARPE-19	19.88	19.82	19.85
PIG1	20.48	20.69	20.9
MEL290	20.87	19.96	21.4
A375	19.96	20.35	20.32
MEL285	20.54	20.58	20.51
MEL202	20.28	20.66	20.47
A2058	18.07	18.84	17.71
CRMM2	17.22	18.42	17.74
MUM2B	18.4	17.86	17.8
MEL270	21.68	21.67	21.88
CRMM1	18.65	19	18.52
92.1	19.65	19.43	19.43
OMM2.3	18.51	18.4	17.87
OMM1	18.74	18.57	18.28

		Actin	
ARPE-19	12.87	13.21	12.95
PIG1	14.63	14.96	14.96
MEL290	13.34	13.44	13.35
A375	14.48	14.4	14.54
MEL285	13.85	13.81	13.74
MEL202	13.8	14.22	13.71
A2058	12.57	13.37	12.94
CRMM2	13.26	12.84	12.23
MUM2B	11.87	12.35	11.9
MEL270	16.41	15.88	16.37
CRMM1	14.42	14.38	14.28
92.1	14.45	14.26	14.48
OMM2.3	13.61	13.52	13.66
OMM1	14.17	13.87	13.68

CT value Fig. S3C

OMM2.3		A3B	
shCtrl	19.07	18.97	19.19
shA3B_1	22.71	22.75	22.63
shA3B_2	22.21	22.61	22.83

OMM2.3		Actin	
shCtrl	14.39	14.76	14.54
shA3B_1	14.5	14.43	14.4
shA3B_2	14.71	14.82	15.2

92.1		A3B	
shCtrl	20.83	20.69	20.97
shA3B_1	23.28	22.67	22.93
shA3B_2	22.68	22.83	22.98

92.1		Actin	
shCtrl	16.31	16.42	16.38
shA3B_1	15.35	15.42	14.92
shA3B_2	15.63	15.7	15.61

Supplementary Material 2. Full and uncropped western blots.

Fig.1d

92.1 OMM2.3

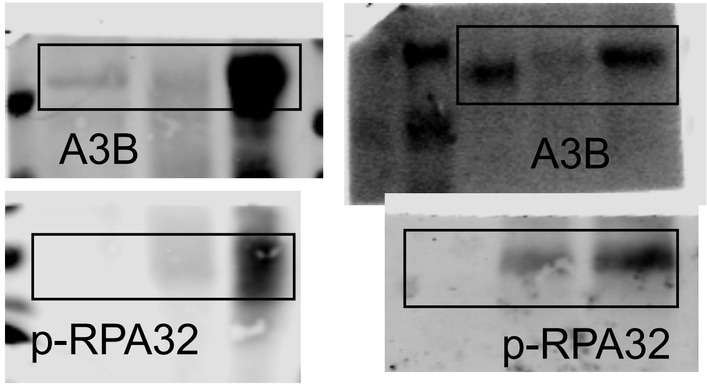

MEL290

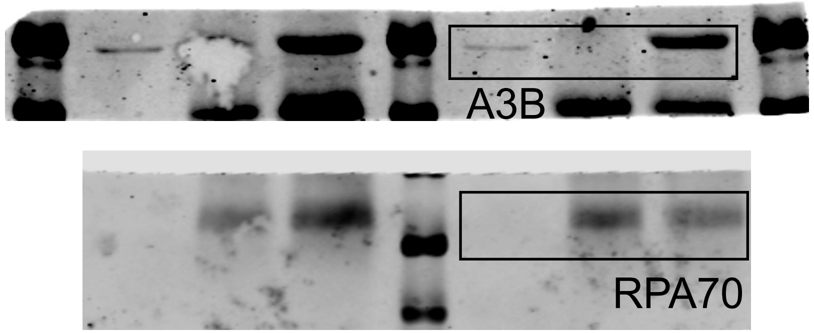

HeLa

A3B
A3B
RPA70
RPA70

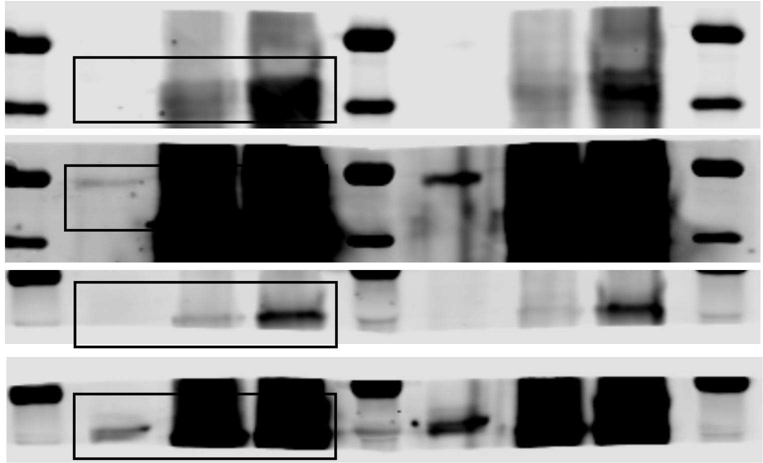

Fig.1f

92.1

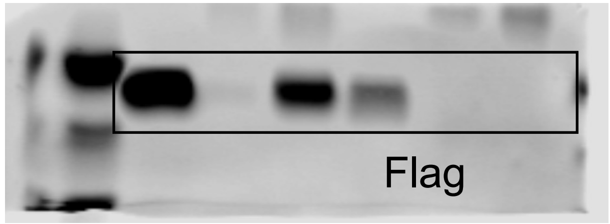

Fig. S3a; S3b

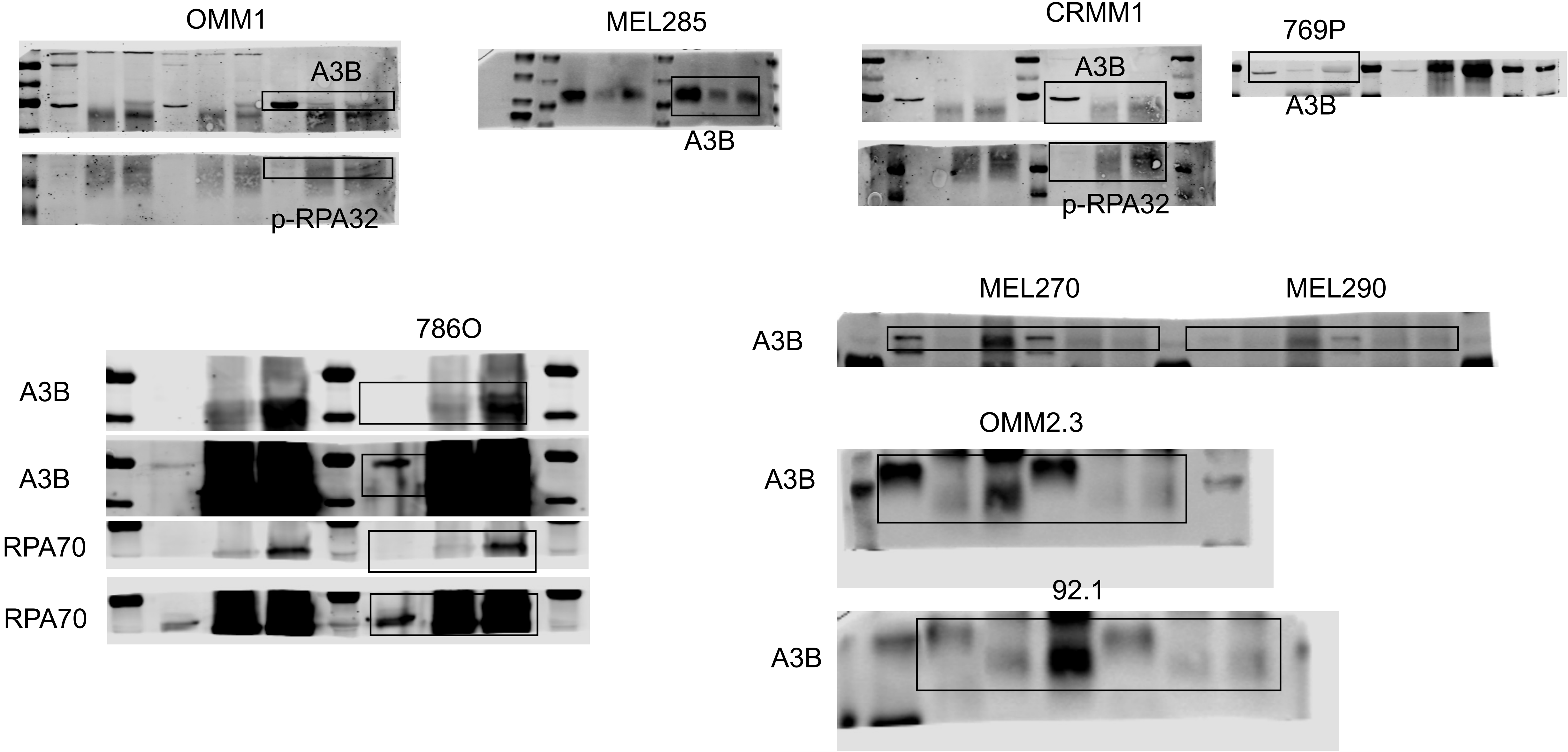
OMM1
A3B
p-RPA32
MEL285
A3B
CRMM1
A3B
p-RPA32
769P
A3B
786O
A3B
A3B
RPA70
RPA70
MEL270
MEL290
A3B
OMM2.3
A3B
92.1
A3B

Fig.S1b

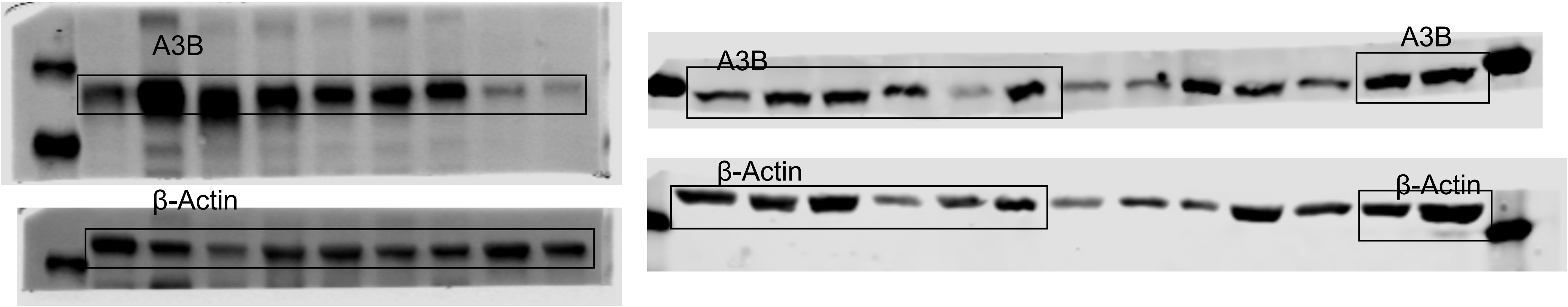

Fig.S3c

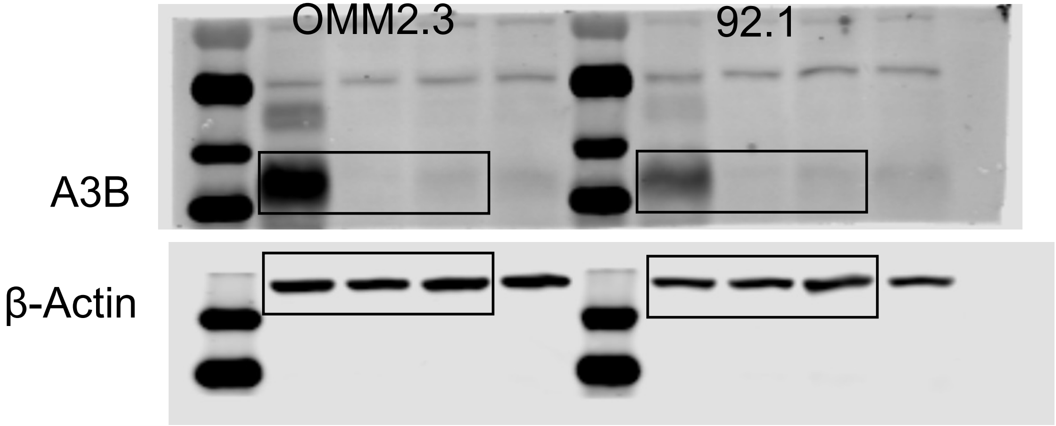

Fig.S3g-S3l

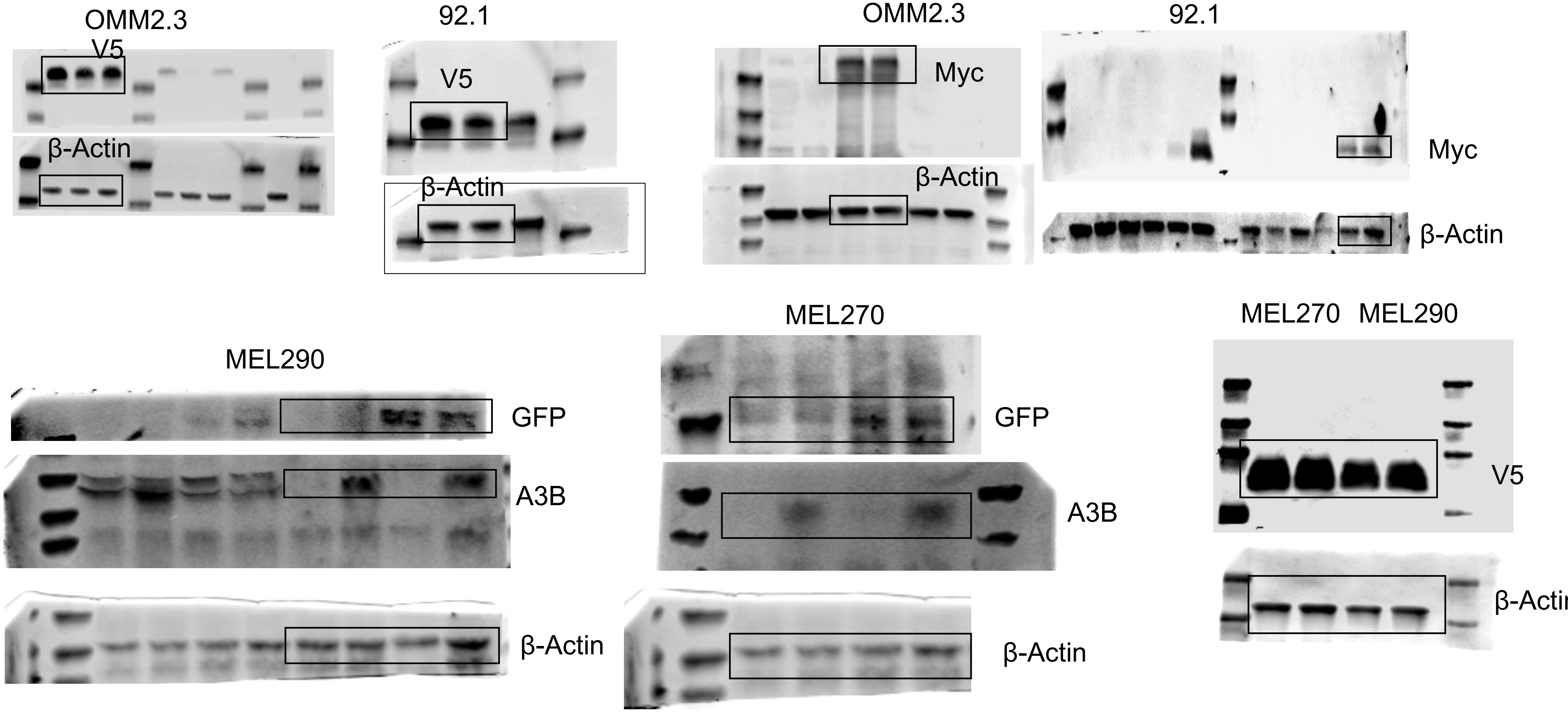
OMM2.3
V5
β-Actin
92.1
V5
β-Actin
OMM2.3
Myc
β-Actin
92.1
Myc
β-Actin
MEL290
GFP
A3B
β-Actin
MEL270
GFP
A3B
β-Actin
MEL270 MEL290
V5
