# Supplementary material for: APOBEC3B coordinates R-loop to promote replication stress and sensitize cancer cells to ATR/Chk1 inhibitors
Source: Cell Death Dis. 2023 Jun 3;14(6):348. doi: 10.1038/s41419-023-05867-0 (PMC10239489; doi:10.1038/s41419-023-05867-0)
Supplement: Supplementary file 1 — Supplementary Figures and Tables [file 41419_2023_5867_MOESM1_ESM.pdf]

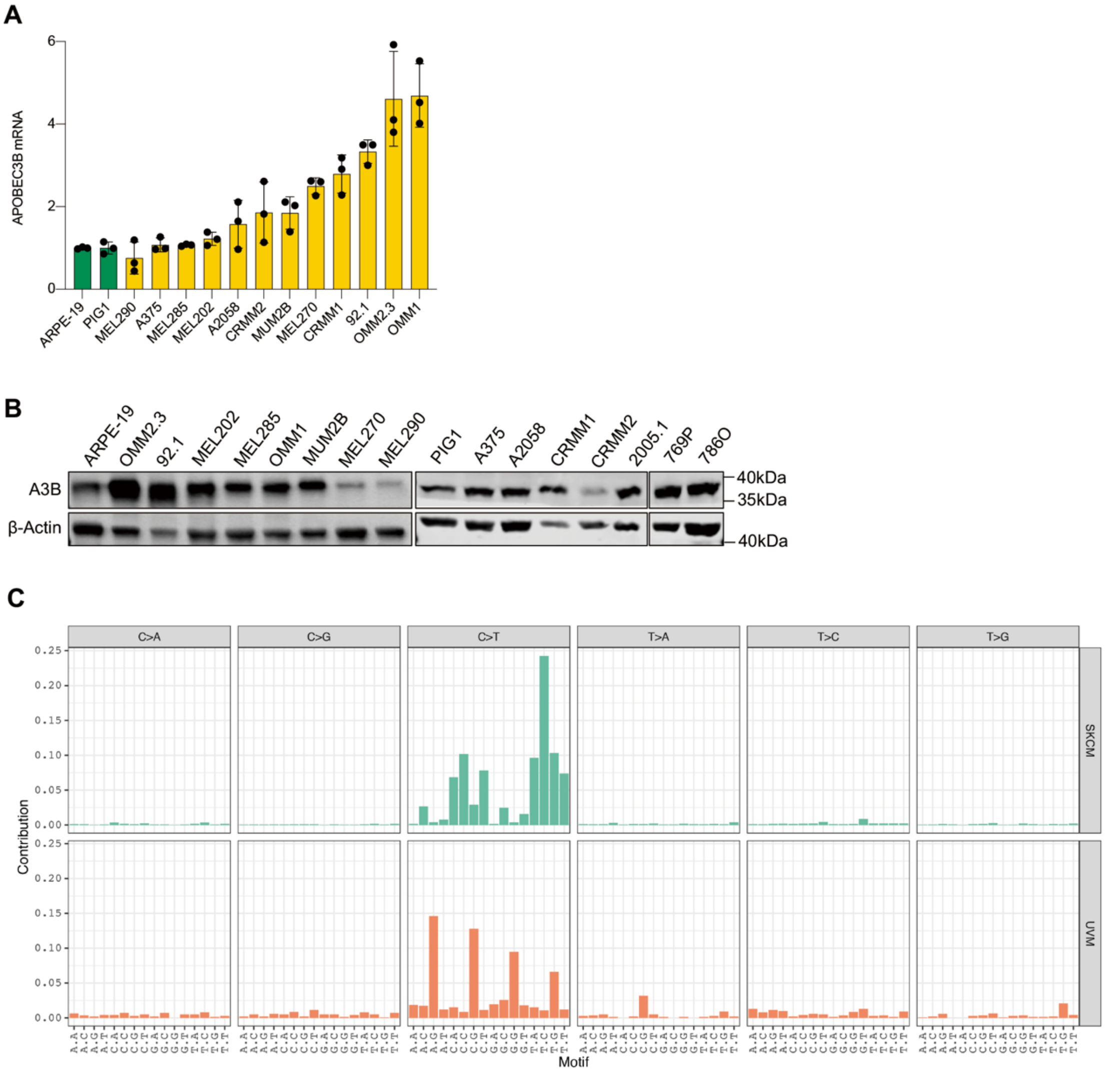

Fig. S1 A3B expression in indicated cell lines.

A A3B mRNA quantification relative to β-actin for the indicated cell lines (n=3 biological replicates, mean±s.e.m). Control normal cell line: ARPE-19, PIG1; UVM: OMM1, OMM2.3, 92.1, MEL270, MUM2B, MEL202, MEL285, MEL290; CoM: CRMM1, CRMM2; SKM: A375, A2058; Renal cancer: 769P, 786O. **B** Immunoblot showing A3B protein levels in the indicated cell lines. **C** Somatic mutation motifs in SKCM and UVM.

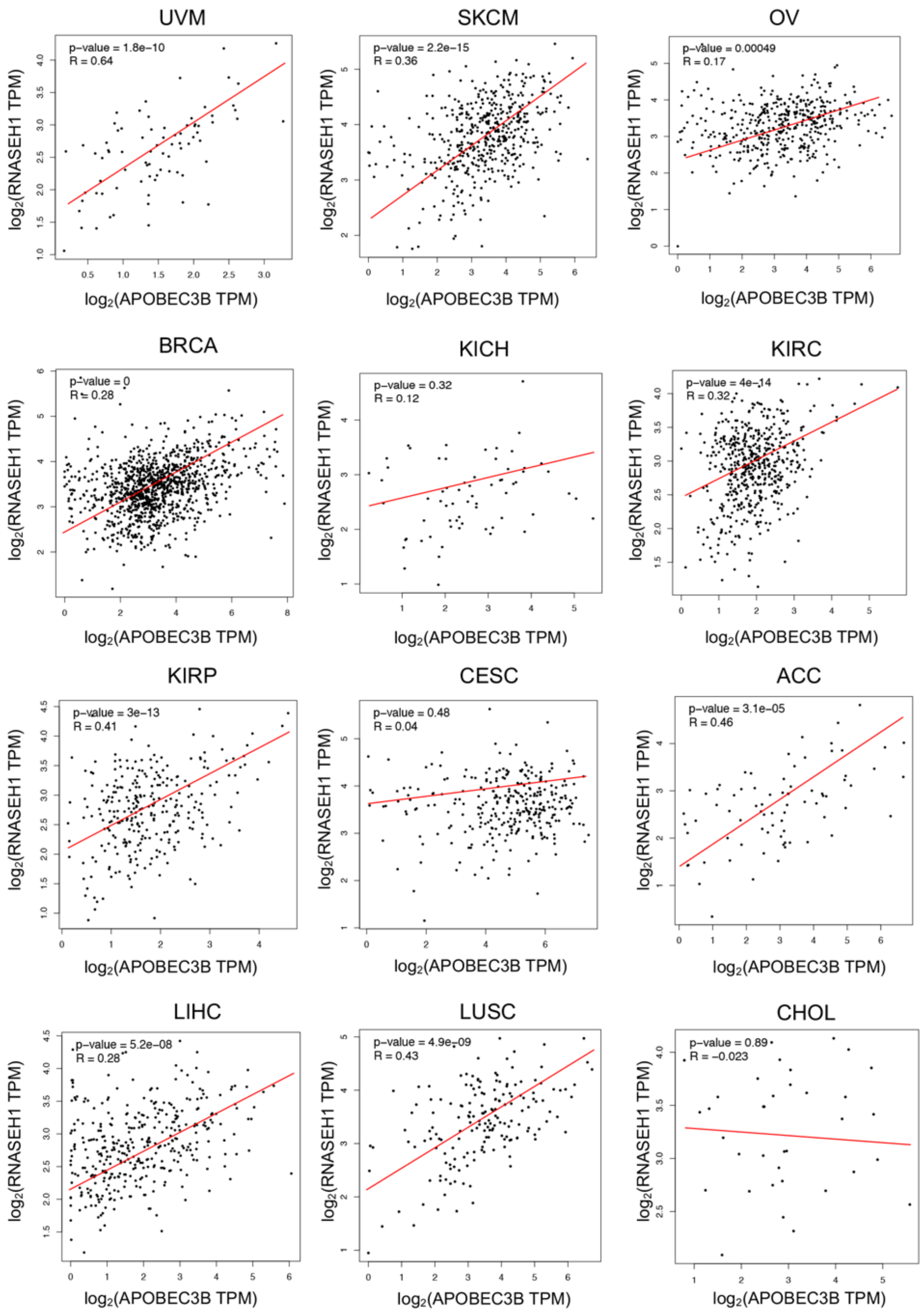

Fig S2. Pearson correlation of A3B and RNaseH1 in multiple cancer types.
UVM, uveal melanoma; SKCM, skin cutaneous melanoma; OV, ovarian serous cystadenocarcinoma; BRCA, breast invasive carcinoma; KICH, kidney chromophobe; KIRC, kidney renal clear cell carcinoma; KIRP, kidney renal papillary cell carcinoma; CESC, cervical squamous cell carcinoma; ACC, adrenocortical carcinoma; LIHC, liver hepatocellular carcinoma; LUSC, lung squamous cell carcinoma; CHOL, cholangiocarcinoma.

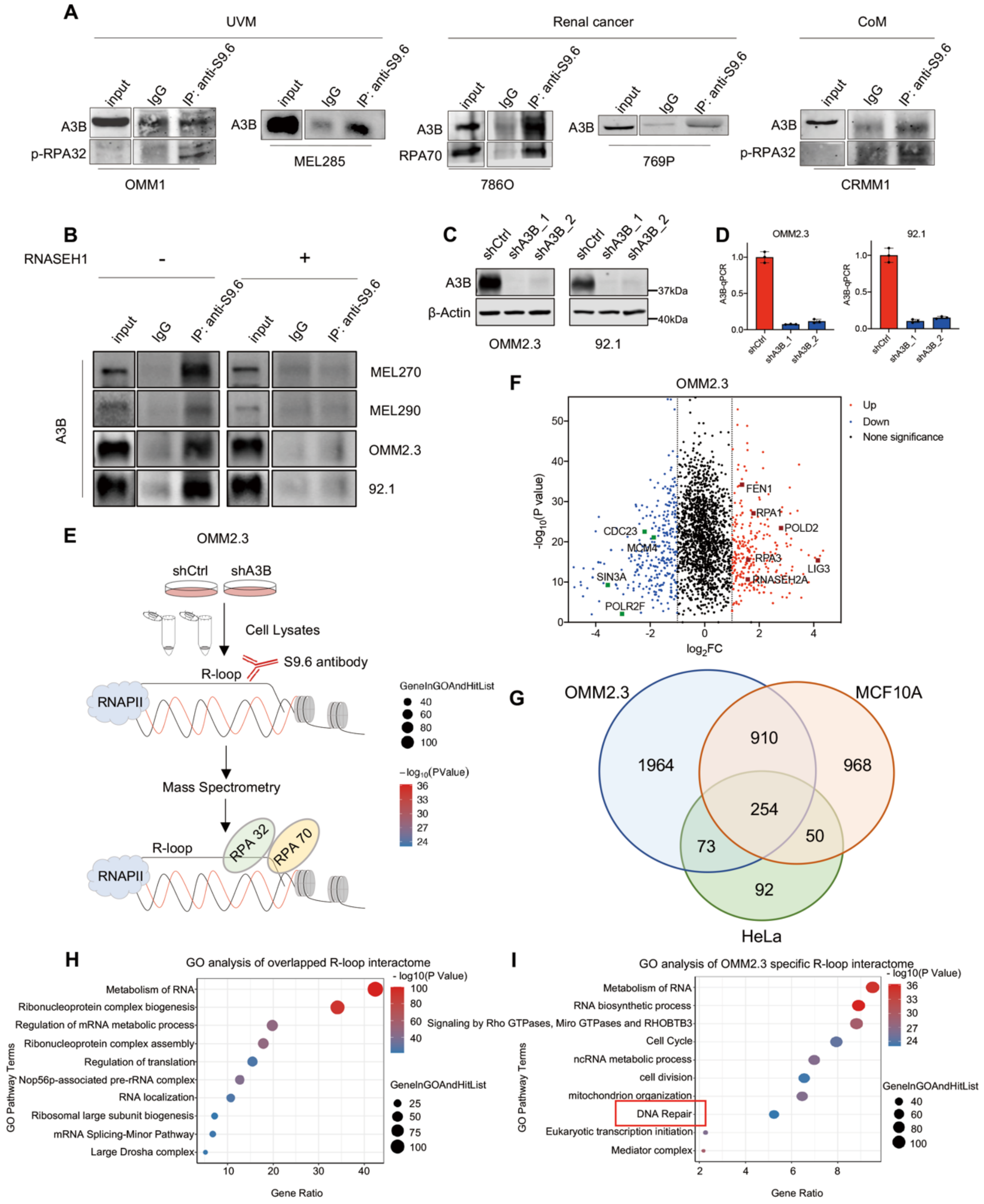

Fig. S3 Co-IP assay.

A Co-IP of R-loop using anti-S9.6 antibody. Cell lysates (1%) were loaded as input. **B** Co-IP of R-loop using anti-S9.6 antibody with RNaseH1 overexpression to confirm R-loop signal specificity. **C** Immunoblot analysis of the knockdown efficiency of A3B in UVM cell lines. OMM2.3 and 92.1 were transfected with shA3B and shCtrl plasmids. **D** A3B mRNA quantification of control (shCtrl) and A3B-depleted (shA3B) cell lines (n=3 biological replicates, mean±s.e.m). **E** Workflow of IP-MS in OMM2.3 cells. **F** Volcano plot of differential proteins except those shown in Fig. 1e in OMM2.3 by IP-MS. P value < 0.05. **G** Venn diagram shown R-loop interactomes in OMM2.3, MCF10A and HeLa cell lines.**H** GO analysis of 254 overlapped R-loop interactors in (**G**). **I** GO analysis of 1964 OMM2.3 specific R-loop interactors.

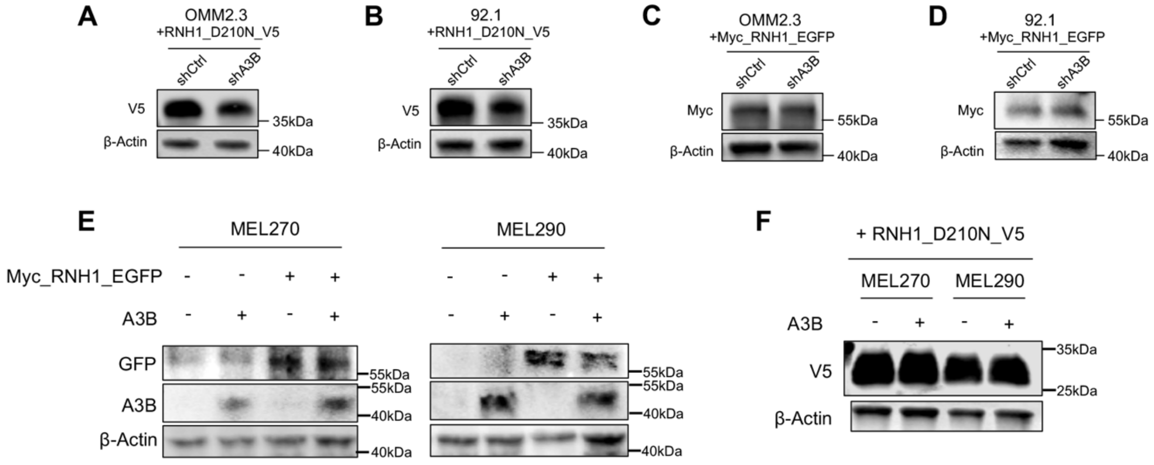

Fig. S4 Validation for stable transfected cell lines.
Immunoblot analysis of the overexpression efficiency of V5-tagged RNH1_D210N in **A** OMM2.3 and **B** 92.1 cell lines. Immunoblot analysis of the overexpression efficiency of Myc-tagged RNH1 in **C** OMM2.3 and **D** 92.1 cell lines. **E** Immunoblot analysis of the overexpression efficiency of A3B and/or EGFP-tagged RNH1 in MEL270 and MEL290 cell lines. **F** Immunoblot analysis of the overexpression efficiency of V5-tagged RNH1_D210N in MEL270 and MEL290 cell lines.

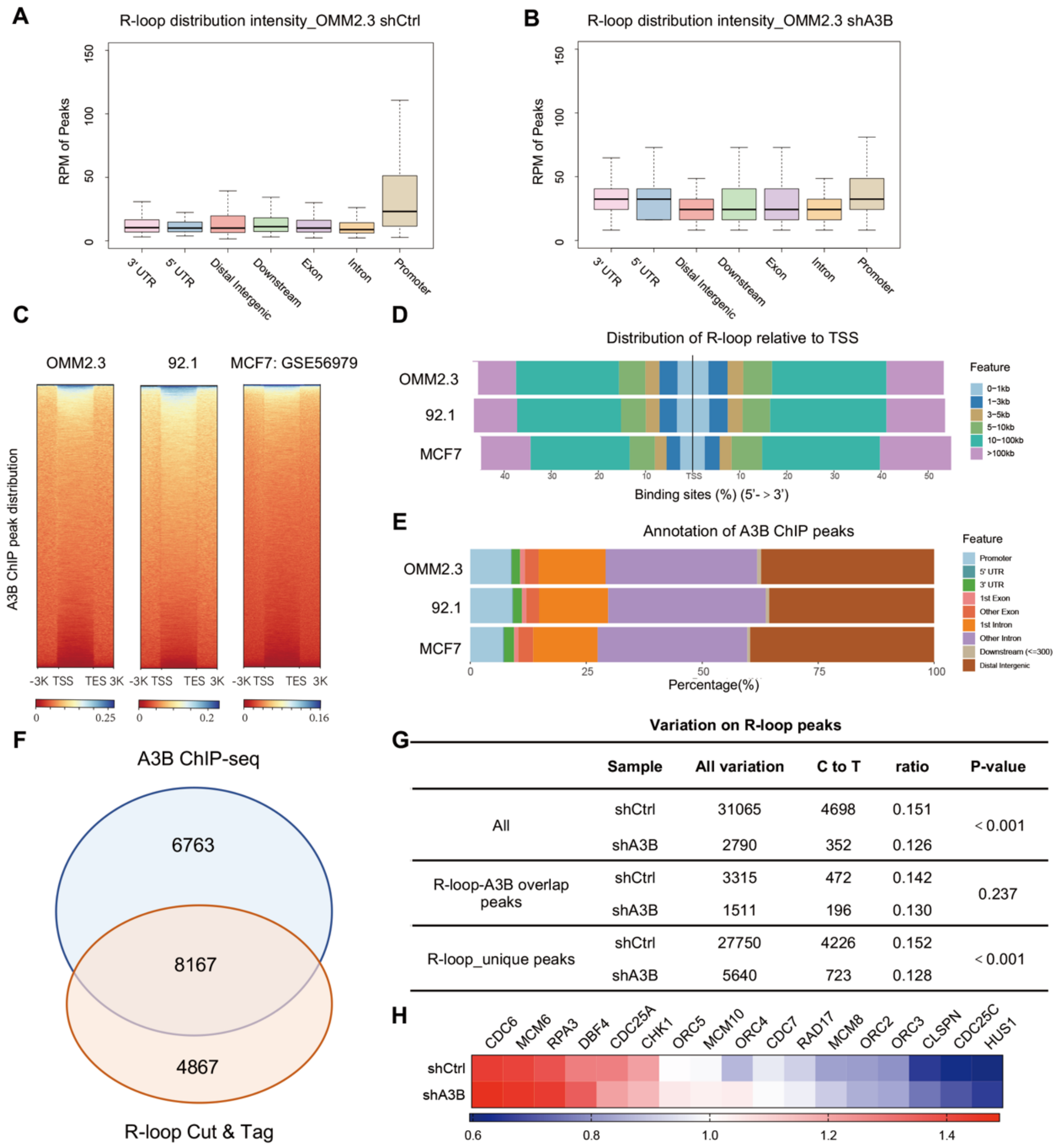

	Sample	All variation	C to T	ratio	P-value
All	shCtrl	31065	4698	0.151	< 0.001
	shA3B	2790	352	0.126	
R-loop-A3B overlap peaks	shCtrl	3315	472	0.142	0.237
	shA3B	1511	196	0.130	
R-loop_unique peaks	shCtrl	27750	4226	0.152	< 0.001
	shA3B	5640	723	0.128	

Fig. S5 Analysis of sequencing data.

A, B Distribution intensity of R-loop peaks captured by CUT&Tag assay in various regions of the genome (mean ± s.e.m). **C** The peak distribution and heatmap presentation of ChIP-seq signals for A3B in regions ±3 Kb from TSS region. **D** The peak distribution of ChIP-seq signals for A3B relative to TSS. **E** Annotation of ChIP-seq peaks. **F** Venn diagram showing genome-wide co-occurrence across A3B target genes (ChIP; blue) and R-loop-prone genes (CUT&Tag; orange) in OMM2.3. **G** Variations of different regions on R-loop peaks. P-values were calculated by Chi-square test. **H** Heatmap of gene expression of 92.1 RNA-seq.

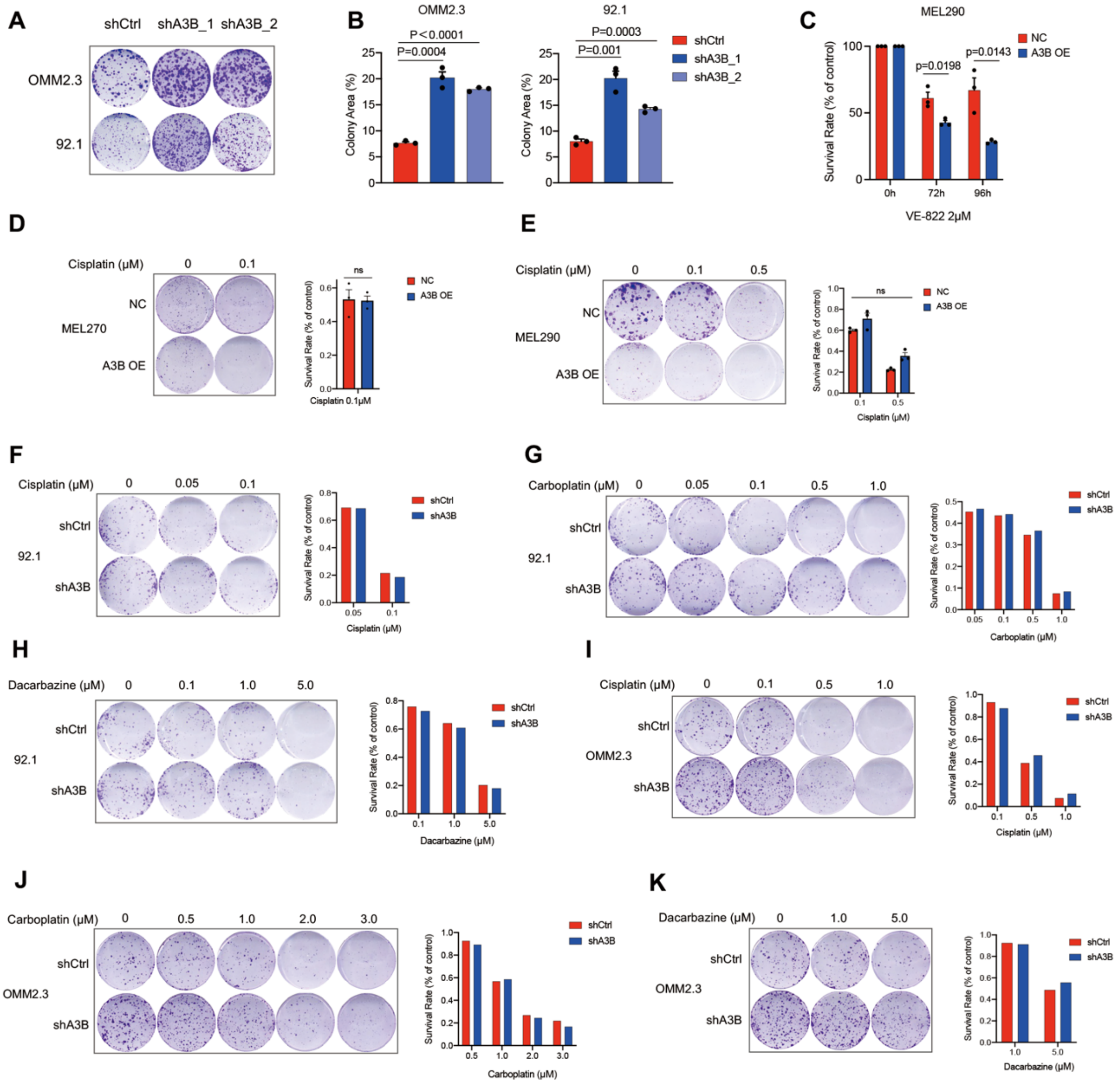

Fig. S6 Drug sensitivity assays.

A Images of representative colony formation assays of control (shCtrl) and A3B-depleted (shA3B) OMM2.3 and 92.1 cells. **B** Bar chart displayed the quantification of colony formation assay of (**A**) (n=3 biological replicates, mean + s.e.m). **C** Survival rate of MEL290 cell lines represented by cell viability treated with 2μM VE-822 for 0, 72, 96h (n=3 biological replicates, mean + s.e.m). **D-K** Colony formation assays of UVM cell lines treated with different concentrations of cisplatin, carboplatin and dacarbazine.

Table S1 OMM2.3 IP-MS proteins

Protein Group	Protein ID	Accession	-10lgP	Area shCtrl-IgG	Area shA3B-IgG	Area shCtrl-IP	Area shA3B-IP	#Peptides	#Unique	#Spec shCtrl-IgG	#Spec shA3B-IgG	#Spec shCtrl-IP	#Spec shA3B-IP	PTM
36	12	sp|Q15029|U5S1_HUMAN	558.54	5.21E+08	8.16E+08	8.65E+08	6.83E+08	73	72	67	77	67	56	Carbamid
3	31	sp|Q13813|SPTN1_HUMAN	554.23	7.34E+06	1.63E+07	3.69E+07	1.44E+07	209	3	210	176	166	142	Carbamid
4	37	tr|A0A0D9SF54|A0A0D9SF54_HUMAN	553.53	1.10E+07	6.67E+06	1.33E+07	5.59E+06	207	1	209	174	164	142	Carbamid
2	311	sp|Q09666|AHNK_HUMAN	552.17	2.10E+09	1.50E+09	1.28E+09	8.80E+08	272	272	280	242	210	177	Carbamid
10	68	sp|Q01082|SPTB2_HUMAN	529.01	8.19E+08	5.73E+08	8.40E+08	3.91E+08	156	145	140	118	122	103	Carbamid
1	104	sp|P35579|MYH9_HUMAN	528.97	3.33E+08	2.65E+09	1.78E+09	4.14E+09	190	138	111	224	169	240	Carbamid
11	63	sp|P46940|IQGA1_HUMAN	520.88	1.31E+09	1.55E+09	2.13E+09	1.48E+09	128	115	134	116	120	110	Carbamid
7	175	sp|Q6P2Q9|PRP8_HUMAN	494.88	9.39E+08	1.60E+09	1.53E+09	1.32E+09	157	157	152	190	151	144	Carbamid
5	284	sp|P78527|PRKDC_HUMAN	494.56	3.29E+08	3.74E+08	5.12E+08	4.95E+08	193	191	142	150	159	148	Carbamid
147	196	sp|Q86XP3|DDX42_HUMAN	490.54	9.63E+07	2.72E+08	8.49E+07	1.95E+08	44	44	25	43	20	30	Carbamid
6	123	sp|P35580|MYH10_HUMAN	488.06	5.05E+07	8.12E+08	3.96E+08	1.17E+09	172	122	66	175	112	176	Carbamid
9	3	sp|P63261|ACTG_HUMAN	472.32	3.73E+07	1.56E+08	1.38E+08	2.09E+08	96	3	75	152	98	150	Carbamid
8	1	sp|P60709|ACTB_HUMAN	472.05	4.10E+07	1.26E+08	1.39E+08	2.54E+08	95	2	75	150	98	152	Carbamid
16	174	sp|O75643|U520_HUMAN	470.53	2.67E+08	5.69E+08	5.38E+08	4.17E+08	119	119	94	114	91	89	Carbamid
13	85	sp|Q15393|SF3B3_HUMAN	469.25	1.23E+09	2.11E+09	1.44E+09	1.46E+09	105	105	112	130	79	102	Carbamid
23	92	sp|O75533|SF3B1_HUMAN	463.39	6.08E+08	1.42E+09	8.33E+08	1.01E+09	96	96	80	101	69	79	Carbamid
93	274	sp|Q08211|DHX9_HUMAN	462.02	3.67E+07	1.02E+08	5.10E+08	4.43E+08	54	54	21	34	52	47	Carbamid
74	17	sp|P07437|TBB5_HUMAN	448.11	8.03E+07	6.62E+07	1.25E+08	7.30E+07	42	4	51	48	49	31	Carbamid
25	75	sp|Q86UE4|LYRIC_HUMAN	446.22	9.81E+07	1.10E+08	1.86E+08	1.67E+08	68	3	92	83	72	73	Oxidation
31	343	sp|Q15149|PLEC_HUMAN	445.04	3.76E+07	9.32E+07	1.30E+07	3.84E+07	166	162	28	69	26	48	Carbamid
86	18	sp|P68371|TBB4B_HUMAN	444.6	1.92E+06	0		4.01E+06	42	1	49	43	44	30	Carbamid
17	60	sp|Q7KZF4|SND1_HUMAN	444.38	1.38E+09	1.62E+09	2.03E+09	1.54E+09	73	72	93	111	85	84	Carbamid
37	32	sp|Q14554|PDIA5_HUMAN	441.76	1.23E+09	1.65E+09	1.43E+09	1.24E+09	67	67	71	86	62	58	Carbamid
35	304	sp|Q9Y2W2|WBP11_HUMAN	441.05	1.02E+09	2.23E+09	9.21E+08	1.70E+09	58	58	70	96	52	74	Oxidation
32	96	sp|P40939|ECHA_HUMAN	432.18	5.36E+08	7.17E+08	9.88E+08	7.43E+08	61	61	63	70	65	59	Carbamid
12	7	sp|P08670|VIME_HUMAN	430.08	1.94E+09	5.69E+09	2.87E+09	5.87E+09	87	77	87	127	90	99	Carbamid
103	82	sp|O60488|ACSL4_HUMAN	427.96	1.53E+08	1.84E+08	2.00E+08	5.95E+08	48	44	39	38	31	36	Carbamid
30	124	sp|P55884|EIF3B_HUMAN	427.58	8.07E+08	5.78E+08	9.36E+08	5.08E+08	73	73	68	52	58	50	Carbamid
62	69	sp|P25205|MCM3_HUMAN	426.38	3.23E+08	3.29E+08	4.83E+08	3.58E+08	56	54	50	45	48	46	Carbamid
39	416	sp|P68104|EF1A1_HUMAN	425.31	3.58E+08	1.48E+08	2.61E+08	1.16E+08	52	6	111	55	37	32	Carbamid
22	122	sp|P09874|PARP1_HUMAN	424.15	6.33E+08	1.16E+09	7.84E+08	9.47E+08	85	84	71	87	62	70	Carbamid
27	253	sp|Q7Z406|MYH14_HUMAN	422.97	5.26E+05	2.58E+07	9.98E+06	4.39E+07	103	41	15	69	41	82	Carbamid
88	45	sp|Q99459|CDC5L_HUMAN	422.22	3.27E+08	3.36E+08	6.02E+08	3.65E+08	51	51	46	43	46	45	Carbamid
28	26	sp|O95573|ACSL3_HUMAN	420.93	7.56E+08	6.87E+08	8.10E+08	6.72E+08	68	64	63	69	54	54	Carbamid
81	87	sp|Q8NE71|ABCF1_HUMAN	420.43	2.61E+08	4.92E+08	6.76E+08	5.52E+08	50	49	48	52	47	47	Carbamid
129	251	sp|P36776|LONM_HUMAN	419.22					52	1	41	30	30	22	Carbamid
127	172	tr|K7EJE8|K7EJE8_HUMAN	418.9				4.22E+05	52	1	41	30	30	23	Carbamid
127	185	tr|K7EKE6|K7EKE6_HUMAN	418.9				4.22E+05	52	1	41	30	30	23	Carbamid
61	362	sp|P19338|NUCL_HUMAN	416.02	1.02E+08	1.47E+08	1.31E+09	1.16E+09	47	23	35	36	62	61	Oxidation
152	211	sp|P04264|K2C1_HUMAN	414.87	1.85E+08	1.86E+08	1.01E+08	4.05E+07	46	25	39	40	28	20	Carbamid
50	131	tr|C9JZR2|C9JZR2_HUMAN	413.97	3.98E+08	3.40E+08	4.38E+08	2.85E+08	60	60	64	53	49	44	Carbamid
50	158	sp|O60716|CTND1_HUMAN	413.97	3.98E+08	3.40E+08	4.38E+08	2.85E+08	60	60	64	53	49	44	Carbamid
119	498	sp|O15372|EIF3H_HUMAN	412.06	4.13E+08	2.85E+08	4.11E+08	2.05E+08	37	37	44	29	34	23	Carbamid
119	499	tr|B3KS98|B3KS98_HUMAN	412.06	4.13E+08	2.85E+08	4.11E+08	2.05E+08	37	37	44	29	34	23	Carbamid
87	97	sp|Q86UP2|KTN1_HUMAN	410.72	6.83E+07	7.48E+07	5.38E+07	6.68E+07	77	77	28	40	19	31	Carbamid
140	149	tr|A0A0U1RRM4|A0A0U1RRM4_HUMAN	409.77	8.50E+07	1.45E+08	4.61E+08	3.61E+08	34	34	26	28	37	39	Carbamid
107	1687	sp|P06748|NPM_HUMAN	407.73	2.02E+08	5.39E+08	1.83E+09	1.80E+09	40	39	22	27	41	46	Carbamid
18	28	sp|Q16181|SEPT7_HUMAN	406.35	1.43E+09	1.41E+09	1.47E+09	8.61E+08	68	62	102	96	81	59	Carbamid
51	13	sp|Q08J23|NSUN2_HUMAN	405.85	2.15E+08	3.50E+08	3.68E+08	3.51E+08	63	63	55	58	50	53	Carbamid
57	86	sp|Q12929|EPS8_HUMAN	405.54	3.58E+08	3.49E+08	4.44E+08	3.14E+08	53	53	58	51	47	42	Carbamid
57	90	tr|A0A2R8YE63|A0A2R8YE63_HUMAN	405.54	3.58E+08	3.49E+08	4.44E+08	3.14E+08	53	53	58	51	47	42	Carbamid
112	79	sp|O94826|TOM70_HUMAN	403.13	3.95E+08	2.33E+08	2.71E+08	2.09E+08	40	40	43	38	33	31	Carbamid
15	43	sp|P11021|BIP_HUMAN	403.06	2.87E+09	2.65E+09	2.88E+09	2.44E+09	79	73	117	93	79	70	Carbamid
33	103	tr|E5RJU9|E5RJU9_HUMAN	401.1	0				61	1	84	73	67	61	Deamidat
52	67	sp|Q15459|SF3A1_HUMAN	400.96	6.80E+08	1.20E+09	7.99E+08	9.31E+08	60	60	53	62	51	50	Carbamid
280	155	sp|Q13263|TIF1B_HUMAN	398.94	1.16E+08	9.20E+07	1.05E+08	9.72E+07	32	32	26	20	21	21	Carbamid
141	233	sp|P07814|SYEP_HUMAN	398.24	2.40E+07	2.32E+07	4.41E+07	5.10E+07	73	72	23	21	28	26	Carbamid
67	286	sp|Q9P2E9|RRBP1_HUMAN	398.18	2.25E+08	2.58E+08	4.28E+08	5.31E+08	61	58	29	35	49	57	Carbamid
20	53	sp|Q9UHD8|SEPT9_HUMAN	397.74	2.09E+09	1.79E+09	1.86E+09	1.32E+09	61	56	94	78	62	67	Carbamid
29	72	sp|P11142|HSP7C_HUMAN	396.26	2.80E+08	3.95E+08	2.77E+08	2.98E+08	55	23	73	78	52	57	Carbamid
144	16	sp|Q9UMS4|PRP19_HUMAN	396.12	2.16E+08	2.83E+08	6.25E+08	4.23E+08	38	38	24	35	37	34	Carbamid
45	11	sp|Q15019|SEPT2_HUMAN	392.39	1.10E+09	9.73E+08	1.24E+09	6.49E+08	60	54	61	55	42	42	Carbamid
65	133	sp|Q9Y4W6|AFG32_HUMAN	392.37	2.26E+08	2.23E+08	2.72E+08	2.23E+08	54	53	51	43	46	40	Carbamid
202	30	sp|Q8WXF1|PSPC1_HUMAN	392.35	1.45E+08	3.04E+08	1.74E+08	2.49E+08	36	35	19	28	20	24	Carbamid
77	93	sp|Q9Y262|EIF3L_HUMAN	392.08	7.01E+08	4.71E+08	5.67E+08	3.44E+08	49	49	52	39	33	35	Carbamid
89	278	tr|F8WE88|F8WE88_HUMAN	391.81		1.35E+06	6.45E+05	7.10E+06	86	2	2	41	18	44	Carbamid
49	200	tr|F5H6E2|F5H6E2_HUMAN	391.19	5.66E+07	4.01E+08	2.44E+08	5.75E+08	63	59	35	59	43	50	Carbamid
70	412	tr|E7EW69|E7EW69_HUMAN	390.41	3.87E+08	4.19E+08	4.20E+08	2.27E+08	45	38	54	51	41	33	Carbamid
70	414	sp|Q9P0V9|SEP10_HUMAN	390.41	3.87E+08	4.19E+08	4.20E+08	2.27E+08	45	38	54	51	41	33	Carbamid
70	423	tr|B5ME97|B5ME97_HUMAN	390.41	3.87E+08	4.19E+08	4.20E+08	2.27E+08	45	38	54	51	41	33	Carbamid
91	227	sp|Q13724|MOGS_HUMAN	390.38	1.30E+08	1.44E+08	3.57E+08	3.25E+08	45	45	39	35	39	43	Carbamid
102	264	tr|G3V394|G3V394_HUMAN	390.31					85	1	2	40	17	42	Carbamid
138	440	sp|P35269|T2FA_HUMAN	390.23	4.41E+08	7.46E+08	3.70E+08	5.74E+08	42	41	41	48	20	31	Acetylatio
79	218	sp|Q9P2J5|SYLC_HUMAN	390.15	1.29E+08	1.27E+08	1.02E+08	9.48E+07	61	61	47	42	32	38	Carbamid
80	624	tr|A0A087WV01|A0A087WV01_HUMAN	388.82					45	1	85	44	27	21	Carbamid
63	128	tr|J3KTA4|J3KTA4_HUMAN	388.75	3.34E+08	4.32E+08	6.13E+08	6.17E+08	56	42	51	56	45	46	Carbamid
63	129	sp|P17844|DDX5_HUMAN	388.75	3.34E+08	4.32E+08	6.13E+08	6.17E+08	56	42	51	56	45	46	Carbamid
250	298	sp|Q9UH99|SUN2_HUMAN	388.65	8.03E+07	1.13E+08	7.87E+07	8.51E+07	26	26	26	27	22	21	Carbamid
64	355	sp|O75165|DJC13_HUMAN	388.28	9.12E+06	3.09E+07	1.36E+08	8.50E+07	80	79	20	37	66	50	Carbamid
40	313	sp|P05023|AT1A1_HUMAN	388.2	5.31E+08	4.75E+08	3.98E+08	3.87E+08	62	62	77	54	51	44	Carbamid
163	4	sp|P68363|TBA1B_HUMAN	388.18	2.17E+07	1.85E+07	2.87E+07	1.96E+07	39	1	37	32	24	25	Carbamid
46	121	sp|O94906|PRP6_HUMAN	388.04	2.80E+08	3.94E+08	4.55E+08	4.17E+08	62	62	66	59	57	50	Carbamid
71	408	sp|P20042|IF2B_HUMAN	387.31	6.29E+08	6.81E+08	9.74E+08	7.16E+08	50	50	48	46	37	38	Carbamid
41	259	tr|E9PDF6|E9PDF6_HUMAN	387.09	1.75E+07	2.61E+08	1.29E+08	3.55E+08	71	64	22	65	45	57	Carbamid
41	279	sp|O43795|MYO1B_HUMAN	387.09	1.75E+07	2.61E+08	1.29E+08	3.55E+08	71	64	22	65	45	57	Carbamid
117	201	sp|P63010|AP2B1_HUMAN	386.73	2.37E+07	3.46E+07	1.14E+08	7.92E+07	50	30	27	31	42	41	Carbamid
48	138	sp|P38646|GRP75_HUMAN	386.47	6.67E+08	7.34E+08	5.21E+08	5.93E+08	49	47	63	63	40	47	Carbamid
109	232	tr|A0A024R4E5|A0A024R4E5_HUMAN	386.3	1.10E+08	3.73E+07	1.87E+08	1.06E+08	59	59	37	21	48	29	Carbamid
53	2160	sp|P32969|RL9_HUMAN	385.67	1.69E+09	1.84E+09	2.28E+09	1.75E+09	44	44	60	71	49	34	Carbamid
205	168	tr|A0A2Q2TH77|A0A2Q2TH77_HUMAN	384.55	4.55E+07	7.17E+07	7.95E+07	3.40E+07	44	28	28	36	28	18	Carbamid
205	177	sp|Q08379|GOGA2_HUMAN	384.55	4.55E+07	7.17E+07	7.95E+07	3.40E+07	44	28	28	36	28	18	Carbamid
177	354	tr|A0A1W2PPS1|A0A1W2PPS1_HUMAN	383.68	1.11E+08	1.54E+08	2.05E+08	1.65E+08	41	41	28	29	26	24	Carbamid
177	360	sp|Q00839|HNRPU_HUMAN	383.68	1.11E+08	1.54E+08	2.05E+08	1.65E+08	41	41	28	29	26	24	Carbamid
99	36	sp|Q9UHB6|LIMA1_HUMAN	382.88	1.34E+07	2.31E+07	8.74E+06	3.86E+07	59	16	30	36	22	41	Carbamid
188	215	sp|Q8NBJ5|GT251_HUMAN	382.37	6.70E+07	1.86E+08	3.12E+08	3.47E+08	36	34	22	28	30	32	Carbamid
154	335	sp|Q12906|ILF3_HUMAN	380.56	4.51E+07	8.67E+07	7.41E+08	4.94E+08	38	38	22	24	38	32	Carbamid
95	56	sp|P49411|EFTU_HUMAN	380	6.57E+08	7.10E+08	5.79E+08	5.84E+08	42	42	48	47	34	32	Carbamid
164	221	sp|O43290|SNUT1_HUMAN	379.89	2.63E+08	3.13E+08	2.27E+08	2.82E+08	49	48	37	48	35	38	Carbamid
155	292	tr|A0A0A0MS51|A0A0A0MS51_HUMAN	379.85	3.90E+07	2.17E+08	1.01E+08	2.35E+08	41	41	22	35	23	32	Carbamid
73	202	tr|M0QY43|M0QY43_HUMAN	379.36				3.65E+06	60	2	11	48	28	51	Carbamid
149	386	sp|O00339|MATN2_HUMAN	378.16	4.52E+07	4.01E+07	6.00E+07	2.53E+07	38	8	34	32	31	27	Carbamid
331	530	sp|O43719|HTSF1_HUMAN	377.47	2.38E+08	3.49E+08	3.88E+08	3.43E+08	28	28	28	34	30	23	Carbamid
124	39	sp|P13010|XRCC5_HUMAN	377.27	5.84E+07	8.85E+07	5.32E+08	3.88E+08	46	46	23	26	47	47	Carbamid
157	293	sp|Q92620|PRP16_HUMAN	376.82	1.65E+08	3.46E+08	8.55E+06	2.46E+07	55	53	46	65	10	16	Carbamid
175	321	sp|Q9NYU2|UGGG1_HUMAN	375.56	4.25E+07	4.86E+07	3.91E+07	3.79E+07	51	50	38	42	25	26	Carbamid
319	4847	tr|A0A2R8Y811|A0A2R8Y811_HUMAN	375.31	1.90E+08	2.65E+08	5.93E+08	6.47E+08	30	30	14	17	27	23	Carbamid
319	4848	sp|P62263|RS14_HUMAN	375.31	1.90E+08	2.65E+08	5.93E+08	6.47E+08	30	30	14	17	27	23	Carbamid
105	345	sp|P55196|AFAD_HUMAN	374.62	2.98E+06	3.95E+06		7.39E+05	64	1	41	59	34	39	Carbamid
19	1639	sp|Q96AG4|LRC59_HUMAN	374.52	5.39E+09	7.13E+09	7.37E+09	6.26E+09	62	62	84	103	65	76	Carbamid
180	25	tr|F5H5D3|F5H5D3_HUMAN	372.98	3.51E+06	2.81E+06	3.97E+06	2.70E+06	37	3	34	29	25	22	Carbamid
131	497	sp|Q13347|EIF3I_HUMAN	372.19	5.97E+08	4.41E+08	5.47E+08	2.92E+08	35	35	39	27	29	18	Carbamid
56	102	sp|Q92499|DDX1_HUMAN	371.98	1.06E+08	2.11E+08	3.19E+08	2.49E+08	49	49	45	41	56	48	Carbamid
59	249	sp|P23246|SFPQ_HUMAN	371.89	4.12E+08	1.29E+09	1.02E+09	1.56E+09	47	45	34	55	37	48	Carbamid
82	206	sp|O94874|UFL1_HUMAN	371.83	3.04E+08	3.45E+08	3.44E+08	2.27E+08	49	49	45	47	44	36	Carbamid

75	81	sp|P55084|ECHB_HUMAN	371.55	4.72E+08	6.69E+08	7.16E+08	7.58E+08	46	45	42	52	46	40	Carbamid
92	198	sp|P42704|LPPRC_HUMAN	371.33	2.46E+07	3.16E+07	1.61E+08	1.37E+08	67	65	27	28	50	47	Carbamid
47	57	sp|Q15233|NONO_HUMAN	370.1	5.30E+08	1.08E+09	9.02E+08	1.17E+09	44	41	45	52	48	52	Carbamid
128	15	sp|P28331|NDUS1_HUMAN	369.01	1.58E+08	1.30E+08	1.68E+08	1.25E+08	44	44	37	26	34	27	Carbamid
90	296	sp|P46199|IF2M_HUMAN	368.72	2.92E+08	3.67E+08	3.12E+08	2.71E+08	43	43	43	52	34	34	Carbamid
69	50	sp|P33992|MCM5_HUMAN	368.66	3.65E+08	4.84E+08	5.88E+08	5.80E+08	53	52	42	53	42	48	Carbamid
257	99	sp|O76094|SRP72_HUMAN	368.65	5.96E+07	7.21E+07	7.20E+07	4.80E+07	37	37	30	30	22	15	Carbamid
376	358	sp|P26358|DNMT1_HUMAN	368.57	6.62E+06	2.98E+06	9.49E+06	8.28E+06	55	55	7	5	8	9	Carbamid
132	95	sp|P33176|KINH_HUMAN	368.49	8.10E+07	5.68E+07	9.35E+07	3.38E+07	52	41	43	36	34	23	Carbamid
72	245	tr|E5KLJ9|E5KLJ9_HUMAN	368.39			8.27E+05		58	5	48	47	43	41	Carbamid
55	171	sp|P52272|HNRPM_HUMAN	368.15	5.86E+07	1.34E+08	2.84E+08	4.82E+08	47	20	31	47	50	56	Carbamid
198	240	sp|P13645|K1C10_HUMAN	368.1	1.80E+08	1.81E+08	3.57E+07	3.75E+07	42	33	46	35	17	13	Carbamid
593	560	sp|Q6WKZ4|RFIP1_HUMAN	367.83	1.15E+08	1.94E+08	8.17E+07	1.08E+08	27	25	14	14	12	10	Carbamid
83	125	sp|P11940|PABP1_HUMAN	366.58	1.91E+07	7.64E+07	1.11E+08	1.34E+08	53	15	27	37	46	47	Carbamid
143	236	tr|H7BXY3|H7BXY3_HUMAN	366.1	3.46E+06	1.73E+07	1.65E+08	1.39E+08	60	59	6	16	50	41	Carbamid
143	247	sp|Q7L2E3|DHX30_HUMAN	366.1	3.46E+06	1.73E+07	1.65E+08	1.39E+08	60	59	6	16	50	41	Carbamid
104	388	sp|O14776|TCRG1_HUMAN	365.58	8.59E+08	7.80E+08	5.83E+08	6.43E+08	49	49	41	40	35	30	Carbamid
43	61	sp|P41091|IF2G_HUMAN	365.37	1.23E+09	1.63E+09	1.95E+09	1.88E+09	59	59	53	57	55	49	Carbamid
137	1139	sp|P13984|T2FB_HUMAN	364.53	7.03E+08	1.16E+09	7.53E+08	9.11E+08	35	35	48	36	27	36	Carbamid
78	203	sp|O60313|OPA1_HUMAN	363.7	4.51E+05	3.70E+05	3.01E+05		54	1	49	48	41	41	Carbamid
181	996	sp|Q7L2H7|EIF3M_HUMAN	362.52	3.38E+08	2.09E+08	3.10E+08	1.70E+08	32	25	35	24	21	18	Carbamid
216	801	sp|P05388|RLA0_HUMAN	362.13	9.75E+07	2.24E+08	1.92E+09	1.49E+09	36	36	15	18	38	27	Carbamid
113	120	sp|Q8N1G4|LRC47_HUMAN	361.88	2.38E+08	1.88E+08	2.79E+08	1.44E+08	35	35	40	39	39	23	Carbamid
139	64	sp|P14618|KPYM_HUMAN	361.69	2.30E+08	1.24E+08	1.95E+08	1.22E+08	41	39	44	32	31	30	Carbamid
169	147	sp|Q07065|CKAP4_HUMAN	361.43	4.72E+07	7.59E+07	4.82E+08	4.73E+08	33	32	24	27	32	32	Carbamid
221	146	sp|P61978|HNRPK_HUMAN	361	1.52E+08	2.08E+08	3.41E+08	2.80E+08	29	29	23	28	22	25	Carbamid
133	192	sp|Q9H0A0|NAT10_HUMAN	360.92	4.22E+07	9.24E+07	1.08E+08	1.49E+08	52	51	29	37	37	40	Carbamid
100	287	sp|O43143|DHX15_HUMAN	360.05	1.76E+08	2.32E+08	3.17E+08	2.51E+08	44	43	38	46	48	36	Carbamid
269	742	sp|Q96DI7|SNR40_HUMAN	359.87	1.02E+08	1.78E+08	2.32E+08	2.00E+08	22	22	21	26	23	25	Carbamid
178	40	tr|F8VQE1|F8VQE1_HUMAN	359.68					44	1	22	27	16	29	Carbamid
232	161	sp|O15371|EIF3D_HUMAN	359.5	1.14E+08	1.02E+08	8.32E+07	5.01E+07	34	34	25	22	24	14	Carbamid
338	394	tr|Q60FE5|Q60FE5_HUMAN	359.11	1.07E+07	1.45E+07	1.30E+07	1.52E+07	55	49	18	21	14	21	Carbamid
338	395	sp|P21333|FLNA_HUMAN	359.11	1.07E+07	1.45E+07	1.30E+07	1.52E+07	55	49	18	21	14	21	Carbamid
173	314	sp|Q9NSE4|SYIM_HUMAN	358.95	9.36E+07	5.92E+07	8.10E+07	5.92E+07	40	40	33	24	26	21	Carbamid
183	109	sp|Q1ED39|KNOP1_HUMAN	358.76	1.42E+08	1.35E+08	1.29E+08	1.34E+08	40	40	48	39	33	38	Carbamid
168	135	sp|P12268|IMDH2_HUMAN	358.51	2.13E+08	1.09E+08	2.08E+08	9.72E+07	36	33	35	30	29	26	Carbamid
199	347	sp|P41252|SYIC_HUMAN	357.96	1.72E+07	1.35E+07	3.47E+07	3.66E+07	54	53	14	16	26	28	Carbamid
161	380	tr|H0YBJ4|H0YBJ4_HUMAN	357.76	5.55E+06	9.47E+05	5.71E+06	5.15E+06	31	1	30	28	28	28	Carbamid
120	207	sp|O60568|PLOD3_HUMAN	357.69	7.88E+07	2.27E+08	3.76E+08	3.94E+08	38	37	29	37	41	40	Carbamid
122	58	tr|A0A0D9SFB3|A0A0D9SFB3_HUMAN	357.19	1.98E+07	3.31E+07	2.52E+07	2.18E+07	42	9	40	42	36	34	Carbamid
122	59	tr|A0A2R8YF78|A0A2R8YF78_HUMAN	357.19	1.98E+07	3.31E+07	2.52E+07	2.18E+07	42	9	40	42	36	34	Carbamid
122	62	tr|A0A0D9SG12|A0A0D9SG12_HUMAN	357.19	1.98E+07	3.31E+07	2.52E+07	2.18E+07	42	9	40	42	36	34	Carbamid
122	80	tr|A0A2R8YFS5|A0A2R8YFS5_HUMAN	357.19	1.98E+07	3.31E+07	2.52E+07	2.18E+07	42	9	40	42	36	34	Carbamid
122	83	tr|A0A2R8Y4A4|A0A2R8Y4A4_HUMAN	357.19	1.98E+07	3.31E+07	2.52E+07	2.18E+07	42	9	40	42	36	34	Carbamid
122	84	sp|O00571|DDX3X_HUMAN	357.19	1.98E+07	3.31E+07	2.52E+07	2.18E+07	42	9	40	42	36	34	Carbamid
122	140	tr|A0A0D9SF53|A0A0D9SF53_HUMAN	357.19	1.98E+07	3.31E+07	2.52E+07	2.18E+07	42	9	40	42	36	34	Carbamid
146	340	sp|Q16531|DDB1_HUMAN	356.89	3.89E+07	6.28E+07	6.28E+07	8.34E+07	51	51	28	36	33	30	Carbamid
212	338	sp|O60841|IF2P_HUMAN	356.13	6.03E+07	1.41E+08	1.20E+08	1.62E+08	48	48	32	40	31	40	Carbamid
212	339	tr|A0A087WUT6|A0A087WUT6_HUMAN	356.13	6.03E+07	1.41E+08	1.20E+08	1.62E+08	48	48	32	40	31	40	Carbamid
248	404	sp|P12107|COBA1_HUMAN	356.1	1.32E+07	5.33E+07	1.31E+08	1.53E+08	34	34	9	20	22	27	Carbamid
121	152	sp|P25705|ATPA_HUMAN	355.89	3.72E+08	4.09E+08	4.61E+08	4.53E+08	39	39	36	34	36	32	Carbamid
66	308	sp|Q9BXP5|SRRT_HUMAN	355.76	6.42E+08	1.06E+09	4.21E+07	1.09E+08	60	60	75	72	16	35	Carbamid
366	290	sp|P35527|K1C9_HUMAN	355.73	1.84E+08	1.50E+08	1.02E+08	4.52E+07	31	30	35	28	21	10	Carbamid
190	1076	sp|Q9BPW8|NIPS1_HUMAN	354.21	3.40E+08	7.33E+08	4.64E+08	4.87E+08	35	34	27	35	26	22	Carbamid
108	182	tr|A0A0A0MRA8|A0A0A0MRA8_HUMAN	353.82	1.31E+07	1.09E+07	3.48E+07	8.75E+06	51	6	41	36	38	32	Carbamid
182	622	sp|Q12905|ILF2_HUMAN	353.17	6.65E+07	1.22E+08	7.64E+08	6.00E+08	30	30	13	23	39	39	Carbamid
234	376	sp|Q00610|CLH1_HUMAN	352.78	1.16E+07	2.37E+07	2.01E+07	2.79E+07	54	54	19	22	18	25	Carbamid
234	377	tr|A0A087WVQ6|A0A087WVQ6_HUMAN	352.78	1.16E+07	2.37E+07	2.01E+07	2.79E+07	54	54	19	22	18	25	Carbamid
126	157	sp|Q9NVI7|ATD3A_HUMAN	351.73	6.66E+06	9.46E+06	1.39E+07	1.79E+07	37	2	29	38	38	32	Acetylatic
135	212	sp|P0DMV8|HS71A_HUMAN	351.46	4.51E+07	5.18E+07	3.41E+07	4.21E+07	38	13	39	43	27	30	Carbamid
135	213	sp|P0DMV9|HS71B_HUMAN	351.46	4.51E+07	5.18E+07	3.41E+07	4.21E+07	38	13	39	43	27	30	Carbamid
135	214	tr|A0A0G2JIW1|A0A0G2JIW1_HUMAN	351.46	4.51E+07	5.18E+07	3.41E+07	4.21E+07	38	13	39	43	27	30	Carbamid
243	396	sp|Q9BQG0|MBB1A_HUMAN	351.15	8.14E+05	7.20E+06	1.38E+08	1.42E+08	41	41	2	10	39	43	Carbamid
210	254	sp|Q12874|SF3A3_HUMAN	350.99	1.26E+08	2.98E+08	2.88E+08	2.75E+08	33	33	22	32	26	29	Carbamid
111	114	sp|P60228|EIF3E_HUMAN	350.07	6.17E+08	4.52E+08	5.14E+08	3.71E+08	40	40	42	38	32	25	Carbamid
24	407	sp|P05141|ADT2_HUMAN	349.79	6.71E+08	8.36E+08	8.06E+08	9.11E+08	50	14	77	82	73	59	Carbamid
68	42	tr|D6RGI3|D6RGI3_HUMAN	348.81	4.96E+08	3.93E+08	4.70E+08	2.19E+08	46	25	48	49	45	34	Carbamid
68	46	sp|Q9NVA2|SEP11_HUMAN	348.81	4.96E+08	3.93E+08	4.70E+08	2.19E+08	46	25	48	49	45	34	Carbamid
68	55	tr|D6RER5|D6RER5_HUMAN	348.81	4.96E+08	3.93E+08	4.70E+08	2.19E+08	46	25	48	49	45	34	Carbamid
116	88	tr|A8K968|A8K968_HUMAN	348.15	1.41E+06	1.40E+06	2.70E+06		46	1	40	37	35	30	Carbamid
247	283	sp|Q8N3F8|MILK1_HUMAN	347.62	9.09E+07	5.51E+07	4.68E+07	3.37E+07	37	37	31	24	17	16	Carbamid
298	326	sp|Q13045|FLII_HUMAN	347.53	5.50E+06	2.70E+07	8.76E+06	3.28E+07	40	40	13	26	10	20	Carbamid
296	156	sp|P61011|SRP54_HUMAN	347.52	9.18E+07	4.23E+07	7.27E+07	5.07E+07	27	27	26	19	22	17	Carbamid
101	136	sp|P12956|XRCC6_HUMAN	347.29	1.55E+08	2.30E+08	8.40E+08	7.68E+08	47	47	31	37	46	45	Carbamid
125	267	sp|O95782|AP2A1_HUMAN	347.12	1.27E+07	2.60E+07	1.08E+08	9.24E+07	46	31	25	31	37	35	Carbamid
284	148	sp|O43660|PLRG1_HUMAN	346.85	7.52E+07	7.83E+07	1.42E+08	1.19E+08	28	28	25	26	24	26	Carbamid
106	409	sp|P05198|IF2A_HUMAN	346.42	5.84E+08	7.68E+08	1.10E+09	8.58E+08	38	38	37	33	37	36	Carbamid
114	302	sp|Q14152|EIF3A_HUMAN	346.29	8.71E+07	1.08E+08	9.89E+06	3.01E+06	73	73	47	57	7	7	Carbamid
283	300	sp|Q9NVP1|DDX18_HUMAN	345.44	8.03E+07	1.15E+08	1.85E+08	1.43E+08	28	27	30	29	26	24	Carbamid
162	320	sp|Q9NR30|DDX21_HUMAN	345.42	1.44E+07	2.40E+07	2.39E+08	1.95E+08	39	35	15	21	44	38	Carbamid
145	154	sp|P34897|GLYM_HUMAN	344.71	2.14E+08	2.42E+08	2.24E+08	2.29E+08	34	34	37	33	30	25	Carbamid
153	204	sp|O15523|DDX3Y_HUMAN	344.48	1.02E+07	1.82E+07	1.64E+07	1.12E+07	40	8	37	38	33	32	Carbamid
123	255	sp|Q14247|SRC8_HUMAN	343.97	1.09E+09	1.17E+09	9.25E+08	9.07E+08	29	29	37	31	33	47	Carbamid
215	151	sp|P30419|NMT1_HUMAN	343.19	7.60E+07	1.34E+08	9.04E+07	1.13E+08	31	31	23	24	21	21	Carbamid
136	806	sp|P39023|RL3_HUMAN	342.3	3.52E+07	1.10E+08	9.21E+08	8.21E+08	36	36	12	22	52	47	Carbamid
213	1205	sp|P39748|FEN1_HUMAN	342.08	1.02E+08	2.58E+08	9.82E+07	2.53E+08	29	27	18	32	13	26	Carbamid
225	242	sp|Q8N442|GUF1_HUMAN	342.07	7.37E+07	1.43E+08	1.24E+08	1.16E+08	30	30	24	30	27	21	Carbamid
314	139	sp|P06576|ATPB_HUMAN	341.52	8.76E+07	1.16E+08	1.45E+08	1.13E+08	27	27	21	19	23	17	Deamidat
34	623	sp|P12236|ADT3_HUMAN	341.21	1.03E+08	1.27E+08	1.42E+08	1.60E+08	47	4	65	67	61	47	Carbamid
274	110	sp|Q9Y450|HBS1L_HUMAN	340.61	1.09E+08	1.10E+08	1.37E+08	6.64E+07	36	36	32	29	23	18	Carbamid
352	384	sp|Q9NTI5|PDS5B_HUMAN	340.5	8.92E+06	1.11E+07	3.09E+07	2.25E+07	45	28	15	19	5	7	Carbamid
159	316	sp|Q9UJS0|CMC2_HUMAN	339.76	7.15E+07	7.22E+07	9.17E+07	9.21E+07	35	26	34	33	26	29	Carbamid
151	328	sp|O60231|DHX16_HUMAN	339.6	1.54E+08	2.83E+08	2.40E+08	2.56E+08	49	48	34	41	35	32	Carbamid
97	186	tr|A0A1X7SBZ2|A0A1X7SBZ2_HUMAN	339.34	1.69E+08	2.07E+08	2.09E+08	2.22E+08	42	29	39	45	33	38	Carbamid
97	187	sp|Q92841|DDX17_HUMAN	339.34	1.69E+08	2.07E+08	2.09E+08	2.22E+08	42	29	39	45	33	38	Carbamid
97	189	tr|A0A1W2PQ51|A0A1W2PQ51_HUMAN	339.34	1.69E+08	2.07E+08	2.09E+08	2.22E+08	42	29	39	45	33	38	Carbamid
312	333	sp|Q8IYB8|SUV3_HUMAN	339.29	3.32E+07	3.52E+07	9.36E+06	2.24E+07	40	40	6	19	3	12	Carbamid
345	378	sp|P16615|AT2A2_HUMAN	339.01	3.29E+07	3.56E+07	2.72E+07	2.90E+07	35	35	21	19	13	21	Carbamid
324	73	sp|Q9BY44|EIF2A_HUMAN	338.8	2.59E+07	8.66E+06	8.41E+07	4.64E+07	30	30	18	8	26	19	Carbamid
297	398	tr|E7EVA0|E7EVA0_HUMAN	338.43	6.71E+07	8.89E+07	6.80E+07	7.01E+07	46	46	18	25	13	17	Carbamid
276	23	sp|Q92917|GPKOW_HUMAN	338.29	9.06E+07	1.55E+08	9.17E+07	1.27E+08	30	30	28	31	22	25	Carbamid
217	349	sp|P35251|RFC1_HUMAN	338.27	2.35E+08	1.42E+08	1.96E+08	1.20E+08	46	46	40	33	36	29	Carbamid
172	808	sp|Q14141|SEPT6_HUMAN	338.21	6.91E+07	4.50E+07	6.25E+07	2.66E+07	32	14	32	34	27	22	Carbamid
258	397	tr|E7EX73|E7EX73_HUMAN	338.17		1.96E+05			42	1	32	23	16	13	Carbamid
258	399	tr|E9PGM1|E9PGM1_HUMAN	338.17		1.96E+05			42	1	32	23	16	13	Carbamid
258	400	tr|E7EUU4|E7EUU4_HUMAN	338.17		1.96E+05			42	1	32	23	16	13	Carbamid
197	243	sp|O76031|CLPX_HUMAN	337.91	1.07E+08	1.02E+08	7.70E+07	7.79E+07	37	35	27	33	19	27	Carbamid
228	273	sp|P08237|PFKAM_HUMAN	337.64	8.37E+07	5.04E+07	8.56E+07	3.86E+07	35	31	30	23	30	19	Carbamid
228	325	tr|A0A2R8Y891|A0A2R8Y891_HUMAN	337.64	8.37E+07	5.04E+07	8.56E+07	3.86E+07	35	31	30	23	30	19	Carbamid
38	504	sp|P12235|ADT1_HUMAN	337.53	3.93E+07	4.82E+07	4.73E+07	5.54E+07	46	5	65	66	60	46	Carbamid
246	401	sp|Q04637|IF4G1_HUMAN	337.38		4.46E+05	6.81E+05	4.45E+05	42	1	32	23	17	14	Carbamid
446	265	sp|Q9C040|TRIM2_HUMAN	337.12	2.55E+07	2.36E+07	3.35E+07	1.23E+07	27	26	15	15	11	9	Carbamid
84	271	sp|P11387|TOP1_HUMAN	337.11	5.24E+08	1.25E+09	4.74E+08	1.10E+09	46	46	40	49	36	43	Carbamid
167	14	sp|P07355|ANXA2_HUMAN	336.85	8.71E+08	7.60E+08	6.58E+08	6.56E+08	30	30	34	32	29	29	Carbamid
170	382	sp|P26640|SYVC_HUMAN	336.73	5.18E+07	5.60E+07	3.56E+07	3.60E+07	45	44	31	30	17	19	Carbamid
438	348	sp|P56192|SYMC_HUMAN	336.48	7.75E+06	1.25E+07	1.92E+07	2.63E+07	34	34	11	14	11	15	Carbamid

737	664	sp|Q13283|G3BP1_HUMAN	334.8	1.35E+06	1.40E+06	2.00E+06	1.74E+07	22	18	4	5	3	10	Carbamid
309	239	sp|O75448|MED24_HUMAN	334.79	3.48E+07	6.58E+07	5.66E+07	3.91E+07	41	41	25	33	23	17	Carbamid
309	257	tr|A0A0B4J1W0|A0A0B4J1W0_HUMAN	334.79	3.48E+07	6.58E+07	5.66E+07	3.91E+07	41	41	25	33	23	17	Carbamid
240	330	tr|A0A0C4DGA6|A0A0C4DGA6_HUMAN	334.71	4.46E+07	8.42E+07	7.79E+07	6.54E+07	36	36	23	39	33	29	Carbamid
240	331	sp|Q14527|HLTF_HUMAN	334.71	4.46E+07	8.42E+07	7.79E+07	6.54E+07	36	36	23	39	33	29	Carbamid
480	318	sp|Q9Y2L1|RRP44_HUMAN	334.62	2.40E+07	8.57E+06	1.96E+07	5.49E+06	34	34	24	8	14	6	Carbamid
156	71	sp|Q7L0Y3|TM10C_HUMAN	334.6	2.07E+08	2.70E+08	2.46E+08	2.27E+08	32	32	30	35	28	28	Carbamid
268	299	sp|Q16513|PKN2_HUMAN	332.65	5.36E+07	3.30E+07	5.89E+07	2.05E+07	36	36	28	22	22	18	Carbamid
251	78	sp|O43172|PRP4_HUMAN	332.1	1.38E+08	9.07E+07	2.26E+08	8.77E+07	31	31	30	27	33	23	Carbamid
560	435	sp|Q13573|SNW1_HUMAN	331.38	6.34E+07	6.63E+07	4.00E+07	5.87E+07	26	26	19	20	11	17	Carbamid
259	277	sp|P54136|SYRC_HUMAN	331.28	1.62E+07	2.22E+07	3.11E+07	5.06E+07	35	35	17	16	18	22	Carbamid
346	1212	sp|O75821|EIF3G_HUMAN	330.65	2.12E+08	1.29E+08	1.95E+08	8.11E+07	26	26	24	14	19	15	Carbamid
166	107	sp|P10809|CH60_HUMAN	330.15	1.35E+08	9.19E+07	1.14E+08	7.97E+07	32	32	40	29	26	21	Carbamid
214	905	sp|Q99988|GDF15_HUMAN	330.05	6.80E+07	2.32E+08	5.01E+08	8.83E+08	30	30	14	23	30	32	Carbamid
203	216	sp|P08240|SRPRA_HUMAN	329.55	6.79E+07	8.58E+07	7.80E+07	8.60E+07	35	35	27	24	22	25	Carbamid
343	418	sp|P42167|LAP2B_HUMAN	328.99	4.52E+07	6.45E+07	6.33E+07	9.98E+07	24	15	17	17	16	23	Carbamid
60	1634	sp|P48047|ATPO_HUMAN	328.85	9.78E+08	1.24E+09	1.15E+09	1.21E+09	41	14	53	65	45	46	Carbamid
207	234	sp|P61221|ABCE1_HUMAN	328.74	1.32E+08	8.72E+07	1.47E+08	9.34E+07	36	36	29	24	28	25	Carbamid
290	145	sp|Q9Y697|NFS1_HUMAN	328	6.07E+07	8.06E+07	8.97E+07	6.97E+07	24	24	21	20	24	20	Carbamid
255	413	sp|Q15120|PDK3_HUMAN	327.87	1.57E+08	1.79E+08	1.76E+08	1.62E+08	30	27	26	30	22	19	Carbamid
209	507	tr|A6NMH6|A6NMH6_HUMAN	327.53	6.91E+07	6.57E+07	7.32E+07	3.07E+07	31	22	26	33	25	19	Carbamid
209	509	tr|A6NFQ9|A6NFQ9_HUMAN	327.53	6.91E+07	6.57E+07	7.32E+07	3.07E+07	31	22	26	33	25	19	Carbamid
209	511	sp|Q92599|SEPT8_HUMAN	327.53	6.91E+07	6.57E+07	7.32E+07	3.07E+07	31	22	26	33	25	19	Carbamid
209	512	tr|F8W8I8|F8W8I8_HUMAN	327.53	6.91E+07	6.57E+07	7.32E+07	3.07E+07	31	22	26	33	25	19	Carbamid
187	191	sp|P04843|RPN1_HUMAN	327.48	1.20E+08	1.19E+08	1.77E+08	1.39E+08	36	36	26	27	26	25	Carbamid
388	2909	sp|P20290|BTF3_HUMAN	327.47	1.95E+08	2.45E+08	1.47E+08	1.24E+08	21	21	20	23	12	14	Acetylatic
349	434	sp|O75306|NDUS2_HUMAN	327.45	7.99E+07	7.70E+07	7.47E+07	6.20E+07	24	24	23	20	13	13	Carbamid
342	89	sp|P41214|EIF2D_HUMAN	327.29	5.27E+07	5.16E+07	6.47E+07	4.08E+07	28	28	22	26	20	15	Carbamid
372	301	sp|Q99575|POP1_HUMAN	326.95	1.52E+08	1.11E+08	1.73E+08	1.54E+08	36	36	11	17	23	31	Carbamid
261	831	sp|P68400|CSK21_HUMAN	326.95	1.10E+08	1.07E+08	1.20E+08	8.03E+07	24	22	25	26	22	23	Carbamid
456	425	sp|P40222|TXLNA_HUMAN	326.24	5.50E+07	3.74E+07	3.08E+07	2.09E+07	27	25	24	18	14	11	Carbamid
578	383	sp|Q92896|GSLG1_HUMAN	326.08	1.23E+07	1.39E+07	1.14E+07	1.09E+07	35	34	14	14	11	11	Carbamid
286	41	sp|Q9UHR4|BI2L1_HUMAN	326.05	1.10E+08	1.61E+08	8.00E+07	1.15E+08	31	31	28	33	19	24	Carbamid
517	217	tr|H3BQQ2|H3BQQ2_HUMAN	325.85	2.00E+06	1.85E+06	1.68E+06		32	1	22	18	14	15	Carbamid
306	51	sp|Q9H223|EHD4_HUMAN	325.55	5.12E+07	3.55E+07	3.26E+07	2.37E+07	30	23	26	21	17	16	Carbamid
281	359	sp|Q96TA2|YMEL1_HUMAN	324.91	7.96E+07	1.00E+08	1.35E+08	1.19E+08	30	30	22	25	19	20	Carbamid
192	2046	sp|O14950|ML12B_HUMAN	324.74		6.20E+05		8.97E+05	23	2	17	33	17	29	Carbamid
218	94	sp|Q9UQB8|BAIP2_HUMAN	324.74	7.89E+07	1.25E+08	8.66E+07	1.04E+08	35	35	28	36	22	33	Carbamid
54	1682	tr|M0R0F0|M0R0F0_HUMAN	324.74	1.39E+09	2.02E+09	2.01E+09	1.70E+09	40	40	57	61	37	51	Carbamid
54	1683	sp|P46782|RS5_HUMAN	324.74	1.39E+09	2.02E+09	2.01E+09	1.70E+09	40	40	57	61	37	51	Carbamid
184	130	sp|Q9H089|LSG1_HUMAN	324.69	7.77E+07	1.47E+08	9.48E+07	1.34E+08	35	35	33	36	29	29	Carbamid
542	281	sp|Q86UK7|ZN598_HUMAN	324.21	1.03E+06				32	1	22	17	13	15	Carbamid
256	568	sp|Q9Y2T2|AP3M1_HUMAN	323.88	8.38E+07	9.14E+07	1.56E+08	9.84E+07	21	17	24	29	20	25	Carbamid
244	2737	tr|H0YBJ8|H0YBJ8_HUMAN	323.6	2.33E+06	2.41E+06	1.42E+06		19	1	29	34	24	28	Deamidat
572	4183	sp|P05386|RLA1_HUMAN	323.56	2.56E+07	4.90E+07	5.43E+08	2.97E+08	18	16	5	6	20	21	Carbamid
193	1427	tr|H7BY16|H7BY16_HUMAN	323.07			2.81E+05	3.47E+05	26	2	22	21	41	32	Oxidation
201	2053	sp|P19105|ML12A_HUMAN	321.85				1.81E+05	22	1	17	31	17	28	Carbamid
201	2054	tr|J3QRS3|J3QRS3_HUMAN	321.85				1.81E+05	22	1	17	31	17	28	Carbamid
364	297	sp|O95202|LETM1_HUMAN	321.7	5.02E+07	4.19E+07	3.09E+07	4.11E+07	34	33	23	19	11	14	Carbamid
465	1219	sp|O43852|CALU_HUMAN	321.28	6.76E+07	6.69E+07	6.36E+07	7.31E+07	22	22	14	16	12	16	Deamidat
396	208	sp|P42166|LAP2A_HUMAN	321.22	1.09E+07	1.93E+07	7.50E+06	1.60E+07	28	19	17	17	10	16	Carbamid
597	1197	sp|O75822|EIF3J_HUMAN	320.91	2.76E+07	1.82E+07	2.99E+07	1.22E+07	21	21	9	8	10	6	Carbamid
291	387	sp|Q9HAU0|PKHA5_HUMAN	320.77	4.17E+07	8.01E+07	2.61E+07	4.40E+07	33	33	32	43	11	19	Carbamid
393	118	sp|P35908|K22E_HUMAN	320.64	4.94E+07	6.07E+07	1.72E+07	6.82E+06	34	22	26	29	15	8	Carbamid
450	953	tr|H0Y8R1|H0Y8R1_HUMAN	320.26	1.99E+06	9.00E+06	1.64E+08	1.13E+08	23	23	5	6	26	24	Carbamid
450	954	tr|F5H5I6|F5H5I6_HUMAN	320.26	1.99E+06	9.00E+06	1.64E+08	1.13E+08	23	23	5	6	26	24	Carbamid
450	956	tr|H0YAK1|H0YAK1_HUMAN	320.26	1.99E+06	9.00E+06	1.64E+08	1.13E+08	23	23	5	6	26	24	Carbamid
450	957	sp|Q12849|GRSF1_HUMAN	320.26	1.99E+06	9.00E+06	1.64E+08	1.13E+08	23	23	5	6	26	24	Carbamid
263	230	sp|Q9H0D6|XRN2_HUMAN	320.09	4.17E+07	4.33E+07	7.14E+07	4.58E+07	43	43	25	23	27	14	Carbamid
293	422	tr|G3V0I5|G3V0I5_HUMAN	319.41	8.17E+07	7.98E+07	8.62E+07	5.83E+07	27	27	22	25	20	17	Carbamid
293	426	sp|P49821|NDUV1_HUMAN	319.41	8.17E+07	7.98E+07	8.62E+07	5.83E+07	27	27	22	25	20	17	Carbamid
482	967	sp|P26368|U2AF2_HUMAN	319.27	7.35E+06	1.74E+07	8.78E+06	1.60E+07	22	6	12	22	22	20	Carbamid
418	994	sp|P62136|PP1A_HUMAN	319.13	6.99E+06	1.93E+07	1.82E+07	1.53E+07	23	6	15	16	17	17	Carbamid
333	777	sp|Q9BUF5|TBB6_HUMAN	318.66		6.33E+04			27	1	25	20	16	14	Carbamid
307	229	sp|O43776|SYNC_HUMAN	318.33	1.30E+08	7.32E+07	1.40E+08	1.09E+08	27	27	20	20	20	18	Carbamid
320	44	sp|P06733|ENOA_HUMAN	318.21	2.14E+08	7.76E+07	8.19E+07	4.85E+07	24	24	33	21	19	17	Carbamid
219	402	sp|O94875|SRBS2_HUMAN	318.17	2.71E+06	6.28E+06	4.42E+05	2.24E+06	36	3	29	34	24	25	Carbamid
287	366	sp|O94973|AP2A2_HUMAN	317.9	2.99E+06	9.47E+06	3.01E+07	2.86E+07	38	23	12	21	27	26	Carbamid
239	190	sp|O43395|PRPF3_HUMAN	317.48	1.78E+08	1.09E+08	1.13E+08	5.17E+07	36	36	38	28	25	16	Carbamid
361	1631	sp|O75323|NIPS2_HUMAN	317.47	1.91E+08	3.35E+08	2.44E+08	2.31E+08	27	18	20	28	18	14	Carbamid
238	276	tr|B9A018|B9A018_HUMAN	317.11	1.26E+08	1.19E+08	2.19E+08	1.68E+08	29	28	29	26	26	22	Carbamid
238	312	sp|Q53GS9|SNUT2_HUMAN	317.11	1.26E+08	1.19E+08	2.19E+08	1.68E+08	29	28	29	26	26	22	Carbamid
505	964	sp|P62140|PP1B_HUMAN	317.11	2.24E+07	2.21E+07	1.74E+07	2.69E+07	22	7	17	14	12	15	Carbamid
430	1511	sp|Q9NY65|TBA8_HUMAN	315.97			3.79E+06	3.72E+06	22	1	21	17	15	15	Carbamid
430	1516	tr|C9J2C0|C9J2C0_HUMAN	315.97			3.79E+06	3.72E+06	22	1	21	17	15	15	Carbamid
300	666	sp|P53677|AP3M2_HUMAN	315.9	9.57E+07	1.24E+08	1.42E+08	9.38E+07	22	18	23	27	19	24	Carbamid
148	829	sp|O00303|EIF3F_HUMAN	315.64	5.78E+08	3.91E+08	5.08E+08	3.15E+08	27	27	38	26	28	20	Carbamid
397	194	sp|P26639|SYTC_HUMAN	315.1	8.98E+07	2.67E+07	8.95E+07	4.02E+07	32	32	31	17	27	19	Carbamid
539	2348	sp|P67809|YBOX1_HUMAN	314.85	2.76E+07	3.35E+07	8.61E+07	1.04E+08	13	9	11	13	12	19	Acetylatic
226	1691	sp|P62424|RL7A_HUMAN	314.37	1.55E+08	3.42E+08	2.42E+09	2.36E+09	23	23	15	18	32	38	Carbamid
631	163	tr|A0A087WZ13|A0A087WZ13_HUMAN	314.05	3.79E+07	2.89E+07	2.35E+07	1.62E+07	24	24	13	12	7	9	Carbamid
631	179	tr|E9PAU2|E9PAU2_HUMAN	314.05	3.79E+07	2.89E+07	2.35E+07	1.62E+07	24	24	13	12	7	9	Carbamid
403	228	sp|Q8WX92|NELFB_HUMAN	313.9	4.44E+07	6.66E+07	2.49E+07	3.09E+07	28	28	20	18	11	10	Carbamid
557	1486	tr|K7ENG2|K7ENG2_HUMAN	313.65	2.80E+06	6.08E+06	3.35E+06	5.53E+06	18	2	12	16	19	16	Carbamid
953	1868	sp|Q6UX04|CWC27_HUMAN	313.4	1.58E+07	2.66E+07	1.82E+07	2.82E+07	11	11	3	9	8	6	Carbamid
651	942	sp|P23588|IF4B_HUMAN	312.47	1.19E+08	1.57E+08	5.35E+06	6.25E+06	24	24	17	21	4	5	
651	943	tr|E7EX17|E7EX17_HUMAN	312.47	1.19E+08	1.57E+08	5.35E+06	6.25E+06	24	24	17	21	4	5	
165	417	tr|M0QZM1|M0QZM1_HUMAN	312.43			2.77E+06	4.49E+06	28	1	17	29	31	30	Oxidation
189	995	tr|F8VVM2|F8VVM2_HUMAN	312.05	3.20E+07	3.51E+07	3.02E+07	4.87E+07	30	10	32	30	20	23	Carbamid
327	241	sp|P00367|DHE3_HUMAN	312	5.63E+06	1.52E+07	2.21E+07	1.84E+07	30	9	11	19	16	21	Carbamid
253	70	sp|Q9Y3I0|RTCB_HUMAN	311.78	1.44E+08	9.16E+07	1.93E+08	1.90E+08	27	27	21	20	23	23	Carbamid
176	2298	tr|H3BNC9|H3BNC9_HUMAN	311.58	1.80E+08	2.87E+08	5.15E+08	5.03E+08	28	28	37	24	28	24	Carbamid
231	431	tr|B1ANR0|B1ANR0_HUMAN	311.55	1.34E+06	2.62E+06	6.87E+06	6.60E+06	33	1	15	21	28	27	Carbamid
231	437	sp|Q13310|PABP4_HUMAN	311.55	1.34E+06	2.62E+06	6.87E+06	6.60E+06	33	1	15	21	28	27	Carbamid
347	6086	sp|P35268|RL22_HUMAN	311.52	1.42E+09	1.25E+09	1.59E+09	9.31E+08	17	17	24	18	17	16	Carbamid
142	570	sp|Q8NC51|PAIRB_HUMAN	311.44	2.48E+08	2.50E+08	3.01E+08	4.71E+08	28	28	35	31	26	26	Carbamid
229	840	sp|P36578|RL4_HUMAN	311.14	4.59E+07	9.58E+07	5.93E+08	5.21E+08	24	24	15	22	33	25	Carbamid
273	178	sp|O15091|MRPP3_HUMAN	310.86	3.65E+07	3.80E+07	4.87E+07	2.30E+07	30	30	24	30	19	16	Carbamid
407	305	sp|P18074|ERCC2_HUMAN	310.75	2.73E+07	4.27E+07	2.99E+07	3.58E+07	30	30	19	23	15	17	Carbamid
449	427	sp|Q9ULV4|COR1C_HUMAN	310.14	9.80E+06	1.05E+08	2.99E+07	1.20E+08	32	31	7	24	8	17	Carbamid
303	272	sp|Q9UHB9|SRP68_HUMAN	310.11	3.55E+07	4.16E+07	5.36E+07	5.34E+07	32	32	18	21	20	22	Carbamid
391	389	sp|Q2NL82|TSR1_HUMAN	310.01	1.10E+07	2.44E+07	4.41E+07	5.80E+07	25	25	14	15	15	19	Carbamid
328	153	sp|O60832|DKC1_HUMAN	309.84	7.46E+07	1.37E+08	2.15E+08	1.70E+08	31	31	20	20	25	19	Carbamid
351	620	sp|P63244|RACK1_HUMAN	309.64	5.71E+07	7.21E+07	1.37E+08	1.18E+08	21	21	22	19	20	19	Carbamid
445	787	sp|P04844|RPN2_HUMAN	309.55	2.56E+07	1.97E+07	6.53E+07	3.37E+07	20	20	13	15	17	14	Deamidat
339	379	sp|A3KMH1|VWA8_HUMAN	309.5	1.66E+07	4.82E+07	2.19E+07	4.08E+07	59	58	20	20	20	13	Carbamid
534	462	sp|Q9Y2U8|MAN1_HUMAN	309.28	2.46E+07	2.88E+07	1.41E+07	1.39E+07	31	31	17	20	8	9	Carbamid
196	167	sp|P48735|IDHP_HUMAN	309.08	2.54E+08	2.06E+08	2.12E+08	1.75E+08	33	33	33	30	26	23	Carbamid
782	1215	sp|P29692|EF1D_HUMAN	309.06	3.61E+07	2.77E+07	4.23E+07	2.69E+07	16	16	10	8	7	6	Carbamid
384	979	sp|Q9UGM6|SYWM_HUMAN	309.03	1.06E+08	1.03E+08	1.14E+08	9.58E+07	22	22	20	16	17	12	Carbamid
98	496	sp|P35749|MYH11_HUMAN	308.52		3.14E+06			42	3	18	43	29	44	Carbamid
444	319	sp|Q9BXF6|RFIP5_HUMAN	308.22	4.96E+07	6.72E+07	4.36E+07	2.91E+07	25	25	22	23	16	16	Carbamid
444	405	tr|A0A1B0GTL5|A0A1B0GTL5_HUMAN	308.22	4.96E+07	6.72E+07	4.36E+07	2.91E+07	25	25	22	23	16	16	Carbamid
746	1123	sp|O15446|RPA34_HUMAN	308.16	3.38E+07	2.25E+07	6.42E+07	5.55E+07	16	16	12	7	15	13	Carbamid
58	578	sp|P30533|AMRP_HUMAN	308.09	4.36E+09	4.60E+09	2.50E+09	3.59E+09	39	39	55	58	43	48	Deamidat
363	704	sp|Q13217|DNJC3_HUMAN	307.87	1.07E+08	7.24E+07	9.72E+07	5.88E+07	26	26	24	19	18	14	Carbamid

429	655	sp|Q8IYB3|SRRM1_HUMAN	307.85	3.06E+08	2.21E+08	2.88E+08	3.02E+08	23	23	19	19	19	26	Carbamid
429	656	tr|A9Z1X7|A9Z1X7_HUMAN	307.85	3.06E+08	2.21E+08	2.88E+08	3.02E+08	23	23	19	19	19	26	Carbamid
371	518	sp|P60842|IF4A1_HUMAN	306.78	2.01E+07	1.91E+07	2.25E+07	9.90E+06	26	13	20	15	18	13	Carbamid
385	1050	sp|Q8IVM0|CCD50_HUMAN	306.36	1.72E+08	1.57E+08	1.59E+08	1.20E+08	22	21	19	19	18	11	Carbamid
292	361	tr|H7BXR3|H7BXR3_HUMAN	306.23		6.60E+06	2.77E+06	2.35E+06	31	3	25	29	22	22	Oxidation
194	692	sp|P23396|RS3_HUMAN	306.16	5.66E+08	6.94E+08	1.54E+09	1.29E+09	28	28	25	26	29	27	Carbamid
458	1439	sp|O14874|BCKD_HUMAN	306.04	7.75E+07	1.18E+08	1.20E+08	9.34E+07	22	22	15	20	16	13	Carbamid
390	1224	sp|Q9NX58|LYAR_HUMAN	305.99	1.21E+08	1.16E+08	2.42E+08	2.31E+08	22	22	18	12	25	17	Carbamid
596	712	sp|O60563|CCNT1_HUMAN	305.82	5.10E+07	8.70E+07	5.58E+07	6.66E+07	18	17	13	17	12	13	Carbamid
413	390	sp|Q14974|IMB1_HUMAN	305.62	2.46E+07	4.37E+07	2.62E+07	1.79E+07	27	27	16	21	10	11	Carbamid
354	1213	sp|Q9GZS3|WDR61_HUMAN	305.59	1.19E+08	1.44E+08	1.83E+08	1.33E+08	20	20	19	23	19	20	Carbamid
289	574	sp|P09543|CN37_HUMAN	305.54	9.98E+07	9.65E+07	1.17E+08	9.14E+07	28	28	24	22	20	20	Carbamid
469	1126	sp|Q99714|HCD2_HUMAN	305.38	4.50E+07	4.31E+07	5.83E+07	5.24E+07	18	18	18	12	14	17	Carbamid
468	776	sp|P18615|NELFE_HUMAN	304.97	7.55E+07	9.55E+07	2.76E+06	1.12E+07	25	25	21	24	2	11	Carbamid
322	1134	sp|Q16698|DECR_HUMAN	304.58	8.40E+06	6.14E+07	5.96E+08	6.73E+08	21	21	11	16	23	25	Carbamid
227	597	sp|Q96A33|CCD47_HUMAN	303.73	2.39E+08	2.45E+08	2.41E+08	2.49E+08	28	27	29	24	19	25	Carbamid
245	353	sp|Q6NUQ4|TM214_HUMAN	303.7	1.02E+08	8.34E+07	1.14E+08	7.12E+07	34	34	27	24	20	17	Carbamid
186	1713	sp|Q02878|RL6_HUMAN	303.25	8.74E+07	2.23E+08	1.76E+09	1.70E+09	27	26	19	20	33	39	Deamidat
21	1513	sp|P23284|PPIB_HUMAN	302.74	1.90E+10	1.67E+10	9.28E+09	1.29E+10	51	49	109	98	56	76	Carbamid
489	1992	sp|Q9BSH4|TACO1_HUMAN	302.62	1.65E+08	1.46E+08	1.41E+08	1.07E+08	22	22	21	17	11	9	Carbamid
380	628	sp|P35249|RFC4_HUMAN	302.61	6.05E+07	5.07E+07	1.44E+08	1.27E+08	20	19	22	17	20	17	Carbamid
414	193	sp|P28340|DPOD1_HUMAN	302.54	3.51E+06	1.60E+07	8.41E+06	1.63E+07	38	38	8	23	13	17	Carbamid
414	219	tr|M0R2B7|M0R2B7_HUMAN	302.54	3.51E+06	1.60E+07	8.41E+06	1.63E+07	38	38	8	23	13	17	Carbamid
325	697	sp|Q9NYL9|TMOD3_HUMAN	302.28	2.23E+07	1.48E+08	9.77E+07	2.47E+08	22	22	12	23	17	23	Carbamid
310	310	sp|Q02809|PLOD1_HUMAN	302.12	4.71E+07	8.42E+07	5.96E+07	4.42E+07	30	29	23	31	18	22	Carbamid
613	231	sp|Q01780|EXOSX_HUMAN	302.1	3.45E+06	4.59E+06	3.28E+07	3.94E+07	33	33	8	6	19	17	Carbamid
323	468	sp|O60506|HNRPQ_HUMAN	301.97	2.40E+06	3.75E+06	1.03E+07	3.13E+06	25	4	19	22	21	21	Carbamid
381	324	sp|P33993|MCM7_HUMAN	301.53	3.75E+07	2.84E+07	2.71E+07	2.25E+07	30	30	22	20	18	13	Carbamid
432	802	sp|O43684|BUB3_HUMAN	301.34	8.53E+07	1.25E+08	1.38E+08	1.39E+08	23	23	19	20	14	14	Carbamid
556	707	tr|A0A0J9YVP6|A0A0J9YVP6_HUMAN	301.3	1.07E+08	1.98E+08	8.96E+07	1.44E+08	22	22	21	27	16	17	Carbamid
556	710	tr|A0A0J9YXX5|A0A0J9YXX5_HUMAN	301.3	1.07E+08	1.98E+08	8.96E+07	1.44E+08	22	22	21	27	16	17	Carbamid
556	711	sp|Q9UHX1|PUF60_HUMAN	301.3	1.07E+08	1.98E+08	8.96E+07	1.44E+08	22	22	21	27	16	17	Carbamid
96	992	sp|P62701|RS4X_HUMAN	301.2	5.02E+08	8.57E+08	1.25E+09	1.14E+09	37	19	38	37	40	48	Carbamid
612	1435	tr|Q5JXI8|Q5JXI8_HUMAN	300.87	2.64E+08	1.35E+08	2.03E+08	1.40E+08	13	13	15	11	12	13	Carbamid
260	646	sp|O43148|MCES_HUMAN	300.73	1.61E+08	2.21E+08	1.99E+08	1.95E+08	26	26	25	28	25	21	Carbamid
408	280	sp|Q16891|MIC60_HUMAN	300.33	1.43E+07	2.40E+07	2.78E+07	2.77E+07	24	24	19	14	12	17	Carbamid
235	285	sp|P47897|SYQ_HUMAN	299.81	1.75E+07	2.48E+07	3.21E+07	3.83E+07	38	37	20	18	20	20	Carbamid
110	1933	sp|P09012|SNRPA_HUMAN	299.5	8.75E+08	8.01E+08	6.29E+08	6.61E+08	21	14	53	47	27	31	Acetylatic
551	403	sp|Q9BX66|SRBS1_HUMAN	299.44					26	3	14	14	3	8	Carbamid
387	464	tr|H0YBP1|H0YBP1_HUMAN	299.04	2.75E+07	1.95E+07	4.22E+07	1.71E+07	32	31	22	23	19	12	Carbamid
387	466	sp|Q05397|FAK1_HUMAN	299.04	2.75E+07	1.95E+07	4.22E+07	1.71E+07	32	31	22	23	19	12	Carbamid
412	694	sp|P40937|RFC5_HUMAN	298.78	3.90E+07	4.44E+07	1.23E+08	1.09E+08	24	22	15	14	22	17	Carbamid
402	969	sp|O76021|RL1D1_HUMAN	298.76	1.14E+08	9.33E+07	2.08E+08	2.33E+08	27	27	12	13	20	23	Carbamid
421	485	sp|Q13136|LIPA1_HUMAN	298.53	4.37E+07	4.01E+07	4.28E+07	1.96E+07	35	25	31	22	22	16	Carbamid
278	1010	tr|A0A0B4J269|A0A0B4J269_HUMAN	298.5	1.71E+05	1.04E+06			25	1	28	27	23	16	Carbamid
301	421	sp|Q9Y295|DRG1_HUMAN	297.72	6.36E+07	8.61E+07	1.30E+08	1.10E+08	23	23	21	20	21	19	Carbamid
600	1041	sp|Q15293|RCN1_HUMAN	297.55	4.49E+07	2.50E+07	3.23E+07	1.57E+07	17	17	14	14	12	7	Deamidat
222	1392	sp|P46777|RL5_HUMAN	297.54	1.01E+08	9.83E+07	4.92E+08	4.00E+08	22	22	25	17	43	20	Carbamid
222	1423	tr|A0A2R8Y6J3|A0A2R8Y6J3_HUMAN	297.54	1.01E+08	9.83E+07	4.92E+08	4.00E+08	22	22	25	17	43	20	Carbamid
262	633	sp|Q9BZF9|UACA_HUMAN	297.53	4.13E+07	1.01E+08	4.73E+07	6.77E+07	64	63	37	41	28	30	Carbamid
270	1188	tr|F8W6I7|F8W6I7_HUMAN	296.92	1.05E+08	1.62E+08	1.98E+08	2.47E+08	20	15	20	23	19	27	Carbamid
270	1189	sp|P09651|ROA1_HUMAN	296.92	1.05E+08	1.62E+08	1.98E+08	2.47E+08	20	15	20	23	19	27	Carbamid
1468	3702	sp|P28799|GRN_HUMAN	296.85	3.11E+07	1.71E+07	2.88E+07	1.22E+07	10	10	10	5	4	5	Carbamid
813	4022	sp|P35637|FUS_HUMAN	296.68	9.39E+07	1.01E+08	2.57E+07	5.54E+07	12	12	11	12	5	9	Carbamid
252	315	sp|P08238|HS90B_HUMAN	296.67	4.48E+07	3.22E+07	4.21E+07	2.37E+07	36	20	26	21	26	17	Carbamid
425	456	sp|P50570|DYN2_HUMAN	296.57	1.61E+07	7.61E+06	8.98E+06	4.12E+06	32	17	21	15	13	9	Carbamid
362	478	sp|O14617|AP3D1_HUMAN	296.38	1.85E+07	4.27E+07	3.93E+07	2.78E+07	38	38	21	29	14	18	Carbamid
1020	567	sp|P11182|ODB2_HUMAN	296.15	2.08E+04	4.86E+07		6.86E+07	23	23	1	21	0	16	Carbamid
514	855	sp|P31943|HNRH1_HUMAN	296.1	3.48E+07	2.59E+07	1.24E+07	1.26E+07	18	11	15	14	11	12	Carbamid
514	857	tr|G8JLB6|G8JLB6_HUMAN	296.1	3.48E+07	2.59E+07	1.24E+07	1.26E+07	18	11	15	14	11	12	Carbamid
42	811	sp|P68032|ACTC_HUMAN	296.05	1.34E+08	6.49E+08	4.02E+08	9.90E+08	44	2	28	69	34	65	Oxidation
191	1172	sp|O43809|CPSF5_HUMAN	295.85	9.32E+08	2.47E+09	1.35E+09	1.79E+09	33	33	35	46	29	32	Carbamid
492	374	sp|P46087|NOP2_HUMAN	295.7	1.84E+07	5.46E+07	1.02E+08	1.35E+08	26	26	8	15	18	24	Carbamid
358	500	sp|P40938|RFC3_HUMAN	295.31	6.14E+07	6.33E+07	1.60E+08	1.42E+08	24	24	17	18	25	27	Carbamid
636	786	sp|P27824|CALX_HUMAN	294.94	4.38E+07	3.56E+07	2.94E+07	2.26E+07	22	22	17	14	7	8	Carbamid
426	475	sp|Q96AQ6|PBIP1_HUMAN	294.84	6.64E+07	9.51E+07	8.51E+07	8.26E+07	25	25	20	21	16	19	Carbamid
383	725	sp|Q15118|PDK1_HUMAN	294.54	5.36E+07	4.72E+07	4.94E+07	2.97E+07	21	18	22	19	18	17	Carbamid
463	1311	sp|P36873|PP1G_HUMAN	294.52	7.34E+06	2.98E+06	1.33E+07	9.49E+06	20	4	16	15	13	15	Carbamid
524	1322	sp|P27695|APEX1_HUMAN	294.12	7.17E+07	3.71E+07	4.41E+07	2.32E+07	17	17	16	12	12	10	Carbamid
410	564	sp|Q53H12|AGK_HUMAN	294.1	7.01E+07	8.61E+07	1.17E+08	6.58E+07	24	24	21	19	16	13	Carbamid
304	830	sp|P50750|CDK9_HUMAN	294.08	1.09E+08	2.00E+08	1.28E+08	1.64E+08	26	23	30	27	15	21	Carbamid
435	470	sp|P42285|MTREX_HUMAN	294.01	1.93E+07	1.24E+07	4.80E+07	3.31E+07	32	32	23	14	22	13	Carbamid
398	137	sp|Q06210|GFPT1_HUMAN	292.97	8.08E+07	1.49E+07	4.57E+07	1.70E+07	33	33	36	18	16	10	Carbamid
692	826	sp|Q4KMP7|TB10B_HUMAN	292.66	1.68E+07	3.20E+07	2.04E+07	2.19E+07	19	17	11	20	10	16	Carbamid
369	1078	sp|P04406|G3P_HUMAN	292.61	2.84E+08	2.34E+08	2.10E+08	1.55E+08	21	21	28	28	20	15	Carbamid
275	294	sp|Q15046|SYK_HUMAN	292.44	6.55E+07	8.06E+07	6.86E+07	8.97E+07	29	29	25	25	17	18	Carbamid
44	848	sp|P61247|RS3A_HUMAN	292.35	1.84E+09	2.06E+09	2.91E+09	2.70E+09	36	36	56	60	68	54	Carbamid
379	317	sp|P12814|ACTN1_HUMAN	292.29	2.28E+06	2.47E+06	1.75E+07	3.04E+06	34	19	13	19	13	15	Carbamid
647	436	tr|K7EIG7|K7EIG7_HUMAN	292.28	1.30E+08	8.61E+07	1.00E+08	1.14E+08	39	37	3	13	3	16	Carbamid
647	451	sp|O94832|MYO1D_HUMAN	292.28	1.30E+08	8.61E+07	1.00E+08	1.14E+08	40	38	3	13	3	16	Carbamid
555	559	sp|P98175|RBM10_HUMAN	291.98	1.01E+08	1.26E+08	5.71E+07	6.89E+07	27	25	19	28	13	16	Carbamid
512	516	sp|Q9Y2X3|NOP58_HUMAN	291.93	1.12E+08	6.80E+07	1.19E+08	9.46E+07	23	23	18	13	22	18	Carbamid
295	2419	sp|P24844|MYL9_HUMAN	291.92	3.35E+06	1.61E+07	2.45E+07	2.39E+07	17	3	15	26	16	22	Carbamid
321	873	sp|P22695|QCR2_HUMAN	291.66	6.59E+07	6.39E+07	5.21E+07	7.23E+07	20	20	23	20	19	20	Carbamid
336	580	sp|Q9NWT1|PK1IP_HUMAN	291.54	4.21E+07	6.59E+07	1.28E+08	1.09E+08	21	21	20	23	23	28	Carbamid
335	336	sp|P46063|RECQ1_HUMAN	291.04	2.21E+07	3.82E+07	8.48E+07	7.73E+07	26	25	17	19	28	22	Carbamid
373	375	sp|O60244|MED14_HUMAN	291.03	2.53E+07	4.70E+07	3.63E+07	3.03E+07	37	37	23	29	19	21	Carbamid
204	1333	sp|P08574|CY1_HUMAN	290.74	2.64E+08	3.39E+08	3.69E+08	3.61E+08	21	21	25	29	23	21	Carbamid
664	1042	sp|Q09028|RBBP4_HUMAN	290.63	1.09E+07	4.30E+06	4.59E+07	1.78E+07	18	11	9	7	12	9	Carbamid
983	984	sp|O00622|CYR61_HUMAN	290.52	2.83E+07	3.44E+06	3.85E+07	7.82E+06	16	16	15	4	12	5	Carbamid
683	461	tr|J3KN66|J3KN66_HUMAN	290.46	1.62E+06	2.39E+06		1.32E+06	20	1	5	7	7	7	Carbamid
478	1148	sp|Q92665|RT31_HUMAN	290.3	8.05E+07	6.07E+07	8.84E+07	4.29E+07	19	18	16	14	19	14	Carbamid
637	2599	sp|P62917|RL8_HUMAN	290.3	1.43E+07	5.02E+07	3.97E+08	4.02E+08	19	19	10	7	16	14	Carbamid
679	810	sp|P61158|ARP3_HUMAN	290.23	2.88E+06	3.87E+07	3.52E+07	4.50E+07	17	17	5	14	13	11	Carbamid
224	2250	sp|P60660|MYL6_HUMAN	289.97		3.19E+06	2.59E+07		18	1	15	29	21	21	Carbamid
224	2251	tr|J3KND3|J3KND3_HUMAN	289.97		3.19E+06	2.59E+07		18	1	15	29	21	21	Carbamid
411	1745	sp|Q9UBQ5|EIF3K_HUMAN	289.94	2.61E+08	1.72E+08	2.33E+08	1.31E+08	22	22	18	17	15	10	Carbamid
577	1337	tr|K7EP90|K7EP90_HUMAN	289.94	5.21E+07	7.35E+07	6.61E+07	7.07E+07	16	15	13	14	12	12	Carbamid
577	1338	sp|Q9BTD8|RBM42_HUMAN	289.94	5.21E+07	7.35E+07	6.61E+07	7.07E+07	16	15	13	14	12	12	Carbamid
778	529	tr|A0A1B0GVQ5|A0A1B0GVQ5_HUMAN	289.83	1.25E+07	7.44E+06	8.73E+06	3.55E+06	22	19	12	10	7	6	Carbamid
778	536	tr|A0A1B0GWF2|A0A1B0GWF2_HUMAN	289.83	1.25E+07	7.44E+06	8.73E+06	3.55E+06	22	19	12	10	7	6	Carbamid
778	538	tr|A0A0D9SG72|A0A0D9SG72_HUMAN	289.83	1.25E+07	7.44E+06	8.73E+06	3.55E+06	22	19	12	10	7	6	Carbamid
778	539	tr|A0A2R8Y5D4|A0A2R8Y5D4_HUMAN	289.83	1.25E+07	7.44E+06	8.73E+06	3.55E+06	22	19	12	10	7	6	Carbamid
778	542	tr|A0A1B0GTP9|A0A1B0GTP9_HUMAN	289.83	1.25E+07	7.44E+06	8.73E+06	3.55E+06	22	19	12	10	7	6	Carbamid
778	543	sp|P61764|STXB1_HUMAN	289.83	1.25E+07	7.44E+06	8.73E+06	3.55E+06	22	19	12	10	7	6	Carbamid
470	332	sp|Q9UQE7|SMC3_HUMAN	289.77	2.71E+06	9.31E+06	9.28E+07	7.65E+07	42	42	6	12	19	15	Carbamid
803	372	sp|Q9BQ52|RNZ2_HUMAN	289.34	8.24E+06	6.40E+06	1.26E+07	3.73E+06	23	23	8	10	8	4	Carbamid
428	562	sp|P51398|RT29_HUMAN	289.28	4.76E+07	5.97E+07	9.46E+07	1.25E+08	21	21	12	14	18	19	Carbamid
940	1409	sp|Q9UQ35|SRRM2_HUMAN	289.28	8.84E+07	1.47E+08	1.22E+08	1.82E+08	23	23	17	18	10	12	Deamidat
285	2425	sp|P23528|COF1_HUMAN	289.25	5.78E+08	2.61E+08	3.14E+08	2.25E+08	21	15	30	24	20	17	Carbamid
712	479	sp|Q9BZH6|WDR11_HUMAN	289.15	1.22E+06	3.22E+06	1.14E+07	1.78E+07	33	33	1	6	10	15	Carbamid
595	576	sp|Q0VDF9|HSP7E_HUMAN	289.11	1.07E+07	1.10E+07	2.42E+07	1.55E+07	20	20	12	14	11	13	Carbamid
731	344	tr|E9PS17|E9PS17_HUMAN	289.05	2.53E+06	1.83E+06	8.12E+05	1.54E+06	29	29	6	4	1	3	Carbamid
731	357	sp|Q96KG9|SCYL1_HUMAN	289.05	2.53E+06	1.83E+06	8.12E+05	1.54E+06	29	29	6	4	1	3	Carbamid
422	476	sp|P78347|GTF2I_HUMAN	288.87	1.33E+07	2.69E+07	1.26E+07	2.49E+07	33	32	9	17	10	16	Carbamid
330	419	sp|P14868|SYDC_HUMAN	288.38	2.93E+07	2.30E+07	4.51E+07	5.52E+07	33	33	17	16	18	15	Carbamid

490	489	sp|Q92922|SMRC1_HUMAN	288.06	6.06E+06	4.80E+06	1.08E+07	1.89E+06	28	21	17	14	16	6	Carbamid
378	469	sp|Q12965|MYO1E_HUMAN	288.01	3.03E+06	1.71E+07	1.06E+07	2.72E+07	38	36	6	19	9	22	Carbamid
695	3051	sp|E9PAV3|NACAM_HUMAN	287.71	2.68E+08	2.60E+08	2.21E+08	2.66E+08	14	13	12	13	6	9	Oxidation
695	3410	tr|H0YHX9|H0YHX9_HUMAN	287.71	2.68E+08	2.60E+08	2.21E+08	2.66E+08	14	13	12	13	6	9	Oxidation
695	3411	sp|Q13765|NACA_HUMAN	287.71	2.68E+08	2.60E+08	2.21E+08	2.66E+08	14	13	12	13	6	9	Oxidation
481	527	sp|O00567|NOP56_HUMAN	287.37	1.47E+07	2.20E+07	4.81E+07	4.79E+07	25	25	11	15	17	18	Carbamid
315	443	sp|Q9H078|CLPB_HUMAN	287.33	1.48E+07	3.47E+07	2.59E+07	2.22E+07	28	10	21	22	21	16	Carbamid
706	460	sp|Q5JTV8|TOIP1_HUMAN	287.1		1.02E+06			20	1	4	7	7	6	Carbamid
466	1225	sp|P31689|DNJA1_HUMAN	286.95	4.47E+07	3.05E+07	5.07E+07	3.71E+07	16	16	30	14	12	10	Carbamid
374	2332	sp|O60828|PQBP1_HUMAN	286.6	7.71E+08	1.27E+09	8.46E+08	1.15E+09	18	18	20	24	16	21	Carbamid
877	764	sp|Q9HCM4|E41L5_HUMAN	286.3	2.75E+07	1.04E+07	4.63E+07	1.07E+07	18	18	14	9	14	8	Carbamid
313	661	sp|Q9UH03|SEPT3_HUMAN	286.22	5.43E+07	5.33E+07	3.06E+07	2.42E+07	25	19	32	25	17	10	Carbamid
313	687	tr|A0A2R8Y4H2|A0A2R8Y4H2_HUMAN	286.22	5.43E+07	5.33E+07	3.06E+07	2.42E+07	25	19	32	25	17	10	Carbamid
443	126	sp|Q9P258|RCC2_HUMAN	286.2	2.47E+07	3.60E+07	7.02E+07	4.05E+07	25	25	13	17	15	12	Carbamid
689	1059	sp|Q9UQ13|SHOC2_HUMAN	286.12	3.02E+07	2.76E+07	2.81E+07	1.28E+07	15	15	15	15	9	5	Carbamid
875	836	sp|Q9BZK7|TBL1R_HUMAN	286.06	5.14E+06	1.12E+07	9.56E+06	4.37E+06	17	17	8	13	9	4	Carbamid
344	438	sp|P02545|LMNA_HUMAN	285.78	1.87E+07	5.13E+07	8.16E+06	5.85E+07	35	33	18	23	6	20	Carbamid
174	2290	sp|P62081|RS7_HUMAN	285.73	1.21E+09	1.53E+09	2.32E+09	1.41E+09	22	22	25	27	29	22	Oxidation
676	483	sp|O00411|RPOM_HUMAN	285.71		1.45E+07	5.49E+07	4.76E+07	33	33	0	1	26	20	Carbamid
236	950	tr|H0YAR2|H0YAR2_HUMAN	285.71					28	1	18	21	27	26	Carbamid
459	522	sp|P49590|SYHM_HUMAN	285.71	1.10E+07	2.24E+07	4.14E+07	4.00E+07	23	22	11	16	16	21	Carbamid
503	632	sp|Q9GZL7|WDR12_HUMAN	285.42	5.60E+07	7.16E+07	8.25E+07	6.36E+07	19	19	13	16	15	12	Carbamid
431	569	sp|P07093|GDN_HUMAN	285.38	3.76E+07	3.60E+07	3.69E+07	2.68E+07	25	25	18	17	14	13	Carbamid
544	724	sp|Q9UN86|G3BP2_HUMAN	285.35	2.86E+06	3.46E+07	8.51E+07	9.19E+07	22	18	3	11	14	14	Carbamid
661	762	sp|Q12797|ASPH_HUMAN	285.02	1.95E+06	1.84E+06	4.86E+07	3.13E+07	24	23	7	7	23	15	Carbamid
401	346	sp|P19447|ERCC3_HUMAN	284.75	1.70E+07	4.13E+07	4.78E+07	3.71E+07	30	30	18	22	21	21	Carbamid
185	906	sp|P23193|TCEA1_HUMAN	284.6	4.70E+08	5.48E+08	2.81E+08	3.95E+08	24	24	32	35	24	31	Carbamid
185	907	tr|A0A1W2PPZ5|A0A1W2PPZ5_HUMAN	284.6	4.70E+08	5.48E+08	2.81E+08	3.95E+08	24	24	32	35	24	31	Carbamid
305	862	sp|Q9Y224|RTRAF_HUMAN	284.4	8.50E+07	1.07E+08	1.41E+08	1.62E+08	22	22	23	19	18	25	Carbamid
308	2606	tr|E9PI65|E9PI65_HUMAN	284.34					21	1	25	27	18	19	Carbamid
348	252	sp|Q6PI48|SYDM_HUMAN	284.11	3.37E+07	2.75E+07	3.01E+07	2.19E+07	28	28	21	17	18	16	Carbamid
781	3306	sp|Q9HCN8|SDF2L_HUMAN	283.52	8.04E+07	6.76E+07	9.36E+07	6.52E+07	13	13	10	10	10	10	Carbamid
691	238	tr|A0A0U1RQI5|A0A0U1RQI5_HUMAN	283.49					22	1	12	12	2	7	Carbamid
504	868	sp|P08559|ODPA_HUMAN	283.39	2.73E+07	2.49E+07	2.86E+07	1.75E+07	23	18	17	14	16	13	Carbamid
299	424	sp|Q6P1J9|CDC73_HUMAN	283.13	8.86E+07	7.64E+07	7.69E+07	6.39E+07	35	35	25	24	19	20	Carbamid
633	1081	tr|B3KTM8|B3KTM8_HUMAN	283.12	3.10E+07	4.90E+07	2.81E+07	3.74E+07	17	14	13	14	12	11	Carbamid
532	1463	sp|P52907|CAZA1_HUMAN	282.96	2.04E+07	6.88E+07	5.83E+07	9.56E+07	13	10	10	16	8	13	Carbamid
206	1140	tr|C9JEH7|C9JEH7_HUMAN	282.71	4.02E+06	8.61E+06	2.61E+07	2.14E+07	26	5	27	25	28	31	Carbamid
206	1142	sp|P22090|RS4Y1_HUMAN	282.71	4.02E+06	8.61E+06	2.61E+07	2.14E+07	26	5	27	25	28	31	Carbamid
657	1315	sp|Q15014|MO4L2_HUMAN	282.63	5.24E+07	5.40E+07	4.41E+07	3.00E+07	17	14	15	14	12	8	Carbamid
538	1095	sp|P52292|IMA1_HUMAN	282.58	3.68E+07	2.81E+07	6.08E+07	4.24E+07	17	17	16	13	14	12	Carbamid
495	2465	sp|Q15427|SF3B4_HUMAN	282.48	1.51E+08	2.09E+08	1.33E+08	1.43E+08	22	22	21	26	9	10	Carbamid
569	775	sp|P35250|RFC2_HUMAN	282.11	2.55E+07	2.24E+07	7.93E+07	7.37E+07	19	19	15	13	17	13	Carbamid
461	523	sp|Q9NVV4|PAPD1_HUMAN	282.09	1.59E+07	2.95E+07	5.82E+07	5.38E+07	23	23	14	20	15	16	Carbamid
200	968	sp|Q14320|FA50A_HUMAN	281.98	5.09E+08	3.62E+08	3.50E+08	2.21E+08	30	30	35	27	23	14	Oxidation
302	579	sp|P35240|MERL_HUMAN	281.8	7.06E+07	6.78E+07	8.66E+07	5.59E+07	36	33	31	23	23	17	Carbamid
635	789	sp|Q9NQW6|ANLN_HUMAN	281.78	2.73E+07	1.86E+07	1.99E+07	1.01E+07	33	33	21	15	15	9	Carbamid
350	2090	tr|J3QL56|J3QL56_HUMAN	281.65	1.19E+08	1.45E+08	1.71E+08	1.54E+08	27	27	22	20	16	19	
350	2091	sp|O75880|SCO1_HUMAN	281.65	1.19E+08	1.45E+08	1.71E+08	1.54E+08	27	27	22	20	16	19	
520	880	sp|O43837|IDH3B_HUMAN	281.27	3.89E+07	3.53E+07	2.65E+07	2.23E+07	19	19	16	14	10	12	Carbamid
520	881	tr|A0A087WZN1|A0A087WZN1_HUMAN	281.27	3.89E+07	3.53E+07	2.65E+07	2.23E+07	19	19	16	14	10	12	Carbamid
472	854	sp|P82933|RT09_HUMAN	281.16	9.35E+06	2.36E+07	4.28E+07	4.17E+07	21	21	10	12	17	18	Carbamid
519	625	tr|H0Y7R8|H0Y7R8_HUMAN	281.09	3.96E+06	7.97E+06	8.37E+06	6.67E+06	20	1	13	20	13	16	Carbamid
493	557	tr|Q5JWT2|Q5JWT2_HUMAN	280.77	1.22E+07	3.72E+07	1.59E+07	1.79E+07	34	34	16	27	14	15	Carbamid
493	558	sp|Q9ULK4|MED23_HUMAN	280.77	1.22E+07	3.72E+07	1.59E+07	1.79E+07	34	34	16	27	14	15	Carbamid
586	1145	sp|P47755|CAZA2_HUMAN	280.73	6.27E+06	1.53E+07	1.44E+07	1.74E+07	16	13	7	12	7	12	Carbamid
535	514	sp|P83111|LACTB_HUMAN	279.86	2.82E+05	2.60E+07	3.82E+07	4.34E+07	25	25	2	16	12	13	Carbamid
271	2383	sp|P09429|HMGB1_HUMAN	279.71	6.68E+08	2.47E+08	1.74E+08	1.39E+08	24	17	37	22	20	15	Carbamid
220	465	sp|Q9UG63|ABCF2_HUMAN	279.45	7.30E+07	7.16E+07	1.51E+08	1.09E+08	30	30	26	26	32	27	Carbamid
453	1780	sp|Q92572|AP3S1_HUMAN	279.33	2.29E+08	2.57E+08	2.69E+08	2.48E+08	16	16	15	18	17	15	Carbamid
562	533	tr|D3DQV9|D3DQV9_HUMAN	279.23	2.16E+07	1.26E+07	8.80E+06	6.07E+06	32	32	18	15	7	8	Carbamid
562	534	sp|P78344|IF4G2_HUMAN	279.23	2.16E+07	1.26E+07	8.80E+06	6.07E+06	32	32	18	15	7	8	Carbamid
529	589	sp|Q9UI10|EI2BD_HUMAN	278.9	1.94E+07	1.19E+07	1.57E+07	1.46E+07	20	20	13	12	14	13	Carbamid
529	643	tr|E7ERK9|E7ERK9_HUMAN	278.9	1.94E+07	1.19E+07	1.57E+07	1.45E+07	19	19	13	12	14	12	Carbamid
471	1991	sp|Q14696|MESD_HUMAN	278.76	2.20E+08	1.87E+08	9.70E+07	1.24E+08	21	20	18	19	11	14	Carbamid
437	583	sp|O14744|ANM5_HUMAN	278.74	3.33E+07	7.29E+07	3.31E+07	5.34E+07	28	28	16	24	13	15	Carbamid
404	520	tr|A0A087WY71|A0A087WY71_HUMAN	278.58	2.34E+07	4.11E+07	8.01E+07	8.27E+07	24	24	14	13	19	19	Carbamid
404	521	sp|Q96CW1|AP2M1_HUMAN	278.58	2.34E+07	4.11E+07	8.01E+07	8.27E+07	24	24	14	13	19	19	Carbamid
404	525	tr|E9PFW3|E9PFW3_HUMAN	278.58	2.34E+07	4.11E+07	8.01E+07	8.27E+07	24	24	14	13	19	19	Carbamid
211	2252	tr|G8JLA2|G8JLA2_HUMAN	278.52		6.48E+06	2.01E+07	3.81E+06	19	2	15	29	19	23	Carbamid
264	441	sp|O43615|TIM44_HUMAN	278.13	1.86E+08	1.35E+08	1.22E+08	1.15E+08	27	27	28	23	17	23	Carbamid
208	2786	sp|P0DP24|CALM2_HUMAN	278.12	1.60E+08	6.52E+08	2.97E+08	9.60E+08	17	15	16	28	19	30	Acetylatio
208	2787	sp|P0DP23|CALM1_HUMAN	278.12	1.60E+08	6.52E+08	2.97E+08	9.60E+08	17	15	16	28	19	30	Acetylatio
208	2788	sp|P0DP25|CALM3_HUMAN	278.12	1.60E+08	6.52E+08	2.97E+08	9.60E+08	17	15	16	28	19	30	Acetylatio
208	2789	tr|E7ETZ0|E7ETZ0_HUMAN	278.12	1.60E+08	6.52E+08	2.97E+08	9.60E+08	17	15	16	28	19	30	Acetylatio
356	1091	sp|Q96EY1|DNJA3_HUMAN	278.11	1.15E+08	1.83E+08	2.10E+08	1.91E+08	21	21	17	23	22	16	Carbamid
622	385	sp|P53621|COPA_HUMAN	277.76	4.08E+06	2.89E+06	9.92E+06	1.31E+07	33	33	8	7	13	17	Carbamid
861	696	sp|Q9BY77|PDIP3_HUMAN	277.62	3.71E+07	3.08E+07	1.24E+07	2.36E+07	21	21	10	5	3	6	Carbamid
523	5826	sp|P30049|ATPD_HUMAN	277.62	7.84E+07	7.74E+07	1.13E+08	1.14E+08	10	10	17	18	10	14	Deamidat
726	748	sp|Q8WWY3|PRP31_HUMAN	277.59	1.08E+07	1.93E+07	3.56E+07	2.07E+07	17	17	7	13	10	10	Carbamid
634	450	sp|Q9H2K8|TAOK3_HUMAN	277.19	1.89E+06	3.73E+06	1.32E+06	1.40E+07	33	28	5	11	2	12	Carbamid
1175	3687	sp|Q9H1E3|NUCKS_HUMAN	276.82	4.75E+07	4.75E+07	3.92E+07	1.85E+07	10	10	6	4	6	3	
821	1016	sp|A1L0T0|ILVBL_HUMAN	276.77	1.10E+07	9.55E+06	4.98E+06	7.47E+06	20	20	11	11	5	7	Carbamid
195	1268	sp|P18124|RL7_HUMAN	276.73	5.92E+07	1.72E+08	1.22E+09	1.27E+09	23	23	16	21	32	36	Carbamid
662	733	sp|O00425|IF2B3_HUMAN	276.64	1.60E+06	6.17E+06	3.05E+07	3.00E+07	18	16	4	10	14	17	Carbamid
242	1185	sp|Q00325|MPCP_HUMAN	276.34	3.49E+07	3.19E+07	4.26E+07	3.60E+07	22	2	29	25	21	19	Carbamid
573	2899	sp|P63241|IF5A1_HUMAN	276.32	1.28E+08	6.32E+07	7.28E+07	4.48E+07	17	17	20	13	12	10	Carbamid
573	2904	tr|I3L504|I3L504_HUMAN	276.32	1.28E+08	6.32E+07	7.28E+07	4.48E+07	17	17	20	13	12	10	Carbamid
433	673	tr|B7Z645|B7Z645_HUMAN	276.31					21	1	12	11	17	19	Carbamid
696	584	sp|P31040|SDHA_HUMAN	276.31	2.37E+07	2.00E+07	2.88E+07	1.13E+07	20	20	13	10	11	5	Carbamid
332	1663	sp|Q15428|SF3A2_HUMAN	276.3	3.04E+08	5.18E+08	2.12E+08	3.25E+08	20	20	24	29	17	19	Carbamid
272	1701	sp|P09661|RU2A_HUMAN	275.82	3.57E+08	6.13E+08	4.10E+08	4.88E+08	18	18	29	27	20	26	Deamidat
442	1871	sp|Q9UBS4|DJB11_HUMAN	275.42	1.31E+08	1.27E+08	1.82E+08	1.64E+08	16	16	20	14	14	16	Carbamid
233	1432	sp|P36542|ATPG_HUMAN	275.35	3.71E+08	7.93E+08	8.64E+08	1.04E+09	22	22	22	33	22	20	Carbamid
709	1019	sp|Q9UKJ3|GPTC8_HUMAN	275.25	3.59E+07	5.16E+07	3.20E+07	4.72E+07	22	22	16	16	10	12	Carbamid
602	658	sp|Q96P11|NSUN5_HUMAN	275.17	5.55E+06	9.68E+06	4.16E+07	5.34E+07	26	26	6	12	13	18	Carbamid
554	458	sp|O95831|AIFM1_HUMAN	275.17	2.34E+07	2.61E+07	2.02E+07	2.60E+07	23	23	14	15	9	13	Carbamid
326	1783	sp|P62826|RAN_HUMAN	275.14	3.98E+08	3.11E+08	4.34E+08	3.39E+08	27	27	29	22	17	16	Carbamid
1122	1800	sp|Q6UN15|FIP1_HUMAN	275.04	2.24E+07	3.88E+07		1.01E+07	14	6	10	12	1	10	
420	2382	sp|O75947|ATP5H_HUMAN	275.03	1.78E+08	2.39E+08	1.48E+08	2.21E+08	17	17	14	20	12	17	Carbamid
1292	3927	sp|Q96HY6|DDRGK_HUMAN	274.84	1.66E+07	1.64E+07	3.02E+07	7.96E+06	7	7	10	6	5	3	
241	1328	sp|O75937|DNJC8_HUMAN	274.82	4.53E+08	3.48E+08	2.47E+08	2.20E+08	24	24	33	24	27	18	Acetylatio
649	832	sp|P62191|PRS4_HUMAN	274.81	1.30E+07	8.28E+06	1.67E+07	6.02E+06	21	19	10	9	16	10	Carbamid
916	1181	sp|Q8TDN6|BRX1_HUMAN	274.68	5.75E+06	7.27E+06	7.46E+07	4.22E+07	17	17	2	7	13	14	Carbamid
899	735	sp|O43272|PROD_HUMAN	274.61	2.67E+06	6.82E+06	1.78E+06	1.25E+07	19	19	7	8	3	7	Carbamid
899	737	tr|A0A087WWM6|A0A087WWM6_HUMAN	274.61	2.67E+06	6.82E+06	1.78E+06	1.25E+07	19	19	7	8	3	7	Carbamid
375	3420	sp|P05387|RLA2_HUMAN	274.48	1.17E+08	2.13E+08	1.82E+09	1.78E+09	14	12	10	14	20	28	Oxidation
359	772	tr|G5E9V5|G5E9V5_HUMAN	274.16	4.58E+07	4.87E+07	1.05E+08	8.98E+07	20	20	20	17	15	24	Carbamid
359	773	sp|P82650|RT22_HUMAN	274.16	4.58E+07	4.87E+07	1.05E+08	8.98E+07	20	20	20	17	15	24	Carbamid
359	892	tr|G5E9W7|G5E9W7_HUMAN	274.16	4.58E+07	4.87E+07	1.05E+08	8.98E+07	20	20	20	17	15	24	Carbamid
553	916	sp|P49448|DHE4_HUMAN	274.12			2.80E+05		23	2	7	11	11	13	Carbamid
766	823	sp|Q13492|PICAL_HUMAN	274.05	3.28E+07	9.99E+06	9.87E+06	7.73E+06	17	11	17	8	9	7	Deamidat
337	626	sp|P38919|IF4A3_HUMAN	274.05	5.13E+07	8.77E+07	6.86E+07	6.67E+07	27	23	25	20	18	17	Carbamid
483	433	sp|Q6GQQ9|OTU7B_HUMAN	273.99	2.83E+07	2.58E+07	2.69E+07	1.19E+07	30	30	20	15	11	10	Carbamid
394	657	sp|Q10567|AP1B1_HUMAN	273.58	4.29E+06	2.38E+06	3.34E+06	4.23E+06	28	8	15	16	17	20	Carbamid
912	577	sp|P30101|PDIA3_HUMAN	273.31	3.57E+07	5.31E+06	6.61E+06	4.03E+06	19	18	16	6	5	3	Carbamid

389	439	sp|Q8IZ69|TRM2A_HUMAN	273.15	3.86E+06	9.67E+06	8.19E+07	7.28E+07	28	28	8	10	27	24	Carbamid
389	446	tr|F2Z2W7|F2Z2W7_HUMAN	273.15	3.86E+06	9.67E+06	8.19E+07	7.28E+07	28	28	8	10	27	24	Carbamid
697	1341	sp|Q15084|PDIA6_HUMAN	273.15	6.78E+07	3.86E+07	4.73E+07	3.73E+07	15	15	13	11	9	9	Carbamid
448	755	sp|Q8N1G2|CMTR1_HUMAN	272.81	6.81E+06	2.32E+07	1.89E+07	1.88E+07	35	34	10	20	15	17	Carbamid
801	976	sp|Q14192|FHL2_HUMAN	272.79	5.37E+07	3.88E+07	2.01E+07	1.81E+07	18	18	16	14	7	9	Carbamid
801	985	tr|J3KNW4|J3KNW4_HUMAN	272.79	5.37E+07	3.88E+07	2.01E+07	1.81E+07	18	18	16	14	7	9	Carbamid
801	987	tr|A0A0A0MSG2|A0A0A0MSG2_HUMAN	272.79	5.37E+07	3.88E+07	2.01E+07	1.81E+07	18	18	16	14	7	9	Carbamid
479	602	sp|Q13576|IQGA2_HUMAN	272.78	3.07E+06	6.92E+06	1.23E+06	2.02E+06	39	29	16	15	11	9	Carbamid
479	603	tr|F5H7S7|F5H7S7_HUMAN	272.78	3.07E+06	6.92E+06	1.23E+06	2.02E+06	38	28	16	15	11	9	Carbamid
382	851	sp|P35659|DEK_HUMAN	272.53	1.57E+08	2.59E+08	1.52E+08	2.68E+08	32	32	22	21	19	21	Carbamid
654	753	sp|P30837|AL1B1_HUMAN	272.5	1.69E+07	2.34E+07	1.41E+07	3.69E+07	20	19	12	11	12	14	Carbamid
579	551	sp|Q9Y2W1|TR150_HUMAN	272.16	8.93E+07	9.03E+07	3.83E+07	6.54E+07	27	26	28	16	16	15	Deamidat
427	700	sp|Q16795|NDUA9_HUMAN	271.77	6.75E+07	6.70E+07	7.04E+07	5.72E+07	19	18	17	16	14	13	Deamidat
566	1549	sp|Q9H2W6|RM46_HUMAN	271.71	7.84E+07	1.65E+08	1.27E+08	1.25E+08	14	13	13	16	13	11	Carbamid
605	803	sp|A4D1E9|GTPBA_HUMAN	271.55	6.28E+06	9.83E+06	4.84E+07	2.96E+07	22	22	8	13	18	13	Carbamid
949	1956	sp|P29083|T2EA_HUMAN	271.24	1.03E+07	1.09E+07	1.41E+07	7.08E+06	14	14	10	8	6	7	Carbamid
316	728	sp|O00541|PESC_HUMAN	271.23	6.14E+07	1.22E+08	9.53E+07	1.27E+08	27	27	23	21	20	21	Carbamid
795	1798	sp|P82673|RT35_HUMAN	271.17	1.83E+07	1.79E+07	4.33E+07	2.53E+07	13	13	8	9	9	7	Carbamid
510	975	sp|O15020|SPTN2_HUMAN	271.03	1.39E+05	1.58E+05	2.48E+05	6.44E+05	32	22	11	9	12	10	Carbamid
749	1251	tr|A0A0C4DGS1|A0A0C4DGS1_HUMAN	270.91	2.61E+07	3.02E+07	5.63E+07	3.53E+07	13	13	9	9	8	10	Carbamid
749	1254	sp|P39656|OST48_HUMAN	270.91	2.61E+07	3.02E+07	5.63E+07	3.53E+07	13	13	9	9	8	10	Carbamid
680	544	sp|P27694|RFA1_HUMAN	270.9	8.53E+05	1.44E+07	3.30E+06	1.14E+07	24	24	5	13	5	14	Carbamid
511	541	sp|Q8NC60|NOA1_HUMAN	270.75	8.53E+06	2.21E+07	3.59E+07	2.26E+07	23	23	11	17	16	13	Carbamid
592	561	sp|Q15648|MED1_HUMAN	270.66	3.98E+07	3.73E+07	5.25E+07	5.20E+07	34	34	27	18	10	18	Carbamid
699	3066	sp|Q07021|C1QBP_HUMAN	270.6	2.10E+08	2.50E+08	2.22E+08	1.36E+08	14	14	12	13	12	8	Carbamid
681	706	sp|Q8NC56|LEMD2_HUMAN	270.47	4.31E+06	4.15E+07	5.17E+06	4.56E+07	20	20	5	16	3	13	Carbamid
820	6068	sp|P41567|EIF1_HUMAN	270.4	1.95E+07	1.35E+07	1.45E+07	8.71E+06	12	5	14	10	9	6	Carbamid
820	6069	tr|K7EM18|K7EM18_HUMAN	270.4	1.95E+07	1.35E+07	1.45E+07	8.71E+06	12	5	14	10	9	6	Carbamid
810	3206	sp|P27635|RL10_HUMAN	270.39	2.62E+06	4.29E+06	5.31E+07	3.16E+07	14	5	6	7	12	12	Carbamid
810	3277	tr|X1WI28|X1WI28_HUMAN	270.39	2.62E+06	4.29E+06	5.31E+07	3.16E+07	14	5	6	7	12	12	Carbamid
742	1061	sp|Q14444|CAPR1_HUMAN	270.28	4.06E+06	1.08E+07	3.90E+07	4.76E+07	17	17	4	7	11	9	Deamidat
607	1127	sp|Q15785|TOM34_HUMAN	270.27	3.90E+07	2.32E+07	2.51E+07	1.29E+07	20	20	15	14	9	7	Carbamid
491	524	sp|Q9NVH0|EXD2_HUMAN	270.21	1.32E+07	2.14E+07	3.04E+07	2.90E+07	28	28	12	16	14	15	Carbamid
525	609	sp|Q13017|RHG05_HUMAN	269.96	1.60E+07	1.37E+07	2.47E+07	1.78E+07	37	37	17	13	17	19	Carbamid
452	693	sp|Q12904|AIMP1_HUMAN	269.9	1.68E+07	1.54E+07	2.27E+07	2.95E+07	22	21	10	9	14	17	Carbamid
531	1725	sp|Q14847|LASP1_HUMAN	269.77	1.97E+08	1.08E+08	8.06E+07	8.42E+07	16	16	25	13	8	12	Carbamid
615	679	tr|A0A024RAC6|A0A024RAC6_HUMAN	269.76	9.33E+07	8.48E+07	5.79E+07	7.43E+07	26	26	32	16	11	15	Carbamid
424	824	sp|Q96PK6|RBM14_HUMAN	269.71	1.49E+08	1.92E+08	9.37E+07	1.14E+08	28	28	26	19	16	13	Carbamid
547	480	tr|A0A087WVM4|A0A087WVM4_HUMAN	269.69	2.94E+07	1.27E+07	3.25E+07	1.52E+07	25	23	21	12	13	8	Carbamid
547	486	sp|Q6UB35|C1TM_HUMAN	269.69	2.94E+07	1.27E+07	3.25E+07	1.52E+07	25	23	21	12	13	8	Carbamid
547	487	tr|B7ZM99|B7ZM99_HUMAN	269.69	2.94E+07	1.27E+07	3.25E+07	1.52E+07	25	23	21	12	13	8	Carbamid
457	184	sp|Q9Y3Z3|SAMH1_HUMAN	269.43	2.32E+07	2.20E+07	2.76E+07	2.80E+07	27	27	18	15	15	13	Carbamid
455	1411	sp|Q9Y5M8|SRPRB_HUMAN	269.19	1.53E+08	1.50E+08	1.87E+08	1.67E+08	16	16	16	15	16	17	Carbamid
526	703	tr|E9PGZ1|E9PGZ1_HUMAN	269.12	2.40E+06	2.33E+06	2.01E+06	3.23E+06	25	2	17	15	6	12	Deamidat
423	471	sp|P07900|HS90A_HUMAN	269.08	1.56E+07	5.73E+06	3.75E+06	5.03E+06	34	19	23	15	16	10	Carbamid
341	1628	sp|Q96CT7|CC124_HUMAN	268.93	8.30E+08	8.32E+08	7.53E+08	6.11E+08	21	21	22	24	19	22	Oxidation
434	1108	sp|P15880|RS2_HUMAN	268.93	2.97E+07	8.01E+07	3.60E+08	3.49E+08	18	18	9	14	21	23	Carbamid
406	690	sp|Q9H2D6|TARA_HUMAN	268.89	2.48E+07	8.00E+07	2.67E+07	4.34E+07	34	32	18	39	17	22	Carbamid
488	844	sp|Q14677|EPN4_HUMAN	268.58	6.72E+07	6.50E+07	6.27E+07	4.18E+07	22	22	17	16	15	11	
734	1801	sp|O43447|PPIH_HUMAN	268.5	7.18E+07	5.23E+07	7.02E+07	4.06E+07	15	14	14	14	15	8	Carbamid
729	1538	sp|O95400|CD2B2_HUMAN	268.44	5.32E+07	1.06E+08	9.84E+07	8.61E+07	13	13	9	12	11	12	Carbamid
583	1442	sp|P62820|RAB1A_HUMAN	268.32	8.74E+06	2.80E+06	9.15E+06	4.00E+06	15	7	14	12	14	12	Acetylatic
618	955	sp|Q9BWS9|CHID1_HUMAN	268.15	3.93E+07	3.45E+07	2.24E+07	2.09E+07	18	18	14	14	13	11	Carbamid
623	484	sp|Q9UNX4|WDR3_HUMAN	267.61	4.71E+06	8.11E+06	2.72E+07	1.07E+07	27	27	9	14	19	13	Carbamid
386	566	sp|Q9Y305|ACOT9_HUMAN	267.48	2.21E+07	2.55E+07	1.35E+08	1.26E+08	22	22	13	14	26	20	Carbamid
508	701	sp|Q9H0B6|KLC2_HUMAN	267.32	1.23E+07	1.03E+07	8.05E+06	7.76E+06	29	21	19	14	11	7	Carbamid
436	879	sp|Q8WU90|ZC3HF_HUMAN	267.28	2.91E+07	3.56E+07	4.14E+07	3.39E+07	21	21	15	15	17	15	Carbamid
549	949	sp|P28482|MK01_HUMAN	267.14	3.93E+07	2.51E+07	4.56E+07	2.15E+07	20	18	21	12	15	10	Carbamid
700	683	sp|O60645|EXOC3_HUMAN	267.08	1.60E+07	1.26E+07	1.86E+07	1.11E+07	24	24	12	11	11	11	Carbamid
921	1903	sp|Q14257|RCN2_HUMAN	266.79	1.19E+07	1.08E+07	2.31E+07	1.62E+07	14	14	8	7	9	9	
340	644	sp|Q5VTR2|BRE1A_HUMAN	266.74	7.90E+07	4.42E+07	8.38E+07	6.09E+07	37	34	32	23	19	21	Carbamid
561	722	tr|E7EX44|E7EX44_HUMAN	266.58	3.22E+06				24	1	17	14	5	11	Deamidat
564	540	sp|Q13200|PSMD2_HUMAN	266.46	1.88E+07	1.27E+07	2.09E+07	1.61E+07	33	33	15	12	15	11	Carbamid
776	837	sp|Q9NUQ8|ABCF3_HUMAN	266.22	9.70E+06	6.68E+06	1.31E+07	4.60E+06	22	22	13	10	8	4	Carbamid
486	641	sp|O43707|ACTN4_HUMAN	266.17	5.65E+05	5.59E+06	7.73E+05	1.93E+06	32	17	9	18	8	13	Carbamid
474	1330	sp|Q53GQ0|DHB12_HUMAN	266.17	6.93E+07	6.36E+07	9.39E+07	8.77E+07	19	19	18	14	13	17	Carbamid
265	2253	tr|G3V1V0|G3V1V0_HUMAN	266.06			2.02E+07		18	1	15	27	17	21	Carbamid
513	582	sp|Q9BVI4|NOC4L_HUMAN	265.58	1.71E+07	4.07E+07	1.61E+07	3.63E+07	23	23	14	23	10	18	Carbamid
678	457	tr|A0A2R8YD30|A0A2R8YD30_HUMAN	265.58	9.30E+06	1.03E+07	3.56E+06	5.16E+06	27	21	11	14	7	7	Carbamid
678	482	sp|P11171|41_HUMAN	265.58	9.30E+06	1.03E+07	3.56E+06	5.16E+06	27	21	11	14	7	7	Carbamid
617	635	sp|P18031|PTN1_HUMAN	265.39	1.69E+07	2.13E+07	1.64E+07	1.80E+07	20	20	13	11	10	11	Carbamid
644	1957	sp|Q9BQ69|MACD1_HUMAN	265.31	1.50E+08	1.54E+08	1.18E+08	1.20E+08	12	12	18	12	9	9	Carbamid
591	618	tr|F8VXC8|F8VXC8_HUMAN	265.25	3.57E+06	5.91E+06	4.21E+06	1.06E+06	21	14	16	15	11	7	Carbamid
591	619	sp|Q8TAQ2|SMRC2_HUMAN	265.25	3.57E+06	5.91E+06	4.21E+06	1.06E+06	21	14	16	15	11	7	Carbamid
773	1893	tr|C9J9K3|C9J9K3_HUMAN	265.23	1.47E+07	1.98E+07	3.72E+07	3.81E+07	13	13	11	10	8	9	Carbamid
773	1894	sp|P08865|RSSA_HUMAN	265.23	1.47E+07	1.98E+07	3.72E+07	3.81E+07	13	13	11	10	8	9	Carbamid
773	1908	tr|A0A0C4DG17|A0A0C4DG17_HUMAN	265.23	1.47E+07	1.98E+07	3.72E+07	3.81E+07	13	13	11	10	8	9	Carbamid
754	549	tr|B7ZL25|B7ZL25_HUMAN	265.07					26	1	7	8	3	12	Carbamid
754	550	tr|B4E0K6|B4E0K6_HUMAN	265.07					26	1	7	8	3	12	Carbamid
754	552	tr|A0A0G2JL52|A0A0G2JL52_HUMAN	265.07					26	1	7	8	3	12	Carbamid
643	874	sp|Q9Y6I4|UBP3_HUMAN	264.63	1.67E+07	3.42E+07	4.44E+07	1.01E+07	19	19	15	16	13	8	Carbamid
812	1307	sp|Q9NWS0|PIHD1_HUMAN	264.6	2.07E+07	1.18E+07	2.53E+07	7.96E+06	14	14	11	9	10	3	Carbamid
1079	1844	sp|P17643|TYRP1_HUMAN	264.4	1.50E+07	1.13E+07	1.11E+07	2.04E+07	14	14	6	5	6	6	Carbamid
646	1130	sp|O15160|RPAC1_HUMAN	264.38	1.72E+07	2.28E+07	6.09E+07	6.72E+07	15	15	10	9	12	15	Carbamid
719	723	sp|Q9Y512|SAM50_HUMAN	264.33	1.09E+07	1.53E+07	1.12E+07	1.13E+07	22	22	12	14	5	5	Carbamid
536	517	sp|P50991|TCPD_HUMAN	264.18	2.38E+07	1.40E+07	1.42E+07	1.00E+07	24	22	20	16	11	7	Carbamid
585	734	sp|Q8TAF3|WDR48_HUMAN	264.12	2.32E+07	2.50E+07	2.91E+07	1.75E+07	21	21	15	16	13	8	Carbamid
392	2420	sp|P61586|RHOA_HUMAN	263.77	1.74E+07	1.70E+07	1.76E+07	1.46E+07	20	3	19	18	13	20	Carbamid
765	604	tr|A0A140TA45|A0A140TA45_HUMAN	263.65				2.48E+05	26	1	7	8	3	13	Carbamid
765	606	tr|A0A0G2JIA5|A0A0G2JIA5_HUMAN	263.65				2.48E+05	26	1	7	8	3	13	Carbamid
765	608	tr|A0A140TA36|A0A140TA36_HUMAN	263.65				2.48E+05	26	1	7	8	3	13	Carbamid
765	611	tr|A0A140TA40|A0A140TA40_HUMAN	263.65				2.48E+05	26	1	7	8	3	13	Carbamid
765	612	tr|A0A0A0MTG1|A0A0A0MTG1_HUMAN	263.65				2.48E+05	26	1	7	8	3	13	Carbamid
765	614	sp|Q5ST30|SYVM_HUMAN	263.65				2.48E+05	26	1	7	8	3	13	Carbamid
765	615	tr|A0A140T8Y0|A0A140T8Y0_HUMAN	263.65				2.48E+05	26	1	7	8	3	13	Carbamid
765	616	tr|A0A140TA35|A0A140TA35_HUMAN	263.65				2.48E+05	26	1	7	8	3	13	Carbamid
739	490	tr|A0A0G2JN29|A0A0G2JN29_HUMAN	263.53	8.31E+06	8.48E+06	5.78E+06	9.66E+06	26	26	10	11	7	11	Carbamid
739	491	sp|Q15155|NOMO1_HUMAN	263.53	8.31E+06	8.48E+06	5.78E+06	9.66E+06	26	26	10	11	7	11	Carbamid
739	492	tr|A0A087X117|A0A087X117_HUMAN	263.53	8.31E+06	8.48E+06	5.78E+06	9.66E+06	26	26	10	11	7	11	Carbamid
739	493	sp|Q5JPE7|NOMO2_HUMAN	263.53	8.31E+06	8.48E+06	7.70E+06	9.66E+06	27	27	10	11	8	11	Carbamid
739	494	tr|J3KN36|J3KN36_HUMAN	263.53	8.31E+06	8.48E+06	7.70E+06	9.66E+06	27	27	10	11	8	11	Carbamid
739	495	sp|P69849|NOMO3_HUMAN	263.53	8.31E+06	8.48E+06	5.78E+06	9.66E+06	26	26	10	11	7	11	Carbamid
1340	4099	sp|Q9NS69|TOM22_HUMAN	263.5	1.24E+07	1.16E+07	1.28E+07	1.08E+07	7	7	5	7	3	4	Acetylatic
409	572	sp|O60825|F262_HUMAN	263.4	1.69E+07	2.03E+07	3.93E+07	2.62E+07	30	25	15	15	20	15	Carbamid
717	1227	sp|Q9NW64|RBM22_HUMAN	263.32	3.43E+07	4.06E+07	1.55E+07	1.95E+07	18	18	14	17	10	12	Carbamid
541	779	sp|P11310|ACADM_HUMAN	263.26	3.03E+07	3.19E+07	2.25E+07	2.78E+07	22	22	16	15	11	14	Carbamid
541	782	tr|Q5T4U5|Q5T4U5_HUMAN	263.26	3.03E+07	3.19E+07	2.25E+07	2.78E+07	22	22	16	15	11	14	Carbamid
484	1192	sp|P63104|1433Z_HUMAN	263.25	7.91E+07	5.71E+07	5.57E+07	4.85E+07	18	12	17	13	13	14	Carbamid
584	1056	sp|P22087|FBRL_HUMAN	263.18	1.47E+07	4.16E+07	1.30E+08	1.31E+08	19	19	12	13	13	14	Carbamid
559	556	sp|O95602|RPA1_HUMAN	263.14	7.59E+05	3.58E+06	2.44E+07	2.96E+07	33	32	4	9	20	23	Carbamid
822	863	sp|Q8WVM0|TFB1M_HUMAN	262.96	8.05E+05	1.18E+06	3.72E+07	2.51E+07	18	18	4	2	14	12	Carbamid
589	884	sp|Q9Y265|RUVB1_HUMAN	262.63	1.93E+07	2.26E+07	3.53E+07	2.57E+07	18	17	14	16	14	13	Carbamid
867	1173	sp|P67775|PP2AA_HUMAN	262.55	7.20E+06	1.78E+06	1.79E+06	6.84E+05	16	2	10	11	9	4	Carbamid
440	554	sp|O14974|MYPT1_HUMAN	262.45	6.97E+07	1.02E+08	8.96E+07	4.48E+07	32	32	16	25	13	15	Carbamid
831	821	sp|Q16643|DREB_HUMAN	262.4		3.09E+07	9.62E+06	3.72E+07	21	21	0	11	5	11	Carbamid
485	547	sp|O75150|BRE1B_HUMAN	262.15	2.47E+07	2.02E+07	1.32E+07	1.26E+07	36	33	21	18	9	12	Carbamid

447	670	tr|E7EVH7|E7EVH7_HUMAN	261.8	5.97E+06	1.22E+07	4.61E+06	3.12E+05	33	6	22	18	12	7	Carbamid
1065	1058	sp|Q9Y250|LZTS1_HUMAN	261.66	7.61E+06	7.80E+06	4.07E+06	5.40E+06	20	20	7	5	4	6	Carbamid
802	767	sp|Q7Z417|NUFP2_HUMAN	261.55	8.64E+07	7.65E+07	7.84E+07	7.26E+07	18	18	11	15	12	13	Carbamid
277	2301	sp|P62906|RL10A_HUMAN	261.4	1.05E+08	2.44E+08	1.91E+09	1.72E+09	19	19	13	20	30	25	Carbamid
460	4235	tr|E9PB61|E9PB61_HUMAN	261.25	5.31E+07	6.59E+07	2.96E+08	3.05E+08	13	13	9	11	19	21	Acetylatic
415	571	sp|Q9UJV9|DDX41_HUMAN	261.03	2.48E+07	2.67E+07	4.14E+07	3.17E+07	33	33	22	21	20	18	Carbamid
415	575	tr|J3KNN5|J3KNN5_HUMAN	261.03	2.48E+07	2.67E+07	4.14E+07	3.17E+07	33	33	22	21	20	18	Carbamid
1141	2187	tr|F8VY35|F8VY35_HUMAN	260.85	8.08E+06	4.16E+06	5.83E+07	1.65E+07	10	9	4	5	11	7	Carbamid
1141	2188	tr|F5H4R6|F5H4R6_HUMAN	260.85	8.08E+06	4.16E+06	5.83E+07	1.65E+07	10	9	4	5	11	7	Carbamid
1141	2189	sp|P55209|NP1L1_HUMAN	260.85	8.08E+06	4.16E+06	5.83E+07	1.65E+07	10	9	4	5	11	7	Carbamid
1141	2236	tr|F8VV59|F8VV59_HUMAN	260.85	8.08E+06	4.16E+06	5.83E+07	1.65E+07	10	9	4	5	11	7	Carbamid
1141	2237	tr|H0YIV4|H0YIV4_HUMAN	260.85	8.08E+06	4.16E+06	5.83E+07	1.65E+07	10	9	4	5	11	7	Carbamid
507	645	sp|P13639|EF2_HUMAN	260.67	1.53E+07	3.44E+06	1.79E+07	8.33E+06	32	31	19	9	17	9	Carbamid
500	676	tr|G3V325|G3V325_HUMAN	260.64	9.21E+06	1.43E+07	1.10E+07	1.13E+07	25	23	15	16	9	12	Carbamid
570	1045	sp|P55010|IF5_HUMAN	260.49	6.67E+07	4.59E+07	6.42E+07	3.18E+07	16	16	14	11	16	12	Carbamid
854	1122	sp|Q6DD87|ZN787_HUMAN	260.49	3.13E+07	2.77E+07	1.36E+07	2.79E+07	19	19	9	9	7	9	Carbamid
1323	1326	sp|P17028|ZNF24_HUMAN	260.45		4.73E+06	3.45E+06	1.14E+07	15	15	0	3	3	7	Carbamid
685	720	sp|Q9NYV4|CDK12_HUMAN	260.27	1.72E+07	1.55E+07	1.28E+07	1.47E+07	26	19	15	19	13	16	Carbamid
725	993	sp|Q13190|STX5_HUMAN	260.2	1.63E+07	1.22E+07	1.07E+07	1.23E+07	16	16	11	10	6	8	Carbamid
334	3017	sp|P62316|SMD2_HUMAN	259.95	1.35E+09	1.67E+09	1.35E+09	1.27E+09	16	16	22	25	20	21	Carbamid
616	1830	sp|Q9H0U4|RAB1B_HUMAN	259.73	9.12E+06	2.37E+06	4.92E+06	5.96E+06	13	5	16	14	13	11	Acetylatic
850	972	sp|Q8NHQ9|DDX55_HUMAN	259.69	2.05E+07	5.10E+06	2.70E+07	1.57E+07	24	23	10	8	18	14	Carbamid
1176	1756	sp|Q9NQT5|EXOS3_HUMAN	259.62	4.20E+06	4.63E+06	1.39E+07	1.32E+07	14	14	5	4	7	6	Carbamid
587	708	sp|Q53EL6|PDCD4_HUMAN	259.58	2.25E+07	1.46E+07	2.25E+07	1.27E+07	17	17	14	14	12	10	Carbamid
543	660	tr|E9PF19|E9PF19_HUMAN	259.4	6.79E+06	1.28E+07	7.38E+07	4.43E+07	22	22	10	11	17	20	Carbamid
543	663	sp|Q9Y4P3|TBL2_HUMAN	259.4	6.79E+06	1.28E+07	7.38E+07	4.43E+07	22	22	10	11	17	20	Carbamid
590	2001	sp|Q14103|HNRPD_HUMAN	259.35	3.12E+07	2.56E+07	1.80E+08	1.41E+08	14	12	12	9	14	14	Carbamid
454	866	tr|I3L1P8|I3L1P8_HUMAN	259.23	3.37E+07	5.16E+07	5.30E+07	6.32E+07	19	19	13	17	15	15	Carbamid
454	878	sp|Q02978|M2OM_HUMAN	259.23	3.37E+07	5.16E+07	5.30E+07	6.32E+07	19	19	13	17	15	15	Carbamid
400	835	sp|Q13418|ILK_HUMAN	258.99	7.96E+07	6.87E+07	8.45E+07	4.39E+07	23	23	22	21	19	17	Carbamid
400	838	tr|A0A0A0MTH3|A0A0A0MTH3_HUMAN	258.99	7.96E+07	6.87E+07	8.45E+07	4.39E+07	23	23	22	21	19	17	Carbamid
911	1730	sp|Q16576|RBBP7_HUMAN	258.83			5.62E+06	1.17E+06	14	7	5	5	8	7	Carbamid
1583	3852	tr|K7EQF8|K7EQF8_HUMAN	258.58			3.47E+07		9	1	3	2	4	1	Deamidat
674	1272	sp|Q9HCU5|PREB_HUMAN	258.58	4.85E+06	8.13E+06	3.01E+07	2.01E+07	16	16	6	8	15	15	Carbamid
667	586	sp|Q9H6T3|RPAP3_HUMAN	258.37	1.94E+07	1.20E+07	1.32E+07	9.39E+06	22	22	16	12	11	11	Carbamid
625	532	sp|P02538|K2C6A_HUMAN	258.27			9.65E+05		29	3	12	17	12	3	Carbamid
688	1752	sp|Q9NX40|OCAD1_HUMAN	258.17	5.31E+07	5.95E+07	4.79E+07	3.43E+07	16	16	11	12	11	8	Carbamid
743	1309	sp|Q9BYG3|MK67I_HUMAN	257.9	4.60E+07	3.08E+07	3.66E+07	3.72E+07	16	16	15	11	7	12	Carbamid
627	867	sp|Q99873|ANM1_HUMAN	257.78	1.36E+07	1.83E+07	9.94E+06	1.25E+07	21	21	13	14	10	8	Carbamid
627	877	tr|E9PKG1|E9PKG1_HUMAN	257.78	1.36E+07	1.83E+07	9.94E+06	1.25E+07	21	21	13	14	10	8	Carbamid
476	913	sp|Q92552|RT27_HUMAN	257.54	1.45E+07	2.72E+07	7.36E+07	6.28E+07	22	22	8	16	16	20	Carbamid
863	1022	sp|Q15397|PUM3_HUMAN	257.5			4.45E+07	4.81E+07	22	22	0	0	17	18	Carbamid
1091	2879	sp|P29966|MARCS_HUMAN	257.31	4.46E+07	2.86E+07	1.10E+07	1.50E+07	9	9	7	6	3	4	Deamidat
603	843	sp|O14578|CTRO_HUMAN	257	1.40E+07	1.19E+07	2.01E+07	7.14E+06	46	46	20	20	17	9	Carbamid
609	1312	sp|Q06830|PRDX1_HUMAN	256.84	1.33E+08	1.36E+08	5.05E+07	9.78E+07	18	15	14	20	9	13	Carbamid
816	833	sp|P35998|PRS7_HUMAN	256.65	1.69E+07	5.84E+06	6.10E+06	5.76E+06	23	22	13	8	5	8	Carbamid
715	819	sp|Q9H0H5|RGAP1_HUMAN	256.33	3.01E+07	1.74E+07	3.08E+07	1.74E+07	24	24	17	13	12	13	Carbamid
367	1567	sp|Q07955|SRSF1_HUMAN	256.27	3.45E+07	7.19E+07	2.52E+08	3.34E+08	19	17	14	16	22	24	Carbamid
367	1568	tr|J3KTL2|J3KTL2_HUMAN	256.27	3.45E+07	7.19E+07	2.52E+08	3.34E+08	19	17	14	16	22	24	Carbamid
648	537	tr|A0A0A0MSB8|A0A0A0MSB8_HUMAN	256.24	1.61E+07	7.70E+06	6.32E+06	5.52E+06	27	27	17	9	11	6	Carbamid
648	545	sp|Q9UPT5|EXOC7_HUMAN	256.24	1.61E+07	7.70E+06	6.32E+06	5.52E+06	27	27	17	9	11	6	Carbamid
368	1115	sp|Q5T9A4|ATD3B_HUMAN	256.15				4.85E+05	23	2	14	23	19	18	Carbamid
550	699	sp|P26641|EF1G_HUMAN	256.1	4.22E+07	2.55E+07	2.03E+07	1.88E+07	22	22	18	14	10	9	Carbamid
974	1377	sp|Q8IWS0|PHF6_HUMAN	256.08	2.28E+07	2.67E+07	3.60E+07	2.77E+07	15	14	12	8	10	8	Carbamid
974	1378	tr|A0A0D9SGE8|A0A0D9SGE8_HUMAN	256.08	2.28E+07	2.67E+07	3.60E+07	2.77E+07	15	14	12	8	10	8	Carbamid
962	598	sp|Q16666|IF16_HUMAN	255.87		4.35E+06	2.59E+06	1.28E+07	26	26	0	7	3	7	Carbamid
741	1546	sp|Q9HB71|CYBP_HUMAN	255.86	5.96E+07	2.22E+07	1.67E+07	1.59E+07	14	14	11	11	10	10	Carbamid
501	815	sp|P82675|RT05_HUMAN	255.77	5.48E+07	3.56E+07	7.85E+07	5.85E+07	18	18	9	13	15	15	Carbamid
632	981	sp|Q8WYA6|CTBL1_HUMAN	255.75	2.21E+07	3.11E+07	3.09E+07	1.81E+07	18	18	11	17	12	8	Carbamid
632	983	tr|A0A087WUB9|A0A087WUB9_HUMAN	255.75	2.21E+07	3.11E+07	3.09E+07	1.81E+07	18	18	11	17	12	8	Carbamid
498	2980	tr|Q5JR08|Q5JR08_HUMAN	255.56	7.26E+06	7.53E+06	9.89E+06	7.50E+06	19	2	17	16	11	14	Carbamid
498	2981	sp|P08134|RHOC_HUMAN	255.56	7.26E+06	7.53E+06	9.89E+06	7.50E+06	19	2	17	16	11	14	Carbamid
1588	1776	sp|P43897|EFTS_HUMAN	255.28					14	14	0	0	0	0	Carbamid
502	647	sp|O75746|CMC1_HUMAN	255.08	2.96E+06	4.76E+06	4.42E+06	9.74E+06	24	15	15	12	11	17	Carbamid
160	1120	sp|Q562R1|ACTBL_HUMAN	255					32	1	16	40	18	35	Carbamid
873	1030	sp|Q8N7H5|PAF1_HUMAN	254.94	2.48E+07	2.80E+07	2.60E+07	2.22E+07	17	17	8	14	6	10	Carbamid
733	1007	sp|Q9Y6M1|IF2B2_HUMAN	254.92	2.15E+06	1.95E+06	2.99E+07	2.67E+07	17	15	5	5	15	14	Deamidat
733	1008	tr|F8W930|F8W930_HUMAN	254.92	2.15E+06	1.95E+06	2.99E+07	2.67E+07	17	15	5	5	15	14	Deamidat
499	691	sp|P49750|YLPM1_HUMAN	254.67	3.31E+07	2.62E+07	1.35E+07	1.21E+07	34	34	25	17	8	11	Carbamid
494	599	sp|O95793|STAU1_HUMAN	254.51	3.40E+06	8.23E+06	6.20E+07	5.95E+07	22	20	8	15	17	21	Carbamid
1007	1355	sp|P62714|PP2AB_HUMAN	254.31			1.21E+06		15	1	8	9	9	3	Carbamid
249	3437	sp|P62829|RL23_HUMAN	254.05	4.81E+08	7.42E+08	9.32E+08	8.64E+08	16	16	23	33	23	24	Carbamid
574	1028	sp|Q9BWF3|RBM4_HUMAN	253.96	3.07E+07	1.27E+07	2.74E+07	2.43E+07	17	17	16	7	13	13	Carbamid
751	686	sp|Q86W92|LIPB1_HUMAN	253.89	8.93E+06	1.20E+07	3.24E+06	3.27E+06	23	21	13	12	4	5	Carbamid
730	684	sp|P78316|NOP14_HUMAN	253.83	3.99E+07	4.18E+07	3.05E+07	4.95E+07	21	21	11	20	9	15	Carbamid
1076	1982	sp|Q9BYD3|RM04_HUMAN	253.73	2.96E+06	5.08E+06	2.61E+07	1.85E+07	12	12	3	5	9	9	Carbamid
922	1477	sp|Q9NQG5|RPR1B_HUMAN	253.42	1.87E+07	1.22E+07	8.83E+06	7.62E+06	14	10	14	7	6	5	Carbamid
716	1558	sp|Q9UHR5|S30BP_HUMAN	253.15	4.41E+07	3.74E+07	5.82E+07	4.17E+07	14	14	16	15	14	13	Carbamid
942	688	sp|Q6Y7W6|GGYF2_HUMAN	252.67	9.46E+06	1.67E+07	1.32E+05	5.50E+06	22	22	7	13	1	8	Carbamid
942	689	tr|I1E4Y6|I1E4Y6_HUMAN	252.67	9.46E+06	1.67E+07	1.32E+05	5.50E+06	22	22	7	13	1	8	Carbamid
516	883	sp|P48426|PI42A_HUMAN	252.38	5.53E+07	6.95E+07	7.99E+07	6.40E+07	18	11	15	16	15	13	Carbamid
936	1911	sp|P53597|SUCA_HUMAN	252.35	3.79E+07	3.51E+07	3.28E+07	3.28E+07	14	14	9	8	4	5	Carbamid
655	1578	tr|C9JJ19|C9JJ19_HUMAN	252.34	2.16E+07	3.18E+07	5.40E+07	5.98E+07	14	13	11	11	11	16	Carbamid
655	1579	sp|P82930|RT34_HUMAN	252.34	2.16E+07	3.18E+07	5.40E+07	5.98E+07	14	13	11	11	11	16	Carbamid
775	817	sp|Q9UBU9|NXF1_HUMAN	252.34	6.97E+06	8.75E+06	9.32E+06	3.71E+06	20	20	8	11	11	6	Carbamid
849	653	sp|Q7Z2W4|ZCCHV_HUMAN	252.04	1.05E+07	9.00E+06	2.96E+07	1.10E+07	23	23	9	11	13	8	Carbamid
762	846	sp|Q13459|MYO9B_HUMAN	251.68	2.85E+07	2.42E+07	5.26E+07	2.89E+07	25	24	20	12	20	12	Carbamid
762	847	tr|M0R0P8|M0R0P8_HUMAN	251.68	2.85E+07	2.42E+07	5.26E+07	2.89E+07	25	24	20	12	20	12	Carbamid
971	702	sp|P43490|NAMPT_HUMAN	251.63	9.35E+06	3.11E+06	1.81E+06	3.05E+06	21	21	9	6	2	1	Carbamid
837	1321	sp|Q99986|VRK1_HUMAN	251.42	1.33E+07	1.74E+07	1.02E+07	1.27E+07	15	15	10	10	8	7	Carbamid
638	2340	sp|Q9H6E4|CC134_HUMAN	251.19	2.58E+08	2.37E+08	1.44E+08	1.93E+08	15	15	15	12	10	11	Deamidat
728	1741	sp|O75934|SPF27_HUMAN	250.99	2.50E+07	3.21E+07	8.12E+07	1.40E+08	15	15	8	10	14	10	Carbamid
874	1174	sp|Q49A26|GLYR1_HUMAN	250.94	3.56E+07	3.86E+07	3.43E+07	3.47E+07	16	16	12	12	9	12	Carbamid
760	1310	sp|P29084|T2EB_HUMAN	250.9	2.89E+07	2.52E+07	3.73E+07	3.10E+07	15	15	12	10	10	8	Carbamid
639	1262	sp|Q8TCJ2|STT3B_HUMAN	250.88	2.43E+07	2.67E+07	4.55E+07	3.52E+07	15	14	14	11	8	10	Carbamid
604	473	tr|B7ZM87|B7ZM87_HUMAN	250.86	1.18E+07	1.37E+07	9.87E+06	6.04E+06	33	30	19	18	9	9	Carbamid
818	1648	sp|Q15637|SF01_HUMAN	250.84	3.46E+07	4.62E+07	5.08E+07	5.60E+07	13	13	9	11	10	10	Carbamid
395	1633	sp|P24539|AT5F1_HUMAN	250.63	1.73E+08	2.14E+08	1.93E+08	2.43E+08	19	19	15	21	16	19	Oxidatior
630	1828	sp|O75489|NDUS3_HUMAN	250.54	5.68E+07	3.49E+07	6.54E+07	4.14E+07	14	14	12	10	10	14	Deamidat
784	2769	tr|D6R9P3|D6R9P3_HUMAN	250.53	4.22E+07	3.99E+07	1.00E+08	1.04E+08	11	10	9	8	12	11	Carbamid
784	2771	tr|D6RD18|D6RD18_HUMAN	250.53	4.22E+07	3.99E+07	1.00E+08	1.04E+08	11	10	9	8	12	11	Carbamid
784	2773	tr|D6RBZ0|D6RBZ0_HUMAN	250.53	4.22E+07	3.99E+07	1.00E+08	1.04E+08	11	10	9	8	12	11	Carbamid
671	1154	sp|Q8N684|CPSF7_HUMAN	250.23	4.98E+07	1.22E+08	5.40E+07	8.19E+07	17	17	15	17	14	13	
399	1006	sp|Q9NYU1|UGGG2_HUMAN	250.13	5.76E+06	1.23E+07	1.58E+07	2.02E+07	42	41	10	19	15	21	Deamidat
1297	1593	sp|Q92733|PRCC_HUMAN	250.08		7.01E+06		1.54E+06	11	11	0	7	0	2	
829	940	tr|M0QWZ7|M0QWZ7_HUMAN	249.98	3.75E+06	5.66E+06	2.21E+07	1.86E+07	17	16	10	6	9	9	Carbamid
829	941	sp|Q9NP81|SYSM_HUMAN	249.98	3.75E+06	5.66E+06	2.21E+07	1.86E+07	17	16	10	6	9	9	Carbamid
797	681	sp|Q96A65|EXOC4_HUMAN	249.86	6.53E+06	7.36E+06	6.30E+06	8.72E+06	27	27	12	8	5	11	Carbamid
708	1446	sp|Q92979|NEP1_HUMAN	249.78	1.73E+07	2.01E+07	3.86E+07	3.47E+07	15	15	10	9	9	11	Carbamid
763	961	sp|Q9H2P0|ADNP_HUMAN	249.44	1.64E+07	1.81E+07	4.42E+07	2.12E+07	20	20	10	11	14	11	Carbamid
1105	719	sp|O95425|SVIL_HUMAN	249.28	1.01E+05	2.81E+06	7.75E+04	5.04E+06	26	26	1	4	1	6	Carbamid
917	1443	tr|E7ESZ7|E7ESZ7_HUMAN	249.21	2.00E+07	2.05E+07	1.99E+07	1.77E+07	16	16	8	6	6	8	Carbamid
917	1447	sp|O95299|NDUAA_HUMAN	249.21	2.00E+07	2.05E+07	1.99E+07	1.77E+07	16	16	8	6	6	8	Carbamid
917	1457	tr|A0A087WXC5|A0A087WXC5_HUMAN	249.21	2.00E+07	2.05E+07	1.99E+07	1.77E+07	16	16	8	6	6	8	Carbamid
608	1472	sp|P53007|TXTP_HUMAN	249.16	1.21E+08	1.90E+08	2.23E+08	2.54E+08	13	13	13	14	11	13	Carbamid
568	790	sp|O43390|HNRPR_HUMAN	249.12	5.17E+06	7.92E+06	2.44E+07	1.40E+07	20	15	14	13	17	14	Carbamid

1063	4306	tr|B0QYK0|B0QYK0_HUMAN	249.02	5.36E+07	4.64E+07	3.58E+07	4.41E+07	8	8	9	7	6	6	Carbamid
1063	4307	sp|Q01844|EWS_HUMAN	249.02	5.36E+07	4.64E+07	3.58E+07	4.41E+07	8	8	9	7	6	6	Carbamid
1145	4172	sp|Q9NX24|NHP2_HUMAN	249	4.83E+07	4.33E+07	9.62E+07	5.54E+07	12	12	5	5	9	6	Carbamid
903	1885	sp|Q13868|EXOS2_HUMAN	248.96	8.20E+06	1.33E+07	4.38E+07	4.90E+07	11	11	8	7	9	9	Carbamid
1224	1400	sp|P55081|MFAP1_HUMAN	248.87	1.08E+07	3.38E+07	7.82E+06	1.69E+07	14	14	7	13	3	6	Acetylatio
266	1536	sp|Q05639|EF1A2_HUMAN	248.65					16	1	48	24	16	13	Carbamid
266	1539	tr|A0A2U3TZH3|A0A2U3TZH3_HUMAN	248.65					16	1	48	24	16	13	Carbamid
1002	1079	sp|Q9UNE7|CHIP_HUMAN	248.51	8.32E+06	8.40E+06	4.14E+06	6.91E+06	16	16	8	10	4	7	Carbamid
650	859	sp|Q8IXB1|DJC10_HUMAN	248.5	1.75E+07	1.04E+07	1.16E+07	9.10E+06	21	19	13	9	13	11	Carbamid
740	850	sp|P48059|LIMS1_HUMAN	247.95	2.97E+07	2.56E+07	3.51E+07	2.01E+07	20	11	13	10	13	10	Carbamid
997	1575	sp|Q9Y608|LRRF2_HUMAN	247.71	3.49E+05	6.17E+06	1.15E+07	1.58E+07	16	14	1	6	6	6	Deamidat
509	649	sp|Q92889|XPF_HUMAN	247.37	1.25E+07	2.86E+07	5.01E+06	4.68E+06	30	30	19	30	9	7	Carbamid
830	849	sp|Q8IVS2|FABD_HUMAN	247.34	1.16E+07	1.14E+07	1.11E+07	9.36E+06	16	16	12	10	7	5	Carbamid
528	1271	sp|Q16630|CPSF6_HUMAN	247.24	1.78E+08	4.11E+08	4.30E+07	6.40E+07	18	18	19	21	13	10	Carbamid
528	1331	tr|F8WJN3|F8WJN3_HUMAN	247.24	1.78E+08	4.11E+08	4.30E+07	6.40E+07	18	18	19	21	13	10	Carbamid
405	1436	sp|P61289|PSME3_HUMAN	247.15	1.16E+08	1.41E+08	9.62E+07	9.66E+07	16	16	18	21	16	15	Carbamid
594	749	sp|P07237|PDIA1_HUMAN	247.12	2.61E+07	3.27E+07	1.55E+07	1.71E+07	24	24	17	16	8	10	Carbamid
935	1480	sp|Q9BXW7|HDHD5_HUMAN	247.12	1.30E+07	5.38E+06	8.38E+06	2.30E+06	15	15	12	7	6	6	Carbamid
880	1287	sp|Q9BVP2|GNL3_HUMAN	246.69	3.84E+06	7.73E+06	2.41E+07	1.81E+07	16	14	6	6	9	11	Carbamid
687	796	sp|Q5SW79|CE170_HUMAN	246.41	5.19E+07	2.59E+07	5.91E+06	1.89E+07	31	31	19	13	7	10	Carbamid
254	2387	sp|P14678|RSMB_HUMAN	245.96	8.78E+06	9.44E+06	5.90E+06	6.54E+06	21	2	27	30	20	30	Carbamid
581	922	tr|G3V5R9|G3V5R9_HUMAN	245.95	3.95E+05	1.70E+05			28	1	21	14	9	6	Carbamid
817	1038	sp|Q9NXD2|MTMRA_HUMAN	245.54	2.39E+07	9.70E+06	9.96E+06	6.02E+06	20	20	13	10	10	8	Carbamid
759	1248	sp|Q14240|IF4A2_HUMAN	245.34					17	4	10	7	9	10	Carbamid
748	2500	sp|P51970|NDUA8_HUMAN	245.28	1.06E+08	1.06E+08	9.82E+07	9.88E+07	12	12	9	10	10	11	Carbamid
824	3291	sp|Q9Y3E5|PTH2_HUMAN	245.28	4.92E+07	3.92E+07	6.49E+07	4.77E+07	9	9	8	8	9	7	Carbamid
824	3292	tr|J3KQ48|J3KQ48_HUMAN	245.28	4.92E+07	3.92E+07	6.49E+07	4.77E+07	9	9	8	8	9	7	Carbamid
94	2449	sp|P39019|RS19_HUMAN	245.17	3.41E+09	3.52E+09	4.04E+09	4.22E+09	26	26	47	43	43	36	Oxidatio
417	1222	sp|P67936|TPM4_HUMAN	245.08	3.87E+06	2.24E+07	1.34E+07	5.56E+07	19	9	11	16	16	21	Carbamid
832	1594	sp|Q5T3I0|GPTC4_HUMAN	244.96	8.57E+07	8.38E+07	7.04E+07	8.35E+07	16	16	13	10	10	10	Carbamid
767	894	sp|Q9Y285|SYFA_HUMAN	244.82	1.63E+07	1.05E+07	2.18E+07	1.14E+07	17	17	14	7	9	7	Carbamid
1795	4661	sp|Q9Y3S2|ZN330_HUMAN	244.76	2.33E+06	1.59E+06	8.58E+06	1.63E+06	6	6	2	2	5	1	Carbamid
628	860	sp|Q96QC0|PP1RA_HUMAN	244.57	2.07E+07	2.95E+07	1.08E+07	2.45E+07	23	23	15	15	9	15	Carbamid
660	607	sp|Q9Y2A7|NCKP1_HUMAN	244.42	2.17E+07	9.90E+06	1.86E+07	7.47E+06	33	33	18	13	16	11	Carbamid
576	2063	sp|P62241|RS8_HUMAN	244.08	5.23E+07	1.02E+08	6.61E+08	4.96E+08	15	15	9	10	20	13	Carbamid
1259	2792	sp|Q9Y221|NIP7_HUMAN	244.08	2.76E+05	6.71E+05	3.37E+07	3.48E+07	11	11	1	2	8	13	Carbamid
722	1417	sp|Q9Y3B7|RM11_HUMAN	244.06	9.87E+06	4.35E+07	1.21E+08	1.52E+08	14	14	4	10	15	12	Carbamid
506	791	tr|A0A087WYN9|A0A087WYN9_HUMAN	243.87	1.13E+07	1.79E+07	5.78E+06	1.29E+07	34	32	20	21	11	16	Carbamid
745	756	sp|Q8IXH7|NELFD_HUMAN	243.77	1.48E+07	2.04E+07	1.32E+07	9.39E+06	21	21	9	12	8	7	Carbamid
745	757	tr|X6RLT1|X6RLT1_HUMAN	243.77	1.48E+07	2.04E+07	1.32E+07	9.39E+06	21	21	9	12	8	7	Carbamid
745	759	tr|H0UI80|H0UI80_HUMAN	243.77	1.48E+07	2.04E+07	1.32E+07	9.39E+06	21	21	9	12	8	7	Carbamid
267	1525	sp|P54652|HSP72_HUMAN	243.76	6.01E+05		1.17E+06		20	1	30	23	22	20	Carbamid
582	784	sp|P13647|K2C5_HUMAN	243.67	5.97E+06	2.79E+06			31	8	18	19	7	3	Carbamid
580	918	sp|P21127|CD11B_HUMAN	243.24	9.43E+05	2.52E+06	1.42E+06	3.73E+06	27	2	14	17	13	18	Carbamid
694	2422	tr|J3QRS9|J3QRS9_HUMAN	243.21	1.16E+08	1.19E+08	1.00E+08	1.33E+08	15	15	13	15	13	10	Carbamid
565	763	sp|Q5SSJ5|HP1B3_HUMAN	243.17	8.80E+05	1.39E+07	1.45E+08	2.25E+08	20	20	2	6	18	20	Acetylatio
624	895	sp|Q9UQ88|CD11A_HUMAN	243.14		5.18E+05			26	1	12	17	12	15	Carbamid
624	896	tr|Q5QPR3|Q5QPR3_HUMAN	243.14		5.18E+05			26	1	12	17	12	15	Carbamid
980	1073	tr|E7EV99|E7EV99_HUMAN	242.69	1.07E+07	2.43E+07	8.49E+06	1.58E+07	17	17	6	11	7	8	Carbamid
980	1074	tr|E7ENY0|E7ENY0_HUMAN	242.69	1.07E+07	2.43E+07	8.49E+06	1.58E+07	17	17	6	11	7	8	Carbamid
980	1087	sp|P35611|ADDA_HUMAN	242.69	1.07E+07	2.43E+07	8.49E+06	1.58E+07	17	17	6	11	7	8	Carbamid
179	2483	sp|Q9BYX7|ACTBM_HUMAN	242.61			3.11E+07		21	1	15	36	17	26	Carbamid
1293	1293	tr|A0A0B4J2A4|A0A0B4J2A4_HUMAN	242.6	1.80E+07	1.28E+07	1.77E+06	8.18E+06	15	14	11	10	3	5	Carbamid
1293	1294	sp|P42765|THIM_HUMAN	242.6	1.80E+07	1.28E+07	1.77E+06	8.18E+06	15	14	11	10	3	5	Carbamid
614	3166	sp|P62979|RS27A_HUMAN	242.49	1.54E+08	1.57E+08	3.40E+08	3.30E+08	13	13	14	12	13	20	Carbamid
965	1072	sp|P32780|TF2H1_HUMAN	242.45	7.52E+06	1.24E+07	5.62E+06	1.15E+07	16	16	10	11	5	10	Carbamid
1206	1501	tr|M0QXS5|M0QXS5_HUMAN	242.32	8.09E+06	5.87E+06	9.74E+06	9.82E+06	12	11	6	4	5	6	Carbamid
1206	1502	tr|A0A3B3ITJ4|A0A3B3ITJ4_HUMAN	242.32	8.09E+06	5.87E+06	9.74E+06	9.82E+06	12	11	6	4	5	6	Carbamid
1206	1507	sp|P14866|HNRPL_HUMAN	242.32	8.09E+06	5.87E+06	9.74E+06	9.82E+06	12	11	6	4	5	6	Carbamid
1001	2272	sp|Q9Y676|RT18B_HUMAN	242.25	1.13E+07	1.06E+07	2.92E+07	4.51E+07	10	9	6	4	6	10	Carbamid
713	1084	sp|Q9Y6V7|DDX49_HUMAN	242.19	4.53E+06	1.22E+07	2.84E+07	1.92E+07	17	16	9	14	16	14	Carbamid
318	1814	sp|P08579|RU2B_HUMAN	241.98	2.68E+08	3.63E+08	1.97E+08	3.08E+08	23	16	22	33	18	23	Acetylatio
294	3931	sp|P46778|RL21_HUMAN	241.94	1.53E+08	1.55E+08	7.24E+08	6.48E+08	23	23	19	20	20	29	Oxidatio
684	727	sp|Q9Y230|RUVB2_HUMAN	241.71	2.66E+07	2.28E+07	2.70E+07	2.80E+07	21	20	14	12	9	10	Carbamid
815	865	sp|P62195|PRS8_HUMAN	241.42	8.94E+06	6.35E+06	1.80E+06	2.91E+06	20	18	15	10	4	8	Carbamid
558	1706	sp|P19404|NDUV2_HUMAN	241.3	4.75E+07	3.25E+07	3.09E+07	3.03E+07	14	14	11	17	11	14	Carbamid
558	1707	tr|E7EPT4|E7EPT4_HUMAN	241.3	4.75E+07	3.25E+07	3.09E+07	3.03E+07	14	14	11	17	11	14	Carbamid
906	726	tr|B1ALK7|B1ALK7_HUMAN	241.18	1.42E+07	1.13E+07	9.18E+06	3.19E+06	23	23	12	8	7	7	Carbamid
906	730	tr|A0A2R8YG42|A0A2R8YG42_HUMAN	241.18	1.42E+07	1.13E+07	9.18E+06	3.19E+06	23	23	12	8	7	7	Carbamid
1194	1451	sp|Q9NUD5|ZCHC3_HUMAN	241.12	2.90E+05	1.84E+06	9.85E+06	3.57E+06	13	13	2	3	8	3	Carbamid
793	797	sp|Q9UEY8|ADDG_HUMAN	241.09	4.60E+07	2.08E+07	1.30E+07	1.86E+07	19	19	10	15	11	12	Carbamid
546	946	tr|E7EPN9|E7EPN9_HUMAN	240.98	8.90E+07	6.24E+07	3.73E+07	4.26E+07	34	32	27	19	12	15	Carbamid
546	947	sp|Q9Y520|PRC2C_HUMAN	240.98	8.90E+07	6.24E+07	3.73E+07	4.26E+07	34	32	27	19	12	15	Carbamid
703	1180	sp|P24941|CDK2_HUMAN	240.56	2.91E+07	1.78E+07	2.13E+07	1.32E+07	15	13	11	15	8	6	Carbamid
703	1182	tr|G3V5T9|G3V5T9_HUMAN	240.56	2.91E+07	1.78E+07	2.13E+07	1.32E+07	15	13	11	15	8	6	Carbamid
808	1098	sp|Q15365|PCBP1_HUMAN	240.47	2.36E+07	2.06E+07	1.89E+07	1.94E+07	15	12	13	12	6	6	Carbamid
621	1261	sp|Q92759|TF2H4_HUMAN	240.15	3.92E+07	5.52E+07	5.54E+07	5.17E+07	13	13	12	13	12	11	Carbamid
887	886	sp|Q8IWZ8|SUGP1_HUMAN	240	1.54E+07	1.31E+07	2.53E+06	4.79E+06	17	17	12	12	2	7	
750	751	tr|A0A0B4J1W3|A0A0B4J1W3_HUMAN	239.99	4.96E+07	3.24E+07	4.49E+07	3.69E+07	31	28	17	6	10	5	Carbamid
750	752	sp|Q9BXJ9|NAA15_HUMAN	239.99	4.96E+07	3.24E+07	4.49E+07	3.69E+07	31	28	17	6	10	5	Carbamid
845	1564	tr|B1AK87|B1AK87_HUMAN	239.93		5.32E+06		1.06E+07	18	3	4	11	6	10	Carbamid
845	1570	tr|B1AK88|B1AK88_HUMAN	239.93		5.32E+06		1.06E+07	18	3	4	11	6	10	Carbamid
540	682	sp|Q93009|UBP7_HUMAN	239.82	8.16E+06	1.52E+07	1.67E+07	1.52E+07	34	34	12	16	15	19	Carbamid
876	2526	sp|P67870|CSK2B_HUMAN	239.81	3.08E+07	3.04E+07	3.40E+07	2.51E+07	11	11	10	7	6	8	Carbamid
876	2543	tr|Q5SRQ6|Q5SRQ6_HUMAN	239.81	3.08E+07	3.04E+07	3.40E+07	2.51E+07	11	11	10	7	6	8	Carbamid
1061	1729	sp|P50402|EMD_HUMAN	239.77	1.24E+07	1.88E+07	8.19E+06	1.28E+07	14	14	10	5	5	4	Carbamid
537	1117	sp|Q9NPH3|IL1AP_HUMAN	239.77	5.33E+07	3.45E+07	4.52E+07	2.40E+07	21	21	16	19	15	11	Carbamid
724	887	tr|E9PC69|E9PC69_HUMAN	239.62	1.47E+07	1.61E+07	5.69E+06	1.79E+07	25	18	14	12	9	10	Carbamid
724	888	sp|Q7KZI7|MARK2_HUMAN	239.62	1.47E+07	1.61E+07	5.69E+06	1.79E+07	25	18	14	12	9	10	Carbamid
835	1052	sp|Q969V3|NCLN_HUMAN	239.38	1.11E+07	8.13E+06	1.69E+07	1.38E+07	19	19	9	6	12	8	Deamidat
1290	2766	sp|Q5JTH9|RRP12_HUMAN	239.37	4.57E+07	4.27E+07	6.60E+07	3.54E+07	14	14	6	7	11	5	Carbamid
515	2604	sp|P61313|RL15_HUMAN	239.36	4.63E+07	1.02E+08	8.82E+08	8.44E+08	15	15	11	13	18	16	Carbamid
668	766	tr|G3V5V3|G3V5V3_HUMAN	239.23	4.97E+07	3.18E+07	4.61E+07	4.16E+07	28	28	17	15	15	12	Carbamid
668	770	sp|O60524|NEMF_HUMAN	239.23	4.97E+07	3.18E+07	4.61E+07	4.16E+07	28	28	17	15	15	12	Carbamid
473	1664	tr|J3QLS3|J3QLS3_HUMAN	239.12	5.70E+07	8.45E+07	1.37E+08	1.46E+08	15	15	13	14	18	17	Carbamid
473	1694	sp|Q9Y2R9|RT07_HUMAN	239.12	5.70E+07	8.45E+07	1.37E+08	1.46E+08	15	15	13	14	18	17	Carbamid
711	845	sp|O15440|MRP5_HUMAN	239.06	7.49E+06	6.41E+06	9.40E+06	8.17E+06	26	24	12	11	11	8	Carbamid
279	5528	sp|P62306|RUXF_HUMAN	239.02	2.45E+08	3.53E+08	3.48E+08	3.07E+08	17	17	23	32	22	19	Carbamid
656	1037	tr|A6PW57|A6PW57_HUMAN	238.85	7.64E+06	6.02E+06	2.60E+07	7.48E+06	17	7	14	15	14	8	Carbamid
677	716	sp|O95239|KIF4A_HUMAN	238.73	7.80E+06	6.97E+06	6.60E+06	5.37E+06	25	25	14	10	8	9	Carbamid
1223	876	sp|Q8NBP0|TTC13_HUMAN	238.54	1.11E+05	5.54E+05	1.17E+06	5.62E+07	19	19	1	2	3	10	Carbamid
924	1917	sp|P55795|HNRH2_HUMAN	238.37	5.08E+06	5.24E+06	2.81E+05	1.42E+06	11	5	9	8	4	7	Carbamid
814	718	sp|Q9H9Y6|RPA2_HUMAN	238.35	2.19E+06	7.72E+06	1.43E+07	2.33E+07	25	25	4	11	11	17	Carbamid
1109	1399	sp|Q9BQP7|MGME1_HUMAN	238.33	2.25E+06	5.10E+06	2.00E+06	3.28E+06	17	17	4	7	1	3	Carbamid
1250	2586	tr|A0A0A0MRJ6|A0A0A0MRJ6_HUMAN	238.27	1.32E+07	1.63E+07	9.99E+06	5.36E+06	10	10	8	9	3	5	Carbamid
1250	2587	tr|H7BY58|H7BY58_HUMAN	238.27	1.32E+07	1.63E+07	9.99E+06	5.36E+06	10	10	8	9	3	5	Carbamid
1250	2589	sp|P22061|PIMT_HUMAN	238.27	1.32E+07	1.63E+07	9.99E+06	5.36E+06	10	10	8	9	3	5	Carbamid
900	1584	sp|Q8TBX8|PI42C_HUMAN	238.27	3.01E+07	2.85E+07	1.96E+07	1.59E+07	14	13	10	11	6	6	Carbamid
761	768	sp|Q9UKE5|TNIK_HUMAN	238.11	2.31E+07	2.23E+07	2.20E+07	1.10E+07	21	15	19	18	11	9	Carbamid
883	1749	sp|P47756|CAPZB_HUMAN	238.08	1.26E+06	3.01E+06	2.14E+06	4.66E+06	16	1	5	11	7	8	Carbamid
548	820	tr|A0A3B3ISJ1|A0A3B3ISJ1_HUMAN	237.86	3.26E+07	1.47E+07	2.49E+07	1.52E+07	20	20	19	9	13	9	Carbamid
548	822	sp|P07225|PROS_HUMAN	237.86	3.26E+07	1.47E+07	2.49E+07	1.52E+07	20	20	19	9	13	9	Carbamid
548	841	tr|A0A0S2Z4L3|A0A0S2Z4L3_HUMAN	237.86	3.26E+07	1.47E+07	2.49E+07	1.52E+07	20	20	19	9	13	9	Carbamid
329	3434	sp|P62750|RL23A_HUMAN	237.82	2.73E+08	4.11E+08	1.15E+09	1.14E+09	17	17	16	32	24	23	Oxidatio
823	1618	sp|Q9UH62|ARMX3_HUMAN	237.63	2.47E+07	2.55E+07	5.51E+07	2.99E+07	11	11	9	12	11	6	Carbamid
237	2968	sp|P46783|RS10_HUMAN	237.42	4.69E+08	4.93E+08	5.34E+08	4.56E+08	16	3	27	27	17	24	Oxidatio

1013	1229	sp|Q9BQ67|GRWD1_HUMAN	237.33	1.14E+06	6.64E+06	2.39E+07	2.06E+07	16	16	2	6	10	10	Carbamid
626	908	tr|G3XAH0|G3XAH0_HUMAN	237.29	3.97E+07	3.12E+07	1.71E+07	8.87E+06	19	17	21	12	8	9	Carbamid
930	1329	sp|O15143|ARC1B_HUMAN	237.08	1.86E+06	4.27E+06	4.93E+06	8.15E+06	14	4	4	7	13	8	Carbamid
747	1986	sp|Q3ZCQ8|TIM50_HUMAN	237.06	3.16E+07	3.57E+07	3.60E+07	4.60E+07	12	12	11	10	8	12	Carbamid
919	1168	sp|Q13888|TF2H2_HUMAN	237.01			8.88E+05		17	1	11	15	12	5	Carbamid
659	978	sp|Q9UNQ2|DIM1_HUMAN	236.77	1.08E+07	1.57E+07	6.76E+07	5.17E+07	16	16	8	11	12	14	Carbamid
756	1231	sp|Q6UXN9|WDR82_HUMAN	236.7	1.36E+07	2.47E+07	2.07E+07	2.16E+07	15	15	12	10	8	9	Carbamid
563	807	sp|P05455|LA_HUMAN	236.65	8.79E+06	1.48E+07	3.75E+07	3.98E+07	22	22	10	15	14	18	Carbamid
710	872	sp|O94776|MTA2_HUMAN	236.42	6.85E+06	7.44E+06	1.50E+07	1.11E+07	21	18	9	9	9	10	Carbamid
1444	4441	tr|C9JQV0|C9JQV0_HUMAN	236.12	4.82E+06	8.93E+06	1.03E+08	1.03E+08	8	8	4	4	5	6	Carbamid
1444	4442	sp|Q9BRJ6|CG050_HUMAN	236.12	4.82E+06	8.93E+06	1.03E+08	1.03E+08	8	8	4	4	5	6	Carbamid
990	1996	sp|Q15024|EXOS7_HUMAN	235.48	2.21E+06	7.74E+06	4.19E+07	4.14E+07	11	11	2	6	10	13	Carbamid
1169	1474	sp|P24752|THIL_HUMAN	235.39	1.53E+07	1.22E+07	4.18E+06	4.80E+06	15	15	9	9	6	5	Carbamid
826	1471	sp|Q9H501|ESF1_HUMAN	235.36	7.43E+07	3.89E+07	2.77E+07	2.29E+07	19	19	20	18	9	10	Oxidation
969	1220	sp|O00148|DX39A_HUMAN	234.78	1.31E+06		1.14E+06		14	5	10	6	8	6	Carbamid
886	1824	sp|Q9NZ63|TLS1_HUMAN	234.78	2.36E+07	1.73E+07	1.87E+07	9.25E+06	15	15	11	8	7	6	Carbamid
1313	2838	sp|Q9NQT4|EXOS5_HUMAN	234.74		5.49E+06	3.31E+07	1.88E+07	8	8	0	4	7	5	Carbamid
736	869	tr|B7ZC06|B7ZC06_HUMAN	234.73					18	2	11	13	12	8	Carbamid
932	1410	sp|P61160|ARP2_HUMAN	234.7	5.73E+05	2.31E+06		3.56E+06	15	3	6	12	4	9	Carbamid
1022	1206	sp|Q9NZU5|LMCD1_HUMAN	234.45	5.18E+07	8.00E+06	2.80E+07	8.88E+06	19	19	16	5	10	7	Carbamid
1419	1235	tr|G3V1N3|G3V1N3_HUMAN	234.44	2.15E+07	1.09E+07	3.58E+06	9.17E+06	15	15	9	5	3	3	Carbamid
1419	1236	sp|Q86XJ1|GA2L3_HUMAN	234.44	2.15E+07	1.09E+07	3.58E+06	9.17E+06	15	15	9	5	3	3	Carbamid
987	1789	sp|Q8IYU8|MICU2_HUMAN	234.28	2.00E+07	1.07E+07	1.48E+07	4.64E+06	12	12	12	8	8	5	Carbamid
878	1497	sp|P78346|RPP30_HUMAN	234.13	2.54E+06	6.07E+06	2.44E+07	3.07E+07	14	14	8	6	8	11	Carbamid
943	1105	sp|Q9Y2X0|MED16_HUMAN	234.11	2.27E+05	1.19E+07	6.04E+06	5.18E+06	21	7	2	17	7	10	Carbamid
690	2718	sp|O60573|IF4E2_HUMAN	234.09		1.93E+06		3.68E+05	14	2	14	12	10	12	Oxidation
959	959	sp|Q86V48|LUZP1_HUMAN	234.06	2.68E+07	2.82E+07	2.45E+07	2.17E+07	20	20	11	10	11	11	
1059	1335	sp|P49005|DPOD2_HUMAN	234.06	1.86E+06	6.45E+06	1.54E+06	1.07E+07	14	14	4	9	2	6	Carbamid
1059	1336	tr|A0A087WWF6|A0A087WWF6_HUMAN	234.06	1.86E+06	6.45E+06	1.54E+06	1.07E+07	14	14	4	9	2	6	Carbamid
843	2763	sp|P11233|RALA_HUMAN	233.85	3.20E+07	3.33E+07	3.16E+07	3.14E+07	10	5	11	6	8	9	Oxidation
705	1456	sp|Q9UKD2|MRT4_HUMAN	233.79	3.60E+06	1.26E+07	8.98E+07	1.05E+08	14	14	5	7	13	14	Carbamid
611	1659	tr|A0A087X2D5|A0A087X2D5_HUMAN	233.78	2.36E+07	3.43E+07	4.82E+07	3.72E+07	12	12	10	16	14	13	Carbamid
611	1660	sp|Q9BRJ2|RM45_HUMAN	233.78	2.36E+07	3.43E+07	4.82E+07	3.72E+07	12	12	10	16	14	13	Carbamid
282	2508	sp|P63162|RSMN_HUMAN	233.59	6.70E+07	6.45E+07	4.33E+07	4.65E+07	20	1	26	30	19	29	Carbamid
282	2556	tr|J3QLE5|J3QLE5_HUMAN	233.59	6.70E+07	6.45E+07	4.33E+07	4.65E+07	20	1	26	30	19	29	Carbamid
882	900	sp|Q9UDY2|ZO2_HUMAN	233.51	1.68E+07	1.49E+07	3.46E+06	9.11E+05	26	26	19	14	6	4	Deamidat
882	901	tr|A0A3B3ITE1|A0A3B3ITE1_HUMAN	233.51	1.62E+07	1.49E+07	3.46E+06	9.11E+05	25	25	18	14	6	4	Deamidat
882	902	tr|A0A2R8YDH4|A0A2R8YDH4_HUMAN	233.51	1.62E+07	1.49E+07	3.46E+06	9.11E+05	25	25	18	14	6	4	Deamidat
882	903	tr|A0A1B0GTW1|A0A1B0GTW1_HUMAN	233.51	1.62E+07	1.49E+07	3.46E+06	9.11E+05	25	25	18	14	6	4	Deamidat
1181	1208	sp|Q8IY81|SPB1_HUMAN	233.45	9.98E+06	5.79E+06	2.29E+07	2.43E+06	16	16	9	6	10	4	Carbamid
996	3370	sp|P01116|RASK_HUMAN	233.45	2.34E+07	2.49E+07	1.89E+07	2.18E+07	10	3	7	7	9	6	Carbamid
857	2379	sp|Q9P035|HACD3_HUMAN	233.36	1.34E+07	2.63E+07	7.27E+07	8.57E+07	13	12	8	9	9	10	Acetylatic
1229	2201	sp|Q13155|AIMP2_HUMAN	233.11	4.34E+06	5.49E+06	4.80E+06	5.44E+06	10	10	6	7	2	4	Carbamid
1118	2139	sp|P62873|GBB1_HUMAN	233.01	3.58E+06	3.28E+06	8.81E+06	3.76E+06	11	6	7	7	7	6	Carbamid
856	754	sp|Q9NR50|EI2BG_HUMAN	232.66	5.25E+06	5.36E+06	1.16E+07	5.82E+06	18	18	8	7	7	5	Carbamid
1135	1366	sp|Q8N163|CCAR2_HUMAN	232.6	1.64E+07	1.06E+07	5.10E+06	2.81E+06	19	19	10	8	3	5	Carbamid
1220	798	tr|A0A0U1RQF0|A0A0U1RQF0_HUMAN	232.48	3.16E+06	2.85E+06	4.55E+06	4.00E+06	23	23	4	4	4	3	Carbamid
1220	799	sp|P49327|FAS_HUMAN	232.48	3.16E+06	2.85E+06	4.55E+06	4.00E+06	23	23	4	4	4	3	Carbamid
786	1350	sp|Q9P2N5|RBM27_HUMAN	232.29	3.28E+07	2.85E+07	1.67E+07	2.43E+07	23	23	18	13	10	12	Carbamid
913	925	sp|Q9P246|STIM2_HUMAN	232.25	1.06E+07	4.84E+06	6.73E+06	5.38E+06	19	18	14	10	5	10	Carbamid
913	926	tr|A0A1X7SBY3|A0A1X7SBY3_HUMAN	232.25	1.06E+07	4.84E+06	6.73E+06	5.38E+06	19	18	14	10	5	10	Carbamid
913	927	tr|H0Y860|H0Y860_HUMAN	232.25	1.06E+07	4.84E+06	6.73E+06	5.38E+06	19	18	14	10	5	10	Carbamid
913	928	tr|A0A1W2PRA1|A0A1W2PRA1_HUMAN	232.25	1.06E+07	4.84E+06	6.73E+06	5.38E+06	19	18	14	10	5	10	Carbamid
1448	3053	sp|P56537|IF6_HUMAN	232.04	3.55E+05	1.08E+05	3.40E+07	1.52E+07	8	8	1	2	8	4	Carbamid
884	1027	sp|Q9H845|ACAD9_HUMAN	231.91	1.02E+07	9.33E+06	1.42E+07	1.16E+07	18	17	9	9	8	8	Carbamid
467	3497	sp|P26583|HMGB2_HUMAN	231.74	1.46E+08	6.15E+07	4.38E+07	5.12E+07	17	10	22	15	15	11	Carbamid
938	2280	sp|P52597|HNRPF_HUMAN	231.37	1.90E+06	5.54E+06	7.02E+06	6.61E+06	11	9	7	9	7	6	Carbamid
992	3757	sp|P11234|RALB_HUMAN	231.15	7.64E+06	4.39E+06	5.17E+06	1.79E+06	9	4	8	5	7	6	Oxidation
533	1971	sp|Q9NX20|RM16_HUMAN	231.14	1.64E+08	2.07E+08	2.42E+08	2.57E+08	14	14	12	13	15	16	Carbamid
794	1792	sp|Q9Y314|NOSIP_HUMAN	230.89	2.86E+07	2.56E+07	1.66E+07	1.74E+07	15	15	12	16	6	9	Carbamid
794	1793	tr|A0A075B6F9|A0A075B6F9_HUMAN	230.89	2.86E+07	2.56E+07	1.66E+07	1.74E+07	15	15	12	16	6	9	Carbamid
223	6084	sp|P62304|RUXE_HUMAN	230.62	3.78E+08	5.02E+08	4.85E+08	3.29E+08	13	13	43	42	18	13	Carbamid
1052	4628	sp|Q14011|CIRBP_HUMAN	230.6	2.42E+07	2.89E+07	3.03E+07	3.81E+07	7	7	8	8	5	7	Deamidat
497	2751	sp|P55145|MANF_HUMAN	230.41	2.50E+08	1.38E+08	9.12E+07	1.12E+08	16	16	16	20	10	14	Carbamid
497	2752	tr|A8K878|A8K878_HUMAN	230.41	2.50E+08	1.38E+08	9.12E+07	1.12E+08	16	16	16	20	10	14	Carbamid
979	1731	sp|Q99848|EBP2_HUMAN	230.38	2.27E+06	1.82E+06	2.34E+07	2.63E+07	13	13	3	4	11	12	Carbamid
979	1751	tr|H7C2Q8|H7C2Q8_HUMAN	230.38	2.27E+06	1.82E+06	2.34E+07	2.63E+07	13	13	3	4	11	12	Carbamid
1010	5021	sp|O60739|EIF1B_HUMAN	230.33	2.48E+06	1.82E+06		1.12E+06	9	2	11	8	6	6	Carbamid
311	1636	sp|Q8TD47|RS4Y2_HUMAN	229.93				3.75E+05	20	1	20	15	23	28	Carbamid
1500	2297	sp|Q9BW19|KIFC1_HUMAN	229.9	2.87E+05	1.43E+06	1.35E+07	1.10E+07	15	14	2	3	7	10	Carbamid
757	1510	sp|O60884|DNJA2_HUMAN	229.84	1.22E+07	1.75E+07	2.41E+07	1.25E+07	15	15	11	16	10	6	Carbamid
419	2542	sp|P30050|RL12_HUMAN	229.73	2.81E+08	3.00E+08	1.63E+09	1.49E+09	14	14	12	14	16	21	Carbamid
860	1029	sp|Q99543|DNJC2_HUMAN	229.66	9.00E+06	5.34E+06	1.12E+07	6.64E+06	16	16	11	6	10	10	Carbamid
714	2723	tr|B8ZZ50|B8ZZ50_HUMAN	229.59	5.32E+05	6.62E+05			13	1	15	12	10	11	Oxidation
889	1167	sp|Q6P1K8|T2H2L_HUMAN	229.4		3.90E+05			17	1	11	17	11	5	Carbamid
571	2275	sp|P62913|RL11_HUMAN	229.38	1.28E+08	1.45E+08	4.65E+08	3.66E+08	14	14	10	8	19	11	Carbamid
1121	1629	sp|Q9BZE1|RM37_HUMAN	229.25	4.34E+06	8.36E+06	7.67E+06	1.13E+07	15	15	5	10	4	6	Carbamid
967	1316	sp|P55036|PSMD4_HUMAN	228.92	1.63E+07	1.03E+07	7.01E+06	5.74E+06	13	8	12	10	5	5	Carbamid
804	1580	sp|Q9NR56|MBNL1_HUMAN	228.72	7.67E+06	8.86E+06	5.76E+06	5.03E+06	13	3	17	14	8	8	Carbamid
804	1583	tr|A0A0A0MQX8|A0A0A0MQX8_HUMAN	228.72	7.67E+06	8.86E+06	5.76E+06	5.03E+06	13	3	17	14	8	8	Carbamid
846	1269	tr|E7ENZ3|E7ENZ3_HUMAN	228.67	9.98E+06	5.25E+06	6.29E+06	2.62E+06	17	16	16	11	7	3	Carbamid
846	1276	sp|P48643|TCPE_HUMAN	228.67	9.98E+06	5.25E+06	6.29E+06	2.62E+06	17	16	16	11	7	3	Carbamid
1031	1750	sp|O75616|ERAL1_HUMAN	228.54	6.63E+06	3.87E+06	1.14E+07	4.75E+06	11	11	9	5	9	4	Carbamid
1075	2732	sp|P82663|RT25_HUMAN	228.29	6.04E+06	1.13E+07	2.36E+07	2.67E+07	9	9	6	5	8	6	Carbamid
641	5781	sp|P62318|SMD3_HUMAN	228.28	5.31E+08	6.70E+08	7.09E+08	8.28E+08	10	10	13	16	10	14	Carbamid
789	1655	sp|Q13435|SF3B2_HUMAN	227.89	4.41E+07	2.13E+07	2.80E+07	8.83E+06	19	19	13	16	9	7	
752	813	sp|O00471|EXOC5_HUMAN	227.8	1.33E+07	8.77E+06	1.07E+07	3.30E+06	21	21	13	12	7	7	Carbamid
752	814	tr|A0A0A0MSI8|A0A0A0MSI8_HUMAN	227.8	1.33E+07	8.77E+06	1.07E+07	3.30E+06	21	21	13	12	7	7	Carbamid
960	891	sp|Q29RF7|PDS5A_HUMAN	227.72	2.16E+06	1.65E+06	3.97E+06	8.37E+06	21	13	4	4	5	7	Carbamid
986	1179	sp|Q9Y5X1|SNX9_HUMAN	227.69	1.12E+07	1.20E+07	9.73E+06	6.35E+06	16	16	11	9	5	4	Carbamid
800	920	sp|Q9UGP8|SEC63_HUMAN	227.6	9.08E+06	4.44E+06	8.91E+06	7.78E+06	19	19	7	5	7	9	Carbamid
735	1445	sp|P61106|RAB14_HUMAN	227.41	1.38E+07	1.32E+07	1.05E+07	1.38E+07	12	11	14	11	9	10	Carbamid
910	1070	sp|P30153|2AAA_HUMAN	227.18	1.28E+07	1.20E+07	1.33E+07	8.39E+06	18	18	7	12	7	6	Carbamid
1265	1540	sp|Q9Y2R4|DDX52_HUMAN	227.18	3.47E+06	1.56E+06	1.35E+07	8.62E+06	14	14	9	3	10	8	Carbamid
1099	2028	sp|O00257|CBX4_HUMAN	227.14	2.68E+07	3.29E+07	2.57E+07	2.84E+07	11	11	9	9	8	10	Carbamid
1081	2592	sp|P05026|AT1B1_HUMAN	227.13	4.70E+07	2.74E+07	3.51E+07	3.26E+07	8	8	7	9	7	6	Carbamid
828	1288	sp|Q9BXI6|TB10A_HUMAN	227.02	1.34E+07	1.60E+07	1.76E+07	1.07E+07	18	16	10	10	14	5	Carbamid
1087	2218	tr|D6RIY6|D6RIY6_HUMAN	227.01	2.92E+06	9.54E+06	3.22E+07	2.07E+07	12	11	6	8	7	4	Carbamid
1087	2219	sp|Q06265|EXOS9_HUMAN	227.01	2.92E+06	9.54E+06	3.22E+07	2.07E+07	12	11	6	8	7	4	Carbamid
772	1770	sp|Q52LJ0|FA98B_HUMAN	226.83	1.69E+07	2.31E+07	2.56E+07	1.78E+07	13	11	10	12	11	6	Carbamid
1005	2215	tr|A9IYQ1|A9IYQ1_HUMAN	226.6	1.62E+06	3.85E+05	4.45E+05	1.45E+06	10	2	10	10	3	6	Carbamid
944	1194	sp|Q9BZE4|NOG1_HUMAN	226.56	9.62E+06	9.44E+06	2.69E+07	1.42E+07	18	18	13	11	12	12	Carbamid
805	1757	sp|Q6P2C8|MED27_HUMAN	226.27	2.13E+07	4.37E+07	3.38E+07	3.88E+07	12	12	11	12	11	13	Carbamid
1513	1839	sp|Q9H7Z7|PGES2_HUMAN	226.19	5.52E+06	6.77E+06	1.19E+06	1.37E+06	11	11	4	6	1	3	Carbamid
673	2393	tr|A0A0C4DGB6|A0A0C4DGB6_HUMAN	226.19	8.89E+04	5.02E+05		2.63E+05	14	2	13	12	9	13	Carbamid
673	2417	sp|P02768|ALBU_HUMAN	226.19	8.89E+04	5.02E+05		2.63E+05	14	2	13	12	9	13	Carbamid
721	4004	sp|P10412|H14_HUMAN	226.11	4.10E+06	5.02E+07	5.35E+08	3.34E+07	14	4	9	15	11	11	Acetylatic
1128	5618	sp|O75531|BAF_HUMAN	225.79	9.27E+06	1.04E+08	3.73E+07	1.50E+08	10	10	3	12	5	7	Carbamid
859	1718	sp|O60282|KIF5C_HUMAN	225.33		2.01E+06	6.83E+04		15	3	15	10	8	6	Carbamid
1100	1308	sp|Q9UJX2|CDC23_HUMAN	225.21	6.23E+06	4.39E+06	7.69E+06	1.67E+06	15	15	10	7	7	2	Carbamid
1212	3476	tr|C9JF46|C9JF46_HUMAN	225.18	1.85E+06	1.82E+06		4.97E+05	10	1	8	4	7	6	Carbamid
827	1604	tr|A0A286YF11|A0A286YF11_HUMAN	225.03	1.39E+07	1.28E+07	2.34E+07	1.60E+07	16	16	8	10	13	10	Carbamid
827	1606	sp|Q9BPX6|MICU1_HUMAN	225.03	1.39E+07	1.28E+07	2.34E+07	1.60E+07	16	16	8	10	13	10	Carbamid
827	1607	tr|F6XEV2|F6XEV2_HUMAN	225.03	1.39E+07	1.28E+07	2.34E+07	1.60E+07	16	16	8	10	13	10	Carbamid
827	1611	tr|A0A0U1RRK1|A0A0U1RRK1_HUMAN	225.03	1.39E+07	1.28E+07	2.34E+07	1.60E+07	16	16	8	10	13	10	Carbamid
1028	1476	sp|Q9BT17|MTG1_HUMAN	224.94	2.86E+06	8.65E+06	1.25E+07	1.00E+07	17	17	4	6	9	9	Carbamid

1068	1207	sp|Q99615|DNJC7_HUMAN	224.69	7.98E+06	7.57E+06	2.21E+06	9.82E+05	19	19	8	12	2	1	Carbamid
847	1412	sp|Q9H9J2|RM44_HUMAN	224.69	4.87E+06	8.27E+06	2.16E+07	1.05E+07	15	14	5	8	13	10	Carbamid
1204	6710	sp|Q96K17|BT3L4_HUMAN	224.58	3.25E+07	4.16E+07	1.25E+07	1.65E+07	8	8	5	7	3	5	Deamidat
1227	7510	sp|Q8IUE6|H2A2B_HUMAN	224.48			4.92E+06		7	1	4	5	7	8	Deamidat
1189	1932	sp|P62879|GBB2_HUMAN	224.36		1.70E+06	1.23E+06	9.21E+05	11	5	5	8	5	4	Carbamid
779	3357	sp|P25398|RS12_HUMAN	224.25	8.57E+07	1.00E+08	2.42E+08	2.24E+08	10	10	8	9	13	10	Carbamid
926	2896	sp|P62937|PPIA_HUMAN	224.24	4.59E+07	3.27E+07	3.28E+07	3.31E+07	11	10	9	7	9	9	Carbamid
1200	1169	sp|P42696|RBM34_HUMAN	224.2	1.38E+05	1.59E+05	3.59E+07	2.02E+07	17	17	2	1	14	8	Carbamid
994	4171	sp|Q9NYL4|FKB11_HUMAN	224.15	8.07E+07	7.18E+07	7.77E+07	7.54E+07	10	10	7	7	8	7	Carbamid
567	1147	sp|Q96HS1|PGAM5_HUMAN	224.06	1.03E+07	2.52E+07	4.12E+07	6.33E+07	21	21	7	10	14	18	Carbamid
925	2260	sp|P16989|YBOX3_HUMAN	223.97	7.58E+05	3.18E+06	1.24E+07	7.43E+06	11	7	6	9	8	10	Deamidat
1124	2487	sp|Q9UI09|NDUAC_HUMAN	223.84	1.26E+07	1.57E+07	5.64E+06	6.50E+06	12	12	5	7	3	4	Acetylatio
1247	2378	sp|Q14993|COJA1_HUMAN	223.39	1.70E+07	7.53E+06	2.36E+07	9.02E+06	13	13	9	7	7	4	Carbamid
945	1710	sp|Q9NXS2|QPCTL_HUMAN	223.37	1.09E+07	1.90E+07	2.96E+07	5.26E+07	14	14	6	9	12	12	Carbamid
1062	1974	sp|Q9H8W4|PKHF2_HUMAN	223.34	1.80E+07	1.53E+07	8.97E+06	1.12E+07	10	10	9	8	5	7	Carbamid
355	2614	sp|P62277|RS13_HUMAN	223.31	4.32E+08	4.92E+08	1.68E+09	1.44E+09	19	19	16	18	20	25	
1137	1242	sp|P02533|K1C14_HUMAN	223.25	3.88E+06				18	2	10	5	5	1	Oxidatio
1266	1023	sp|Q9NP61|ARFG3_HUMAN	223.09	5.01E+06			1.13E+06	17	17	6	0	0	2	Carbamid
948	1517	sp|Q92769|HDAC2_HUMAN	222.99	1.50E+06	0	4.01E+06	2.09E+06	14	7	3	5	7	7	Carbamid
939	2452	sp|Q9BRP8|PYM1_HUMAN	222.87	3.29E+07	2.80E+07	3.70E+07	3.88E+07	11	11	13	8	5	7	
1501	1692	tr|H3BLV9|H3BLV9_HUMAN	222.68			1.91E+07	6.73E+06	11	9	0	0	11	5	Carbamid
1501	1695	sp|Q96SB4|SRPK1_HUMAN	222.68			1.91E+07	6.73E+06	11	9	0	0	11	5	Carbamid
834	1997	sp|Q9P0L0|VAPA_HUMAN	222.68	2.54E+07	2.83E+07	1.58E+07	1.68E+07	15	13	9	11	9	6	Carbamid
755	919	sp|P41250|GARS_HUMAN	222.56	1.21E+07	1.09E+07	9.79E+06	5.58E+06	21	20	16	14	12	5	Carbamid
840	2344	sp|P18085|ARF4_HUMAN	222.16	1.01E+07	1.08E+07	8.68E+06	1.24E+07	11	7	9	9	9	5	Carbamid
365	3849	sp|P62888|RL30_HUMAN	222.12	1.31E+08	1.85E+08	1.23E+09	1.46E+09	18	18	11	12	24	29	Carbamid
978	1068	sp|Q7L7X3|TAOK1_HUMAN	222.08		1.22E+06	1.01E+06	3.84E+06	24	19	2	4	2	6	Carbamid
888	1605	sp|P27348|1433T_HUMAN	221.91	4.91E+06	1.35E+06	2.75E+06	1.01E+06	13	7	10	7	8	8	Carbamid
686	1067	sp|O95453|PARN_HUMAN	221.67	1.19E+07	2.33E+07	5.75E+06	7.71E+06	23	23	14	16	4	8	Carbamid
1055	1218	sp|Q9BVJ6|UT14A_HUMAN	221.46	5.66E+07	5.88E+07	4.88E+07	4.31E+07	19	19	12	14	8	7	
866	1005	sp|Q8N6R0|EFNMT_HUMAN	221.28	9.58E+06	5.56E+06	1.10E+07	4.58E+06	18	18	11	9	8	8	Carbamid
1270	2476	sp|Q9Y2C4|EXOG_HUMAN	221.18	9.25E+06	9.60E+06	1.22E+07	1.06E+07	9	9	7	4	5	5	Carbamid
1228	1672	sp|O96019|ACL6A_HUMAN	221.05	4.89E+06	6.52E+06	1.08E+07	6.51E+06	12	11	4	8	7	3	Carbamid
1298	1852	sp|Q13610|PWP1_HUMAN	220.9		6.03E+05	3.40E+07	2.14E+07	13	13	0	1	11	9	Carbamid
1192	3322	sp|P59998|ARPC4_HUMAN	220.9	4.99E+06	2.00E+07	1.19E+07	3.87E+07	9	9	5	5	5	7	Carbamid
1192	3323	tr|F8WCF6|F8WCF6_HUMAN	220.9	4.99E+06	2.00E+07	1.19E+07	3.87E+07	9	9	5	5	5	7	Carbamid
1093	1585	sp|Q9HDC5|JPH1_HUMAN	220.51	3.97E+07	2.92E+07	9.48E+07	8.85E+07	20	20	17	13	8	7	Carbamid
915	3311	sp|P62753|RS6_HUMAN	220.47	1.27E+08	8.58E+07	3.53E+08	2.84E+08	9	9	13	8	13	9	Carbamid
1037	1929	sp|Q86U38|NOP9_HUMAN	220.32	4.02E+06	3.51E+06	1.17E+07	9.22E+06	11	10	6	7	9	9	Carbamid
851	4713	sp|P18859|ATP5J_HUMAN	220.22	1.16E+08	1.31E+08	9.16E+07	1.30E+08	9	9	8	7	7	11	Oxidatio
670	3349	tr|A0A0A6YYL6|A0A0A6YYL6_HUMAN	219.89	5.60E+07	6.80E+07	5.03E+08	3.82E+08	14	14	10	7	23	13	Carbamid
670	3470	tr|A0A087WXM6|A0A087WXM6_HUMAN	219.89	5.60E+07	6.80E+07	5.03E+08	3.82E+08	14	14	10	7	23	13	Carbamid
670	3471	tr|J3QQT2|J3QQT2_HUMAN	219.89	5.60E+07	6.80E+07	5.03E+08	3.82E+08	14	14	10	7	23	13	Carbamid
670	3472	tr|J3KRX5|J3KRX5_HUMAN	219.89	5.60E+07	6.80E+07	5.03E+08	3.82E+08	14	14	10	7	23	13	Carbamid
670	3473	sp|P18621|RL17_HUMAN	219.89	5.60E+07	6.80E+07	5.03E+08	3.82E+08	14	14	10	7	23	13	Carbamid
620	6079	sp|P62314|SMD1_HUMAN	219.79	3.73E+08	5.28E+08	6.18E+08	4.91E+08	8	8	11	15	11	10	Oxidatio
1049	1837	tr|A0A087WYF7|A0A087WYF7_HUMAN	219.31	1.71E+07	1.27E+07	1.55E+07	1.46E+07	14	14	7	6	7	7	Carbamid
1049	1838	sp|Q6UXV4|MIC27_HUMAN	219.31	1.71E+07	1.27E+07	1.55E+07	1.46E+07	14	14	7	6	7	7	Carbamid
718	1404	sp|Q9HBI1|PARVB_HUMAN	219.26	4.85E+07	3.35E+07	4.43E+07	2.52E+07	15	15	14	13	13	9	
1180	1159	sp|Q9Y2D4|EXC6B_HUMAN	219.18	4.09E+06	2.91E+06	1.64E+06	6.28E+05	16	14	7	5	4	1	Carbamid
1180	1160	tr|A0A0U1RRB6|A0A0U1RRB6_HUMAN	219.18	4.09E+06	2.91E+06	1.64E+06	6.28E+05	16	14	7	5	4	1	Carbamid
1299	2356	tr|G3V150|G3V150_HUMAN	219.18	3.15E+06	4.34E+06	7.74E+06	6.21E+07	10	10	3	3	4	6	
1299	2358	sp|O94766|B3GA3_HUMAN	219.18	3.15E+06	4.34E+06	7.74E+06	6.21E+07	10	10	3	3	4	6	
727	3840	sp|P16403|H12_HUMAN	219.15	1.48E+07	2.55E+07	2.74E+08	1.14E+08	13	3	9	14	8	12	Acetylatio
1116	1455	sp|Q9NR12|PDLI7_HUMAN	219.03	9.24E+06	9.22E+06	5.41E+06	5.87E+06	13	13	6	5	5	6	Carbamid
1077	2897	sp|P04792|HSPB1_HUMAN	218.97	9.07E+06	5.53E+06	8.56E+06	6.09E+06	9	9	9	5	6	5	
905	2703	sp|P43307|SSRA_HUMAN	218.74	6.43E+07	7.03E+07	1.38E+08	1.12E+08	8	8	7	6	7	9	Oxidatio
905	2704	tr|A0A3B3IRT8|A0A3B3IRT8_HUMAN	218.74	6.43E+07	7.03E+07	1.38E+08	1.12E+08	8	8	7	6	7	9	Oxidatio
496	1920	sp|Q9NZ01|TECR_HUMAN	218.53	1.67E+08	1.90E+08	2.95E+08	2.78E+08	16	16	14	15	14	16	Carbamid
839	2662	sp|Q9Y281|COF2_HUMAN	218.5		5.02E+06		9.50E+06	12	6	10	12	4	12	Carbamid
693	1487	sp|P14649|MYL6B_HUMAN	218.41	7.66E+05	1.82E+07	4.44E+06	2.18E+07	15	12	3	16	9	12	Carbamid
897	2933	sp|Q13151|ROA0_HUMAN	218.33	1.17E+07	1.51E+07	4.57E+07	3.01E+07	9	7	8	10	7	9	Carbamid
552	6654	sp|P62841|RS15_HUMAN	218.29	6.56E+07	9.93E+07	2.03E+08	2.42E+08	11	11	13	8	15	20	Oxidatio
552	6812	tr|K7ELC2|K7ELC2_HUMAN	218.29	6.56E+07	9.93E+07	2.03E+08	2.42E+08	11	11	13	8	15	20	Oxidatio
552	7087	tr|K7EM56|K7EM56_HUMAN	218.29	6.56E+07	9.93E+07	2.03E+08	2.42E+08	11	11	13	8	15	20	Oxidatio
552	7138	tr|A0A0B4J2B4|A0A0B4J2B4_HUMAN	218.29	6.56E+07	9.93E+07	2.03E+08	2.42E+08	11	11	13	8	15	20	Oxidatio
552	7139	tr|S4R417|S4R417_HUMAN	218.29	6.56E+07	9.93E+07	2.03E+08	2.42E+08	11	11	13	8	15	20	Oxidatio
862	970	sp|P49368|TCPG_HUMAN	218.28	1.26E+07	8.65E+06	1.15E+07	7.74E+06	18	18	10	8	8	9	Carbamid
1084	2123	sp|P11177|ODPB_HUMAN	218.28	8.97E+06	1.04E+07	9.28E+06	5.42E+06	10	10	7	8	7	4	Carbamid
610	4007	sp|P61353|RL27_HUMAN	217.89	6.32E+07	1.37E+08	1.24E+09	1.30E+09	14	14	8	9	17	16	Oxidatio
918	2922	sp|Q04837|SSBP_HUMAN	217.88	5.79E+07	3.84E+07	3.89E+07	3.06E+07	11	11	11	11	9	5	
868	1114	sp|Q13895|BYST_HUMAN	217.59	1.15E+06	8.25E+06	1.20E+07	1.90E+07	16	16	3	9	7	8	Carbamid
1047	1591	sp|Q13838|DX39B_HUMAN	217.58		5.36E+05	1.76E+06		14	5	7	7	10	6	Carbamid
770	4214	sp|P84103|SRSF3_HUMAN	217.56	1.70E+07	3.50E+07	1.06E+08	1.68E+08	12	8	4	7	10	14	Carbamid
799	1075	sp|Q96KP1|EXOC2_HUMAN	217.38	1.05E+07	7.03E+06	1.32E+07	8.17E+06	23	23	10	7	9	10	Carbamid
870	4075	sp|P62899|RL31_HUMAN	217.24	1.26E+08	1.76E+08	7.30E+08	6.10E+08	10	10	7	8	14	8	Oxidatio
809	1437	sp|Q13601|KRR1_HUMAN	217.19	1.81E+07	1.54E+07	6.31E+07	5.18E+07	17	17	6	7	13	17	Carbamid
1274	4514	sp|Q99470|SDF2_HUMAN	217.08	1.43E+07	1.36E+07	8.18E+06	9.34E+06	8	8	6	6	2	4	Carbamid
1275	1656	sp|P18754|RCC1_HUMAN	217.08	1.06E+07	2.85E+06	5.18E+06	4.66E+06	13	13	8	3	4	4	Carbamid
934	1217	sp|Q96KC8|DNJC1_HUMAN	216.89	1.59E+07	1.56E+07	1.67E+07	1.54E+07	17	17	6	10	6	7	Carbamid
1027	2760	sp|P84077|ARF1_HUMAN	216.86	1.32E+07	1.36E+07	1.07E+07	8.26E+06	9	4	8	7	8	3	Deamidat
1027	2806	sp|P61204|ARF3_HUMAN	216.86	1.32E+07	1.36E+07	1.07E+07	8.26E+06	9	4	8	7	8	3	Deamidat
1625	3910	tr|H0YEF7|H0YEF7_HUMAN	216.7	2.05E+06	7.66E+05	1.23E+06		7	1	5	3	3	3	Deamidat
1625	3994	tr|H0YEH1|H0YEH1_HUMAN	216.7	2.05E+06	7.66E+05	1.23E+06		7	1	5	3	3	3	Deamidat
848	1918	sp|P31946|1433B_HUMAN	216.68	2.46E+06	4.65E+05	1.72E+06	2.71E+06	13	5	9	6	10	9	Oxidatio
1053	1062	sp|Q9UBB9|TFP11_HUMAN	216.54	1.15E+07	1.70E+07	9.68E+06	4.37E+07	23	23	11	15	8	10	Acetylatio
464	4545	sp|P53999|TCP4_HUMAN	216.54	1.07E+09	1.12E+09	7.29E+08	9.10E+08	12	12	14	21	12	15	Oxidatio
1171	4309	sp|Q9NY12|GAR1_HUMAN	216.5	3.57E+07	5.31E+07	1.13E+08	9.57E+07	8	8	4	6	6	7	Carbamid
487	2891	sp|P62244|RS15A_HUMAN	216.45	3.61E+07	6.00E+07	1.70E+08	1.89E+08	12	6	13	17	18	18	Carbamid
753	890	sp|A3KN83|SBNO1_HUMAN	216.34	1.00E+07	1.45E+07	3.94E+06	7.94E+06	22	22	10	16	6	15	Carbamid
1132	1623	sp|Q99613|EIF3C_HUMAN	216.25	5.23E+06	4.47E+06	6.59E+06	2.55E+06	19	19	7	7	6	3	Carbamid
1132	1626	sp|B5ME19|EIFCL_HUMAN	216.25	5.23E+06	4.47E+06	6.59E+06	2.55E+06	18	18	7	7	6	3	Carbamid
972	2867	sp|P0DN76|U2AF5_HUMAN	215.96	1.15E+07	4.63E+07	2.86E+07	4.66E+07	11	11	7	10	7	12	Carbamid
972	2868	sp|Q01081|U2AF1_HUMAN	215.96	1.15E+07	4.63E+07	2.86E+07	4.66E+07	11	11	7	10	7	12	Carbamid
842	1465	sp|Q96JB5|CK5P3_HUMAN	215.88	2.13E+07	2.67E+07	1.96E+07	1.37E+07	12	11	12	12	8	7	Acetylatio
1069	2032	tr|F5H6T1|F5H6T1_HUMAN	215.57					13	1	5	10	4	7	Carbamid
1261	1978	tr|R4GNH3|R4GNH3_HUMAN	215.53	2.82E+06	1.94E+06	2.99E+06	3.29E+06	13	12	6	4	5	5	Acetylatio
1261	1980	sp|P17980|PRS6A_HUMAN	215.53	2.82E+06	1.94E+06	2.99E+06	3.29E+06	13	12	6	4	5	5	Acetylatio
1261	2003	tr|E9PM69|E9PM69_HUMAN	215.53	2.82E+06	1.94E+06	2.99E+06	3.29E+06	13	12	6	4	5	5	Acetylatio
1579	1370	sp|P29279|CTGF_HUMAN	215.46	2.21E+07	1.48E+06	1.13E+07		14	14	14	2	5	0	Carbamid
780	2031	sp|Q00688|FKBP3_HUMAN	215.45	5.55E+07	2.12E+07	1.09E+07	1.51E+07	12	12	13	10	8	5	Carbamid
937	1444	sp|P10155|RO60_HUMAN	215.45	9.68E+06	1.18E+07	1.56E+07	1.29E+07	12	12	9	7	7	13	Carbamid
946	2534	sp|Q8NCA5|FA98A_HUMAN	215.3	2.31E+07	1.67E+07	1.84E+07	1.81E+07	10	8	9	8	10	6	Carbamid
1240	7360	sp|P07919|QCR6_HUMAN	215.18	8.20E+07	7.27E+07	1.17E+08	6.74E+07	7	7	6	4	5	5	Carbamid
1211	2746	sp|Q96P16|RPR1A_HUMAN	215.04	5.94E+06	3.67E+06	5.28E+05	2.75E+06	10	6	9	6	3	6	Deamidat
1170	2202	sp|Q12824|SNF5_HUMAN	215.03	6.77E+06	5.73E+06	1.25E+07	4.50E+06	10	10	4	5	7	5	Carbamid
852	2224	sp|Q9NVM6|DJC17_HUMAN	215	4.87E+07	5.45E+07	3.44E+07	4.31E+07	11	11	7	7	9	9	Carbamid
999	2738	sp|O96000|NDUBA_HUMAN	214.93	2.27E+07	1.99E+07	2.77E+07	9.98E+07	13	13	10	7	8	8	Carbamid
1507	1186	sp|Q9Y5T5|UBP16_HUMAN	214.88	3.99E+06	1.41E+07	3.39E+06	1.04E+06	14	14	8	12	2	1	Carbamid
1041	1847	sp|P12004|PCNA_HUMAN	214.87	1.42E+07	1.39E+07	1.57E+07	1.41E+07	13	13	10	6	6	10	Carbamid
1085	2172	sp|P19388|RPAB1_HUMAN	214.85	1.77E+07	2.86E+07	5.02E+07	3.79E+07	12	12	6	9	7	7	Carbamid
357	1856	sp|P62280|RS11_HUMAN	214.72	1.68E+08	2.91E+08	8.28E+08	6.89E+08	21	21	15	19	20	23	Carbamid
1193	2583	sp|Q9H944|MED20_HUMAN	214.69	2.18E+07	2.78E+07	2.80E+07	2.48E+07	10	10	9	8	6	6	Carbamid
790	1563	sp|P49770|EI2BB_HUMAN	214.67	1.29E+07	1.28E+07	1.91E+07	1.14E+07	14	14	11	10	7	6	Carbamid
968	2663	sp|Q07666|KHDR1_HUMAN	214.61	3.98E+07	4.28E+07	4.27E+07	4.75E+07	9	8	11	8	6	9	Oxidatio
1548	3235	sp|P52943|CRIP2_HUMAN	214.52	1.74E+07	1.74E+07	1.53E+07	8.52E+06	8	8	6	7	4	2	Carbamid

1548	3656	tr|H0YFA4|H0YFA4_HUMAN	214.52	1.74E+07	1.36E+07	1.53E+07	8.52E+06	7	7	6	6	4	2	Carbamid
629	1015	sp|Q9BXS6|NUSAP_HUMAN	214.19	5.95E+07	5.72E+07	3.46E+07	5.11E+07	20	20	18	12	12	17	Carbamid
675	3438	sp|P41223|BUD31_HUMAN	213.81	6.99E+07	6.08E+07	5.78E+07	5.52E+07	10	10	11	11	15	11	Carbamid
1054	1083	sp|P54577|SYYC_HUMAN	213.61	3.34E+07	3.21E+06	3.45E+05	9.22E+05	24	24	24	5	1	3	Carbamid
964	1677	sp|O60583|CCNT2_HUMAN	213.56	3.16E+07	3.48E+07	2.31E+07	1.93E+07	13	12	11	14	10	8	Carbamid
777	1621	sp|P62258|1433E_HUMAN	213.44	2.67E+07	2.98E+07	1.96E+07	1.28E+07	13	10	10	14	9	9	Carbamid
1362	3021	sp|Q16543|CDC37_HUMAN	213.26	7.55E+06	3.37E+06	6.42E+06	2.77E+06	7	7	7	3	4	2	Carbamid
1136	1273	sp|Q99460|PSMD1_HUMAN	213.25	4.55E+06	4.63E+06	5.10E+06	4.73E+06	19	19	8	6	4	7	Carbamid
1136	1274	tr|A0A087WW66|A0A087WW66_HUMAN	213.25	4.55E+06	4.63E+06	5.10E+06	4.73E+06	19	19	8	6	4	7	Carbamid
966	2328	sp|O95816|BAG2_HUMAN	213.19	1.36E+07	1.76E+07	2.54E+07	3.18E+07	13	13	9	6	9	8	Carbamid
904	2434	sp|Q9Y3D9|RT23_HUMAN	213.18	3.51E+07	3.65E+07	8.14E+07	8.07E+07	11	11	8	6	9	9	
1119	1270	sp|Q00013|EM55_HUMAN	213.14	1.36E+07	1.09E+07	6.87E+06	6.38E+06	14	14	7	7	4	6	Carbamid
933	962	sp|P49790|NU153_HUMAN	213.08	2.45E+07	1.71E+07	2.23E+07	1.12E+07	22	22	16	10	13	8	Carbamid
1366	2386	sp|P27105|STOM_HUMAN	213.05	1.98E+06	5.95E+06	5.75E+06	8.12E+06	10	10	2	4	4	4	
1321	1054	sp|P46939|UTRO_HUMAN	213.05	1.25E+07	3.02E+06	3.59E+06	2.80E+06	29	28	9	4	9	5	Carbamid
864	1376	sp|Q14232|EI2BA_HUMAN	212.95	9.06E+06	9.12E+06	1.60E+07	1.81E+07	16	16	9	7	9	8	Carbamid
841	4049	sp|Q9NQ50|RM40_HUMAN	212.92	8.16E+07	1.38E+08	8.53E+07	1.25E+08	9	9	9	13	11	8	
1177	2451	sp|Q8TBP6|S2540_HUMAN	212.77	6.26E+06	4.87E+06	7.14E+06	3.58E+06	10	10	5	9	6	4	Carbamid
806	1609	sp|P08621|RU17_HUMAN	212.41	5.73E+07	9.30E+07	1.46E+06	5.40E+06	17	17	15	14	3	3	Carbamid
1376	2920	sp|P21964|COMT_HUMAN	212.38	8.20E+06	4.76E+06	2.98E+06	4.19E+05	11	11	8	5	3	1	Carbamid
682	1797	sp|P60981|DEST_HUMAN	212.36	9.26E+07	3.86E+07	2.64E+07	2.50E+07	14	13	16	14	11	6	Carbamid
1038	1684	sp|Q8IUF8|RIOX2_HUMAN	212.23	5.91E+06	6.00E+06	3.94E+07	1.86E+07	11	11	6	4	12	7	
892	1365	sp|Q9UEW8|STK39_HUMAN	212.23	9.54E+06	3.18E+06	3.02E+06	3.30E+06	15	11	16	10	5	5	Carbamid
1561	6669	sp|Q6P161|RM54_HUMAN	212.21	3.60E+06	5.69E+06	2.73E+07	2.20E+07	4	4	2	2	4	4	Carbamid
989	1234	sp|O75063|XYLK_HUMAN	212.18	1.39E+07	8.63E+06	1.05E+07	7.49E+06	15	15	8	5	6	8	Carbamid
527	3220	sp|P16401|H15_HUMAN	212.01	2.48E+08	3.82E+08	1.80E+09	2.43E+09	13	12	10	12	19	20	Acetylatio
947	1223	sp|O15144|ARPC2_HUMAN	211.93	1.79E+06	2.60E+07	9.40E+06	2.19E+07	16	16	3	11	5	7	Carbamid
1134	1099	sp|P54098|DPOG1_HUMAN	211.88	4.16E+06	2.23E+06	1.76E+06	1.17E+06	23	23	14	5	3	2	Carbamid
811	2740	sp|Q9H2K0|IF3M_HUMAN	211.67	6.99E+07	8.70E+07	5.57E+07	6.89E+07	11	11	10	11	10	8	Carbamid
954	2075	tr|A0A3B3ITL8|A0A3B3ITL8_HUMAN	211.54	3.74E+05	5.95E+05			14	2	10	11	6	6	Carbamid
951	2130	sp|P51153|RAB13_HUMAN	211.53	9.17E+06	1.01E+07	8.92E+06	7.77E+06	11	8	8	9	8	8	Deamidat
723	5722	tr|A0A2R8Y849|A0A2R8Y849_HUMAN	211.52	8.69E+07	1.67E+08	3.64E+08	3.36E+08	9	9	7	9	11	16	Acetylatio
723	5723	tr|E7ETK0|E7ETK0_HUMAN	211.52	8.69E+07	1.67E+08	3.64E+08	3.36E+08	9	9	7	9	11	16	Acetylatio
723	5724	tr|A0A087WUS0|A0A087WUS0_HUMAN	211.52	8.69E+07	1.67E+08	3.64E+08	3.36E+08	9	9	7	9	11	16	Acetylatio
723	5725	sp|P62847|RS24_HUMAN	211.52	8.69E+07	1.67E+08	3.64E+08	3.36E+08	9	9	7	9	11	16	Acetylatio
723	5726	tr|A0A2R8YD14|A0A2R8YD14_HUMAN	211.52	8.69E+07	1.67E+08	3.64E+08	3.36E+08	9	9	7	9	11	16	Acetylatio
1092	1214	sp|P55265|DSRAD_HUMAN	211.25	9.83E+06	4.02E+06	6.65E+06	1.07E+07	24	24	13	4	8	12	Carbamid
1011	1031	sp|Q09161|NCBP1_HUMAN	211.15	2.16E+07	1.91E+07	5.41E+06	2.00E+07	16	16	3	6	4	7	Carbamid
970	1518	sp|P49757|NUMB_HUMAN	211.1	1.27E+07	9.91E+06	1.81E+07	2.31E+07	16	16	6	8	10	13	Carbamid
1160	1017	sp|Q8NI27|THOC2_HUMAN	210.99	3.31E+06	5.70E+06	6.06E+06	5.34E+06	20	20	6	10	6	8	Carbamid
872	1395	tr|A0A3B3IU45|A0A3B3IU45_HUMAN	210.91	1.02E+07	9.00E+06	1.46E+07	4.01E+06	22	22	15	10	12	3	Carbamid
872	1398	sp|P33991|MCM4_HUMAN	210.91	1.02E+07	9.00E+06	1.46E+07	4.01E+06	22	22	15	10	12	3	Carbamid
982	1459	sp|P51798|CLCN7_HUMAN	210.86	5.84E+06	3.29E+06	1.68E+07	1.66E+07	14	14	6	3	11	10	Carbamid
788	1277	sp|Q92738|US6NL_HUMAN	210.71	1.07E+07	2.08E+07	1.02E+07	9.81E+06	17	17	12	14	7	11	Carbamid
758	2249	sp|P61981|1433G_HUMAN	210.63	1.40E+07	7.52E+06	6.22E+06	7.78E+06	9	5	12	9	9	9	Oxidatio
764	1247	sp|Q4G0J3|LARP7_HUMAN	210.62	2.37E+07	3.39E+07	2.05E+07	2.92E+07	20	20	14	17	8	10	Carbamid
642	2565	sp|Q02543|RL18A_HUMAN	210.59	4.02E+07	1.08E+08	7.82E+08	8.37E+08	12	12	7	8	13	19	Carbamid
1036	1375	sp|Q9NUL7|DDX28_HUMAN	210.49	5.67E+06	5.62E+06	5.24E+06	3.99E+06	16	16	5	7	8	4	Carbamid
1174	4659	sp|P52815|RM12_HUMAN	210.41	3.08E+06	3.76E+06	7.26E+06	9.11E+06	8	1	5	5	7	5	Oxidatio
957	1702	sp|Q9H0S4|DDX47_HUMAN	210.4	5.65E+06	7.60E+06	2.42E+07	1.48E+07	12	11	6	7	13	8	Carbamid
1386	1462	sp|Q01970|PLCB3_HUMAN	210.4	4.38E+06	2.78E+06	1.57E+06	7.37E+05	16	16	7	6	4	1	
1158	5027	tr|H7BYD0|H7BYD0_HUMAN	210.37	4.48E+07	3.19E+07	3.43E+07	3.25E+07	6	6	7	7	7	6	Carbamid
1158	5028	sp|Q16718|NDUA5_HUMAN	210.37	4.48E+07	3.19E+07	3.43E+07	3.25E+07	6	6	7	7	7	6	Carbamid
1154	2870	sp|P51148|RAB5C_HUMAN	210.05	4.49E+06	5.03E+06	2.73E+06	3.03E+06	9	5	9	12	3	4	
1044	2057	sp|O60762|DPM1_HUMAN	209.82	8.63E+06	1.60E+07	2.61E+07	1.61E+07	13	13	6	9	7	5	Deamidat
1044	2062	tr|Q5QPK2|Q5QPK2_HUMAN	209.82	8.63E+06	1.60E+07	2.61E+07	1.61E+07	13	13	6	9	7	5	Deamidat
1044	2065	tr|H0Y368|H0Y368_HUMAN	209.82	8.63E+06	1.60E+07	2.61E+07	1.61E+07	13	13	6	9	7	5	Deamidat
1202	1499	tr|J3KMX2|J3KMX2_HUMAN	209.73	2.16E+06	2.13E+06	1.93E+06	2.69E+06	14	13	4	2	2	4	Carbamid
1202	1504	tr|B9EGA3|B9EGA3_HUMAN	209.73	2.16E+06	2.13E+06	1.93E+06	2.69E+06	14	13	4	2	2	4	Carbamid
1202	1505	sp|Q92925|SMRD2_HUMAN	209.73	2.16E+06	2.13E+06	1.93E+06	2.69E+06	14	13	4	2	2	4	Carbamid
704	1109	sp|Q9BWM7|SFXN3_HUMAN	209.73	1.80E+07	2.09E+07	2.02E+07	2.24E+07	15	12	9	13	11	10	Carbamid
704	1110	tr|A0A0A0MS41|A0A0A0MS41_HUMAN	209.73	1.80E+07	2.09E+07	2.02E+07	2.24E+07	15	12	9	13	11	10	Carbamid
704	1111	tr|A0A1P0AYU5|A0A1P0AYU5_HUMAN	209.73	1.80E+07	2.09E+07	2.02E+07	2.24E+07	15	12	9	13	11	10	Carbamid
1089	4211	tr|E5RHG8|E5RHG8_HUMAN	209.48	3.90E+07	6.67E+07	6.83E+07	6.55E+07	6	6	7	6	8	6	Carbamid
1089	4213	sp|Q15369|ELOC_HUMAN	209.48	3.90E+07	6.67E+07	6.83E+07	6.55E+07	6	6	7	6	8	6	Carbamid
1393	1754	sp|Q00403|TF2B_HUMAN	209.47	1.13E+07	5.44E+06	2.12E+06	5.74E+06	12	12	10	6	2	4	Carbamid
1056	1715	sp|Q9H3P2|NELFA_HUMAN	209.32	1.10E+07	9.34E+06	5.77E+06	5.19E+06	11	11	8	6	4	5	Carbamid
1056	1716	tr|A0A0C4DFX9|A0A0C4DFX9_HUMAN	209.32	1.10E+07	9.34E+06	5.77E+06	5.19E+06	11	11	8	6	4	5	Carbamid
1252	1202	sp|Q658Y4|F91A1_HUMAN	209.3	2.30E+05	6.19E+05	6.34E+06	7.96E+06	14	14	1	2	7	7	Carbamid
1400	1560	sp|Q8WVM8|SCFD1_HUMAN	209.3	1.32E+06	1.50E+06	2.52E+06	1.38E+06	13	13	1	2	4	5	Carbamid
792	1874	tr|D6RCD0|D6RCD0_HUMAN	209.11	2.89E+07	2.16E+07	2.90E+07	3.58E+07	11	11	11	9	9	10	
792	1884	sp|Q8NBQ5|DHB11_HUMAN	209.11	2.89E+07	2.16E+07	2.90E+07	3.58E+07	11	11	11	9	9	10	
1083	1498	sp|P19525|E2AK2_HUMAN	208.97	9.36E+06	4.74E+06	2.18E+07	1.69E+07	13	13	7	4	9	9	Carbamid
1295	1209	sp|Q32MZ4|LRRF1_HUMAN	208.83	9.83E+05	1.37E+06	1.36E+06	1.16E+06	15	14	2	5	2	3	Carbamid
984	2377	sp|O75940|SPF30_HUMAN	208.44	4.38E+07	5.14E+07	3.74E+07	4.49E+07	12	12	14	8	8	8	Carbamid
134	2167	sp|P62269|RS18_HUMAN	208.43	3.68E+09	5.50E+09	5.70E+09	5.76E+09	26	26	34	43	33	38	Acetylatio
1352	1657	sp|P48634|PRC2A_HUMAN	208.27	1.90E+06	2.67E+06	1.29E+06	4.65E+06	18	16	6	4	2	7	
961	1281	sp|P49792|RBP2_HUMAN	208.24	1.44E+07	5.65E+06	6.16E+06	3.50E+06	25	24	17	8	7	7	Carbamid
1070	1534	sp|Q13547|HDAC1_HUMAN	208.07	6.76E+05	1.03E+06	5.86E+06	5.37E+06	14	7	2	6	7	7	Carbamid
1273	1619	sp|Q96EY7|PTCD3_HUMAN	207.88	5.44E+06	7.48E+05	1.59E+06	1.44E+07	13	13	2	2	2	9	Carbamid
1060	1746	sp|O95747|OXSR1_HUMAN	207.73	8.02E+06	9.87E+05	5.40E+06	1.38E+06	15	11	13	8	4	3	Carbamid
1042	2011	sp|Q15796|SMAD2_HUMAN	207.66	1.24E+07	2.14E+07	8.39E+06	3.73E+07	11	9	8	9	7	7	Carbamid
825	3485	sp|Q7RTV0|PHF5A_HUMAN	207.46	2.80E+08	3.94E+08	1.49E+08	3.07E+08	9	9	9	10	7	8	Carbamid
785	3956	sp|O00488|ZN593_HUMAN	207.45	7.17E+07	9.03E+07	7.58E+07	9.48E+07	8	8	11	13	10	13	
1255	4254	tr|I3L0E3|I3L0E3_HUMAN	207.37	1.34E+07	1.52E+07	5.62E+07	4.71E+07	7	7	5	4	5	6	Carbamid
1255	4556	sp|Q9Y2R5|RT17_HUMAN	207.37	1.34E+07	1.52E+07	5.62E+07	4.71E+07	7	7	5	4	5	6	Carbamid
1088	3491	sp|Q8N5N7|RM50_HUMAN	207.27	8.36E+06	4.20E+06	9.32E+06	1.14E+07	8	8	7	4	6	8	Carbamid
1207	2101	sp|P60953|CDC42_HUMAN	207.18	2.74E+07	2.24E+07	9.97E+06	1.02E+07	11	9	11	8	5	4	Carbamid
1117	4041	sp|Q96L21|RL10L_HUMAN	207.07				2.90E+06	10	1	5	6	7	8	Carbamid
1168	1265	sp|Q9Y5X2|SNX8_HUMAN	207.06	5.25E+06	5.37E+06	1.13E+07	1.08E+07	15	15	6	4	7	9	Carbamid
672	1617	sp|P22626|ROA2_HUMAN	206.94	2.39E+07	2.56E+07	3.58E+06	4.31E+06	13	9	15	18	7	5	Carbamid
1035	1561	sp|Q9GZS1|RPA49_HUMAN	206.93	6.47E+04	1.87E+06	1.12E+07	1.27E+07	12	12	1	3	9	12	Carbamid
1506	1861	sp|P04181|OAT_HUMAN	206.92	2.81E+06	2.67E+06			12	12	6	3	0	0	Carbamid
1243	1177	sp|Q9Y5B6|PAXB1_HUMAN	206.64	4.30E+06	6.49E+06	7.89E+06	7.18E+06	17	17	9	10	7	8	Carbamid
1226	2831	sp|Q96Q45|TM237_HUMAN	206.59	8.55E+06	5.72E+06		4.28E+06	11	11	7	6	0	3	Deamidat
1221	1040	sp|Q14008|CKAP5_HUMAN	206.51	1.44E+06	3.00E+05	2.48E+06	2.13E+06	27	27	1	2	5	6	Carbamid
1643	4080	sp|O14579|COPE_HUMAN	206.23		9.76E+05	7.42E+06	9.38E+06	7	7	0	3	4	5	Carbamid
1643	4081	tr|M0QXB4|M0QXB4_HUMAN	206.23		9.76E+05	7.42E+06	9.38E+06	7	7	0	3	4	5	Carbamid
901	3108	sp|P59780|AP3S2_HUMAN	206.18	1.85E+07	3.23E+07	3.23E+07	1.87E+07	8	8	8	10	8	9	Carbamid
901	3109	tr|H0YLI7|H0YLI7_HUMAN	206.18	1.85E+07	3.23E+07	3.23E+07	1.87E+07	8	8	8	10	8	9	Carbamid
1024	2902	sp|O43324|MCA3_HUMAN	206.16	1.89E+07	1.67E+07	1.25E+07	2.20E+07	10	10	8	8	4	4	Acetylatio
1339	2632	sp|Q5RKV6|EXOS6_HUMAN	206.02	3.21E+06	6.32E+06	2.65E+07	2.69E+07	10	10	4	6	6	6	Carbamid
1337	2931	sp|Q8N4T8|CBR4_HUMAN	205.96	8.36E+06	1.56E+07	9.90E+06	7.08E+06	8	8	4	8	4	3	Carbamid
1394	1152	tr|A0A2R8Y804|A0A2R8Y804_HUMAN	205.95	5.43E+05	9.51E+05	6.58E+05	4.71E+05	18	15	1	2	2	2	Carbamid
1394	1153	tr|A0A2R8Y543|A0A2R8Y543_HUMAN	205.95	5.43E+05	9.51E+05	6.58E+05	4.71E+05	18	15	1	2	2	2	Carbamid
1394	1155	tr|B4DGU4|B4DGU4_HUMAN	205.95	5.43E+05	9.51E+05	6.58E+05	4.71E+05	18	15	1	2	2	2	Carbamid
1394	1161	tr|A0A2R8Y5Z1|A0A2R8Y5Z1_HUMAN	205.95	5.43E+05	9.51E+05	6.58E+05	4.71E+05	18	15	1	2	2	2	Carbamid
1394	1162	tr|A0A2R8YCH5|A0A2R8YCH5_HUMAN	205.95	5.43E+05	9.51E+05	6.58E+05	4.71E+05	18	15	1	2	2	2	Carbamid
1394	1163	sp|P35222|CTNB1_HUMAN	205.95	5.43E+05	9.51E+05	6.58E+05	4.71E+05	18	15	1	2	2	2	Carbamid
1394	1164	tr|A0A2R8Y5A3|A0A2R8Y5A3_HUMAN	205.95	5.43E+05	9.51E+05	6.58E+05	4.71E+05	18	15	1	2	2	2	Carbamid
907	1260	sp|Q10570|CPSF1_HUMAN	205.9	4.18E+05	5.54E+06	2.03E+06	6.73E+06	28	28	3	13	3	9	Carbamid
1032	2026	sp|P78356|PI42B_HUMAN	205.81	5.03E+05	2.36E+06			12	4	6	12	6	6	Carbamid
1000	1086	sp|Q9NVC6|MED17_HUMAN	205.45	7.00E+06	1.67E+07	1.15E+07	1.86E+07	17	17	9	12	7	12	Carbamid
1000	1097	tr|A0A1W2PRB8|A0A1W2PRB8_HUMAN	205.45	7.00E+06	1.67E+07	1.15E+07	1.86E+07	17	17	9	12	7	12	Carbamid
1151	1782	sp|Q9NZJ7|MTCH1_HUMAN	205.34	2.26E+06	3.64E+06	8.25E+06	1.03E+07	12	12	1	5	5	5	Carbamid
665	4288	sp|O43920|NDUS5_HUMAN	205.28	4.36E+08	5.04E+08	3.57E+08	4.96E+08	13	13	13	11	10	11	Carbamid

941	1093	tr|A0A0D9SFK2|A0A0D9SFK2_HUMAN	205.26	9.94E+06	3.30E+07	1.01E+07	2.10E+07	29	29	5	9	5	7	Carbamid
941	1094	sp|Q92614|MY18A_HUMAN	205.26	9.94E+06	3.30E+07	1.01E+07	2.10E+07	29	29	5	9	5	7	Carbamid
416	5078	tr|S4R435|S4R435_HUMAN	205.13			6.20E+05		13	1	19	21	13	12	Acetylatic
1616	2135	tr|C9JRP1|C9JRP1_HUMAN	205.05	5.44E+05	5.82E+05	2.78E+05	5.06E+05	15	15	4	3	1	2	Carbamid
1616	2154	sp|Q9Y6M7|S4A7_HUMAN	205.05	5.44E+05	5.82E+05	2.78E+05	5.06E+05	15	15	4	3	1	2	Carbamid
1034	4696	sp|Q13442|HAP28_HUMAN	204.79	1.17E+08	6.74E+07	5.81E+07	5.99E+07	7	7	8	7	6	6	
1253	1879	sp|Q969S3|ZN622_HUMAN	204.78	8.13E+06	3.65E+06	5.30E+06	4.82E+06	11	11	4	4	3	5	Carbamid
1163	1703	sp|Q5JPH6|SYEM_HUMAN	204.78	5.10E+06	5.19E+06	2.58E+06	5.51E+06	12	12	7	6	5	5	Carbamid
1195	5763	tr|C9J7B1|C9J7B1_HUMAN	204.48		1.40E+06		6.68E+05	9	1	7	9	4	4	
1006	3069	sp|Q96G25|MED8_HUMAN	204.32	1.27E+07	2.08E+07	1.78E+07	2.28E+07	8	8	8	9	12	8	Carbamid
669	1349	sp|P26038|MOES_HUMAN	204.23	9.63E+06	9.65E+05	1.25E+07	2.37E+06	25	15	18	10	15	10	Carbamid
1106	1245	sp|Q8N3R3|TCAIM_HUMAN	203.77	8.76E+06	7.90E+06	9.76E+06	6.94E+06	14	14	6	6	8	4	Carbamid
640	2364	sp|P57088|TMM33_HUMAN	203.77	3.73E+07	5.45E+07	7.29E+07	5.23E+07	12	12	11	14	14	11	Carbamid
1417	1369	sp|Q6ZXV5|TMTC3_HUMAN	203.67	4.57E+06	9.43E+06	1.15E+06	2.45E+06	14	14	8	6	1	4	Carbamid
1425	1339	sp|P49748|ACADV_HUMAN	203.61	1.64E+06	1.86E+06	1.02E+06	1.87E+06	15	15	3	4	3	3	Carbamid
1071	1873	sp|P52294|IMA5_HUMAN	203.59	3.02E+06	1.69E+06	2.67E+06	2.37E+06	13	4	8	7	4	4	Carbamid
1196	1144	sp|P11586|C1TC_HUMAN	203.09	9.80E+06	1.50E+06	5.33E+06	9.04E+06	20	17	12	3	8	5	Carbamid
1196	1150	tr|F5H2F4|F5H2F4_HUMAN	203.09	9.80E+06	1.50E+06	5.33E+06	9.04E+06	20	17	12	3	8	5	Carbamid
1350	5019	sp|P09132|SRP19_HUMAN	202.81	1.73E+07	9.95E+06	1.42E+07	1.03E+07	6	6	5	4	5	5	Carbamid
1157	3130	sp|Q9NPD3|EXOS4_HUMAN	202.63	1.42E+07	8.67E+06	2.84E+07	4.72E+07	9	9	3	6	7	11	Carbamid
1375	4664	sp|Q9NWA0|MED9_HUMAN	202.6	2.03E+07	1.83E+07	2.15E+07	1.67E+07	6	6	7	4	7	5	Carbamid
1443	3133	sp|O00217|NDUS8_HUMAN	202.48	1.40E+07	7.82E+06	7.42E+06	1.10E+07	7	7	7	4	2	4	Carbamid
645	3779	sp|Q07020|RL18_HUMAN	202.45	6.36E+07	1.71E+08	1.46E+09	1.34E+09	13	13	7	9	16	20	Carbamid
645	3780	tr|J3QQ67|J3QQ67_HUMAN	202.45	6.36E+07	1.71E+08	1.46E+09	1.34E+09	13	13	7	9	16	20	Carbamid
645	3986	tr|G3V203|G3V203_HUMAN	202.45	6.36E+07	1.71E+08	1.46E+09	1.34E+09	13	13	7	9	16	20	Carbamid
798	1577	tr|E9PII3|E9PII3_HUMAN	202.35	9.62E+06	7.00E+06	7.49E+06	5.94E+06	19	11	13	12	12	9	Carbamid
798	1601	tr|E9PK52|E9PK52_HUMAN	202.35	9.62E+06	7.00E+06	7.49E+06	5.94E+06	19	11	13	12	12	9	Carbamid
798	1603	tr|A0A2R8Y5B3|A0A2R8Y5B3_HUMAN	202.35	9.62E+06	7.00E+06	7.49E+06	5.94E+06	19	11	13	12	12	9	Carbamid
798	1625	tr|E9PHY5|E9PHY5_HUMAN	202.35	9.62E+06	7.00E+06	7.49E+06	5.94E+06	19	11	13	12	12	9	Carbamid
798	1640	sp|O43491|E41L2_HUMAN	202.35	9.62E+06	7.00E+06	7.49E+06	5.94E+06	19	11	13	12	12	9	Carbamid
893	1819	sp|Q15717|ELAV1_HUMAN	202.34	2.81E+06	1.16E+07	9.64E+06	2.96E+07	12	12	5	7	8	12	Deamidat
1355	2595	sp|Q13523|PRP4B_HUMAN	202.29	9.52E+06	1.41E+07	1.28E+07	8.32E+06	10	10	5	9	8	7	Carbamid
1018	1393	sp|P49336|CDK8_HUMAN	202.27	6.09E+06	6.80E+06	5.01E+06	3.45E+06	18	12	8	10	7	7	Carbamid
1133	1332	sp|P40818|UBP8_HUMAN	202.09	1.06E+07	7.61E+06	5.68E+06	4.05E+06	19	19	10	10	6	4	Carbamid
1236	2795	sp|Q9BQA1|MEP50_HUMAN	202.05	1.33E+07	1.89E+07	9.91E+06	1.10E+07	8	8	6	8	6	5	Carbamid
530	1342	tr|Q5TCU3|Q5TCU3_HUMAN	201.78	2.15E+06	7.40E+06	3.67E+06	9.59E+06	15	2	11	14	14	14	Carbamid
858	4245	sp|P37108|SRP14_HUMAN	201.78	8.84E+07	8.98E+07	2.25E+08	1.63E+08	8	8	8	8	11	10	Oxidatior
1454	1645	sp|Q14980|NUMA1_HUMAN	201.76	4.45E+07	2.65E+07	5.30E+06	7.06E+06	18	17	10	9	4	5	Deamidat
1454	1646	tr|A0A087WY61|A0A087WY61_HUMAN	201.76	4.45E+07	2.65E+07	5.30E+06	7.06E+06	18	17	10	9	4	5	Deamidat
774	2243	sp|P51991|ROA3_HUMAN	201.65	8.44E+06	8.90E+06	1.15E+07	9.60E+06	13	9	12	12	10	9	Carbamid
836	2146	sp|Q14331|FRG1_HUMAN	201.59	2.30E+07	4.10E+07	2.80E+07	2.98E+07	12	12	23	14	6	4	Carbamid
956	4079	sp|Q9Y3B4|SF3B6_HUMAN	201.57	8.62E+07	1.63E+08	1.01E+08	1.36E+08	7	7	8	10	7	6	Carbamid
1502	1693	sp|P35606|COPB2_HUMAN	201.4	1.87E+06	2.59E+05	1.99E+06	2.38E+06	14	14	4	1	2	3	Carbamid
1399	2546	sp|Q9ULX3|NOB1_HUMAN	201.25	9.80E+05	1.81E+06	1.19E+06	1.83E+06	10	10	2	3	3	4	Carbamid
1716	5919	sp|Q9P0P8|CF203_HUMAN	201.18	1.31E+07	9.23E+06	6.56E+06	8.48E+06	6	6	3	5	1	4	
1716	5920	tr|A0A0D9SEI0|A0A0D9SEI0_HUMAN	201.18	1.31E+07	9.23E+06	6.56E+06	8.48E+06	6	6	3	5	1	4	
1287	3612	sp|Q9BTT4|MED10_HUMAN	201.16	2.09E+07	2.10E+07	2.11E+07	1.49E+07	8	8	7	6	6	5	Acetylatic
1021	1675	sp|Q9HAN9|NMNA1_HUMAN	201.13	4.16E+05	4.55E+06	1.32E+07	3.20E+07	11	11	3	6	7	10	Carbamid
1508	2371	sp|P00750|TPA_HUMAN	201.12	3.94E+06	5.10E+06	3.41E+06	2.76E+06	12	12	8	7	3	4	Carbamid
1281	2311	sp|A6NJ78|MET15_HUMAN	201.02	5.87E+06	5.83E+06	7.63E+06	2.62E+06	10	10	5	7	4	3	Carbamid
952	2133	tr|H7C4T5|H7C4T5_HUMAN	200.87	6.63E+05	1.06E+06		3.19E+05	11	1	16	12	6	7	Carbamid
1208	2720	sp|Q14318|FKBP8_HUMAN	200.84	7.14E+06	5.69E+06	8.24E+06	1.04E+07	9	9	8	7	6	7	Carbamid
1233	3031	sp|Q96E11|RRFM_HUMAN	200.76	3.31E+07	2.51E+07	5.26E+06	8.29E+06	8	8	7	8	4	4	Carbamid
1155	2914	sp|P63000|RAC1_HUMAN	200.67	1.79E+07	1.85E+07	1.26E+07	1.99E+07	9	7	8	6	4	7	Carbamid
1126	2392	sp|Q8WUY1|THEM6_HUMAN	200.38	5.64E+06	7.58E+06	2.11E+07	1.32E+07	12	12	7	6	8	6	Carbamid
1156	3506	sp|Q9Y3B2|EXOS1_HUMAN	200.37	9.44E+05	4.03E+06	1.55E+07	3.48E+07	9	9	2	3	6	10	Carbamid
1095	1833	sp|Q96RN5|MED15_HUMAN	200.33	4.64E+06	2.18E+07	7.86E+06	1.17E+07	13	13	5	11	5	6	Carbamid
1427	1440	sp|P13667|PDIA4_HUMAN	200.33	1.56E+07	2.11E+06		2.49E+06	15	14	16	4	0	4	Carbamid
744	1902	sp|P61006|RAB8A_HUMAN	200.27	1.25E+07	1.06E+07	1.05E+07	9.36E+06	14	9	13	15	9	9	Deamidat
1325	3126	sp|Q8TAE8|G45IP_HUMAN	200.17	1.54E+07	1.29E+07	9.05E+06	1.42E+07	8	8	7	6	2	4	Carbamid
666	4096	sp|P47813|IF1AX_HUMAN	199.98	6.66E+07	5.18E+07	3.54E+07	3.62E+07	10	2	11	17	11	9	Carbamid
439	2918	sp|P62249|RS16_HUMAN	199.88	4.32E+08	6.41E+08	1.04E+09	1.05E+09	15	15	16	15	16	16	Deamidat
1629	2030	sp|Q96I59|SYNM_HUMAN	199.71	1.11E+06	5.97E+05	6.70E+05	7.48E+05	10	10	3	1	1	1	Carbamid
1420	1596	sp|Q7L2J0|MEPCE_HUMAN	199.7	2.04E+06	1.14E+07	5.84E+06	2.06E+06	13	13	3	7	4	3	Carbamid
896	1323	sp|P19784|CSK22_HUMAN	199.67	3.04E+06	4.09E+06	2.44E+06	6.41E+05	17	14	10	12	5	6	
1016	3207	tr|A0A087WUK2|A0A087WUK2_HUMAN	199.39	1.13E+07	3.87E+06	1.29E+07	1.06E+07	8	6	8	4	8	8	Carbamid
1016	3211	sp|O14979|HNRDL_HUMAN	199.39	1.13E+07	3.87E+06	1.29E+07	1.06E+07	8	6	8	4	8	8	Carbamid
1043	1203	sp|Q86VP6|CAND1_HUMAN	199.3	5.38E+06	3.33E+06	1.37E+06	3.78E+06	19	19	8	7	3	6	Carbamid
1094	982	sp|P35221|CTNA1_HUMAN	199.28	3.23E+07	1.65E+06	2.68E+06	1.03E+06	22	16	16	8	4	4	Carbamid
1097	1647	sp|P55039|DRG2_HUMAN	199.24	7.89E+06	7.38E+06	1.17E+07	5.93E+06	13	13	8	9	7	6	Carbamid
1115	1216	sp|Q53EP0|FND3B_HUMAN	199.21	5.79E+06	4.60E+06	2.04E+07	5.69E+06	19	19	8	6	10	7	Carbamid
1336	1913	sp|Q99523|SORT_HUMAN	199.19	6.04E+06	3.19E+06	4.36E+06	7.02E+06	11	11	5	5	5	6	Carbamid
1424	2569	sp|P30048|PRDX3_HUMAN	199.14	7.30E+06	8.19E+06	1.93E+06	4.71E+06	9	9	7	6	2	4	Carbamid
902	3522	sp|Q15370|ELOB_HUMAN	199.12	6.74E+07	7.67E+07	5.92E+07	9.03E+07	11	11	7	9	8	10	Carbamid
1478	2089	sp|Q9UKS6|PACN3_HUMAN	198.96		8.51E+05	4.38E+06	6.29E+06	10	10	0	3	6	5	Carbamid
869	2147	sp|Q9Y6C9|MTCH2_HUMAN	198.77	1.38E+07	1.06E+07	1.01E+07	1.16E+07	12	12	11	6	7	9	Carbamid
545	2728	sp|P26373|RL13_HUMAN	198.62	1.48E+08	2.31E+08	1.48E+09	1.43E+09	14	14	10	9	19	19	Oxidatior
1395	1362	sp|Q96N67|DOCK7_HUMAN	198.58	8.40E+05	1.12E+06	1.50E+06	1.59E+06	22	19	1	6	3	1	Carbamid
1086	3461	sp|O75586|MED6_HUMAN	198.58	2.33E+07	3.59E+07	2.22E+07	2.42E+07	8	8	9	11	7	6	Carbamid
1086	3462	tr|G3V4A5|G3V4A5_HUMAN	198.58	2.33E+07	3.59E+07	2.22E+07	2.42E+07	8	8	9	11	7	6	Carbamid
1086	3463	tr|A0A087WYL7|A0A087WYL7_HUMAN	198.58	2.33E+07	3.59E+07	2.22E+07	2.42E+07	8	8	9	11	7	6	Carbamid
1538	1901	sp|O00566|MPP10_HUMAN	198.39	3.39E+06	1.11E+06	1.19E+07	5.79E+06	14	14	6	3	8	8	
1125	4082	sp|P40967|PMEL_HUMAN	198.21	6.82E+06	2.56E+07	3.53E+07	2.65E+07	12	12	4	9	6	5	Carbamid
1048	2492	sp|Q13363|CTBP1_HUMAN	197.87	2.25E+06	5.04E+06	1.69E+06	2.65E+06	10	5	8	7	6	6	Carbamid
1244	1428	tr|S4R3M9|S4R3M9_HUMAN	197.86	1.28E+07	2.07E+07	3.28E+06	1.98E+07	16	16	9	12	3	11	Carbamid
1244	1429	sp|O75764|TCEA3_HUMAN	197.86	1.28E+07	2.07E+07	3.28E+06	1.98E+07	16	16	9	12	3	11	Carbamid
1285	4957	sp|O00767|ACOD_HUMAN	197.75	1.17E+07	1.44E+07	3.20E+07	1.73E+07	8	8	5	3	4	5	Deamidat
1314	3242	sp|P09001|RM03_HUMAN	197.73	2.82E+06	6.01E+05	1.26E+07	6.61E+06	8	8	4	1	5	5	Carbamid
1314	3243	tr|H0Y9G6|H0Y9G6_HUMAN	197.73	2.82E+06	6.01E+05	1.26E+07	6.61E+06	8	8	4	1	5	5	Carbamid
1314	3244	tr|E7ETU7|E7ETU7_HUMAN	197.73	2.82E+06	6.01E+05	1.26E+07	6.61E+06	8	8	4	1	5	5	Carbamid
1370	3814	sp|Q9P287|BCCIP_HUMAN	197.7	1.52E+06	1.78E+06	4.40E+06	6.62E+06	7	7	2	6	3	5	Carbamid
1332	2389	tr|J3QTA6|J3QTA6_HUMAN	197.66	5.97E+06	7.08E+06	2.52E+06	4.82E+06	9	9	5	7	2	2	Carbamid
1332	2400	sp|Q9BRQ6|MIC25_HUMAN	197.66	5.97E+06	7.08E+06	2.52E+06	4.82E+06	9	9	5	7	2	2	Carbamid
1111	3091	tr|G3V2Q1|G3V2Q1_HUMAN	197.66	4.25E+06	6.22E+06	1.95E+07	2.53E+07	10	10	4	3	6	8	Deamidat
1111	3092	sp|P07910|HNRPC_HUMAN	197.66	4.25E+06	6.22E+06	1.95E+07	2.53E+07	10	10	4	3	6	8	Deamidat
1111	3105	tr|G3V576|G3V576_HUMAN	197.66	4.25E+06	6.22E+06	1.95E+07	2.53E+07	10	10	4	3	6	8	Deamidat
707	4098	sp|O14602|IF1AY_HUMAN	197.64	2.53E+07	2.28E+07	2.10E+07	2.41E+07	9	1	11	16	10	8	Carbamid
1488	2916	sp|O00560|SDCB1_HUMAN	197.6	0	2.31E+05	6.00E+06	1.07E+07	8	8	1	1	5	5	Carbamid
879	1280	sp|P26196|DDX6_HUMAN	197.43	1.22E+07	1.10E+07	1.71E+07	1.43E+07	14	14	10	9	8	8	Carbamid
1636	3990	sp|Q9UKY7|CDV3_HUMAN	197.42	2.12E+07	4.92E+06	1.15E+06	3.44E+06	7	7	6	3	1	2	
891	3752	sp|Q92522|H1X_HUMAN	197.42	2.10E+07	4.85E+07	1.10E+08	1.61E+08	7	7	4	9	11	14	Acetylatic
1267	1666	sp|Q9UKX7|NUP50_HUMAN	197.24	1.45E+07	8.28E+06	6.57E+06	4.24E+06	13	13	9	6	4	5	Carbamid
909	1118	sp|P19367|HXK1_HUMAN	197.17	1.14E+07	2.47E+06	4.55E+06	1.84E+06	25	19	15	12	9	5	Carbamid
1356	1151	sp|O94913|PCF11_HUMAN	196.77	1.57E+07	9.30E+06	4.21E+06	6.72E+06	16	16	15	6	3	5	Carbamid
1025	3739	sp|P13686|PPA5_HUMAN	196.54	1.84E+07	3.00E+07	3.35E+07	3.15E+07	8	8	6	7	6	11	Carbamid
720	2955	tr|V9GYY5|V9GYY5_HUMAN	196.53	1.63E+08	1.46E+08	1.78E+08	1.46E+08	13	13	14	12	12	13	
720	2956	sp|Q9UGY1|NOL12_HUMAN	196.53	1.63E+08	1.46E+08	1.78E+08	1.46E+08	13	13	14	12	12	13	
1380	2555	sp|Q9BVC5|ASHWN_HUMAN	196.52	2.56E+06	9.89E+05	2.21E+06	3.03E+06	10	10	2	2	3	3	
783	2068	sp|Q9UFW8|CGBP1_HUMAN	196.41	5.33E+07	8.11E+07	5.27E+07	6.42E+07	10	10	8	11	10	9	Carbamid
738	1597	sp|Q14978|NOLC1_HUMAN	196.27	6.80E+07	1.30E+08	4.53E+07	8.75E+07	16	16	11	18	12	11	Deamidat
738	1602	tr|A0A0A0MRM9|A0A0A0MRM9_HUMAN	196.27	6.80E+07	1.30E+08	4.53E+07	8.75E+07	16	16	11	18	12	11	Deamidat
598	1448	tr|A7XZE4|A7XZE4_HUMAN	196.19		4.61E+06		7.59E+06	15	2	9	13	13	14	
1377	3219	sp|Q9H1K1|ISCU_HUMAN	196.14	1.34E+07	1.31E+07	7.74E+05	6.17E+06	8	8	7	8	2	3	Carbamid
1351	1402	sp|Q9UPQ0|LIMC1_HUMAN	196.07	7.51E+05	2.96E+06		4.10E+06	23	23	2	7	0	6	Carbamid
1008	2554	sp|Q9NRX1|PNO1_HUMAN	196.03	2.24E+07	1.82E+07	3.89E+07	2.41E+07	10	10	6	6	9	8	Carbamid
1289	4201	sp|Q8IXM3|RM41_HUMAN	196.01	2.38E+06	1.25E+06	2.10E+07	2.53E+07	7	7	3	2	6	8	

1040	1506	sp|P13674|P4HA1_HUMAN	195.93	3.11E+06	1.23E+07	6.67E+06	6.58E+06	16	16	5	10	8	6	Carbamid
844	1356	tr|Q5JY65|Q5JY65_HUMAN	195.83	9.97E+06	3.10E+06	9.25E+06	1.40E+07	23	23	12	6	7	13	Carbamid
844	1357	sp|Q9BZJ0|CRNL1_HUMAN	195.83	9.97E+06	3.10E+06	9.25E+06	1.40E+07	23	23	12	6	7	13	Carbamid
1203	1977	tr|Q5T8C6|Q5T8C6_HUMAN	195.63	8.13E+06	5.92E+06	5.68E+06	3.07E+06	12	12	9	9	5	3	Carbamid
1203	1989	sp|Q13042|CDC16_HUMAN	195.63	8.13E+06	5.92E+06	5.68E+06	3.07E+06	12	12	9	9	5	3	Carbamid
995	2403	sp|Q9NVJ2|ARL8B_HUMAN	195.31	6.15E+06	7.61E+06	2.21E+07	1.47E+07	9	9	6	5	9	7	Carbamid
1318	6103	sp|P98179|RBM3_HUMAN	195.3	1.10E+08	7.31E+07	6.62E+06	5.90E+06	6	6	8	8	2	2	Oxidatio
475	3873	tr|J3QQQ9|J3QQQ9_HUMAN	195.18	3.01E+07	4.62E+07	2.61E+08	2.34E+08	13	2	14	12	17	21	Deamidat
475	3874	tr|J3QRI7|J3QRI7_HUMAN	195.18	3.01E+07	4.62E+07	2.61E+08	2.34E+08	13	2	14	12	17	21	Deamidat
475	3875	tr|J3QQV1|J3QQV1_HUMAN	195.18	3.01E+07	4.62E+07	2.61E+08	2.34E+08	13	2	14	12	17	21	Deamidat
475	3882	tr|J3QRC4|J3QRC4_HUMAN	195.18	3.01E+07	4.62E+07	2.61E+08	2.34E+08	13	2	14	12	17	21	Deamidat
475	3883	sp|P61254|RL26_HUMAN	195.18	3.01E+07	4.62E+07	2.61E+08	2.34E+08	13	2	14	12	17	21	Deamidat
1262	1990	sp|Q71RC2|LARP4_HUMAN	195.17	8.83E+06	5.55E+06	7.20E+06	8.22E+06	14	14	7	5	4	6	Carbamid
1551	2923	sp|Q9BT22|ALG1_HUMAN	195.17	2.27E+06	1.92E+06	1.55E+06	3.82E+06	8	8	5	4	3	6	Carbamid
976	2488	sp|P82979|SARNP_HUMAN	194.94	7.39E+07	3.38E+07	2.19E+07	2.32E+07	9	9	8	6	8	6	Acetylatio
1649	4110	sp|Q8TDP1|RNH2C_HUMAN	194.87	1.87E+06	2.18E+06	8.60E+05	5.05E+05	6	6	5	3	1	1	Carbamid
1649	4196	tr|H0YEF3|H0YEF3_HUMAN	194.87	1.87E+06	2.18E+06	8.60E+05	5.05E+05	6	6	5	3	1	1	Carbamid
1480	3536	tr|J3KR35|J3KR35_HUMAN	194.84	1.03E+07	3.59E+06	7.14E+06	1.38E+06	8	8	8	4	4	2	Acetylatio
1480	3570	sp|Q8WUD4|CCD12_HUMAN	194.84	1.03E+07	3.59E+06	7.14E+06	1.38E+06	8	8	8	4	4	2	Acetylatio
1046	2748	sp|P47985|UCRI_HUMAN	194.75	7.77E+06	7.98E+06	1.68E+06	3.00E+06	10	9	9	10	6	6	Carbamid
1030	1637	sp|P61964|WDR5_HUMAN	194.59	1.19E+07	1.01E+07	1.71E+07	1.25E+07	12	12	9	9	5	5	Carbamid
898	1857	sp|Q96CB9|NSUN4_HUMAN	194.57	2.16E+07	2.88E+07	2.45E+07	3.24E+07	13	13	9	9	8	8	Carbamid
1494	1988	sp|Q02241|KIF23_HUMAN	194.53	1.53E+06	1.59E+06	2.09E+06	3.55E+06	18	17	7	6	5	5	Carbamid
1245	1554	sp|P08779|K1C16_HUMAN	194.47			3.72E+06		16	4	8	3	6	1	Oxidatio
1317	4736	sp|P24534|EF1B_HUMAN	194.42	2.37E+07	1.28E+07	2.51E+07	1.91E+07	6	6	5	4	5	4	Carbamid
1447	2355	tr|A0A0C4DGA2|A0A0C4DGA2_HUMAN	194.3	2.93E+06	5.28E+06	9.93E+05	6.01E+05	9	9	4	6	1	1	Carbamid
1447	2357	sp|O75521|ECI2_HUMAN	194.3	2.93E+06	5.28E+06	9.93E+05	6.01E+05	9	9	4	6	1	1	Carbamid
1120	1438	sp|Q96C36|P5CR2_HUMAN	194.28	2.08E+06	3.28E+06	2.55E+06	5.04E+06	15	13	6	5	4	6	Carbamid
1165	1221	tr|E7EW05|E7EW05_HUMAN	194.2	9.76E+06	8.11E+07	1.33E+07	2.16E+07	18	17	4	13	6	8	Carbamid
1165	1228	sp|Q9NVU7|SDA1_HUMAN	194.2	9.76E+06	8.11E+07	1.33E+07	2.16E+07	18	17	4	13	6	8	Carbamid
1064	1347	sp|Q96PY5|FMNL2_HUMAN	194.19	2.64E+06	2.06E+06	1.97E+07	1.23E+07	17	17	5	5	12	7	Carbamid
1152	2958	sp|P82664|RT10_HUMAN	194.16	9.99E+06	8.44E+06	2.38E+07	1.30E+07	11	11	6	7	5	5	Carbamid
1316	4170	sp|Q16540|RM23_HUMAN	193.98	2.19E+06	2.08E+06	1.90E+07	1.97E+07	8	8	2	2	6	6	
477	1241	tr|H7BYY1|H7BYY1_HUMAN	193.85	2.73E+06	2.49E+07	1.10E+07	2.94E+07	22	4	10	14	17	16	Acetylatio
853	2317	sp|Q6PK04|CC137_HUMAN	193.79	9.88E+07	1.01E+08	5.78E+07	8.74E+07	12	12	13	13	8	13	
653	6797	sp|P61513|RL37A_HUMAN	193.65	1.94E+08	1.82E+08	4.48E+08	5.02E+08	10	10	9	7	14	28	Carbamid
653	6962	tr|C9J4Z3|C9J4Z3_HUMAN	193.65	1.94E+08	1.82E+08	4.48E+08	5.02E+08	10	10	9	7	14	28	Carbamid
1324	1252	sp|P53618|COPB_HUMAN	193.54	1.51E+06	2.45E+06	9.12E+05	9.59E+05	18	18	8	6	2	1	Carbamid
1345	4037	tr|F5H5N1|F5H5N1_HUMAN	193.41	2.06E+07	2.15E+07	2.34E+07	2.59E+07	8	8	6	4	3	4	Carbamid
1345	4039	sp|O75251|NDUS7_HUMAN	193.41	2.06E+07	2.15E+07	2.34E+07	2.59E+07	8	8	6	4	3	4	Carbamid
2232	3453	sp|Q01469|FABP5_HUMAN	193.3					8	8	0	0	0	0	Carbamid
1190	2799	sp|O43709|BUD23_HUMAN	193.2	5.62E+06	1.15E+07	4.90E+06	8.09E+06	8	8	8	9	5	5	Carbamid
838	5822	sp|P62266|RS23_HUMAN	193.07	6.66E+07	5.79E+07	9.40E+07	8.48E+07	8	8	18	5	7	7	Carbamid
1140	1201	sp|Q9BZL4|PP12C_HUMAN	192.9		7.54E+05		4.76E+05	19	1	8	9	6	11	Carbamid
1477	2734	sp|Q7LGA3|HS2ST_HUMAN	192.86		2.79E+06	2.82E+07	1.84E+07	9	9	0	3	8	5	Carbamid
1231	2747	sp|P13995|MTDC_HUMAN	192.7	7.15E+06	7.67E+06	8.66E+06	9.28E+06	9	9	6	4	4	7	Carbamid
1178	2505	sp|P32322|P5CR1_HUMAN	192.46	3.78E+06	4.12E+06	2.87E+06	1.76E+06	10	8	7	5	4	3	Carbamid
1338	1935	sp|Q9HC36|MRM3_HUMAN	192.06	8.63E+05	3.37E+06	4.56E+06	4.04E+07	12	12	2	5	2	5	Carbamid
1166	1299	sp|P17987|TCPA_HUMAN	192.04	3.14E+06	3.54E+06	2.42E+06	6.70E+05	16	12	9	8	4	3	Carbamid
929	4212	sp|Q13243|SRSF5_HUMAN	192	1.13E+06	1.32E+07	8.93E+07	9.99E+07	8	7	2	5	11	10	
1110	1314	sp|Q8N4C8|MINK1_HUMAN	191.76	4.16E+06	6.88E+06	4.15E+06	4.46E+06	16	10	9	13	6	6	Carbamid
1251	1866	sp|P13746|1A11_HUMAN	191.64	9.58E+05	9.94E+05			13	3	7	5	4	2	Carbamid
521	5236	sp|Q8N257|H2B3B_HUMAN	191.31	7.12E+06	8.66E+06	2.51E+07	3.14E+07	11	3	15	15	15	16	Oxidatio
1201	1132	sp|Q7L576|CYFP1_HUMAN	191.26	2.52E+06	5.37E+05	1.23E+06	2.73E+05	20	12	11	6	6	1	Carbamid
1186	1211	tr|K7EL81|K7EL81_HUMAN	191.22		5.12E+05			19	1	8	8	6	10	Carbamid
1144	2455	tr|E9PR44|E9PR44_HUMAN	191.13	1.37E+07	8.49E+06	2.57E+07	9.42E+06	9	9	6	5	9	4	Acetylatio
1144	2456	sp|P02511|CRYAB_HUMAN	191.13	1.37E+07	8.49E+06	2.57E+07	9.42E+06	9	9	6	5	9	4	Acetylatio
1335	1912	sp|O00154|BACH_HUMAN	191.06	7.00E+06	5.43E+06	1.19E+06	3.38E+06	11	11	6	5	2	4	Carbamid
1237	3622	sp|Q9UI30|TR112_HUMAN	191.04	8.41E+07	1.79E+07	1.76E+08	1.96E+08	7	7	6	6	4	6	Carbamid
522	5891	sp|P62807|H2B1C_HUMAN	190.94	3.52E+07	4.93E+07	1.07E+08	9.97E+07	11	3	15	15	15	16	Oxidatio
522	5892	sp|Q99880|H2B1L_HUMAN	190.94	3.52E+07	4.93E+07	1.07E+08	9.97E+07	11	3	15	15	15	16	Oxidatio
522	5893	sp|P58876|H2B1D_HUMAN	190.94	3.52E+07	4.93E+07	1.07E+08	9.97E+07	11	3	15	15	15	16	Oxidatio
522	5894	sp|Q99877|H2B1N_HUMAN	190.94	3.52E+07	4.93E+07	1.07E+08	9.97E+07	11	3	15	15	15	16	Oxidatio
522	5895	sp|O60814|H2B1K_HUMAN	190.94	3.52E+07	4.93E+07	1.07E+08	9.97E+07	11	3	15	15	15	16	Oxidatio
522	5896	sp|Q99879|H2B1M_HUMAN	190.94	3.52E+07	4.93E+07	1.07E+08	9.97E+07	11	3	15	15	15	16	Oxidatio
522	5897	sp|Q93079|H2B1H_HUMAN	190.94	3.52E+07	4.93E+07	1.07E+08	9.97E+07	11	3	15	15	15	16	Oxidatio
522	5898	sp|Q5QNW6|H2B2F_HUMAN	190.94	3.52E+07	4.93E+07	1.07E+08	9.97E+07	11	3	15	15	15	16	Oxidatio
522	5899	sp|P57053|H2BFS_HUMAN	190.94	3.52E+07	4.93E+07	1.07E+08	9.97E+07	11	3	15	15	15	16	Oxidatio
522	5904	tr|U3KQK0|U3KQK0_HUMAN	190.94	3.52E+07	4.93E+07	1.07E+08	9.97E+07	11	3	15	15	15	16	Oxidatio
1096	6051	sp|P20671|H2A1D_HUMAN	190.85		1.94E+06	7.92E+05	4.98E+06	9	4	5	7	6	9	Deamidat
1096	6059	tr|A0A0U1RR32|A0A0U1RR32_HUMAN	190.85		1.94E+06	7.92E+05	4.98E+06	9	4	5	7	6	9	Deamidat
1096	6060	tr|A0A0U1RRH7|A0A0U1RRH7_HUMAN	190.85		1.94E+06	7.92E+05	4.98E+06	9	4	5	7	6	9	Deamidat
1096	6292	sp|Q99878|H2A1J_HUMAN	190.85		1.94E+06	7.92E+05	4.98E+06	9	4	5	7	6	9	Deamidat
1096	6293	sp|Q96KK5|H2A1H_HUMAN	190.85		1.94E+06	7.92E+05	4.98E+06	9	4	5	7	6	9	Deamidat
1096	6295	sp|Q9BTM1|H2AJ_HUMAN	190.85		1.94E+06	7.92E+05	4.98E+06	9	4	5	7	6	9	Deamidat
1096	6296	sp|P0C0S8|H2A1_HUMAN	190.85		1.94E+06	7.92E+05	4.98E+06	9	4	5	7	6	9	Deamidat
1096	6583	sp|Q16777|H2A2C_HUMAN	190.85		1.94E+06	7.92E+05	4.98E+06	9	4	5	7	6	9	Deamidat
1096	6584	sp|Q6FI13|H2A2A_HUMAN	190.85		1.94E+06	7.92E+05	4.98E+06	9	4	5	7	6	9	Deamidat
1096	6616	sp|P04908|H2A1B_HUMAN	190.85		1.94E+06	7.92E+05	4.98E+06	8	3	5	7	6	9	Deamidat
1096	6617	sp|Q7L7L0|H2A3_HUMAN	190.85		1.94E+06	7.92E+05	4.98E+06	8	3	5	7	6	9	Deamidat
1096	6690	sp|Q93077|H2A1C_HUMAN	190.85		1.94E+06	7.92E+05	4.98E+06	8	3	5	7	6	9	Deamidat
1225	1306	sp|P17858|PFKAL_HUMAN	190.81	3.57E+06	2.15E+06	2.27E+06	1.80E+06	15	11	9	5	4	4	Carbamid
1279	1450	sp|O00231|PSD11_HUMAN	190.75	7.51E+06	2.45E+06	8.98E+05	2.10E+06	13	13	9	5	2	3	Carbamid
1149	1771	sp|P43155|CACP_HUMAN	190.72	1.66E+07	1.64E+07	1.04E+07	7.52E+06	14	14	9	8	4	4	Carbamid
1187	1759	sp|Q15392|DHC24_HUMAN	190.51	2.12E+07	1.42E+07	1.62E+07	1.88E+07	13	13	8	6	4	5	Carbamid
1187	1760	tr|A0A0A0MTI1|A0A0A0MTI1_HUMAN	190.51	2.12E+07	1.42E+07	1.62E+07	1.88E+07	13	13	8	6	4	5	Carbamid
1652	2541	sp|Q14258|TRI25_HUMAN	190.3	1.23E+06	1.44E+06	4.85E+06	2.86E+06	9	9	2	2	4	3	Carbamid
1580	2948	sp|P48745|CCN3_HUMAN	190.26	1.97E+07	4.32E+06	2.94E+06	2.64E+06	9	9	8	3	3	1	Carbamid
1291	1528	sp|O60341|KDM1A_HUMAN	190.12	9.46E+05	2.96E+05	1.81E+06	3.35E+06	15	15	2	1	3	6	Carbamid
1291	1529	tr|R4GMQ1|R4GMQ1_HUMAN	190.12	9.46E+05	2.96E+05	1.81E+06	3.35E+06	15	15	2	1	3	6	Carbamid
1584	4564	sp|O43513|MED7_HUMAN	190.06	8.16E+06	8.56E+06	1.19E+07	4.32E+06	6	6	6	5	4	2	Carbamid
1199	2126	sp|Q9H6F5|CCD86_HUMAN	189.83	1.70E+07	1.95E+07	1.61E+07	2.45E+07	12	12	5	8	4	5	Carbamid
1535	2038	sp|O43159|RRP8_HUMAN	189.71	3.69E+06	5.09E+06	7.74E+06	7.58E+06	12	12	4	4	3	12	Carbamid
1098	1845	sp|P22830|HEMH_HUMAN	189.62	1.06E+07	1.02E+07	4.10E+06	6.04E+06	15	15	9	7	4	5	Carbamid
1167	1755	sp|Q86UY8|NT5D3_HUMAN	189.53	3.82E+06	3.68E+06	3.95E+06	1.25E+06	12	11	7	7	4	4	
1239	5029	sp|Q13185|CBX3_HUMAN	189.45	1.73E+07	6.76E+06	5.84E+06	1.14E+07	7	5	7	5	5	6	Carbamid
2030	4009	sp|Q96EY5|MB12A_HUMAN	189.28	5.40E+06	1.73E+06		4.78E+05	6	6	5	3	0	1	Carbamid
2030	4010	tr|E9PQA6|E9PQA6_HUMAN	189.28	5.40E+06	1.73E+06		4.78E+05	6	6	5	3	0	1	Carbamid
1039	2497	sp|Q9BYN8|RT26_HUMAN	189.28	1.30E+07	2.40E+07	4.17E+07	3.67E+07	13	13	6	9	9	7	
1721	7003	sp|P23511|NFYA_HUMAN	189.19	2.84E+06	1.32E+07	5.54E+06	5.62E+06	4	4	2	4	2	2	
1257	2668	sp|Q15366|PCBP2_HUMAN	189.15	2.16E+06	1.96E+06	5.38E+06	1.61E+06	8	3	6	4	5	4	Carbamid
1834	3613	tr|Q96SH1|Q96SH1_HUMAN	189.13	7.07E+05	1.32E+06	2.33E+06	3.18E+06	7	7	2	3	2	3	Carbamid
1834	3614	sp|P05549|AP2A_HUMAN	189.13	7.07E+05	1.32E+06	2.33E+06	3.18E+06	7	7	2	3	2	3	Carbamid
1216	6087	sp|Q9BWJ5|SF3B5_HUMAN	189.13	1.42E+08	2.89E+08	8.49E+07	2.19E+08	6	6	7	10	3	4	Carbamid
1198	1278	sp|Q93074|MED12_HUMAN	188.98	5.49E+05	4.94E+06	1.71E+06	4.02E+06	23	21	5	11	7	10	Carbamid
1598	4389	sp|Q7Z2W9|RM21_HUMAN	188.83	9.14E+05	7.98E+05	9.71E+06	3.95E+06	7	7	3	3	6	5	Carbamid
895	5052	sp|Q96BP2|CHCH1_HUMAN	188.56	8.64E+07	1.81E+08	5.91E+07	1.59E+08	9	9	8	12	4	6	Carbamid
1112	1515	tr|Q5T5C7|Q5T5C7_HUMAN	188.52	1.13E+07	4.05E+06	5.62E+06	1.16E+06	17	17	7	5	5	1	Carbamid
1112	1519	sp|P49591|SYSC_HUMAN	188.52	1.13E+07	4.05E+06	5.62E+06	1.16E+06	17	17	7	5	5	1	Carbamid
1183	1925	tr|E7EWD3|E7EWD3_HUMAN	188.5	1.29E+07	7.74E+06	8.72E+06	6.41E+06	15	15	10	6	5	8	Carbamid
1183	1926	sp|O75077|ADA23_HUMAN	188.5	1.29E+07	7.74E+06	8.72E+06	6.41E+06	15	15	10	6	5	8	Carbamid
1777	2255	sp|Q12792|TWF1_HUMAN	188.41		2.62E+07	9.04E+05	7.36E+06	12	9	0	3	1	4	Carbamid
1621	2641	sp|Q9H444|CHM4B_HUMAN	188.33	9.80E+05	9.06E+06	4.49E+06	1.52E+07	11	11	1	6	2	5	Deamidat
1280	1600	tr|E7EPM6|E7EPM6_HUMAN	188.32	1.41E+06	3.18E+06		3.43E+06	20	20	3	4	0	5	Carbamid
1280	1612	sp|P33121|ACSL1_HUMAN	188.32	1.41E+06	3.18E+06		3.43E+06	20	20	3	4	0	5	Carbamid
1309	4623	sp|P84085|ARF5_HUMAN	188.24	2.08E+05	1.07E+05			6	1	5	5	6	1	Carbamid

1309	4853	tr|C9J1Z8|C9J1Z8_HUMAN	188.24	2.08E+05	1.07E+05			6	1	5	5	6	1	Carbamid
769	3951	sp|Q6P1L8|RM14_HUMAN	188.22	2.18E+08	2.72E+08	2.10E+08	2.41E+08	9	9	11	9	11	13	Carbamid
1451	3033	sp|Q9NZM5|NOP53_HUMAN	188.19	1.59E+07	3.18E+07	4.24E+07	5.37E+07	9	9	5	7	5	6	Acetylatic
950	2616	sp|Q9H0U3|MAGT1_HUMAN	188.17	2.76E+07	3.00E+07	6.70E+07	5.95E+07	11	11	8	7	11	8	
950	2623	tr|A0A087WU53|A0A087WU53_HUMAN	188.17	2.76E+07	3.00E+07	6.70E+07	5.95E+07	11	11	8	7	11	8	
1197	1527	sp|Q9UBP9|GULP1_HUMAN	188.06	1.66E+07	1.40E+07	5.27E+06	5.02E+06	13	13	10	9	6	6	Carbamid
1067	1931	sp|Q14004|CDK13_HUMAN	188.01	2.78E+06	7.61E+05	2.38E+06	7.41E+05	15	7	12	11	9	5	Carbamid
1432	2774	sp|O75396|SC22B_HUMAN	187.66	9.45E+06	6.74E+06	6.44E+06	4.52E+06	10	10	7	5	4	2	
1411	6136	sp|Q9BRT6|LLPH_HUMAN	187.62	5.38E+07	3.94E+07	5.93E+07	7.44E+07	5	5	7	4	4	5	Carbamid
1050	2939	tr|A0A087WUQ6|A0A087WUQ6_HUMAN	187.52	1.76E+07	2.23E+07	1.49E+07	2.06E+07	10	10	10	10	5	8	Carbamid
1050	2940	sp|P07203|GPX1_HUMAN	187.52	1.76E+07	2.23E+07	1.49E+07	2.06E+07	10	10	10	10	5	8	Carbamid
1210	2698	tr|F5GWD3|F5GWD3_HUMAN	187.38	1.86E+07	3.27E+07	2.38E+07	3.87E+07	8	8	5	8	7	8	Carbamid
1210	2705	sp|Q13889|TF2H3_HUMAN	187.38	1.86E+07	3.27E+07	2.38E+07	3.87E+07	8	8	5	8	7	8	Carbamid
1294	2257	sp|Q9BTA9|WAC_HUMAN	187.28	6.47E+06	9.79E+06	4.53E+06	4.45E+06	10	10	7	7	3	4	Carbamid
1384	2529	sp|O94992|HEXI1_HUMAN	187.26	6.36E+06	1.88E+07	4.55E+06	2.60E+06	9	9	7	7	2	4	Carbamid
890	2128	sp|P48729|KC1A_HUMAN	187.1	7.95E+06	2.18E+07	2.49E+07	1.81E+07	9	9	9	11	9	7	Carbamid
1142	1887	sp|Q92945|FUBP2_HUMAN	187.09	8.26E+06	1.62E+07	1.11E+07	1.04E+07	11	11	5	12	7	6	Carbamid
1142	1888	tr|A0A087WTP3|A0A087WTP3_HUMAN	187.09	8.26E+06	1.62E+07	1.11E+07	1.04E+07	11	11	5	12	7	6	Carbamid
1707	3362	sp|Q15417|CNN3_HUMAN	187.01	1.15E+07	2.76E+06	2.32E+05	7.44E+05	7	6	6	4	1	1	Carbamid
1182	1458	sp|Q9H4M9|EHD1_HUMAN	186.99	1.43E+06		5.11E+05	6.52E+05	17	9	10	2	5	4	Deamidat
1182	1464	tr|A0A024R571|A0A024R571_HUMAN	186.99	1.43E+06		5.11E+05	6.52E+05	17	9	10	2	5	4	Deamidat
1407	2708	sp|Q330K2|NDUF6_HUMAN	186.89	1.48E+06	1.87E+06	4.15E+06	3.12E+06	8	8	4	4	5	4	Carbamid
1017	1361	sp|Q9BUQ8|DDX23_HUMAN	186.75	7.38E+06	1.82E+07	6.56E+06	1.19E+07	22	22	10	14	9	8	Carbamid
1354	1678	sp|Q9Y490|TLN1_HUMAN	186.67	1.05E+07	2.62E+06	6.18E+05		17	17	14	4	1	0	Carbamid
1360	1841	sp|Q9UQ80|PA2G4_HUMAN	186.67	1.02E+07		1.34E+07	1.20E+07	11	11	6	0	6	7	Carbamid
1536	1526	sp|P48681|NEST_HUMAN	186.63	3.46E+05	1.20E+06		5.73E+05	16	16	2	3	0	2	
985	4718	tr|J3QR09|J3QR09_HUMAN	186.62	1.98E+07	2.85E+07	2.35E+08	2.59E+08	8	8	6	5	8	12	Oxidatior
985	4719	tr|J3KTE4|J3KTE4_HUMAN	186.62	1.98E+07	2.85E+07	2.35E+08	2.59E+08	8	8	6	5	8	12	Oxidatior
985	4720	sp|P84098|RL19_HUMAN	186.62	1.98E+07	2.85E+07	2.35E+08	2.59E+08	8	8	6	5	8	12	Oxidatior
1434	2214	sp|Q6P6C2|ALKB5_HUMAN	186.49	3.20E+06	1.14E+07	5.37E+06	6.72E+06	10	10	3	6	5	4	Carbamid
1310	2228	sp|Q15691|MARE1_HUMAN	186.45	1.84E+07	5.67E+06	5.37E+06	1.28E+06	11	10	10	6	4	1	Acetylatic
1246	1951	sp|Q9UBX3|DIC_HUMAN	186.22	7.63E+06	7.58E+06	1.49E+07	8.06E+06	12	2	6	9	3	5	Carbamid
1213	3318	sp|Q9NV31|IMP3_HUMAN	185.87	6.59E+06	6.26E+06	1.48E+07	8.88E+06	8	8	6	7	7	6	Carbamid
1462	1949	sp|Q13823|NOG2_HUMAN	185.78	1.35E+07	1.47E+07	1.98E+07	2.22E+07	13	13	4	3	9	8	Carbamid
771	7467	sp|A0A075B6S2|KVD29_HUMAN	185.78	1.01E+07	1.13E+07	1.45E+10	7.44E+09	14	9	2	3	18	16	
1474	2109	sp|Q13769|THOC5_HUMAN	185.51	3.80E+06	6.59E+06	8.89E+06	5.97E+06	12	12	5	8	5	5	Carbamid
975	2447	tr|A0A0A0MR02|A0A0A0MR02_HUMAN	185.31	1.36E+07	9.57E+06	1.22E+07	1.33E+07	11	10	8	7	7	6	Carbamid
975	2448	sp|P45880|VDAC2_HUMAN	185.31	1.36E+07	9.57E+06	1.22E+07	1.33E+07	11	10	8	7	7	6	Carbamid
1232	1891	sp|O00139|KIF2A_HUMAN	185.23		1.58E+06	1.22E+07	9.57E+06	14	12	0	5	9	11	Carbamid
963	4023	sp|Q9UBC5|MYO1A_HUMAN	185.04					7	1	5	8	5	7	Carbamid
981	1802	sp|Q68D10|SPT2_HUMAN	184.84	3.86E+07	7.50E+07	3.27E+07	5.36E+07	16	16	13	15	12	8	Deamidat
1751	2354	sp|P78406|RAE1L_HUMAN	184.7	1.69E+05	1.01E+06	4.20E+05	9.79E+05	10	10	2	2	1	1	Carbamid
1555	1493	sp|Q9HD20|AT131_HUMAN	184.61	1.27E+06		8.80E+05	1.09E+06	13	13	4	0	2	4	Carbamid
1516	3135	sp|P48651|PTSS1_HUMAN	184.6	2.10E+06	1.36E+07	1.18E+07	1.80E+07	8	8	4	3	4	7	Carbamid
1418	2687	sp|Q5VUA4|ZN318_HUMAN	184.54	1.60E+07	1.26E+07	1.05E+07	1.20E+07	12	12	11	7	4	2	Carbamid
1528	4891	sp|O75380|NDUS6_HUMAN	184.52	1.82E+07	1.27E+07	1.14E+07	1.04E+07	7	7	6	3	4	3	Carbamid
1531	4116	sp|P01111|RASN_HUMAN	184.49			4.13E+05		9	1	4	3	8	2	Carbamid
927	2482	sp|Q9Y6A4|CFA20_HUMAN	184.45	2.98E+07	4.39E+07	4.99E+07	4.81E+07	12	12	7	9	11	7	Carbamid
908	1542	tr|A0A2R8Y7M3|A0A2R8Y7M3_HUMAN	184.38	3.05E+06	1.02E+06	8.44E+05	1.10E+06	19	9	14	8	11	10	Carbamid
908	1557	sp|P35241|RADI_HUMAN	184.38	3.05E+06	1.02E+06	8.44E+05	1.10E+06	19	9	14	8	11	10	Carbamid
908	1559	tr|A0A2R8Y5S7|A0A2R8Y5S7_HUMAN	184.38	3.05E+06	1.02E+06	8.44E+05	1.10E+06	19	9	14	8	11	10	Carbamid
1019	1829	sp|P27448|MARK3_HUMAN	184.37	8.13E+06	8.71E+06	3.98E+06	5.58E+06	16	9	11	8	7	7	Carbamid
955	3426	sp|Q9GZT3|SLIRP_HUMAN	184.29	2.91E+07	2.41E+07	6.39E+07	4.03E+07	9	9	8	4	11	7	Carbamid
1436	3006	sp|Q9UJA5|TRM6_HUMAN	184.26	1.28E+06	4.40E+05	1.73E+06	2.13E+06	9	9	4	2	2	5	Carbamid
931	1742	sp|P21912|SDHB_HUMAN	184.11	1.67E+07	8.85E+06	3.33E+06	9.84E+06	13	13	12	10	4	7	Carbamid
1807	4741	sp|O15511|ARPC5_HUMAN	184.08	1.11E+06	9.78E+06		9.22E+06	5	4	2	4	0	4	Carbamid
1191	3218	sp|Q92504|S39A7_HUMAN	183.91	6.62E+07	6.07E+07	2.73E+07	4.90E+07	8	8	7	7	5	4	Carbamid
575	3904	sp|Q9UNX3|RL26L_HUMAN	183.61			1.19E+07		12	1	11	9	15	17	Deamidat
1078	2184	sp|O60684|IMA7_HUMAN	183.59	1.11E+06	1.67E+06	1.33E+06	3.21E+06	11	3	8	6	3	5	Carbamid
1073	1454	sp|Q969X6|UTP4_HUMAN	183.45	1.85E+06	5.84E+06	5.89E+06	2.32E+06	15	15	4	12	6	5	Carbamid
1545	3226	sp|Q00577|PURA_HUMAN	183.36	2.08E+05	1.51E+06	9.69E+06	9.87E+06	8	7	1	2	10	6	Carbamid
1684	3115	sp|O95243|MBD4_HUMAN	183.18	7.34E+06	7.02E+06	3.72E+06	3.55E+06	7	7	6	6	3	4	Carbamid
1023	1791	sp|P63092|GNAS2_HUMAN	183.14	1.21E+07	2.59E+06	1.61E+06	2.83E+06	13	9	16	4	4	3	Carbamid
1023	1820	sp|Q5JWF2|GNAS1_HUMAN	183.14	1.21E+07	2.59E+06	1.61E+06	2.83E+06	13	9	16	4	4	3	Carbamid
1771	2764	tr|E9PFR3|E9PFR3_HUMAN	183.09	1.13E+06	1.42E+06		4.20E+05	8	6	2	3	0	2	Carbamid
1771	2765	sp|Q14738|2A5D_HUMAN	183.09	1.13E+06	1.42E+06		4.20E+05	8	6	2	3	0	2	Carbamid
1402	3058	sp|Q9Y679|AUP1_HUMAN	182.58	4.24E+06	4.90E+06	1.18E+06	4.35E+06	7	7	5	5	4	4	Carbamid
1490	2153	sp|Q9P015|RM15_HUMAN	182.36	3.42E+05	1.47E+06	3.93E+06	5.00E+06	10	10	1	2	3	7	Carbamid
1490	2193	tr|E5RIZ4|E5RIZ4_HUMAN	182.36	3.42E+05	1.47E+06	3.93E+06	5.00E+06	10	10	1	2	3	7	Carbamid
1481	4055	sp|Q9H204|MED28_HUMAN	182.25	1.22E+07	2.52E+07	1.63E+07	1.59E+07	6	6	4	5	3	6	Carbamid
1523	2047	sp|Q8TBZ3|WDR20_HUMAN	181.88	1.85E+06	3.28E+06		3.38E+06	11	11	2	5	0	4	Carbamid
1523	2049	tr|A0A088AWN2|A0A088AWN2_HUMAN	181.88	1.85E+06	3.28E+06		3.38E+06	11	11	2	5	0	4	Carbamid
1415	2841	sp|Q9NQX4|MYO5C_HUMAN	181.61			2.05E+04	1.43E+06	9	2	0	4	6	7	Carbamid
1319	2985	sp|Q9BUP0|EFHD1_HUMAN	181.58		7.54E+06	1.60E+06	9.89E+06	8	6	2	8	2	6	
1185	1367	sp|O00410|IPO5_HUMAN	181.47	2.99E+06	2.07E+06	2.78E+06	1.16E+06	17	17	7	8	4	2	Carbamid
1185	1368	tr|H0Y8C6|H0Y8C6_HUMAN	181.47	2.99E+06	2.07E+06	2.78E+06	1.16E+06	17	17	7	8	4	2	Carbamid
1456	1796	sp|Q14683|SMC1A_HUMAN	181.46	1.09E+06	2.45E+05	7.20E+05	9.51E+05	16	16	4	2	3	2	Carbamid
1080	1881	tr|A0A0A0MQR2|A0A0A0MQR2_HUMAN	181.43	2.27E+07	1.21E+07	7.69E+06	1.06E+07	11	11	9	7	4	9	Carbamid
1080	1886	sp|Q9BY42|RTF2_HUMAN	181.43	2.27E+07	1.21E+07	7.69E+06	1.06E+07	11	11	9	7	4	9	Carbamid
1238	3909	sp|P06730|IF4E_HUMAN	181.38	9.64E+06	3.16E+07	1.75E+07	2.46E+07	7	7	3	12	3	5	Carbamid
1205	1848	tr|F5GZS6|F5GZS6_HUMAN	181.31	5.38E+06	4.38E+06	3.64E+06	4.16E+06	13	13	5	7	5	5	
1205	1849	sp|P08195|4F2_HUMAN	181.31	5.38E+06	4.38E+06	3.64E+06	4.16E+06	13	13	5	7	5	5	
1205	1850	tr|J3KPF3|J3KPF3_HUMAN	181.31	5.38E+06	4.38E+06	3.64E+06	4.16E+06	13	13	5	7	5	5	
1441	3023	sp|Q9BSF4|TIM29_HUMAN	181.3	5.73E+06	2.16E+06	6.48E+06	3.30E+06	8	8	6	5	4	3	Carbamid
1450	3237	sp|P84095|RHOG_HUMAN	181.26	2.76E+06	1.94E+06	1.53E+06	1.31E+06	8	7	5	4	3	1	Carbamid
1348	6089	sp|P51571|SSRD_HUMAN	181.18	1.84E+07	1.28E+07	3.25E+07	2.57E+07	4	4	5	4	4	4	Deamidat
1849	5082	sp|O75607|NPM3_HUMAN	181.16		1.36E+06	1.89E+06	5.75E+06	5	5	0	3	1	4	Carbamid
1215	6963	sp|O14880|MGST3_HUMAN	180.83	1.17E+07	1.18E+07	2.60E+07	1.51E+07	5	5	6	6	6	4	Carbamid
1215	6964	tr|Q5VV89|Q5VV89_HUMAN	180.83	1.17E+07	1.18E+07	2.60E+07	1.51E+07	5	5	6	6	6	4	Carbamid
1090	1818	sp|P57729|RAB38_HUMAN	180.63	4.82E+06	4.70E+06	8.27E+06	3.68E+06	11	9	5	4	8	3	Carbamid
1359	2994	sp|O00422|SAP18_HUMAN	180.35	1.62E+07	1.82E+07	2.05E+07	1.21E+07	10	10	6	9	10	4	Carbamid
1359	3241	tr|H7BZW6|H7BZW6_HUMAN	180.35	1.62E+07	9.82E+06	2.05E+07	1.21E+07	9	9	6	7	10	4	Carbamid
1153	6124	sp|P84090|ERH_HUMAN	180.27	1.11E+08	9.74E+07	1.24E+08	1.35E+08	6	6	5	7	8	7	Carbamid
1284	9292	sp|P60903|S10AA_HUMAN	180.18	7.61E+07	5.79E+07	6.53E+07	4.50E+07	8	8	7	8	5	4	
1396	1434	sp|Q9NQ66|PLCB1_HUMAN	179.7	1.19E+06	8.88E+06	5.51E+04	6.42E+05	14	14	6	8	1	4	Carbamid
1614	2454	sp|Q9Y6V0|PCLO_HUMAN	179.69	5.06E+06	1.70E+06	2.93E+06	3.60E+05	10	10	5	3	5	2	Carbamid
1409	5091	sp|Q9NX55|HYPK_HUMAN	179.64	9.87E+06	5.63E+06	5.99E+06	4.15E+06	6	6	5	5	5	4	Acetylatic
658	2941	sp|P40429|RL13A_HUMAN	179.63	4.42E+07	1.30E+08	8.83E+08	8.90E+08	10	10	6	7	17	17	Carbamid
1256	1966	sp|Q9NVH1|DJC11_HUMAN	179.58	4.49E+06	4.41E+06	6.71E+05	2.09E+06	12	12	7	6	3	3	Carbamid
1878	2041	sp|O60701|UGDH_HUMAN	179.53	2.44E+06	1.64E+06		2.62E+06	10	10	6	1	0	3	Carbamid
1860	2240	tr|A0A2R8Y840|A0A2R8Y840_HUMAN	179.49	3.26E+06	1.02E+07		5.03E+06	12	10	2	10	0	4	Carbamid
1860	2264	sp|Q9HCK8|CHD8_HUMAN	179.49	3.26E+06	1.02E+07		5.03E+06	12	10	2	10	0	4	Carbamid
1387	1490	tr|G8JLD3|G8JLD3_HUMAN	179.46	1.33E+06	1.38E+06	1.01E+07	9.29E+05	14	14	4	4	2	2	Carbamid
1387	1491	sp|Q8IUD2|RB6I2_HUMAN	179.46	1.33E+06	1.38E+06	1.01E+07	9.29E+05	14	14	4	4	2	2	Carbamid
1387	1492	tr|X6RLX0|X6RLX0_HUMAN	179.46	1.33E+06	1.38E+06	1.01E+07	9.29E+05	14	14	4	4	2	2	Carbamid
1735	3632	sp|Q9NSY1|BMP2K_HUMAN	179.31	2.26E+07	1.07E+07	4.61E+06	1.33E+06	7	6	6	6	3	2	Carbamid
1147	2113	sp|P30040|ERP29_HUMAN	178.99	6.10E+07	2.75E+07	1.04E+07	1.92E+07	10	10	10	5	4	5	
1557	1719	tr|Q5THR1|Q5THR1_HUMAN	178.95	2.30E+06	5.52E+06	4.10E+06	3.56E+06	12	12	5	5	6	5	Carbamid
1557	1721	sp|Q9H5Z1|DHX35_HUMAN	178.95	2.30E+06	5.52E+06	4.10E+06	3.56E+06	12	12	5	5	6	5	Carbamid
1416	1846	sp|Q13884|SNTB1_HUMAN	178.76	6.81E+06	7.56E+06	5.36E+06	3.31E+06	14	14	7	6	4	4	Carbamid
1589	2517	sp|Q9GZR2|REXO4_HUMAN	178.58	2.25E+07	1.95E+07	1.55E+07	2.05E+07	10	10	5	5	5	5	Carbamid
1463	4100	sp|O15258|RER1_HUMAN	178.48	1.50E+07	8.48E+06	2.39E+07	2.26E+07	9	9	5	3	5	5	Acetylatic
1463	4485	tr|A0A0A0MR06|A0A0A0MR06_HUMAN	178.48	1.50E+07	8.48E+06	2.04E+07	2.26E+07	8	8	5	3	4	5	Acetylatic
1463	4575	tr|Q5T092|Q5T092_HUMAN	178.48	1.50E+07	8.48E+06	2.04E+07	2.26E+07	8	8	5	3	4	5	Acetylatic
1412	7184	sp|O43678|NDUA2_HUMAN	178.46	1.91E+07	1.51E+07	7.35E+06	9.07E+06	5	5	5	4	3	2	Deamidat
1322	1811	sp|Q9C037|TRIM4_HUMAN	178.42	3.74E+06	8.20E+06	4.56E+06	4.30E+06	14	14	3	9	5	4	Carbamid

923	4241	sp|P14854|CX6B1_HUMAN	178.24	3.42E+07	2.46E+07	1.71E+07	2.21E+07	6	6	7	9	6	11	Carbamid
1015	4246	sp|P55769|NH2L1_HUMAN	178.2	4.78E+07	5.58E+07	4.97E+07	7.76E+07	11	11	10	9	6	7	Carbamid
1015	4247	tr|B1AHD1|B1AHD1_HUMAN	178.2	4.78E+07	5.58E+07	4.97E+07	7.76E+07	10	10	10	9	6	7	Carbamid
1497	2942	sp|Q96CS3|FAF2_HUMAN	178.12	3.63E+06	3.66E+06	6.60E+06	1.49E+06	8	8	4	5	5	2	Carbamid
1003	2103	sp|Q00059|TFAM_HUMAN	178.05	1.28E+07	3.35E+07	1.39E+06	2.82E+07	13	13	9	9	1	7	Carbamid
1435	2674	tr|A0A286YF22|A0A286YF22_HUMAN	177.92	5.99E+06	2.13E+06	6.09E+06	5.32E+06	8	7	5	2	6	5	Carbamid
1435	2675	tr|A0A2C9F2M7|A0A2C9F2M7_HUMAN	177.92	5.99E+06	2.13E+06	6.09E+06	5.32E+06	8	7	5	2	6	5	Carbamid
1435	2676	sp|O43175|SERA_HUMAN	177.92	5.99E+06	2.13E+06	6.09E+06	5.32E+06	8	7	5	2	6	5	Carbamid
1235	3046	sp|Q92820|GGH_HUMAN	177.67	1.11E+07	1.00E+07	6.01E+06	7.08E+06	9	9	8	8	4	5	Deamidat
1288	3125	sp|Q3B726|RPA43_HUMAN	177.48	1.85E+07	4.15E+07	1.14E+07	3.42E+07	8	7	6	8	4	6	Carbamid
1009	3714	tr|A0A0B4J220|A0A0B4J220_HUMAN	177.42	6.10E+07	1.13E+08	1.50E+08	1.62E+08	9	7	6	8	7	9	Oxidation
1101	3267	sp|Q15404|RSU1_HUMAN	177.29	1.04E+07	6.66E+06	1.13E+07	6.30E+06	8	8	8	5	6	6	
1472	2056	sp|Q14498|RBM39_HUMAN	177.28	4.46E+06	5.11E+06	4.84E+06	5.79E+06	10	10	5	6	4	6	Deamidat
1214	3934	sp|Q15629|TRAM1_HUMAN	177.18	1.49E+07	1.81E+07	4.55E+07	3.77E+07	8	8	5	5	8	5	Deamidat
1214	4236	tr|G3XAN4|G3XAN4_HUMAN	177.18	1.49E+07	1.81E+07	4.55E+07	3.77E+07	8	8	5	5	8	5	Deamidat
1342	3971	sp|Q9Y2Q9|RT28_HUMAN	176.98	1.03E+07	1.79E+07	2.34E+07	3.18E+07	6	6	4	5	4	4	Oxidation
1656	2213	sp|Q9UKN8|TF3C4_HUMAN	176.89	1.59E+05		9.23E+06	8.61E+06	14	14	2	0	7	8	Carbamid
1518	2273	sp|Q14558|KPRA_HUMAN	176.78	2.67E+06	3.16E+06	9.61E+05	1.68E+05	11	11	5	9	2	1	Deamidat
1108	1777	sp|Q9NTJ5|SAC1_HUMAN	176.6	4.91E+06	5.43E+06	7.37E+06	2.61E+06	17	16	5	9	7	3	Carbamid
855	3751	tr|H3BN98|H3BN98_HUMAN	176.54	0		6.96E+05	2.18E+06	8	2	8	9	9	13	Carbamid
1941	2166	sp|Q9NZ32|ARP10_HUMAN	176.44	1.10E+05	1.30E+06		5.96E+05	11	11	1	2	0	2	Carbamid
1327	1665	sp|P38159|RBMX_HUMAN	176.36	8.01E+07	6.48E+07	9.69E+05	4.70E+05	14	14	10	11	1	1	Acetylatic
1571	2067	sp|P43246|MSH2_HUMAN	176.05	2.25E+06	1.11E+06	2.84E+06	4.96E+06	14	14	6	2	4	5	Carbamid
1769	3113	sp|Q7L804|RFIP2_HUMAN	175.98	1.08E+06	5.15E+05			7	5	4	4	1	2	Deamidat
928	1946	tr|Q32Q12|Q32Q12_HUMAN	175.59	7.69E+06	8.48E+06	3.32E+06	4.85E+06	11	5	9	9	7	9	Carbamid
885	1812	sp|Q13247|SRSF6_HUMAN	175.52	2.61E+06	6.99E+06	1.54E+07	1.51E+07	13	8	4	9	12	15	Carbamid
1371	2245	sp|P13861|KAP2_HUMAN	175.41	1.86E+06	3.27E+06	1.22E+06	2.47E+05	10	10	6	6	4	1	Carbamid
1541	2413	sp|Q8TDD1|DDX54_HUMAN	175.31	5.44E+05	1.02E+06	4.87E+06	4.50E+06	10	10	4	1	4	8	Carbamid
1029	3358	sp|Q9P0J0|NDUAD_HUMAN	175.15	1.87E+07	3.11E+07	4.26E+07	3.81E+07	9	9	5	7	7	7	
1385	1976	sp|P52701|MSH6_HUMAN	175.12	2.82E+05	1.78E+06	2.24E+05	7.93E+05	15	15	2	6	1	3	
1681	1649	tr|A0A0A0MRM8|A0A0A0MRM8_HUMAN	175.01	2.88E+06	2.78E+05		1.95E+06	18	17	3	1	1	3	Deamidat
1681	1650	sp|Q9UM54|MYO6_HUMAN	175.01	2.88E+06	2.78E+05		1.95E+06	18	17	3	1	1	3	Deamidat
1681	1651	tr|A0A0D9SGC1|A0A0D9SGC1_HUMAN	175.01	2.88E+06	2.78E+05		1.95E+06	18	17	3	1	1	3	Deamidat
1681	1652	tr|E7EW20|E7EW20_HUMAN	175.01	2.88E+06	2.78E+05		1.95E+06	18	17	3	1	1	3	Deamidat
1471	3139	tr|F6WQW2|F6WQW2_HUMAN	174.92	2.27E+07	6.39E+06	1.18E+07	9.11E+06	9	9	6	3	4	4	Carbamid
1074	1679	sp|Q70UQ0|IKIP_HUMAN	174.89	1.23E+07	5.19E+06	9.55E+06	2.06E+07	12	12	9	4	6	9	Deamidat
1537	1728	tr|A0A2R8Y425|A0A2R8Y425_HUMAN	174.81	7.94E+05	1.47E+06	1.66E+06	7.81E+05	20	20	3	3	4	3	Carbamid
1537	1732	tr|A0A2R8Y5J0|A0A2R8Y5J0_HUMAN	174.81	7.94E+05	1.47E+06	1.66E+06	7.81E+05	20	20	3	3	4	3	Carbamid
1537	1733	tr|A0A2R8YFK9|A0A2R8YFK9_HUMAN	174.81	7.94E+05	1.47E+06	1.66E+06	7.81E+05	20	20	3	3	4	3	Carbamid
1537	1734	tr|A0A2U3TZM0|A0A2U3TZM0_HUMAN	174.81	7.94E+05	1.47E+06	1.66E+06	7.81E+05	20	20	3	3	4	3	Carbamid
1537	1735	tr|A0A2R8YFD8|A0A2R8YFD8_HUMAN	174.81	7.94E+05	1.47E+06	1.66E+06	7.81E+05	20	20	3	3	4	3	Carbamid
1537	1736	tr|F5GWX5|F5GWX5_HUMAN	174.81	7.94E+05	1.47E+06	1.66E+06	7.81E+05	20	20	3	3	4	3	Carbamid
1537	1737	sp|Q14839|CHD4_HUMAN	174.81	7.94E+05	1.47E+06	1.66E+06	7.81E+05	20	20	3	3	4	3	Carbamid
1537	1738	tr|A0A2R8Y212|A0A2R8Y212_HUMAN	174.81	7.94E+05	1.47E+06	1.66E+06	7.81E+05	20	20	3	3	4	3	Carbamid
1537	1739	tr|A0A2R8Y521|A0A2R8Y521_HUMAN	174.81	7.94E+05	1.47E+06	1.66E+06	7.81E+05	20	20	3	3	4	3	Carbamid
1537	1740	tr|A0A0C4DGG9|A0A0C4DGG9_HUMAN	174.81	7.94E+05	1.47E+06	1.66E+06	7.81E+05	20	20	3	3	4	3	Carbamid
1148	2155	sp|Q9H9B4|SFXN1_HUMAN	174.47	5.09E+06	4.85E+06	8.97E+05	4.79E+06	11	8	7	10	4	4	Carbamid
1264	1624	sp|Q14149|MORC3_HUMAN	174.45	4.65E+06	6.00E+06	4.64E+06	3.69E+06	18	18	8	7	4	6	Carbamid
1696	2550	sp|P51553|IDH3G_HUMAN	174.34	3.68E+06	2.11E+06	1.28E+06	1.17E+06	13	13	5	2	2	1	Carbamid
1570	1537	sp|Q9UJF2|NGAP_HUMAN	174.25	4.21E+06	5.73E+06	5.55E+06	2.95E+06	15	15	8	7	6	4	Carbamid
1357	1460	sp|Q92974|ARHG2_HUMAN	174.24	1.26E+07	2.16E+07	6.70E+06	1.33E+07	14	14	3	6	4	6	Carbamid
1357	1461	tr|V9GYM8|V9GYM8_HUMAN	174.24	1.26E+07	2.16E+07	6.70E+06	1.33E+07	14	14	3	6	4	6	Carbamid
1992	2829	sp|Q8TB36|GDAP1_HUMAN	174.02	7.21E+05	1.45E+06	7.89E+05	2.46E+06	9	8	2	2	1	2	Carbamid
1449	3136	tr|A0A0C4DGQ8|A0A0C4DGQ8_HUMAN	173.89	2.55E+06	2.26E+06	5.92E+06	7.15E+06	8	8	5	4	5	6	Carbamid
1449	3137	sp|Q6IAN0|DRS7B_HUMAN	173.89	2.55E+06	2.26E+06	5.92E+06	7.15E+06	8	8	5	4	5	6	Carbamid
1623	1880	sp|Q96GM8|TOE1_HUMAN	173.89	4.80E+05	8.52E+05	8.09E+06	5.17E+06	12	12	1	1	5	6	Carbamid
1446	4491	sp|P62633|CNBP_HUMAN	173.88	3.21E+07	1.37E+07	1.22E+07	5.56E+06	6	6	5	5	6	3	Carbamid
1343	1916	sp|Q9UNF1|MAGD2_HUMAN	173.81	4.11E+05	2.22E+06	1.36E+07	8.26E+06	12	12	1	3	7	5	Carbamid
1530	2620	sp|P61225|RAP2B_HUMAN	173.8	2.67E+06	2.04E+06	1.33E+06	1.78E+06	10	4	6	6	2	2	Carbamid
1667	3514	sp|Q86W42|THOC6_HUMAN	173.76	2.47E+06	4.95E+06	2.04E+06	2.12E+06	8	8	5	6	3	5	Carbamid
1587	4242	sp|P0DPB6|RPAC2_HUMAN	173.69	1.59E+06	2.18E+06	2.19E+07	1.63E+07	7	7	4	2	6	7	Carbamid
1559	4345	sp|O95983|MBD3_HUMAN	173.52	8.04E+05	1.80E+06	1.64E+06	8.13E+05	7	7	4	5	2	3	Carbamid
1640	4403	sp|Q9P013|CWC15_HUMAN	173.42	7.98E+06	9.49E+06	1.20E+07	5.01E+06	7	7	4	6	3	3	
1498	1620	sp|Q9P265|DIP2B_HUMAN	173.41	1.48E+06	3.88E+06	2.68E+06	3.97E+06	19	19	4	5	5	5	Carbamid
1026	1696	sp|P06493|CDK1_HUMAN	173.27	1.33E+07	3.34E+06	3.32E+06	5.87E+06	13	11	15	8	4	6	Deamidat
1026	1697	tr|A0A024QZP7|A0A024QZP7_HUMAN	173.27	1.33E+07	3.34E+06	3.32E+06	5.87E+06	13	11	15	8	4	6	Deamidat
1358	2265	sp|P51948|MAT1_HUMAN	173.1	8.80E+06	1.06E+07	5.60E+06	9.53E+06	10	10	4	4	4	7	Carbamid
1159	2118	sp|O43314|VIP2_HUMAN	173.03	4.99E+06	2.12E+06	4.07E+06	1.37E+06	24	24	12	4	8	2	Carbamid
1159	2124	tr|A0A087WZV0|A0A087WZV0_HUMAN	173.03	4.99E+06	2.12E+06	4.07E+06	1.37E+06	24	24	12	4	8	2	Carbamid
1391	2501	tr|K7EMQ3|K7EMQ3_HUMAN	172.82	1.16E+06			4.22E+05	11	2	7	5	2	4	Carbamid
1634	2519	sp|Q8NFW8|NEUA_HUMAN	172.72	8.11E+04	1.98E+05	3.86E+06	5.00E+06	9	9	1	1	5	6	Carbamid
2147	4550	sp|P09211|GSTP1_HUMAN	172.69	3.73E+06	2.82E+06			6	6	3	1	0	0	Carbamid
1661	2960	sp|Q9NPE2|NGRN_HUMAN	172.66	2.00E+06	7.15E+05	7.98E+05	3.62E+06	10	10	3	1	1	6	
1230	1787	sp|Q9H5Q4|TFB2M_HUMAN	172.61	5.22E+06	4.75E+06	6.74E+06	5.44E+06	14	14	7	6	5	4	Carbamid
1558	1999	sp|O00178|GTPB1_HUMAN	172.54	3.72E+05	0	1.08E+07	7.35E+06	11	11	2	1	9	5	Carbamid
732	4357	sp|P09669|COX6C_HUMAN	172.39	1.24E+08	1.67E+08	1.40E+08	1.89E+08	11	11	10	10	9	12	
1455	2573	tr|A0A087X0K9|A0A087X0K9_HUMAN	172.37	3.36E+06	6.47E+06	1.48E+06	5.00E+05	14	13	7	11	2	2	
1455	2574	tr|G5E9E7|G5E9E7_HUMAN	172.37	3.36E+06	6.47E+06	1.48E+06	5.00E+05	14	13	7	11	2	2	
871	1781	sp|Q9Y399|RT02_HUMAN	172.06	1.65E+07	1.74E+07	4.59E+07	4.25E+07	13	13	8	7	10	8	
1784	3857	sp|Q9H3Q1|BORG4_HUMAN	171.76	3.06E+06	3.64E+06	1.75E+06	1.55E+06	6	6	3	4	2	2	
1504	2039	sp|Q15650|TRIP4_HUMAN	171.65	5.53E+06	3.72E+06	7.52E+06	7.41E+06	14	14	5	3	4	6	Carbamid
1676	4796	tr|B4DHE8|B4DHE8_HUMAN	171.43	5.72E+05	1.89E+06	4.29E+06	3.91E+06	5	5	1	3	3	4	
1676	4797	sp|Q96DH6|MSI2H_HUMAN	171.43	5.72E+05	1.89E+06	4.29E+06	3.91E+06	5	5	1	3	3	4	
1429	2816	sp|O75909|CCNK_HUMAN	171.21	1.19E+07	6.99E+06	1.66E+07	9.80E+06	13	13	11	5	7	5	Carbamid
1546	1643	sp|Q96SZ6|CK5P1_HUMAN	171.14	1.23E+06	6.19E+05	3.76E+06	8.78E+05	12	12	3	1	3	2	Carbamid
1755	2580	sp|Q9BZI7|REN3B_HUMAN	171.01	9.39E+06	9.88E+06	1.29E+06	2.02E+06	10	10	6	5	2	3	Carbamid
1563	4945	sp|Q9BQ61|TRIR_HUMAN	170.99	1.78E+07	3.88E+07	1.07E+07	1.06E+07	7	7	5	6	5	3	Carbamid
1405	1842	sp|Q6PML9|ZNT9_HUMAN	170.85	2.38E+06	5.33E+06	1.30E+06	3.30E+06	13	13	2	7	2	6	Carbamid
1688	3072	sp|Q9H4I3|TRABD_HUMAN	170.82	1.62E+06	1.48E+06	4.77E+05	2.88E+06	8	8	3	3	1	3	Carbamid
1688	3073	tr|J3KPT4|J3KPT4_HUMAN	170.82	1.62E+06	1.48E+06	4.77E+05	2.88E+06	8	8	3	3	1	3	Carbamid
1524	2921	sp|O43818|U3IP2_HUMAN	170.61	2.05E+05	1.12E+06	9.89E+06	1.70E+07	10	10	1	3	6	7	Carbamid
2076	3754	sp|P45954|ACDSB_HUMAN	170.4	1.39E+06	3.99E+06	1.66E+06	3.63E+06	7	7	1	2	2	2	Carbamid
1564	3190	sp|Q9Y6M9|NDUB9_HUMAN	170.33	8.51E+06	1.21E+07	9.41E+06	4.83E+06	8	8	2	5	6	2	Carbamid
1760	5272	sp|Q567V2|M17L2_HUMAN	170.31	1.47E+06	3.09E+06	0	9.82E+06	5	5	2	5	1	5	Carbamid
451	2318	tr|A0A024R4M0|A0A024R4M0_HUMAN	170.03	5.71E+07	9.12E+07	6.83E+08	6.48E+08	16	16	12	12	19	18	Deamidat
451	2319	sp|P46781|RS9_HUMAN	170.03	5.71E+07	9.12E+07	6.83E+08	6.48E+08	16	16	12	12	19	18	Deamidat
1014	1704	sp|P49406|RM19_HUMAN	169.81	3.88E+06	6.07E+06	1.56E+07	1.18E+07	13	13	6	7	10	6	Carbamid
1505	2414	sp|P51114|FXR1_HUMAN	169.47		2.02E+06	3.05E+06	3.79E+06	11	7	0	2	6	6	Carbamid
1505	2462	tr|E9PFF5|E9PFF5_HUMAN	169.47		2.02E+06	3.05E+06	2.03E+06	10	6	0	2	6	5	Carbamid
1549	2074	sp|P49756|RBM25_HUMAN	169.42	1.10E+07	8.07E+06	4.51E+05	1.19E+06	14	14	10	8	2	2	Carbamid
1103	5359	sp|O14561|ACPM_HUMAN	169.22	7.13E+07	7.13E+07	6.30E+07	6.46E+07	8	8	5	6	7	6	Carbamid
1552	2929	sp|Q15119|PDK2_HUMAN	169.1	1.96E+06	8.50E+05		1.20E+06	8	6	6	4	2	4	Carbamid
1822	2300	sp|Q96H55|MYO19_HUMAN	169.06	1.46E+07	1.70E+06	9.61E+06	6.15E+06	11	11	2	4	1	4	Carbamid
1624	2571	sp|Q9NP72|RAB18_HUMAN	168.96	2.50E+06	1.87E+06	2.11E+06	0	10	10	3	2	2	2	Carbamid
1173	2410	sp|P54709|AT1B3_HUMAN	168.88	1.81E+07	3.72E+06	5.94E+06	6.73E+06	9	9	8	4	4	4	Deamidat
993	5050	sp|P62805|H4_HUMAN	168.85	8.63E+07	1.33E+08	2.42E+08	3.31E+08	8	8	8	7	6	12	Oxidation
1361	1979	sp|O43242|PSMD3_HUMAN	168.65	2.78E+06	2.68E+06	2.28E+06	1.58E+06	12	11	6	6	5	4	
1388	1909	sp|Q6PKG0|LARP1_HUMAN	168.6	2.27E+05	1.72E+06	4.22E+06	1.00E+07	15	11	4	6	8	8	Carbamid
1315	3532	sp|Q9NX70|MED29_HUMAN	168.56	1.11E+07	2.11E+07	8.96E+06	1.51E+07	7	7	7	9	4	6	Carbamid
1315	3534	tr|B4DUA7|B4DUA7_HUMAN	168.56	1.11E+07	2.11E+07	8.96E+06	1.51E+07	7	7	7	9	4	6	Carbamid
1526	2238	sp|O75648|MTU1_HUMAN	168.05	3.37E+06	2.13E+07	1.58E+06	2.11E+06	10	10	5	8	3	2	Carbamid
1785	4215	sp|Q9NP92|RT30_HUMAN	168.01	1.23E+06	1.56E+06	7.32E+06	3.47E+06	6	6	3	3	6	4	Carbamid
1821	3151	sp|P18583|SON_HUMAN	167.97	2.12E+06	1.16E+06	5.08E+06	7.85E+05	7	7	3	3	6	2	Acetylatic
1599	4635	tr|H0Y9X1|H0Y9X1_HUMAN	167.96	1.08E+07	1.07E+07	4.82E+06	9.43E+06	5	5	4	4	2	3	Carbamid
1599	4682	sp|Q96EY4|TMA16_HUMAN	167.96	1.08E+07	1.07E+07	4.82E+06	9.43E+06	5	5	4	4	2	3	Carbamid
1104	2793	sp|Q9NSI2|F207A_HUMAN	167.89	2.95E+07	4.11E+07	3.43E+07	4.74E+07	10	10	5	8	5	8	

1138	1784	tr|F2Z2I2|F2Z2I2_HUMAN	167.79	4.19E+06		2.38E+06	2.43E+06	15	10	11	3	9	7	Carbamid
1138	1804	tr|Q5W015|Q5W015_HUMAN	167.79	4.19E+06		2.38E+06	2.43E+06	15	10	11	3	9	7	Carbamid
1138	1805	sp|Q16875|F263_HUMAN	167.79	4.19E+06		2.38E+06	2.43E+06	15	10	11	3	9	7	Carbamid
1138	1806	tr|Q5VX20|Q5VX20_HUMAN	167.79	4.19E+06		2.38E+06	2.43E+06	15	10	11	3	9	7	Carbamid
1138	1813	tr|A0A1W2PR17|A0A1W2PR17_HUMAN	167.79	4.19E+06		2.38E+06	2.43E+06	15	10	11	3	9	7	Carbamid
1543	4464	sp|Q96HR3|MED30_HUMAN	167.67	3.77E+07	1.64E+07	5.42E+06	1.21E+07	7	7	5	5	3	5	Acetylatio
1217	3504	sp|P09382|LEG1_HUMAN	167.54	3.10E+07	2.38E+07	1.61E+07	1.24E+07	7	7	7	5	6	5	Carbamid
1512	2511	sp|Q8IX18|DHX40_HUMAN	167.53	1.51E+06	6.61E+06	4.31E+06	3.35E+06	11	10	3	7	4	7	Carbamid
1426	5991	sp|P62854|RS26_HUMAN	167.25	1.46E+07	1.12E+07	1.52E+08	1.59E+08	6	6	3	2	7	7	Carbamid
1139	2086	sp|Q9NZN3|EHD3_HUMAN	167.24	3.54E+06	2.22E+06			12	1	10	5	7	3	
1776	6311	sp|Q8IY37|DHX37_HUMAN	167.16	4.60E+07	3.16E+07	2.64E+07	2.75E+07	6	6	5	4	2	6	
1595	2320	sp|Q8IWT6|LRC8A_HUMAN	167.1			4.48E+06	5.35E+06	9	9	0	0	5	7	Carbamid
1457	2269	sp|Q9Y5S2|MRCKB_HUMAN	167.02	1.07E+06	2.51E+06	2.24E+06	4.21E+05	20	20	5	7	4	2	Carbamid
1276	1892	sp|P38606|VATA_HUMAN	166.78	7.18E+06	4.37E+06	1.93E+06	4.32E+06	13	13	2	5	3	6	Carbamid
1889	5056	sp|Q9BPX5|ARP5L_HUMAN	166.78	6.18E+05	5.77E+06	4.32E+05	6.08E+06	6	4	1	4	2	4	Deamidat
958	4261	tr|E7EPB3|E7EPB3_HUMAN	166.43	4.73E+07	1.28E+08	9.93E+08	7.97E+08	8	8	6	5	9	10	Carbamid
958	4270	sp|P50914|RL14_HUMAN	166.43	4.73E+07	1.28E+08	9.93E+08	7.97E+08	8	8	6	5	9	10	Carbamid
1057	1906	sp|P46977|STT3A_HUMAN	166.36	5.99E+06	8.97E+06	5.67E+06	1.27E+07	12	11	6	10	4	8	Carbamid
1842	5694	sp|Q8N488|RYBP_HUMAN	166.22	2.96E+06	1.22E+07	1.32E+06	7.17E+06	4	4	2	5	1	3	Carbamid
1353	1970	sp|Q9ULW0|TPX2_HUMAN	166.12	1.22E+07	6.77E+06	4.89E+06	4.95E+06	16	15	9	10	7	6	Carbamid
1734	2528	sp|Q9UKM7|MA1B1_HUMAN	165.98	3.08E+05	3.84E+05	2.30E+06	2.97E+06	9	9	1	1	4	4	
2035	4465	sp|Q03701|CEBPZ_HUMAN	165.95	2.47E+06	5.60E+06		4.32E+06	5	5	2	4	0	5	Oxidation
1114	2173	sp|P15311|EZRI_HUMAN	165.81			5.33E+05	1.76E+06	12	4	6	6	7	8	Carbamid
1114	2174	tr|E7EQR4|E7EQR4_HUMAN	165.81			5.33E+05	1.76E+06	12	4	6	6	7	8	Carbamid
1467	2996	sp|P51946|CCNH_HUMAN	165.67	4.21E+06	5.89E+06	6.52E+06	4.18E+06	11	11	5	6	6	3	Carbamid
1467	2997	tr|A0A2R8YEM2|A0A2R8YEM2_HUMAN	165.67	4.21E+06	5.89E+06	6.52E+06	4.18E+06	11	11	5	6	6	3	Carbamid
1479	2643	sp|Q9NPL8|TIDC1_HUMAN	165.57	3.31E+06	2.65E+06	0	3.21E+06	10	10	6	2	1	3	
1296	2207	sp|P57772|SELB_HUMAN	165.54	4.06E+06	1.60E+06	6.45E+06	7.64E+06	13	13	5	2	7	6	Carbamid
1646	2305	sp|Q12788|TBL3_HUMAN	165.3		2.71E+05	5.77E+06	6.63E+06	11	11	0	1	5	7	Carbamid
1647	3165	sp|Q9UL25|RAB21_HUMAN	165.28	1.65E+06	1.59E+06	1.25E+06	2.34E+07	7	7	3	4	2	3	Carbamid
1162	1943	sp|P30260|CDC27_HUMAN	165.18			1.15E+06		16	1	5	9	7	7	Carbamid
1550	2441	sp|Q13131|AAPK1_HUMAN	165.17	2.60E+06	1.36E+06	4.69E+06	9.24E+05	9	9	4	3	5	2	Carbamid
1072	1386	sp|Q6PD62|CTR9_HUMAN	165.08	8.01E+06	9.78E+06	1.23E+07	6.15E+06	17	17	11	10	10	5	Carbamid
1863	2204	tr|F8VQ14|F8VQ14_HUMAN	165.03	3.28E+06	2.93E+06	2.11E+06	3.45E+06	13	13	6	3	1	2	Acetylatio
1863	2205	tr|F5GWF6|F5GWF6_HUMAN	165.03	3.28E+06	2.93E+06	2.11E+06	3.45E+06	13	13	6	3	1	2	Acetylatio
1863	2206	sp|P78371|TCPB_HUMAN	165.03	3.28E+06	2.93E+06	2.11E+06	3.45E+06	13	13	6	3	1	2	Acetylatio
1487	6029	sp|P63167|DYL1_HUMAN	164.79	1.45E+07	8.76E+06	1.52E+07	1.79E+07	5	2	5	3	5	5	Carbamid
1378	4144	tr|Q5T8T7|Q5T8T7_HUMAN	164.78	1.46E+07	2.12E+07	1.54E+07	1.60E+07	6	6	5	5	5	5	
1378	4145	tr|E7EN96|E7EN96_HUMAN	164.78	1.46E+07	2.12E+07	1.54E+07	1.60E+07	6	6	5	5	5	5	
1378	4146	tr|Q5T8T8|Q5T8T8_HUMAN	164.78	1.46E+07	2.12E+07	1.54E+07	1.60E+07	6	6	5	5	5	5	
1378	4148	sp|Q15528|MED22_HUMAN	164.78	1.46E+07	2.12E+07	1.54E+07	1.60E+07	6	6	5	5	5	5	
1378	4289	tr|E9PGW7|E9PGW7_HUMAN	164.78	1.46E+07	2.12E+07	1.54E+07	1.60E+07	6	6	5	5	5	5	
1818	2669	sp|O00443|P3C2A_HUMAN	164.44	5.04E+06	2.24E+06	6.29E+06	1.49E+06	10	10	5	3	5	2	
1758	5796	sp|Q8IY57|YAF2_HUMAN	164.34	7.01E+06	6.96E+06	4.09E+06	7.73E+06	4	4	3	5	4	2	Carbamid
1787	4762	tr|Q86WV7|Q86WV7_HUMAN	164.25	2.19E+06	6.82E+05		1.14E+06	7	6	3	6	0	2	Acetylatio
1787	5349	sp|Q96MW1|CCD43_HUMAN	164.25	2.19E+06	6.82E+05		1.14E+06	6	6	3	5	0	2	Acetylatio
1150	2394	sp|Q96A35|RM24_HUMAN	164.12	5.11E+06	3.80E+06	4.00E+07	2.48E+07	10	10	5	4	12	7	Carbamid
1127	3747	sp|O15145|ARPC3_HUMAN	163.84	4.05E+06	1.94E+07	1.19E+07	2.16E+07	8	8	4	8	5	6	
1591	4083	sp|Q13405|RM49_HUMAN	163.77	5.44E+06	6.96E+06	9.76E+06	1.37E+07	7	7	4	4	3	4	
1560	4933	sp|P82912|RT11_HUMAN	163.75	6.91E+06	9.88E+06	1.66E+07	2.26E+07	6	6	5	5	3	4	Carbamid
1113	1984	tr|G5EA36|G5EA36_HUMAN	163.72	3.65E+05		4.28E+05	3.19E+05	16	1	6	9	7	8	Carbamid
1988	4598	sp|Q9BX40|LS14B_HUMAN	163.63	2.95E+05		3.82E+06	3.16E+06	7	7	3	0	4	3	Carbamid
1374	4651	tr|C9JFR7|C9JFR7_HUMAN	163.54	2.58E+07	2.20E+07	1.72E+07	2.04E+07	7	7	6	6	4	4	
1374	4652	sp|P99999|CYC_HUMAN	163.54	2.58E+07	2.20E+07	1.72E+07	2.04E+07	7	7	6	6	4	4	
1511	1969	sp|P12931|SRC_HUMAN	163.5	1.25E+06	2.62E+06		1.31E+06	14	9	6	8	2	3	Carbamid
1602	3251	sp|P25685|DNJB1_HUMAN	163.39	7.61E+06	2.00E+06	2.12E+06	2.36E+06	8	8	8	2	3	3	Carbamid
1367	2405	sp|Q8N2K0|ABD12_HUMAN	163.38	4.32E+06	5.89E+06	6.53E+06	5.82E+06	10	10	6	7	3	4	Carbamid
1277	4526	sp|P53680|AP2S1_HUMAN	163.37	6.12E+06	5.26E+06	2.91E+07	2.67E+07	6	6	4	4	6	6	
1277	4528	tr|M0R0N4|M0R0N4_HUMAN	163.37	6.12E+06	5.26E+06	2.91E+07	2.67E+07	6	6	4	4	6	6	
1277	4531	tr|M0QYZ2|M0QYZ2_HUMAN	163.37	6.12E+06	5.26E+06	2.91E+07	2.67E+07	6	6	4	4	6	6	
2468	5034	sp|Q9NZT1|CALL5_HUMAN	163.32					5	5	0	0	0	0	Acetylatio
1611	5793	sp|Q96EL3|RM53_HUMAN	163.27	4.47E+06	5.62E+06	1.37E+07	1.54E+07	4	4	4	3	3	3	Carbamid
1766	2114	sp|P61163|ACTZ_HUMAN	163.2	1.74E+06	3.24E+06	2.17E+06	2.05E+06	12	11	2	5	1	3	Carbamid
1766	2190	tr|R4GMT0|R4GMT0_HUMAN	163.2	1.74E+06	3.24E+06	2.17E+06	2.05E+06	11	10	2	5	1	3	Carbamid
1496	1875	sp|Q7RTP6|MICA3_HUMAN	163.19	1.09E+07	4.27E+06	4.89E+06	7.63E+05	14	14	9	8	4	1	Carbamid
920	3876	tr|D3YTB1|D3YTB1_HUMAN	163.12	8.41E+07	9.33E+07	7.25E+08	8.40E+08	8	8	6	6	8	10	Carbamid
920	3881	sp|P62910|RL32_HUMAN	163.12	8.41E+07	9.33E+07	7.25E+08	8.40E+08	8	8	6	6	8	10	Carbamid
920	3885	tr|F8W727|F8W727_HUMAN	163.12	8.41E+07	9.33E+07	7.25E+08	8.40E+08	8	8	6	6	8	10	Carbamid
1509	2131	tr|A0A1B0GVY1|A0A1B0GVY1_HUMAN	163.07	1.00E+05	4.27E+05	4.29E+05		16	14	2	5	3	0	Deamidat
1414	4868	sp|Q9Y5L4|TIM13_HUMAN	162.5	3.27E+07	2.73E+07	2.08E+07	2.84E+07	5	5	5	3	5	4	Carbamid
1131	4814	sp|O43181|NDUS4_HUMAN	162.37	2.88E+07	2.06E+07	1.64E+07	1.39E+07	5	5	7	6	6	4	Deamidat
1568	2376	sp|A6NHR9|SMHD1_HUMAN	162			1.86E+06	3.84E+06	17	17	0	0	5	8	Carbamid
1692	3988	sp|P51149|RAB7A_HUMAN	161.96	1.26E+06	1.31E+06	1.36E+06	1.25E+06	6	6	3	3	3	3	Carbamid
977	7340	sp|P63173|RL38_HUMAN	161.93	7.92E+07	1.42E+08	1.06E+08	1.72E+08	7	7	9	9	7	8	
1937	3789	sp|O95159|ZFPL1_HUMAN	161.81	4.15E+06	1.88E+06	3.20E+06	9.30E+05	8	8	3	2	3	1	Carbamid
1786	4641	sp|Q8TAT6|NPL4_HUMAN	161.77	1.50E+06	4.06E+05	5.19E+05	3.97E+05	6	6	6	1	2	1	Carbamid
1525	5559	sp|Q16629|SRSF7_HUMAN	161.76	6.27E+05	1.39E+06	9.34E+05	5.21E+05	9	5	2	4	4	6	Deamidat
1248	3441	sp|O95182|NDUA7_HUMAN	161.57	1.45E+07	9.72E+06	2.61E+06	7.75E+06	11	11	5	5	2	5	Carbamid
1179	2650	sp|Q08170|SRSF4_HUMAN	161.48	3.18E+06	2.02E+06	2.49E+06	1.63E+06	9	4	4	6	8	9	Carbamid
1639	3548	sp|Q12931|TRAP1_HUMAN	161.27	6.71E+05	5.88E+05	3.83E+05	2.88E+05	7	6	4	4	2	2	
1870	2165	sp|Q92793|CBP_HUMAN	161.24					11	3	1	0	0	1	Carbamid
1701	5574	tr|A0A0A6YYL4|A0A0A6YYL4_HUMAN	161.14	1.12E+07	1.22E+07	8.29E+06	2.23E+07	5	5	3	3	2	5	
1701	5600	sp|Q9Y3D7|TIM16_HUMAN	161.14	1.12E+07	1.22E+07	8.29E+06	2.23E+07	5	5	3	3	2	5	
1701	5602	tr|I3L0X9|I3L0X9_HUMAN	161.14	1.12E+07	1.22E+07	8.29E+06	2.23E+07	5	5	3	3	2	5	
1701	5605	tr|I3L3T0|I3L3T0_HUMAN	161.14	1.12E+07	1.22E+07	8.29E+06	2.23E+07	5	5	3	3	2	5	
1701	6360	tr|I3L1U7|I3L1U7_HUMAN	161.14	1.12E+07	1.22E+07	8.29E+06	2.23E+07	5	5	3	3	2	5	
1780	2180	sp|Q92900|RENT1_HUMAN	161.13	1.16E+05	3.20E+05	3.26E+06	1.13E+06	12	12	1	2	6	6	Carbamid
1161	1944	sp|Q99661|KIF2C_HUMAN	161.03	3.14E+06	1.74E+06	7.57E+06	4.92E+06	11	9	7	4	8	4	Carbamid
1686	2399	sp|P46060|RAGP1_HUMAN	161.03	1.70E+06		8.45E+05	1.67E+06	11	11	3	0	1	2	Carbamid
1268	2182	sp|P52789|HXK2_HUMAN	161	1.31E+06	1.67E+06	2.77E+06	1.59E+06	13	7	6	9	4	7	Carbamid
1268	2186	tr|E9PB90|E9PB90_HUMAN	161	1.31E+06	1.67E+06	2.77E+06	1.59E+06	13	7	6	9	4	7	Carbamid
2000	2535	sp|P43304|GPDM_HUMAN	160.86	7.19E+06	6.52E+06	2.93E+05		9	9	3	4	1	0	Carbamid
1404	5159	tr|J3QL05|J3QL05_HUMAN	160.84	6.49E+06	3.96E+07	5.25E+07	1.14E+08	5	5	5	5	4	4	Acetylatio
1404	5161	tr|J3KP15|J3KP15_HUMAN	160.84	6.49E+06	3.96E+07	5.25E+07	1.14E+08	5	5	5	5	4	4	Acetylatio
1404	5186	sp|Q01130|SRSF2_HUMAN	160.84	6.49E+06	3.96E+07	5.25E+07	1.14E+08	5	5	5	5	4	4	Acetylatio
1260	3138	sp|O43432|IF4G3_HUMAN	160.75	8.98E+03				9	2	8	6	4	5	Carbamid
1260	3143	tr|A0A0A0MSA7|A0A0A0MSA7_HUMAN	160.75	8.98E+03				9	2	8	6	4	5	Carbamid
1260	3157	tr|A0A0U1RQK7|A0A0U1RQK7_HUMAN	160.75	8.98E+03				9	2	8	6	4	5	Carbamid
1898	6025	sp|Q8WV22|NSE1_HUMAN	160.73	1.37E+06		6.14E+06	3.70E+06	4	4	2	0	3	3	
2089	4208	sp|P29372|3MG_HUMAN	160.55			1.88E+05	3.10E+06	6	6	0	0	1	2	
1638	2359	tr|A0A1W2PPI7|A0A1W2PPI7_HUMAN	160.51					10	1	3	2	5	3	Carbamid
1241	7531	sp|P60866|RS20_HUMAN	160.49	2.36E+08	1.87E+08	3.93E+08	3.93E+08	7	7	6	3	5	4	Carbamid
1569	2070	sp|Q7Z6E9|RBBP6_HUMAN	160.49	1.56E+07	9.42E+06	7.60E+06	7.44E+06	13	13	9	6	4	4	
1836	2846	sp|P78362|SRPK2_HUMAN	160.41			5.46E+06	5.07E+06	9	7	0	0	8	4	Carbamid
1143	2037	sp|Q96BJ3|AIDA_HUMAN	160.24	1.79E+07	8.86E+06	5.49E+06	2.97E+06	10	10	13	8	3	3	Carbamid
1885	4122	sp|Q99961|SH3G1_HUMAN	160.1	3.59E+06	3.13E+06	1.12E+06	0	6	6	4	2	1	1	Carbamid
1601	3154	sp|Q92947|GCDH_HUMAN	160.08	1.94E+06	2.26E+06	3.36E+06	2.87E+06	8	8	2	3	4	4	Carbamid
1482	3964	sp|Q9BUE0|MED18_HUMAN	159.98	1.52E+07	2.06E+07	1.23E+07	1.78E+07	6	6	5	5	3	4	Carbamid
2436	5291	sp|Q9BRU9|UTP23_HUMAN	159.94	1.41E+06	2.47E+05	8.89E+06	3.13E+06	5	5	1	1	4	2	Carbamid
1499	2368	sp|Q04695|K1C17_HUMAN	159.9					15	2	7	4	3	1	
1635	2986	sp|Q99717|SMAD5_HUMAN	159.87	3.51E+06		1.61E+06	3.07E+06	9	7	7	2	3	4	Carbamid
1312	2493	sp|Q13637|RAB32_HUMAN	159.35	1.85E+06	0	4.89E+06	4.16E+06	9	7	5	3	6	4	Deamidat
1768	2722	sp|Q9Y5B9|SP16H_HUMAN	159.23	4.15E+05	2.12E+06	4.74E+05	1.13E+06	10	10	2	6	1	2	Carbamid
1875	4421	sp|Q8TEA8|DTD1_HUMAN	159.18	1.60E+07	3.25E+06	2.11E+06	3.08E+05	7	7	5	1	1	1	Carbamid
1590	3122	sp|P54619|AAKG1_HUMAN	159.15	3.42E+06	1.58E+06	3.37E+06	1.45E+06	8	8	4	3	4	1	Deamidat
1663	5365	sp|P09234|RU1C_HUMAN	159.11	2.04E+08	1.66E+08	1.40E+08	2.06E+08	5	5	5	3	4	4	Carbamid

1663	5367	tr|A0A0A0MRR7|A0A0A0MRR7_HUMAN	159.11	2.04E+08	1.66E+08	1.40E+08	2.06E+08	5	5	5	3	4	4	Carbamid
1666	2156	sp|Q9UNM6|PSD13_HUMAN	158.92	1.38E+06	1.12E+06		3.39E+05	10	10	4	3	0	3	Carbamid
1887	3621	sp|Q96GM5|SMRD1_HUMAN	158.82	8.38E+05	2.91E+05	8.56E+05	2.91E+05	8	7	4	1	3	3	Carbamid
1577	3762	sp|Q9HA38|ZMAT3_HUMAN	158.72	3.24E+06	2.89E+06	2.53E+06	6.58E+06	6	6	5	2	2	5	Carbamid
1483	5624	sp|Q7Z7F7|RM55_HUMAN	158.56	1.85E+07	4.03E+07	4.03E+07	4.44E+07	5	5	3	4	4	6	
1789	4141	sp|Q96I51|RCC1L_HUMAN	158.48	2.55E+06	3.49E+06	3.87E+06	1.51E+06	6	6	3	5	2	1	Carbamid
1320	6123	tr|E9PLL6|E9PLL6_HUMAN	158.39	7.89E+07	8.20E+07	2.77E+08	2.82E+08	6	6	6	5	4	4	Carbamid
1320	6127	sp|P46776|RL27A_HUMAN	158.39	7.89E+07	8.20E+07	2.77E+08	2.82E+08	6	6	6	5	4	4	Carbamid
1788	2659	sp|Q96B26|EXOS8_HUMAN	158.35		4.11E+05	1.94E+07	2.78E+07	9	9	0	1	5	6	Carbamid
1271	2004	sp|P78345|RPP38_HUMAN	158.23	4.45E+06	7.31E+06	9.42E+06	2.10E+07	11	11	4	5	5	9	Carbamid
1304	2667	sp|Q9NYK5|RM39_HUMAN	158.22	9.37E+05	3.08E+06	9.01E+06	8.84E+06	10	10	3	6	6	9	Carbamid
1304	2697	tr|C9JG87|C9JG87_HUMAN	158.22	9.37E+05	3.08E+06	9.01E+06	8.84E+06	10	10	3	6	6	9	Carbamid
1728	3389	sp|P31948|STIP1_HUMAN	158.18	1.10E+07	9.62E+05	2.46E+06	4.66E+05	8	8	7	2	3	2	Carbamid
2169	4015	tr|H3BQN4|H3BQN4_HUMAN	158.15	5.13E+06	2.37E+06	1.73E+05		7	7	5	2	1	0	
2169	4016	sp|P04075|ALDOA_HUMAN	158.15	5.13E+06	2.37E+06	1.73E+05		7	7	5	2	1	0	
2169	4017	tr|J3KPS3|J3KPS3_HUMAN	158.15	5.13E+06	2.37E+06	1.73E+05		7	7	5	2	1	0	
1725	1572	tr|F8WAN1|F8WAN1_HUMAN	158.11	4.90E+04	9.40E+05		3.43E+05	14	13	1	2	0	2	Carbamid
1725	1586	sp|Q69YQ0|CYTSA_HUMAN	158.11	4.90E+04	9.40E+05		3.43E+05	14	13	1	2	0	2	Carbamid
1685	3156	sp|Q6P087|RUSD3_HUMAN	158.06	4.65E+05	1.94E+06	3.22E+06	1.04E+07	10	10	1	2	2	6	Carbamid
2095	4691	sp|P31942|HNRH3_HUMAN	158.01	4.55E+06	9.31E+05			5	4	4	3	0	1	Carbamid
1222	1790	sp|Q9NV70|EXOC1_HUMAN	157.69	4.30E+06	5.70E+06	5.50E+06	3.82E+07	18	18	6	7	7	3	Carbamid
1750	4658	sp|P60604|UB2G2_HUMAN	157.44	5.28E+05	5.56E+05	3.40E+06	3.06E+06	5	5	2	4	2	5	Carbamid
1209	2984	sp|P61020|RAB5B_HUMAN	157.38	9.20E+05	5.14E+05	9.82E+05	1.08E+06	9	4	8	9	3	4	Deamidat
1234	2044	tr|X6R7X0|X6R7X0_HUMAN	157.37	2.63E+07	2.90E+07	1.97E+07	2.16E+07	11	11	10	9	8	7	
1234	2069	sp|Q96MN5|TEAN2_HUMAN	157.37	2.63E+07	2.90E+07	1.97E+07	2.16E+07	11	11	10	9	8	7	
1475	2179	sp|P54886|P5CS_HUMAN	157.36	3.91E+06	1.46E+06	1.97E+08	1.36E+08	14	14	4	4	2	3	Carbamid
1930	3627	sp|P23381|SYWC_HUMAN	157.28	1.70E+06	4.75E+05	7.08E+05	6.06E+05	6	6	4	1	1	1	Carbamid
1947	5434	sp|Q13526|PIN1_HUMAN	157.26	6.73E+06	2.07E+06	3.15E+06	2.11E+06	5	5	4	2	3	2	Carbamid
2055	3482	sp|P10515|ODP2_HUMAN	157.08	9.61E+05	3.57E+06	6.14E+06	1.13E+05	8	8	3	2	7	1	Carbamid
1710	3601	sp|Q9H6H4|REEP4_HUMAN	157.07	0		1.13E+07	1.08E+07	7	6	1	0	4	3	
1596	2479	sp|P62333|PRS10_HUMAN	157.01	2.10E+06	1.48E+06	3.03E+05	1.05E+06	12	12	5	3	1	3	Carbamid
1596	2481	tr|A0A087X2I1|A0A087X2I1_HUMAN	157.01	2.10E+06	1.48E+06	3.03E+05	1.05E+06	12	12	5	3	1	3	Carbamid
1575	2428	sp|Q9NYF8|BCLF1_HUMAN	156.74	7.64E+06	6.18E+06	2.45E+06	1.05E+06	11	10	7	6	5	2	
1438	2016	sp|Q13356|PPIL2_HUMAN	156.54	2.74E+06	8.18E+06	3.09E+06	6.89E+06	11	11	4	8	2	6	Carbamid
1778	3359	sp|Q86XZ4|SPAS2_HUMAN	156.5	2.55E+06	4.92E+06	8.43E+06	2.62E+06	8	8	4	7	5	2	Carbamid
1817	2191	sp|Q09472|EP300_HUMAN	156.5			5.52E+06		12	4	1	0	1	1	Carbamid
1944	4409	sp|Q5T653|RM02_HUMAN	156.37	3.16E+05	4.10E+05	8.61E+06	4.84E+06	6	6	1	1	4	3	Carbamid
1431	2100	sp|P50281|MMP14_HUMAN	156.37	4.87E+06	2.65E+06	1.46E+06	4.52E+05	12	12	5	6	4	3	Carbamid
1918	3395	sp|Q13428|TCOF_HUMAN	156.22	7.65E+06	6.39E+06	1.05E+07	1.92E+07	8	8	2	2	2	5	
1918	3396	tr|E7ETY2|E7ETY2_HUMAN	156.22	7.65E+06	6.39E+06	1.05E+07	1.92E+07	8	8	2	2	2	5	
1918	3397	tr|J3KQ96|J3KQ96_HUMAN	156.22	7.65E+06	6.39E+06	1.05E+07	1.92E+07	8	8	2	2	2	5	
1918	3477	tr|A0A3B3IS06|A0A3B3IS06_HUMAN	156.22	7.65E+06	6.39E+06	1.05E+07	1.92E+07	8	8	2	2	2	5	
1164	1899	sp|P04899|GNAI2_HUMAN	156.08	3.81E+05	7.36E+04		6.21E+05	11	5	8	5	6	5	Carbamid
1172	6947	sp|Q9NPE3|NOP10_HUMAN	155.89	3.95E+07	3.62E+07	4.77E+07	4.53E+07	5	5	12	7	7	8	Carbamid
1906	7808	sp|Q8WY22|BRI3B_HUMAN	155.77	7.04E+05		1.67E+07	2.30E+07	3	3	2	0	4	3	Deamidat
1847	3807	sp|Q93096|TP4A1_HUMAN	155.55	5.90E+06	4.83E+06	1.89E+06	1.51E+06	6	6	5	4	2	1	Deamidat
1700	4632	sp|P19387|RPB3_HUMAN	155.53	1.73E+06	1.21E+06	4.66E+06	1.77E+06	5	5	4	3	3	3	
894	4518	tr|C9JNW5|C9JNW5_HUMAN	155.38	3.10E+08	1.72E+08	4.80E+08	4.06E+08	8	8	11	7	11	10	Carbamid
894	4681	sp|P83731|RL24_HUMAN	155.38	3.10E+08	1.72E+08	4.80E+08	4.06E+08	8	8	11	7	11	10	Carbamid
894	4735	tr|C9JXB8|C9JXB8_HUMAN	155.38	3.10E+08	1.72E+08	4.80E+08	4.06E+08	8	8	11	7	11	10	Carbamid
2097	4979	sp|Q9P086|MED11_HUMAN	155.3	8.08E+06	1.55E+07	2.82E+06		5	4	3	5	1	0	Acetylatic
1263	1883	sp|Q08945|SSRP1_HUMAN	155.29	2.44E+06	5.92E+06	1.19E+06	1.90E+06	16	16	5	10	4	4	Carbamid
1493	4184	sp|P35244|RFA3_HUMAN	155.27	2.44E+06	7.88E+06	2.34E+06	7.05E+06	6	6	3	6	2	4	Carbamid
1397	2076	sp|Q9BZG1|RAB34_HUMAN	155.26	2.42E+06	2.50E+06	1.43E+06	2.88E+06	10	9	5	6	4	4	Carbamid
1763	3760	sp|Q9NWU2|GID8_HUMAN	155.02	1.70E+06	4.36E+06	2.11E+06	1.37E+06	7	7	2	5	3	2	Carbamid
1816	3381	sp|P15924|DESP_HUMAN	154.89	1.42E+05				13	13	1	0	0	0	
1914	7310	sp|P60002|ELOF1_HUMAN	154.88	1.86E+07	1.17E+07	1.21E+07	1.41E+07	3	3	3	3	2	2	Carbamid
1914	7311	tr|K7EPC8|K7EPC8_HUMAN	154.88	1.86E+07	1.17E+07	1.21E+07	1.41E+07	3	3	3	3	2	2	Carbamid
1914	7312	tr|A0A024R7E8|A0A024R7E8_HUMAN	154.88	1.86E+07	1.17E+07	1.21E+07	1.41E+07	3	3	3	3	2	2	Carbamid
1645	4763	sp|P45973|CBX5_HUMAN	154.8	3.22E+06	3.91E+06	7.20E+05	4.32E+05	5	4	5	5	2	3	Carbamid
1344	3398	tr|A0A024R7W5|A0A024R7W5_HUMAN	154.73	2.93E+05				7	2	6	3	5	2	
1344	3401	tr|A0A087X0Q1|A0A087X0Q1_HUMAN	154.73	2.93E+05				7	2	6	3	5	2	
1344	3403	sp|Q7Z739|YTHD3_HUMAN	154.73	2.93E+05				7	2	6	3	5	2	
1344	3404	tr|A0A087WY31|A0A087WY31_HUMAN	154.73	2.93E+05				7	2	6	3	5	2	
1943	3382	sp|Q2TAY7|SMU1_HUMAN	154.67	2.15E+06	5.33E+06	1.74E+06	8.05E+05	7	7	4	7	2	2	Carbamid
1305	2971	tr|A0A3B3IRL5|A0A3B3IRL5_HUMAN	154.67	3.95E+06	6.10E+04	9.83E+05	3.98E+05	9	6	6	5	6	5	Carbamid
1305	2972	tr|C9J381|C9J381_HUMAN	154.67	3.95E+06	6.10E+04	9.83E+05	3.98E+05	9	6	6	5	6	5	Carbamid
1305	2973	sp|P20839|IMDH1_HUMAN	154.67	3.95E+06	6.10E+04	9.83E+05	3.98E+05	9	6	6	5	6	5	Carbamid
1249	2783	sp|Q8TED1|GPX8_HUMAN	154.49	1.09E+07	9.10E+06	7.31E+06	7.99E+06	9	9	8	7	5	8	Carbamid
1401	2987	sp|Q96L58|B3GT6_HUMAN	154.31	3.15E+04	1.39E+06	1.18E+07	1.20E+07	8	8	1	3	8	6	Carbamid
588	10003	sp|P62861|RS30_HUMAN	154.29	1.36E+09	1.72E+09	8.53E+08	1.35E+09	10	10	17	17	6	18	Deamidat
588	10018	tr|E9PR30|E9PR30_HUMAN	154.29	1.36E+09	1.72E+09	8.53E+08	1.35E+09	10	10	17	17	6	18	Deamidat
1495	2106	sp|Q8IWR0|Z3H7A_HUMAN	154.19	1.64E+06	2.64E+06	2.59E+06	4.38E+05	15	15	2	5	5	1	Carbamid
1654	1870	sp|Q8N766|EMC1_HUMAN	154.15	1.33E+06	5.44E+05	7.80E+05	3.83E+06	14	14	5	2	2	7	Carbamid
1967	5990	sp|Q9ULC4|MCTS1_HUMAN	154.11	4.97E+06	2.79E+06		1.49E+05	4	4	3	2	0	1	Carbamid
1745	3074	tr|F8VRR3|F8VRR3_HUMAN	154.07	1.02E+06	1.19E+06	3.86E+06	1.00E+06	7	7	2	4	5	3	Carbamid
1745	3075	tr|F8VQZ7|F8VQZ7_HUMAN	154.07	1.02E+06	1.19E+06	3.86E+06	1.00E+06	7	7	2	4	5	3	Carbamid
1745	3076	sp|P50579|MAP2_HUMAN	154.07	1.02E+06	1.19E+06	3.86E+06	1.00E+06	7	7	2	4	5	3	Carbamid
1544	1947	sp|P49916|DNLI3_HUMAN	153.84	5.95E+04	5.72E+05	2.81E+05	5.00E+06	14	14	1	5	1	5	Carbamid
1581	3187	sp|P41227|NAA10_HUMAN	153.81	3.96E+06	4.55E+06	2.84E+06	9.79E+05	8	8	5	5	3	2	Carbamid
1406	4698	tr|A0A087WVC1|A0A087WVC1_HUMAN	153.75			3.37E+05	2.39E+05	5	1	2	4	8	5	Deamidat
1406	4699	sp|Q9BQ39|DDX50_HUMAN	153.75			3.37E+05	2.39E+05	5	1	2	4	8	5	Deamidat
1703	2203	sp|O75083|WDR1_HUMAN	153.66		2.52E+06		2.54E+06	11	11	0	4	0	3	Carbamid
1698	3608	sp|Q14739|LBR_HUMAN	153.64	8.51E+06	7.64E+06	2.94E+06	9.77E+05	8	8	5	4	3	1	
1706	3161	sp|P21281|VATB2_HUMAN	153.61	3.59E+06	4.47E+06		1.17E+06	8	8	8	3	0	1	Carbamid
1741	2634	sp|P53582|MAP11_HUMAN	153.36	3.91E+06	4.06E+06	1.25E+06		10	10	3	6	1	0	Carbamid
1102	2291	sp|P61026|RAB10_HUMAN	153.31	5.74E+06	3.29E+06	6.72E+06	1.53E+06	10	7	8	6	8	6	
1820	2635	tr|E7EVJ5|E7EVJ5_HUMAN	153.02			4.43E+05		9	1	4	3	4	0	Carbamid
1820	2640	sp|Q96F07|CYFP2_HUMAN	153.02			4.43E+05		9	1	4	3	4	0	Carbamid
1369	3080	sp|Q9BYD1|RM13_HUMAN	152.97	1.12E+06	7.24E+06	2.10E+07	1.25E+07	9	9	2	4	7	6	
2090	5007	sp|Q6P587|FAHD1_HUMAN	152.92	1.29E+06	7.54E+05		9.66E+05	6	6	2	2	0	2	Carbamid
2206	3239	sp|Q96DF8|ESS2_HUMAN	152.84		3.57E+05	1.06E+05	5.11E+05	7	7	0	2	1	2	Acetylatic
1852	5119	sp|O95295|SNAPN_HUMAN	152.71	5.67E+06	2.81E+06	1.41E+06	1.53E+06	5	5	4	3	1	2	Carbamid
1514	2412	sp|P22234|PUR6_HUMAN	152.68	4.82E+06	2.65E+06	6.13E+05	1.52E+06	11	11	8	5	1	2	Carbamid
1466	4143	sp|Q8N5K1|CISD2_HUMAN	152.63	7.78E+06	8.71E+06	6.47E+06	1.06E+07	7	7	6	5	3	4	Carbamid
2059	2678	tr|F5H0F9|F5H0F9_HUMAN	152.43	1.81E+06	1.71E+06	9.13E+05		11	11	6	5	2	0	Carbamid
2059	2690	sp|Q9UJX4|APC5_HUMAN	152.43	1.81E+06	1.71E+06	9.13E+05		11	11	6	5	2	0	Carbamid
1957	5533	sp|O14828|SCAM3_HUMAN	152.22	2.19E+06		1.04E+06	1.31E+06	6	6	4	0	2	2	Deamidat
702	3153	tr|H0YKD8|H0YKD8_HUMAN	151.96	7.46E+07	9.11E+07	6.64E+08	6.41E+08	12	12	7	7	17	18	Carbamid
702	3158	sp|P46779|RL28_HUMAN	151.96	7.46E+07	9.11E+07	6.64E+08	6.41E+08	12	12	7	7	17	18	Carbamid
1705	3705	sp|Q8N183|NDUF2_HUMAN	151.93	3.79E+06	5.23E+05	6.48E+05	9.38E+05	7	7	6	2	2	3	
1286	6209	sp|Q9HD34|LYRM4_HUMAN	151.9	3.72E+07	4.16E+07	3.36E+07	3.65E+07	6	6	7	7	4	7	
1651	2258	sp|O60264|SMCA5_HUMAN	151.89	2.15E+05	5.30E+05	1.72E+06	1.43E+06	15	11	2	5	5	2	Carbamid
2192	4544	sp|Q9BUL8|PDC10_HUMAN	151.88	2.39E+06	1.19E+06			5	5	3	2	0	0	
1390	2199	sp|Q13586|STIM1_HUMAN	151.88	3.64E+06	3.32E+06	6.71E+05	1.26E+06	13	12	5	6	2	2	Carbamid
1390	2208	tr|G0XQ39|G0XQ39_HUMAN	151.88	3.64E+06	3.32E+06	6.71E+05	1.26E+06	13	12	5	6	2	2	Carbamid
1869	2854	sp|Q96BT7|ALKB8_HUMAN	151.79	3.14E+06	1.29E+06	2.96E+06	4.52E+05	10	10	3	4	6	1	Carbamid
2036	2576	sp|O60610|DIAP1_HUMAN	151.72	5.72E+05	2.43E+05			9	8	3	3	0	1	Carbamid
2036	2577	tr|A0A0G2JH68|A0A0G2JH68_HUMAN	151.72	5.72E+05	2.43E+05			9	8	3	3	0	1	Carbamid
2036	2578	tr|H9KV28|H9KV28_HUMAN	151.72	5.72E+05	2.43E+05			9	8	3	3	0	1	Carbamid
2036	2579	tr|A0A140T8Z0|A0A140T8Z0_HUMAN	151.72	5.72E+05	2.43E+05			9	8	3	3	0	1	Carbamid
1899	3260	sp|Q9Y3F4|STRAP_HUMAN	151.57		9.99E+05	6.67E+06	2.37E+06	8	8	0	3	4	2	Carbamid
1975	3315	tr|A6NEM2|A6NEM2_HUMAN	151.56	9.92E+05	1.26E+06	3.91E+05	8.37E+05	8	8	2	3	1	3	Acetylatic
1975	3321	sp|P51610|HCFC1_HUMAN	151.56	9.92E+05	1.26E+06	3.91E+05	8.37E+05	8	8	2	3	1	3	Acetylatic
1428	2289	sp|P40227|TCPZ_HUMAN	151.54	6.42E+06	1.72E+06	3.27E+06	2.45E+06	12	12	7	5	3	3	Carbamid
2037	3183	sp|Q8IWW6|RHG12_HUMAN	151.37	1.18E+06	6.73E+05		2.03E+05	7	7	2	3	0	1	

1282	2700	sp|O00505|IMA4_HUMAN	151.33	2.14E+06	3.51E+06	3.83E+06	2.45E+06	10	6	5	7	5	3	Carbamid
1372	4761	sp|Q9NX63|MIC19_HUMAN	151.31	8.54E+06	1.13E+07	6.34E+06	1.04E+07	6	6	6	4	3	4	Carbamid
1372	4805	tr|C9JRZ6|C9JRZ6_HUMAN	151.31	8.54E+06	1.13E+07	6.34E+06	1.04E+07	6	6	6	4	3	4	Carbamid
1534	6261	tr|B9A057|B9A057_HUMAN	151.3	3.11E+07	2.49E+07	1.75E+07	2.29E+07	4	4	6	4	3	3	Carbamid
1534	6262	sp|Q86WW8|COA5_HUMAN	151.3	3.11E+07	2.49E+07	1.75E+07	2.29E+07	4	4	6	4	3	3	Carbamid
1383	4275	sp|Q9Y3C6|PPIL1_HUMAN	151.22	1.60E+07	2.06E+07	1.18E+07	1.66E+07	7	7	5	6	5	4	Carbamid
1254	2961	sp|Q96DV4|RM38_HUMAN	151.17	6.83E+05	4.96E+06	7.26E+06	1.31E+07	12	12	4	6	10	6	Carbamid
1381	2572	sp|Q9HD33|RM47_HUMAN	150.98	1.97E+06	9.00E+05	1.61E+07	2.74E+07	9	9	2	3	7	8	Carbamid
2124	3530	sp|Q96MF7|NSE2_HUMAN	150.98	1.81E+05		1.50E+06	1.18E+06	7	7	1	0	3	2	Carbamid
1566	6435	sp|O75915|PRAF3_HUMAN	150.89	4.97E+06	4.74E+06	5.66E+06	5.75E+06	5	5	4	3	5	3	Acetylatic
1452	3571	sp|Q8TB61|S35B2_HUMAN	150.86	2.08E+06	2.92E+06	3.94E+06	9.82E+06	7	7	4	4	3	6	Carbamid
1489	5834	tr|J9JIE6|J9JIE6_HUMAN	150.69	3.37E+07	3.88E+07	5.31E+07	3.14E+07	4	4	5	4	4	3	
1489	6229	sp|Q9UM00|TMCO1_HUMAN	150.69	3.37E+07	3.88E+07	5.31E+07	3.14E+07	4	4	5	4	4	3	
1699	3940	sp|O95639|CPSF4_HUMAN	150.65	1.37E+06	4.77E+06	5.26E+05	2.94E+06	6	6	4	5	2	3	Carbamid
1051	3110	tr|O95205|O95205_HUMAN	150.63	3.19E+06	1.63E+06	3.21E+06	1.72E+06	8	3	13	7	8	6	Carbamid
1051	3128	sp|Q5VZF2|MBNL2_HUMAN	150.63	3.19E+06	1.63E+06	3.21E+06	1.72E+06	8	3	13	7	8	6	Carbamid
1973	3564	tr|G3V1C3|G3V1C3_HUMAN	150.55	6.89E+06	9.62E+05	1.37E+06		8	8	10	1	2	0	
1973	3565	sp|Q9BZZ5|API5_HUMAN	150.55	6.89E+06	9.62E+05	1.37E+06		8	8	10	1	2	0	
1486	4487	tr|K4DI92|K4DI92_HUMAN	150.49	1.54E+07	1.13E+07	8.95E+06	5.10E+06	6	6	5	4	3	2	Acetylatic
1486	4488	sp|Q6NW29|RWDD4_HUMAN	150.49	1.54E+07	1.13E+07	8.95E+06	5.10E+06	6	6	5	4	3	2	Acetylatic
1754	3932	sp|Q96C19|EFHD2_HUMAN	150.47		2.78E+06		1.46E+06	6	4	2	4	1	4	Carbamid
1752	2457	tr|F8W7U8|F8W7U8_HUMAN	150.4	6.73E+04	5.32E+04		7.64E+05	11	11	1	1	0	2	Deamidat
1752	2458	sp|P49959|MRE11_HUMAN	150.4	6.73E+04	5.32E+04		7.64E+05	11	11	1	1	0	2	Deamidat
1767	2149	tr|G5EA48|G5EA48_HUMAN	150.33	2.30E+06	7.78E+05		1.88E+05	12	9	4	3	2	1	Carbamid
1767	2150	sp|Q7Z6B7|SRGP1_HUMAN	150.33	2.30E+06	7.78E+05		1.88E+05	12	9	4	3	2	1	Carbamid
1739	3694	tr|A0A087X1N3|A0A087X1N3_HUMAN	150.24		2.36E+06	3.36E+06	4.40E+06	7	7	0	4	1	3	Carbamid
1739	3697	sp|O75818|RPP40_HUMAN	150.24		2.36E+06	3.36E+06	4.40E+06	7	7	0	4	1	3	Carbamid
1218	4127	sp|P82932|RT06_HUMAN	150.06	1.69E+07	2.77E+07	4.26E+07	4.57E+07	6	6	4	6	6	6	Carbamid
1307	3168	sp|P62834|RAP1A_HUMAN	150.02	5.83E+06	5.30E+06	2.85E+06	2.50E+06	10	7	6	3	5	6	Carbamid
1578	4209	tr|A0A0R4J2E8|A0A0R4J2E8_HUMAN	149.85	2.21E+06	1.49E+07		3.67E+06	10	10	4	9	0	5	Carbamid
1578	4210	sp|P43243|MATR3_HUMAN	149.85	2.21E+06	1.49E+07		3.67E+06	10	10	4	9	0	5	Carbamid
1578	4230	tr|A8MXP9|A8MXP9_HUMAN	149.85	2.21E+06	1.49E+07		3.67E+06	10	10	4	9	0	5	Carbamid
1969	5217	sp|Q9Y5S9|RBM8A_HUMAN	149.8	4.19E+06	2.99E+06	9.05E+05	1.67E+06	5	5	3	2	1	1	Acetylatic
1613	3582	sp|Q9ULV0|MYO5B_HUMAN	149.47	1.69E+06	1.47E+06	1.69E+07	7.26E+06	8	3	1	5	5	5	Carbamid
2034	3595	sp|P26232|CTNA2_HUMAN	149.37	5.11E+05				7	1	5	4	0	0	Carbamid
1617	3667	tr|A0A2R8Y765|A0A2R8Y765_HUMAN	149.15	4.48E+06	2.15E+06	7.15E+06	4.79E+05	7	6	5	3	6	3	Deamidat
1617	3670	tr|J3QKS7|J3QKS7_HUMAN	149.15	4.48E+06	2.15E+06	7.15E+06	4.79E+05	7	6	5	3	6	3	Deamidat
1617	3671	tr|A0A2R8Y7U4|A0A2R8Y7U4_HUMAN	149.15	4.48E+06	2.15E+06	7.15E+06	4.79E+05	7	6	5	3	6	3	Deamidat
1617	3672	tr|A0A2U3TZQ7|A0A2U3TZQ7_HUMAN	149.15	4.48E+06	2.15E+06	7.15E+06	4.79E+05	7	6	5	3	6	3	Deamidat
1617	3676	tr|A0A2R8Y7I9|A0A2R8Y7I9_HUMAN	149.15	4.48E+06	2.15E+06	7.15E+06	4.79E+05	7	6	5	3	6	3	Deamidat
1617	3677	tr|A0A2R8Y4T4|A0A2R8Y4T4_HUMAN	149.15	4.48E+06	2.15E+06	7.15E+06	4.79E+05	7	6	5	3	6	3	Deamidat
1617	3678	tr|A0A2R8Y855|A0A2R8Y855_HUMAN	149.15	4.48E+06	2.15E+06	7.15E+06	4.79E+05	7	6	5	3	6	3	Deamidat
1617	3680	tr|B4DGM3|B4DGM3_HUMAN	149.15	4.48E+06	2.15E+06	7.15E+06	4.79E+05	7	6	5	3	6	3	Deamidat
1617	3685	sp|Q969G3|SMCE1_HUMAN	149.15	4.48E+06	2.15E+06	7.15E+06	4.79E+05	7	6	5	3	6	3	Deamidat
1272	4331	tr|A0A0C4DGS0|A0A0C4DGS0_HUMAN	149.14	2.78E+07	3.16E+07	2.77E+07	1.77E+07	9	9	6	8	4	3	
1272	4332	sp|P56556|NDUA6_HUMAN	149.14	2.78E+07	3.16E+07	2.77E+07	1.77E+07	9	9	6	8	4	3	
1272	4335	tr|A0A2Y9D025|A0A2Y9D025_HUMAN	149.14	2.78E+07	3.16E+07	2.77E+07	1.77E+07	9	9	6	8	4	3	
1606	3937	sp|P19474|RO52_HUMAN	149.05	1.00E+05		8.69E+06	1.80E+06	8	8	1	0	7	6	Carbamid
1683	4861	tr|H7BXJ4|H7BXJ4_HUMAN	149.02	5.65E+06		2.40E+06		6	5	10	1	4	3	Carbamid
1683	4883	sp|Q0JRZ9|FCHO2_HUMAN	149.02	5.65E+06		2.40E+06		6	5	10	1	4	3	Carbamid
1845	3000	tr|A0A1W2PPH1|A0A1W2PPH1_HUMAN	148.78	7.03E+05	6.27E+05	1.13E+06	3.91E+06	8	8	1	2	1	7	
1845	3001	sp|P23368|MAOM_HUMAN	148.78	7.03E+05	6.27E+05	1.13E+06	3.91E+06	8	8	1	2	1	7	
1845	3010	tr|A0A1W2PQF8|A0A1W2PQF8_HUMAN	148.78	7.03E+05	6.27E+05	1.13E+06	3.91E+06	8	8	1	2	1	7	
1845	3011	tr|A0A1W2PQT3|A0A1W2PQT3_HUMAN	148.78	7.03E+05	6.27E+05	1.13E+06	3.91E+06	8	8	1	2	1	7	
1845	3013	tr|A0A1W2PQY8|A0A1W2PQY8_HUMAN	148.78	7.03E+05	6.27E+05	1.13E+06	3.91E+06	8	8	1	2	1	7	
1845	3024	tr|A0A1W2PQH3|A0A1W2PQH3_HUMAN	148.78	7.03E+05	6.27E+05	1.13E+06	3.91E+06	8	8	1	2	1	7	
1845	3036	tr|A0A1W2PQ37|A0A1W2PQ37_HUMAN	148.78	7.03E+05	6.27E+05	1.13E+06	3.91E+06	8	8	1	2	1	7	
2140	4890	sp|Q9Y3A4|RRP7A_HUMAN	148.76		9.83E+05	9.22E+06	1.20E+07	5	5	0	1	2	5	Carbamid
1932	5987	sp|Q9HAF1|EAF6_HUMAN	148.74	1.03E+06	3.63E+06	4.80E+06	2.00E+06	6	6	2	3	4	2	Deamidat
1347	7290	sp|P56134|ATPK_HUMAN	148.64	1.92E+07	2.16E+07	2.08E+07	2.42E+07	4	2	5	5	4	4	Carbamid
1980	2313	tr|A0A088AWN3|A0A088AWN3_HUMAN	148.49	6.62E+05	2.99E+05	2.93E+06	2.07E+05	11	11	4	1	2	2	Carbamid
2381	3651	sp|P47929|LEG7_HUMAN	148.45					7	7	0	0	0	0	Carbamid
1990	4842	sp|Q9Y277|VDAC3_HUMAN	148.36	1.12E+06	9.77E+05	2.66E+06	3.26E+06	6	4	2	2	2	3	Carbamid
1398	3659	sp|O60869|EDF1_HUMAN	148.34	3.13E+07	1.14E+07	2.39E+06	6.84E+06	7	7	8	5	3	4	Acetylatic
1936	2613	sp|Q8N3Z3|GTPB8_HUMAN	148.31	1.64E+06		1.26E+06	7.37E+05	9	9	5	0	2	1	Carbamid
1485	4436	sp|P00403|COX2_HUMAN	148.22	9.86E+06	1.03E+07	9.79E+06	7.98E+06	6	6	6	4	6	2	
1515	3005	sp|Q8WVX9|FACR1_HUMAN	148.16	4.17E+06	3.24E+06	3.59E+06	3.44E+06	8	8	6	3	4	2	
1876	2974	sp|Q9NXV6|CARF_HUMAN	148.15	2.12E+06	2.56E+06	2.82E+06		9	9	3	6	3	0	Carbamid
2191	4412	sp|Q15056|IF4H_HUMAN	148.12	1.05E+07	1.78E+05			6	6	6	2	0	0	Carbamid
2407	4249	tr|D6R938|D6R938_HUMAN	147.92	8.18E+05	1.03E+06		2.14E+05	7	5	3	3	0	1	Carbamid
2407	4250	sp|Q13557|KCC2D_HUMAN	147.92	8.18E+05	1.03E+06		2.14E+05	7	5	3	3	0	1	Carbamid
2407	4251	tr|E9PBG7|E9PBG7_HUMAN	147.92	8.18E+05	1.03E+06		2.14E+05	7	5	3	3	0	1	Carbamid
2407	4252	tr|E9PF82|E9PF82_HUMAN	147.92	8.18E+05	1.03E+06		2.14E+05	7	5	3	3	0	1	Carbamid
1694	5849	sp|O15235|RT12_HUMAN	147.84	4.64E+06	4.23E+06	1.63E+07	1.81E+07	5	5	2	3	3	4	Carbamid
1619	2461	sp|Q9NP80|PLPL8_HUMAN	147.7	3.96E+06	4.41E+06	2.61E+06	5.35E+06	12	11	7	5	4	8	Carbamid
1690	2995	sp|Q15054|DPOD3_HUMAN	147.62	1.04E+06	6.25E+06		3.98E+06	9	9	2	9	0	4	Carbamid
1919	3056	tr|H0Y714|H0Y714_HUMAN	147.51	4.35E+06	1.02E+07	1.43E+07	4.53E+06	10	10	3	6	6	2	
1919	3059	sp|Q96G21|IMP4_HUMAN	147.51	4.35E+06	1.02E+07	1.43E+07	4.53E+06	10	10	3	6	6	2	
1919	3120	tr|E7ENR5|E7ENR5_HUMAN	147.51	3.12E+06	8.67E+06	1.19E+07	4.53E+06	9	9	2	5	5	2	
1364	2210	sp|Q15599|NHRF2_HUMAN	147.49	5.45E+06	3.28E+06	3.72E+06	8.02E+06	11	11	5	4	6	6	Acetylatic
1421	3178	tr|A0A0D9SEY1|A0A0D9SEY1_HUMAN	147.43	1.45E+05				8	1	7	6	4	4	Carbamid
1985	2965	sp|P23634|AT2B4_HUMAN	147.33	1.23E+06	3.57E+05		8.49E+05	8	7	3	1	0	2	Carbamid
1986	3199	tr|A0A087WT95|A0A087WT95_HUMAN	147.21	3.15E+06	2.43E+06	5.10E+05	1.12E+06	9	9	4	3	2	1	Carbamid
1986	3200	sp|P49753|ACOT2_HUMAN	147.21	3.15E+06	2.43E+06	5.10E+05	1.12E+06	9	9	4	3	2	1	Carbamid
1986	3208	sp|Q86TX2|ACOT1_HUMAN	147.21	3.15E+06	2.43E+06	5.10E+05	1.12E+06	9	9	4	3	2	1	Carbamid
1986	3209	tr|A0A087X0W7|A0A087X0W7_HUMAN	147.21	3.15E+06	2.43E+06	5.10E+05	1.12E+06	9	9	4	3	2	1	Carbamid
1886	3596	sp|Q14525|KT33B_HUMAN	147.13					7	1	2	1	1	1	Carbamid
1812	5128	sp|Q7Z7K0|COXM1_HUMAN	147.1	1.92E+07	1.25E+07	2.70E+06	1.95E+06	5	5	4	3	1	2	Carbamid
1926	3224	sp|P42338|PK3CB_HUMAN	146.98	1.41E+06	1.55E+06	6.07E+05		13	13	7	3	2	0	Carbamid
1977	5360	tr|E9PFK9|E9PFK9_HUMAN	146.89	6.87E+06	3.54E+06	5.31E+06	1.93E+06	5	5	5	2	1	2	Carbamid
1977	5363	sp|Q9UJ41|RABX5_HUMAN	146.89	6.87E+06	3.54E+06	5.31E+06	1.93E+06	5	5	5	2	1	2	Carbamid
1977	5542	tr|Q3HKR1|Q3HKR1_HUMAN	146.89	6.87E+06	3.54E+06	5.31E+06	1.93E+06	5	5	5	2	1	2	Carbamid
1567	6160	sp|O95168|NDUB4_HUMAN	146.69	8.47E+06	9.24E+06	1.83E+07	1.23E+07	4	4	5	4	4	3	
1974	4142	sp|P30041|PRDX6_HUMAN	146.67	1.14E+07	1.51E+06	1.89E+06	1.01E+06	7	7	8	2	2	1	
1630	2411	sp|Q9H857|NT5D2_HUMAN	146.53	1.90E+06	1.13E+06	1.18E+06	7.63E+05	10	10	4	4	4	2	Carbamid
1724	3174	sp|Q9P0W2|HM20B_HUMAN	146.43	6.56E+06	1.04E+06	1.22E+06	0	10	10	7	1	2	1	
1905	3466	sp|O94842|TOX4_HUMAN	146.25	1.64E+06	6.67E+06	6.55E+05	1.92E+06	7	7	2	5	1	2	
1258	3119	sp|Q04917|1433F_HUMAN	146.24	1.33E+06	1.02E+07	1.55E+06		9	5	6	7	6	4	Carbamid
2129	3805	sp|P36957|ODO2_HUMAN	146.08	6.72E+05	1.26E+06	8.82E+05	8.88E+05	7	7	2	3	1	2	
1521	3050	sp|Q13162|PRDX4_HUMAN	145.87	3.22E+06	2.48E+06	1.21E+06		9	7	5	6	3	1	Carbamid
2014	5432	tr|A0A024R216|A0A024R216_HUMAN	145.83	2.85E+06	6.02E+06	1.43E+06	1.63E+06	6	4	3	5	2	2	
2014	5433	sp|Q9Y3E1|HDGR3_HUMAN	145.83	2.85E+06	6.02E+06	1.43E+06	1.63E+06	6	4	3	5	2	2	
791	5198	sp|P17661|DESM_HUMAN	145.82		0			7	1	6	9	11	9	Oxidation
1855	3298	sp|Q96H79|ZCCHL_HUMAN	145.59	2.05E+06	9.78E+05	2.01E+06	3.09E+06	7	7	3	4	3	4	Carbamid
1798	4029	sp|Q15758|AAAT_HUMAN	145.47	2.16E+06	1.87E+06	1.09E+06	2.03E+06	6	6	4	4	1	3	Carbamid
1476	2725	sp|P78539|SRPX_HUMAN	145.41	5.16E+06	5.63E+06	9.15E+06	5.17E+06	10	10	3	5	6	4	Carbamid
1459	2316	sp|O15042|SR140_HUMAN	145.4	1.05E+07	6.92E+06	1.07E+07	8.35E+05	11	11	11	7	6	1	
1840	3611	sp|P51648|AL3A2_HUMAN	145.23	4.11E+05	2.48E+05	5.86E+05	2.74E+06	7	6	1	1	2	4	Carbamid
1840	3783	tr|J3QRD1|J3QRD1_HUMAN	145.23	4.11E+05	2.48E+05	5.86E+05	2.74E+06	7	6	1	1	2	4	Carbamid
2148	4896	sp|Q9Y5K8|VATD_HUMAN	145.08	2.66E+05	3.03E+05		0	6	6	1	1	0	1	
2148	5100	tr|G3V2S6|G3V2S6_HUMAN	145.08	2.66E+05	3.03E+05		0	6	6	1	1	0	1	
1723	6337	sp|Q96HQ2|C2AIL_HUMAN	145.07	6.74E+06	4.69E+06	5.57E+06	4.59E+06	5	5	4	3	2	2	
1615	2360	sp|P46013|KI67_HUMAN	145.03	3.15E+06	1.19E+06	2.56E+06	1.94E+06	15	15	6	3	4	4	Carbamid
2165	5706	sp|P19634|SL9A1_HUMAN	145.02	1.00E+06	1.17E+06	4.32E+06	5.14E+06	4	4	2	2	2	1	Deamidat
1269	2390	sp|P50990|TCPQ_HUMAN	144.97	5.16E+06	1.24E+06	4.96E+06	5.00E+06	11	11	8	3	6	5	
2226	4721	sp|Q9BSD7|NTPCR_HUMAN	144.84	7.34E+05	7.23E+05	1.18E+06	8.18E+05	5	5	1	1	2	1	

2226	4781	tr|Q5TDF0|Q5TDF0_HUMAN	144.84	7.34E+05	7.23E+05	1.18E+06	8.18E+05	5	5	1	1	2	1	
2040	3758	tr|E7EQ69|E7EQ69_HUMAN	144.81	9.41E+05	3.85E+05		3.30E+05	8	8	3	1	0	1	Carbamid
2040	3759	sp|Q9GZZ1|NAA50_HUMAN	144.81	9.41E+05	3.85E+05		3.30E+05	8	8	3	1	0	1	Carbamid
1814	7341	sp|Q6ZYL4|TF2H5_HUMAN	144.61	1.28E+07	1.38E+07	7.71E+06	2.52E+07	3	3	3	4	2	3	Carbamid
1422	3314	tr|G5E948|G5E948_HUMAN	144.54					8	1	6	6	4	4	
1713	4216	sp|P17568|NDUB7_HUMAN	144.54	5.05E+06	7.44E+06	6.88E+06	8.10E+06	6	6	3	5	2	3	Carbamid
1609	6155	sp|P52298|NCBP2_HUMAN	144.43	1.60E+07	1.64E+07	1.17E+07	1.35E+07	5	5	4	4	3	3	Carbamid
1442	3067	sp|Q96PU8|QKI_HUMAN	144.33	4.90E+06	6.81E+05	5.92E+06	4.61E+06	8	8	7	3	7	5	Carbamid
865	7811	tr|Q49AN9|Q49AN9_HUMAN	144.3	3.82E+08	4.86E+08	3.38E+08	3.39E+08	8	8	11	13	9	8	Carbamid
865	7812	tr|F5H013|F5H013_HUMAN	144.3	3.82E+08	4.86E+08	3.38E+08	3.39E+08	8	8	11	13	9	8	Carbamid
865	7813	sp|P62308|RUXG_HUMAN	144.3	3.82E+08	4.86E+08	3.38E+08	3.39E+08	8	8	11	13	9	8	Carbamid
1012	2396	sp|P61619|S61A1_HUMAN	144.27	1.62E+07	1.64E+07	1.99E+07	2.10E+07	10	10	5	5	6	11	Carbamid
1012	2398	tr|B4DR61|B4DR61_HUMAN	144.27	1.62E+07	1.64E+07	1.99E+07	2.10E+07	10	10	5	5	6	11	Carbamid
1519	6236	tr|Q5TAQ0|Q5TAQ0_HUMAN	144.11	1.16E+07	7.05E+06	8.17E+06	4.79E+06	6	6	4	3	5	3	Carbamid
1519	6241	tr|V5IRT4|V5IRT4_HUMAN	144.11	1.16E+07	7.05E+06	8.17E+06	4.79E+06	6	6	4	3	5	3	Carbamid
1519	6242	sp|Q9BRT2|UQCC2_HUMAN	144.11	1.16E+07	7.05E+06	8.17E+06	4.79E+06	6	6	4	3	5	3	Carbamid
1460	2229	sp|Q8IU81|I2BP1_HUMAN	143.79	6.84E+06	3.48E+06	1.14E+06	0	12	12	9	5	2	3	Carbamid
1928	3169	sp|Q9UFC0|LRWD1_HUMAN	143.76	3.04E+05	2.21E+05	7.02E+05	1.30E+05	8	8	2	1	1	1	Carbamid
1219	3577	sp|P15531|NDKA_HUMAN	143.59	1.61E+06				7	1	6	5	5	5	Carbamid
1862	2582	sp|Q9BTC0|DIDO1_HUMAN	143.52	1.51E+07	9.24E+06	4.88E+06	3.10E+06	14	14	7	3	6	5	
1618	2440	tr|B6VEX4|B6VEX4_HUMAN	143.3	1.17E+07	3.54E+06	6.40E+06	6.82E+06	11	7	5	2	3	5	Carbamid
1618	2442	tr|A0A0A0MRT6|A0A0A0MRT6_HUMAN	143.3	1.17E+07	3.54E+06	6.40E+06	6.82E+06	11	7	5	2	3	5	Carbamid
1618	2445	sp|Q8IZP0|ABI1_HUMAN	143.3	1.17E+07	3.54E+06	6.40E+06	6.82E+06	11	7	5	2	3	5	Carbamid
1894	3225	sp|Q15738|NSDHL_HUMAN	143.15	6.37E+05	1.57E+06		6.61E+05	8	8	2	4	0	1	Carbamid
1894	3236	tr|C9JDR0|C9JDR0_HUMAN	143.15	6.37E+05	1.57E+06		6.61E+05	8	8	2	4	0	1	Carbamid
2022	5133	sp|Q8WUY8|NAT14_HUMAN	143.08		7.78E+05	1.39E+06	3.94E+06	5	4	1	1	1	4	Carbamid
1935	5438	tr|H0YDT0|H0YDT0_HUMAN	142.97	1.40E+07	9.95E+06	3.09E+06	4.47E+06	7	7	6	5	2	2	
1935	5441	sp|Q96D71|REPS1_HUMAN	142.97	1.40E+07	9.95E+06	3.09E+06	4.47E+06	7	7	6	5	2	2	
1935	5629	tr|E9PMG1|E9PMG1_HUMAN	142.97	1.40E+07	9.95E+06	3.09E+06	4.47E+06	7	7	6	5	2	2	
1850	2823	sp|Q9NRW7|VPS45_HUMAN	142.97	4.36E+06	9.79E+05	4.30E+06	1.08E+06	10	10	7	2	3	3	Acetylatic
2168	2886	tr|F5H365|F5H365_HUMAN	142.96				1.67E+05	8	5	1	0	0	1	Carbamid
2168	2887	sp|Q15436|SC23A_HUMAN	142.96				1.67E+05	8	5	1	0	0	1	Carbamid
1711	5074	sp|Q96EL2|RT24_HUMAN	142.96	4.79E+06	7.36E+06	7.95E+06	5.81E+06	6	6	3	4	3	2	Carbamid
1838	4444	tr|A0A0D9SF58|A0A0D9SF58_HUMAN	142.95	7.33E+04	7.94E+04	2.90E+06	5.23E+05	5	5	2	3	4	2	Carbamid
1838	4714	tr|E7EXA6|E7EXA6_HUMAN	142.95	7.33E+04	7.94E+04	2.90E+06	5.23E+05	5	5	2	3	4	2	Carbamid
1838	4715	sp|Q8WVB6|CTF18_HUMAN	142.95	7.33E+04	7.94E+04	2.90E+06	5.23E+05	5	5	2	3	4	2	Carbamid
2330	6555	tr|A0A0C4DGZ1|A0A0C4DGZ1_HUMAN	142.84	1.83E+06	2.59E+06	1.98E+06	2.20E+06	3	3	1	2	1	2	Carbamid
2330	6603	sp|Q8N5P1|ZC3H8_HUMAN	142.84	1.83E+06	2.59E+06	1.98E+06	2.20E+06	3	3	1	2	1	2	Carbamid
1326	3186	tr|A0A0D9SFL2|A0A0D9SFL2_HUMAN	142.81		4.01E+06		7.82E+06	10	10	0	6	0	8	Carbamid
1326	3193	sp|Q96T60|PNKP_HUMAN	142.81		4.01E+06		7.82E+06	10	10	0	6	0	8	Carbamid
1891	3991	sp|Q9UJX5|APC4_HUMAN	142.78	1.26E+06	1.46E+06	1.85E+06		8	8	4	4	4	0	Carbamid
1880	3043	sp|Q6IBS0|TWF2_HUMAN	142.74		1.85E+06	2.84E+05	1.94E+06	10	7	0	2	1	2	Carbamid
1146	6216	sp|P0C0S5|H2AZ_HUMAN	142.69	1.11E+08	1.75E+08	1.66E+08	1.47E+08	8	6	6	8	7	5	Deamidat
1146	6217	sp|Q71UI9|H2AV_HUMAN	142.69	1.11E+08	1.75E+08	1.66E+08	1.47E+08	8	6	6	8	7	5	Deamidat
2461	6763	sp|Q5D862|FILA2_HUMAN	142.65	4.05E+05	6.38E+05			3	3	2	1	0	0	
1565	4264	sp|Q86U42|PABP2_HUMAN	142.64	2.17E+06	7.71E+06	5.96E+06	5.62E+06	6	6	3	5	3	3	Carbamid
1740	4397	sp|Q9BUL5|PHF23_HUMAN	142.32	9.48E+06	9.52E+06	2.42E+06	2.66E+06	7	7	7	5	2	2	Carbamid
2075	3623	tr|A0A087WYF8|A0A087WYF8_HUMAN	142.06	6.82E+06		9.21E+05	1.89E+05	7	7	7	0	2	1	Carbamid
2075	3624	sp|Q53GG5|PDLI3_HUMAN	142.06	6.82E+06		9.21E+05	1.89E+05	7	7	7	0	2	1	Carbamid
2042	4571	sp|Q92615|LAR4B_HUMAN	141.98	1.37E+06	2.37E+06	8.32E+05	2.90E+06	5	5	1	2	1	3	Deamidat
1610	4879	sp|O96008|TOM40_HUMAN	141.92	7.87E+06	7.00E+06	4.69E+06	3.96E+06	5	5	6	4	2	2	
1670	3801	sp|Q9BWU1|CDK19_HUMAN	141.91		3.69E+05			7	1	3	4	2	4	Carbamid
663	4588	sp|P62851|RS25_HUMAN	141.72	8.57E+08	1.19E+09	1.55E+09	1.65E+09	9	8	9	13	12	13	Deamidat
1797	3907	tr|D6RDV0|D6RDV0_HUMAN	141.65	3.44E+05	1.32E+06	5.57E+06	4.14E+06	8	8	1	3	3	1	Carbamid
1797	3908	tr|D6RA37|D6RA37_HUMAN	141.65	3.44E+05	1.32E+06	5.57E+06	4.14E+06	8	8	1	3	3	1	Carbamid
1797	3911	tr|D6RIY7|D6RIY7_HUMAN	141.65	3.44E+05	1.32E+06	5.57E+06	4.14E+06	8	8	1	3	3	1	Carbamid
1797	3914	sp|Q13454|TUSC3_HUMAN	141.65	3.44E+05	1.32E+06	5.57E+06	4.14E+06	8	8	1	3	3	1	Carbamid
1408	3865	sp|P61604|CH10_HUMAN	141.59	2.75E+07	1.15E+07	3.74E+06	4.92E+06	9	5	7	6	1	3	
1582	3939	sp|P49643|PRI2_HUMAN	141.52	7.16E+06	8.60E+06	1.11E+07	8.78E+06	9	8	7	5	2	3	Deamidat
1522	3246	sp|O00629|IMA3_HUMAN	141.37	7.78E+05	6.74E+05	3.50E+06		9	5	6	3	5	1	Carbamid
1542	2045	sp|Q9H1A4|APC1_HUMAN	141.16	2.00E+06	2.09E+06	1.51E+06	3.82E+05	18	18	9	7	6	1	Carbamid
2276	3744	sp|Q6YN16|HSDL2_HUMAN	141.11	5.04E+05	3.51E+05	5.40E+05	2.77E+05	7	7	1	1	1	1	Carbamid
2058	3725	sp|Q9Y5Q9|TF3C3_HUMAN	140.99			4.56E+06	2.00E+06	7	7	0	0	5	2	Carbamid
2045	2833	sp|Q12789|TF3C1_HUMAN	140.91			5.47E+06	2.63E+06	11	11	0	0	7	5	Carbamid
1365	3709	sp|Q9Y5A9|YTHD2_HUMAN	140.8	0	2.59E+05	7.82E+05	2.54E+06	7	3	4	4	6	3	Deamidat
1470	2778	sp|Q96I25|SPF45_HUMAN	140.78	1.37E+07	1.62E+07	4.95E+06	9.55E+06	9	8	6	7	3	7	Carbamid
1662	3821	sp|Q6PK18|OGFD3_HUMAN	140.77	4.44E+06	3.14E+06	3.55E+06	5.91E+06	6	6	4	4	4	4	Carbamid
1373	2621	tr|C9J494|C9J494_HUMAN	140.73	6.62E+06	3.52E+06	5.00E+06	1.93E+06	9	9	7	4	6	2	Carbamid
1373	2622	sp|Q9H000|MKRN2_HUMAN	140.73	6.62E+06	3.52E+06	5.00E+06	1.93E+06	9	9	7	4	6	2	Carbamid
1900	3639	tr|D6RJ07|D6RJ07_HUMAN	140.72	7.37E+06	8.36E+06	6.67E+06	5.16E+06	7	7	4	5	3	3	Carbamid
1900	4095	sp|Q9UL40|ZN346_HUMAN	140.72	7.37E+06	8.36E+06	6.67E+06	5.09E+06	6	6	4	5	3	2	Carbamid
1744	3018	sp|O96013|PAK4_HUMAN	140.7	6.02E+06	3.65E+06		1.01E+06	9	9	5	6	0	2	Carbamid
1844	2762	sp|Q9NW13|RBM28_HUMAN	140.66	4.30E+07	3.83E+07	2.95E+07	4.50E+07	9	9	2	2	3	5	Acetylatic
2007	4085	tr|E9PJH7|E9PJH7_HUMAN	140.49	1.17E+06	1.51E+06	2.26E+06	4.75E+06	9	9	1	3	2	4	Carbamid
2007	4087	sp|Q9H936|GHC1_HUMAN	140.49	1.17E+06	1.51E+06	2.26E+06	4.75E+06	9	9	1	3	2	4	Carbamid
2236	5177	sp|Q9BZV1|UBXN6_HUMAN	140.32	1.95E+06	1.25E+06		5.36E+05	5	5	3	2	0	1	Carbamid
1637	4365	sp|Q9BYJ9|YTHD1_HUMAN	140.29	4.39E+04				6	1	5	2	5	2	
1859	10063	sp|Q9C005|DPY30_HUMAN	139.98	3.31E+06	4.05E+06	3.06E+06	8.35E+05	3	3	2	1	3	1	Acetylatic
1691	3238	sp|O00401|WASL_HUMAN	139.88	1.05E+07	7.45E+06		1.32E+06	8	8	8	5	0	3	Carbamid
1082	6633	sp|Q9Y3U8|RL36_HUMAN	139.83	3.44E+07	9.39E+07	7.32E+08	6.49E+08	6	6	6	7	7	9	Carbamid
2073	5057	tr|B1AKP7|B1AKP7_HUMAN	139.5	7.51E+05	8.95E+05	1.75E+06	1.30E+06	6	6	1	2	1	3	
2073	5058	tr|G3V162|G3V162_HUMAN	139.5	7.51E+05	8.95E+05	1.75E+06	1.30E+06	6	6	1	2	1	3	
2073	5059	tr|A0A087X260|A0A087X260_HUMAN	139.5	7.51E+05	8.95E+05	1.75E+06	1.30E+06	6	6	1	2	1	3	
2073	5060	tr|A0A087WYY0|A0A087WYY0_HUMAN	139.5	7.51E+05	8.95E+05	1.75E+06	1.30E+06	6	6	1	2	1	3	
2073	5073	sp|Q13148|TADBP_HUMAN	139.5	7.51E+05	8.95E+05	1.75E+06	1.30E+06	6	6	1	2	1	3	
1757	3987	sp|Q96FZ2|HMCES_HUMAN	139.36	2.57E+06	4.87E+06	6.31E+05	6.42E+05	6	6	4	5	1	2	Carbamid
1553	3037	sp|Q8IWB7|WDFY1_HUMAN	139.34	2.94E+06	3.67E+06	1.36E+06	1.65E+06	8	8	5	6	4	4	Carbamid
1333	4035	sp|Q9Y3C1|NOP16_HUMAN	139.27	8.34E+06	4.31E+06	2.36E+07	2.21E+07	7	7	7	4	4	8	
1851	4742	sp|P61326|MGN_HUMAN	139.25	2.48E+05				5	1	7	1	0	1	Acetylatic
1503	2247	sp|P43686|PRS6B_HUMAN	139.23	5.49E+06	1.65E+06	1.89E+06	5.44E+05	11	10	6	3	2	3	Acetylatic
1130	5317	sp|Q9NPA8|ENY2_HUMAN	139.05	1.07E+08	1.85E+08	1.21E+08	1.42E+08	6	6	6	9	7	6	Carbamid
1959	6606	sp|Q9UKK6|NXT1_HUMAN	138.89	2.22E+06	1.95E+06	2.12E+06	1.68E+06	4	4	3	2	2	1	Carbamid
1665	4260	sp|O43293|DAPK3_HUMAN	138.68	3.23E+06	3.86E+06	8.00E+06	4.62E+06	6	6	4	5	5	3	
1882	3249	sp|Q92466|DDB2_HUMAN	138.48		1.00E+06	2.12E+06	2.35E+06	9	9	0	4	4	4	Carbamid
1912	3699	sp|Q96J01|THOC3_HUMAN	138.43	2.05E+06	2.85E+06	2.63E+06	7.44E+05	7	7	3	5	3	1	Carbamid
2060	3856	sp|Q6NUM9|RETST_HUMAN	138.34	5.56E+05	1.45E+05			8	8	1	1	0	0	Carbamid
2116	3792	sp|Q16850|CP51A_HUMAN	138.21	5.59E+05		1.35E+06	2.59E+05	8	7	3	0	2	1	Carbamid
2116	3793	tr|A0A0C4DFL7|A0A0C4DFL7_HUMAN	138.21	5.59E+05		1.35E+06	2.59E+05	8	7	3	0	2	1	Carbamid
1873	3512	sp|Q10713|MPPA_HUMAN	138.17	1.97E+06	1.44E+06	8.95E+04	8.33E+05	8	8	5	3	1	2	Carbamid
1714	4384	sp|Q86UL3|GPAT4_HUMAN	138.13	1.09E+06	1.10E+06	3.26E+05	1.02E+06	6	6	3	4	1	3	
1802	3733	sp|O75569|PRKRA_HUMAN	138.09	6.44E+05	1.51E+06	8.09E+06	4.94E+06	7	7	1	2	6	4	Carbamid
1593	5143	tr|E5RJR5|E5RJR5_HUMAN	137.81	4.19E+06	6.07E+06	5.60E+06	1.02E+07	7	6	4	6	5	4	Carbamid
1593	5144	sp|P63208|SKP1_HUMAN	137.81	4.19E+06	6.07E+06	5.60E+06	1.02E+07	7	6	4	6	5	4	Carbamid
1708	3542	sp|O14818|PSA7_HUMAN	137.68	8.85E+06	4.72E+06	3.24E+06	3.48E+06	8	8	5	3	3	2	Deamidat
1921	3617	sp|Q14684|RRP1B_HUMAN	137.64	3.66E+05	1.19E+06	1.68E+06	8.04E+06	9	9	2	4	1	6	Carbamid
1772	2836	sp|Q8IZH2|XRN1_HUMAN	137.61	7.92E+05	9.26E+05	7.29E+05	1.42E+05	9	9	3	4	3	1	Carbamid
1861	5104	sp|Q9HCH5|SYTL2_HUMAN	137.56	1.52E+06	1.93E+06	4.65E+05	3.77E+05	4	4	4	6	1	2	Carbamid
1861	5153	tr|A0A0U1RQP0|A0A0U1RQP0_HUMAN	137.56	1.52E+06	1.93E+06	4.65E+05	3.77E+05	4	4	4	6	1	2	Carbamid
1861	5158	tr|A0A0U1RQH1|A0A0U1RQH1_HUMAN	137.56	1.52E+06	1.93E+06	4.65E+05	3.77E+05	4	4	4	6	1	2	Carbamid
1861	5247	tr|A0A0U1RR07|A0A0U1RR07_HUMAN	137.56	1.52E+06	1.93E+06	4.65E+05	3.77E+05	4	4	4	6	1	2	Carbamid
1726	3305	sp|Q5JSZ5|PRC2B_HUMAN	137.39	4.78E+06	8.93E+06	1.46E+06	2.95E+06	8	5	7	5	2	2	
2041	4140	sp|P50416|CPT1A_HUMAN	137.35		2.72E+05	3.49E+06	1.05E+06	7	7	0	2	3	2	Carbamid
2582	6032	tr|B4DDF4|B4DDF4_HUMAN	137.34	3.20E+05				4	3	3	0	0	0	Carbamid
2582	6033	sp|Q99439|CNN2_HUMAN	137.34	3.20E+05				4	3	3	0	0	0	Carbamid
2582	6034	tr|B4DUT8|B4DUT8_HUMAN	137.34	3.20E+05				4	3	3	0	0	0	Carbamid
2582	6062	tr|A0A087X271|A0A087X271_HUMAN	137.34	3.20E+05				4	3	3	0	0	0	Carbamid

2250	3889	tr|Q5JVZ5|Q5JVZ5_HUMAN	137.29			5.52E+05		7	7	0	0	2	0	
2250	3890	sp|Q96JJ3|ELMO2_HUMAN	137.29			5.52E+05		7	7	0	0	2	0	
2085	3527	tr|F8W726|F8W726_HUMAN	137.17	3.00E+05	6.79E+05		2.97E+05	7	7	1	2	0	1	Carbamid
2085	3528	sp|Q14157|UBP2L_HUMAN	137.17	3.00E+05	6.79E+05		2.97E+05	7	7	1	2	0	1	Carbamid
2207	4014	sp|Q66K74|MAP1S_HUMAN	137.16		1.94E+05	2.00E+05		6	6	0	1	1	0	Carbamid
796	6814	sp|P42766|RL35_HUMAN	137.15	6.46E+08	7.02E+08	1.55E+09	1.44E+09	7	6	9	9	15	10	Deamidat
1756	3769	sp|Q96BZ8|LENG1_HUMAN	137.1	1.42E+07	3.45E+07		2.04E+07	7	7	4	6	0	4	
2429	4852	sp|Q9Y673|ALG5_HUMAN	136.88	1.29E+06	1.93E+06	1.24E+06	4.44E+04	7	7	2	2	1	1	
1278	3524	tr|E5RFK5|E5RFK5_HUMAN	136.77	4.03E+06	1.22E+07	9.11E+06	9.49E+06	9	8	4	6	8	6	Carbamid
1278	3525	sp|P24863|CCNC_HUMAN	136.77	4.03E+06	1.22E+07	9.11E+06	9.49E+06	9	8	4	6	8	6	Carbamid
1641	5204	tr|E7ETU5|E7ETU5_HUMAN	136.73	2.91E+06	4.51E+06	2.08E+06	3.90E+06	5	5	3	4	3	2	Carbamid
1641	5206	tr|F6Y5H0|F6Y5H0_HUMAN	136.73	2.91E+06	4.51E+06	2.08E+06	3.90E+06	5	5	3	4	3	2	Carbamid
1641	5207	sp|P29558|RBMS1_HUMAN	136.73	2.91E+06	4.51E+06	2.08E+06	3.90E+06	5	5	3	4	3	2	Carbamid
1753	3520	tr|D6RAD4|D6RAD4_HUMAN	136.72	7.83E+06	5.91E+06	4.22E+06	2.17E+06	8	8	8	5	2	1	Carbamid
1753	3521	sp|P50613|CDK7_HUMAN	136.72	7.83E+06	5.91E+06	4.22E+06	2.17E+06	8	8	8	5	2	1	Carbamid
1833	3131	sp|Q01968|OCRL_HUMAN	136.66	2.51E+06	1.22E+06	4.89E+05	7.90E+03	9	9	4	2	2	1	Carbamid
1329	3578	sp|Q9BU23|LMF2_HUMAN	136.57	1.22E+06	3.34E+06	1.88E+07	1.38E+07	9	8	3	5	7	6	Carbamid
1790	4557	sp|P31153|METK2_HUMAN	136.53	1.54E+06	8.52E+05		9.73E+05	5	5	6	4	0	2	Carbamid
1547	2825	sp|P07992|ERCC1_HUMAN	136.53	1.06E+07	3.49E+07	2.37E+06	1.95E+06	11	11	5	10	2	3	Carbamid
1979	2308	sp|P98160|PGBM_HUMAN	136.35	1.00E+06	2.56E+05	3.68E+05		12	12	3	2	2	0	Carbamid
1461	2715	tr|A0A087X020|A0A087X020_HUMAN	136.32	2.58E+07	4.58E+06	4.50E+05	5.58E+05	13	13	14	2	1	3	Carbamid
1461	2716	sp|Q9Y3A5|SBDS_HUMAN	136.32	2.58E+07	4.58E+06	4.50E+05	5.58E+05	13	13	14	2	1	3	Carbamid
1659	3230	sp|Q96RP9|EFGM_HUMAN	136.26	3.86E+06	1.10E+06	8.31E+05	7.45E+05	7	7	6	2	1	1	
1331	2092	sp|Q13496|MTM1_HUMAN	136.14	4.88E+06	6.31E+06	4.62E+06	4.41E+06	13	11	8	10	3	3	Carbamid
2208	4053	sp|Q15555|MARE2_HUMAN	136.13	2.57E+06		2.10E+05		6	5	4	1	1	0	Carbamid
1626	3604	sp|P28288|ABCD3_HUMAN	136.13	4.75E+05	1.53E+06	4.68E+06	4.59E+06	7	7	3	6	3	4	
1825	3643	sp|P17612|KAPCA_HUMAN	136.12	4.37E+05	9.09E+05	9.10E+05	9.78E+05	9	3	2	6	4	3	Deamidat
1392	5538	sp|O95139|NDUB6_HUMAN	136	3.13E+06	1.02E+07	4.07E+06	5.70E+06	5	5	8	5	1	3	
1678	7350	sp|P17096|HMGA1_HUMAN	135.99	4.67E+07	9.85E+07	4.16E+07	1.15E+08	4	4	2	2	2	3	
1689	2686	sp|P35232|PHB_HUMAN	135.95	1.68E+06	2.18E+06	2.30E+06	3.78E+06	9	9	3	5	1	5	Carbamid
1858	6446	tr|Q8WVC2|Q8WVC2_HUMAN	135.91	4.45E+06	3.04E+06	1.35E+06	3.65E+06	4	4	4	2	1	3	Carbamid
1858	6447	sp|P63220|RS21_HUMAN	135.91	4.45E+06	3.04E+06	1.35E+06	3.65E+06	4	4	4	2	1	3	Carbamid
1946	4656	sp|O95292|VAPB_HUMAN	135.68	8.24E+05	1.97E+07			6	4	2	3	2	1	
2313	5951	sp|O15269|SPTC1_HUMAN	135.67	1.69E+05		1.70E+06	6.10E+05	4	4	1	0	3	2	Carbamid
1872	2967	sp|O75400|PR40A_HUMAN	135.64	1.04E+06	2.34E+06	4.22E+06	4.35E+06	9	9	2	4	5	4	
1872	2970	tr|A0A3F2YNY6|A0A3F2YNY6_HUMAN	135.64	1.04E+06	2.34E+06	4.22E+06	4.35E+06	9	9	2	4	5	4	
1811	5944	sp|Q13503|MED21_HUMAN	135.61	1.09E+07	1.14E+07	8.08E+06	9.15E+06	4	4	4	3	2	2	Carbamid
1737	3182	sp|Q8N5A5|ZGPAT_HUMAN	135.59	5.24E+06	9.94E+05	2.29E+06	4.41E+06	10	8	6	2	3	5	Carbamid
2087	3265	sp|Q96BK5|PINX1_HUMAN	135.42	4.73E+06	4.78E+06	1.37E+06	3.63E+06	8	8	4	4	1	4	Carbamid
2717	8967	sp|Q86TS9|RM52_HUMAN	135.27	6.25E+05		8.93E+04		2	2	2	0	1	0	Deamidat
1413	4760	sp|O14949|QCR8_HUMAN	135.1	1.96E+07	3.21E+07	4.32E+07	2.84E+07	7	7	3	6	5	3	Deamidat
1586	6602	sp|P68036|UB2L3_HUMAN	135.08	1.65E+07	2.78E+06	4.78E+06	2.49E+06	4	4	4	2	5	3	Carbamid
1791	3999	sp|Q8NBU5|ATAD1_HUMAN	135.05	1.99E+06	1.11E+06	3.41E+05		8	8	4	2	2	0	Carbamid
2424	5401	sp|Q6DD88|ATLA3_HUMAN	135.04	2.48E+05	2.96E+05	9.80E+05		5	4	1	2	1	1	Carbamid
1517	3653	sp|Q9Y5T4|DJC15_HUMAN	135.01	3.75E+06	8.22E+06	5.70E+06	8.02E+06	8	8	3	4	4	7	
1465	2231	sp|P14625|ENPL_HUMAN	134.97	4.85E+06	3.83E+06	1.69E+06	1.48E+06	12	11	5	5	3	5	
1749	4291	sp|Q8IXK0|PHC2_HUMAN	134.91	3.27E+06	5.14E+06	9.85E+05	1.19E+06	6	6	4	5	2	3	Carbamid
1749	4316	tr|B3KPJ4|B3KPJ4_HUMAN	134.91	3.27E+06	5.14E+06	9.85E+05	1.19E+06	6	6	4	5	2	3	Carbamid
1749	4320	tr|A0A0A0MSI2|A0A0A0MSI2_HUMAN	134.91	3.27E+06	5.14E+06	9.85E+05	1.19E+06	6	6	4	5	2	3	Carbamid
1805	4943	sp|P61081|UBC12_HUMAN	134.75	7.29E+06	3.98E+06	2.86E+05	5.29E+05	5	5	3	5	1	1	Carbamid
1983	3185	sp|Q14161|GIT2_HUMAN	134.68	1.05E+06	4.80E+05	1.15E+06	5.78E+05	8	5	4	3	2	1	Carbamid
1675	2478	sp|Q8TAA9|VANG1_HUMAN	134.66	2.07E+06	4.30E+06	1.66E+06	3.01E+06	9	9	2	5	1	3	Carbamid
1747	5630	sp|Q9BQ95|ECSIT_HUMAN	134.62	1.20E+06	1.20E+06	0	1.03E+06	4	4	4	3	1	4	
1510	2651	tr|B1AXG1|B1AXG1_HUMAN	134.49	1.97E+06		3.48E+05	1.92E+05	12	7	9	1	6	2	
1510	2652	sp|P51812|KS6A3_HUMAN	134.49	1.97E+06		3.48E+05	1.92E+05	12	7	9	1	6	2	
1510	2653	tr|B4DG22|B4DG22_HUMAN	134.49	1.97E+06		3.48E+05	1.92E+05	12	7	9	1	6	2	
1510	2654	tr|B7ZB17|B7ZB17_HUMAN	134.49	1.97E+06		3.48E+05	1.92E+05	12	7	9	1	6	2	
1866	3029	sp|Q96AE7|TTC17_HUMAN	134.49	2.96E+06	1.06E+06	5.55E+06	1.10E+06	8	7	4	2	5	2	Deamidat
2178	4710	sp|Q96EE3|SEH1_HUMAN	134.38			1.21E+06	2.92E+05	5	5	0	0	1	1	Carbamid
2178	4734	tr|K7ELV2|K7ELV2_HUMAN	134.38			1.21E+06	2.92E+05	5	5	0	0	1	1	Carbamid
1854	4754	sp|Q96A72|MGN2_HUMAN	134.26				2.74E+05	5	1	6	1	0	2	Acetylatic
2126	3804	sp|P08243|ASNS_HUMAN	134.24	2.06E+06	7.27E+05	1.34E+06	4.85E+05	7	7	5	1	1	1	Carbamid
2145	4680	sp|Q9NUQ2|PLCE_HUMAN	134.08	1.45E+06	5.71E+05	1.26E+06	5.79E+04	5	5	2	2	2	1	Carbamid
1996	4614	sp|Q96HE9|PRR11_HUMAN	134.03	3.57E+06	1.61E+06	4.35E+06	4.53E+06	6	6	4	1	1	2	Carbamid
1718	6045	sp|Q99653|CHP1_HUMAN	133.97	3.03E+06	1.45E+06	4.36E+05	2.19E+06	4	4	5	2	1	4	
1911	3552	sp|Q15323|K1H1_HUMAN	133.83					7	1	2	1	1	1	Carbamid
2144	3878	tr|A2A2V1|A2A2V1_HUMAN	133.81	1.38E+07	5.00E+06			7	7	5	3	0	0	Carbamid
2144	3879	sp|P04156|PRIO_HUMAN	133.81	1.38E+07	5.00E+06			7	7	5	3	0	0	Carbamid
998	3679	tr|A2IDC6|A2IDC6_HUMAN	133.69	2.67E+06	3.29E+07	2.04E+07	1.69E+07	9	9	4	8	11	6	
998	3683	sp|Q13084|RM28_HUMAN	133.69	2.67E+06	3.29E+07	2.04E+07	1.69E+07	9	9	4	8	11	6	
1633	2310	tr|Q5VWU8|Q5VWU8_HUMAN	133.68	3.04E+06	3.76E+06	8.12E+05	1.63E+06	13	13	6	4	1	4	Carbamid
2015	5673	sp|Q3MHD2|LSM12_HUMAN	133.59		1.43E+06	1.35E+06	4.17E+06	4	4	0	1	2	2	Carbamid
2015	5905	tr|K7ELG9|K7ELG9_HUMAN	133.59		1.43E+06	1.35E+06	4.17E+06	4	4	0	1	2	2	Carbamid
1382	3579	sp|O95716|RAB3D_HUMAN	133.44			6.11E+05	5.53E+05	7	3	3	4	6	4	Carbamid
2050	4351	sp|O60870|KIN17_HUMAN	133.29	3.19E+06	2.85E+06	6.47E+05	6.60E+05	6	6	3	4	1	2	Deamidat
2808	8136	sp|P30405|PPIF_HUMAN	133.27	3.01E+06			2.07E+05	3	3	3	0	0	1	Carbamid
2002	3605	sp|O00232|PSD12_HUMAN	132.98	1.77E+06	9.17E+05	1.03E+06		6	6	5	2	2	0	
1283	7496	sp|Q71UM5|RS27L_HUMAN	132.97	6.00E+06	7.00E+06	2.22E+07	2.83E+07	5	2	5	6	5	8	Carbamid
1283	7499	tr|H0YMV8|H0YMV8_HUMAN	132.97	6.00E+06	7.00E+06	2.22E+07	2.83E+07	5	2	5	6	5	8	Carbamid
1458	2197	sp|P23458|JAK1_HUMAN	132.94	2.15E+06	5.25E+06	5.38E+06	4.60E+06	16	16	3	7	9	7	Carbamid
1884	3546	sp|O43427|FIBP_HUMAN	132.87	9.08E+05	8.36E+05	1.46E+06	8.54E+04	7	7	2	3	2	1	
2128	4954	sp|Q9UBM7|DHCR7_HUMAN	132.83	2.13E+06	3.87E+06		1.95E+06	6	6	2	3	0	1	
1608	5096	tr|A0A2R8YDL4|A0A2R8YDL4_HUMAN	132.68	1.02E+06	4.31E+06	3.66E+06	1.83E+06	5	5	2	8	3	2	Carbamid
1608	5097	sp|A0JLT2|MED19_HUMAN	132.68	1.02E+06	4.31E+06	3.66E+06	1.83E+06	5	5	2	8	3	2	Carbamid
1608	5098	tr|J3KR33|J3KR33_HUMAN	132.68	1.02E+06	4.31E+06	3.66E+06	1.83E+06	5	5	2	8	3	2	Carbamid
2065	4440	sp|Q9HD23|MRS2_HUMAN	132.62	1.36E+06	9.05E+05	6.93E+05	1.23E+06	6	6	5	2	2	2	Carbamid
2105	3347	tr|A0A1W2PNZ9|A0A1W2PNZ9_HUMAN	132.28	8.37E+05	8.08E+05	7.04E+05		8	8	4	4	2	0	
2105	3354	sp|Q15652|JHD2C_HUMAN	132.28	8.37E+05	8.08E+05	7.04E+05		8	8	4	4	2	0	
1679	5396	sp|Q8WUW1|BRK1_HUMAN	132.17	8.56E+06	2.95E+06	6.18E+06	7.07E+06	5	5	4	2	4	3	Carbamid
1697	3099	sp|Q13393|PLD1_HUMAN	132.03	1.87E+06	2.04E+06	3.58E+06	3.09E+06	8	8	4	4	2	5	
2337	5122	sp|Q00587|BORG5_HUMAN	131.9	2.71E+05	1.03E+06	7.09E+05	4.75E+05	5	5	1	1	1	1	
2063	4276	sp|Q86X10|RLGPB_HUMAN	131.88	9.30E+05	5.36E+05	4.06E+05	1.62E+06	8	8	2	1	2	3	Carbamid
1960	2608	tr|F5H1S9|F5H1S9_HUMAN	131.48		1.66E+05			9	9	0	1	0	0	Carbamid
1960	2609	tr|G8JLB3|G8JLB3_HUMAN	131.48		1.66E+05			9	9	0	1	0	0	Carbamid
1960	2611	sp|Q9Y606|TRUA_HUMAN	131.48		1.66E+05			9	9	0	1	0	0	Carbamid
2161	6873	sp|P02792|FRIL_HUMAN	131.28	1.04E+06	1.22E+06	1.55E+06	1.21E+06	4	4	1	3	1	1	Carbamid
1682	4981	sp|Q12802|AKP13_HUMAN	131.18	6.28E+07	7.03E+07	4.46E+07	5.86E+07	6	6	3	6	2	6	Carbamid
1410	5888	tr|F5H702|F5H702_HUMAN	131.15	4.00E+07	6.08E+07	3.83E+07	5.14E+07	4	4	6	5	5	4	Oxidation
1410	6121	sp|Q96GC5|RM48_HUMAN	131.15	4.00E+07	6.08E+07	3.83E+07	5.14E+07	4	4	6	5	5	4	Oxidation
787	7422	sp|P62273|RS29_HUMAN	130.99	1.41E+09	1.59E+09	1.43E+09	1.75E+09	7	7	16	14	10	7	Carbamid
1978	2302	sp|Q9Y4C1|KDM3A_HUMAN	130.91	3.52E+06	1.77E+06	9.96E+05	1.79E+05	11	11	6	4	2	1	Carbamid
1978	2303	tr|F5H070|F5H070_HUMAN	130.91	3.52E+06	1.77E+06	9.96E+05	1.79E+05	11	11	6	4	2	1	Carbamid
2405	4987	sp|Q9P0V3|SH3B4_HUMAN	130.85	3.52E+05	3.78E+05		2.95E+05	5	5	2	2	0	1	Carbamid
2061	3201	sp|Q8TEX9|IPO4_HUMAN	130.77	1.47E+04	1.19E+05	1.64E+05		9	9	1	1	1	0	Carbamid
1765	2277	sp|Q13464|ROCK1_HUMAN	130.76	3.09E+05	8.85E+05	2.56E+05	3.50E+05	15	11	1	4	2	1	Carbamid
1883	3510	sp|O00746|NDKM_HUMAN	130.75		3.73E+05	1.76E+07	1.41E+07	9	9	0	1	6	6	
1883	3529	tr|F2Z2X0|F2Z2X0_HUMAN	130.75		3.73E+05	1.76E+07	1.41E+07	9	9	0	1	6	6	
1330	4980	sp|Q9UMY1|NOL7_HUMAN	130.74	2.99E+07	5.31E+07	2.43E+07	5.25E+07	7	7	7	8	1	7	Deamidat
2358	5653	sp|Q6NUK4|REEP3_HUMAN	130.72					5	4	0	0	0	0	
1669	3255	sp|Q12768|WASC5_HUMAN	130.64	1.18E+06	3.86E+05	1.65E+06	2.00E+05	9	8	5	4	2	3	Deamidat
1592	4450	sp|Q9Y3C7|MED31_HUMAN	130.53	8.74E+06	1.56E+07	7.05E+06	1.16E+07	8	8	4	8	3	4	Carbamid
1600	5933	tr|E9PN17|E9PN17_HUMAN	130.41	1.51E+07	8.68E+06	1.80E+07	1.10E+07	6	6	5	2	4	2	
1600	5936	tr|A0A3B3ITX4|A0A3B3ITX4_HUMAN	130.41	1.51E+07	8.68E+06	1.80E+07	1.10E+07	6	6	5	2	4	2	
1600	5937	sp|O75964|ATP5L_HUMAN	130.41	1.51E+07	8.68E+06	1.80E+07	1.10E+07	6	6	5	2	4	2	
2009	3499	sp|Q8WXX5|DNJC9_HUMAN	130.31	9.84E+04	8.92E+04	5.39E+06	3.90E+06	9	9	1	1	5	3	Carbamid
1874	3782	sp|Q7L8L6|FAKD5_HUMAN	130.3	9.37E+05	2.27E+06	1.78E+06	2.27E+06	9	9	2	3	3	4	Carbamid

1793	4867	sp|P49755|TMEDA_HUMAN	130.29	4.49E+06	3.45E+06	2.21E+06	3.19E+06	6	6	3	3	1	2	
2247	4408	sp|Q96PE3|INP4A_HUMAN	130.09	8.93E+05			6.72E+05	7	7	2	0	0	2	Carbamid
1938	4054	sp|Q8NEF9|SRFB1_HUMAN	129.95	2.77E+07	1.84E+07	1.23E+07	1.32E+07	8	8	4	4	3	4	
1687	3950	sp|P36543|VATE1_HUMAN	129.82	5.79E+06	3.91E+06	1.59E+06	3.00E+06	7	7	5	3	1	3	Acetylatic
1658	2812	sp|P48730|KC1D_HUMAN	129.78	1.70E+06	1.70E+06	6.69E+06	6.20E+06	10	9	6	3	3	7	Carbamid
1658	2813	tr|H7BYT1|H7BYT1_HUMAN	129.78	1.70E+06	1.70E+06	6.69E+06	6.20E+06	10	9	6	3	3	7	Carbamid
1963	6433	tr|A0A087X1G7|A0A087X1G7_HUMAN	129.74	4.28E+06	2.61E+06	3.39E+06	2.23E+06	4	4	3	2	3	1	Carbamid
1963	6434	sp|O60613|SEP15_HUMAN	129.74	4.28E+06	2.61E+06	3.39E+06	2.23E+06	4	4	3	2	3	1	Carbamid
1871	2796	sp|Q6P1N0|C2D1A_HUMAN	129.67	1.11E+06	3.18E+05	6.47E+05	6.88E+05	12	12	4	1	1	3	
2077	4329	sp|P61962|DCAF7_HUMAN	129.66	3.20E+06	2.86E+05	1.03E+06	0	7	7	5	1	2	1	Carbamid
2096	4697	sp|Q9H2D1|MFTC_HUMAN	129.61	1.01E+06	1.31E+06	1.48E+06		6	6	2	3	1	0	
1803	4073	tr|E9PP16|E9PP16_HUMAN	129.52	6.72E+05	6.96E+05			6	4	4	4	1	1	Deamidat
1803	4074	sp|Q8ND30|LIPB2_HUMAN	129.52	6.72E+05	6.96E+05			6	4	4	4	1	1	Deamidat
1556	3845	sp|P12036|NFH_HUMAN	129.38					9	5	3	2	2	2	Carbamid
1828	3351	tr|H0Y488|H0Y488_HUMAN	129.28	1.82E+05	1.46E+06	9.62E+05		12	11	3	2	5	0	Carbamid
1828	3352	tr|A0A1B0GTU5|A0A1B0GTU5_HUMAN	129.28	1.82E+05	1.46E+06	9.62E+05		12	11	3	2	5	0	Carbamid
1828	3361	sp|O14497|ARI1A_HUMAN	129.28	1.82E+05	1.46E+06	9.62E+05		12	11	3	2	5	0	Carbamid
1966	5226	sp|Q9P032|NDUF4_HUMAN	129.23	3.50E+06	3.17E+06	7.57E+05	7.81E+05	5	5	3	3	1	2	
1303	4543	tr|F2Z2E2|F2Z2E2_HUMAN	129.17					8	1	6	5	4	6	
1303	4551	sp|Q86VI3|IQGA3_HUMAN	129.17					8	1	6	5	4	6	
2167	5342	sp|Q9UL54|TAOK2_HUMAN	129.1	9.56E+04				5	2	1	1	0	0	
2176	4426	sp|Q96JM3|CHAP1_HUMAN	129.02	2.37E+06	2.52E+06		9.07E+04	6	6	4	3	0	1	Carbamid
2219	7016	sp|Q9P021|CRIPT_HUMAN	129.01	6.07E+06	2.82E+06	4.15E+05	3.80E+06	4	4	2	2	1	2	Carbamid
2241	3993	sp|Q6PGP7|TTC37_HUMAN	129	2.18E+05	1.46E+05	1.34E+05	2.59E+05	7	7	2	2	1	1	Carbamid
2338	6494	sp|Q96QR8|PURB_HUMAN	128.97			1.88E+06	1.17E+07	5	4	0	0	1	5	Carbamid
2326	4381	sp|O75718|CRTAP_HUMAN	128.92		3.41E+06	1.05E+06	1.08E+06	6	6	0	6	1	2	Carbamid
2326	4414	tr|C9JP16|C9JP16_HUMAN	128.92		3.41E+06	1.05E+06	1.08E+06	6	6	0	6	1	2	Carbamid
1693	2596	sp|Q96T88|UHRF1_HUMAN	128.79	4.00E+06	2.08E+06	6.79E+05		12	12	7	5	2	0	Carbamid
1693	2597	tr|A0A087WVR3|A0A087WVR3_HUMAN	128.79	4.00E+06	2.08E+06	6.79E+05		12	12	7	5	2	0	Carbamid
1693	2600	tr|A0A087WTW0|A0A087WTW0_HUMAN	128.79	4.00E+06	2.08E+06	6.79E+05		12	12	7	5	2	0	Carbamid
1810	6589	sp|Q9UBI6|GBG12_HUMAN	128.68	1.27E+07	1.24E+07	8.80E+06	1.09E+07	4	4	3	3	2	3	Carbamid
1877	3734	tr|G3V167|G3V167_HUMAN	128.66		9.41E+04	1.58E+06	1.38E+06	8	7	0	2	5	4	Carbamid
1877	3743	sp|Q9UGN5|PARP2_HUMAN	128.66		9.41E+04	1.58E+06	1.38E+06	8	7	0	2	5	4	Carbamid
1892	4492	sp|Q15008|PSMD6_HUMAN	128.65	2.31E+06	1.13E+06		1.28E+06	5	5	4	2	0	4	Carbamid
1868	2710	tr|G3V0E5|G3V0E5_HUMAN	128.39	4.49E+06	2.69E+06	1.75E+06	5.01E+05	9	9	9	5	2	1	
1868	2713	sp|P02786|TFR1_HUMAN	128.39	4.49E+06	2.69E+06	1.75E+06	5.01E+05	9	9	9	5	2	1	
1729	2925	sp|Q9NRZ9|HELLS_HUMAN	128.29	2.28E+06	3.60E+06	6.45E+06	1.16E+06	13	13	5	5	6	2	Carbamid
1729	2930	tr|A0A0B4J1V9|A0A0B4J1V9_HUMAN	128.29	2.28E+06	3.60E+06	6.45E+06	1.16E+06	13	13	5	5	6	2	Carbamid
2229	6024	sp|Q9H0U6|RM18_HUMAN	128.17		7.10E+05	5.32E+06	2.51E+06	4	4	0	1	2	1	
1867	2256	sp|O94885|SASH1_HUMAN	128.09	8.35E+06	2.88E+06	4.06E+05	3.53E+05	15	15	9	7	1	1	Carbamid
2111	4405	sp|Q05655|KPCD_HUMAN	128.01	2.34E+06	1.23E+05		5.00E+05	5	5	3	1	0	2	Carbamid
1948	5671	sp|O00299|CLIC1_HUMAN	128.01	3.49E+06	1.33E+06	3.85E+06	2.19E+06	5	5	4	2	3	3	
1655	2950	sp|P56545|CTBP2_HUMAN	127.95	1.68E+05		2.34E+05	3.04E+05	9	5	5	2	3	3	Carbamid
1655	2959	tr|Q5SQP8|Q5SQP8_HUMAN	127.95	1.68E+05		2.34E+05	3.04E+05	9	5	5	2	3	3	Carbamid
1801	5337	tr|B0QXZ6|B0QXZ6_HUMAN	127.82	1.29E+07	1.75E+07	8.55E+06	1.10E+07	5	4	4	4	4	5	Acetylatic
1801	5339	sp|O95503|CBX6_HUMAN	127.82	1.29E+07	1.75E+07	8.55E+06	1.10E+07	5	4	4	4	4	5	Acetylatic
1520	2811	sp|P07858|CATB_HUMAN	127.82	1.18E+07	5.53E+06	6.14E+06	2.04E+06	9	9	7	5	4	1	Carbamid
2017	7110	sp|P62310|LSM3_HUMAN	127.76	3.99E+05	1.68E+06	1.59E+06	3.38E+06	3	3	2	2	2	2	Acetylatic
1907	5218	sp|O60220|TIM8A_HUMAN	127.75	5.11E+06	4.85E+06	1.94E+06	2.42E+06	5	5	3	3	1	1	Carbamid
1813	7024	sp|O75348|VATG1_HUMAN	127.73	6.60E+06	4.29E+06	1.09E+06	2.04E+06	3	3	5	3	1	1	Acetylatic
2056	3718	sp|P30084|ECHM_HUMAN	127.65	3.26E+06	1.59E+06	2.62E+06		7	7	4	2	2	0	Carbamid
2057	4653	sp|P56693|SOX10_HUMAN	127.52	8.04E+05			3.63E+06	6	6	1	0	0	2	Carbamid
2057	4730	tr|A6PVD3|A6PVD3_HUMAN	127.52	8.04E+05			3.63E+06	6	6	1	0	0	2	Carbamid
1709	6462	sp|Q86WX3|AROS_HUMAN	127.49	6.12E+06	1.06E+07	7.61E+06	1.14E+07	6	6	3	4	4	4	
2053	3152	sp|P33981|TTK_HUMAN	127.44	8.41E+05	3.31E+05	1.27E+06		10	9	4	1	4	0	Carbamid
2555	7107	sp|Q6Y288|B3GLT_HUMAN	127.41	1.14E+06	8.06E+05	5.38E+05		3	3	2	2	1	0	
1720	5853	sp|O00165|HAX1_HUMAN	127.27		6.05E+06	1.76E+06	2.03E+06	4	4	0	4	2	2	
1731	2803	sp|P11216|PYGB_HUMAN	127.2	1.52E+06	1.05E+06	1.51E+06	1.08E+06	11	11	4	3	3	2	Carbamid
1864	2495	sp|O75116|ROCK2_HUMAN	126.86	1.66E+06	4.01E+05	1.21E+05		14	10	3	3	2	0	Carbamid
1657	3026	sp|Q9H2U2|IPYR2_HUMAN	126.74	3.41E+06	1.74E+06	1.73E+06	1.38E+06	9	9	3	2	3	2	Carbamid
1680	6267	sp|P49458|SRP09_HUMAN	126.66	6.12E+07	5.82E+07	9.07E+07	1.10E+08	4	4	4	4	3	3	Carbamid
1958	6048	tr|E7EWP0|E7EWP0_HUMAN	126.62	3.60E+06	7.01E+06	2.74E+06	3.48E+06	5	5	2	4	1	2	
1958	6050	sp|O43674|NDUB5_HUMAN	126.62	3.60E+06	7.01E+06	2.74E+06	3.48E+06	5	5	2	4	1	2	
1958	6355	tr|H0Y886|H0Y886_HUMAN	126.62	3.60E+06	7.01E+06	2.74E+06	3.48E+06	5	5	2	4	1	2	
2548	4468	sp|Q9BSJ8|ESYT1_HUMAN	126.43	1.14E+06				5	5	3	0	0	0	
2334	4914	sp|Q9BR76|COR1B_HUMAN	126.36		7.02E+05		8.18E+05	5	5	0	1	0	1	Carbamid
1403	3957	sp|Q15070|OXA1L_HUMAN	126.32	1.48E+07	2.63E+07	1.97E+07	1.80E+07	6	6	4	7	5	4	Carbamid
1403	3958	tr|S4R3Q9|S4R3Q9_HUMAN	126.32	1.48E+07	2.63E+07	1.97E+07	1.80E+07	6	6	4	7	5	4	Carbamid
1403	3963	tr|J3KNA0|J3KNA0_HUMAN	126.32	1.48E+07	2.63E+07	1.97E+07	1.80E+07	6	6	4	7	5	4	Carbamid
2240	3586	tr|E3W994|E3W994_HUMAN	126.26	2.09E+05	5.41E+06	2.02E+05		8	4	4	2	1	2	Carbamid
2240	3588	tr|J3KR49|J3KR49_HUMAN	126.26	2.09E+05	5.41E+06	2.02E+05		8	4	4	2	1	2	Carbamid
2240	3589	sp|O75122|CLAP2_HUMAN	126.26	2.09E+05	5.41E+06	2.02E+05		8	4	4	2	1	2	Carbamid
2240	3590	tr|A0A0U1RQI6|A0A0U1RQI6_HUMAN	126.26	2.09E+05	5.41E+06	2.02E+05		8	4	4	2	1	2	Carbamid
2240	3599	tr|E7ERI8|E7ERI8_HUMAN	126.26	2.09E+05	5.41E+06	2.02E+05		8	4	4	2	1	2	Carbamid
2240	3600	tr|E7EW49|E7EW49_HUMAN	126.26	2.09E+05	5.41E+06	2.02E+05		8	4	4	2	1	2	Carbamid
1762	3443	sp|P54803|GALC_HUMAN	126.15	7.98E+05	7.58E+05	8.94E+05	1.56E+06	7	7	3	3	2	4	
1835	3912	sp|O43913|ORC5_HUMAN	126.01	7.04E+05	1.59E+06	9.25E+05	1.19E+06	6	6	2	4	2	3	
1955	4615	sp|Q9BTL3|RAMAC_HUMAN	125.91	1.52E+07	1.65E+07			6	6	6	4	0	0	Acetylatic
1955	5172	tr|A0A3B3IU46|A0A3B3IU46_HUMAN	125.91	1.50E+07	1.65E+07			5	5	5	4	0	0	
2146	4425	sp|Q8WVC0|LEO1_HUMAN	125.86		5.14E+06	3.69E+06	2.99E+06	7	7	0	3	3	2	
2369	4295	sp|P31930|QCR1_HUMAN	125.74	1.23E+06	6.48E+04			6	6	5	1	0	0	Carbamid
1648	5180	sp|Q9NVA1|UQCC1_HUMAN	125.64	5.26E+06	5.19E+06	1.41E+06	5.43E+06	5	5	6	3	1	3	
1562	3819	sp|Q9NRX2|RM17_HUMAN	125.63	5.16E+06	4.03E+06	1.04E+07	1.05E+07	7	7	4	3	4	4	Carbamid
2164	7892	sp|Q9NZZ3|CHMP5_HUMAN	125.58	2.52E+06	1.61E+06		1.95E+06	3	3	2	1	0	2	
2020	7973	sp|Q5XKP0|MIC13_HUMAN	125.55	2.02E+06	3.24E+06	2.22E+06	5.69E+06	3	3	1	2	1	2	
2020	7975	tr|A0A140TA86|A0A140TA86_HUMAN	125.55	2.02E+06	3.24E+06	2.22E+06	5.69E+06	3	3	1	2	1	2	
1808	4826	sp|P83876|TXN4A_HUMAN	125.48	2.05E+06	5.96E+06	8.98E+05	4.51E+06	7	7	2	5	1	4	Acetylatic
2524	6465	sp|Q9NP66|HM20A_HUMAN	125.44				1.04E+05	5	5	0	0	0	1	Deamidat
2143	3713	tr|B7WPG3|B7WPG3_HUMAN	125.36	2.86E+05	2.75E+05	1.39E+06	6.94E+05	8	7	2	2	2	3	Carbamid
2143	3715	tr|C9IYN3|C9IYN3_HUMAN	125.36	2.86E+05	2.75E+05	1.39E+06	6.94E+05	8	7	2	2	2	3	Carbamid
2143	3716	sp|Q8WVV9|HNRLL_HUMAN	125.36	2.86E+05	2.75E+05	1.39E+06	6.94E+05	8	7	2	2	2	3	Carbamid
2143	3717	tr|D6W592|D6W592_HUMAN	125.36	2.86E+05	2.75E+05	1.39E+06	6.94E+05	8	7	2	2	2	3	Carbamid
1341	7612	tr|A0A075B6R9|A0A075B6R9_HUMAN	125.31			9.39E+06	4.82E+06	7	3	1	2	14	7	
1576	3079	sp|P10636|TAU_HUMAN	125.22	1.71E+07	6.55E+06	1.35E+06	8.30E+06	10	10	7	6	1	3	Deamidat
1576	3081	tr|A0A0G2JPD5|A0A0G2JPD5_HUMAN	125.22	1.71E+07	6.55E+06	1.35E+06	8.30E+06	10	10	7	6	1	3	Deamidat
1576	3089	tr|A0A0G2JQJ7|A0A0G2JQJ7_HUMAN	125.22	1.71E+07	6.55E+06	1.35E+06	8.30E+06	10	10	7	6	1	3	Deamidat
1668	6207	sp|O00483|NDUA4_HUMAN	125.18	2.16E+07	3.72E+07	2.47E+07	3.90E+07	4	4	4	4	4	4	Carbamid
2351	7603	sp|Q9BYC8|RM32_HUMAN	124.84	1.84E+06			2.88E+06	4	4	1	0	0	2	Carbamid
1430	3100	sp|P48444|COPD_HUMAN	124.79	3.51E+06	1.63E+06	7.39E+05	2.25E+06	7	7	8	5	1	4	Carbamid
1430	3103	tr|B0YIW6|B0YIW6_HUMAN	124.79	3.51E+06	1.63E+06	7.39E+05	2.25E+06	7	7	8	5	1	4	Carbamid
1748	4060	sp|O95363|SYFM_HUMAN	124.62	9.18E+05	3.06E+05	2.85E+06	4.91E+05	7	7	3	2	4	1	Carbamid
1748	4064	tr|A0A3B3ITR6|A0A3B3ITR6_HUMAN	124.62	9.18E+05	3.06E+05	2.85E+06	4.91E+05	7	7	3	2	4	1	Carbamid
1976	3364	tr|F8WA11|F8WA11_HUMAN	124.61	2.33E+05			5.85E+04	11	7	3	0	0	4	Carbamid
1976	3367	sp|Q7Z460|CLAP1_HUMAN	124.61	2.33E+05			5.85E+04	11	7	3	0	0	4	Carbamid
1773	3155	sp|Q9NQ29|LUC7L_HUMAN	124.49		2.55E+06	4.04E+06	1.39E+07	10	5	1	5	3	5	Carbamid
2487	7317	sp|P08493|MGP_HUMAN	124.43	5.35E+06	1.21E+06		1.60E+06	3	3	2	1	0	1	Oxidatior
1781	3391	sp|P17706|PTN2_HUMAN	124.36	1.41E+06	2.67E+06	2.16E+06		8	8	3	6	2	0	Carbamid
2249	4670	sp|Q8NEM2|SHCBP_HUMAN	124.31	3.24E+06	1.00E+06		6.73E+05	7	7	4	3	0	1	Carbamid
2046	4806	sp|P09496|CLCA_HUMAN	124.13	9.95E+05			3.34E+06	6	6	2	0	0	4	Carbamid
1775	3674	sp|Q9Y383|LC7L2_HUMAN	124.01	1.36E+06	2.80E+06	2.25E+06	3.42E+06	9	5	3	5	3	3	Carbamid
2443	4423	sp|Q96PZ0|PUS7_HUMAN	124.01					6	6	0	0	0	0	
2001	3304	tr|A0A3B3ITR4|A0A3B3ITR4_HUMAN	123.34	1.01E+06	6.74E+05	8.07E+05	5.69E+05	9	8	5	3	2	1	Carbamid
2052	3040	sp|Q9H3U1|UN45A_HUMAN	123.3	2.13E+06	2.23E+05		3.53E+05	9	9	1	1	0	1	
2052	3041	tr|A0A1W2PNX8|A0A1W2PNX8_HUMAN	123.3	2.13E+06	2.23E+05		3.53E+05	9	9	1	1	0	1	
1794	4065	sp|Q9ULW3|ABT1_HUMAN	123.05	2.28E+05	1.66E+06	5.99E+06	1.37E+07	6	6	1	2	3	6	
2013	5354	sp|Q9UJK0|TSR3_HUMAN	122.93	1.50E+06	1.13E+06	1.46E+06	1.77E+06	6	6	3	2	2	3	Carbamid

2263	4439	sp|Q86YP4|P66A_HUMAN	122.83	3.05E+05			2.34E+06	6	5	1	0	0	3	Deamidat
1733	3933	tr|F5GY37|F5GY37_HUMAN	122.8	3.63E+06	9.49E+05	3.02E+06	3.25E+06	7	7	5	3	2	4	
1733	3935	tr|J3KPX7|J3KPX7_HUMAN	122.8	3.63E+06	9.49E+05	3.02E+06	3.25E+06	7	7	5	3	2	4	
1733	3936	sp|Q99623|PHB2_HUMAN	122.8	3.63E+06	9.49E+05	3.02E+06	3.25E+06	7	7	5	3	2	4	
1999	4813	sp|Q6I9Y2|THOC7_HUMAN	122.72	2.22E+06	3.93E+06	1.39E+06	0	7	7	3	4	3	1	Carbamid
1642	6055	sp|Q96FJ2|DYL2_HUMAN	122.68	2.10E+06	3.98E+06	3.16E+06	6.44E+06	5	2	4	3	4	5	Carbamid
2079	4667	tr|B0QYW5|B0QYW5_HUMAN	122.54	5.92E+05	5.95E+05	6.63E+05		5	5	2	2	1	0	
2079	4668	tr|F6RTR7|F6RTR7_HUMAN	122.54	5.92E+05	5.95E+05	6.63E+05		5	5	2	2	1	0	
2079	4669	sp|O43808|PM34_HUMAN	122.54	5.92E+05	5.95E+05	6.63E+05		5	5	2	2	1	0	
1736	4645	sp|Q96HP4|OXND1_HUMAN	122.54	2.74E+06	2.18E+06	1.26E+06	2.60E+06	6	6	3	2	2	4	Deamidat
1736	4646	tr|C9JLB7|C9JLB7_HUMAN	122.54	2.74E+06	2.18E+06	1.26E+06	2.60E+06	6	6	3	2	2	4	Deamidat
1736	4777	tr|U3KQU8|U3KQU8_HUMAN	122.54	2.74E+06	2.18E+06	1.26E+06	2.60E+06	6	6	3	2	2	4	Deamidat
1597	3773	sp|P07948|LYN_HUMAN	122.47	7.31E+04			5.87E+04	7	4	4	4	2	3	
2048	3657	sp|Q6NUK1|SCMC1_HUMAN	122.28	2.27E+06	1.09E+06	1.59E+06	6.15E+05	9	9	4	2	3	1	
1837	2988	sp|Q6PII3|CC174_HUMAN	122.06	3.77E+06	1.23E+07		4.05E+06	9	9	5	8	0	3	
1837	3008	tr|A0A0B4J1R8|A0A0B4J1R8_HUMAN	122.06	3.77E+06	1.23E+07		4.05E+06	9	9	5	8	0	3	
2323	3735	sp|Q9UHV7|MED13_HUMAN	122.05	1.56E+05	1.43E+06		1.23E+06	7	7	1	3	0	4	Carbamid
1951	3992	tr|E7EQY4|E7EQY4_HUMAN	122.03		3.43E+05		3.09E+05	7	4	1	2	0	2	
1951	4002	tr|E7EV10|E7EV10_HUMAN	122.03		3.43E+05		3.09E+05	7	4	1	2	0	2	
1951	4005	sp|Q9BTC8|MTA3_HUMAN	122.03		3.43E+05		3.09E+05	7	4	1	2	0	2	
1951	4038	tr|E9PCS8|E9PCS8_HUMAN	122.03		3.43E+05		3.09E+05	7	4	1	2	0	2	
2331	8297	sp|Q15005|SPCS2_HUMAN	121.99	1.39E+06	2.19E+05		1.35E+05	4	4	2	2	0	1	
2331	8298	tr|A0A087WUC6|A0A087WUC6_HUMAN	121.99	1.39E+06	2.19E+05		1.35E+05	4	4	2	2	0	1	
2331	8299	tr|E9PI68|E9PI68_HUMAN	121.99	1.39E+06	2.19E+05		1.35E+05	4	4	2	2	0	1	
2331	8398	tr|E9PRB9|E9PRB9_HUMAN	121.99	1.39E+06	2.19E+05		1.35E+05	4	4	2	2	0	1	
2331	8399	tr|E9PL01|E9PL01_HUMAN	121.99	1.39E+06	2.19E+05		1.35E+05	4	4	2	2	0	1	
2010	5169	sp|Q9HCU8|DPOD4_HUMAN	121.91	3.69E+05	3.80E+06		1.94E+06	5	5	1	3	0	2	Carbamid
1742	3584	sp|Q9BV36|MELPH_HUMAN	121.72	1.02E+07	9.97E+06	1.10E+07	7.40E+06	7	7	5	6	4	3	Carbamid
1574	3449	sp|O75145|LIPA3_HUMAN	121.51	2.49E+05	1.43E+05			10	5	8	4	4	1	Carbamid
2214	3295	sp|Q9NS86|LANC2_HUMAN	121.48	3.07E+05		3.77E+05	2.99E+06	8	8	1	0	1	2	Carbamid
1841	3929	sp|Q9BSQ5|CCM2_HUMAN	121.41	1.78E+06	2.71E+06	5.33E+05	1.43E+06	6	6	4	5	1	2	
1841	4089	tr|C9JUH3|C9JUH3_HUMAN	121.41	1.78E+06	2.71E+06	5.33E+05	1.43E+06	6	6	4	5	1	2	
1841	4090	tr|A0A0A0MT72|A0A0A0MT72_HUMAN	121.41	1.78E+06	2.71E+06	5.33E+05	1.43E+06	6	6	4	5	1	2	
2253	4585	sp|Q70CQ3|UBP30_HUMAN	121.05	2.73E+05	8.95E+05		6.85E+05	6	6	1	2	0	4	Carbamid
2043	4611	sp|O43663|PRC1_HUMAN	120.62			3.88E+06	1.59E+06	7	6	1	1	5	4	Carbamid
1970	7585	sp|O60783|RT14_HUMAN	120.51	5.63E+06	1.00E+07	1.00E+07	1.91E+07	3	3	2	2	1	3	
2125	3607	sp|Q9P0K7|RAI14_HUMAN	120.51		6.15E+05		7.05E+05	7	7	0	2	0	1	Carbamid
1993	5368	sp|Q13825|AUHM_HUMAN	120.5	4.83E+06	1.21E+06	7.96E+05	8.87E+05	5	5	2	1	1	1	Carbamid
2521	5408	tr|A0A0C4DFT3|A0A0C4DFT3_HUMAN	120.47	1.93E+06	2.75E+05	4.60E+05		5	5	4	1	1	0	Carbamid
2521	5442	sp|Q12959|DLG1_HUMAN	120.47	1.93E+06	2.75E+05	4.60E+05		5	5	4	1	1	0	Carbamid
2816	6330	tr|A0A2Q3DQE3|A0A2Q3DQE3_HUMAN	120.44					3	1	0	1	0	0	
2816	6333	tr|Q8WU40|Q8WU40_HUMAN	120.44					3	1	0	1	0	0	
2816	6414	tr|Q5SWX3|Q5SWX3_HUMAN	120.44					3	1	0	1	0	0	
2816	6422	sp|Q13555|KCC2G_HUMAN	120.44					3	1	0	1	0	0	
2371	5373	tr|A6NJA2|A6NJA2_HUMAN	120.29	2.55E+06	1.68E+05	2.27E+05		4	4	3	1	1	0	Carbamid
2371	5374	sp|P54578|UBP14_HUMAN	120.29	2.55E+06	1.68E+05	2.27E+05		4	4	3	1	1	0	Carbamid
2515	5831	sp|Q9UHD1|CHRD1_HUMAN	120.18	2.24E+06				4	4	5	0	0	0	Carbamid
2377	8535	tr|E9PQR7|E9PQR7_HUMAN	120.13	1.24E+06	3.98E+05			2	2	3	1	0	0	Acetylatic
2377	8536	tr|E9PM90|E9PM90_HUMAN	120.13	1.24E+06	3.98E+05			2	2	3	1	0	0	Acetylatic
2377	8537	sp|Q9UK41|VPS28_HUMAN	120.13	1.24E+06	3.98E+05			2	2	3	1	0	0	Acetylatic
2377	9964	tr|E9PR04|E9PR04_HUMAN	120.13	1.24E+06	3.98E+05			2	2	3	1	0	0	Acetylatic
1779	4740	sp|Q14197|ICT1_HUMAN	120.1	5.20E+06	4.40E+06	1.04E+07	4.14E+06	6	6	4	3	3	2	Carbamid
1779	4775	tr|J3KS15|J3KS15_HUMAN	120.1	5.20E+06	4.40E+06	1.04E+07	4.14E+06	6	6	4	3	3	2	Carbamid
2064	4117	sp|Q8IYA6|CKP2L_HUMAN	120.1	1.42E+06	3.84E+06		4.09E+04	6	6	4	7	0	1	Carbamid
2203	6531	tr|K7EQ55|K7EQ55_HUMAN	120.03	1.16E+06	2.80E+06	2.74E+06	8.66E+05	4	4	1	2	3	1	Carbamid
2203	6532	tr|K7EK33|K7EK33_HUMAN	120.03	1.16E+06	2.80E+06	2.74E+06	8.66E+05	4	4	1	2	3	1	Carbamid
2203	6533	sp|Q96EP5|DAZP1_HUMAN	120.03	1.16E+06	2.80E+06	2.74E+06	8.66E+05	4	4	1	2	3	1	Carbamid
2459	6030	sp|Q96FX7|TRM61_HUMAN	120.01			3.42E+05	4.19E+05	5	5	0	0	1	1	Carbamid
1888	4616	sp|P15927|RFA2_HUMAN	119.94		1.66E+06	3.14E+06	5.78E+06	5	5	0	1	3	4	Carbamid
2267	4410	sp|Q13362|2A5G_HUMAN	119.89	1.75E+05	3.05E+05			5	3	2	1	0	0	Carbamid
2267	4411	tr|H0YJ75|H0YJ75_HUMAN	119.89	1.75E+05	3.05E+05			5	3	2	1	0	0	Carbamid
2267	4716	tr|Q96B13|Q96B13_HUMAN	119.89	1.75E+05	3.05E+05			5	3	2	1	0	0	Carbamid
2837	6982	sp|Q9NSU2|TREX1_HUMAN	119.85			7.73E+04	9.48E+05	3	3	0	0	1	1	Carbamid
1942	3248	sp|Q8IXI1|MIRO2_HUMAN	119.84	1.01E+05	3.66E+05	2.41E+05	6.03E+05	8	7	2	3	2	3	Carbamid
2388	7485	tr|E9PND3|E9PND3_HUMAN	119.74					4	4	0	0	0	0	
2388	7501	tr|E9PN10|E9PN10_HUMAN	119.74					4	4	0	0	0	0	
2388	7505	sp|O00559|RCAS1_HUMAN	119.74					4	4	0	0	0	0	
1722	7783	sp|P49006|MRP_HUMAN	119.7	2.75E+07	2.32E+07	1.78E+07	1.64E+07	3	3	3	3	3	3	
1738	2594	sp|Q8IUC4|RHPN2_HUMAN	119.7	5.43E+05	1.29E+06	5.38E+05		11	11	3	5	1	0	Carbamid
1453	4341	sp|P20339|RAB5A_HUMAN	119.63	4.49E+05	4.38E+05			6	2	7	9	2	2	
2425	5721	sp|Q8IV48|ERI1_HUMAN	119.6		2.14E+05	0	9.75E+05	4	4	0	1	1	1	Carbamid
2303	7787	tr|H3BPC4|H3BPC4_HUMAN	119.22	2.64E+06	1.71E+06	1.38E+06	1.97E+06	3	3	2	2	1	2	Carbamid
2303	7788	tr|H3BQQ9|H3BQQ9_HUMAN	119.22	2.64E+06	1.71E+06	1.38E+06	1.97E+06	3	3	2	2	1	2	Carbamid
2303	7789	sp|P63279|UBC9_HUMAN	119.22	2.64E+06	1.71E+06	1.38E+06	1.97E+06	3	3	2	2	1	2	Carbamid
2303	7790	tr|B0QYN7|B0QYN7_HUMAN	119.22	2.64E+06	1.71E+06	1.38E+06	1.97E+06	3	3	2	2	1	2	Carbamid
1832	6198	sp|P56385|ATP5I_HUMAN	119.16	8.45E+06	7.66E+06	1.46E+07	1.77E+07	4	4	3	3	2	3	
2083	5424	sp|Q6NS38|ALKB2_HUMAN	119.06	1.14E+06	2.70E+06	1.54E+06	2.09E+06	4	4	1	3	1	2	Carbamid
1981	5014	sp|Q6P1X5|TAF2_HUMAN	118.98	1.32E+05	4.43E+06		8.47E+05	6	6	1	5	0	2	Carbamid
1605	5545	sp|P62330|ARF6_HUMAN	118.93	7.70E+06	9.16E+06	7.70E+06	6.06E+06	5	5	4	5	3	3	
1949	3795	tr|B7Z6D5|B7Z6D5_HUMAN	118.82	1.81E+06	1.88E+06	3.52E+06	5.06E+06	6	6	2	2	2	5	
1949	3797	sp|Q96GQ7|DDX27_HUMAN	118.82	1.81E+06	1.88E+06	3.52E+06	5.06E+06	6	6	2	2	2	5	
2294	7115	sp|Q96KR6|F210B_HUMAN	118.76	3.78E+06	3.69E+06	2.86E+06		3	3	3	3	2	0	Carbamid
2157	7064	sp|P61769|B2MG_HUMAN	118.59	6.31E+06	3.92E+06		9.39E+06	3	3	2	2	0	3	Carbamid
1796	3319	sp|Q9UJW0|DCTN4_HUMAN	118.4	5.08E+05	3.32E+06	1.31E+06	3.25E+06	9	9	1	4	1	4	Carbamid
2101	5211	tr|Q5T8U5|Q5T8U5_HUMAN	118.35	1.46E+06	1.65E+06	8.01E+06	2.38E+06	5	5	1	1	2	2	Carbamid
2101	5213	sp|O15260|SURF4_HUMAN	118.35	1.46E+06	1.65E+06	8.01E+06	2.38E+06	5	5	1	1	2	2	Carbamid
2683	8249	sp|Q9Y333|LSM2_HUMAN	118.29				1.22E+06	3	3	0	0	0	2	Carbamid
1917	3837	sp|O00267|SPT5H_HUMAN	118.27	1.48E+06	8.37E+05	8.20E+05	8.38E+05	10	10	7	3	3	2	
2098	6331	tr|K7EIY1|K7EIY1_HUMAN	117.96	6.34E+06	9.81E+06	5.76E+06	1.08E+06	4	4	3	4	3	3	Acetylatic
2098	6332	sp|Q9Y421|FA32A_HUMAN	117.96	6.34E+06	9.81E+06	5.76E+06	1.08E+06	4	4	3	4	3	3	Acetylatic
1529	7497	sp|P42677|RS27_HUMAN	117.81	7.05E+06	1.47E+07	1.28E+07	1.21E+07	5	2	4	6	3	4	Carbamid
2343	4602	sp|Q6NW34|NEPRO_HUMAN	117.8			2.41E+06	1.07E+06	7	7	0	0	3	3	Carbamid
2019	7663	sp|Q9NYP7|ELOV5_HUMAN	117.49	7.02E+06	5.48E+06	1.04E+07	4.69E+06	3	3	4	2	2	2	Carbamid
1933	5394	sp|Q9BRG1|VPS25_HUMAN	117.45	2.86E+06	5.04E+05	1.73E+06		6	6	5	2	2	0	
2012	6018	sp|Q8NAT1|PMGT2_HUMAN	117.23	2.35E+05	6.32E+05	6.40E+05	4.49E+06	5	5	1	2	2	5	
1301	2514	sp|O00186|STXB3_HUMAN	117.22	2.63E+06	8.91E+05	1.61E+06	1.64E+06	11	10	6	3	4	4	Carbamid
2155	5594	sp|Q6IN84|MRM1_HUMAN	117.04			1.62E+06	1.52E+06	5	5	0	0	3	2	Carbamid
2599	5785	sp|O60216|RAD21_HUMAN	117.03					5	5	0	0	0	0	Carbamid
1727	3090	sp|P55072|TERA_HUMAN	117.02	4.87E+05	1.24E+06	8.76E+05	3.23E+06	11	10	1	4	2	5	Carbamid
1311	3078	sp|Q9ULF5|S39AA_HUMAN	116.99	2.03E+07	4.78E+06	1.29E+07	4.74E+06	12	12	9	2	6	3	Carbamid
1540	3850	sp|Q6N069|NAA16_HUMAN	116.88	6.57E+05	5.49E+05	1.15E+06	1.94E+05	12	8	6	4	5	2	
1764	7123	sp|Q9P0S2|COX16_HUMAN	116.6	5.19E+06	8.55E+06	2.89E+06	5.54E+06	4	4	3	4	1	3	
2119	4368	tr|C9JNK6|C9JNK6_HUMAN	116.54		3.62E+06	6.60E+05	1.53E+06	7	7	0	3	1	1	Carbamid
2119	4369	sp|O75431|MTX2_HUMAN	116.54		3.62E+06	6.60E+05	1.53E+06	7	7	0	3	1	1	Carbamid
2039	3400	sp|Q99832|TCPH_HUMAN	116.44	1.47E+06	1.52E+06	9.20E+05		8	8	4	3	1	0	Carbamid
2373	6300	sp|Q99805|TM9S2_HUMAN	116.43	1.12E+06	7.93E+04			5	5	2	1	0	0	Carbamid
2342	8274	sp|Q9Y6N1|COX11_HUMAN	116.25	7.79E+05	8.21E+05	1.38E+06		3	3	2	1	1	0	Carbamid
1573	3545	sp|Q9H2U1|DHX36_HUMAN	116.25	2.63E+05		3.07E+06	1.13E+06	11	9	2	0	9	5	Carbamid
2092	5642	tr|E9PES6|E9PES6_HUMAN	116.07	4.95E+06				4	3	3	1	1	1	Carbamid
2092	5645	tr|E7ES08|E7ES08_HUMAN	116.07	4.95E+06				4	3	3	1	1	1	Carbamid
2092	5648	tr|E7EQU1|E7EQU1_HUMAN	116.07	4.95E+06				4	3	3	1	1	1	Carbamid
2092	5649	sp|O15347|HMGB3_HUMAN	116.07	4.95E+06				4	3	3	1	1	1	Carbamid
1931	5515	sp|Q16655|MAR1_HUMAN	115.88	4.71E+06	1.12E+06	1.17E+06	1.27E+06	5	5	5	2	2	3	Carbamid
2290	5695	sp|Q96DP5|FMT_HUMAN	115.85	1.14E+05	3.34E+05	9.34E+05	9.79E+05	4	4	2	2	1	1	Carbamid
1846	3116	sp|P07384|CAN1_HUMAN	115.52	1.72E+06	5.09E+05	7.84E+05	2.96E+05	10	10	3	2	3	1	Carbamid
1865	4817	sp|Q6DKI1|RL7L_HUMAN	115.41	3.90E+05	1.04E+06	6.27E+06	8.43E+06	6	6	1	1	3	4	

1865	4832	tr|R4GMU7|R4GMU7_HUMAN	115.41	3.90E+05	1.04E+06	6.27E+06	8.43E+06	6	6	1	1	3	4	
2392	5443	sp|P24666|PPAC_HUMAN	115.31	8.17E+06	3.15E+06	2.86E+06		5	5	4	2	2	0	Carbamid
2587	7714	tr|E9PQL1|E9PQL1_HUMAN	115.3	1.32E+06	1.92E+06		1.94E+06	3	3	1	1	0	3	Carbamid
2587	7716	sp|Q8TAV0|FA76A_HUMAN	115.3	1.32E+06	1.92E+06		1.94E+06	3	3	1	1	0	3	Carbamid
2175	3753	sp|P49642|PRI1_HUMAN	115.03	2.72E+05		4.04E+05		7	7	2	0	1	0	Carbamid
1908	5347	sp|P35610|SOAT1_HUMAN	115.01	5.10E+05	6.65E+05	1.16E+06	2.60E+05	5	5	1	1	3	1	Carbamid
2234	7338	sp|O43504|LTOR5_HUMAN	115.01	1.58E+06	1.44E+06		5.36E+05	3	3	4	3	0	1	Carbamid
2103	9997	sp|Q96IX5|USMG5_HUMAN	114.95	4.84E+06	7.12E+06	1.30E+07	1.11E+07	2	2	1	2	2	2	
1897	5361	sp|Q7Z7H8|RM10_HUMAN	114.91	7.48E+04	1.29E+06	4.29E+05	3.31E+06	6	6	1	1	2	1	Carbamid
2244	4031	sp|O14802|RPC1_HUMAN	114.9			8.87E+05	8.02E+05	7	6	0	0	1	3	Carbamid
2457	4679	tr|F8W914|F8W914_HUMAN	114.89	7.00E+05	1.79E+06			5	5	1	2	0	0	Carbamid
2016	6019	tr|H3BNT4|H3BNT4_HUMAN	114.81			7.19E+06	7.73E+06	5	5	0	0	2	7	
2016	6023	sp|Q99547|MPH6_HUMAN	114.81			7.19E+06	7.73E+06	5	5	0	0	2	7	
2102	7194	sp|Q92685|ALG3_HUMAN	114.46	8.00E+05	1.22E+06	4.36E+06	3.23E+06	3	3	1	1	2	2	Carbamid
2091	5473	sp|Q9BWG6|SCNM1_HUMAN	114.36	1.55E+06	2.48E+06	1.86E+06	1.75E+06	6	6	3	3	2	3	
2153	4744	sp|Q9NP64|NO40_HUMAN	114.31	9.02E+05	3.49E+06	3.14E+06	1.39E+06	5	5	1	2	3	2	Carbamid
2153	4745	tr|A0A0D9SEW3|A0A0D9SEW3_HUMAN	114.31	9.02E+05	3.49E+06	3.14E+06	1.39E+06	5	5	1	2	3	2	Carbamid
2153	4746	tr|A0A087WXF8|A0A087WXF8_HUMAN	114.31	9.02E+05	3.49E+06	3.14E+06	1.39E+06	5	5	1	2	3	2	Carbamid
2117	4419	sp|P21283|VATC1_HUMAN	114.3	2.66E+05	8.51E+04	1.34E+05	3.00E+05	6	6	1	1	1	2	Carbamid
2187	5154	sp|Q9BU61|NDUF3_HUMAN	114.16	2.82E+06			2.90E+05	5	5	3	0	0	1	
2011	5190	sp|Q9UPT9|UBP22_HUMAN	114.14	1.09E+06	9.80E+05	4.81E+05	4.87E+05	8	8	2	2	1	1	Carbamid
2054	3268	sp|O15226|NKRF_HUMAN	113.71		7.00E+05	1.94E+06	4.77E+06	7	7	0	2	2	6	Carbamid
1363	3489	sp|P08754|GNAI3_HUMAN	113.46			1.23E+06		7	1	5	4	7	4	Carbamid
2231	7333	sp|O14735|CDIPT_HUMAN	113.25	2.10E+06	2.76E+06	1.72E+06	2.27E+06	3	3	2	2	1	1	
2231	7334	tr|B3KY94|B3KY94_HUMAN	113.25	2.10E+06	2.76E+06	1.72E+06	2.27E+06	3	3	2	2	1	1	
2518	6899	tr|E7ERS3|E7ERS3_HUMAN	113.17	1.35E+07	7.53E+06		1.74E+06	4	4	3	1	0	2	
2518	7008	sp|Q86VM9|ZCH18_HUMAN	113.17	1.35E+07	7.53E+06		1.74E+06	4	4	3	1	0	2	
2266	4299	sp|Q9NUJ1|ABHDA_HUMAN	113.14	1.40E+06	4.77E+05	5.18E+05	1.34E+05	6	6	4	1	1	1	Carbamid
2312	6670	sp|Q92747|ARC1A_HUMAN	113.1					5	1	1	3	1	1	Carbamid
2376	7426	sp|O95167|NDUA3_HUMAN	113.09		1.03E+06	1.92E+06		3	3	0	1	1	0	
2376	7427	tr|Q6FGG4|Q6FGG4_HUMAN	113.09		1.03E+06	1.92E+06		3	3	0	1	1	0	
2139	4539	sp|P51858|HDGF_HUMAN	113.01	7.49E+06	1.40E+06			7	6	5	4	0	1	Carbamid
2271	5960	sp|O75554|WBP4_HUMAN	112.82	7.28E+05	4.00E+06		9.37E+05	5	5	1	6	0	1	Carbamid
1895	3796	tr|A0A0C4DGX4|A0A0C4DGX4_HUMAN	112.81	4.12E+05	4.21E+05	2.86E+05	2.05E+05	7	6	3	3	2	2	Deamidat
1895	3799	sp|Q13616|CUL1_HUMAN	112.81	4.12E+05	4.21E+05	2.86E+05	2.05E+05	7	6	3	3	2	2	Deamidat
1774	3664	sp|O43633|CHM2A_HUMAN	112.76	1.02E+06	1.39E+06		2.88E+06	7	7	2	2	0	3	Acetylatic
2233	6932	sp|Q9UJZ1|STML2_HUMAN	112.27	4.84E+05	4.54E+05		3.21E+05	4	4	3	2	0	2	Deamidat
1783	3329	tr|A0A087WWS1|A0A087WWS1_HUMAN	112.16	1.25E+06	6.82E+06	5.44E+05	1.37E+06	10	10	3	9	1	2	Carbamid
1783	3330	sp|Q96FV9|THOC1_HUMAN	112.16	1.25E+06	6.82E+06	5.44E+05	1.37E+06	10	10	3	9	1	2	Carbamid
2422	4147	tr|A0A2R8YD50|A0A2R8YD50_HUMAN	112.12	9.06E+04	1.70E+05			7	7	1	1	0	0	
2422	4149	sp|P51659|DHB4_HUMAN	112.12	9.06E+04	1.70E+05			7	7	1	1	0	0	
1972	2934	sp|Q14204|DYHC1_HUMAN	112.02	7.09E+05	1.62E+05	3.89E+04		11	11	2	2	1	0	
1761	5308	sp|P51151|RAB9A_HUMAN	111.89	1.43E+06	1.72E+06	1.85E+06	1.70E+06	5	4	2	3	1	2	
2227	4751	sp|Q9NUP9|LIN7C_HUMAN	111.81	6.06E+06	1.25E+06	6.84E+05		6	6	5	1	1	0	
2227	4862	tr|G3V1D4|G3V1D4_HUMAN	111.81	6.06E+06	1.25E+06	6.84E+05		6	6	5	1	1	0	
2418	5482	sp|O75175|CNOT3_HUMAN	111.73	1.68E+05		2.47E+05		5	5	2	0	1	0	Deamidat
2418	5497	tr|B7Z6J7|B7Z6J7_HUMAN	111.73	1.68E+05		2.47E+05		5	5	2	0	1	0	Deamidat
2647	5331	sp|Q9NPF5|DMAP1_HUMAN	111.64	1.69E+06		1.52E+06	8.81E+04	6	6	2	0	2	1	
2647	5375	tr|Q5TG40|Q5TG40_HUMAN	111.64	1.69E+06		1.52E+06	8.81E+04	6	6	2	0	2	1	
2647	5377	tr|Q5TG39|Q5TG39_HUMAN	111.64	1.69E+06		1.52E+06	8.81E+04	6	6	2	0	2	1	
2647	5378	tr|Q5TG38|Q5TG38_HUMAN	111.64	1.69E+06		1.52E+06	8.81E+04	6	6	2	0	2	1	
2251	4959	sp|Q3YEC7|RABL6_HUMAN	111.49	5.00E+05	1.59E+06	1.74E+06		5	5	2	4	2	0	Carbamid
2038	5415	sp|O43583|DENR_HUMAN	111.46	3.36E+06	6.88E+05	1.34E+06	4.01E+05	5	5	5	1	1	1	Carbamid
2184	3293	sp|Q99798|ACON_HUMAN	111.36	2.97E+05	1.31E+06		7.30E+05	9	9	1	4	0	2	
2184	3294	tr|A2A274|A2A274_HUMAN	111.36	2.97E+05	1.31E+06		7.30E+05	9	9	1	4	0	2	
1715	4753	sp|Q00535|CDK5_HUMAN	111.31	2.26E+06	3.02E+05	1.76E+06		5	3	5	4	3	2	
1987	3328	tr|A0A1B0GUI1|A0A1B0GUI1_HUMAN	111.23	6.05E+06		1.24E+06	4.79E+06	9	9	4	0	3	4	Carbamid
1650	5358	sp|P53985|MOT1_HUMAN	111.22	1.45E+06		7.19E+06	4.81E+06	5	4	3	1	4	3	
1759	5968	sp|P19013|K2C4_HUMAN	111.09					5	1	3	4	3	1	
2374	6411	tr|I3L2Z5|I3L2Z5_HUMAN	111.08	2.79E+06	2.15E+06		5.93E+05	4	4	4	2	0	2	Carbamid
2374	6413	tr|A0A087WWR2|A0A087WWR2_HUMAN	111.08	2.79E+06	2.15E+06		5.93E+05	4	4	4	2	0	2	Carbamid
2374	6421	sp|P56270|MAZ_HUMAN	111.08	2.79E+06	2.15E+06		5.93E+05	4	4	4	2	0	2	Carbamid
1607	10026	sp|Q9BQ48|RM34_HUMAN	110.9	1.91E+07	3.93E+07	1.30E+07	2.90E+07	3	3	3	4	5	6	Deamidat
1607	10034	tr|M0R226|M0R226_HUMAN	110.9	1.91E+07	3.93E+07	1.30E+07	2.90E+07	3	3	3	4	5	6	Deamidat
2562	5740	sp|Q96ES7|SGF29_HUMAN	110.87	2.06E+05	1.89E+05	5.03E+05	5.83E+05	5	5	1	1	1	1	
2435	4807	sp|Q13303|KCAB2_HUMAN	110.7			0	3.05E+05	5	5	0	0	1	1	Carbamid
2170	4406	sp|Q96KR1|ZFR_HUMAN	110.57	6.52E+06	3.66E+06	5.58E+06	1.69E+06	6	6	5	2	3	3	Carbamid
1924	5362	sp|Q08AD1|CAMP2_HUMAN	110.53	1.68E+06	1.45E+06	1.39E+06	1.69E+06	4	4	3	3	3	3	
2081	6328	sp|Q9BSV6|SEN34_HUMAN	110.53	1.40E+05	9.68E+05	1.38E+05	3.68E+05	4	4	1	4	1	1	
2081	6329	tr|E7EQB3|E7EQB3_HUMAN	110.53	1.40E+05	9.68E+05	1.38E+05	3.68E+05	4	4	1	4	1	1	
2088	3892	sp|O75439|MPPB_HUMAN	110.38	8.36E+05	8.47E+05		2.49E+05	6	6	1	5	0	1	Carbamid
1881	3117	sp|Q15050|RRS1_HUMAN	110.34	1.19E+06	6.56E+05	4.91E+06	3.21E+06	9	9	3	1	7	6	Carbamid
2127	4525	sp|Q6P1M0|S27A4_HUMAN	110.33	3.59E+05	2.76E+05	5.79E+05	4.97E+06	6	6	2	1	1	4	
2185	4415	tr|B7Z7P8|B7Z7P8_HUMAN	110.32	3.32E+06	4.01E+05	0		7	7	5	1	1	0	Carbamid
2185	4418	sp|P62495|ERF1_HUMAN	110.32	3.32E+06	4.01E+05	0		7	7	5	1	1	0	Carbamid
2018	7214	sp|Q9UHV9|PFD2_HUMAN	110.21	2.66E+06	2.78E+06	0	3.34E+06	3	3	2	2	1	2	
1950	3822	tr|A0A1W2PP57|A0A1W2PP57_HUMAN	110.2	1.60E+06	3.35E+06		2.31E+06	8	8	3	4	0	2	Carbamid
1950	3823	tr|A0A1W2PPC3|A0A1W2PPC3_HUMAN	110.2	1.60E+06	3.35E+06		2.31E+06	8	8	3	4	0	2	Carbamid
1950	3826	tr|A0A1W2PPS0|A0A1W2PPS0_HUMAN	110.2	1.60E+06	3.35E+06		2.31E+06	8	8	3	4	0	2	Carbamid
1950	3827	sp|Q969N2|PIGT_HUMAN	110.2	1.60E+06	3.35E+06		2.31E+06	8	8	3	4	0	2	Carbamid
1950	3828	tr|A0A1W2PNP0|A0A1W2PNP0_HUMAN	110.2	1.60E+06	3.35E+06		2.31E+06	8	8	3	4	0	2	Carbamid
2415	4078	sp|Q9BQ70|TCF25_HUMAN	109.91	8.07E+04	2.92E+05		1.81E+04	7	7	1	1	0	1	Carbamid
2254	5952	sp|Q9HCS7|SYF1_HUMAN	109.89			9.07E+05	2.54E+05	5	5	0	0	4	1	Carbamid
1927	3523	sp|Q96RS6|NUDC1_HUMAN	109.89	2.68E+06	1.03E+06	9.74E+05	4.19E+05	8	8	7	3	2	1	Carbamid
2592	8906	sp|P36954|RPB9_HUMAN	109.81	5.37E+05	6.31E+05	1.04E+06		2	2	1	1	2	0	Acetylatic
2592	9952	tr|K7EKS1|K7EKS1_HUMAN	109.81	5.37E+05	6.31E+05	1.04E+06		2	2	1	1	2	0	Acetylatic
1628	3146	tr|A0A0A0MTB8|A0A0A0MTB8_HUMAN	109.67	3.12E+04	2.83E+05	5.53E+06	4.15E+06	10	10	1	2	4	4	Deamidat
1628	3150	sp|Q8NI36|WDR36_HUMAN	109.67	3.12E+04	2.83E+05	5.53E+06	4.15E+06	10	10	1	2	4	4	Deamidat
1971	10170	sp|Q9UDW1|QCR9_HUMAN	109.57	3.48E+07	3.94E+07	5.46E+07	5.55E+07	2	2	2	3	2	2	
2216	6268	sp|Q15390|MTFR1_HUMAN	109.48	4.99E+04	2.12E+06	3.09E+05		5	5	1	4	1	0	Carbamid
1746	4480	sp|Q8NBS9|TXND5_HUMAN	109.42	3.42E+06	2.07E+06	1.10E+06	1.13E+06	7	7	5	3	2	2	Carbamid
1346	4120	tr|E9PCW1|E9PCW1_HUMAN	109.35	9.71E+05	1.90E+06		3.16E+06	6	6	4	5	0	2	Carbamid
1346	4121	sp|O95249|GOSR1_HUMAN	109.35	9.71E+05	1.90E+06		3.16E+06	6	6	4	5	0	2	Carbamid
1719	7511	sp|P60468|SC61B_HUMAN	109.14	1.70E+07	2.08E+07	2.01E+07	3.77E+07	3	3	3	3	5	3	Oxidation
1830	5382	sp|Q9H875|PKRI1_HUMAN	109.06	4.57E+06	1.02E+07	9.41E+05	6.87E+06	5	5	5	5	1	4	
1929	3313	sp|O94804|STK10_HUMAN	108.97	1.95E+06	2.70E+06		7.95E+05	8	6	5	3	0	1	Carbamid
1792	4603	sp|Q8WV74|NUDT8_HUMAN	108.96	4.06E+05	3.41E+06	1.90E+06	3.35E+06	8	8	1	5	2	2	Carbamid
2478	4404	sp|P29508|SPB3_HUMAN	108.83					7	7	0	0	0	0	
1964	6777	tr|H3BMM9|H3BMM9_HUMAN	108.81		5.98E+05	3.25E+06	4.62E+06	3	3	0	1	3	4	
1964	6779	tr|H3BTC0|H3BTC0_HUMAN	108.81		5.98E+05	3.25E+06	4.62E+06	3	3	0	1	3	4	
1964	6780	sp|Q15287|RNPS1_HUMAN	108.81		5.98E+05	3.25E+06	4.62E+06	3	3	0	1	3	4	
1964	6966	tr|H3BPG5|H3BPG5_HUMAN	108.81		5.98E+05	3.25E+06	4.62E+06	3	3	0	1	3	4	
1964	6967	tr|H3BV80|H3BV80_HUMAN	108.81		5.98E+05	3.25E+06	4.62E+06	3	3	0	1	3	4	
1965	10088	tr|H0Y8D1|H0Y8D1_HUMAN	108.81	1.52E+07	1.28E+07	1.15E+07	1.15E+07	3	3	2	2	2	2	Deamidat
1965	10091	sp|P07477|TRY1_HUMAN	108.81	1.52E+07	1.28E+07	1.15E+07	1.15E+07	3	3	2	2	2	2	Deamidat
1965	10093	tr|E7EQ64|E7EQ64_HUMAN	108.81	1.52E+07	1.28E+07	1.15E+07	1.15E+07	3	3	2	2	2	2	Deamidat
2006	3483	sp|Q06203|PUR1_HUMAN	108.76	2.51E+06	8.51E+05	1.67E+06		8	8	4	3	1	0	Carbamid
2268	4437	tr|A0A087X054|A0A087X054_HUMAN	108.59	2.79E+05	4.27E+05			6	6	3	2	0	0	Carbamid
2268	4438	sp|Q9Y4L1|HYOU1_HUMAN	108.59	2.79E+05	4.27E+05			6	6	3	2	0	0	Carbamid
2268	4456	tr|E9PJ21|E9PJ21_HUMAN	108.59	2.79E+05	4.27E+05			6	6	3	2	0	0	Carbamid
2268	4457	tr|A0A087WWI4|A0A087WWI4_HUMAN	108.59	2.79E+05	4.27E+05			6	6	3	2	0	0	Carbamid
2268	4458	tr|K7EQK2|K7EQK2_HUMAN	108.59	2.79E+05	4.27E+05			6	6	3	2	0	0	Carbamid
2698	8799	sp|Q5SRD1|TI23B_HUMAN	108.59			2.41E+06		2	2	0	0	1	0	
2698	9483	sp|O14925|TIM23_HUMAN	108.59			2.41E+06		2	2	0	0	1	0	
2961	9957	tr|Q5T3N1|Q5T3N1_HUMAN	108.5	1.02E+06	1.16E+05			2	2	2	1	0	0	
2961	9959	sp|P04083|ANXA1_HUMAN	108.5	1.02E+06	1.16E+05			2	2	2	1	0	0	

2109	4560	sp|Q9H267|VP33B_HUMAN	108.5	3.66E+06	1.32E+06		5.97E+05	8	8	7	3	0	1	Carbamid
2109	4572	tr|F5H008|F5H008_HUMAN	108.5	3.66E+06	1.32E+06		5.97E+05	8	8	7	3	0	1	Carbamid
2109	4613	tr|A0A2R8YDQ0|A0A2R8YDQ0_HUMAN	108.5	3.46E+06	1.32E+06		5.97E+05	7	7	6	3	0	1	Carbamid
2707	7504	sp|P25789|PSA4_HUMAN	108.47	1.71E+06	2.14E+05			3	3	3	1	0	0	Carbamid
2707	7627	tr|H0YMZ1|H0YMZ1_HUMAN	108.47	1.71E+06	2.14E+05			3	3	3	1	0	0	Carbamid
2707	7628	tr|H0YL69|H0YL69_HUMAN	108.47	1.71E+06	2.14E+05			3	3	3	1	0	0	Carbamid
2707	7936	tr|H0YMI6|H0YMI6_HUMAN	108.47	1.71E+06	2.14E+05			3	3	3	1	0	0	Carbamid
2707	7937	tr|H0YMA1|H0YMA1_HUMAN	108.47	1.71E+06	2.14E+05			3	3	3	1	0	0	Carbamid
2707	7985	tr|H0YLC2|H0YLC2_HUMAN	108.47	1.71E+06	2.14E+05			3	3	3	1	0	0	Carbamid
1632	4977	sp|Q9BYC9|RM20_HUMAN	108.36	9.22E+05	2.54E+06	6.53E+06	1.03E+07	5	5	2	2	3	6	
2182	3630	sp|Q9H2G2|SLK_HUMAN	108.14	4.95E+06	4.24E+05	1.90E+06	1.84E+06	7	5	4	1	1	2	Carbamid
2563	5742	tr|A0A2R8YFH5|A0A2R8YFH5_HUMAN	107.96	2.20E+04			2.28E+04	4	1	2	0	0	1	Carbamid
2563	5743	sp|Q15437|SC23B_HUMAN	107.96	2.20E+04			2.28E+04	4	1	2	0	0	1	Carbamid
2563	5821	tr|Q5QPE2|Q5QPE2_HUMAN	107.96	2.20E+04			2.28E+04	4	1	2	0	0	1	Carbamid
2086	4810	sp|P78386|KRT85_HUMAN	107.94					5	1	1	2	1	1	Carbamid
1308	12651	sp|P62891|RL39_HUMAN	107.83	3.57E+08	4.20E+08	9.14E+08	7.01E+08	6	6	5	6	5	7	Deamidat
1308	12652	sp|Q59GN2|R39L5_HUMAN	107.83	3.57E+08	4.20E+08	9.14E+08	7.01E+08	6	6	5	6	5	7	Deamidat
2469	5924	sp|P05423|RPC4_HUMAN	107.66			2.64E+05	5.85E+05	4	4	0	0	1	1	Carbamid
2526	7841	sp|Q8NCF5|NF2IP_HUMAN	107.63	8.25E+05	8.14E+05			3	3	1	1	0	0	
2363	7820	sp|Q9H649|NSUN3_HUMAN	107.52		3.13E+05	5.70E+05		3	3	0	2	2	0	
1674	5619	sp|P31947|1433S_HUMAN	107.26					5	1	3	3	3	5	Oxidatio
1989	4676	tr|A0A3B3IT37|A0A3B3IT37_HUMAN	107.23	1.44E+05	4.66E+05		1.51E+06	7	5	2	2	1	3	Carbamid
1989	4705	sp|Q8IYK4|GT252_HUMAN	107.23	1.44E+05	4.66E+05		1.51E+06	7	5	2	2	1	3	Carbamid
1484	6571	sp|Q15286|RAB35_HUMAN	107.23			1.43E+07		4	2	4	4	8	3	Deamidat
2133	6010	sp|Q99733|NP1L4_HUMAN	107.09		4.56E+05			5	2	0	2	2	2	Carbamid
1732	3203	tr|E9PGT3|E9PGT3_HUMAN	106.9	7.77E+05	1.56E+05		2.09E+05	10	5	8	2	4	2	Carbamid
1732	3204	sp|Q15418|KS6A1_HUMAN	106.9	7.77E+05	1.56E+05		2.09E+05	10	5	8	2	4	2	Carbamid
2113	3221	sp|Q92888|ARHG1_HUMAN	106.88	2.10E+06	4.30E+05	1.04E+06		10	10	4	1	1	0	Carbamid
2113	3222	tr|M0QZR4|M0QZR4_HUMAN	106.88	2.10E+06	4.30E+05	1.04E+06		10	10	4	1	1	0	Carbamid
2078	4638	tr|A0A087X106|A0A087X106_HUMAN	106.88					6	2	1	2	1	1	Carbamid
2078	4639	sp|O43790|KRT86_HUMAN	106.88					6	2	1	2	1	1	Carbamid
2078	4648	sp|Q14533|KRT81_HUMAN	106.88					6	2	1	2	1	1	Carbamid
1848	4827	tr|B4E1G1|B4E1G1_HUMAN	106.82	3.33E+06	5.43E+06	4.36E+06	3.98E+06	7	7	2	4	1	3	Acetylatio
1848	4858	sp|Q9BUN8|DERL1_HUMAN	106.82	3.33E+06	5.43E+06	4.36E+06	3.98E+06	7	7	2	4	1	3	Acetylatio
2245	4863	sp|P14923|PLAK_HUMAN	106.78	4.08E+05				5	2	1	0	0	1	Carbamid
2100	11206	tr|H0YAG5|H0YAG5_HUMAN	106.61	4.71E+06	5.30E+06	1.67E+06	6.33E+06	3	3	2	2	1	2	Carbamid
2100	11207	tr|D6RCM2|D6RCM2_HUMAN	106.61	4.71E+06	5.30E+06	1.67E+06	6.33E+06	3	3	2	2	1	2	Carbamid
2100	11208	sp|Q9Y3D5|RT18C_HUMAN	106.61	4.71E+06	5.30E+06	1.67E+06	6.33E+06	3	3	2	2	1	2	Carbamid
2356	6534	sp|O75792|RNH2A_HUMAN	106.6	1.01E+06	1.86E+06	4.87E+05	1.46E+06	5	3	1	2	2	1	Carbamid
2186	4800	sp|Q96ST2|IWS1_HUMAN	106.59	6.44E+06	2.25E+06	2.32E+06	2.01E+06	5	5	4	1	2	2	Carbamid
2230	6326	sp|O95478|NSA2_HUMAN	106.41	1.40E+07	1.05E+07	8.20E+06	2.03E+06	4	4	2	1	3	2	Carbamid
1664	3269	sp|Q9BTE3|MCMBP_HUMAN	106.38	4.01E+06	1.25E+06	1.19E+06	9.70E+05	10	10	7	3	3	2	Carbamid
2333	4854	sp|Q96DZ1|ERLEC_HUMAN	106.27	1.68E+06	1.84E+06			5	5	2	3	0	0	
2375	7164	tr|K7ENV7|K7ENV7_HUMAN	106.27	2.77E+05	0	6.17E+05		3	3	2	1	1	0	Carbamid
2375	7165	tr|K7EKW4|K7EKW4_HUMAN	106.27	2.77E+05	0	6.17E+05		3	3	2	1	1	0	Carbamid
2375	7166	sp|Q96AB3|ISOC2_HUMAN	106.27	2.77E+05	0	6.17E+05		3	3	2	1	1	0	Carbamid
1660	4069	sp|P82914|RT15_HUMAN	106.25	6.29E+05	4.17E+06	6.12E+06	1.02E+07	7	7	1	3	5	5	
1954	4092	sp|O75131|CPNE3_HUMAN	106.22	7.12E+05	5.78E+05	3.91E+05	3.79E+05	7	6	3	2	2	2	Carbamid
1945	4453	sp|Q9NVS2|RT18A_HUMAN	106.2	1.38E+06	7.59E+05	9.19E+06	5.58E+06	7	7	1	2	4	3	Carbamid
2609	8580	sp|Q5JTJ3|COA6_HUMAN	106.1	4.45E+06	1.76E+06			2	2	2	1	0	0	Carbamid
2609	8581	tr|X6R5Z6|X6R5Z6_HUMAN	106.1	4.45E+06	1.76E+06			2	2	2	1	0	0	Carbamid
2498	7624	sp|P60510|PP4C_HUMAN	106.04	1.00E+05			1.04E+05	3	1	1	2	1	1	Carbamid
2513	4738	sp|Q13416|ORC2_HUMAN	106.02	1.48E+05				5	5	1	0	0	0	
2354	5356	tr|F8VZA2|F8VZA2_HUMAN	106.01		9.73E+05		2.88E+06	5	5	0	2	0	3	
2354	5357	sp|Q6P1Q0|LTMD1_HUMAN	106.01		9.73E+05		2.88E+06	5	5	0	2	0	3	
2354	5465	tr|F8VP71|F8VP71_HUMAN	106.01		9.73E+05		2.88E+06	5	5	0	2	0	3	
2354	5466	tr|F8VVQ3|F8VVQ3_HUMAN	106.01		9.73E+05		2.88E+06	5	5	0	2	0	3	
2082	6526	sp|Q99496|RING2_HUMAN	105.87	1.88E+05	7.19E+05	5.79E+05	3.92E+05	4	3	1	2	3	2	Carbamid
2082	7450	tr|X6RFN3|X6RFN3_HUMAN	105.87	1.88E+05		5.79E+05	3.92E+05	3	2	1	1	3	2	Carbamid
2093	6897	sp|P82921|RT21_HUMAN	105.76	2.23E+06	1.86E+06	5.52E+06	5.16E+06	3	3	1	2	2	2	
2033	10010	sp|P60059|SC61G_HUMAN	105.67	5.27E+06	5.41E+06	6.77E+06	6.13E+06	2	2	2	2	2	2	Acetylatio
1896	4317	sp|Q9UNF0|PACN2_HUMAN	105.53	2.32E+06	7.10E+05	3.77E+05	3.36E+05	7	7	3	3	1	1	
1896	4361	tr|A0A0U1RR22|A0A0U1RR22_HUMAN	105.53	2.32E+06	7.10E+05	3.77E+05	3.36E+05	7	7	3	3	1	1	
2522	5492	sp|Q9UBV2|SE1L1_HUMAN	105.51	5.92E+05	3.37E+05			5	5	3	1	0	0	
2166	3777	sp|O94822|LTN1_HUMAN	105.5	2.49E+06	2.60E+05	4.86E+05		8	8	4	1	1	0	Carbamid
2005	3302	tr|F5H442|F5H442_HUMAN	105.29	4.46E+05	1.50E+06	6.47E+05	2.98E+05	8	8	3	3	1	1	
2005	3303	sp|Q99816|TS101_HUMAN	105.29	4.46E+05	1.50E+06	6.47E+05	2.98E+05	8	8	3	3	1	1	
2028	10195	sp|P47914|RL29_HUMAN	105.28	3.40E+07	4.31E+07	2.01E+08	2.86E+08	2	2	2	2	2	2	
2028	10198	tr|A0A3B3ITT5|A0A3B3ITT5_HUMAN	105.28	3.40E+07	4.31E+07	2.01E+08	2.86E+08	2	2	2	2	2	2	
1770	7532	sp|Q14956|GPNMB_HUMAN	105.19	5.36E+07	5.52E+07	5.55E+07	6.80E+07	6	6	5	4	2	5	Carbamid
1770	7533	tr|A0A3B3ISS6|A0A3B3ISS6_HUMAN	105.19	5.36E+07	5.52E+07	5.55E+07	6.80E+07	6	6	5	4	2	5	Carbamid
2023	5282	tr|E7ENV7|E7ENV7_HUMAN	105.14	5.44E+05	1.81E+05		2.23E+05	5	4	4	2	1	2	
2023	5283	sp|Q86YQ8|CPNE8_HUMAN	105.14	5.44E+05	1.81E+05		2.23E+05	5	4	4	2	1	2	
1925	3665	tr|H0YM30|H0YM30_HUMAN	104.92	1.55E+06	1.81E+06	1.86E+06	1.04E+06	9	9	2	2	2	1	Carbamid
1925	3669	sp|Q68DA7|FMN1_HUMAN	104.92	1.55E+06	1.81E+06	1.86E+06	1.04E+06	9	9	2	2	2	1	Carbamid
2345	5166	sp|P40926|MDHM_HUMAN	104.17	1.84E+06	8.32E+05	1.93E+05		5	5	5	2	1	0	
1712	5728	sp|Q13433|S39A6_HUMAN	104.03	8.38E+06	2.10E+06	2.99E+06	2.62E+06	4	4	3	1	6	3	Carbamid
1856	7903	sp|Q15388|TOM20_HUMAN	103.93	1.78E+07	3.02E+07	4.34E+07	4.90E+07	3	3	2	3	2	3	
1800	4694	sp|Q9ULK5|VANG2_HUMAN	103.9	6.22E+05	9.11E+05	1.10E+06	2.83E+05	6	6	2	3	4	1	
1730	4380	sp|P51116|FXR2_HUMAN	103.38					7	2	1	0	5	2	Carbamid
2237	6279	sp|Q9NUP1|BL1S4_HUMAN	103.32	3.31E+05	5.33E+05	1.53E+05		4	4	2	3	1	0	
2261	4860	sp|Q7L014|DDX46_HUMAN	103.25	7.69E+05	1.83E+06		3.15E+05	8	8	3	4	0	1	Carbamid
2327	4401	sp|O00203|AP3B1_HUMAN	103.25	2.20E+06	5.10E+04	8.07E+05	9.64E+05	9	9	3	1	3	3	Carbamid
2507	5804	tr|E7ETZ4|E7ETZ4_HUMAN	103.08		1.24E+05	4.14E+05	6.12E+05	6	6	0	1	1	2	Carbamid
2507	5805	sp|Q9Y6E2|BZW2_HUMAN	103.08		1.24E+05	4.14E+05	6.12E+05	6	6	0	1	1	2	Carbamid
2507	5886	tr|Q75MG1|Q75MG1_HUMAN	103.08		1.24E+05	4.14E+05	6.12E+05	6	6	0	1	1	2	Carbamid
2353	3824	sp|Q8WUA2|PPIL4_HUMAN	103.05	0	3.08E+06	8.55E+05	6.13E+05	7	7	1	4	2	2	Carbamid
2032	7454	sp|Q9Y5V0|ZN706_HUMAN	102.91	3.90E+07	3.43E+07	1.92E+07	4.67E+06	3	3	3	3	2	1	Carbamid
2047	8201	tr|F8VP67|F8VP67_HUMAN	102.9					5	1	2	4	1	1	Acetylatio
1782	3661	sp|Q92576|PHF3_HUMAN	102.76	2.92E+05	2.25E+06	1.31E+06	2.88E+06	9	9	2	5	4	4	Carbamid
1902	6425	tr|C9JYQ9|C9JYQ9_HUMAN	102.74	2.15E+07	1.16E+07	1.22E+07	1.25E+07	4	4	3	2	3	3	
1902	6426	sp|Q6P5R6|RL22L_HUMAN	102.74	2.15E+07	1.16E+07	1.22E+07	1.25E+07	4	4	3	2	3	3	
1902	6427	tr|H0Y8C2|H0Y8C2_HUMAN	102.74	2.15E+07	1.16E+07	1.22E+07	1.25E+07	4	4	3	2	3	3	
1923	4855	sp|O75369|FLNB_HUMAN	102.56				5.55E+04	6	1	2	1	2	5	Carbamid
1823	5389	sp|Q14315|FLNC_HUMAN	102.49	8.51E+06		1.33E+07	1.22E+07	7	1	3	1	3	6	Carbamid
1961	3815	sp|P78368|KC1G2_HUMAN	102.21		2.41E+06	2.78E+05	1.03E+06	8	7	1	3	1	3	Carbamid
2026	7211	tr|H7BYR8|H7BYR8_HUMAN	102.13	9.21E+06	1.70E+07	3.98E+06	7.02E+06	3	3	3	3	2	2	
2026	7383	tr|E9PMR5|E9PMR5_HUMAN	102.13	9.21E+06	1.70E+07	3.98E+06	7.02E+06	3	3	3	3	2	2	
2026	7384	tr|H0YF02|H0YF02_HUMAN	102.13	9.21E+06	1.70E+07	3.98E+06	7.02E+06	3	3	3	3	2	2	
2026	7385	tr|H0YEA2|H0YEA2_HUMAN	102.13	9.21E+06	1.70E+07	3.98E+06	7.02E+06	3	3	3	3	2	2	
2026	7386	tr|E9PJ72|E9PJ72_HUMAN	102.13	9.21E+06	1.70E+07	3.98E+06	7.02E+06	3	3	3	3	2	2	
2026	7387	tr|J3QL64|J3QL64_HUMAN	102.13	9.21E+06	1.70E+07	3.98E+06	7.02E+06	3	3	3	3	2	2	
2026	7388	tr|J3QKL5|J3QKL5_HUMAN	102.13	9.21E+06	1.70E+07	3.98E+06	7.02E+06	3	3	3	3	2	2	
2026	7389	tr|A8MZH3|A8MZH3_HUMAN	102.13	9.21E+06	1.70E+07	3.98E+06	7.02E+06	3	3	3	3	2	2	
2026	7390	tr|J3QQK6|J3QQK6_HUMAN	102.13	9.21E+06	1.70E+07	3.98E+06	7.02E+06	3	3	3	3	2	2	
2026	7391	sp|P02686|MBP_HUMAN	102.13	9.21E+06	1.70E+07	3.98E+06	7.02E+06	3	3	3	3	2	2	
2445	5803	tr|A2A2Q9|A2A2Q9_HUMAN	102.04	2.18E+05	6.57E+05		8.81E+05	4	4	1	3	0	1	
2445	6006	sp|Q9Y312|AAR2_HUMAN	102.04	2.18E+05	6.57E+05		8.81E+05	4	4	1	3	0	1	
2383	5436	sp|Q5T280|CI114_HUMAN	101.44	5.45E+05	1.05E+06	1.27E+06	8.79E+05	5	5	2	2	2	2	Carbamid
1612	7593	sp|L0R8F8|MIDUO_HUMAN	101.32	9.16E+06	5.53E+06	7.60E+06	7.36E+06	4	4	5	3	3	3	Deamidat
2416	4934	sp|Q6WCQ1|MPRIP_HUMAN	101.24		3.77E+04		2.86E+05	7	6	1	1	0	2	
2588	7896	sp|Q9Y5J9|TIM8B_HUMAN	101.06	4.67E+06	3.38E+06			3	3	3	2	0	0	Acetylatio
2588	7897	tr|G3XAN8|G3XAN8_HUMAN	101.06	4.67E+06	3.38E+06			3	3	3	2	0	0	Acetylatio
1901	4887	sp|Q96DA6|TIM14_HUMAN	100.95	2.87E+06	3.03E+06	5.98E+05	4.02E+06	6	6	2	2	2	3	
2066	4663	sp|Q14566|MCM6_HUMAN	100.86	2.08E+06	4.66E+05		1.80E+05	6	6	4	2	0	1	Carbamid
2217	6343	sp|Q14692|BMS1_HUMAN	100.76	6.44E+06	1.56E+06	1.83E+06	1.57E+06	4	4	5	1	3	1	

2209	4586	sp|Q14669|TRIPC_HUMAN	100.62	1.58E+06	9.52E+05	1.31E+06	1.95E+06	5	5	1	2	2	4	Carbamid
2347	5384	tr|E7EW18|E7EW18_HUMAN	100.6			1.18E+05		5	5	0	0	1	0	Carbamid
2347	5386	sp|P06746|DPOLB_HUMAN	100.6			1.18E+05		5	5	0	0	1	0	Carbamid
1913	4562	sp|P52747|ZN143_HUMAN	100.54	1.82E+06	7.15E+05	7.06E+05	3.54E+05	6	6	6	1	1	1	Carbamid
2357	8329	tr|L0R6P0|L0R6P0_HUMAN	100.46	2.03E+06	1.62E+06	5.42E+06		2	2	2	1	2	0	Deamidat
2357	8330	sp|P86397|HTD2_HUMAN	100.46	2.03E+06	1.62E+06	5.42E+06		2	2	2	1	2	0	Deamidat
3047	8186	sp|P20930|FILA_HUMAN	100.43					3	3	0	0	0	0	Deamidat
2134	6520	sp|Q9Y4Z0|LSM4_HUMAN	100.41	7.57E+05	9.68E+05	1.19E+06	4.12E+06	5	5	1	1	1	2	Carbamid
2134	6537	tr|V9GZ56|V9GZ56_HUMAN	100.41	7.57E+05	9.68E+05	1.19E+06	4.12E+06	5	5	1	1	1	2	Carbamid
2473	8139	sp|P61960|UFM1_HUMAN	100.33	9.72E+06	6.22E+06	4.10E+05	3.26E+06	3	3	2	2	1	1	
2211	6161	sp|P36404|ARL2_HUMAN	100.24	2.19E+06	1.00E+06	0	6.99E+06	4	4	3	1	1	3	
2366	8820	tr|E9PLN8|E9PLN8_HUMAN	100.24	7.59E+06	2.07E+06	4.60E+06		2	2	2	2	1	0	Carbamid
2366	10224	tr|I3L3B4|I3L3B4_HUMAN	100.24	7.59E+06	2.07E+06	4.60E+06		2	2	2	2	1	0	Carbamid
2366	10225	tr|F8W9H0|F8W9H0_HUMAN	100.24	7.59E+06	2.07E+06	4.60E+06		2	2	2	2	1	0	Carbamid
2366	10226	sp|Q9BQB6|VKOR1_HUMAN	100.24	7.59E+06	2.07E+06	4.60E+06		2	2	2	2	1	0	Carbamid
1704	5171	tr|G5EA18|G5EA18_HUMAN	100.17			6.82E+05	6.68E+05	6	3	4	2	5	4	Deamidat
1704	5189	tr|E5RJN7|E5RJN7_HUMAN	100.17			6.82E+05	6.68E+05	6	3	4	2	5	4	Deamidat
1704	5231	tr|A0A0A0MTC4|A0A0A0MTC4_HUMAN	100.17			6.82E+05	6.68E+05	6	3	4	2	5	4	Deamidat
1704	5241	tr|A0A0A0MTD1|A0A0A0MTD1_HUMAN	100.17			6.82E+05	6.68E+05	6	3	4	2	5	4	Deamidat
1704	5244	tr|F8VPI7|F8VPI7_HUMAN	100.17			6.82E+05	6.68E+05	6	3	4	2	5	4	Deamidat
1704	5258	tr|E7EPX0|E7EPX0_HUMAN	100.17			6.82E+05	6.68E+05	6	3	4	2	5	4	Deamidat
1704	5262	tr|A0A0A0MTC6|A0A0A0MTC6_HUMAN	100.17			6.82E+05	6.68E+05	6	3	4	2	5	4	Deamidat
1704	5265	tr|E7EVJ4|E7EVJ4_HUMAN	100.17			6.82E+05	6.68E+05	6	3	4	2	5	4	Deamidat
1704	5277	tr|E9PH62|E9PH62_HUMAN	100.17			6.82E+05	6.68E+05	6	3	4	2	5	4	Deamidat
1704	5285	sp|Q9NUL3|STAU2_HUMAN	100.17			6.82E+05	6.68E+05	6	3	4	2	5	4	Deamidat
2070	3746	sp|Q53F19|NCBP3_HUMAN	100.12	2.33E+06	3.20E+06	4.97E+05	2.20E+06	8	8	3	4	1	2	
2775	7774	sp|O75955|FLOT1_HUMAN	100.07	1.46E+05		2.05E+05	3.35E+04	3	3	1	0	1	1	
2004	6701	tr|S4R3N1|S4R3N1_HUMAN	100.07			3.49E+06	1.49E+05	6	2	3	2	1	2	Carbamid
2400	10266	tr|K7ELA4|K7ELA4_HUMAN	99.95					2	1	1	1	1	1	
2400	10267	tr|B5MD17|B5MD17_HUMAN	99.95					2	1	1	1	1	1	
2400	10268	tr|J3KS05|J3KS05_HUMAN	99.95					2	1	1	1	1	1	
2400	10269	sp|P83916|CBX1_HUMAN	99.95					2	1	1	1	1	1	
2281	7629	sp|P10301|RRAS_HUMAN	99.92	1.82E+06	0		8.76E+05	4	4	4	1	0	2	Carbamid
2361	6832	sp|Q6KB66|K2C80_HUMAN	99.9					3	2	1	1	1	0	
2514	5684	sp|Q9H6R4|NOL6_HUMAN	99.88			1.40E+06	2.36E+06	7	7	0	0	3	4	
1627	4493	sp|Q9NWU5|RM22_HUMAN	99.64	1.79E+06	6.10E+06	1.13E+07	5.34E+06	6	5	1	4	4	4	
1627	4495	tr|E7ESL0|E7ESL0_HUMAN	99.64	1.79E+06	6.10E+06	1.13E+07	5.34E+06	6	5	1	4	4	4	
1627	4499	tr|J3KQY1|J3KQY1_HUMAN	99.64	1.79E+06	6.10E+06	1.13E+07	5.34E+06	6	5	1	4	4	4	
2692	6725	sp|Q8TAD8|SNIP1_HUMAN	99.54	7.12E+05			1.58E+06	4	4	1	0	0	3	
2110	4104	sp|Q9NW08|RPC2_HUMAN	99.54			1.61E+05	2.27E+06	7	7	0	0	3	2	Carbamid
2159	9975	sp|Q99735|MGST2_HUMAN	99.33	4.46E+06	5.50E+06	1.42E+07	1.53E+07	2	2	2	1	2	1	
2114	5468	tr|J3QRU8|J3QRU8_HUMAN	99.11	4.97E+05	8.71E+05	1.05E+06		5	2	3	3	1	0	Carbamid
2114	5474	tr|A0A0C4DGN6|A0A0C4DGN6_HUMAN	99.11	4.97E+05	8.71E+05	1.05E+06		5	2	3	3	1	0	Carbamid
2114	5478	sp|Q9Y2X7|GIT1_HUMAN	99.11	4.97E+05	8.71E+05	1.05E+06		5	2	3	3	1	0	Carbamid
2238	9993	sp|O75394|RM33_HUMAN	98.97	4.48E+06	4.80E+06	3.09E+06	5.57E+06	2	2	2	2	1	2	Carbamid
1922	3866	sp|P11137|MTAP2_HUMAN	98.96	1.82E+07	1.30E+07	1.47E+06	8.73E+06	8	8	4	4	1	3	
2118	5310	sp|Q14147|DHX34_HUMAN	98.92	3.79E+05	9.36E+05		5.87E+05	5	5	1	3	0	1	Carbamid
1743	4466	tr|B1ALA9|B1ALA9_HUMAN	98.9	3.23E+06	1.94E+06	5.52E+06	4.99E+06	5	5	3	3	2	3	Carbamid
1743	4467	sp|P60891|PRPS1_HUMAN	98.9	3.23E+06	1.94E+06	5.52E+06	4.99E+06	5	5	3	3	2	3	Carbamid
2246	4538	sp|P23258|TBG1_HUMAN	98.87	8.18E+05	7.51E+05	4.52E+05	1.48E+06	7	7	2	1	1	2	Carbamid
2686	10304	sp|Q86WP2|GPBP1_HUMAN	98.73	5.79E+05	1.56E+06		8.11E+05	2	2	2	2	0	1	
2686	10306	tr|D4PHA4|D4PHA4_HUMAN	98.73	5.79E+05	1.56E+06		8.11E+05	2	2	2	2	0	1	
1940	7015	sp|Q6IQ22|RAB12_HUMAN	98.7	0			6.76E+05	5	4	3	2	2	2	Acetylatio
1940	7265	tr|A0A3B3ITT1|A0A3B3ITT1_HUMAN	98.7	0				4	3	3	2	2	1	
2156	6416	sp|P54274|TERF1_HUMAN	98.67	4.32E+06	3.38E+06	1.72E+06	3.31E+06	6	6	3	2	2	2	Carbamid
2121	5302	tr|R9WNI0|R9WNI0_HUMAN	98.57		6.63E+04			5	2	1	1	3	1	Carbamid
2121	5304	tr|A8MQB8|A8MQB8_HUMAN	98.57		6.63E+04			5	2	1	1	3	1	Carbamid
2121	5305	tr|G3V0J0|G3V0J0_HUMAN	98.57		6.63E+04			5	2	1	1	3	1	Carbamid
2121	5309	sp|Q06787|FMR1_HUMAN	98.57		6.63E+04			5	2	1	1	3	1	Carbamid
2847	6544	sp|O43824|GTPB6_HUMAN	98.56			1.69E+05		4	4	0	0	1	0	
2854	8159	sp|Q8NCW5|NNRE_HUMAN	98.35		1.74E+05		3.56E+05	3	3	0	1	0	1	
2154	5196	sp|Q5JVF3|PCID2_HUMAN	98.21	3.49E+05	3.92E+05		5.41E+05	5	5	5	3	0	2	Carbamid
2025	6576	tr|Q66S35|Q66S35_HUMAN	98.15					3	1	2	2	2	2	Carbamid
2025	6577	sp|Q16877|F264_HUMAN	98.15					3	1	2	2	2	2	Carbamid
2062	3345	sp|Q9BTV4|TMM43_HUMAN	98.1	2.35E+06		3.11E+06	1.10E+06	10	10	4	0	2	3	Deamidat
2141	5939	sp|P51810|GP143_HUMAN	98	1.23E+06	4.05E+05	4.27E+05	1.37E+06	4	4	2	1	1	2	Carbamid
2410	6204	sp|Q9BUI4|RPC3_HUMAN	97.86	2.33E+06	4.03E+06	4.31E+04	8.12E+04	5	5	1	1	1	1	Deamidat
2851	8093	sp|O14523|C2C2L_HUMAN	97.72				1.19E+06	3	3	0	0	0	1	Carbamid
2901	6862	sp|P05109|S10A8_HUMAN	97.62					3	3	0	0	0	0	Carbamid
1631	3766	sp|Q8IVH8|M4K3_HUMAN	97.49	1.27E+06	1.85E+06	1.61E+06	1.41E+06	11	8	3	6	3	4	Carbamid
2293	6831	sp|Q9NV56|MRGBP_HUMAN	97.44	1.46E+06	7.45E+05	8.15E+05	7.03E+05	4	4	2	1	1	1	Carbamid
2282	5390	sp|Q96RT1|ERBIN_HUMAN	97.22	2.03E+06	4.26E+05	5.72E+05	4.36E+04	5	5	4	1	3	1	
2149	5940	sp|Q70Z53|F10C1_HUMAN	97.16	2.51E+06	1.18E+06		2.03E+06	5	5	3	1	0	3	Carbamid
2541	5762	tr|A0A0A0MRP0|A0A0A0MRP0_HUMAN	97.08	2.97E+06	1.61E+06			5	5	5	1	0	0	
2541	5772	sp|Q8IWC1|MA7D3_HUMAN	97.08	2.97E+06	1.61E+06			5	5	5	1	0	0	
2450	8167	sp|Q15269|PWP2_HUMAN	96.96		5.93E+04	1.03E+06	5.01E+05	4	4	0	1	2	2	
2450	8168	tr|A0A0B4J2E5|A0A0B4J2E5_HUMAN	96.96		5.93E+04	1.03E+06	5.01E+05	4	4	0	1	2	2	
2488	7696	tr|J3KPP0|J3KPP0_HUMAN	96.92	1.90E+06		1.19E+06	1.53E+06	3	3	2	0	1	1	Carbamid
2488	7697	sp|Q9Y6G3|RM42_HUMAN	96.92	1.90E+06		1.19E+06	1.53E+06	3	3	2	0	1	1	Carbamid
2488	7781	tr|S4R360|S4R360_HUMAN	96.92	1.90E+06		1.19E+06	1.53E+06	3	3	2	0	1	1	Carbamid
2488	7782	tr|S4R2Z7|S4R2Z7_HUMAN	96.92	1.90E+06		1.19E+06	1.53E+06	3	3	2	0	1	1	Carbamid
2320	5663	sp|P53701|CCHL_HUMAN	96.87	2.48E+06	2.78E+06	3.80E+05		5	5	2	3	1	0	Carbamid
2202	6383	sp|Q86TU7|SETD3_HUMAN	96.8	1.83E+06	0		1.14E+06	4	4	3	1	0	2	
2679	8439	sp|Q9NRP4|SDHF3_HUMAN	96.67	2.03E+06				2	2	2	0	0	0	Carbamid
2712	8063	sp|P41208|CETN2_HUMAN	96.62			3.03E+05	3.50E+05	3	3	0	0	1	1	
2278	5453	tr|E7EW77|E7EW77_HUMAN	96.58	2.46E+05				5	1	3	0	1	1	
2278	5455	tr|E7EP65|E7EP65_HUMAN	96.58	2.46E+05				5	1	3	0	1	1	
2278	5456	tr|A0A0C4DG21|A0A0C4DG21_HUMAN	96.58	2.46E+05				5	1	3	0	1	1	
2278	5457	sp|Q9NYB9|ABI2_HUMAN	96.58	2.46E+05				5	1	3	0	1	1	
2278	5458	tr|F8WAL6|F8WAL6_HUMAN	96.58	2.46E+05				5	1	3	0	1	1	
2278	5687	tr|E9PEZ7|E9PEZ7_HUMAN	96.58	2.46E+05				5	1	3	0	1	1	
2193	4675	sp|Q69YN2|C19L1_HUMAN	96.52	7.16E+05	3.85E+05		8.88E+04	6	6	2	2	0	1	Carbamid
2412	4665	sp|P29401|TKT_HUMAN	96.49	7.43E+05	7.54E+05			7	7	2	1	0	0	
2412	4932	tr|A0A0B4J1R6|A0A0B4J1R6_HUMAN	96.49	7.43E+05	7.54E+05			6	6	2	1	0	0	
2346	5284	sp|Q9NY93|DDX56_HUMAN	96.45			1.40E+06	2.43E+06	6	6	0	0	1	3	
2479	5651	sp|Q11206|SIA4C_HUMAN	96.36	8.84E+05		3.09E+05	3.23E+05	5	5	3	0	2	1	
2049	4137	tr|A0A384DVU0|A0A384DVU0_HUMAN	96.33	1.45E+05	7.66E+05	1.43E+06	1.41E+06	9	9	1	3	2	3	Carbamid
2049	4138	sp|Q8IY17|PLPL6_HUMAN	96.33	1.45E+05	7.66E+05	1.43E+06	1.41E+06	9	9	1	3	2	3	Carbamid
2437	5913	sp|Q8WW22|DNJA4_HUMAN	96.24	1.03E+05	6.27E+05	8.14E+05		4	4	1	2	2	0	
2317	5223	sp|P28370|SMCA1_HUMAN	96.2					5	1	1	1	0	0	Carbamid
2317	5224	tr|A0A0A0MRP6|A0A0A0MRP6_HUMAN	96.2					5	1	1	1	0	0	Carbamid
2317	5225	tr|B7ZLQ5|B7ZLQ5_HUMAN	96.2					5	1	1	1	0	0	Carbamid
2446	6786	sp|Q9UL03|INT6_HUMAN	96.18	2.32E+05	8.68E+04		2.30E+05	3	3	2	1	0	1	
3160	8379	sp|P31151|S10A7_HUMAN	96.11					3	3	0	0	0	0	Carbamid
2210	5562	tr|A0A087WWP4|A0A087WWP4_HUMAN	95.96	3.39E+06	2.86E+06	1.11E+06	9.46E+05	7	7	5	3	3	1	
2210	5579	sp|Q96T37|RBM15_HUMAN	95.96	3.39E+06	2.86E+06	1.11E+06	9.46E+05	7	7	5	3	3	1	
2221	7847	sp|O95104|SFR15_HUMAN	95.93	5.72E+05	1.03E+06	4.16E+05	1.30E+06	3	3	2	2	1	2	
2151	6718	sp|Q92785|REQU_HUMAN	95.89	8.99E+05	1.45E+06	6.31E+05		3	3	2	2	1	0	Carbamid
2151	6723	tr|J3KMZ8|J3KMZ8_HUMAN	95.89	8.99E+05	1.45E+06	6.31E+05		3	3	2	2	1	0	Carbamid
2130	5404	sp|Q7L2Z9|CENPQ_HUMAN	95.78	4.16E+05	4.58E+05	1.52E+06	3.19E+06	6	6	3	3	2	1	Carbamid
2549	5553	sp|Q7L1W4|LRC8D_HUMAN	95.74				1.02E+06	5	5	0	0	0	2	Carbamid
2530	6807	tr|K7EK00|K7EK00_HUMAN	95.73	4.49E+05	8.70E+05	1.06E+06	2.04E+05	5	5	1	1	2	1	
2530	6808	sp|Q96ND0|F210A_HUMAN	95.73	4.49E+05	8.70E+05	1.06E+06	2.04E+05	5	5	1	1	2	1	
2300	10013	sp|P14406|CX7A2_HUMAN	95.71	8.99E+05	1.92E+06	6.69E+06	3.65E+06	2	2	1	1	2	1	
2300	10016	tr|D6RIE3|D6RIE3_HUMAN	95.71	8.99E+05	1.92E+06	6.69E+06	3.65E+06	2	2	1	1	2	1	
2300	10019	tr|D6RGV5|D6RGV5_HUMAN	95.71	8.99E+05	1.92E+06	6.69E+06	3.65E+06	2	2	1	1	2	1	

2300	10022	tr|H0UI06|H0UI06_HUMAN	95.71	8.99E+05	1.92E+06	6.69E+06	3.65E+06	2	2	1	1	2	1	
2071	3820	sp|Q14764|MVP_HUMAN	95.67		1.85E+06	1.32E+05	1.21E+06	9	9	0	3	1	2	Carbamid
1984	4416	sp|Q13242|SRSF9_HUMAN	95.61	4.79E+05	1.36E+06	5.61E+05	5.55E+05	8	7	1	3	2	3	
1826	4130	tr|E9PD53|E9PD53_HUMAN	95.57		3.12E+05	2.76E+06	7.71E+06	7	7	0	1	5	4	Carbamid
1826	4132	sp|Q9NTJ3|SMC4_HUMAN	95.57		3.12E+05	2.76E+06	7.71E+06	7	7	0	1	5	4	Carbamid
2308	4808	tr|A0A1W2PQ90|A0A1W2PQ90_HUMAN	95.52		1.64E+05		2.44E+05	6	6	0	1	0	1	Carbamid
2308	4809	sp|Q92878|RAD50_HUMAN	95.52		1.64E+05		2.44E+05	6	6	0	1	0	1	Carbamid
1819	4927	sp|O14980|XPO1_HUMAN	95.37	5.52E+04	2.99E+05		3.16E+05	8	7	2	5	1	3	Carbamid
1644	4515	sp|Q99442|SEC62_HUMAN	95.26	5.33E+06	1.24E+07	1.10E+07	8.56E+06	7	7	5	5	5	3	Carbamid
1916	10280	tr|J3KT68|J3KT68_HUMAN	95.24	7.80E+06	6.60E+06	9.83E+06	8.19E+06	3	3	2	2	3	2	
1916	10282	sp|Q5BJF2|SGMR2_HUMAN	95.24	7.80E+06	6.60E+06	9.83E+06	8.19E+06	3	3	2	2	3	2	
2586	7662	sp|Q86TN4|TRPT1_HUMAN	95.01	7.49E+05	1.82E+06		6.45E+05	3	3	2	3	0	1	
2406	8865	sp|Q13445|TMED1_HUMAN	94.96	9.56E+05		1.11E+06	3.34E+05	2	2	2	0	1	1	Carbamid
1934	5795	sp|Q9HAV0|GBB4_HUMAN	94.91	1.06E+05				4	1	2	2	1	3	Carbamid
2818	7116	tr|F5H5P2|F5H5P2_HUMAN	94.9				4.78E+05	3	3	0	0	0	1	
2818	7135	sp|P12694|ODBA_HUMAN	94.9				4.78E+05	3	3	0	0	0	1	
2223	8610	sp|Q9Y241|HIG1A_HUMAN	94.73	6.70E+06	6.96E+06	6.19E+06	5.88E+06	2	2	2	2	1	1	Acetylatic
2223	9167	tr|C9JAW5|C9JAW5_HUMAN	94.73	6.70E+06	6.96E+06	6.19E+06	5.88E+06	2	2	2	2	1	1	Acetylatic
2223	9168	tr|C9JNU6|C9JNU6_HUMAN	94.73	6.70E+06	6.96E+06	6.19E+06	5.88E+06	2	2	2	2	1	1	Acetylatic
2523	5875	sp|Q8N8R7|AL14E_HUMAN	94.72	2.87E+06	5.00E+05	6.00E+05	1.55E+06	4	4	3	1	1	3	Carbamid
2523	6367	tr|E9PRK7|E9PRK7_HUMAN	94.72	2.87E+06	5.00E+05	6.00E+05	1.55E+06	4	4	3	1	1	3	Carbamid
2384	6040	sp|Q9Y619|ORNT1_HUMAN	94.69		3.62E+05		5.39E+05	4	4	0	1	0	2	
2288	6874	sp|Q86Y79|PTH_HUMAN	94.64			1.98E+06	8.43E+05	3	3	0	0	4	2	Carbamid
2288	6977	tr|A0A096LP22|A0A096LP22_HUMAN	94.64			1.98E+06	8.43E+05	3	3	0	0	4	2	Carbamid
2288	7039	tr|C9J7Z1|C9J7Z1_HUMAN	94.64			1.98E+06	8.43E+05	3	3	0	0	4	2	Carbamid
2391	9323	sp|Q8TDX7|NEK7_HUMAN	94.62		2.59E+05	2.27E+05		2	1	0	2	1	0	Carbamid
2486	6505	sp|Q8NEW0|ZNT7_HUMAN	94.61			6.58E+05	7.19E+05	4	4	0	0	1	1	Carbamid
2439	6697	sp|Q9Y678|COPG1_HUMAN	94.58		2.06E+05		1.77E+05	3	2	1	1	0	2	
2108	6504	tr|Q5QPL9|Q5QPL9_HUMAN	94.53	9.18E+05	4.05E+06	2.78E+06	3.62E+06	5	5	1	5	1	2	
2108	6506	sp|Q9UKM9|RALY_HUMAN	94.53	9.18E+05	4.05E+06	2.78E+06	3.62E+06	5	5	1	5	1	2	
2585	7625	sp|Q05048|CSTF1_HUMAN	94.49	2.40E+05	4.37E+05	6.03E+05		4	4	1	2	1	0	Carbamid
2900	6605	tr|A0A087WTZ5|A0A087WTZ5_HUMAN	94.42	1.41E+05	6.21E+05			3	3	1	2	0	0	
2900	6608	tr|E9PJ81|E9PJ81_HUMAN	94.42	1.41E+05	6.21E+05			3	3	1	2	0	0	
2900	6609	tr|E9PRQ7|E9PRQ7_HUMAN	94.42	1.41E+05	6.21E+05			3	3	1	2	0	0	
2900	6610	sp|Q04323|UBXN1_HUMAN	94.42	1.41E+05	6.21E+05			3	3	1	2	0	0	
2421	7858	sp|Q9NXR1|NDE1_HUMAN	94.41	9.27E+05	5.08E+05	5.67E+05		3	3	3	2	1	0	
2264	5338	tr|W5RWE6|W5RWE6_HUMAN	94.38	1.66E+06		2.76E+05	7.20E+05	6	6	5	0	1	1	Carbamid
2264	5343	sp|Q7Z7A4|PXK_HUMAN	94.38	1.66E+06		2.76E+05	7.20E+05	6	6	5	0	1	1	Carbamid
2540	5503	sp|Q9H9A5|CNO10_HUMAN	94.36		1.89E+05	9.30E+05	5.98E+05	6	6	0	1	1	1	
2423	5011	sp|Q15477|SKIV2_HUMAN	94.32	3.83E+05	1.15E+05			5	5	3	1	0	0	Carbamid
2286	5328	sp|Q9BQ75|CMS1_HUMAN	94.31			1.26E+07	8.02E+06	5	5	0	0	4	3	Carbamid
2286	5383	tr|C9J384|C9J384_HUMAN	94.31			1.26E+07	8.02E+06	5	5	0	0	4	3	Carbamid
2107	4375	sp|Q9Y620|RA54B_HUMAN	94.21	2.16E+06	8.03E+05	3.59E+06	3.74E+05	7	7	3	2	4	1	Carbamid
2107	4376	tr|A0A087X0H2|A0A087X0H2_HUMAN	94.21	2.16E+06	8.03E+05	3.59E+06	3.74E+05	7	7	3	2	4	1	Carbamid
2745	7630	tr|F6T1Q0|F6T1Q0_HUMAN	94.08				2.54E+05	4	4	0	0	0	1	Carbamid
2745	7634	sp|Q6L8Q7|PDE12_HUMAN	94.08				2.54E+05	4	4	0	0	0	1	Carbamid
1997	5475	sp|Q92890|UFD1_HUMAN	93.94	4.32E+06	2.56E+06	9.58E+05		4	4	5	4	1	0	Deamidat
3656	12436	tr|B8ZZF8|B8ZZF8_HUMAN	93.76					1	1	0	0	0	0	Acetylatic
3656	12443	sp|Q9Y4Y9|LSM5_HUMAN	93.76					1	1	0	0	0	0	Acetylatic
2131	4229	sp|Q8N556|AFAP1_HUMAN	93.71	2.90E+06	1.07E+06	1.48E+06	1.73E+06	7	7	4	1	2	3	
2215	5998	sp|Q96EV2|RBM33_HUMAN	93.65		3.00E+05	5.31E+05	1.64E+06	4	4	0	1	1	4	
2215	6248	tr|H0Y3K4|H0Y3K4_HUMAN	93.65		3.00E+05	5.31E+05	1.64E+06	4	4	0	1	1	4	
2031	5292	sp|Q92600|CNOT9_HUMAN	93.6	6.05E+05	4.12E+05	4.86E+05	4.87E+05	5	5	1	2	1	1	
2511	8244	sp|Q9UHB7|AFF4_HUMAN	93.52	1.11E+06	4.29E+06		1.46E+06	3	3	1	3	0	3	
2511	8879	tr|C9JCE0|C9JCE0_HUMAN	93.52	1.11E+06	4.29E+06		1.23E+06	2	2	1	3	0	1	
2528	5595	sp|Q16763|UBE2S_HUMAN	93.51	7.81E+06		4.12E+05	9.95E+05	5	5	5	0	1	2	Carbamid
2528	5957	tr|K7EPJ1|K7EPJ1_HUMAN	93.51	6.65E+06		4.12E+05	6.59E+05	4	4	4	0	1	1	Carbamid
2259	5447	sp|Q96J02|ITCH_HUMAN	93.51		2.63E+05		6.13E+03	6	4	1	4	1	1	Carbamid
2272	5999	sp|Q9BYD2|RM09_HUMAN	93.5	3.26E+05	1.19E+06	3.24E+06	2.45E+06	5	5	1	2	4	2	
2485	12447	sp|Q8N6L1|KTAP2_HUMAN	93.5	1.19E+06	1.03E+06	2.20E+06	2.68E+06	1	1	1	1	1	1	
2385	6431	sp|P55735|SEC13_HUMAN	93.49	3.85E+05	2.94E+05	9.87E+05	3.41E+05	4	4	1	1	1	1	Carbamid
2340	6875	sp|Q9UGR2|Z3H7B_HUMAN	93.49	3.86E+05	1.73E+05		1.76E+06	5	5	1	2	0	3	Carbamid
2332	4660	sp|P40126|TYRP2_HUMAN	93.25	1.19E+07	1.79E+06	6.48E+05	1.07E+06	6	6	1	1	2	2	Carbamid
2574	8319	sp|Q14766|LTBP1_HUMAN	93.19	1.42E+06	1.76E+06			3	3	3	2	0	0	
2689	5549	sp|Q14435|GALT3_HUMAN	93.18		2.22E+05			4	4	0	1	0	0	Carbamid
2428	4704	sp|Q9H6S0|YTDC2_HUMAN	92.94			2.57E+06	3.68E+05	7	7	0	0	4	1	Carbamid
2177	4536	sp|Q9H0G5|NSRP1_HUMAN	92.91	2.07E+06	1.59E+07		1.19E+06	6	6	3	6	0	1	Carbamid
2177	4608	tr|A0A024QZ33|A0A024QZ33_HUMAN	92.91	2.07E+06	1.59E+07		1.19E+06	6	6	3	6	0	1	Carbamid
2051	4857	sp|Q8TF74|WIPF2_HUMAN	92.89	4.35E+06	1.37E+06		3.49E+05	6	6	4	2	0	1	
2328	5141	sp|Q8N0T1|CH059_HUMAN	92.84				1.45E+06	5	5	0	0	0	2	
2508	8239	sp|Q8WY36|BBX_HUMAN	92.82	2.66E+06	1.17E+06	1.89E+06	6.25E+05	4	4	3	2	1	1	
2172	4343	sp|Q96ST3|SIN3A_HUMAN	92.77	2.33E+05		5.97E+06	5.09E+05	10	10	1	0	5	2	Carbamid
2339	6502	sp|P25490|TYY1_HUMAN	92.68	3.54E+06	2.16E+06	9.40E+05		4	4	2	1	1	0	Carbamid
2339	6635	tr|H0YJV7|H0YJV7_HUMAN	92.68	3.54E+06	2.16E+06	9.40E+05		4	4	2	1	1	0	Carbamid
2314	6480	sp|Q5T8P6|RBM26_HUMAN	92.65	1.60E+05	5.35E+05		1.08E+06	5	5	2	2	0	2	Carbamid
2314	6481	tr|A0A087X0H9|A0A087X0H9_HUMAN	92.65	1.60E+05	5.35E+05		1.08E+06	5	5	2	2	0	2	Carbamid
2280	7157	sp|Q8N0V3|RBFA_HUMAN	92.49		3.67E+06	2.27E+06		3	3	0	3	2	0	Carbamid
2885	8337	tr|H3BST1|H3BST1_HUMAN	92.39	6.60E+05	2.42E+05			2	2	2	1	0	0	Carbamid
2885	8433	sp|Q9UL45|BL1S6_HUMAN	92.39	6.60E+05	2.42E+05			2	2	2	1	0	0	Carbamid
2714	8828	sp|Q16533|SNPC1_HUMAN	92.23	1.97E+06	1.51E+06	1.30E+06	5.62E+04	2	2	1	1	1	1	
2270	4944	sp|Q9H6W3|RIOX1_HUMAN	92.07			1.11E+06	4.36E+06	6	6	0	0	1	6	Acetylatic
2448	7535	sp|Q86Y07|VRK2_HUMAN	91.98	4.38E+06	1.59E+05		1.46E+06	4	4	3	1	0	2	Deamidat
2296	7726	sp|P28066|PSA5_HUMAN	91.96	1.17E+06	1.01E+06	6.96E+05	7.36E+04	3	3	2	2	1	1	Carbamid
2258	5288	sp|O75475|PSIP1_HUMAN	91.91	4.38E+05	8.72E+05		9.26E+04	6	5	1	3	0	2	
2194	5195	sp|Q53S58|TM177_HUMAN	91.88	6.66E+05	1.10E+06		3.60E+05	5	5	2	2	0	1	
1952	5501	sp|Q9Y291|RT33_HUMAN	91.83	2.96E+06	6.87E+06	7.41E+06	1.34E+07	5	5	2	2	1	3	Acetylatic
2344	5093	sp|Q6NYC8|PPR18_HUMAN	91.78		7.23E+05			7	6	1	3	0	0	Carbamid
2344	5094	tr|A0A0G2JHC2|A0A0G2JHC2_HUMAN	91.78		7.23E+05			7	6	1	3	0	0	Carbamid
2298	9682	sp|Q86SX6|GLRX5_HUMAN	91.72		1.99E+05		3.58E+05	3	2	1	2	2	2	
1909	5672	tr|H7BZK6|H7BZK6_HUMAN	91.66	1.78E+06	2.13E+06	2.13E+06	5.49E+06	4	4	3	4	2	2	
1909	5674	sp|P62068|UBP46_HUMAN	91.66	1.78E+06	2.13E+06	2.13E+06	5.49E+06	4	4	3	4	2	2	
2084	5428	sp|Q9HAV7|GRPE1_HUMAN	91.64	2.67E+06	1.79E+06		4.68E+05	5	5	4	2	0	1	
2285	6720	sp|O75351|VPS4B_HUMAN	91.58	2.83E+05				4	3	2	0	1	1	Carbamid
2190	7442	tr|H0YE28|H0YE28_HUMAN	91.57	1.11E+06	5.79E+06	1.10E+06	4.69E+06	4	4	1	4	1	2	
2190	7443	tr|A0A087WYP2|A0A087WYP2_HUMAN	91.57	1.11E+06	5.79E+06	1.10E+06	4.69E+06	4	4	1	4	1	2	
2190	7444	sp|Q8IZQ5|SELH_HUMAN	91.57	1.11E+06	5.79E+06	1.10E+06	4.69E+06	4	4	1	4	1	2	
3064	8022	sp|Q96EU6|RRP36_HUMAN	91.54		1.03E+06	2.37E+06	6.19E+05	4	4	0	1	2	1	
1920	6565	tr|M0QZQ3|M0QZQ3_HUMAN	91.1	6.49E+07	5.66E+07	5.12E+07	5.61E+07	4	2	3	3	3	4	Carbamid
1920	6581	tr|C9JY79|C9JY79_HUMAN	91.1	6.49E+07	5.66E+07	5.12E+07	5.61E+07	4	2	3	3	3	4	Carbamid
1920	6582	sp|Q9H254|SPTN4_HUMAN	91.1	6.49E+07	5.66E+07	5.12E+07	5.61E+07	4	2	3	3	3	4	Carbamid
2365	7854	sp|Q92506|DHB8_HUMAN	91.06	1.53E+06	4.26E+06	1.62E+06		3	3	1	3	2	0	
2324	5407	sp|P56182|RRP1_HUMAN	90.94	9.02E+05	6.26E+05	3.73E+06	1.20E+06	7	7	2	1	3	1	Carbamid
2653	5267	sp|Q92598|HS105_HUMAN	90.92		4.41E+04	9.68E+05		5	5	0	1	3	0	Carbamid
2120	4105	tr|A0A0A0MRA5|A0A0A0MRA5_HUMAN	90.9		2.61E+06		2.85E+05	7	7	0	3	0	1	Carbamid
2120	4106	tr|B7Z4B8|B7Z4B8_HUMAN	90.9		2.61E+06		2.85E+05	7	7	0	3	0	1	Carbamid
2120	4113	sp|Q9BUJ2|HNRL1_HUMAN	90.9		2.61E+06		2.85E+05	7	7	0	3	0	1	Carbamid
2322	4947	sp|O75691|UTP20_HUMAN	90.89	1.39E+05		5.15E+05	1.68E+06	5	5	1	0	1	4	
1491	6308	sp|Q969Q0|RL36L_HUMAN	90.67	6.91E+06	5.84E+06	7.46E+06	1.39E+07	4	1	4	4	4	4	Carbamid
1594	6863	sp|P49207|RL34_HUMAN	90.65	3.11E+07	4.99E+07	2.93E+08	3.21E+08	4	4	2	2	3	6	Carbamid
2283	5655	tr|E7EM64|E7EM64_HUMAN	90.62	1.54E+06	7.70E+05			6	6	4	2	0	0	Carbamid
2283	5656	sp|Q7L5N1|CSN6_HUMAN	90.62	1.54E+06	7.70E+05			6	6	4	2	0	0	Carbamid
2509	5299	sp|Q6PCB5|RSBNL_HUMAN	90.61			4.41E+06	1.40E+06	5	5	0	0	4	2	Carbamid
2372	5813	sp|Q9NXW2|DJB12_HUMAN	90.53			4.85E+05	0	5	5	0	0	1	1	Acetylatic
2372	6385	tr|J3KPS0|J3KPS0_HUMAN	90.53			4.85E+05	0	4	4	0	0	1	1	
2220	7833	sp|P63272|SPT4H_HUMAN	90.48	4.14E+06	0	1.53E+06	2.48E+06	4	4	3	1	1	4	Carbamid
2174	4569	sp|P55199|ELL_HUMAN	90.47	4.96E+06	1.94E+06		5.23E+05	7	7	6	2	0	1	Carbamid

2248	5264	sp|Q13425|SNTB2_HUMAN	90.47	8.61E+05	1.62E+06			7	7	2	3	0	0	Carbamid
2490	8065	sp|P07737|PROF1_HUMAN	90.17	1.01E+06	7.72E+05			3	3	2	2	0	0	
1962	5069	sp|Q99417|MYCBP_HUMAN	90.16		1.66E+06		1.68E+06	6	6	0	2	0	3	
1962	5072	tr|A0A087WV05|A0A087WV05_HUMAN	90.16		1.66E+06		1.68E+06	6	6	0	2	0	3	
2150	6501	tr|K7ERG2|K7ERG2_HUMAN	90	9.71E+05	1.40E+06	7.65E+05	5.02E+05	3	3	2	2	1	1	
2150	6503	sp|Q9NYR9|KBRS2_HUMAN	90	9.71E+05	1.40E+06	7.65E+05	5.02E+05	3	3	2	2	1	1	
1991	5614	tr|A0A1W2PPM2|A0A1W2PPM2_HUMAN	89.9	6.99E+05	1.36E+06	6.08E+06	1.75E+06	6	6	2	2	2	2	Carbamid
1991	5615	sp|O43766|LIAS_HUMAN	89.9	6.99E+05	1.36E+06	6.08E+06	1.75E+06	6	6	2	2	2	2	Carbamid
2793	7768	sp|Q9HD67|MYO10_HUMAN	89.82		6.36E+05			4	4	0	2	0	0	Carbamid
2793	7771	tr|A0A0A0MQX1|A0A0A0MQX1_HUMAN	89.82		6.36E+05			4	4	0	2	0	0	Carbamid
2608	7901	tr|A0A087WY54|A0A087WY54_HUMAN	89.78	4.92E+06	1.58E+06		1.29E+06	3	3	3	1	0	1	Carbamid
2608	7902	sp|Q15649|ZNHI3_HUMAN	89.78	4.92E+06	1.58E+06		1.29E+06	3	3	3	1	0	1	Carbamid
2752	9092	sp|Q86UY6|NAA40_HUMAN	89.73	4.72E+05	4.95E+05		9.99E+05	2	2	1	1	0	2	
2481	6679	tr|C9JVN9|C9JVN9_HUMAN	89.69			1.14E+06		3	3	0	0	1	0	
2481	6681	sp|Q9H9P8|L2HDH_HUMAN	89.69			1.14E+06		3	3	0	0	1	0	
2292	6327	sp|Q96CU9|FXRD1_HUMAN	89.65	2.87E+05	1.05E+05		1.66E+06	4	4	2	1	0	1	
2074	5617	sp|O43819|SCO2_HUMAN	89.58	3.97E+06	6.30E+06	3.76E+06	3.11E+05	6	6	3	3	2	1	
2916	9347	tr|F8VZ95|F8VZ95_HUMAN	89.57	7.68E+05				2	2	2	0	0	0	
2916	10037	tr|C9JX57|C9JX57_HUMAN	89.57	7.68E+05				2	2	2	0	0	0	
2916	10038	tr|D3DSE6|D3DSE6_HUMAN	89.57	7.68E+05				2	2	2	0	0	0	
2916	10039	tr|C9J818|C9J818_HUMAN	89.57	7.68E+05				2	2	2	0	0	0	
2916	10040	sp|P57076|CF298_HUMAN	89.57	7.68E+05				2	2	2	0	0	0	
2462	8507	sp|Q8N490|PNKD_HUMAN	89.53	8.84E+05		0	1.99E+06	2	2	1	0	1	1	Carbamid
2080	6157	sp|Q8N9N2|ASCC1_HUMAN	89.5	4.71E+05	7.00E+05	4.45E+06	3.43E+06	4	4	1	1	2	2	Acetylatic
2316	7098	sp|Q7Z4V5|HDGR2_HUMAN	89.2		8.06E+05	3.00E+05	5.45E+05	4	2	1	2	1	2	
2432	6289	sp|Q8TCS8|PNPT1_HUMAN	89.06			4.51E+05		4	3	0	0	1	0	
2650	6953	sp|O95232|LC7L3_HUMAN	89.01	4.03E+04	3.52E+05	3.30E+05		3	2	2	2	1	0	Carbamid
2650	6965	tr|J3KPP4|J3KPP4_HUMAN	89.01	4.03E+04	3.52E+05	3.30E+05		3	2	2	2	1	0	Carbamid
2235	4506	sp|Q9NSD9|SYFB_HUMAN	88.99	8.35E+04	3.81E+05	6.39E+05		6	6	1	2	2	0	Carbamid
1843	4244	sp|P00338|LDHA_HUMAN	88.98	4.04E+06	3.84E+05	2.06E+06	3.88E+05	6	6	7	1	3	1	Carbamid
2397	6352	sp|O60930|RNH1_HUMAN	88.88	8.83E+05	4.89E+05	4.88E+05	7.92E+05	4	4	1	1	1	1	
2651	7245	sp|Q71SY5|MED25_HUMAN	88.88		2.76E+06		1.37E+06	5	5	0	4	0	1	Carbamid
2364	7839	sp|Q8ND56|LS14A_HUMAN	88.84	6.97E+05	1.26E+06	2.18E+06	1.29E+06	3	3	1	3	2	1	Carbamid
2863	7346	sp|O96028|NSD2_HUMAN	88.73	7.40E+05				3	3	3	0	0	0	
2863	7824	tr|D6R9V2|D6R9V2_HUMAN	88.73	7.40E+05				3	3	3	0	0	0	
2444	5753	sp|Q13123|RED_HUMAN	88.7	3.11E+05	1.68E+06	5.55E+05	7.67E+05	4	4	1	4	1	1	
2370	5199	sp|Q9NYY8|FAKD2_HUMAN	88.69		0	4.67E+05	2.37E+05	5	5	0	1	1	1	Carbamid
2470	5967	sp|Q86YM7|HOME1_HUMAN	88.68	1.94E+05	3.20E+04	2.13E+05		4	2	3	2	1	0	
1879	5175	tr|F5H6Z0|F5H6Z0_HUMAN	88.63	5.88E+04	6.05E+05		9.66E+05	6	4	3	5	2	4	Deamidat
1879	5184	sp|Q00537|CDK17_HUMAN	88.63	5.88E+04	6.05E+05		9.66E+05	6	4	3	5	2	4	Deamidat
1956	5464	sp|Q9HC52|CBX8_HUMAN	88.6	1.01E+07	7.60E+06	5.16E+06	8.07E+06	5	4	5	3	3	3	Carbamid
3018	8155	sp|O15212|PFD6_HUMAN	88.3	1.12E+06		4.88E+05		4	4	2	0	1	0	
2551	6334	sp|Q9BRD0|BUD13_HUMAN	88.28	7.17E+05	1.07E+06	7.12E+05	1.41E+06	5	5	1	2	1	2	
2761	7657	sp|P50552|VASP_HUMAN	88.24	2.45E+05	1.98E+05	4.50E+05	1.02E+05	3	3	1	1	1	1	
1809	6056	sp|Q8N1N4|K2C78_HUMAN	88.18					5	2	3	3	2	1	Carbamid
2789	6314	tr|A0A3B3IRT3|A0A3B3IRT3_HUMAN	88.17		7.92E+05		2.66E+05	3	3	0	2	0	1	Carbamid
2789	6317	sp|O60343|TBCD4_HUMAN	88.17		7.92E+05		2.66E+05	3	3	0	2	0	1	Carbamid
2774	7483	sp|Q9NZI7|UBIP1_HUMAN	88.11		6.72E+04			3	2	0	1	1	0	
2386	6752	sp|Q96DB5|RMD1_HUMAN	87.96	4.47E+06	1.21E+06		1.44E+05	4	4	3	1	0	1	
2386	7578	tr|H0YB83|H0YB83_HUMAN	87.96	3.00E+06	1.21E+06		1.44E+05	3	3	2	1	0	1	
2386	7579	tr|E5RH53|E5RH53_HUMAN	87.96	3.00E+06	1.21E+06		1.44E+05	3	3	2	1	0	1	
2336	4474	sp|P28702|RXRB_HUMAN	87.77	1.05E+06			1.88E+05	6	4	3	0	1	1	Carbamid
2926	12456	sp|Q53HI1|UNC50_HUMAN	87.73				0	1	1	0	0	0	1	Acetylatic
2926	12458	tr|J3KQ47|J3KQ47_HUMAN	87.73				0	1	1	0	0	0	1	Acetylatic
2197	4910	sp|Q9BVV7|TIM21_HUMAN	87.71	2.84E+06	3.35E+06	3.60E+06	4.43E+06	6	6	4	2	2	2	
2550	6283	sp|O96011|PX11B_HUMAN	87.67	1.67E+06	5.49E+05			5	5	4	2	0	0	Carbamid
2532	7862	sp|Q6P444|MTFR2_HUMAN	87.57	5.01E+06	6.37E+05	2.34E+05	1.07E+06	3	3	2	1	1	1	
2401	10591	sp|Q9H5V9|CX056_HUMAN	87.54	1.55E+06	1.89E+06	1.73E+06	1.64E+06	2	2	1	1	2	1	
1492	6461	sp|P83881|RL36A_HUMAN	87.31	7.40E+06	5.36E+06	1.13E+07	1.40E+07	4	1	4	4	4	4	Carbamid
1492	6464	tr|J3KQN4|J3KQN4_HUMAN	87.31	7.40E+06	5.36E+06	1.13E+07	1.40E+07	4	1	4	4	4	4	Carbamid
2411	6250	sp|Q9NVF7|FBX28_HUMAN	87.22	1.22E+06		3.22E+06	1.17E+05	4	4	1	0	2	2	
2189	5626	sp|P38117|ETFB_HUMAN	87.2	9.55E+05	8.14E+05	4.54E+05	4.99E+05	5	5	2	2	1	2	Carbamid
2189	6485	tr|M0QY67|M0QY67_HUMAN	87.2	9.55E+05	8.14E+05	4.54E+05	4.99E+05	5	5	2	2	1	2	Carbamid
1915	7512	sp|P61009|SPCS3_HUMAN	87.16	1.00E+06	5.40E+05	8.23E+05	1.09E+06	3	3	3	1	1	3	Deamidat
2003	4429	sp|Q7L592|NDUF7_HUMAN	87.13	8.88E+05	1.74E+06	3.36E+05	3.95E+05	8	8	1	4	1	1	
2003	4818	tr|C9J236|C9J236_HUMAN	87.13	8.88E+05	1.69E+06	3.36E+05	3.95E+05	6	6	1	3	1	1	
2003	4820	tr|C9JS27|C9JS27_HUMAN	87.13	8.88E+05	1.69E+06	3.36E+05	3.95E+05	6	6	1	3	1	1	
2612	9503	sp|Q9BT09|CNPY3_HUMAN	86.77	1.60E+06	6.81E+05			2	2	3	1	0	0	Carbamid
2903	7922	sp|Q15361|TTF1_HUMAN	86.7	7.46E+06	2.47E+06	9.24E+05		3	3	1	1	1	0	
2279	5843	sp|Q15139|KPCD1_HUMAN	86.59	6.89E+05	4.23E+05	2.53E+06		4	4	2	1	4	0	Carbamid
2279	5844	tr|F8WBA3|F8WBA3_HUMAN	86.59	6.89E+05	4.23E+05	2.53E+06		4	4	2	1	4	0	Carbamid
2600	6369	sp|O00487|PSDE_HUMAN	86.55		9.22E+05	4.68E+05	6.06E+05	5	5	0	1	1	1	
2477	10043	sp|Q9UHA3|RLP24_HUMAN	86.49	6.15E+05		6.36E+06	2.70E+06	2	2	1	0	1	2	Carbamid
2725	6007	sp|P14735|IDE_HUMAN	86.41					4	4	0	0	0	0	
2725	6008	tr|A0A3B3ISG5|A0A3B3ISG5_HUMAN	86.41					4	4	0	0	0	0	
2556	7748	sp|Q9UK58|CCNL1_HUMAN	86.3		9.90E+05	5.14E+05	1.78E+06	4	4	0	1	2	2	
2024	6535	sp|P20674|COX5A_HUMAN	86.22	4.23E+06	2.55E+06	8.47E+05	1.64E+06	4	4	4	2	1	2	
2024	6536	tr|H3BNX8|H3BNX8_HUMAN	86.22	4.23E+06	2.55E+06	8.47E+05	1.64E+06	4	4	4	2	1	2	
2442	7837	sp|Q96HC4|PDLI5_HUMAN	86.18	7.68E+05	3.56E+05		1.75E+05	3	3	2	1	0	2	
2433	6664	sp|Q8IUW5|RELL1_HUMAN	86.17	2.03E+06		3.93E+06	2.15E+06	4	4	1	0	2	1	Carbamid
2256	4496	sp|Q96MG7|NSE3_HUMAN	86.17		1.77E+05	5.45E+05	1.59E+06	7	7	0	1	1	4	
2838	7755	sp|Q3KQU3|MA7D1_HUMAN	86.15	1.92E+06	4.85E+05		9.08E+05	3	3	1	1	0	1	
1939	5413	tr|E9PGM9|E9PGM9_HUMAN	86.08	1.21E+06	5.34E+05	8.94E+04		6	4	8	3	3	1	Carbamid
1939	5461	sp|P78332|RBM6_HUMAN	86.08	1.21E+06	5.34E+05	8.94E+04		6	4	8	3	3	1	Carbamid
1806	6452	sp|P18077|RL35A_HUMAN	86.07	2.16E+07	6.03E+07	2.40E+08	1.57E+08	4	4	3	4	4	2	Oxidatior
2389	8266	sp|Q06587|RING1_HUMAN	85.92	3.35E+05	8.04E+05			3	2	1	2	1	1	Carbamid
2483	8058	sp|P62312|LSM6_HUMAN	85.78	5.05E+05				3	3	2	0	0	0	
2430	5573	sp|O43865|SAHH2_HUMAN	85.76	3.23E+05	4.21E+05	2.27E+05		5	5	1	2	1	0	Carbamid
2645	6827	sp|Q8TD26|CHD6_HUMAN	85.61			4.21E+04		3	1	0	2	1	1	
2832	8260	sp|Q9BW85|YJU2_HUMAN	85.51	1.64E+06	1.69E+05			5	5	4	1	0	0	Carbamid
2832	8504	tr|M0R2S3|M0R2S3_HUMAN	85.51	1.01E+06	1.69E+05			4	4	3	1	0	0	Carbamid
2819	8242	tr|H7C1N2|H7C1N2_HUMAN	85.47	5.50E+04			1.64E+05	3	3	1	0	0	1	Carbamid
2819	8243	sp|O75128|COBL_HUMAN	85.47	5.50E+04			1.64E+05	3	3	1	0	0	1	Carbamid
2205	7857	sp|Q99943|PLCA_HUMAN	85.42	4.87E+05	7.24E+05	1.35E+06	2.10E+06	4	4	2	1	1	1	Carbamid
2726	6393	tr|A0A0U1RRM1|A0A0U1RRM1_HUMAN	85.36	7.81E+05	1.03E+05		6.03E+04	5	4	2	1	0	1	Carbamid
2726	6394	sp|Q8WXI9|P66B_HUMAN	85.36	7.81E+05	1.03E+05		6.03E+04	5	4	2	1	0	1	Carbamid
1910	7614	sp|Q9BQC6|RT63_HUMAN	85.34	8.92E+06	9.84E+06	1.43E+07	1.62E+07	3	3	3	2	2	2	
2072	4935	sp|Q15742|NAB2_HUMAN	85.31			9.40E+05	1.33E+05	5	4	1	0	2	1	Carbamid
2534	5496	sp|Q9BT78|CSN4_HUMAN	85.23	3.50E+05		2.32E+05		5	5	2	0	1	0	
2907	8395	sp|Q8NEJ9|NGDN_HUMAN	85.21				2.86E+06	2	2	0	0	0	1	
2027	10154	sp|P62857|RS28_HUMAN	85.21	1.26E+07	1.48E+07	1.85E+07	2.06E+07	3	2	2	2	3	2	
2287	6607	tr|A0A0A0MRE5|A0A0A0MRE5_HUMAN	84.97	2.21E+05		2.17E+05	2.50E+05	4	3	2	1	1	3	Carbamid
2287	6611	sp|Q9ULH1|ASAP1_HUMAN	84.97	2.21E+05		2.17E+05	2.50E+05	4	3	2	1	1	3	Carbamid
2601	6613	sp|O00264|PGRC1_HUMAN	84.92	1.99E+06	7.27E+05	2.04E+06	2.36E+06	4	4	2	1	1	1	Carbamid
2489	8032	sp|Q9UBF2|COPG2_HUMAN	84.68			1.71E+06	8.65E+04	3	2	1	0	1	2	Deamidat
2517	6344	sp|Q8N567|ZCHC9_HUMAN	84.68	1.11E+05	4.13E+06	3.32E+06		6	6	1	2	3	0	Carbamid
2480	6514	tr|E7ESG2|E7ESG2_HUMAN	84.67	4.62E+06	9.74E+05	1.41E+06	0	4	4	3	1	1	1	
2480	6515	sp|Q9HAW4|CLSPN_HUMAN	84.67	4.62E+06	9.74E+05	1.41E+06	0	4	4	3	1	1	1	
2471	6922	tr|A0A1B0GW44|A0A1B0GW44_HUMAN	84.45	3.04E+05	1.65E+05			3	1	1	1	0	0	
2471	6923	tr|A0A1B0GVP3|A0A1B0GVP3_HUMAN	84.45	3.04E+05	1.65E+05			3	1	1	1	0	0	
2471	6924	tr|A0A1B0GV23|A0A1B0GV23_HUMAN	84.45	3.04E+05	1.65E+05			3	1	1	1	0	0	
2471	6925	tr|A0A1B0GVD5|A0A1B0GVD5_HUMAN	84.45	3.04E+05	1.65E+05			3	1	1	1	0	0	
2471	6926	tr|A0A1B0GWE8|A0A1B0GWE8_HUMAN	84.45	3.04E+05	1.65E+05			3	1	1	1	0	0	
2471	6927	sp|P07339|CATD_HUMAN	84.45	3.04E+05	1.65E+05			3	1	1	1	0	0	
2471	7101	tr|A0A1B0GU03|A0A1B0GU03_HUMAN	84.45	3.04E+05	1.65E+05			3	1	1	1	0	0	
2471	7455	tr|H7C469|H7C469_HUMAN	84.45	3.04E+05	1.65E+05			3	1	1	1	0	0	

2471	7457	tr|A0A1B0GU92|A0A1B0GU92_HUMAN	84.45	3.04E+05	1.65E+05			3	1	1	1	0	0	
2992	7995	sp|Q92797|SYMPK_HUMAN	84.35					4	3	0	0	0	0	Carbamid
3200	10047	sp|Q99595|TI17A_HUMAN	84.26	2.69E+04	5.17E+05			2	2	1	1	0	0	Carbamid
2228	4999	sp|Q9BU76|MMTA2_HUMAN	84.18	2.86E+06	5.76E+05	1.73E+06	2.84E+05	6	6	4	1	2	2	
2880	7970	sp|Q16537|2A5E_HUMAN	84.17	3.81E+04				3	1	1	1	0	0	Carbamid
1857	9566	sp|Q9UII2|ATIF1_HUMAN	84.15	1.40E+08	1.16E+08	3.85E+07	6.92E+07	3	3	3	3	2	2	
1982	5480	tr|X6RAJ1|X6RAJ1_HUMAN	84.12	1.93E+07	2.38E+06	1.50E+06	1.80E+06	7	7	4	4	3	3	
1982	5483	sp|Q02040|AK17A_HUMAN	84.12	1.93E+07	2.38E+06	1.50E+06	1.80E+06	7	7	4	4	3	3	
2464	9125	tr|A0A087WY55|A0A087WY55_HUMAN	84.07	1.29E+06	5.66E+05	5.26E+05	6.47E+05	2	2	2	1	1	1	Acetylatic
2464	9126	sp|Q9NP79|VTA1_HUMAN	84.07	1.29E+06	5.66E+05	5.26E+05	6.47E+05	2	2	2	1	1	1	Acetylatic
2529	6594	sp|Q9NPA0|EMC7_HUMAN	84.04	2.56E+05	1.74E+05	6.19E+05	1.07E+06	4	4	1	1	1	2	
2362	7041	tr|A0A182DWI4|A0A182DWI4_HUMAN	84.02			1.54E+06	2.08E+06	4	4	0	0	2	2	Carbamid
2362	7044	sp|Q9BQE4|SELS_HUMAN	84.02			1.54E+06	2.08E+06	4	4	0	0	2	2	Carbamid
2067	5395	tr|H0YNU5|H0YNU5_HUMAN	83.98	8.50E+05	3.78E+05	7.67E+05		6	5	4	3	3	1	Carbamid
2067	5399	tr|A0A3B3IT82|A0A3B3IT82_HUMAN	83.98	8.50E+05	3.78E+05	7.67E+05		6	5	4	3	3	1	Carbamid
2067	5403	sp|P54132|BLM_HUMAN	83.98	8.50E+05	3.78E+05	7.67E+05		6	5	4	3	3	1	Carbamid
2460	6524	sp|P30307|MPIP3_HUMAN	83.96	3.97E+05	3.26E+05	1.16E+06	1.27E+05	4	4	1	1	1	1	
2224	10107	sp|Q9BVC6|TM109_HUMAN	83.92	6.21E+06	3.85E+06	6.22E+06	5.26E+06	2	2	2	1	1	1	
2516	6280	sp|Q9HCE1|MOV10_HUMAN	83.91			9.04E+05	7.76E+05	6	6	0	0	3	3	Carbamid
2394	7541	tr|H0Y485|H0Y485_HUMAN	83.75	8.40E+06		3.05E+06		3	3	4	0	2	0	Carbamid
2394	7542	tr|H0Y5K2|H0Y5K2_HUMAN	83.75	8.40E+06		3.05E+06		3	3	4	0	2	0	Carbamid
2394	7545	tr|B3KWK7|B3KWK7_HUMAN	83.75	8.40E+06		3.05E+06		3	3	4	0	2	0	Carbamid
2394	7554	tr|A6XND1|A6XND1_HUMAN	83.75	8.40E+06		3.05E+06		3	3	4	0	2	0	Carbamid
2394	7555	tr|A6XND0|A6XND0_HUMAN	83.75	8.40E+06		3.05E+06		3	3	4	0	2	0	Carbamid
2394	7558	sp|P17936|IBP3_HUMAN	83.75	8.40E+06		3.05E+06		3	3	4	0	2	0	Carbamid
2304	10176	tr|J3QS48|J3QS48_HUMAN	83.72	1.53E+06	2.36E+06	2.11E+06	1.92E+06	2	2	1	2	1	1	
2304	10177	tr|I3L295|I3L295_HUMAN	83.72	1.53E+06	2.36E+06	2.11E+06	1.92E+06	2	2	1	2	1	1	
2304	10178	tr|J3KTK8|J3KTK8_HUMAN	83.72	1.53E+06	2.36E+06	2.11E+06	1.92E+06	2	2	1	2	1	1	
2304	10179	tr|J3QQZ4|J3QQZ4_HUMAN	83.72	1.53E+06	2.36E+06	2.11E+06	1.92E+06	2	2	1	2	1	1	
2304	10180	tr|J3KT75|J3KT75_HUMAN	83.72	1.53E+06	2.36E+06	2.11E+06	1.92E+06	2	2	1	2	1	1	
2304	10181	tr|J3QW43|J3QW43_HUMAN	83.72	1.53E+06	2.36E+06	2.11E+06	1.92E+06	2	2	1	2	1	1	
2304	10182	tr|J3QRD5|J3QRD5_HUMAN	83.72	1.53E+06	2.36E+06	2.11E+06	1.92E+06	2	2	1	2	1	1	
2304	10183	sp|O75352|MPU1_HUMAN	83.72	1.53E+06	2.36E+06	2.11E+06	1.92E+06	2	2	1	2	1	1	
2393	6265	sp|Q9UHK0|NUFP1_HUMAN	83.61	1.71E+06	4.24E+06	2.26E+06	1.91E+06	4	4	1	3	2	1	Carbamid
2132	5006	sp|Q02318|CP27A_HUMAN	83.44	1.02E+06	1.05E+06		9.85E+05	6	6	5	2	0	1	
2180	6719	sp|Q9BRS2|RIOK1_HUMAN	83.39	5.21E+05	2.70E+06	5.30E+05	1.86E+06	5	5	1	6	1	3	Carbamid
2776	7966	sp|P46109|CRKL_HUMAN	83.35	1.38E+06				3	3	2	0	0	0	
2311	5622	sp|Q9H8V3|ECT2_HUMAN	83.15	3.60E+05	5.90E+04	3.05E+05	6.41E+04	5	5	4	1	1	1	Carbamid
2453	8396	tr|B4DI03|B4DI03_HUMAN	83.03	7.64E+05	3.92E+05	4.04E+05	9.96E+04	2	2	2	1	1	1	
2453	8397	tr|K7EJQ7|K7EJQ7_HUMAN	83.03	7.64E+05	3.92E+05	4.04E+05	9.96E+04	2	2	2	1	1	1	
2453	8401	sp|Q9BY50|SC11C_HUMAN	83.03	7.64E+05	3.92E+05	4.04E+05	9.96E+04	2	2	2	1	1	1	
2453	10127	tr|A0A0A0MR04|A0A0A0MR04_HUMAN	83.03	7.64E+05	3.92E+05	4.04E+05	9.96E+04	2	2	2	1	1	1	
2212	8393	sp|Q9Y4B6|DCAF1_HUMAN	82.83	1.41E+04				3	2	2	1	1	1	Carbamid
2200	7439	sp|Q86Y39|NDUAB_HUMAN	82.83	9.77E+05	1.66E+06	1.63E+06	3.38E+06	4	4	1	2	1	2	Carbamid
1839	6456	sp|P27482|CALL3_HUMAN	82.83		2.16E+05		9.46E+05	5	3	3	3	2	3	Acetylatic
2396	11813	tr|J3QSZ6|J3QSZ6_HUMAN	82.77	1.22E+06	2.03E+06	1.34E+06	2.38E+06	1	1	1	1	1	1	
2396	11814	sp|Q9NPI0|TM138_HUMAN	82.77	1.22E+06	2.03E+06	1.34E+06	2.38E+06	1	1	1	1	1	1	
3081	8005	sp|Q8NEB9|PK3C3_HUMAN	82.72			1.86E+05		3	3	0	0	1	0	
3081	8006	tr|A0A1W2PS05|A0A1W2PS05_HUMAN	82.72			1.86E+05		3	3	0	0	1	0	
3081	8085	tr|A8MYT4|A8MYT4_HUMAN	82.72			1.86E+05		3	3	0	0	1	0	
2198	5575	tr|H0Y6Y8|H0Y6Y8_HUMAN	82.65	7.40E+05	7.19E+06	1.75E+06	1.40E+06	7	7	3	2	1	1	Deamidat
2198	5580	tr|B1AL05|B1AL05_HUMAN	82.65	7.40E+05	7.19E+06	1.75E+06	1.40E+06	7	7	3	2	1	1	Deamidat
2198	5596	sp|Q8N983|RM43_HUMAN	82.65	7.40E+05	7.19E+06	1.75E+06	1.40E+06	7	7	3	2	1	1	Deamidat
2617	7055	sp|Q9NZJ9|NUDT4_HUMAN	82.41	1.56E+06	9.67E+05	1.50E+06		3	1	3	1	1	0	Carbamid
2545	7984	sp|Q9NV06|DCA13_HUMAN	82.41	0	4.02E+06			4	4	1	4	0	0	Carbamid
2545	8017	tr|A0A087WT20|A0A087WT20_HUMAN	82.41	0	4.02E+06			4	4	1	4	0	0	Carbamid
1831	4923	tr|A0A3B3ITP4|A0A3B3ITP4_HUMAN	82.38	6.50E+05	1.72E+06	5.36E+05	1.11E+06	7	7	3	4	1	4	Carbamid
1831	4924	sp|Q8IVH4|MMAA_HUMAN	82.38	6.50E+05	1.72E+06	5.36E+05	1.11E+06	7	7	3	4	1	4	Carbamid
1831	4925	tr|Q495G5|Q495G5_HUMAN	82.38	6.50E+05	1.72E+06	5.36E+05	1.11E+06	7	7	3	4	1	4	Carbamid
2463	8947	sp|Q9NQZ2|SAS10_HUMAN	82.14	8.14E+06	6.10E+06	6.25E+06	5.13E+06	2	2	1	1	2	2	
2875	7126	tr|C9JGJ9|C9JGJ9_HUMAN	82.09			4.25E+05	5.37E+05	3	3	0	0	1	1	
2875	7127	tr|F8WB99|F8WB99_HUMAN	82.09			4.25E+05	5.37E+05	3	3	0	0	1	1	
2875	7128	tr|C9IYK6|C9IYK6_HUMAN	82.09			4.25E+05	5.37E+05	3	3	0	0	1	1	
2875	7130	tr|C9JTE9|C9JTE9_HUMAN	82.09			4.25E+05	5.37E+05	3	3	0	0	1	1	
2875	7131	sp|Q9UHQ4|BAP29_HUMAN	82.09			4.25E+05	5.37E+05	3	3	0	0	1	1	
2875	7199	tr|H7C5E2|H7C5E2_HUMAN	82.09			4.25E+05	5.37E+05	3	3	0	0	1	1	
2638	12462	tr|K7EML3|K7EML3_HUMAN	82.06		2.67E+05		3.73E+05	1	1	0	1	0	1	
2638	12464	sp|Q9NUL5|RYDEN_HUMAN	82.06		2.67E+05		3.73E+05	1	1	0	1	0	1	
2669	8707	sp|Q9H9L3|I20L2_HUMAN	81.97	5.98E+05		1.51E+06	8.02E+05	2	2	2	0	1	1	Acetylatic
2162	7553	sp|Q9BRX9|WDR83_HUMAN	81.96	4.94E+05	8.84E+05	6.96E+05	8.20E+05	3	3	2	2	1	1	Carbamid
2355	6525	sp|Q96EH3|MASU1_HUMAN	81.95	6.45E+05	7.28E+05	4.50E+05		4	4	1	2	1	0	Carbamid
2426	6573	sp|O95140|MFN2_HUMAN	81.85		2.35E+05	2.16E+05		4	3	0	1	2	1	Carbamid
1994	5914	sp|Q9UG56|PISD_HUMAN	81.72	1.24E+06	7.73E+06	9.71E+05	9.33E+05	7	7	3	2	1	2	Deamidat
3037	9015	tr|A0A0G2JJL1|A0A0G2JJL1_HUMAN	81.45		8.25E+04			2	1	0	1	0	0	
3037	9016	tr|A0A140T9L0|A0A140T9L0_HUMAN	81.45		8.25E+04			2	1	0	1	0	0	
2943	10112	sp|Q96N66|MBOA7_HUMAN	81.44					2	2	0	0	0	0	
2886	8505	sp|O94887|FARP2_HUMAN	81.31	5.93E+05			1.24E+05	2	2	1	0	0	1	
2360	6271	tr|K7EJR3|K7EJR3_HUMAN	81.22	8.77E+05	2.70E+06	1.02E+06		4	4	1	3	1	0	
2360	6379	tr|R4GMR5|R4GMR5_HUMAN	81.22	8.77E+05	2.70E+06	1.02E+06		4	4	1	3	1	0	
2360	6381	sp|P48556|PSMD8_HUMAN	81.22	8.77E+05	2.70E+06	1.02E+06		4	4	1	3	1	0	
2310	5306	sp|O95347|SMC2_HUMAN	81.19			2.76E+05	3.00E+05	5	5	0	0	1	2	
2648	5638	tr|H0YJA2|H0YJA2_HUMAN	81.17		8.78E+05			5	5	0	3	0	0	Carbamid
2648	5643	sp|Q6PJT7|ZC3HE_HUMAN	81.17		8.78E+05			5	5	0	3	0	0	Carbamid
2648	5664	tr|G3V5I6|G3V5I6_HUMAN	81.17		8.78E+05			5	5	0	3	0	0	Carbamid
2648	5669	tr|G3V256|G3V256_HUMAN	81.17		8.78E+05			5	5	0	3	0	0	Carbamid
2884	8300	sp|Q16822|PCKGM_HUMAN	81.09		4.13E+04			3	3	0	1	0	0	
2884	10751	tr|A0A0A0MS74|A0A0A0MS74_HUMAN	81.09		4.13E+04			2	2	0	1	0	0	
2884	10752	tr|H0YM31|H0YM31_HUMAN	81.09		4.13E+04			2	2	0	1	0	0	
2352	8061	sp|Q01658|NC2B_HUMAN	81.03	1.90E+06	5.21E+05	1.07E+06	4.04E+06	4	4	2	2	1	2	Carbamid
2431	5833	sp|Q10471|GALT2_HUMAN	81.01	2.95E+05	1.90E+05	5.27E+05		4	4	2	3	1	0	
2269	4687	sp|Q53EZ4|CEP55_HUMAN	81	5.91E+05	1.42E+06	3.76E+06		6	6	3	3	2	0	Carbamid
2578	9002	sp|P14635|CCNB1_HUMAN	80.97	3.86E+05		3.35E+05		2	2	1	0	2	0	
2222	7974	sp|O14966|RAB7L_HUMAN	80.84	2.08E+05	4.44E+05			3	2	2	3	1	1	Carbamid
2754	9173	sp|Q96T52|IMP2L_HUMAN	80.82		6.34E+05		5.68E+05	2	2	0	1	0	1	
2277	5229	sp|O75907|DGAT1_HUMAN	80.79	4.61E+05	1.74E+06	1.05E+06	1.88E+06	6	6	1	1	2	3	Carbamid
2531	7797	sp|O60861|GAS7_HUMAN	80.77	2.95E+05	4.65E+05	1.37E+05	4.83E+05	5	2	4	2	1	2	Carbamid
2941	9315	tr|J3KNC0|J3KNC0_HUMAN	80.7	1.46E+06	0			2	2	2	1	0	0	
2941	9317	sp|P52655|TF2AA_HUMAN	80.7	1.46E+06	0			2	2	2	1	0	0	
2387	7246	sp|Q5F1R6|DJC21_HUMAN	80.65		1.33E+05	2.86E+06	9.42E+05	4	4	0	1	3	2	
2715	8902	sp|O00458|IFRD1_HUMAN	80.58	3.34E+05				2	2	1	0	0	0	
2265	7706	sp|Q96E29|MTEF3_HUMAN	80.51	5.74E+05	5.03E+05	8.91E+05	2.80E+05	3	3	2	2	1	2	Carbamid
2482	7215	tr|H0YMU3|H0YMU3_HUMAN	80.51	7.39E+05	6.30E+05			3	3	2	1	0	0	
2482	7227	tr|H0YL72|H0YL72_HUMAN	80.51	7.39E+05	6.30E+05			3	3	2	1	0	0	
2482	7228	sp|P50213|IDH3A_HUMAN	80.51	7.39E+05	6.30E+05			3	3	2	1	0	0	
2482	7551	tr|H0YLI6|H0YLI6_HUMAN	80.51	7.39E+05	6.30E+05			3	3	2	1	0	0	
2318	5398	sp|Q9NSC5|HOME3_HUMAN	80.51	1.45E+06	5.54E+05	1.03E+06		6	4	5	2	1	0	
2318	6346	tr|M0R2U7|M0R2U7_HUMAN	80.51	1.63E+05	5.54E+05	1.03E+06		4	2	3	2	1	0	
1824	7650	sp|P30613|KPYR_HUMAN	80.37	3.33E+07		1.73E+05	2.42E+07	4	2	3	2	2	5	Deamidat
2777	10152	sp|O60725|ICMT_HUMAN	80.36	3.31E+05			1.22E+06	2	2	1	0	0	1	
1827	4771	sp|P22694|KAPCB_HUMAN	80.33		5.73E+05		2.59E+05	8	2	1	8	3	2	Deamidat
1827	4772	tr|A0A0A0MS54|A0A0A0MS54_HUMAN	80.33		5.73E+05		2.59E+05	8	2	1	8	3	2	Deamidat
2183	5448	sp|Q9NUQ3|TXLNG_HUMAN	80.19					6	3	2	2	1	0	Carbamid
2708	7582	sp|Q5HYI7|MTX3_HUMAN	80.1			8.10E+05	3.97E+05	6	6	0	0	1	1	Carbamid
2484	10089	tr|C9JLU1|C9JLU1_HUMAN	80.02	1.05E+06	2.00E+06	5.39E+06	3.25E+06	2	2	2	2	1	1	
2484	10090	sp|P52434|RPAB3_HUMAN	80.02	1.05E+06	2.00E+06	5.39E+06	3.25E+06	2	2	2	2	1	1	
2225	11680	tr|C9JA28|C9JA28_HUMAN	79.89	1.15E+07	6.88E+06	1.85E+07	1.06E+07	1	1	2	2	2	1	

2225	11681	sp|Q9UNL2|SSRG_HUMAN	79.89	1.15E+07	6.88E+06	1.85E+07	1.06E+07	1	1	2	2	2	1	
2564	6522	sp|Q99633|PRP18_HUMAN	79.87		4.89E+05			4	4	0	1	0	0	
2576	8455	sp|Q9BUL9|RPP25_HUMAN	79.85	1.69E+05	6.71E+05	2.61E+06	1.82E+06	4	4	1	1	2	1	Carbamid
2747	7815	sp|Q9Y5J1|UTP18_HUMAN	79.84	1.10E+06	6.39E+05		1.17E+06	4	4	1	2	0	2	
2419	6256	sp|Q9UL26|RB22A_HUMAN	79.74	7.49E+05	5.44E+05			6	6	3	3	0	0	
2319	5430	sp|Q9UJX3|APC7_HUMAN	79.6	2.81E+06	3.19E+06	5.07E+05	4.73E+05	4	4	1	4	1	1	
2559	8119	sp|P18846|ATF1_HUMAN	79.54	1.07E+06				3	2	2	0	0	0	
3595	11630	sp|P60602|ROMO1_HUMAN	79.33				2.92E+06	1	1	0	0	0	1	Carbamid
1702	9403	sp|Q9UNZ5|L10K_HUMAN	79.29	4.94E+07	1.12E+08	2.32E+07	6.38E+07	4	4	4	5	3	4	Oxidatior
3202	10386	tr|K7ESE8|K7ESE8_HUMAN	79.17					2	2	0	0	0	0	
3202	10388	tr|J3KSD8|J3KSD8_HUMAN	79.17					2	2	0	0	0	0	
3202	10389	tr|K7ES02|K7ES02_HUMAN	79.17					2	2	0	0	0	0	
3202	10390	sp|Q13867|BLMH_HUMAN	79.17					2	2	0	0	0	0	
2672	7170	sp|Q96F86|EDC3_HUMAN	79.11	2.28E+06				4	4	3	0	0	0	
2295	7670	sp|P81605|DCD_HUMAN	79.01	2.76E+06	6.58E+06	1.71E+06	2.79E+05	3	3	2	3	1	1	Carbamid
2565	6548	tr|A0A087WW77|A0A087WW77_HUMAN	78.93	3.10E+05		6.96E+05	2.96E+05	4	4	2	0	1	1	
2565	6549	sp|Q15334|L2GL1_HUMAN	78.93	3.10E+05		6.96E+05	2.96E+05	4	4	2	0	1	1	
2566	7058	sp|Q9H4M7|PKHA4_HUMAN	78.93		4.71E+05	4.32E+05	1.02E+06	4	4	0	2	1	2	
2634	7852	tr|H0YJL5|H0YJL5_HUMAN	78.92		6.82E+05		2.12E+05	3	3	0	2	0	1	
2634	7853	sp|Q9UEU0|VTI1B_HUMAN	78.92		6.82E+05		2.12E+05	3	3	0	2	0	1	
2257	4759	sp|Q8N8R3|MCATL_HUMAN	78.84	3.78E+05	2.54E+06	3.97E+05	1.25E+06	7	7	1	4	1	1	Carbamid
2403	5520	sp|Q8TAG9|EXOC6_HUMAN	78.73	2.81E+05	2.73E+04		2.10E+05	6	3	2	1	1	1	Carbamid
2403	5667	tr|E7EW84|E7EW84_HUMAN	78.73	2.81E+05	2.73E+04		2.10E+05	5	2	2	1	1	1	
2797	7763	sp|Q9BWT3|PAPOG_HUMAN	78.68	2.97E+05	1.47E+05			4	4	2	1	0	0	Carbamid
2797	7981	tr|F8WAT4|F8WAT4_HUMAN	78.68	2.97E+05	1.47E+05			4	4	2	1	0	0	Carbamid
2390	9245	sp|P32119|PRDX2_HUMAN	78.63					2	1	1	1	1	2	Deamidat
1904	7850	tr|R4GND3|R4GND3_HUMAN	78.63	4.47E+06	3.76E+06	3.98E+06	1.33E+06	3	3	3	2	3	1	Carbamid
1904	7860	sp|Q8WV24|PHLA1_HUMAN	78.63	4.47E+06	3.76E+06	3.98E+06	1.33E+06	3	3	3	2	3	1	Carbamid
2581	5558	sp|P22033|MUTA_HUMAN	78.49	5.69E+04	2.12E+05	2.71E+05		5	5	1	1	1	0	
2302	6574	sp|Q32P28|P3H1_HUMAN	78.39	1.54E+05	2.31E+06		2.82E+05	4	4	1	4	0	1	Carbamid
3093	8787	tr|A0A087WUI6|A0A087WUI6_HUMAN	78.35					2	2	0	0	0	0	
3093	8790	sp|Q8WXW3|PIBF1_HUMAN	78.35					2	2	0	0	0	0	
2413	5766	sp|Q9UGI8|TES_HUMAN	78.35	1.10E+06	4.61E+05	4.19E+05		4	4	2	2	1	0	Carbamid
2730	8031	tr|C9JH19|C9JH19_HUMAN	78.15					3	1	0	0	0	0	Acetylatic
2734	8418	sp|Q13561|DCTN2_HUMAN	78.08		1.70E+05		1.42E+05	2	2	0	1	0	1	
2734	9388	tr|H0YI98|H0YI98_HUMAN	78.08		1.70E+05		1.42E+05	2	2	0	1	0	1	
3007	8195	sp|Q9Y4F1|FARP1_HUMAN	78.04			2.41E+05		5	4	1	0	1	0	Deamidat
3007	8200	tr|C9JME2|C9JME2_HUMAN	78.04			2.41E+05		5	4	1	0	1	0	Deamidat
2535	6572	sp|O00459|P85B_HUMAN	77.86		4.80E+04			4	2	1	1	1	0	
2817	6685	sp|Q8N5C6|SRBD1_HUMAN	77.77				3.71E+05	3	3	0	0	0	2	
2962	10146	tr|F2Z2T2|F2Z2T2_HUMAN	77.7	7.89E+05				2	2	1	0	0	0	Acetylatic
2962	10147	sp|P23025|XPA_HUMAN	77.7	7.89E+05				2	2	1	0	0	0	Acetylatic
2611	8979	tr|F5H6U2|F5H6U2_HUMAN	77.35	1.11E+06	1.61E+05	1.15E+06		2	2	2	1	2	0	Carbamid
2611	8980	tr|F5GZ82|F5GZ82_HUMAN	77.35	1.11E+06	1.61E+05	1.15E+06		2	2	2	1	2	0	Carbamid
2611	8981	sp|Q9NP50|SHCAF_HUMAN	77.35	1.11E+06	1.61E+05	1.15E+06		2	2	2	1	2	0	Carbamid
2441	7219	tr|G3V192|G3V192_HUMAN	77.33	1.75E+06	1.38E+06	1.34E+06	1.89E+06	4	4	2	1	2	1	Carbamid
2441	7222	sp|P02794|FRIH_HUMAN	77.33	1.75E+06	1.38E+06	1.34E+06	1.89E+06	4	4	2	1	2	1	Carbamid
2441	7509	tr|G3V1D1|G3V1D1_HUMAN	77.33	1.75E+06	1.38E+06	1.34E+06	1.89E+06	4	4	2	1	2	1	Carbamid
2833	8369	tr|A0A2R8Y7B8|A0A2R8Y7B8_HUMAN	77.22	8.27E+04	3.13E+04	8.51E+05		3	3	1	1	1	0	Carbamid
2940	9247	sp|Q6P1S2|CC033_HUMAN	77.22	4.54E+04	0	8.72E+04		2	2	1	1	1	0	
2398	7538	sp|Q15006|EMC2_HUMAN	77.08	3.95E+05		3.33E+05	3.87E+05	3	3	2	0	1	1	
2946	10609	sp|O15116|LSM1_HUMAN	76.88				0	2	2	0	0	0	1	Acetylatic
2630	12059	sp|Q9BW72|HIG2A_HUMAN	76.68		1.09E+06	3.28E+06	2.37E+06	1	1	0	1	1	1	Acetylatic
2152	10783	sp|P51159|RB27A_HUMAN	76.66	2.13E+05				2	1	3	2	2	1	
2094	9364	sp|P20340|RAB6A_HUMAN	76.6					2	1	2	2	2	1	
2094	9365	sp|Q9NRW1|RAB6B_HUMAN	76.6					2	1	2	2	2	1	
2452	8380	tr|F8VUA2|F8VUA2_HUMAN	76.58	4.37E+05	1.05E+06		1.70E+06	3	3	1	1	0	1	Acetylatic
2452	8382	sp|Q9HD42|CHM1A_HUMAN	76.58	4.37E+05	1.05E+06		1.70E+06	3	3	1	1	0	1	Acetylatic
3551	11441	sp|P05089|ARGI1_HUMAN	76.5					1	1	0	0	0	0	
2299	8098	tr|A0A0C4DFV9|A0A0C4DFV9_HUMAN	76.46	1.12E+06		5.06E+05		3	3	3	0	1	0	
2299	8099	sp|Q01105|SET_HUMAN	76.46	1.12E+06		5.06E+05		3	3	3	0	1	0	
2218	6621	tr|F5H1S8|F5H1S8_HUMAN	76.45	6.79E+05	7.39E+05	5.35E+05	4.51E+05	3	3	2	2	2	1	
2218	6625	sp|Q14165|MLEC_HUMAN	76.45	6.79E+05	7.39E+05	5.35E+05	4.51E+05	3	3	2	2	2	1	
2618	8009	sp|P36915|GNL1_HUMAN	76.39		1.54E+05	8.88E+05		3	3	0	2	1	0	Carbamid
2670	8939	sp|Q15283|RASA2_HUMAN	76.26	4.40E+05		2.40E+05	6.66E+04	2	2	2	0	1	1	Carbamid
2670	8940	tr|A0A0A0MSJ9|A0A0A0MSJ9_HUMAN	76.26	4.40E+05		2.40E+05	6.66E+04	2	2	2	0	1	1	Carbamid
2720	9147	tr|F8W1A4|F8W1A4_HUMAN	76.24	2.74E+05	5.64E+05			2	2	1	2	0	0	Deamidat
2720	9148	sp|P54819|KAD2_HUMAN	76.24	2.74E+05	5.64E+05			2	2	1	2	0	0	Deamidat
2720	9224	tr|F8VZG5|F8VZG5_HUMAN	76.24	2.74E+05	5.64E+05			2	2	1	2	0	0	Deamidat
2720	10148	tr|G3V213|G3V213_HUMAN	76.24	2.74E+05	5.64E+05			2	2	1	2	0	0	Deamidat
2289	10437	sp|P56378|68MP_HUMAN	76.24	1.93E+06	1.91E+06		2.86E+06	3	2	2	1	1	2	
2289	10438	tr|A0A0A0MTQ5|A0A0A0MTQ5_HUMAN	76.24	1.93E+06	1.91E+06		2.86E+06	3	2	2	1	1	2	
2289	10440	tr|G3V556|G3V556_HUMAN	76.24	1.93E+06	1.91E+06		2.86E+06	3	2	2	1	1	2	
2930	7080	sp|A0A024RBG1|NUD4B_HUMAN	76.21	1.04E+06				3	1	3	0	0	0	Carbamid
3128	8526	sp|Q15773|MLF2_HUMAN	76.2					2	2	0	0	0	0	Deamidat
2625	10496	sp|Q6NTF9|RHBD2_HUMAN	76.13	3.48E+05	6.92E+05	8.24E+05	4.69E+05	2	2	1	2	1	1	
2213	8850	sp|Q71F23|CENPU_HUMAN	76.11	7.80E+05	5.69E+05	1.60E+06	9.14E+05	2	2	2	3	1	2	
2512	5908	sp|Q9BW92|SYTM_HUMAN	76.11			6.41E+05	1.20E+06	4	4	0	0	1	4	
2309	4916	sp|A5YKK6|CNOT1_HUMAN	76.08	5.74E+05	7.34E+05	2.74E+05	1.14E+07	8	8	2	3	1	1	Deamidat
2659	7093	sp|Q14CX7|NAA25_HUMAN	75.85	2.04E+05	2.16E+05			4	4	1	2	0	0	
2571	7834	tr|B8ZZS2|B8ZZS2_HUMAN	75.41					4	3	1	0	1	0	
2571	7865	sp|Q13506|NAB1_HUMAN	75.41					4	3	1	0	1	0	
3657	12504	sp|P15259|PGAM2_HUMAN	75.37					1	1	0	0	0	0	
3657	12505	sp|P18669|PGAM1_HUMAN	75.37					1	1	0	0	0	0	
3657	12506	sp|Q8N0Y7|PGAM4_HUMAN	75.37					1	1	0	0	0	0	
2910	9030	sp|O60503|ADCY9_HUMAN	75.35		2.57E+04			3	3	0	1	0	0	Carbamid
3170	9655	sp|Q2M2I8|AAK1_HUMAN	75.13					2	1	0	0	1	0	Carbamid
2746	7728	tr|I3L1K7|I3L1K7_HUMAN	75.12				4.60E+05	3	3	0	0	0	1	Acetylatic
2746	7729	tr|A0A1W2PR23|A0A1W2PR23_HUMAN	75.12				4.60E+05	3	3	0	0	0	1	Acetylatic
2746	7730	tr|A0A1W2PPW8|A0A1W2PPW8_HUMAN	75.12				4.60E+05	3	3	0	0	0	1	Acetylatic
2746	7731	tr|A0A1W2PR02|A0A1W2PR02_HUMAN	75.12				4.60E+05	3	3	0	0	0	1	Acetylatic
2746	7733	tr|A0A1W2PRV0|A0A1W2PRV0_HUMAN	75.12				4.60E+05	3	3	0	0	0	1	Acetylatic
2746	7734	tr|A0A1W2PRP7|A0A1W2PRP7_HUMAN	75.12				4.60E+05	3	3	0	0	0	1	Acetylatic
2746	7735	tr|A0A1W2PPG5|A0A1W2PPG5_HUMAN	75.12				4.60E+05	3	3	0	0	0	1	Acetylatic
2746	7736	tr|A0A1W2PPP5|A0A1W2PPP5_HUMAN	75.12				4.60E+05	3	3	0	0	0	1	Acetylatic
2746	7737	tr|A0A1W2PPG1|A0A1W2PPG1_HUMAN	75.12				4.60E+05	3	3	0	0	0	1	Acetylatic
2746	7738	sp|O14653|GOSR2_HUMAN	75.12				4.60E+05	3	3	0	0	0	1	Acetylatic
2746	7739	tr|A0A1W2PQM3|A0A1W2PQM3_HUMAN	75.12				4.60E+05	3	3	0	0	0	1	Acetylatic
2746	7740	tr|A0A1W2PS81|A0A1W2PS81_HUMAN	75.12				4.60E+05	3	3	0	0	0	1	Acetylatic
2746	7741	tr|E7EQ34|E7EQ34_HUMAN	75.12				4.60E+05	3	3	0	0	0	1	Acetylatic
2746	7742	tr|I3NI02|I3NI02_HUMAN	75.12				4.60E+05	3	3	0	0	0	1	Acetylatic
2158	10558	sp|O00194|RB27B_HUMAN	75.08		4.36E+05	1.99E+06		2	1	2	3	3	1	Acetylatic
2135	6984	sp|Q96LL9|DJC30_HUMAN	74.98		1.46E+06		2.36E+06	4	4	0	4	0	2	
3184	11571	sp|Q9HCC0|MCCB_HUMAN	74.96					1	1	0	0	0	0	
3184	12497	tr|D6RD67|D6RD67_HUMAN	74.96					1	1	0	0	0	0	
2811	6419	tr|H0Y360|H0Y360_HUMAN	74.85	3.65E+05	4.79E+05		1.29E+05	5	5	1	1	0	2	
2811	6420	sp|Q01433|AMPD2_HUMAN	74.85	3.65E+05	4.79E+05		1.29E+05	5	5	1	1	0	2	
2099	6663	sp|O75525|KHDR3_HUMAN	74.77			1.50E+06	4.34E+05	4	3	3	1	1	2	
2796	7550	sp|Q5VT06|CE350_HUMAN	74.61	2.06E+06	1.55E+06			5	5	3	3	0	0	
2307	7181	tr|H0Y390|H0Y390_HUMAN	74.47			7.02E+04	3.16E+05	6	4	1	0	1	4	
2307	7689	sp|Q9UPN3|MACF1_HUMAN	74.47			7.02E+04	3.16E+05	7	4	1	1	1	4	
2307	7707	tr|H3BPE1|H3BPE1_HUMAN	74.47			7.02E+04	3.16E+05	8	4	1	1	1	5	
2307	7713	tr|H3BQK9|H3BQK9_HUMAN	74.47			7.02E+04	3.16E+05	8	4	1	1	1	5	
2723	9849	sp|P62877|RBX1_HUMAN	74.44	1.32E+06		1.90E+06	1.99E+06	1	1	1	0	1	1	Acetylatic
2629	11832	tr|A0A0B4J2F8|A0A0B4J2F8_HUMAN	74.42		9.57E+05	5.56E+05	2.38E+05	2	2	0	2	1	2	
2629	11833	sp|Q9GZM3|RPB1B_HUMAN	74.42		9.57E+05	5.56E+05	2.38E+05	2	2	0	2	1	2	

2629	11834	sp|P52435|RPB11_HUMAN	74.42		9.57E+05	5.56E+05	2.38E+05	2	2	0	2	1	2	
2629	11835	tr|A6NFM0|A6NFM0_HUMAN	74.42		9.57E+05	5.56E+05	2.38E+05	2	2	0	2	1	2	
2629	11836	tr|F6S314|F6S314_HUMAN	74.42		9.57E+05	5.56E+05	2.38E+05	2	2	0	2	1	2	
2629	11837	tr|E2QRJ6|E2QRJ6_HUMAN	74.42		9.57E+05	5.56E+05	2.38E+05	2	2	0	2	1	2	
2629	12633	sp|Q9H1A7|RPB1C_HUMAN	74.42		9.57E+05	5.56E+05	2.38E+05	2	2	0	2	1	2	
3100	10739	sp|Q16864|VATF_HUMAN	74.34					1	1	0	0	0	0	
2115	6231	sp|Q14185|DOCK1_HUMAN	74.22			1.21E+05		5	4	1	1	2	1	Carbamid
2115	6247	tr|A0A096LNH6|A0A096LNH6_HUMAN	74.22			1.21E+05		5	4	1	1	2	1	Carbamid
3174	10645	sp|Q96D70|R3HD4_HUMAN	74.15		8.20E+04			2	2	0	1	0	0	Carbamid
2839	7880	tr|F5H1N7|F5H1N7_HUMAN	74.1		1.39E+05		6.12E+05	3	3	0	1	0	1	
2839	7893	sp|Q6ZU35|K1211_HUMAN	74.1		1.39E+05		6.12E+05	3	3	0	1	0	1	
2619	8497	sp|Q9BQ24|ZFY21_HUMAN	73.98	1.25E+06	3.84E+05		4.63E+05	2	2	2	1	0	1	Carbamid
2633	7589	tr|H0YNG3|H0YNG3_HUMAN	73.96	0	8.29E+04			3	3	1	1	0	0	
2633	7591	sp|P67812|SC11A_HUMAN	73.96	0	8.29E+04			3	3	1	1	0	0	
2291	5976	sp|O95707|RPP29_HUMAN	73.93				2.51E+06	5	5	0	0	0	2	Carbamid
2690	6388	sp|Q96QK1|VPS35_HUMAN	73.86	1.55E+04			1.32E+05	4	4	1	0	0	2	Carbamid
3189	12105	tr|F8WCQ4|F8WCQ4_HUMAN	73.84				4.45E+05	1	1	0	0	0	1	
3189	12106	tr|A0A1W2PS40|A0A1W2PS40_HUMAN	73.84				4.45E+05	1	1	0	0	0	1	
3189	12107	tr|C9JNP8|C9JNP8_HUMAN	73.84				4.45E+05	1	1	0	0	0	1	
3189	12108	sp|Q9H300|PARL_HUMAN	73.84				4.45E+05	1	1	0	0	0	1	
3189	12109	tr|A0A1W2PP11|A0A1W2PP11_HUMAN	73.84				4.45E+05	1	1	0	0	0	1	
3189	12508	tr|H7C0U0|H7C0U0_HUMAN	73.84				4.45E+05	1	1	0	0	0	1	
2604	7171	sp|Q16775|GLO2_HUMAN	73.84	8.05E+05	6.45E+05			3	3	1	1	0	0	
2447	6800	tr|I3L2L5|I3L2L5_HUMAN	73.82	4.84E+05	6.63E+05		7.37E+05	4	4	1	1	0	1	
2447	6801	sp|C9JLW8|MCRI1_HUMAN	73.82	4.84E+05	6.63E+05		7.37E+05	4	4	1	1	0	1	
2447	6805	tr|I3L4Q0|I3L4Q0_HUMAN	73.82	4.84E+05	6.63E+05		7.37E+05	4	4	1	1	0	1	
2160	10691	sp|Q9H082|RB33B_HUMAN	73.82					2	1	2	2	2	1	
2262	5312	sp|Q14997|PSME4_HUMAN	73.79	2.76E+04	2.34E+05	7.02E+05	8.43E+05	8	8	1	2	1	4	Deamidat
2750	8510	sp|P56937|DHB7_HUMAN	73.57	3.16E+05	5.73E+05			2	2	1	1	0	0	Carbamid
1815	10174	sp|P62875|RPAB5_HUMAN	73.55	6.05E+06	9.33E+06	1.45E+07	1.55E+07	2	2	2	3	3	3	
2359	5948	sp|P07195|LDHB_HUMAN	73.47	2.34E+06	1.23E+06	2.41E+05		5	5	3	3	2	0	
2417	5366	sp|O60306|AQR_HUMAN	73.28		5.32E+05	1.40E+06	8.09E+05	5	5	0	2	2	2	
2722	9231	sp|Q9Y232|CDYL_HUMAN	73.21	2.84E+05	6.16E+05	3.38E+05		2	2	1	1	1	0	Deamidat
2123	9011	tr|D6RHX1|D6RHX1_HUMAN	73.21	5.96E+06	1.30E+07	2.83E+06	1.13E+07	2	2	3	4	1	3	
2123	9012	sp|Q8TAX7|MUC7_HUMAN	73.21	5.96E+06	1.30E+07	2.83E+06	1.13E+07	2	2	3	4	1	3	
3171	10150	tr|Q5T179|Q5T179_HUMAN	73.17	7.60E+05				2	2	2	0	0	0	
3171	10151	sp|P61024|CKS1_HUMAN	73.17	7.60E+05				2	2	2	0	0	0	
2348	5732	tr|C9JFE4|C9JFE4_HUMAN	73.04	3.63E+05	2.22E+05	4.45E+05	4.21E+05	5	5	1	1	1	2	
2348	5733	tr|A0A096LP07|A0A096LP07_HUMAN	73.04	3.63E+05	2.22E+05	4.45E+05	4.21E+05	5	5	1	1	1	2	
2348	5734	tr|A0A096LPJ3|A0A096LPJ3_HUMAN	73.04	3.63E+05	2.22E+05	4.45E+05	4.21E+05	5	5	1	1	1	2	
2348	5735	sp|Q13098|CSN1_HUMAN	73.04	3.63E+05	2.22E+05	4.45E+05	4.21E+05	5	5	1	1	1	2	
2348	5737	tr|A8K070|A8K070_HUMAN	73.04	3.63E+05	2.22E+05	4.45E+05	4.21E+05	5	5	1	1	1	2	
2765	8174	sp|Q9BS26|ERP44_HUMAN	72.95		3.04E+05	6.24E+05	1.50E+05	3	3	0	1	1	1	Carbamid
3082	8100	sp|Q5TKA1|LIN9_HUMAN	72.94		2.34E+05			3	3	0	1	0	0	
3082	8101	tr|H0Y322|H0Y322_HUMAN	72.94		2.34E+05			3	3	0	1	0	0	
3082	8163	tr|C9J5J4|C9J5J4_HUMAN	72.94		2.34E+05			3	3	0	1	0	0	
3082	8164	tr|H7C4T7|H7C4T7_HUMAN	72.94		2.34E+05			3	3	0	1	0	0	
2876	7259	sp|Q9H269|VPS16_HUMAN	72.92	3.81E+04	7.57E+04			4	4	1	1	0	0	
2760	7144	sp|O15460|P4HA2_HUMAN	72.84			7.42E+04	4.01E+05	4	4	0	0	1	1	
2849	6828	sp|Q96GA3|LTV1_HUMAN	72.64	9.21E+05	1.02E+05		1.71E+05	3	3	1	1	0	1	
2728	7762	sp|Q969X5|ERGI1_HUMAN	72.5		1.89E+05		1.35E+05	3	3	0	1	0	1	
2408	7266	tr|D6RA03|D6RA03_HUMAN	72.33	5.50E+05	0		1.23E+06	3	3	1	1	0	2	Carbamid
2408	7613	sp|Q9UQ26|RIMS2_HUMAN	72.33	5.50E+05	0		1.23E+06	3	3	1	1	0	2	Carbamid
2204	7102	sp|Q9H7E9|CH033_HUMAN	72.29	6.45E+04	2.22E+05	1.05E+06	9.06E+06	5	5	1	1	1	4	Carbamid
2204	7105	tr|A0A3B3IT54|A0A3B3IT54_HUMAN	72.29	6.45E+04	2.22E+05	1.05E+06	9.06E+06	5	5	1	1	1	4	Carbamid
2706	7203	tr|H7BXI1|H7BXI1_HUMAN	72.27	2.76E+05	1.86E+05			3	3	2	1	0	0	Carbamid
2706	7204	sp|A0FGR8|ESYT2_HUMAN	72.27	2.76E+05	1.86E+05			3	3	2	1	0	0	Carbamid
2706	7205	tr|A0A087WXU3|A0A087WXU3_HUMAN	72.27	2.76E+05	1.86E+05			3	3	2	1	0	0	Carbamid
2696	7859	sp|P48960|CD97_HUMAN	72.08	7.82E+05		3.71E+05	2.36E+05	3	3	1	0	1	2	Carbamid
2552	6826	sp|Q53R41|FAKD1_HUMAN	72	1.77E+05	6.18E+05	7.57E+05	3.86E+05	4	4	1	2	1	1	
2572	7882	sp|P10599|THIO_HUMAN	71.99	1.23E+06	3.39E+06		4.08E+05	3	3	1	3	0	2	Carbamid
2795	7232	tr|M0QZS0|M0QZS0_HUMAN	71.93	3.16E+05			1.64E+05	4	4	1	0	0	1	
2795	7236	tr|A0A087WZG4|A0A087WZG4_HUMAN	71.93	3.16E+05			1.64E+05	4	4	1	0	0	1	
2795	7238	tr|A0A3B3IPE9|A0A3B3IPE9_HUMAN	71.93	3.16E+05			1.64E+05	4	4	1	0	0	1	
2795	7240	sp|Q6ZSZ5|ARHGI_HUMAN	71.93	3.16E+05			1.64E+05	4	4	1	0	0	1	
2456	8985	sp|O95900|TRUB2_HUMAN	71.91	2.21E+05	3.46E+05		2.55E+05	2	2	1	2	0	1	Carbamid
2472	8079	sp|O95057|DIRA1_HUMAN	71.85	9.97E+04	1.33E+05			3	2	1	3	0	0	
2665	5543	tr|A0A087WXN9|A0A087WXN9_HUMAN	71.79	5.36E+05	5.61E+05		2.71E+05	5	5	1	1	0	1	
2665	5544	sp|Q765P7|MTSSL_HUMAN	71.79	5.36E+05	5.61E+05		2.71E+05	5	5	1	1	0	1	
2654	6743	tr|A0A087X0K1|A0A087X0K1_HUMAN	71.54	3.44E+06	2.46E+06	2.32E+06	1.16E+06	5	3	3	3	1	1	
2654	6744	sp|Q9Y376|CAB39_HUMAN	71.54	3.44E+06	2.46E+06	2.32E+06	1.16E+06	5	3	3	3	1	1	
2939	9044	sp|Q9UER7|DAXX_HUMAN	71.51				2.45E+05	2	2	0	0	0	2	Carbamid
2179	6335	sp|Q674X7|KAZRN_HUMAN	71.49	3.84E+06	1.53E+06	0	1.11E+06	5	5	3	1	1	1	Carbamid
2179	6338	tr|A0A1B0GWK2|A0A1B0GWK2_HUMAN	71.49	3.84E+06	1.53E+06	0	1.11E+06	5	5	3	1	1	1	Carbamid
2569	7546	sp|Q9NW15|ANO10_HUMAN	71.46		4.49E+05			3	3	0	1	0	0	
2868	8775	sp|Q96S52|PIGS_HUMAN	71.45	1.41E+05	1.49E+05	1.41E+05		2	2	1	1	1	0	
2874	6931	sp|O75717|WDHD1_HUMAN	71.41					3	3	0	0	0	0	
2557	7777	sp|Q96QE5|TEFM_HUMAN	71.33	1.44E+05	2.71E+05		3.36E+06	5	5	1	1	0	3	
2902	7832	sp|Q5SW96|ARH_HUMAN	71.18	1.76E+06				3	3	3	0	0	0	
2955	12112	tr|A0A087WU03|A0A087WU03_HUMAN	70.99				2.43E+05	1	1	0	0	0	1	Acetylatic
2069	5791	tr|I3L4J1|I3L4J1_HUMAN	70.94				7.29E+05	4	3	1	0	1	3	Carbamid
2069	6014	sp|Q9UN37|VPS4A_HUMAN	70.94				7.29E+05	4	3	1	0	1	3	Carbamid
3143	10236	tr|H3BQH3|H3BQH3_HUMAN	70.92		1.19E+06			2	2	0	2	0	0	
3143	10238	sp|Q8TBB5|KLDC4_HUMAN	70.92		1.19E+06			2	2	0	2	0	0	
2826	6652	sp|Q6ZRP7|QSOX2_HUMAN	70.86	9.48E+04	7.78E+04		2.93E+04	3	3	1	1	0	1	
2273	6495	sp|Q5JTZ9|SYAM_HUMAN	70.79	2.53E+05	3.39E+05		9.27E+05	5	5	1	2	0	1	
2865	8071	sp|P23470|PTPRG_HUMAN	70.76	1.36E+05				3	3	2	0	0	0	
2759	6901	sp|Q5VV42|CDKAL_HUMAN	70.61	3.44E+05				4	4	1	0	0	0	
3213	12520	sp|P17152|TMM11_HUMAN	70.55				1.70E+06	1	1	0	0	0	1	
2909	8840	tr|H0Y7F0|H0Y7F0_HUMAN	70.46	6.47E+04				2	2	1	0	0	0	
2909	8841	tr|H0Y9H6|H0Y9H6_HUMAN	70.46	6.47E+04				2	2	1	0	0	0	
2909	8842	tr|E9PDE4|E9PDE4_HUMAN	70.46	6.47E+04				2	2	1	0	0	0	
2909	8843	tr|E7ES10|E7ES10_HUMAN	70.46	6.47E+04				2	2	1	0	0	0	
2909	8844	tr|E9PCH5|E9PCH5_HUMAN	70.46	6.47E+04				2	2	1	0	0	0	
2909	8845	tr|E7EVY3|E7EVY3_HUMAN	70.46	6.47E+04				2	2	1	0	0	0	
2909	8846	tr|B7Z574|B7Z574_HUMAN	70.46	6.47E+04				2	2	1	0	0	0	
2909	8847	sp|P20810|ICAL_HUMAN	70.46	6.47E+04				2	2	1	0	0	0	
2909	8848	tr|A0A0C4DGB5|A0A0C4DGB5_HUMAN	70.46	6.47E+04				2	2	1	0	0	0	
2694	7608	tr|H0YJ34|H0YJ34_HUMAN	70.45	1.21E+06				4	4	4	0	0	0	Carbamid
2694	7610	sp|Q96AC1|FERM2_HUMAN	70.45	1.21E+06				4	4	4	0	0	0	Carbamid
2694	7890	tr|A0A0U1RRM8|A0A0U1RRM8_HUMAN	70.45	9.28E+05				3	3	3	0	0	0	Carbamid
2911	9060	sp|Q5QJE6|TDIF2_HUMAN	70.41	0		3.25E+05		2	2	1	0	2	0	
3125	8331	sp|P00558|PGK1_HUMAN	70.27	1.31E+06				3	3	3	0	0	0	Carbamid
2727	7096	sp|P43121|MUC18_HUMAN	70.14	5.92E+05	7.48E+05			3	3	1	1	0	0	
2945	10536	sp|Q8WW12|PCNP_HUMAN	70.11	3.43E+06	1.86E+06			2	2	2	1	0	0	
2558	7786	sp|Q9P2H3|IFT80_HUMAN	70.02	1.61E+05	2.03E+05			3	3	1	4	0	0	
2466	10212	tr|K7ENP1|K7ENP1_HUMAN	69.95	3.00E+05		7.99E+05		2	2	2	0	2	0	
2466	10213	sp|Q7Z4G4|TRM11_HUMAN	69.95	3.00E+05		7.99E+05		2	2	2	0	2	0	
2542	6518	sp|Q8TBN0|R3GEF_HUMAN	69.91	9.28E+05	3.87E+05	4.32E+05		5	5	2	2	2	0	Carbamid
3048	8231	sp|Q14966|ZN638_HUMAN	69.91	4.15E+05				3	3	1	0	0	0	
3048	9650	tr|A0A096LPK8|A0A096LPK8_HUMAN	69.91					2	2	0	0	0	0	
3169	9465	tr|M0R0Y2|M0R0Y2_HUMAN	69.87					2	2	0	0	0	0	
3169	9466	sp|P54920|SNAA_HUMAN	69.87					2	2	0	0	0	0	
2554	6943	sp|Q9H7E2|TDRD3_HUMAN	69.87		8.21E+05		2.01E+06	3	3	0	2	0	2	
2684	8424	tr|F8W689|F8W689_HUMAN	69.79			2.04E+06	0	3	3	0	0	1	1	
2684	8425	sp|P48382|RFX5_HUMAN	69.79			2.04E+06	0	3	3	0	0	1	1	

2853	8137	tr|A0A0A0MS68|A0A0A0MS68_HUMAN	69.76			2.38E+06		4	1	3	0	1	0	Carbamid
2378	8653	sp|Q9BPZ7|SIN1_HUMAN	69.7	7.07E+05	6.17E+05	5.15E+05	4.38E+05	2	2	3	2	1	1	
3141	9822	sp|P24390|ERD21_HUMAN	69.61	8.68E+05				2	2	1	0	0	0	
2349	7065	sp|Q86TB9|PATL1_HUMAN	69.59		2.70E+05	2.55E+06	3.22E+05	5	4	0	1	5	2	
2106	6263	sp|P0C7P0|CISD3_HUMAN	69.58		4.00E+06		4.75E+06	5	4	1	4	0	7	Carbamid
2195	6840	sp|P13073|COX41_HUMAN	69.58	1.46E+06	5.23E+05	1.25E+06	1.23E+06	4	4	1	2	1	1	
2756	9620	sp|Q969H6|POP5_HUMAN	69.51		0	2.30E+06	2.28E+06	1	1	0	1	1	1	Carbamid
3083	8175	tr|F8WAJ0|F8WAJ0_HUMAN	69.4		1.23E+06	3.78E+05		3	3	0	1	1	0	Carbamid
3083	8214	sp|Q9H8H2|DDX31_HUMAN	69.4		1.23E+06	3.78E+05		3	3	0	1	1	0	Carbamid
2399	7743	tr|E9PIF4|E9PIF4_HUMAN	69.39		6.71E+05	1.26E+05		4	3	1	4	1	1	Carbamid
2399	7744	sp|Q99519|NEUR1_HUMAN	69.39		6.71E+05	1.26E+05		4	3	1	4	1	1	Carbamid
2493	11013	sp|Q9NRP0|OSTC_HUMAN	69.38	3.63E+06	5.04E+06	4.38E+06	6.80E+06	2	2	1	1	1	2	Carbamid
2493	12542	tr|A0A087WUD3|A0A087WUD3_HUMAN	69.38	3.63E+06	5.04E+06	4.38E+06	6.45E+06	1	1	1	1	1	1	Carbamid
2553	6906	sp|P63151|2ABA_HUMAN	69.35	4.62E+05	5.60E+05	6.04E+05		3	3	1	1	1	0	
2872	6660	tr|A0A1B0GW05|A0A1B0GW05_HUMAN	69.18	9.24E+04		7.68E+04	8.27E+04	3	3	1	0	1	1	
1998	6656	sp|Q14344|GNA13_HUMAN	69.18	5.69E+05				4	1	3	2	2	2	
2451	8219	sp|Q86W50|MET16_HUMAN	69.13			1.65E+06	2.10E+06	3	3	0	0	2	2	
2505	5824	sp|Q9C0D5|TANC1_HUMAN	69.12	1.01E+05	3.74E+06	2.16E+05	2.62E+07	10	10	1	2	1	2	Carbamid
2329	5758	sp|Q8N3C0|ASCC3_HUMAN	69.03	8.92E+04	3.40E+05	1.30E+06	3.78E+05	8	8	1	2	3	2	Carbamid
2681	5982	sp|P37287|PIGA_HUMAN	68.91		6.75E+04	3.78E+04	4.77E+04	4	4	0	2	1	1	Carbamid
2812	6612	sp|Q9UPP1|PHF8_HUMAN	68.9	8.54E+05	1.37E+05	6.80E+05	1.92E+05	3	3	1	1	1	1	Deamidat
2812	6775	tr|H0Y3N9|H0Y3N9_HUMAN	68.9	8.54E+05	1.37E+05	6.80E+05	1.92E+05	3	3	1	1	1	1	Deamidat
2506	6260	sp|Q15020|SART3_HUMAN	68.85	1.47E+06			1.84E+05	8	8	1	0	0	1	Carbamid
2882	8142	sp|O75530|EED_HUMAN	68.84			6.71E+05	6.30E+05	3	3	0	0	1	1	
2440	7188	sp|Q15172|2A5A_HUMAN	68.61	5.77E+05				5	4	2	1	0	0	
2458	5910	sp|Q01518|CAP1_HUMAN	68.59	9.47E+05	9.32E+05		2.36E+05	5	5	2	2	0	1	Carbamid
2621	9163	sp|Q6QNY1|BL1S2_HUMAN	68.56	3.84E+05	7.57E+05			2	2	1	1	0	0	Carbamid
2297	9636	sp|P11166|GTR1_HUMAN	68.48	2.04E+06	1.81E+06	7.20E+05	5.31E+05	3	3	2	3	1	1	
3168	9100	sp|Q13144|EI2BE_HUMAN	68.42					2	2	0	0	0	0	Carbamid
3168	9101	tr|A0A3B3IUB1|A0A3B3IUB1_HUMAN	68.42					2	2	0	0	0	0	Carbamid
2958	7891	sp|Q13501|SQSTM_HUMAN	68.36	1.66E+05			2.22E+05	3	3	1	0	0	1	Carbamid
2768	10368	tr|J3QT77|J3QT77_HUMAN	68.31			1.40E+06	1.83E+06	2	2	0	0	1	2	
2768	10369	sp|Q15165|PON2_HUMAN	68.31			1.40E+06	1.83E+06	2	2	0	0	1	2	
2768	10370	tr|A0A0J9YYG4|A0A0J9YYG4_HUMAN	68.31			1.40E+06	1.83E+06	2	2	0	0	1	2	
2768	10371	tr|A0A0J9YXF2|A0A0J9YXF2_HUMAN	68.31			1.40E+06	1.83E+06	2	2	0	0	1	2	
1677	7560	sp|P56381|ATP5E_HUMAN	68.18	3.91E+07	7.78E+07	2.96E+07	3.90E+07	3	3	5	4	2	2	Carbamid
1677	7561	sp|Q5VTU8|AT5EL_HUMAN	68.18	3.91E+07	7.78E+07	2.96E+07	3.90E+07	3	3	5	4	2	2	Carbamid
2438	5973	sp|Q99569|PKP4_HUMAN	68.03	7.57E+05	1.06E+06	0	2.60E+05	5	5	2	2	1	1	
2438	5974	tr|A0A0D9SF60|A0A0D9SF60_HUMAN	68.03	7.57E+05	1.06E+06	0	2.60E+05	5	5	2	2	1	1	
2639	12537	sp|Q5RI15|COX20_HUMAN	67.95	5.43E+05	6.19E+05	3.48E+05		1	1	1	1	1	0	Carbamid
2881	8115	sp|Q96T49|PP16B_HUMAN	67.94	3.54E+04	2.85E+05	2.73E+05		3	3	1	1	1	0	Carbamid
2933	8177	sp|Q12800|TFCP2_HUMAN	67.64					3	2	0	0	1	0	Carbamid
3165	8976	sp|P16591|FER_HUMAN	67.46	4.60E+05				2	2	1	0	0	0	
2661	5607	tr|A0A2U3TZN6|A0A2U3TZN6_HUMAN	67.38	1.28E+06	8.69E+05	2.87E+05		7	7	3	2	2	0	
2661	5608	tr|A0A2U3TZM4|A0A2U3TZM4_HUMAN	67.38	1.28E+06	8.69E+05	2.87E+05		7	7	3	2	2	0	
2661	5609	tr|A0A2U3TZM1|A0A2U3TZM1_HUMAN	67.38	1.28E+06	8.69E+05	2.87E+05		7	7	3	2	2	0	
2661	5610	tr|A0A2R8YE77|A0A2R8YE77_HUMAN	67.38	1.28E+06	8.69E+05	2.87E+05		7	7	3	2	2	0	
2661	5611	sp|O14936|CSKP_HUMAN	67.38	1.28E+06	8.69E+05	2.87E+05		7	7	3	2	2	0	
2848	6706	tr|A0A087WZ88|A0A087WZ88_HUMAN	67.37			1.89E+05		4	1	1	1	2	0	Carbamid
2848	6707	sp|P48443|RXRG_HUMAN	67.37			1.89E+05		4	1	1	1	2	0	Carbamid
2284	6476	sp|Q9UHQ9|NB5R1_HUMAN	67.33	2.72E+05	3.31E+05	3.06E+05		4	4	1	1	1	0	
2284	6570	tr|H7C0R7|H7C0R7_HUMAN	67.33	2.72E+05	3.31E+05	3.06E+05		4	4	1	1	1	0	
2866	8438	sp|Q9P031|TAP26_HUMAN	67.3	4.46E+05	1.32E+06	7.06E+05	1.05E+06	3	3	1	1	1	1	
3135	9067	sp|Q6JQN1|ACD10_HUMAN	67.24				9.30E+05	2	2	0	0	0	1	
2627	11082	sp|Q8NHZ8|CDC26_HUMAN	67.08	2.52E+06	7.84E+06			3	3	2	2	0	0	
3069	8383	sp|P78395|PRAME_HUMAN	67.04				1.60E+05	2	2	0	0	0	1	Carbamid
3069	8651	tr|E7EW99|E7EW99_HUMAN	67.04				1.60E+05	2	2	0	0	0	1	Carbamid
2944	10211	sp|Q52LA3|LIN52_HUMAN	67.01				6.40E+05	2	2	0	0	0	1	
2964	10362	sp|P08047|SP1_HUMAN	67	1.17E+06	1.12E+06			2	2	1	1	0	0	Carbamid
2663	8390	sp|O15344|TRI18_HUMAN	67	3.14E+05	3.45E+05			2	2	2	2	0	0	Carbamid
2663	8410	tr|C9J453|C9J453_HUMAN	67	3.14E+05	3.45E+05			2	2	2	2	0	0	Carbamid
2663	8411	tr|A0A087X255|A0A087X255_HUMAN	67	3.14E+05	3.45E+05			2	2	2	2	0	0	Carbamid
2963	10334	tr|H0Y6D8|H0Y6D8_HUMAN	66.93				3.45E+05	2	2	0	0	0	3	Carbamid
2963	10335	tr|Q32MN6|Q32MN6_HUMAN	66.93				3.45E+05	2	2	0	0	0	3	Carbamid
2963	10336	sp|P20226|TBP_HUMAN	66.93				3.45E+05	2	2	0	0	0	3	Carbamid
2879	7932	sp|Q8NDT2|RB15B_HUMAN	66.8		1.34E+06			3	3	0	3	0	0	Carbamid
2905	8296	sp|P43007|SATT_HUMAN	66.75		4.78E+05		5.66E+05	3	3	0	1	0	1	
3071	8739	sp|P23921|RIR1_HUMAN	66.7	1.83E+05				2	2	1	0	0	0	Carbamid
3071	8911	tr|E9PL69|E9PL69_HUMAN	66.7	1.83E+05				2	2	1	0	0	0	Carbamid
2525	6857	sp|Q96L92|SNX27_HUMAN	66.58	2.93E+05	9.86E+05	2.05E+05		4	4	1	2	1	0	
2744	6575	sp|Q16610|ECM1_HUMAN	66.5	4.17E+05	7.75E+04			5	5	2	1	0	0	Carbamid
3033	8188	sp|Q7L3T8|SYPM_HUMAN	66.32			3.36E+06		3	3	0	0	2	0	Carbamid
2857	8788	sp|Q92575|UBXN4_HUMAN	66.26	4.38E+05	3.74E+05			2	2	1	1	0	0	
2196	6847	tr|H7C1U3|H7C1U3_HUMAN	66.26	7.72E+05	3.91E+05			4	4	3	2	0	0	Deamidat
2196	6853	sp|Q5T0F9|C2D1B_HUMAN	66.26	7.72E+05	3.91E+05			4	4	3	2	0	0	Deamidat
2615	11508	tr|C9JEZ4|C9JEZ4_HUMAN	66.22	6.41E+05	6.84E+05	2.69E+05	3.62E+05	1	1	1	1	1	1	
2615	11509	sp|Q9UKI2|BORG2_HUMAN	66.22	6.41E+05	6.84E+05	2.69E+05	3.62E+05	1	1	1	1	1	1	
2628	11721	tr|A0A2R8YDZ0|A0A2R8YDZ0_HUMAN	66.08	0		0	1.63E+06	1	1	1	0	1	1	
2628	12562	tr|A0A087X215|A0A087X215_HUMAN	66.08	0		0	1.63E+06	1	1	1	0	1	1	
2605	7230	sp|O95870|ABHGA_HUMAN	65.85				1.74E+05	3	3	0	0	0	1	
2605	7233	tr|A0A0G2JJD3|A0A0G2JJD3_HUMAN	65.85				1.74E+05	3	3	0	0	0	1	
2414	7405	sp|Q969Z0|FAKD4_HUMAN	65.71	1.59E+06	8.57E+05			3	3	2	1	0	0	Carbamid
3186	11890	tr|F5H4M0|F5H4M0_HUMAN	65.69	4.30E+05				1	1	1	0	0	0	
3186	11891	sp|Q9H9H4|VP37B_HUMAN	65.69	4.30E+05				1	1	1	0	0	0	
2305	10235	sp|Q8NHG7|SVIP_HUMAN	65.68	3.40E+06	2.61E+06	4.62E+06	4.44E+06	2	2	1	1	2	1	
2709	7617	tr|Q5T7U1|Q5T7U1_HUMAN	65.66			9.99E+05	4.76E+05	3	3	0	0	2	1	Carbamid
2709	7620	sp|Q9Y5Q8|TF3C5_HUMAN	65.66			9.99E+05	4.76E+05	3	3	0	0	2	1	Carbamid
2136	7097	sp|Q9HC21|TPC_HUMAN	65.52	4.69E+05	9.54E+05		7.01E+05	3	3	1	2	0	2	
2935	8478	sp|Q92688|AN32B_HUMAN	65.5	2.15E+05	7.04E+05			2	2	1	2	0	0	Carbamid
1995	6778	sp|Q03113|GNA12_HUMAN	65.42				1.89E+05	4	1	2	2	2	3	
3098	9241	sp|Q92621|NU205_HUMAN	65.39		4.43E+04		1.09E+04	2	2	0	1	0	1	Acetylatic
2335	6665	tr|H0YHS6|H0YHS6_HUMAN	65.36			6.09E+05	1.49E+06	4	4	0	0	1	4	
2335	6666	sp|Q9Y2Z4|SYYM_HUMAN	65.36			6.09E+05	1.49E+06	4	4	0	0	1	4	
3321	8502	sp|Q13177|PAK2_HUMAN	65.35	4.67E+05				3	3	2	0	0	0	Carbamid
3321	9513	tr|H7C1X3|H7C1X3_HUMAN	65.35	2.03E+05				2	2	1	0	0	0	Carbamid
3321	9659	tr|E9PM17|E9PM17_HUMAN	65.35					2	2	0	0	0	0	
3321	9660	tr|B3KNX7|B3KNX7_HUMAN	65.35					2	2	0	0	0	0	
3321	9661	sp|Q13153|PAK1_HUMAN	65.35					2	2	0	0	0	0	
3321	10703	tr|A0A087X294|A0A087X294_HUMAN	65.35					1	1	0	0	0	0	
3321	10704	sp|O75914|PAK3_HUMAN	65.35					1	1	0	0	0	0	
3321	12211	tr|H0YCG5|H0YCG5_HUMAN	65.35					1	1	0	0	0	0	
2104	10296	sp|O14519|CDKA1_HUMAN	65.3	5.52E+06	4.39E+06	3.24E+06	2.10E+06	2	2	2	2	1	2	Carbamid
2104	10297	sp|O75956|CDKA2_HUMAN	65.3	5.52E+06	4.39E+06	3.24E+06	2.10E+06	2	2	2	2	1	2	Carbamid
2827	6951	sp|Q96KA5|CLP1L_HUMAN	65.29		1.13E+05	2.99E+05		3	3	0	1	1	0	
2827	7410	tr|G5E9Z2|G5E9Z2_HUMAN	65.29		1.13E+05	2.99E+05		3	3	0	1	1	0	
2341	8049	sp|Q96JC1|VPS39_HUMAN	65.26	1.37E+05	4.74E+04	5.41E+04	5.57E+04	3	3	2	1	1	1	Carbamid
3131	8570	sp|O94763|RMP_HUMAN	65.2	9.89E+04				2	2	1	0	0	0	
3008	8585	sp|Q7Z2T5|TRM1L_HUMAN	65.02				2.93E+05	2	2	0	0	0	1	
2969	12573	sp|Q9H0W8|SMG9_HUMAN	64.93					1	1	0	0	0	0	
3436	9524	sp|O75365|TP4A3_HUMAN	64.69					1	1	0	0	0	0	
2786	7566	sp|P49454|CENPF_HUMAN	64.58	7.27E+04			6.17E+05	4	4	2	0	0	1	Acetylatic
2666	6671	tr|B4E321|B4E321_HUMAN	64.55	1.10E+06	1.77E+05	1.10E+06	4.98E+04	4	4	3	2	1	1	
2666	6674	sp|Q13438|OS9_HUMAN	64.55	1.10E+06	1.77E+05	1.10E+06	4.98E+04	4	4	3	2	1	1	
2577	8954	sp|Q9NZ45|CISD1_HUMAN	64.52	1.52E+06	5.27E+05	8.92E+05		2	2	2	1	1	0	
2731	8095	sp|P55011|S12A2_HUMAN	64.43	5.60E+05	2.96E+05			3	3	2	1	0	0	
2584	6866	sp|Q9UM13|APC10_HUMAN	64.41	1.33E+06		1.00E+06		4	4	2	0	1	0	

3103	8281	tr|F8VUB4|F8VUB4_HUMAN	64.36		1.81E+05			2	2	0	1	0	0	
3103	8282	tr|F8VV52|F8VV52_HUMAN	64.36		1.81E+05			2	2	0	1	0	0	
3103	8283	sp|Q9NZN8|CNOT2_HUMAN	64.36		1.81E+05			2	2	0	1	0	0	
3103	8908	tr|F8VP97|F8VP97_HUMAN	64.36		1.81E+05			2	2	0	1	0	0	
3341	9110	tr|G5E9C8|G5E9C8_HUMAN	64.32					1	1	0	0	0	0	
3341	9111	sp|Q07889|SOS1_HUMAN	64.32					1	1	0	0	0	0	
2862	6673	sp|Q9Y375|CIA30_HUMAN	64.14	2.07E+06				4	4	2	0	0	0	Carbamid
2862	6740	tr|H0YL22|H0YL22_HUMAN	64.14	2.07E+06				4	4	2	0	0	0	Carbamid
2862	6970	tr|H0YNN4|H0YNN4_HUMAN	64.14	2.07E+06				4	4	2	0	0	0	Carbamid
2862	6971	tr|H0YNB7|H0YNB7_HUMAN	64.14	2.07E+06				4	4	2	0	0	0	Carbamid
2454	8479	sp|Q9NXZ2|DDX43_HUMAN	64.1		2.19E+05	1.15E+06	3.58E+05	2	2	0	1	2	1	
2667	6974	tr|C9J4M6|C9J4M6_HUMAN	64.09	4.37E+05	1.99E+06	4.24E+05		5	5	2	1	2	0	
2667	6976	tr|A0A1W2PPT5|A0A1W2PPT5_HUMAN	64.09	4.37E+05	1.99E+06	4.24E+05		5	5	2	1	2	0	
2667	6978	tr|C9J2Y9|C9J2Y9_HUMAN	64.09	4.37E+05	1.99E+06	4.24E+05		5	5	2	1	2	0	
2667	6979	sp|P30876|RPB2_HUMAN	64.09	4.37E+05	1.99E+06	4.24E+05		5	5	2	1	2	0	
2491	9612	tr|C9JYM0|C9JYM0_HUMAN	64.07	6.05E+05	6.97E+05	0		2	2	1	1	1	0	Carbamid
2491	9613	sp|O75817|POP7_HUMAN	64.07	6.05E+05	6.97E+05	0		2	2	1	1	1	0	Carbamid
2589	8064	sp|Q8N392|RHG18_HUMAN	64.04		2.12E+05	1.76E+05	8.60E+04	3	3	0	1	1	1	
2583	6455	sp|Q9HCD5|NCOA5_HUMAN	64	4.77E+05	1.42E+06			4	4	1	4	0	0	Carbamid
2934	8295	sp|P20618|PSB1_HUMAN	63.97	3.01E+05	4.55E+05			3	3	1	2	0	0	
2260	6624	tr|C9JEX3|C9JEX3_HUMAN	63.82	3.23E+05	5.74E+05		3.21E+05	4	2	4	3	0	1	
2260	6626	sp|Q13614|MTMR2_HUMAN	63.82	3.23E+05	5.74E+05		3.21E+05	4	2	4	3	0	1	
3534	10595	sp|Q8WVV4|POF1B_HUMAN	63.75					1	1	0	0	0	0	
2749	8374	tr|F2Z2Y6|F2Z2Y6_HUMAN	63.73	7.72E+05	6.46E+05			3	3	1	1	0	0	
2749	8376	tr|C9JNV3|C9JNV3_HUMAN	63.73	7.72E+05	6.46E+05			3	3	1	1	0	0	
2749	8377	sp|O95777|LSM8_HUMAN	63.73	7.72E+05	6.46E+05			3	3	1	1	0	0	
2908	8719	sp|O95218|ZRAB2_HUMAN	63.69	1.76E+06	2.54E+06			2	2	1	2	0	0	Carbamid
2603	7133	tr|J3KMW5|J3KMW5_HUMAN	63.67	4.75E+04			3.55E+05	4	4	1	0	0	1	Carbamid
2603	7134	sp|Q9BQD7|F173A_HUMAN	63.67	4.75E+04			3.55E+05	4	4	1	0	0	1	Carbamid
2502	12609	sp|Q8WVI0|SMIM4_HUMAN	63.62	9.17E+05	3.09E+06	3.53E+06	5.73E+06	1	1	1	1	1	1	
2920	10255	tr|K7EQB1|K7EQB1_HUMAN	63.6			1.07E+06		3	3	0	0	1	0	Acetylatic
2920	10256	sp|Q9UNK0|STX8_HUMAN	63.6					2	2	0	0	0	0	
3019	8417	sp|Q14694|UBP10_HUMAN	63.6		2.06E+05	8.01E+05	3.32E+05	4	4	0	1	1	1	Carbamid
3019	10899	tr|H3BQC6|H3BQC6_HUMAN	63.6		2.06E+05		3.32E+05	2	2	0	1	0	1	Carbamid
2163	7874	sp|Q96LJ7|DHRS1_HUMAN	63.6		4.40E+05	2.89E+05		3	2	1	3	2	1	Carbamid
2301	10515	tr|C9J719|C9J719_HUMAN	63.59	3.41E+06	3.73E+06		2.12E+06	2	2	2	2	0	1	Acetylatic
2301	10516	tr|C9JJ78|C9JJ78_HUMAN	63.59	3.41E+06	3.73E+06		2.12E+06	2	2	2	2	0	1	Acetylatic
2301	10517	sp|Q15125|EBP_HUMAN	63.59	3.41E+06	3.73E+06		2.12E+06	2	2	2	2	0	1	Acetylatic
2693	7152	sp|O14733|MP2K7_HUMAN	63.55					3	3	0	0	0	0	Carbamid
3041	9508	tr|A0A087WW76|A0A087WW76_HUMAN	63.42	3.09E+05	2.48E+05			1	1	1	1	0	0	
3041	9510	sp|Q5T5U3|RHG21_HUMAN	63.42	3.09E+05	2.48E+05			1	1	1	1	0	0	
2821	9112	sp|Q6UB98|ANR12_HUMAN	63.37	0	0		0	1	1	1	1	0	1	
2821	12593	tr|F5GYX2|F5GYX2_HUMAN	63.37	0	0		0	1	1	1	1	0	1	
3105	8839	sp|Q9NY61|AATF_HUMAN	63.37	2.58E+05		8.76E+04		2	2	1	0	1	0	Deamidat
3139	9359	sp|P20585|MSH3_HUMAN	63.37					2	2	0	0	0	0	
2822	7715	sp|Q9Y2H6|FND3A_HUMAN	63.19	8.37E+04		5.10E+05	1.04E+05	4	4	1	0	1	1	
2691	6415	tr|C9JB30|C9JB30_HUMAN	63.14	5.81E+06			2.85E+05	5	3	2	1	0	1	Carbamid
2568	7191	sp|P06702|S10A9_HUMAN	63.09					4	4	0	0	0	0	
2820	9031	sp|Q16204|CCDC6_HUMAN	63.02	3.43E+05	1.92E+05	1.11E+05		2	2	1	1	1	0	
3167	9033	sp|Q8TED9|AF1L1_HUMAN	62.93	4.09E+04		1.61E+05		2	2	1	0	1	0	Carbamid
2544	7486	sp|Q8WYJ6|SEPT1_HUMAN	62.9					4	1	0	2	0	0	Carbamid
2544	7507	tr|J3KNL2|J3KNL2_HUMAN	62.9					4	1	0	2	0	0	Carbamid
3108	9132	sp|P13489|RINI_HUMAN	62.87					2	2	0	0	0	0	Carbamid
3210	10792	sp|Q9H009|NACA2_HUMAN	62.82	9.14E+06				2	1	1	0	0	0	Deamidat
2748	7888	sp|Q9NXG2|THUM1_HUMAN	62.73	6.33E+04		1.31E+05	9.48E+05	3	3	1	0	1	1	Carbamid
2971	12649	tr|H3BUD2|H3BUD2_HUMAN	62.61		2.05E+05		0	1	1	0	1	0	1	Acetylatic
2971	12671	tr|H3BS05|H3BS05_HUMAN	62.61		2.05E+05		0	1	1	0	1	0	1	Acetylatic
2971	12716	tr|H3BR94|H3BR94_HUMAN	62.61		2.05E+05		0	1	1	0	1	0	1	Acetylatic
2971	12730	sp|Q9BTE1|DCTN5_HUMAN	62.61		2.05E+05		0	1	1	0	1	0	1	Acetylatic
2275	8821	sp|P51665|PSMD7_HUMAN	62.57	8.04E+05	1.12E+06	1.37E+06	5.89E+05	2	2	1	1	2	1	
2275	8935	tr|H3BNT7|H3BNT7_HUMAN	62.57	8.04E+05	1.12E+06	1.37E+06	5.89E+05	2	2	1	1	2	1	
2275	8936	tr|H3BTM8|H3BTM8_HUMAN	62.57	8.04E+05	1.12E+06	1.37E+06	5.89E+05	2	2	1	1	2	1	
2635	10264	sp|Q9BW60|ELOV1_HUMAN	62.53		4.34E+05		1.79E+06	2	2	0	2	0	2	Acetylatic
3034	8321	sp|Q9UDT6|CLIP2_HUMAN	62.47		1.46E+06	1.58E+05		3	2	1	1	1	0	Deamidat
2610	8663	sp|Q96EY8|MMAB_HUMAN	62.41	0			3.93E+05	2	2	1	0	0	1	
3011	8872	sp|P53814|SMTN_HUMAN	62.18	1.94E+05		4.73E+05		2	2	1	0	1	0	Acetylatic
3126	8403	tr|A0A0C4DFM1|A0A0C4DFM1_HUMAN	62.13				1.97E+05	2	2	0	0	0	1	Carbamid
3126	8404	sp|Q92544|TM9S4_HUMAN	62.13				1.97E+05	2	2	0	0	0	1	Carbamid
2763	8127	sp|Q9UQC2|GAB2_HUMAN	61.96	8.07E+05	1.79E+06	1.29E+06		3	3	1	2	1	0	Carbamid
2763	8196	tr|E9PJE2|E9PJE2_HUMAN	61.96	8.07E+05	1.79E+06	1.29E+06		3	3	1	2	1	0	Carbamid
2922	10463	sp|Q6UW78|UQCC3_HUMAN	61.94	1.13E+06	7.46E+05	6.75E+05		2	2	1	2	1	0	Deamidat
2970	12605	sp|Q7Z5G4|GOGA7_HUMAN	61.87	4.59E+04				1	1	1	0	0	0	
2607	7793	tr|A0A0C4DGT8|A0A0C4DGT8_HUMAN	61.85	7.08E+04		6.50E+05		3	3	1	0	3	0	Carbamid
2607	7794	tr|A0A087WWP8|A0A087WWP8_HUMAN	61.85	7.08E+04		6.50E+05		3	3	1	0	3	0	Carbamid
2607	7795	sp|Q5VWQ0|RSBN1_HUMAN	61.85	7.08E+04		6.50E+05		3	3	1	0	3	0	Carbamid
3127	8501	sp|Q96MX6|WDR92_HUMAN	61.69				2.58E+05	3	3	0	0	0	1	Carbamid
3127	10435	tr|Q8ND98|Q8ND98_HUMAN	61.69				2.58E+05	2	2	0	0	0	1	Carbamid
2835	8753	sp|P10646|TFPI1_HUMAN	61.66	9.07E+05	8.08E+05	9.34E+05	8.96E+05	1	1	1	1	1	1	Carbamid
2835	12637	tr|C9JKV3|C9JKV3_HUMAN	61.66	9.07E+05	8.08E+05	9.34E+05	8.96E+05	1	1	1	1	1	1	Carbamid
2122	8530	sp|P03928|ATP8_HUMAN	61.64	1.24E+07	6.69E+06	2.72E+06	2.19E+07	2	2	2	1	1	2	Carbamid
3204	10454	tr|G3V2W9|G3V2W9_HUMAN	61.63			1.06E+06		2	2	0	0	1	0	
3204	10455	tr|A0A087X060|A0A087X060_HUMAN	61.63			1.06E+06		2	2	0	0	1	0	
3204	10456	sp|Q56P03|EAPP_HUMAN	61.63			1.06E+06		2	2	0	0	1	0	
2794	7913	tr|A0A087WV90|A0A087WV90_HUMAN	61.62	3.43E+05	2.38E+05	8.17E+05		3	3	1	2	1	0	Carbamid
2794	7914	tr|E9PDN5|E9PDN5_HUMAN	61.62	3.43E+05	2.38E+05	8.17E+05		3	3	1	2	1	0	Carbamid
2794	7915	tr|A0A087WTU7|A0A087WTU7_HUMAN	61.62	3.43E+05	2.38E+05	8.17E+05		3	3	1	2	1	0	Carbamid
2794	7916	sp|P11532|DMD_HUMAN	61.62	3.43E+05	2.38E+05	8.17E+05		3	3	1	2	1	0	Carbamid
2794	7917	tr|A0A075B6G3|A0A075B6G3_HUMAN	61.62	3.43E+05	2.38E+05	8.17E+05		3	3	1	2	1	0	Carbamid
2794	7982	tr|E7EQR9|E7EQR9_HUMAN	61.62	3.43E+05	2.38E+05	8.17E+05		3	3	1	2	1	0	Carbamid
2794	7986	tr|F5GZY3|F5GZY3_HUMAN	61.62	3.43E+05	2.38E+05	8.17E+05		3	3	1	2	1	0	Carbamid
2794	7987	tr|E7EQS5|E7EQS5_HUMAN	61.62	3.43E+05	2.38E+05	8.17E+05		3	3	1	2	1	0	Carbamid
2794	7988	tr|F8VX32|F8VX32_HUMAN	61.62	3.43E+05	2.38E+05	8.17E+05		3	3	1	2	1	0	Carbamid
2794	7989	tr|E7ESB2|E7ESB2_HUMAN	61.62	3.43E+05	2.38E+05	8.17E+05		3	3	1	2	1	0	Carbamid
2794	7992	tr|H0Y304|H0Y304_HUMAN	61.62	3.43E+05	2.38E+05	8.17E+05		3	3	1	2	1	0	Carbamid
3066	8166	sp|P24530|EDNRB_HUMAN	61.59	1.40E+05				3	3	1	0	0	0	Carbamid
3066	8191	tr|A0A2R8Y748|A0A2R8Y748_HUMAN	61.59	1.40E+05				3	3	1	0	0	0	Carbamid
2925	11827	sp|Q5VWP3|MLIP_HUMAN	61.52	9.80E+05	6.86E+05		6.08E+05	1	1	1	1	0	1	
2925	12627	tr|E9PFJ4|E9PFJ4_HUMAN	61.52	9.80E+05	6.86E+05		6.08E+05	1	1	1	1	0	1	
3214	12789	sp|O14613|BORG1_HUMAN	61.46		1.29E+04			2	2	0	1	0	0	
2767	10278	tr|A6NGW1|A6NGW1_HUMAN	61.39	8.34E+05		0		2	2	1	0	1	0	
2767	10279	sp|O95070|YIF1A_HUMAN	61.39	8.34E+05		0		2	2	1	0	1	0	
3065	8070	sp|Q9P0J1|PDP1_HUMAN	61.3		2.81E+05		3.89E+05	3	3	0	1	0	1	
2501	10726	sp|Q99471|PFD5_HUMAN	61.16	4.82E+05	6.41E+05	3.17E+05	3.70E+06	2	2	1	1	1	2	Deamidat
2420	6266	sp|Q13188|STK3_HUMAN	60.99	8.23E+05	2.64E+05		3.23E+05	5	4	3	4	2	2	
3496	10788	sp|Q9NTX5|ECHD1_HUMAN	60.94				1.16E+05	1	1	0	0	0	1	
2778	10470	sp|Q8IZ52|CHSS2_HUMAN	60.84				4.64E+05	2	2	0	0	0	2	
3091	8717	sp|Q8N954|GPT11_HUMAN	60.82	6.88E+05		7.77E+05	3.93E+05	3	3	2	0	2	1	
2770	11646	sp|Q9H479|FN3K_HUMAN	60.78	1.91E+05	2.25E+05		2.01E+05	1	1	1	1	0	1	
3090	8670	tr|C9J4K5|C9J4K5_HUMAN	60.78	1.86E+05	1.88E+05			2	1	1	1	1	0	
3090	8671	tr|C9JSP0|C9JSP0_HUMAN	60.78	1.86E+05	1.88E+05			2	1	1	1	1	0	
3090	8672	tr|F8W8A5|F8W8A5_HUMAN	60.78	1.86E+05	1.88E+05			2	1	1	1	1	0	
3090	8673	sp|Q9NYP3|DONS_HUMAN	60.78	1.86E+05	1.88E+05			2	1	1	1	1	0	
3090	8732	tr|H7C1C1|H7C1C1_HUMAN	60.78	1.86E+05	1.88E+05			2	1	1	1	1	0	
3090	9424	tr|F8WD19|F8WD19_HUMAN	60.78	1.86E+05	1.88E+05			2	1	1	1	1	0	
2697	8204	sp|Q6UWU2|GLB1L_HUMAN	60.62		6.63E+05		1.22E+06	3	3	0	1	0	2	
2434	9502	sp|Q92629|SGCD_HUMAN	60.53	1.74E+05	1.61E+05	3.82E+05	2.70E+05	1	1	1	1	1	1	

2595	10506	sp|P53803|RPAB4_HUMAN	60.48	5.33E+06	2.60E+06	2.64E+06	3.47E+06	2	2	2	1	1	1	Carbamid
2780	10823	tr|E9PP21|E9PP21_HUMAN	60.36	1.51E+06		5.54E+05		2	2	2	0	1	0	Carbamid
3172	10307	sp|Q9UKU7|ACAD8_HUMAN	60.24					2	1	0	1	0	0	Carbamid
2368	10293	sp|Q9Y2S6|TMA7_HUMAN	60.19	2.73E+07	6.10E+06	3.32E+06	6.00E+06	2	2	4	1	1	2	
2856	8606	tr|H0YJG7|H0YJG7_HUMAN	60.13		2.01E+05	6.28E+05		2	2	0	1	1	0	
2856	8607	sp|O95433|AHSA1_HUMAN	60.13		2.01E+05	6.28E+05		2	2	0	1	1	0	
2856	10604	tr|H0YJ63|H0YJ63_HUMAN	60.13		2.01E+05	6.28E+05		2	2	0	1	1	0	
2856	10605	tr|G3V438|G3V438_HUMAN	60.13		2.01E+05	6.28E+05		2	2	0	1	1	0	
2427	6651	sp|Q05823|RN5A_HUMAN	60.1	0	2.06E+05		6.59E+05	3	3	1	2	0	4	
2533	8213	sp|Q8WYP5|ELYS_HUMAN	60.05	1.37E+06	5.19E+06		6.62E+06	3	3	2	3	0	2	
3140	9486	tr|A0A087WUN5|A0A087WUN5_HUMAN	60.04	5.72E+05				1	1	1	0	0	0	
3140	9487	sp|Q96DE5|APC16_HUMAN	60.04	5.72E+05				1	1	1	0	0	0	
2620	9024	sp|O75110|ATP9A_HUMAN	59.97			5.92E+05	3.38E+05	2	2	0	0	1	1	
2695	7752	sp|O43813|LANC1_HUMAN	59.81	4.17E+05	8.98E+05	1.28E+06	8.70E+05	4	4	1	2	2	2	Carbamid
2705	7196	tr|B8ZZX2|B8ZZX2_HUMAN	59.77	1.88E+05	4.48E+05			3	3	1	1	0	0	
2705	7197	tr|B8ZZV9|B8ZZV9_HUMAN	59.77	1.88E+05	4.48E+05			3	3	1	1	0	0	
2705	7198	sp|Q9H8M1|CQ10B_HUMAN	59.77	1.88E+05	4.48E+05			3	3	1	1	0	0	
3035	8375	sp|Q9BY12|SCAPE_HUMAN	59.71	1.90E+05		3.00E+05		3	3	2	0	1	0	Carbamid
2921	10300	sp|Q9Y5J7|TIM9_HUMAN	59.64		3.47E+05		5.15E+05	2	2	0	1	0	1	Carbamid
2142	8036	sp|Q07002|CDK18_HUMAN	59.58			1.62E+06		4	2	2	2	3	1	
3136	9221	tr|Q5TB53|Q5TB53_HUMAN	59.5	1.35E+05	3.96E+05			2	2	1	1	0	0	Carbamid
3136	9222	sp|Q9HD45|TM9S3_HUMAN	59.5	1.35E+05	3.96E+05			2	2	1	1	0	0	Carbamid
2699	8974	sp|Q96GW9|SYMM_HUMAN	59.49	1.27E+05		2.31E+05		2	2	1	0	1	0	
2919	9549	sp|Q7Z699|SPRE1_HUMAN	59.48	1.73E+05		0		2	2	1	0	1	0	Carbamid
3142	9857	tr|H0YJM8|H0YJM8_HUMAN	59.46			5.04E+05		1	1	0	0	1	0	
3142	9858	sp|P28074|PSB5_HUMAN	59.46			5.04E+05		1	1	0	0	1	0	
3032	8039	sp|Q12996|CSTF3_HUMAN	59.46		1.69E+05	1.71E+05		4	4	0	1	1	0	
3032	8040	tr|E9PLP8|E9PLP8_HUMAN	59.46		1.69E+05	1.71E+05		4	4	0	1	1	0	
3515	11907	tr|H0YJH0|H0YJH0_HUMAN	59.42	6.94E+06	4.47E+06			2	2	2	1	0	0	
3515	11908	sp|Q9H4B6|SAV1_HUMAN	59.42	6.94E+06	4.47E+06			2	2	2	1	0	0	
2710	7688	sp|Q8IYI6|EXOC8_HUMAN	59.4	8.28E+04	8.48E+04			3	3	1	1	0	0	Carbamid
3201	10353	tr|K7ESP4|K7ESP4_HUMAN	59.3	3.28E+05				2	2	2	0	0	0	
3201	10354	sp|Q8WVC6|DCAKD_HUMAN	59.3	3.28E+05				2	2	2	0	0	0	
2538	7935	sp|Q6KC79|NIPBL_HUMAN	59.26	4.75E+06	3.23E+06	2.32E+06	2.53E+06	3	3	3	1	1	1	Carbamid
2546	8267	tr|A0A087X2B5|A0A087X2B5_HUMAN	59.11	1.01E+06	1.05E+06		7.70E+05	3	3	1	1	0	1	
2546	8268	sp|P35613|BASI_HUMAN	59.11	1.01E+06	1.05E+06		7.70E+05	3	3	1	1	0	1	
2546	8301	tr|A0A087WUV8|A0A087WUV8_HUMAN	59.11	1.01E+06	1.05E+06		7.70E+05	3	3	1	1	0	1	
2807	7596	sp|Q9UPT8|ZC3H4_HUMAN	59.11	1.79E+06	0	5.47E+06		4	4	2	1	1	0	
3050	8286	sp|P27986|P85A_HUMAN	59.08	3.35E+05				4	2	2	0	1	0	
3050	8475	sp|Q92569|P55G_HUMAN	59.08					3	1	1	0	1	0	
3050	8476	tr|F6TDL0|F6TDL0_HUMAN	59.08					3	1	1	0	1	0	
2732	8120	tr|K7ERP4|K7ERP4_HUMAN	59.04		1.75E+06		4.34E+05	3	3	0	2	0	1	Carbamid
2732	8121	tr|K7EKX7|K7EKX7_HUMAN	59.04		1.75E+06		4.34E+05	3	3	0	2	0	1	Carbamid
2732	8122	tr|K7ENB4|K7ENB4_HUMAN	59.04		1.75E+06		4.34E+05	3	3	0	2	0	1	Carbamid
2732	8123	tr|A0A087X2I2|A0A087X2I2_HUMAN	59.04		1.75E+06		4.34E+05	3	3	0	2	0	1	Carbamid
2732	8124	sp|P36969|GPX4_HUMAN	59.04		1.75E+06		4.34E+05	3	3	0	2	0	1	Carbamid
2732	8125	tr|A0A087WT12|A0A087WT12_HUMAN	59.04		1.75E+06		4.34E+05	3	3	0	2	0	1	Carbamid
2732	8126	tr|K7EJ20|K7EJ20_HUMAN	59.04		1.75E+06		4.34E+05	3	3	0	2	0	1	Carbamid
3448	10436	sp|Q13325|IFIT5_HUMAN	58.99			6.89E+05		1	1	0	0	1	0	Carbamid
2834	8373	tr|J3KNG6|J3KNG6_HUMAN	58.96	1.69E+06	1.38E+06			2	2	1	1	0	0	
2858	8805	tr|A2RQR6|A2RQR6_HUMAN	58.96	1.20E+06				2	2	2	0	0	0	
2858	8806	sp|Q96EI5|TCAL4_HUMAN	58.96	1.20E+06				2	2	2	0	0	0	
2858	9437	sp|Q9H3H9|TCAL2_HUMAN	58.96	1.20E+06				2	2	2	0	0	0	
2954	11816	tr|K7EKX0|K7EKX0_HUMAN	58.86		1.47E+05		1.48E+05	1	1	0	1	0	1	
2954	11817	sp|Q15836|VAMP3_HUMAN	58.86		1.47E+05		1.48E+05	1	1	0	1	0	1	
2954	12682	tr|K7ENK9|K7ENK9_HUMAN	58.86		1.47E+05		1.48E+05	1	1	0	1	0	1	
2954	12683	tr|J3QRU4|J3QRU4_HUMAN	58.86		1.47E+05		1.48E+05	1	1	0	1	0	1	
2954	12684	sp|P63027|VAMP2_HUMAN	58.86		1.47E+05		1.48E+05	1	1	0	1	0	1	
2954	12685	tr|F8WCA0|F8WCA0_HUMAN	58.86		1.47E+05		1.48E+05	1	1	0	1	0	1	
2954	12686	tr|L7N2F9|L7N2F9_HUMAN	58.86		1.47E+05		1.48E+05	1	1	0	1	0	1	
2657	6894	sp|P20700|LMNB1_HUMAN	58.75				8.19E+04	5	4	1	1	0	1	
3610	12039	sp|P49720|PSB3_HUMAN	58.71		8.22E+04			1	1	0	1	0	0	
2864	7767	sp|Q96HP0|DOCK6_HUMAN	58.71	1.50E+04				4	1	1	3	0	0	
3149	11838	sp|Q8ND76|CCNY_HUMAN	58.7	1.08E+05				1	1	1	0	0	0	
2602	7028	sp|Q9GZT9|EGLN1_HUMAN	58.68	1.99E+06				5	5	5	0	0	0	Carbamid
3209	10553	sp|Q9NVH6|TMLH_HUMAN	58.68					2	2	0	0	0	0	
2606	7607	sp|Q93050|VPP1_HUMAN	58.6			1.38E+05	1.43E+05	3	3	0	0	1	1	
2606	7864	tr|K7EM24|K7EM24_HUMAN	58.6			1.38E+05	1.43E+05	3	3	0	0	1	1	
3162	8440	sp|P78549|NTH_HUMAN	58.59		1.51E+05			3	3	0	1	0	0	
3402	9641	tr|E7EX90|E7EX90_HUMAN	58.54			3.24E+04		1	1	0	0	1	0	
3402	9642	sp|Q14203|DCTN1_HUMAN	58.54			3.24E+04		1	1	0	0	1	0	
3402	10919	tr|Q6AWB1|Q6AWB1_HUMAN	58.54			3.24E+04		1	1	0	0	1	0	
2597	10657	tr|A6NCC9|A6NCC9_HUMAN	58.48	2.69E+05	3.06E+06	1.91E+06	1.59E+06	2	2	1	2	2	2	Carbamid
2597	10658	tr|E9PME3|E9PME3_HUMAN	58.48	2.69E+05	3.06E+06	1.91E+06	1.59E+06	2	2	1	2	2	2	Carbamid
2597	10659	tr|C9JHT9|C9JHT9_HUMAN	58.48	2.69E+05	3.06E+06	1.91E+06	1.59E+06	2	2	1	2	2	2	Carbamid
2597	10660	tr|C9JJV1|C9JJV1_HUMAN	58.48	2.69E+05	3.06E+06	1.91E+06	1.59E+06	2	2	1	2	2	2	Carbamid
2597	10661	sp|Q9Y5U2|TSSC4_HUMAN	58.48	2.69E+05	3.06E+06	1.91E+06	1.59E+06	2	2	1	2	2	2	Carbamid
3192	12222	sp|Q8TB37|NUBPL_HUMAN	58.38	4.14E+05			2.20E+05	1	1	1	0	0	1	
3192	12678	tr|H0YHR7|H0YHR7_HUMAN	58.38	4.14E+05			2.20E+05	1	1	1	0	0	1	
3192	12679	tr|F8W061|F8W061_HUMAN	58.38	4.14E+05			2.20E+05	1	1	1	0	0	1	
2892	9277	tr|H7BZK2|H7BZK2_HUMAN	58.38					2	2	0	0	0	0	Carbamid
2892	9278	sp|Q9Y5S1|TRPV2_HUMAN	58.38					2	2	0	0	0	0	Carbamid
2931	7438	sp|Q92570|NR4A3_HUMAN	58.38					3	3	0	0	0	0	
2932	8145	sp|Q92989|CLP1_HUMAN	58.33	5.29E+05				3	3	3	0	0	0	
2932	8146	tr|E9PL17|E9PL17_HUMAN	58.33	5.29E+05				3	3	3	0	0	0	
3055	8731	tr|F2Z2B9|F2Z2B9_HUMAN	58.27					2	2	0	0	0	0	
3055	8738	sp|Q9BSJ2|GCP2_HUMAN	58.27					2	2	0	0	0	0	
3055	9153	tr|B3KTU7|B3KTU7_HUMAN	58.27					2	2	0	0	0	0	
3658	12680	sp|P14927|QCR7_HUMAN	58.23	5.98E+05				1	1	1	0	0	0	
2718	8991	tr|G8JLQ3|G8JLQ3_HUMAN	58.11	1.21E+06				2	2	1	0	0	0	
2718	8999	sp|P78537|BL1S1_HUMAN	58.11	1.21E+06				2	2	1	0	0	0	
2543	6595	tr|E9PLT0|E9PLT0_HUMAN	57.97	1.77E+05		7.41E+05	8.89E+05	4	4	1	0	1	2	Carbamid
3432	9319	tr|A6NHV6|A6NHV6_HUMAN	57.94		6.43E+05	5.22E+05		2	2	0	1	1	0	Carbamid
3432	9320	sp|Q99640|PMYT1_HUMAN	57.94		6.43E+05	5.22E+05		2	2	0	1	1	0	Carbamid
3432	9321	tr|B4DZM6|B4DZM6_HUMAN	57.94		6.43E+05	5.22E+05		2	2	0	1	1	0	Carbamid
2496	12706	tr|B8ZZV5|B8ZZV5_HUMAN	57.92		1.21E+06	2.22E+06	2.56E+06	1	1	0	1	1	1	
2496	12714	sp|Q8TCC3|RM30_HUMAN	57.92		1.21E+06	2.22E+06	2.56E+06	1	1	0	1	1	1	
3543	11106	sp|Q14137|BOP1_HUMAN	57.88			1.79E+05	2.33E+05	2	2	0	0	1	1	
3027	8253	tr|A0A087WVF7|A0A087WVF7_HUMAN	57.88	9.32E+05				3	3	2	0	0	0	
3027	8254	sp|Q9NZM3|ITSN2_HUMAN	57.88	9.32E+05				3	3	2	0	0	0	
2395	7828	sp|Q9H061|T126A_HUMAN	57.84	3.37E+06	1.98E+06			3	3	3	2	0	0	
2733	8178	sp|Q9BRK5|CAB45_HUMAN	57.82		7.75E+05	3.84E+05		3	3	0	1	1	0	
2873	6902	tr|A0A0A0MTN3|A0A0A0MTN3_HUMAN	57.71	3.80E+05	6.39E+04			4	4	1	1	0	0	
2873	6903	sp|P21266|GSTM3_HUMAN	57.71	3.80E+05	6.39E+04			4	4	1	1	0	0	
2527	7849	sp|O75844|FACE1_HUMAN	57.57	8.39E+05	1.16E+06	4.39E+05		4	4	1	2	2	0	
2590	8666	tr|B8ZZY2|B8ZZY2_HUMAN	57.4	2.22E+06	4.13E+05	2.13E+05	1.69E+05	2	2	2	1	1	1	
2590	8667	sp|P52594|AGFG1_HUMAN	57.4	2.22E+06	4.13E+05	2.13E+05	1.69E+05	2	2	2	1	1	1	
2467	10781	sp|Q8WWC4|MAIP1_HUMAN	57.37	5.95E+05	6.79E+05	1.05E+06	4.83E+05	1	1	1	1	1	1	
2467	11110	tr|H7C0V0|H7C0V0_HUMAN	57.37	5.95E+05	6.79E+05	1.05E+06	4.83E+05	1	1	1	1	1	1	
3660	12697	sp|Q53RT3|APRV1_HUMAN	57.23					1	1	0	0	0	0	
3659	12690	tr|H7C131|H7C131_HUMAN	57.17					1	1	0	0	0	0	
3659	12691	tr|C9JDE9|C9JDE9_HUMAN	57.17					1	1	0	0	0	0	
3659	12692	sp|P09110|THIK_HUMAN	57.17					1	1	0	0	0	0	
3191	12160	tr|J3KT86|J3KT86_HUMAN	57.13			1.67E+06		1	1	0	0	1	0	
3191	12161	sp|Q96GD4|AURKB_HUMAN	57.13			1.67E+06		1	1	0	0	1	0	
3191	12723	tr|J3KRJ2|J3KRJ2_HUMAN	57.13			1.67E+06		1	1	0	0	1	0	

3191	12724	tr|J3KRF8|J3KRF8_HUMAN	57.13			1.67E+06		1	1	0	0	1	0	
3191	12725	tr|J3QR41|J3QR41_HUMAN	57.13			1.67E+06		1	1	0	0	1	0	
3191	12726	tr|J3KTD6|J3KTD6_HUMAN	57.13			1.67E+06		1	1	0	0	1	0	
3191	12727	tr|J3QLN8|J3QLN8_HUMAN	57.13			1.67E+06		1	1	0	0	1	0	
2673	7249	sp|Q9Y5K6|CD2AP_HUMAN	57.1	5.11E+05	1.20E+05	3.40E+05		4	4	2	1	1	0	
2711	8044	tr|B4DEM9|B4DEM9_HUMAN	57.08		5.31E+05			3	3	0	1	0	0	
2711	8045	sp|Q9Y2S7|PDIP2_HUMAN	57.08		5.31E+05			3	3	0	1	0	0	
2350	7129	sp|P33240|CSTF2_HUMAN	56.93	1.93E+06	1.75E+06		2.63E+05	3	3	3	2	0	1	
2350	7132	tr|E7EWR4|E7EWR4_HUMAN	56.93	1.93E+06	1.75E+06		2.63E+05	3	3	3	2	0	1	
2350	7267	tr|E9PID8|E9PID8_HUMAN	56.93	1.93E+06	1.75E+06		2.63E+05	3	3	3	2	0	1	
3063	7899	sp|Q6KCM7|SCMC2_HUMAN	56.9	2.68E+05				4	4	1	0	0	0	
3481	9514	tr|A0A087WYS1|A0A087WYS1_HUMAN	56.89	1.97E+05				2	2	1	0	0	0	
3481	9515	sp|Q16851|UGPA_HUMAN	56.89	1.97E+05				2	2	1	0	0	0	
3481	9516	tr|E7EUC7|E7EUC7_HUMAN	56.89	1.97E+05				2	2	1	0	0	0	
3481	10794	tr|C9JWG0|C9JWG0_HUMAN	56.89					1	1	0	0	0	0	
3481	10796	tr|C9JVG3|C9JVG3_HUMAN	56.89					1	1	0	0	0	0	
3481	10797	tr|C9JQU9|C9JQU9_HUMAN	56.89					1	1	0	0	0	0	
3481	10798	tr|C9JNZ1|C9JNZ1_HUMAN	56.89					1	1	0	0	0	0	
3588	11431	sp|P25787|PSA2_HUMAN	56.88		8.98E+05			1	1	0	1	0	0	
3588	11432	tr|A0A024RA52|A0A024RA52_HUMAN	56.88		8.98E+05			1	1	0	1	0	0	
3588	12695	tr|H3BT36|H3BT36_HUMAN	56.88		8.98E+05			1	1	0	1	0	0	
2682	7968	tr|A0A3B3ITL0|A0A3B3ITL0_HUMAN	56.76		7.83E+05		3.10E+05	4	4	0	2	0	2	Carbamid
2682	7969	sp|Q9Y2H2|SAC2_HUMAN	56.76		7.83E+05		3.10E+05	4	4	0	2	0	2	Carbamid
2682	8004	tr|A0A3B3ITR9|A0A3B3ITR9_HUMAN	56.76		7.83E+05		3.10E+05	4	4	0	2	0	2	Carbamid
2682	8114	tr|A0A3B3IRJ9|A0A3B3IRJ9_HUMAN	56.76		6.62E+04		3.10E+05	3	3	0	1	0	2	Carbamid
2998	7956	sp|Q5JRX3|PREP_HUMAN	56.69	4.10E+06	6.41E+06	5.67E+06	9.31E+06	4	4	1	1	1	1	Carbamid
3145	10753	tr|H7C1M5|H7C1M5_HUMAN	56.6	1.70E+04	1.39E+04			1	1	1	1	0	0	
3145	10754	sp|Q9H147|TDIF1_HUMAN	56.6	1.70E+04	1.39E+04			1	1	1	1	0	0	
3145	12712	tr|F2Z2A4|F2Z2A4_HUMAN	56.6	1.70E+04	1.39E+04			1	1	1	1	0	0	
3145	12713	tr|H0Y501|H0Y501_HUMAN	56.6	1.70E+04	1.39E+04			1	1	1	1	0	0	
3137	9250	tr|K7ES52|K7ES52_HUMAN	56.4	1.31E+06	3.36E+05			2	2	2	1	0	0	Carbamid
3137	9251	tr|K7ERJ1|K7ERJ1_HUMAN	56.4	1.31E+06	3.36E+05			2	2	2	1	0	0	Carbamid
3137	9252	sp|P04183|KITH_HUMAN	56.4	1.31E+06	3.36E+05			2	2	2	1	0	0	Carbamid
3137	9253	tr|K7ERV3|K7ERV3_HUMAN	56.4	1.31E+06	3.36E+05			2	2	2	1	0	0	Carbamid
3611	12116	tr|H0Y544|H0Y544_HUMAN	56.26					1	1	0	0	0	0	
3611	12117	sp|Q9UGH3|S23A2_HUMAN	56.26					1	1	0	0	0	0	
2500	10366	sp|P06312|KV401_HUMAN	56.22	1.03E+06	1.17E+06			2	1	1	1	1	1	
3163	8468	sp|Q15554|TERF2_HUMAN	56.2			3.26E+05		3	3	0	0	1	0	
3163	10499	tr|J3KSZ6|J3KSZ6_HUMAN	56.2					2	2	0	0	0	0	
3163	10500	tr|U3KQ35|U3KQ35_HUMAN	56.2					2	2	0	0	0	0	
3163	10501	tr|H3BR06|H3BR06_HUMAN	56.2					2	2	0	0	0	0	
2596	10606	sp|Q14CB8|RHG19_HUMAN	56.2	1.51E+05	2.13E+05			1	1	1	1	0	0	
2596	12703	tr|R4GMS4|R4GMS4_HUMAN	56.2	1.51E+05	2.13E+05			1	1	1	1	0	0	
2800	9162	tr|A0A087WZL0|A0A087WZL0_HUMAN	56.15	2.96E+05			1.66E+05	2	1	2	0	0	1	Deamidat
2912	9074	sp|Q9NYG2|ZDHC3_HUMAN	56.13	5.11E+05	1.95E+05			2	2	1	1	0	0	
2883	8255	sp|Q8N6M0|OTU6B_HUMAN	56.12	6.87E+05	3.71E+05			3	2	3	1	0	0	
2883	8256	tr|A0A087X0W9|A0A087X0W9_HUMAN	56.12	6.87E+05	3.71E+05			3	2	3	1	0	0	
2623	10347	sp|Q9Y3C8|UFC1_HUMAN	56.11	2.15E+06				2	2	2	0	0	0	
2492	10441	sp|O43731|ERD23_HUMAN	56.08	3.87E+05		9.98E+05	1.16E+06	2	2	1	0	1	2	
3017	8077	sp|Q07617|SPAG1_HUMAN	56.06	1.17E+06			2.06E+05	5	5	2	0	0	1	Carbamid
3113	9720	sp|O75600|KBL_HUMAN	55.97	1.85E+05				1	1	1	0	0	0	
2274	7522	sp|Q9UQN3|CHM2B_HUMAN	55.91	1.35E+07	8.78E+06	6.69E+06	4.66E+06	4	4	4	3	2	1	
3058	9390	sp|Q9BVC3|DCC1_HUMAN	55.84				4.10E+05	2	2	0	0	0	1	Carbamid
2890	8925	tr|F8W785|F8W785_HUMAN	55.84				7.23E+05	2	2	0	0	0	2	
2890	8926	sp|O00461|GOLI4_HUMAN	55.84				7.23E+05	2	2	0	0	0	2	
3134	8903	sp|Q9NYL2|M3K20_HUMAN	55.69				1.56E+06	2	2	0	0	0	1	Acetylatic
3585	10903	tr|I3L1T3|I3L1T3_HUMAN	55.66		7.42E+05			2	2	0	1	0	0	
3585	10905	sp|Q9GZP9|DERL2_HUMAN	55.66		7.42E+05			2	2	0	1	0	0	
2321	8264	sp|Q6P158|DHX57_HUMAN	55.65			4.06E+05	1.07E+05	3	1	1	1	3	2	Carbamid
3494	10677	sp|Q9BVS5|TR61B_HUMAN	55.62					1	1	0	0	0	0	Carbamid
3494	11853	tr|F8WDR2|F8WDR2_HUMAN	55.62					1	1	0	0	0	0	Carbamid
2499	8087	sp|Q8N5L8|RP25L_HUMAN	55.61		3.02E+05	1.46E+06	1.05E+06	3	3	0	1	1	1	
3546	11274	tr|A0A0A0MSQ0|A0A0A0MSQ0_HUMAN	55.52					1	1	0	0	0	0	
3546	11275	sp|P13797|PLST_HUMAN	55.52					1	1	0	0	0	0	
3059	9645	tr|B4DKY1|B4DKY1_HUMAN	55.46	5.04E+05				1	1	1	0	0	0	
3059	9646	sp|P49589|SYCC_HUMAN	55.46	5.04E+05				1	1	1	0	0	0	
2624	10385	sp|P14174|MIF_HUMAN	55.38	1.52E+06	4.72E+05	1.33E+06		2	2	1	2	1	0	Carbamid
2867	8589	sp|Q15291|RBBP5_HUMAN	55.27					2	2	0	0	0	0	Carbamid
3427	8697	tr|X6RJP6|X6RJP6_HUMAN	55.2			2.57E+06		2	2	0	0	2	0	Deamidat
3427	8698	sp|P37802|TAGL2_HUMAN	55.2			2.57E+06		2	2	0	0	2	0	Deamidat
3544	11118	sp|Q92750|TAF4B_HUMAN	55.14					1	1	0	0	0	0	
3544	11119	tr|J3KTH2|J3KTH2_HUMAN	55.14					1	1	0	0	0	0	
3544	11374	sp|O00268|TAF4_HUMAN	55.14					1	1	0	0	0	0	
3544	12127	tr|A0A0G2JRY5|A0A0G2JRY5_HUMAN	55.14					1	1	0	0	0	0	
3544	12740	tr|V9GY03|V9GY03_HUMAN	55.14					1	1	0	0	0	0	
3010	8642	tr|F8VVX6|F8VVX6_HUMAN	55.14	2.26E+05	1.01E+05			2	2	1	1	0	0	
3010	8937	sp|Q5HYK3|COQ5_HUMAN	55.14	2.26E+05	1.01E+05			2	2	1	1	0	0	
3097	9210	sp|Q8NE86|MCU_HUMAN	55.04	3.89E+04			3.32E+05	2	2	1	0	0	2	
2792	7866	sp|P49915|GUAA_HUMAN	54.9		6.07E+04		1.17E+05	3	3	0	1	0	1	Carbamid
2536	6727	tr|A0A0B4J2G4|A0A0B4J2G4_HUMAN	54.9		8.94E+05		7.20E+05	4	4	0	1	0	1	Carbamid
2536	6728	sp|Q15345|LRC41_HUMAN	54.9		8.94E+05		7.20E+05	4	4	0	1	0	1	Carbamid
2536	6821	tr|A0A087WTU1|A0A087WTU1_HUMAN	54.9		8.94E+05		7.20E+05	4	4	0	1	0	1	Carbamid
2735	9172	sp|O14965|AURKA_HUMAN	54.81	2.46E+05	3.28E+05		4.16E+05	2	2	1	2	0	1	Carbamid
2567	7070	sp|Q86XL3|ANKL2_HUMAN	54.72	2.52E+05		1.36E+05	4.91E+06	6	6	2	0	1	1	Carbamid
2474	8650	sp|Q9NRP2|COXM2_HUMAN	54.71	1.33E+06	7.80E+05		1.47E+06	3	3	3	2	0	1	Carbamid
2474	11116	tr|H3BNN8|H3BNN8_HUMAN	54.71	1.33E+06	7.80E+05		1.47E+06	2	2	3	2	0	1	Carbamid
2474	11117	tr|H3BTB6|H3BTB6_HUMAN	54.71	1.33E+06	7.80E+05		1.47E+06	2	2	3	2	0	1	Carbamid
2474	12781	tr|H3BQQ1|H3BQQ1_HUMAN	54.71	1.33E+06	7.80E+05		1.47E+06	1	1	3	2	0	1	Carbamid
3052	8512	tr|K7EQ34|K7EQ34_HUMAN	54.58	4.00E+05		5.07E+05		2	2	1	0	1	0	
3052	8513	tr|U3KQT6|U3KQT6_HUMAN	54.58	4.00E+05		5.07E+05		2	2	1	0	1	0	
3052	8514	tr|K7EPA3|K7EPA3_HUMAN	54.58	4.00E+05		5.07E+05		2	2	1	0	1	0	
3052	8515	sp|Q9NX61|T161A_HUMAN	54.58	4.00E+05		5.07E+05		2	2	1	0	1	0	
2896	10793	sp|Q96BD8|SKA1_HUMAN	54.46	3.35E+05	2.04E+05			1	1	1	1	0	0	
2966	10466	tr|V9GY01|V9GY01_HUMAN	54.44	2.07E+05		2.71E+05		2	2	1	0	1	0	
2966	10467	sp|Q9Y448|SKAP_HUMAN	54.44	2.07E+05		2.71E+05		2	2	1	0	1	0	
2646	6912	tr|A0A2R8Y8D8|A0A2R8Y8D8_HUMAN	54.38	1.07E+05		1.26E+06	3.82E+05	6	6	2	0	2	2	
2646	6913	tr|A0A2R8Y5E1|A0A2R8Y5E1_HUMAN	54.38	1.07E+05		1.26E+06	3.82E+05	6	6	2	0	2	2	
2646	6914	tr|A0A2R8YFD4|A0A2R8YFD4_HUMAN	54.38	1.07E+05		1.26E+06	3.82E+05	6	6	2	0	2	2	
2646	6915	sp|Q13191|CBLB_HUMAN	54.38	1.07E+05		1.26E+06	3.82E+05	6	6	2	0	2	2	
2646	6916	tr|H0Y3L9|H0Y3L9_HUMAN	54.38	1.07E+05		1.26E+06	3.82E+05	6	6	2	0	2	2	
2570	7686	sp|Q8IUR7|ARMC8_HUMAN	54.32	1.59E+05				4	4	1	0	0	0	Carbamid
2570	7872	tr|B7Z637|B7Z637_HUMAN	54.32	1.59E+05				4	4	1	0	0	0	Carbamid
3661	12749	tr|B8ZZC5|B8ZZC5_HUMAN	54.29					1	1	0	0	0	0	
3661	12750	tr|B8ZZA8|B8ZZA8_HUMAN	54.29					1	1	0	0	0	0	
2888	8652	sp|Q8NFX7|STXB6_HUMAN	54.26	8.40E+05			3.50E+05	2	2	1	0	0	1	
2766	9345	sp|Q9BQQ3|GORS1_HUMAN	54.13		5.33E+05			2	2	0	1	0	0	
2656	6692	tr|I3L0N3|I3L0N3_HUMAN	54.11		1.17E+05		2.21E+05	4	3	1	1	1	2	Carbamid
3092	8749	sp|Q9H0E3|SP130_HUMAN	54.08	2.02E+05	4.73E+05			2	2	1	1	0	0	
3092	8750	tr|H7BXF5|H7BXF5_HUMAN	54.08	2.02E+05	4.73E+05			2	2	1	1	0	0	
3092	8751	tr|A0A2R8YDB8|A0A2R8YDB8_HUMAN	54.08	2.02E+05	4.73E+05			2	2	1	1	0	0	
3053	8522	tr|H7C1F9|H7C1F9_HUMAN	54.04					2	2	0	0	0	0	Carbamid
3053	8527	sp|Q2PPJ7|RGPA2_HUMAN	54.04					2	2	0	0	0	0	Carbamid
3053	9705	tr|H7BZA8|H7BZA8_HUMAN	54.04					2	2	0	0	0	0	Carbamid
3053	10348	tr|A0A087WYA8|A0A087WYA8_HUMAN	54.04					2	2	0	0	0	0	Carbamid
2965	10401	sp|Q9H4E5|RHOJ_HUMAN	54.01		9.59E+04			2	1	1	2	0	0	Carbamid
2828	7784	sp|P51608|MECP2_HUMAN	53.9		1.03E+06			4	4	0	3	0	0	

3498	10856	tr|A0A1W2PNM1|A0A1W2PNM1_HUMAN	53.89					1	1	0	0	0	0	
3498	10859	sp|Q16836|HCDH_HUMAN	53.89					1	1	0	0	0	0	
3498	10861	tr|A0A0A0MSE2|A0A0A0MSE2_HUMAN	53.89					1	1	0	0	0	0	
3498	10862	tr|A0A0D9SFP2|A0A0D9SFP2_HUMAN	53.89					1	1	0	0	0	0	
3498	10867	tr|E9PF18|E9PF18_HUMAN	53.89					1	1	0	0	0	0	
3498	11802	tr|A0A1W2PQC2|A0A1W2PQC2_HUMAN	53.89					1	1	0	0	0	0	
3498	11803	tr|A0A1W2PRT2|A0A1W2PRT2_HUMAN	53.89					1	1	0	0	0	0	
3498	11806	tr|A0A1W2PQV5|A0A1W2PQV5_HUMAN	53.89					1	1	0	0	0	0	
2877	7846	sp|Q86VN1|VPS36_HUMAN	53.76			3.48E+05		3	3	0	0	2	0	Carbamid
2960	8141	sp|Q9UQR0|SCML2_HUMAN	53.71		6.21E+05			3	3	0	1	0	0	Carbamid
2716	8950	sp|Q16831|UPP1_HUMAN	53.68	8.15E+04	4.00E+04			2	2	1	1	0	0	Carbamid
3662	12763	tr|E9PNS3|E9PNS3_HUMAN	53.65					1	1	0	0	0	0	
3662	12764	tr|F2Z2Y8|F2Z2Y8_HUMAN	53.65					1	1	0	0	0	0	
3662	12765	sp|Q9H0E2|TOLIP_HUMAN	53.65					1	1	0	0	0	0	
2813	7887	sp|Q8TEQ6|GEMI5_HUMAN	53.6			2.87E+05	3.76E+05	4	4	0	0	1	2	Carbamid
2762	7779	sp|P61019|RAB2A_HUMAN	53.58	0				3	2	1	2	0	0	
2762	7898	tr|A0A3B3ITL1|A0A3B3ITL1_HUMAN	53.58	0				3	2	1	2	0	0	
2762	7900	sp|Q8WUD1|RAB2B_HUMAN	53.58	0				3	2	1	2	0	0	
3374	8597	sp|Q9NUQ7|UFSP2_HUMAN	53.49					2	2	0	0	0	0	Carbamid
3374	8958	tr|H0YA18|H0YA18_HUMAN	53.49					2	2	0	0	0	0	Carbamid
3374	8972	tr|H0Y9B0|H0Y9B0_HUMAN	53.49					2	2	0	0	0	0	Carbamid
2850	8012	sp|Q9H3G5|CPVL_HUMAN	53.47	7.15E+05	1.66E+05		2.95E+05	3	3	1	1	0	1	
3089	8600	sp|Q9Y6X9|MORC2_HUMAN	53.4		2.42E+05			2	2	0	1	0	0	Carbamid
3606	11994	tr|J3KSP0|J3KSP0_HUMAN	53.4					1	1	0	0	0	0	
3606	11995	sp|Q8WVK7|SKA2_HUMAN	53.4					1	1	0	0	0	0	
3606	12768	tr|J3QS74|J3QS74_HUMAN	53.4					1	1	0	0	0	0	
3606	12769	tr|J3KRZ0|J3KRZ0_HUMAN	53.4					1	1	0	0	0	0	
3606	12770	tr|J3QL03|J3QL03_HUMAN	53.4					1	1	0	0	0	0	
2325	9003	sp|Q9H8P0|PORED_HUMAN	53.37	2.40E+05	6.61E+05		1.70E+06	2	2	1	1	0	4	
2938	8978	sp|Q6YP21|KAT3_HUMAN	53.37		2.03E+05	1.70E+05	4.29E+05	2	2	0	1	1	1	
2593	9260	tr|C9J2C4|C9J2C4_HUMAN	53.12	4.82E+05	1.94E+05	1.34E+05		2	2	1	2	1	0	Deamidat
2593	9261	sp|O75190|DNJB6_HUMAN	53.12	4.82E+05	1.94E+05	1.34E+05		2	2	1	2	1	0	Deamidat
2593	9262	tr|A0A0J9YX62|A0A0J9YX62_HUMAN	53.12	4.82E+05	1.94E+05	1.34E+05		2	2	1	2	1	0	Deamidat
2593	10696	tr|C9JDR7|C9JDR7_HUMAN	53.12	4.82E+05	1.94E+05	1.34E+05		2	2	1	2	1	0	Deamidat
2593	10697	tr|C9JDX6|C9JDX6_HUMAN	53.12	4.82E+05	1.94E+05	1.34E+05		2	2	1	2	1	0	Deamidat
2593	10698	tr|F8WCZ4|F8WCZ4_HUMAN	53.12	4.82E+05	1.94E+05	1.34E+05		2	2	1	2	1	0	Deamidat
2593	10699	tr|C9J2P2|C9J2P2_HUMAN	53.12	4.82E+05	1.94E+05	1.34E+05		2	2	1	2	1	0	Deamidat
2593	10700	tr|E9PH18|E9PH18_HUMAN	53.12	4.82E+05	1.94E+05	1.34E+05		2	2	1	2	1	0	Deamidat
2703	10689	tr|H0YDU8|H0YDU8_HUMAN	53.04	1.28E+05		4.32E+05		1	1	1	0	1	0	
2703	10690	sp|P53041|PPP5_HUMAN	53.04	1.28E+05		4.32E+05		1	1	1	0	1	0	
2703	11121	tr|A8MU39|A8MU39_HUMAN	53.04	1.28E+05		4.32E+05		1	1	1	0	1	0	
2658	6937	sp|Q2M389|WASC4_HUMAN	52.86	1.15E+05	1.33E+05		1.08E+05	5	5	1	1	0	1	Carbamid
2658	6938	tr|A0A087X256|A0A087X256_HUMAN	52.86	1.15E+05	1.33E+05		1.08E+05	5	5	1	1	0	1	Carbamid
2923	11296	sp|Q8IWF6|DEN6A_HUMAN	52.75	1.80E+05	1.55E+05			1	1	1	1	0	0	
2923	12791	tr|H7C598|H7C598_HUMAN	52.75	1.80E+05	1.55E+05			1	1	1	1	0	0	
3087	8494	sp|Q9NTZ6|RBM12_HUMAN	52.63	7.98E+05			3.56E+05	2	2	1	0	0	1	
3116	10767	tr|Q5RGS4|Q5RGS4_HUMAN	52.59	9.08E+05			3.21E+05	1	1	1	0	0	1	
3116	10768	sp|Q9HB21|PKHA1_HUMAN	52.59	9.08E+05			3.21E+05	1	1	1	0	0	1	
3028	8290	sp|Q9P2I0|CPSF2_HUMAN	52.52					2	2	0	0	0	0	Carbamid
2637	10404	sp|Q14814|MEF2D_HUMAN	52.52		6.31E+05		3.11E+05	2	2	0	3	0	1	
2840	8068	sp|P13994|CC130_HUMAN	52.47	2.33E+05	3.67E+05			3	3	2	1	0	0	
3545	11183	sp|O75362|ZN217_HUMAN	52.39	5.54E+04				1	1	1	0	0	0	
2675	7687	sp|P25786|PSA1_HUMAN	52.38	1.18E+06	7.21E+05			5	4	2	3	0	0	
2831	8245	sp|Q9UL18|AGO1_HUMAN	52.34	6.24E+04	3.03E+05	2.28E+06	1.56E+05	5	5	1	2	1	1	Carbamid
2830	8138	sp|Q9H1X3|DJC25_HUMAN	52.18	4.52E+05	1.94E+06			3	3	1	3	0	0	Carbamid
2830	8963	tr|A0A024R161|A0A024R161_HUMAN	52.18	4.52E+05	1.55E+06			2	2	1	2	0	0	Carbamid
2830	9117	tr|F2Z3D1|F2Z3D1_HUMAN	52.18	4.52E+05	1.55E+06			2	2	1	2	0	0	Carbamid
2830	9118	tr|J3QR79|J3QR79_HUMAN	52.18	4.52E+05	1.55E+06			2	2	1	2	0	0	Carbamid
3665	12813	tr|H0Y8R3|H0Y8R3_HUMAN	52.17				2.22E+04	1	1	0	0	0	1	Carbamid
3665	12814	sp|Q06330|SUH_HUMAN	52.17				2.22E+04	1	1	0	0	0	1	Carbamid
2674	7513	sp|O95373|IPO7_HUMAN	52.16			1.11E+05		4	4	0	0	1	0	Carbamid
3599	11771	sp|Q9Y478|AAKB1_HUMAN	52.14		6.60E+04			1	1	0	1	0	0	
3599	12800	tr|F5H610|F5H610_HUMAN	52.14		6.60E+04			1	1	0	1	0	0	
3599	12801	tr|F5H2X8|F5H2X8_HUMAN	52.14		6.60E+04			1	1	0	1	0	0	
3203	10448	sp|Q9Y394|DHRS7_HUMAN	52.11		1.50E+05			2	2	0	1	0	0	
3203	10449	tr|A0A087X0Z7|A0A087X0Z7_HUMAN	52.11		1.50E+05			2	2	0	1	0	0	
3203	10450	tr|H0YJ66|H0YJ66_HUMAN	52.11		1.50E+05			2	2	0	1	0	0	
2729	8027	sp|Q5T5Y3|CAMP1_HUMAN	52.09	6.91E+05	6.56E+05			3	3	3	3	0	0	Carbamid
2475	8970	sp|P35625|TIMP3_HUMAN	52.01		1.04E+06	1.09E+06		2	2	0	2	2	0	Carbamid
2671	6815	tr|A0A2R8YDF3|A0A2R8YDF3_HUMAN	51.9	2.75E+05	1.16E+05			4	4	1	1	0	0	
2671	6816	tr|A0A2R8Y6L5|A0A2R8Y6L5_HUMAN	51.9	2.75E+05	1.16E+05			4	4	1	1	0	0	
2671	6822	tr|A0A2R8Y443|A0A2R8Y443_HUMAN	51.9	2.75E+05	1.16E+05			4	4	1	1	0	0	
2671	6823	tr|A0A2R8Y6I6|A0A2R8Y6I6_HUMAN	51.9	2.75E+05	1.16E+05			4	4	1	1	0	0	
2671	6824	sp|Q13627|DYR1A_HUMAN	51.9	2.75E+05	1.16E+05			4	4	1	1	0	0	
3330	9078	sp|O75319|DUS11_HUMAN	51.87					1	1	0	0	0	0	Carbamid
3330	9249	tr|H7C0E6|H7C0E6_HUMAN	51.87					1	1	0	0	0	0	Carbamid
2956	12815	tr|U3KQF3|U3KQF3_HUMAN	51.82	0			5.89E+04	1	1	1	0	0	1	
2956	12816	tr|U3KPZ0|U3KPZ0_HUMAN	51.82	0			5.89E+04	1	1	1	0	0	1	
2956	12817	sp|P60174|TPIS_HUMAN	51.82	0			5.89E+04	1	1	1	0	0	1	
3208	10548	sp|Q8N0U8|VKORL_HUMAN	51.76	3.96E+05				2	2	2	0	0	0	Carbamid
3208	10549	tr|A0A3B3ISV4|A0A3B3ISV4_HUMAN	51.76	3.96E+05				2	2	2	0	0	0	Carbamid
3483	9577	tr|K7EJS5|K7EJS5_HUMAN	51.6					2	2	0	0	0	0	Carbamid
3483	9578	sp|Q6NUT3|MFS12_HUMAN	51.6					2	2	0	0	0	0	Carbamid
3084	8311	tr|F5H148|F5H148_HUMAN	51.56	5.01E+04	3.91E+05			3	3	1	2	0	0	
3084	8312	tr|H3BMT1|H3BMT1_HUMAN	51.56	5.01E+04	3.91E+05			3	3	1	2	0	0	
3084	8313	sp|Q9NYV6|RRN3_HUMAN	51.56	5.01E+04	3.91E+05			3	3	1	2	0	0	
3084	9583	sp|A6NIE6|RN3P2_HUMAN	51.56	5.01E+04	1.17E+05			2	2	1	1	0	0	
3560	12220	sp|Q86SK9|SCD5_HUMAN	51.5			8.92E+05		1	1	0	0	1	0	
2949	10826	tr|H0YJD0|H0YJD0_HUMAN	51.49	1.29E+06		8.20E+05		2	2	2	0	1	0	Carbamid
2949	10828	sp|Q9NYT0|PLEK2_HUMAN	51.49	1.29E+06		8.20E+05		2	2	2	0	1	0	Carbamid
3663	12794	sp|Q9BW83|IFT27_HUMAN	51.47					1	1	0	0	0	0	Carbamid
3664	12795	sp|O00399|DCTN6_HUMAN	51.46					1	1	0	0	0	0	
2573	8303	tr|Q5TBI2|Q5TBI2_HUMAN	51.42	2.65E+06	3.56E+05	2.36E+05		4	4	4	1	1	0	Deamidat
2573	8304	sp|Q8NDD1|CA131_HUMAN	51.42	2.65E+06	3.56E+05	2.36E+05		4	4	4	1	1	0	Deamidat
2579	10522	sp|A0PJW6|TM223_HUMAN	51.38	2.34E+07	1.86E+07	2.21E+07	2.01E+07	2	2	1	1	2	2	
2495	12063	sp|P00846|ATP6_HUMAN	51.11	2.42E+06	2.59E+06	3.87E+06	6.61E+06	1	1	1	1	1	1	
2537	7825	sp|Q8WWM7|ATX2L_HUMAN	50.9		2.63E+05	3.42E+05	7.65E+05	6	6	0	1	1	2	
3215	12826	tr|H0YK33|H0YK33_HUMAN	50.77	1.44E+05	1.01E+05			1	1	1	1	0	0	
3215	12827	sp|Q6UUV7|CRTC3_HUMAN	50.77	1.44E+05	1.01E+05			1	1	1	1	0	0	
2560	8918	sp|P61129|ZC3H6_HUMAN	50.71		1.16E+06	1.37E+06		2	1	0	4	3	1	
2836	8860	tr|A0A0C4DH01|A0A0C4DH01_HUMAN	50.69					2	2	0	0	0	0	
2836	8861	sp|Q9NVU0|RPC5_HUMAN	50.69					2	2	0	0	0	0	
2878	7848	sp|P30038|AL4A1_HUMAN	50.58				1.33E+05	3	3	0	0	0	1	
3121	11011	sp|Q8WUM4|PDC6I_HUMAN	50.54	4.42E+05				1	1	1	0	0	0	
3444	9802	sp|Q9Y613|FHOD1_HUMAN	50.4	1.37E+05	9.36E+05			2	2	1	1	0	0	
2972	12856	sp|Q15035|TRAM2_HUMAN	50.26				8.85E+05	1	1	0	0	0	1	
3617	12834	sp|Q96CX2|KCD12_HUMAN	50.25					1	1	0	0	0	0	
2764	8147	tr|G5E931|G5E931_HUMAN	50.21	1.25E+06	9.50E+05			3	3	2	1	0	0	
2764	8148	tr|H3BN57|H3BN57_HUMAN	50.21	1.25E+06	9.50E+05			3	3	2	1	0	0	
2764	8149	sp|Q8TDH9|BL1S5_HUMAN	50.21	1.25E+06	9.50E+05			3	3	2	1	0	0	
2721	9181	tr|K7EJL1|K7EJL1_HUMAN	50.18		1.13E+05	2.19E+05	3.87E+05	2	2	0	1	1	1	Acetylatic
2721	9182	sp|Q9BXS5|AP1M1_HUMAN	50.18		1.13E+05	2.19E+05	3.87E+05	2	2	0	1	1	1	Acetylatic
2721	9234	tr|E7ENJ6|E7ENJ6_HUMAN	50.18		1.13E+05	2.19E+05	3.87E+05	2	2	0	1	1	1	Acetylatic
3609	12033	tr|J3KRG0|J3KRG0_HUMAN	50.15					1	1	0	0	0	0	
3609	12034	tr|J3QR36|J3QR36_HUMAN	50.15					1	1	0	0	0	0	
3609	12035	tr|J3KTN3|J3KTN3_HUMAN	50.15					1	1	0	0	0	0	

3609	12036	tr|J3KSW2|J3KSW2_HUMAN	50.15					1	1	0	0	0	0	
3609	12037	tr|J3KQ09|J3KQ09_HUMAN	50.15					1	1	0	0	0	0	
3609	12038	sp|Q9UNA4|POLI_HUMAN	50.15					1	1	0	0	0	0	
3173	10488	tr|F8VXY6|F8VXY6_HUMAN	50.02		5.10E+04			2	2	0	1	0	0	
3173	10490	tr|G3V1V1|G3V1V1_HUMAN	50.02		5.10E+04			2	2	0	1	0	0	
3173	10491	sp|Q8TBF4|ZCRB1_HUMAN	50.02		5.10E+04			2	2	0	1	0	0	
3207	10537	sp|Q6UX07|DHR13_HUMAN	49.94			9.61E+04		2	2	0	0	1	0	Carbamid
3666	12831	sp|Q96NB2|SFXN2_HUMAN	49.94					1	1	0	0	0	0	
3504	11050	sp|P13693|TCTP_HUMAN	49.93			9.87E+04		1	1	0	0	1	0	
3504	11051	tr|Q5W0H4|Q5W0H4_HUMAN	49.93			9.87E+04		1	1	0	0	1	0	
3504	11052	tr|A0A0B4J2C3|A0A0B4J2C3_HUMAN	49.93			9.87E+04		1	1	0	0	1	0	
2510	7083	sp|Q9Y4K4|M4K5_HUMAN	49.91	1.25E+06		0		5	4	2	0	2	0	Carbamid
3602	11921	sp|Q9BYI3|HYCCI_HUMAN	49.81					1	1	0	0	0	0	
3602	12846	tr|H7C0W7|H7C0W7_HUMAN	49.81					1	1	0	0	0	0	
3278	9447	sp|P53350|PLK1_HUMAN	49.6					1	1	0	0	0	0	Carbamid
3025	7869	sp|Q9UNS2|CSN3_HUMAN	49.57	2.30E+05				4	4	1	0	0	0	Carbamid
2918	9476	sp|Q69YL0|NCAS2_HUMAN	49.42				3.00E+06	1	1	0	0	0	1	Carbamid
3552	11505	tr|K7EJB9|K7EJB9_HUMAN	49.35	1.10E+06				1	1	1	0	0	0	
3552	11506	sp|P27797|CALR_HUMAN	49.35	1.10E+06				1	1	1	0	0	0	
3587	11282	sp|Q6P2I3|FAH2B_HUMAN	49.31	1.16E+05				1	1	1	0	0	0	Carbamid
3587	12859	sp|Q96GK7|FAH2A_HUMAN	49.31	1.16E+05				1	1	1	0	0	0	Carbamid
2201	7945	tr|A0A087X0M4|A0A087X0M4_HUMAN	49.23	5.36E+05	8.54E+05	5.17E+05	9.40E+05	3	3	1	2	1	2	Carbamid
2201	7946	sp|Q9BWU0|NADAP_HUMAN	49.23	5.36E+05	8.54E+05	5.17E+05	9.40E+05	3	3	1	2	1	2	Carbamid
2561	10461	sp|P84101|SERF2_HUMAN	49.21	6.18E+06	1.11E+06	8.07E+05		2	2	3	1	2	0	
2887	8582	sp|Q9H3N1|TMX1_HUMAN	49.01	4.66E+05	4.93E+05			2	2	1	1	0	0	Carbamid
3020	8423	sp|Q96SB8|SMC6_HUMAN	48.85			3.42E+05		2	2	0	0	1	0	
2476	9164	sp|Q92887|MRP2_HUMAN	48.85	6.55E+04	9.66E+04	2.61E+05		2	1	1	1	2	1	
3491	10398	sp|Q13751|LAMB3_HUMAN	48.83	2.27E+04				1	1	1	0	0	0	
2367	9179	sp|Q7KZN9|COX15_HUMAN	48.56	2.92E+05		1.80E+06	2.19E+06	2	2	1	0	3	3	
2942	9493	sp|Q9H3K6|BOLA2_HUMAN	48.55	9.23E+05		1.22E+05		2	2	2	0	1	0	Acetylatic
3476	8567	sp|A8CG34|P121C_HUMAN	48.54	1.82E+06	6.34E+05		1.33E+06	3	3	1	1	0	2	Carbamid
3476	8568	sp|Q96HA1|P121A_HUMAN	48.54	1.82E+06	6.34E+05		1.33E+06	3	3	1	1	0	2	Carbamid
3476	11112	tr|C9JER0|C9JER0_HUMAN	48.54	1.82E+06			3.39E+05	2	2	1	0	0	1	Carbamid
3476	11113	sp|Q6PJE2|POZP3_HUMAN	48.54	1.82E+06			3.39E+05	2	2	1	0	0	1	Carbamid
3476	11114	tr|A0A0J9YWR1|A0A0J9YWR1_HUMAN	48.54	1.82E+06			3.39E+05	2	2	1	0	0	1	Carbamid
3476	11115	tr|C9J370|C9J370_HUMAN	48.54	1.82E+06			3.39E+05	2	2	1	0	0	1	Carbamid
2676	7977	tr|H0YKC5|H0YKC5_HUMAN	48.49	9.69E+05	1.00E+06	3.56E+05		3	3	1	2	1	0	Carbamid
2676	7980	sp|P33316|DUT_HUMAN	48.49	9.69E+05	1.00E+06	3.56E+05		3	3	1	2	1	0	Carbamid
2676	8053	tr|A0A0C4DGL3|A0A0C4DGL3_HUMAN	48.49	9.69E+05	1.00E+06	3.56E+05		3	3	1	2	1	0	Carbamid
2676	8054	tr|H0YNW5|H0YNW5_HUMAN	48.49	9.69E+05	1.00E+06	3.56E+05		3	3	1	2	1	0	Carbamid
3152	12053	sp|Q5VT52|RPRD2_HUMAN	48.41	1.22E+05	1.40E+05			1	1	1	1	0	0	
2662	8197	tr|A0A2R8YCX2|A0A2R8YCX2_HUMAN	48.35	1.67E+06	9.50E+05		4.97E+05	3	3	2	1	0	1	
2662	8198	tr|A0A2R8YGP2|A0A2R8YGP2_HUMAN	48.35	1.67E+06	9.50E+05		4.97E+05	3	3	2	1	0	1	
2662	8199	sp|Q5TBB1|RNH2B_HUMAN	48.35	1.67E+06	9.50E+05		4.97E+05	3	3	2	1	0	1	
3667	12858	sp|Q5T7V8|GORAB_HUMAN	48.33					1	1	0	0	0	0	
3206	10527	tr|B5MCD7|B5MCD7_HUMAN	48.27	3.22E+05				2	2	2	0	0	0	
3206	10528	sp|O43759|SNG1_HUMAN	48.27	3.22E+05				2	2	2	0	0	0	
3450	10669	tr|A0A0A0MSH9|A0A0A0MSH9_HUMAN	47.99				1.14E+04	1	1	0	0	0	1	
3450	10672	sp|Q86WJ1|CHD1L_HUMAN	47.99				1.14E+04	1	1	0	0	0	1	
3450	10713	tr|A0A0A0MRH8|A0A0A0MRH8_HUMAN	47.99				1.14E+04	1	1	0	0	0	1	
2713	8543	tr|H0YN65|H0YN65_HUMAN	47.89	4.22E+05		3.81E+05		2	2	1	0	1	0	
2713	8544	sp|Q8NCH0|CHSTE_HUMAN	47.89	4.22E+05		3.81E+05		2	2	1	0	1	0	
2842	8343	sp|P34932|HSP74_HUMAN	47.77					2	2	0	0	0	0	
2631	12918	sp|Q9BS18|APC13_HUMAN	47.75	1.50E+06	6.86E+05	9.83E+05	3.83E+05	1	1	1	1	1	1	
2503	12900	sp|Q9NWT8|AKIP_HUMAN	47.67	2.18E+06	4.07E+06	1.43E+07	2.14E+07	1	1	1	1	1	1	
2614	10612	sp|Q9BUB7|TMM70_HUMAN	47.65		4.40E+05	2.87E+05	6.07E+05	1	1	0	1	2	1	
3668	12874	tr|C9JGR9|C9JGR9_HUMAN	47.61		2.20E+05			1	1	0	1	0	0	
3668	12876	tr|E7EQ49|E7EQ49_HUMAN	47.61		2.20E+05			1	1	0	1	0	0	
3668	12877	tr|A0A087WTQ2|A0A087WTQ2_HUMAN	47.61		2.20E+05			1	1	0	1	0	0	
3668	12879	sp|Q9Y2I8|WDR37_HUMAN	47.61		2.20E+05			1	1	0	1	0	0	
3668	12881	tr|A0A3B3IT47|A0A3B3IT47_HUMAN	47.61		2.20E+05			1	1	0	1	0	0	
2755	9415	tr|Q5JWM4|Q5JWM4_HUMAN	47.58			1.56E+05	8.84E+05	2	2	0	0	1	3	Carbamid
2755	9418	sp|Q9H6P5|TASP1_HUMAN	47.58			1.56E+05	8.84E+05	2	2	0	0	1	3	Carbamid
3528	8518	sp|Q9C0I1|MTMRC_HUMAN	47.58	1.11E+05	4.21E+04	1.95E+05		3	3	1	1	1	0	Carbamid
3482	9519	sp|Q9NZH0|GPC5B_HUMAN	47.53			2.32E+05		1	1	0	0	1	0	
3618	12873	sp|Q9NPJ3|ACO13_HUMAN	47.46					1	1	0	0	0	0	
3596	11638	tr|J3QR27|J3QR27_HUMAN	47.39					1	1	0	0	0	0	
2913	9128	tr|G3V3I1|G3V3I1_HUMAN	47.37	7.60E+05	7.54E+05			2	2	1	1	0	0	
2913	9129	tr|G3V295|G3V295_HUMAN	47.37	7.60E+05	7.54E+05			2	2	1	1	0	0	
2913	9130	sp|P60900|PSA6_HUMAN	47.37	7.60E+05	7.54E+05			2	2	1	1	0	0	
2913	9131	tr|G3V5Z7|G3V5Z7_HUMAN	47.37	7.60E+05	7.54E+05			2	2	1	1	0	0	
2913	11007	tr|G3V3U4|G3V3U4_HUMAN	47.37	7.60E+05	7.54E+05			2	2	1	1	0	0	
2622	9421	sp|A6NIH7|U119B_HUMAN	47.35	5.98E+05	3.82E+05		1.80E+05	2	2	2	1	0	1	
3133	8664	sp|Q92604|LGAT1_HUMAN	47.18					2	2	0	0	0	0	
3175	10725	sp|Q6UWH4|GAK1B_HUMAN	47.04		7.63E+05		4.46E+05	2	2	0	1	0	1	
2779	10712	sp|P00387|NB5R3_HUMAN	47.03	2.46E+05	8.84E+04			2	2	2	1	0	0	
2678	8323	sp|O95470|SGPL1_HUMAN	46.9	6.84E+04		9.09E+05		2	2	1	0	4	0	
3619	12909	tr|G3V5P8|G3V5P8_HUMAN	46.87	2.72E+05				1	1	1	0	0	0	Carbamid
3619	12910	tr|G3V3B2|G3V3B2_HUMAN	46.87	2.72E+05				1	1	1	0	0	0	Carbamid
3619	12911	sp|O95406|CNIH1_HUMAN	46.87	2.72E+05				1	1	1	0	0	0	Carbamid
3619	12912	tr|G3V4K0|G3V4K0_HUMAN	46.87	2.72E+05				1	1	1	0	0	0	Carbamid
2449	7810	sp|Q8NBN7|RDH13_HUMAN	46.85	7.08E+05	4.39E+05	1.66E+05	3.74E+05	3	3	3	2	1	1	
2455	8730	sp|O75694|NU155_HUMAN	46.77	5.27E+04	1.43E+05	8.68E+04	9.62E+04	2	2	1	1	1	1	
2455	8800	tr|E9PF10|E9PF10_HUMAN	46.77	5.27E+04	1.43E+05	8.68E+04	9.62E+04	2	2	1	1	1	1	
2852	8117	tr|E9PEX6|E9PEX6_HUMAN	46.69			3.63E+05	1.93E+05	3	3	0	0	1	1	Carbamid
2852	8118	sp|P09622|DLDH_HUMAN	46.69			3.63E+05	1.93E+05	3	3	0	0	1	1	Carbamid
2814	8708	sp|Q9BZF1|OSBL8_HUMAN	46.68		1.46E+05		3.00E+05	2	2	0	1	0	1	
2814	8736	tr|F8VQX7|F8VQX7_HUMAN	46.68		1.46E+05		3.00E+05	2	2	0	1	0	1	
2814	8746	tr|F8VUA7|F8VUA7_HUMAN	46.68		1.46E+05		3.00E+05	2	2	0	1	0	1	
2959	8130	sp|Q9H7B4|SMYD3_HUMAN	46.58	2.33E+05	8.71E+05			3	3	1	1	0	0	Carbamid
3216	12904	sp|Q96GI7|FA89A_HUMAN	46.53	2.49E+05	2.34E+05			1	1	1	1	0	0	
3109	9161	tr|B8ZZ77|B8ZZ77_HUMAN	46.47		6.15E+05			2	2	0	2	0	0	Acetylatic
3109	10510	sp|Q9H2H8|PPIL3_HUMAN	46.47		6.15E+05			2	2	0	2	0	0	Acetylatic
3109	10511	tr|H7BZ14|H7BZ14_HUMAN	46.47		6.15E+05			2	2	0	2	0	0	Acetylatic
3161	8434	sp|Q96RS0|TGS1_HUMAN	46.34			8.71E+04		3	3	0	0	1	0	
3205	10526	sp|Q9Y3E7|CHMP3_HUMAN	46.33					2	2	0	0	0	0	
2861	10940	tr|B1AJY5|B1AJY5_HUMAN	46.3	5.01E+05	1.75E+05	4.42E+05		2	2	2	1	1	0	
2861	10941	tr|B1AJY7|B1AJY7_HUMAN	46.3	5.01E+05	1.75E+05	4.42E+05		2	2	2	1	1	0	
2861	10942	sp|O75832|PSD10_HUMAN	46.3	5.01E+05	1.75E+05	4.42E+05		2	2	2	1	1	0	
2861	12905	tr|B1AJY6|B1AJY6_HUMAN	46.3		1.75E+05	4.42E+05		1	1	0	1	1	0	
2769	11023	tr|A6NEM5|A6NEM5_HUMAN	46.3		1.03E+06		6.00E+05	2	2	0	2	0	1	
2769	11024	sp|Q92643|GPI8_HUMAN	46.3		1.03E+06		6.00E+05	2	2	0	2	0	1	
2769	12930	tr|B1AK81|B1AK81_HUMAN	46.3		6.50E+05		6.00E+05	1	1	0	1	0	1	
3124	8225	sp|Q8N9T8|KRI1_HUMAN	46.24			1.17E+06	3.88E+05	3	3	0	0	2	1	Carbamid
3088	8509	sp|Q86WA8|LONP2_HUMAN	46.17					2	2	0	0	0	0	
2904	8215	tr|B7ZBJ4|B7ZBJ4_HUMAN	46.11	4.50E+05				3	1	2	1	0	0	
2904	8216	tr|Q5TAW7|Q5TAW7_HUMAN	46.11	4.50E+05				3	1	2	1	0	0	
2904	8217	sp|Q9H9S4|CB39L_HUMAN	46.11	4.50E+05				3	1	2	1	0	0	
2575	8429	sp|P40616|ARL1_HUMAN	46.1	1.07E+06	3.47E+05		1.01E+06	3	3	1	1	0	1	
2575	8430	tr|F8VYN9|F8VYN9_HUMAN	46.1	1.07E+06	3.47E+05		1.01E+06	3	3	1	1	0	1	
3437	9584	tr|E9PG73|E9PG73_HUMAN	45.82		0			2	1	0	2	0	0	Carbamid
3437	9586	sp|Q13427|PPIG_HUMAN	45.82		0			2	1	0	2	0	0	Carbamid
3437	10966	tr|C9JN15|C9JN15_HUMAN	45.82		0			2	1	0	2	0	0	Carbamid
3437	12919	tr|C9JT64|C9JT64_HUMAN	45.82		0			1	1	0	1	0	0	Carbamid
3437	12920	tr|C9J679|C9J679_HUMAN	45.82		0			1	1	0	1	0	0	Carbamid
3593	11546	tr|M0QXN5|M0QXN5_HUMAN	45.78					1	1	0	0	0	0	

3593	11547	sp|P37198|NUP62_HUMAN	45.78					1	1	0	0	0	0	
3056	8882	sp|Q15545|TAF7_HUMAN	45.57	2.01E+06	5.47E+05			2	2	2	1	0	0	Carbamid
2649	7918	sp|Q15075|EEA1_HUMAN	45.51	1.76E+05	2.01E+05		1.03E+06	4	4	1	1	0	2	
3492	10408	tr|D6R9I2|D6R9I2_HUMAN	45.5					1	1	0	0	0	0	
3492	10409	sp|P52739|ZN131_HUMAN	45.5					1	1	0	0	0	0	
3492	11866	tr|D6RJH2|D6RJH2_HUMAN	45.5					1	1	0	0	0	0	
3217	12934	sp|Q8TD43|TRPM4_HUMAN	45.47			4.00E+04		1	1	0	0	1	0	Carbamid
3320	8284	sp|Q9NZC9|SMAL1_HUMAN	45.44		9.26E+05			3	3	0	1	0	0	
3320	8741	tr|H7BYI2|H7BYI2_HUMAN	45.44		9.26E+05			2	2	0	1	0	0	
3478	8703	sp|Q53H96|P5CR3_HUMAN	45.38				9.86E+04	1	1	0	0	0	1	Carbamid
3478	8704	tr|A0A0A0MQS1|A0A0A0MQS1_HUMAN	45.38				9.86E+04	1	1	0	0	0	1	Carbamid
2616	11966	sp|P05204|HMGN2_HUMAN	45.15		2.37E+06			1	1	0	2	0	0	
2616	12935	sp|Q15651|HMGN3_HUMAN	45.15		2.37E+06			1	1	0	2	0	0	
2616	12936	tr|A0A087WZE9|A0A087WZE9_HUMAN	45.15		2.37E+06			1	1	0	2	0	0	
2869	8966	sp|Q9ULX6|AKP8L_HUMAN	44.96			1.56E+05	3.72E+05	2	2	0	0	1	1	
3130	8565	sp|Q13505|MTX1_HUMAN	44.94	1.32E+06				3	3	2	0	0	0	Carbamid
3130	10851	tr|A0A0C4DFQ1|A0A0C4DFQ1_HUMAN	44.94	1.20E+06				2	2	1	0	0	0	Carbamid
3102	8218	sp|Q01085|TIAR_HUMAN	44.93	0	4.22E+04			3	3	1	1	0	0	
2906	8350	sp|Q9NVX2|NLE1_HUMAN	44.91	3.04E+05		6.31E+05		3	3	2	0	2	0	
2937	8856	tr|A0A1W2PQD2|A0A1W2PQD2_HUMAN	44.9	1.62E+05		1.38E+05		2	2	1	0	1	0	
2937	8857	sp|Q9UBB4|ATX10_HUMAN	44.9	1.62E+05		1.38E+05		2	2	1	0	1	0	
3195	12944	sp|A0A0J9YX35|HV64D_HUMAN	44.83		1.12E+06			1	1	0	2	0	0	
2947	10638	tr|H0YAE8|H0YAE8_HUMAN	44.45	4.16E+05		3.99E+05		2	2	2	0	1	0	
2947	10639	tr|E7EUV9|E7EUV9_HUMAN	44.45	4.16E+05		3.99E+05		2	2	2	0	1	0	
2947	10640	sp|Q14493|SLBP_HUMAN	44.45	4.16E+05		3.99E+05		2	2	2	0	1	0	
2947	10641	tr|F8W8D3|F8W8D3_HUMAN	44.45	4.16E+05		3.99E+05		2	2	2	0	1	0	
2594	9715	tr|A0A0C4DG68|A0A0C4DG68_HUMAN	44.4	1.56E+05	2.02E+05		2.36E+05	2	2	1	1	0	2	
2594	9716	tr|A0A0A0MR59|A0A0A0MR59_HUMAN	44.4	1.56E+05	2.02E+05		2.36E+05	2	2	1	1	0	2	
2594	9717	sp|Q8TB72|PUM2_HUMAN	44.4	1.56E+05	2.02E+05		2.36E+05	2	2	1	1	0	2	
3001	8289	sp|O15213|WDR46_HUMAN	44.32	6.46E+04	1.07E+06			3	2	1	1	0	0	Acetylatic
3218	12957	sp|Q86SE9|PCGF5_HUMAN	44.29	1.86E+05	1.71E+05			1	1	1	1	0	0	
2915	9259	sp|Q9UJ70|NAGK_HUMAN	44.17	4.42E+04	1.42E+05		5.83E+04	1	1	1	1	0	1	
2915	11232	tr|H7C286|H7C286_HUMAN	44.17	4.42E+04	1.42E+05		5.83E+04	1	1	1	1	0	1	
2915	11233	tr|C9JEV6|C9JEV6_HUMAN	44.17	4.42E+04	1.42E+05		5.83E+04	1	1	1	1	0	1	
3148	11468	tr|F5GX09|F5GX09_HUMAN	44.12	1.43E+06			9.96E+05	1	1	1	0	0	1	
3148	11469	sp|Q5HYJ3|FA76B_HUMAN	44.12	1.43E+06			9.96E+05	1	1	1	0	0	1	
3603	11924	tr|E9PKV8|E9PKV8_HUMAN	44.11	8.37E+05				1	1	1	0	0	0	
3603	11925	sp|Q8N5M4|TTC9C_HUMAN	44.11	8.37E+05				1	1	1	0	0	0	
3603	12958	tr|H0YDR3|H0YDR3_HUMAN	44.11	8.37E+05				1	1	1	0	0	0	
2948	10681	sp|P0CG08|GPHRB_HUMAN	44.08		1.45E+05	2.04E+05	2.80E+05	2	2	0	1	1	1	
2948	10682	sp|B7ZAQ6|GPHRA_HUMAN	44.08		1.45E+05	2.04E+05	2.80E+05	2	2	0	1	1	1	
3026	8169	tr|G3V529|G3V529_HUMAN	44.08			1.53E+06	7.85E+05	4	4	0	0	2	2	
3290	8983	tr|C9K0J5|C9K0J5_HUMAN	43.99	3.73E+05	2.02E+07	1.96E+07	4.06E+07	2	2	1	1	1	1	
3290	9686	tr|C9J164|C9J164_HUMAN	43.99	3.73E+05				1	1	1	0	0	0	
3290	9706	sp|Q70E73|RAPH1_HUMAN	43.99	3.73E+05				1	1	1	0	0	0	
2803	7606	sp|Q15424|SAFB1_HUMAN	43.92	1.43E+06	3.44E+06			5	5	2	4	0	0	Carbamid
3531	9855	sp|Q8WZA1|PMGT1_HUMAN	43.91		7.23E+04			1	1	0	1	0	0	
3039	9047	sp|Q9NVN8|GNL3L_HUMAN	43.74			4.36E+05		2	1	0	0	1	1	
3112	9553	sp|Q6IN85|P4R3A_HUMAN	43.73		1.51E+05	2.41E+04		2	2	0	1	1	0	
3112	11008	tr|G3V5Z3|G3V5Z3_HUMAN	43.73		1.51E+05	2.41E+04		1	1	0	1	1	0	
3112	11693	tr|H0YIY8|H0YIY8_HUMAN	43.73		1.51E+05	2.41E+04		1	1	0	1	1	0	
2751	8591	sp|Q9NRK6|ABCBA_HUMAN	43.69				1.39E+05	2	1	0	0	0	1	
3158	8310	sp|Q9UGU5|HMGX4_HUMAN	43.69		6.40E+05		1.96E+05	3	3	0	2	0	1	
2738	9752	sp|O00151|PDLI1_HUMAN	43.56	6.28E+05		1.22E+05	1.23E+05	2	2	2	0	1	2	Acetylatic
3118	10785	sp|Q9UPY5|XCT_HUMAN	43.51				3.95E+05	2	2	0	0	0	1	
3601	11909	sp|Q9BU64|CENPO_HUMAN	43.49			1.95E+05		1	1	0	0	1	0	
3340	8877	tr|E9PRI1|E9PRI1_HUMAN	43.35				4.96E+05	2	2	0	0	0	1	
3340	8878	sp|Q96NY9|MUS81_HUMAN	43.35				4.96E+05	2	2	0	0	0	1	
3340	8897	tr|H0YDE3|H0YDE3_HUMAN	43.35				4.96E+05	2	2	0	0	0	1	
3447	9910	sp|P38935|SMBP2_HUMAN	43.34					1	1	0	0	0	0	
2936	8563	sp|Q13111|CAF1A_HUMAN	43.27		1.16E+05		5.88E+05	2	2	0	1	0	2	Carbamid
2741	11411	tr|D6RAN8|D6RAN8_HUMAN	43.25		2.02E+05		7.15E+05	1	1	0	1	0	2	Carbamid
2741	11412	tr|H7C5U8|H7C5U8_HUMAN	43.25		2.02E+05		7.15E+05	1	1	0	1	0	2	Carbamid
2741	11413	sp|Q9P0M9|RM27_HUMAN	43.25		2.02E+05		7.15E+05	1	1	0	1	0	2	Carbamid
3072	8752	sp|Q8NBJ4|GOLM1_HUMAN	43		6.42E+05		5.88E+05	3	3	0	1	0	1	
3086	8445	sp|A1L390|PKHG3_HUMAN	42.95					2	2	0	0	0	0	
2889	8656	sp|Q9BUK0|CHCH7_HUMAN	42.92	2.40E+06	3.10E+05			2	2	1	1	0	0	Carbamid
2889	8657	tr|J3KPV1|J3KPV1_HUMAN	42.92	2.40E+06	3.10E+05			2	2	1	1	0	0	Carbamid
3541	10970	sp|Q9NQA3|WASH6_HUMAN	42.92		3.40E+05			2	2	0	2	0	0	
3541	10971	sp|C4AMC7|WASH3_HUMAN	42.92		3.40E+05			2	2	0	2	0	0	
3541	10972	sp|Q6VEQ5|WASH2_HUMAN	42.92		3.40E+05			2	2	0	2	0	0	
3541	10973	sp|A8K0Z3|WASH1_HUMAN	42.92		3.40E+05			2	2	0	2	0	0	
3541	10974	sp|A8MWX3|WASH4_HUMAN	42.92		3.40E+05			2	2	0	2	0	0	
2953	11784	sp|Q6ZRY4|RBPS2_HUMAN	42.89	1.32E+05	1.28E+05			1	1	1	1	0	0	
3094	8934	sp|P21359|NF1_HUMAN	42.81				5.67E+04	2	2	0	0	0	1	
2829	7878	sp|Q9NUQ6|SPS2L_HUMAN	42.68	4.23E+05	4.34E+06	4.58E+05	4.40E+05	4	4	1	2	1	1	Carbamid
2829	8144	tr|B8ZZZ7|B8ZZZ7_HUMAN	42.68	4.23E+05	4.10E+06	4.58E+05	4.40E+05	3	3	1	1	1	1	Carbamid
2855	8458	tr|A0A384DVL6|A0A384DVL6_HUMAN	42.53	6.78E+05				2	2	2	0	0	0	
3166	9018	sp|Q9NZB2|F120A_HUMAN	42.51		5.87E+04			2	2	0	1	0	0	
3166	10835	tr|A0A0C4DG79|A0A0C4DG79_HUMAN	42.51		5.87E+04			2	2	0	1	0	0	
3344	9518	sp|Q9HCG8|CWC22_HUMAN	42.44	1.09E+06				1	1	1	0	0	0	
3185	11675	tr|F6QUN3|F6QUN3_HUMAN	42.35				3.90E+05	1	1	0	0	0	1	Carbamid
3185	11676	tr|H0Y4B0|H0Y4B0_HUMAN	42.35				3.90E+05	1	1	0	0	0	1	Carbamid
3185	11677	tr|F6UGJ8|F6UGJ8_HUMAN	42.35				3.90E+05	1	1	0	0	0	1	Carbamid
3185	11678	tr|A0A3B3ISV9|A0A3B3ISV9_HUMAN	42.35				3.90E+05	1	1	0	0	0	1	Carbamid
3185	11679	sp|O43414|ERI3_HUMAN	42.35				3.90E+05	1	1	0	0	0	1	Carbamid
3132	8625	tr|H7BZE2|H7BZE2_HUMAN	42.25	1.85E+05				2	2	1	0	0	0	Carbamid
3132	8626	sp|Q15654|TRIP6_HUMAN	42.25	1.85E+05				2	2	1	0	0	0	Carbamid
3608	12024	sp|Q96E52|OMA1_HUMAN	42.17					1	1	0	0	0	0	
3608	12963	tr|X6RD49|X6RD49_HUMAN	42.17					1	1	0	0	0	0	
3608	12964	tr|X6RL62|X6RL62_HUMAN	42.17					1	1	0	0	0	0	
3608	12965	tr|X6RIG5|X6RIG5_HUMAN	42.17					1	1	0	0	0	0	
3608	12966	tr|X6RDQ1|X6RDQ1_HUMAN	42.17					1	1	0	0	0	0	
2719	9133	sp|P21953|ODBB_HUMAN	42.12	6.35E+05			7.49E+05	2	2	1	0	0	1	Carbamid
3110	9257	sp|Q9H7N4|SFR19_HUMAN	42.12	1.01E+06	3.72E+05			2	2	3	1	0	0	
3378	9813	sp|Q8NB78|KDM1B_HUMAN	41.87	4.02E+06	3.24E+06	3.12E+06	2.86E+06	2	2	1	1	1	1	
3378	9814	tr|H0Y6H0|H0Y6H0_HUMAN	41.87	4.02E+06	3.24E+06	3.12E+06	2.86E+06	2	2	1	1	1	1	
3153	13011	sp|Q9ULR0|ISY1_HUMAN	41.66			1.60E+06		1	1	0	0	1	0	
3669	13020	sp|Q8N697|S15A4_HUMAN	41.58	1.92E+04				1	1	1	0	0	0	Carbamid
3107	8946	tr|A0A075B6Q2|A0A075B6Q2_HUMAN	41.57			7.69E+04		2	2	0	0	1	0	
3107	8948	sp|Q5T0N5|FBP1L_HUMAN	41.57			7.69E+04		2	2	0	0	1	0	
3107	8949	tr|S4R347|S4R347_HUMAN	41.57			7.69E+04		2	2	0	0	1	0	
3029	8364	tr|A8MT37|A8MT37_HUMAN	41.55		1.57E+06		1.87E+06	3	3	0	2	0	1	
3029	8370	sp|P49840|GSK3A_HUMAN	41.55		1.57E+06		1.87E+06	3	3	0	2	0	1	
3111	9438	tr|A0A0D9SFS3|A0A0D9SFS3_HUMAN	41.52		1.85E+05			2	2	0	1	0	0	
3111	9439	sp|Q02218|ODO1_HUMAN	41.52		1.85E+05			2	2	0	1	0	0	
3111	9440	tr|E9PDF2|E9PDF2_HUMAN	41.52		1.85E+05			2	2	0	1	0	0	
3111	9441	tr|E9PCR7|E9PCR7_HUMAN	41.52		1.85E+05			2	2	0	1	0	0	
3553	11517	sp|O15294|OGT1_HUMAN	41.42					1	1	0	0	0	0	
3604	11927	sp|Q15633|TRBP2_HUMAN	41.3				2.08E+05	1	1	0	0	0	1	Carbamid
3605	11941	sp|Q6PK81|ZN773_HUMAN	41.22			0		1	1	0	0	1	0	
2547	8921	sp|Q9Y2G8|DJC16_HUMAN	41.11	1.25E+05	1.44E+05	1.46E+06		2	2	2	1	2	0	
2547	9122	tr|Q5TDG9|Q5TDG9_HUMAN	41.11	1.25E+05	1.44E+05	1.46E+06		2	2	2	1	2	0	
2547	9123	tr|Q5TDH4|Q5TDH4_HUMAN	41.11	1.25E+05	1.44E+05	1.46E+06		2	2	2	1	2	0	
2967	10731	tr|A0A2R8Y5M6|A0A2R8Y5M6_HUMAN	41.07	1.90E+06				2	2	3	0	0	0	Deamidat
2967	10733	sp|P51572|BAP31_HUMAN	41.07	1.90E+06				2	2	3	0	0	0	Deamidat

2701	9526	sp|Q9NX00|TM160_HUMAN	41.05		0	1.62E+06	2.78E+06	2	2	0	1	1	2	
3404	9846	sp|Q9P0B6|CC167_HUMAN	40.95					1	1	0	0	0	0	
3426	8614	sp|Q9Y2D5|AKAP2_HUMAN	40.76				5.31E+04	2	2	0	0	0	1	
2825	10821	sp|B9A064|IGLL5_HUMAN	40.57			3.34E+08	2.79E+08	1	1	0	0	2	1	
2825	10822	tr|A0A0B4J231|A0A0B4J231_HUMAN	40.57			3.34E+08	2.79E+08	1	1	0	0	2	1	
3323	9702	sp|Q05209|PTN12_HUMAN	40.55		8.62E+04			1	1	0	1	0	0	Carbamid
3323	13044	tr|C9J1X8|C9J1X8_HUMAN	40.55		8.62E+04			1	1	0	1	0	0	Carbamid
3323	13045	tr|F8WB51|F8WB51_HUMAN	40.55		8.62E+04			1	1	0	1	0	0	Carbamid
3586	10907	sp|Q9UNH7|SNX6_HUMAN	40.55					2	2	0	0	0	0	Carbamid
3586	10908	tr|A0A0A0MRI2|A0A0A0MRI2_HUMAN	40.55					2	2	0	0	0	0	Carbamid
2841	8152	sp|Q9H4Z3|CAPAM_HUMAN	40.52	2.29E+05	1.12E+05			3	3	2	1	0	0	Carbamid
2891	8988	tr|A0A087WZ32|A0A087WZ32_HUMAN	40.34	3.68E+05	6.06E+05			2	2	1	1	0	0	
2891	8989	tr|A0A087X038|A0A087X038_HUMAN	40.34	3.68E+05	6.06E+05			2	2	1	1	0	0	
2891	8990	tr|A8K727|A8K727_HUMAN	40.34	3.68E+05	6.06E+05			2	2	1	1	0	0	
2891	9171	sp|Q9HB19|PKHA2_HUMAN	40.34	3.68E+05	6.06E+05			2	2	1	1	0	0	
2859	9594	sp|Q13112|CAF1B_HUMAN	40.29		0		3.50E+05	1	1	0	1	0	1	
3403	9842	tr|A0A075B7G4|A0A075B7G4_HUMAN	40.28		3.85E+05			1	1	0	1	0	0	
3403	9844	sp|Q8IYB9|ZN595_HUMAN	40.28		3.85E+05			1	1	0	1	0	0	
3403	11867	tr|A0A075B7G3|A0A075B7G3_HUMAN	40.28		3.85E+05			1	1	0	1	0	0	
3403	13060	sp|Q05481|ZNF91_HUMAN	40.28		3.85E+05			1	1	0	1	0	0	
3144	10314	sp|Q99570|PI3R4_HUMAN	40.12		9.98E+04			1	1	0	1	0	0	
3179	10923	sp|Q9Y2Y1|RPC10_HUMAN	40.11			2.49E+05	4.03E+05	2	2	0	0	1	1	
3085	8338	sp|Q9C0B0|UNK_HUMAN	40	1.35E+06	5.02E+05			3	3	3	1	0	0	
3181	11141	tr|A0A0J9YWR7|A0A0J9YWR7_HUMAN	39.93			3.82E+06		2	2	0	0	2	0	Deamidat
3181	11142	tr|A0A0J9YXE4|A0A0J9YXE4_HUMAN	39.93			3.82E+06		2	2	0	0	2	0	Deamidat
3181	11143	sp|Q8N2F6|ARM10_HUMAN	39.93			3.82E+06		2	2	0	0	2	0	Deamidat
3539	10864	sp|Q5SNT2|TM201_HUMAN	39.83				3.83E+05	1	1	0	0	0	1	Acetylatio
3187	12095	sp|Q9Y3D6|FIS1_HUMAN	39.78		4.36E+05			1	1	0	1	0	0	
2809	8559	sp|A8MPP1|D11L8_HUMAN	39.77	1.36E+06				2	2	1	0	0	0	
2809	8560	sp|Q96FC9|DDX11_HUMAN	39.77	1.36E+06				2	2	1	0	0	0	
2809	9188	tr|F5H235|F5H235_HUMAN	39.77	1.36E+06				2	2	1	0	0	0	
2809	9189	tr|C9K0E8|C9K0E8_HUMAN	39.77	1.36E+06				2	2	1	0	0	0	
2809	9708	tr|F6WZM0|F6WZM0_HUMAN	39.77	1.36E+06				2	2	1	0	0	0	
3009	8598	sp|Q69YN4|VIR_HUMAN	39.7			9.43E+04		2	2	0	0	1	0	
3009	9313	tr|H0YBN5|H0YBN5_HUMAN	39.7			9.43E+04		2	2	0	0	1	0	
2626	10800	sp|Q8NI60|COQ8A_HUMAN	39.67	5.51E+05	5.55E+05		4.89E+05	2	2	1	1	0	1	
2973	13077	sp|Q8IUX1|T126B_HUMAN	39.56	4.55E+05	2.22E+05		1.75E+05	1	1	1	1	0	1	
2677	8235	tr|F5H4X5|F5H4X5_HUMAN	39.54	5.10E+06	8.84E+06		6.00E+06	3	3	4	3	0	2	Carbamid
2805	8291	tr|F8WE49|F8WE49_HUMAN	39.54	2.31E+05	4.90E+05		1.35E+05	3	3	1	1	0	1	
2805	8292	sp|Q9NS91|RAD18_HUMAN	39.54	2.31E+05	4.90E+05		1.35E+05	3	3	1	1	0	1	
3592	11538	sp|O15169|AXIN1_HUMAN	39.48	3.01E+05				1	1	1	0	0	0	
3138	9311	sp|Q8N3D4|EH1L1_HUMAN	39.42					2	2	0	0	0	0	
3487	9823	tr|A0A0D9SG71|A0A0D9SG71_HUMAN	39.4	1.26E+06	6.54E+05			1	1	1	1	0	0	
3487	9824	tr|A0A0R4J2E5|A0A0R4J2E5_HUMAN	39.4	1.26E+06	6.54E+05			1	1	1	1	0	0	
3487	9825	sp|P49459|UBE2A_HUMAN	39.4	1.26E+06	6.54E+05			1	1	1	1	0	0	
3487	11674	tr|A0A0D9SEZ6|A0A0D9SEZ6_HUMAN	39.4	1.26E+06	6.54E+05			1	1	1	1	0	0	
3147	10949	tr|M0R0M7|M0R0M7_HUMAN	39.31					2	1	0	0	0	0	
3147	10957	sp|Q15833|STXB2_HUMAN	39.31					2	1	0	0	0	0	
3096	9174	sp|Q96S59|RANB9_HUMAN	39.29			6.40E+05		2	2	0	0	2	0	Carbamid
2757	9664	tr|H7C3S9|H7C3S9_HUMAN	39.26		1.07E+06			2	2	0	2	0	0	
2757	9665	tr|E9PGT6|E9PGT6_HUMAN	39.26		1.07E+06			2	2	0	2	0	0	
2757	9666	sp|Q99627|CSN8_HUMAN	39.26		1.07E+06			2	2	0	2	0	0	
3129	8551	sp|Q6ZMI0|PPR21_HUMAN	39.04	1.43E+06				3	3	2	0	0	0	
3117	10784	sp|Q9UIV1|CNOT7_HUMAN	39.04		2.52E+05			2	2	0	1	0	0	
3114	9815	sp|P10606|COX5B_HUMAN	39.02	4.14E+05				1	1	1	0	0	0	
2702	9542	sp|P05091|ALDH2_HUMAN	38.96				7.42E+05	2	1	0	0	1	2	
3003	8762	tr|A0A2U3U055|A0A2U3U055_HUMAN	38.85	3.14E+06				2	1	1	0	0	0	
3003	8765	tr|A0A2R8Y4R4|A0A2R8Y4R4_HUMAN	38.85	3.14E+06				2	1	1	0	0	0	
3003	8766	tr|A0A2U3TZI3|A0A2U3TZI3_HUMAN	38.85	3.14E+06				2	1	1	0	0	0	
3003	8769	sp|Q01814|AT2B2_HUMAN	38.85	3.14E+06				2	1	1	0	0	0	
3003	8770	tr|H0Y7S3|H0Y7S3_HUMAN	38.85	3.14E+06				2	1	1	0	0	0	
3036	8552	sp|Q9UKV0|HDAC9_HUMAN	38.74	3.12E+05			1.79E+05	3	2	1	0	1	1	Deamidat
3036	8555	tr|B7Z3P7|B7Z3P7_HUMAN	38.74	3.12E+05			1.79E+05	3	2	1	0	1	1	Deamidat
3036	8556	tr|B5MCF1|B5MCF1_HUMAN	38.74	3.12E+05			1.79E+05	3	2	1	0	1	1	Deamidat
3176	10824	sp|Q9UK45|LSM7_HUMAN	38.73					2	2	0	0	0	0	Carbamid
2846	10667	tr|F6XHT0|F6XHT0_HUMAN	38.66	4.80E+05		2.51E+06		2	2	1	0	2	0	Deamidat
2846	10668	sp|Q9Y6D0|SELK_HUMAN	38.66	4.80E+05		2.51E+06		2	2	1	0	2	0	Deamidat
2846	11124	tr|F8WAX7|F8WAX7_HUMAN	38.66	4.80E+05		2.51E+06		2	2	1	0	2	0	Deamidat
3120	10962	tr|R4GMW8|R4GMW8_HUMAN	38.64	1.00E+05	1.24E+05			1	1	1	1	0	0	
3120	11946	sp|P28715|ERCC5_HUMAN	38.64	1.00E+05	1.24E+05			1	1	1	1	0	0	
3120	11947	tr|A0A1W2PS85|A0A1W2PS85_HUMAN	38.64	1.00E+05	1.24E+05			1	1	1	1	0	0	
3076	9573	sp|Q9HAU5|RENT2_HUMAN	38.5		7.77E+04			1	1	0	1	0	0	
3106	8874	sp|O75446|SAP30_HUMAN	38.4	5.87E+04	1.48E+05			2	2	1	1	0	0	Carbamid
3451	10887	sp|Q96ST8|CEP89_HUMAN	38.33					1	1	0	0	0	0	
3451	13073	tr|K7EQI2|K7EQI2_HUMAN	38.33					1	1	0	0	0	0	
3451	13074	tr|A0A0C4DGP8|A0A0C4DGP8_HUMAN	38.33					1	1	0	0	0	0	
3451	13075	tr|K7EJF0|K7EJF0_HUMAN	38.33					1	1	0	0	0	0	
2810	9425	sp|Q9P2P6|STAR9_HUMAN	38.32	0	0	1.70E+07	2.05E+07	2	1	1	1	2	1	Deamidat
3490	10051	sp|Q14160|SCRIB_HUMAN	38.25		7.39E+04			1	1	0	1	0	0	
3490	10052	tr|A0A0G2JNZ2|A0A0G2JNZ2_HUMAN	38.25		7.39E+04			1	1	0	1	0	0	
3490	10057	tr|A0A0G2JPP5|A0A0G2JPP5_HUMAN	38.25		7.39E+04			1	1	0	1	0	0	
3490	13089	tr|Q5T0G3|Q5T0G3_HUMAN	38.25		7.39E+04			1	1	0	1	0	0	
3490	13090	sp|Q9BTT6|LRRC1_HUMAN	38.25		7.39E+04			1	1	0	1	0	0	
3123	13078	tr|G5EA44|G5EA44_HUMAN	38.03			2.16E+05		1	1	0	0	1	0	
2844	9043	sp|P36551|HEM6_HUMAN	37.96	2.07E+05	2.30E+05		2.45E+05	1	1	1	1	0	1	
2844	9219	tr|H0YA22|H0YA22_HUMAN	37.96	2.07E+05	2.30E+05		2.45E+05	1	1	1	1	0	1	
2736	9427	tr|F8WCL7|F8WCL7_HUMAN	37.94	1.41E+06	1.53E+06	5.63E+05	9.05E+05	2	2	2	1	1	2	
2736	10933	tr|H7C171|H7C171_HUMAN	37.94	1.41E+06	1.53E+06	5.63E+05	9.05E+05	2	2	2	1	1	2	
2736	10934	tr|C9JKY0|C9JKY0_HUMAN	37.94	1.41E+06	1.53E+06	5.63E+05	9.05E+05	2	2	2	1	1	2	
2736	10935	tr|B8ZZ93|B8ZZ93_HUMAN	37.94	1.41E+06	1.53E+06	5.63E+05	9.05E+05	2	2	2	1	1	2	
2736	10936	tr|B8ZZH7|B8ZZH7_HUMAN	37.94	1.41E+06	1.53E+06	5.63E+05	9.05E+05	2	2	2	1	1	2	
2736	10937	sp|Q9C086|IN80B_HUMAN	37.94	1.41E+06	1.53E+06	5.63E+05	9.05E+05	2	2	2	1	1	2	
2736	10938	tr|J3KQ70|J3KQ70_HUMAN	37.94	1.41E+06	1.53E+06	5.63E+05	9.05E+05	2	2	2	1	1	2	
3095	9145	tr|H3BRY6|H3BRY6_HUMAN	37.76		1.74E+05		1.97E+05	1	1	0	1	0	1	
3095	9146	tr|B4DVT3|B4DVT3_HUMAN	37.76		1.74E+05		1.97E+05	1	1	0	1	0	1	
3095	9149	sp|Q96SY0|INT14_HUMAN	37.76		1.74E+05		1.97E+05	1	1	0	1	0	1	
3095	9150	tr|B4DJL6|B4DJL6_HUMAN	37.76		1.74E+05		1.97E+05	1	1	0	1	0	1	
3554	11716	sp|Q9NW81|DMAC2_HUMAN	37.67					1	1	0	0	0	0	Carbamid
3554	13109	tr|M0QZP7|M0QZP7_HUMAN	37.67					1	1	0	0	0	0	Carbamid
3054	8729	sp|Q8NHQ8|RASF8_HUMAN	37.66					2	2	0	0	0	0	
2968	10854	tr|G5E9Q6|G5E9Q6_HUMAN	37.65	2.15E+05	1.08E+05		1.64E+05	2	2	1	1	0	1	Deamidat
3159	8342	sp|Q9NX05|F120C_HUMAN	37.65	5.49E+05	2.08E+05	9.81E+04		3	3	1	1	1	0	Carbamid
3070	8443	sp|Q9UGU0|TCF20_HUMAN	37.59		2.41E+05		1.21E+05	2	2	0	1	0	1	Carbamid
2781	13107	sp|Q96BR5|COA7_HUMAN	37.53	1.01E+06	8.64E+05			1	1	1	1	0	0	
3670	13096	sp|Q9NXW9|ALKB4_HUMAN	37.51					1	1	0	0	0	0	Carbamid
2685	8693	sp|O95619|YETS4_HUMAN	37.49	1.30E+05	2.92E+05	1.18E+06		2	2	2	1	2	0	
2685	9020	tr|F8W0J4|F8W0J4_HUMAN	37.49	1.30E+05	2.92E+05	1.18E+06		2	2	2	1	2	0	
2895	10715	tr|F8W038|F8W038_HUMAN	37.46	6.39E+05	9.67E+05		1.21E+06	1	1	1	1	0	1	
2895	10716	sp|Q8IXM2|BAP18_HUMAN	37.46	6.39E+05	9.67E+05		1.21E+06	1	1	1	1	0	1	
2895	10717	tr|H0YIS7|H0YIS7_HUMAN	37.46	6.39E+05	9.67E+05		1.21E+06	1	1	1	1	0	1	
2379	9470	sp|Q9Y3D3|RT16_HUMAN	37.37	2.84E+06	3.98E+06	8.41E+06	8.85E+06	2	2	1	1	1	1	
3672	13110	sp|Q6ZVX7|FBX50_HUMAN	37.28					1	1	0	0	0	0	
3671	13098	sp|O95429|BAG4_HUMAN	37.23					1	1	0	0	0	0	
2404	8402	sp|Q14789|GOGB1_HUMAN	37.18		1.83E+05		0	3	2	1	2	1	1	Deamidat
2404	8705	tr|E7EU81|E7EU81_HUMAN	37.18				0	2	1	1	1	1	1	Deamidat
2404	8706	tr|H0Y867|H0Y867_HUMAN	37.18				0	2	1	1	1	1	1	Deamidat

3150	11929	tr|B8ZZS0|B8ZZS0_HUMAN	37.05				4.54E+05	1	1	0	0	0	1	
3150	13133	tr|H7BXT7|H7BXT7_HUMAN	37.05				4.54E+05	1	1	0	0	0	1	
3150	13134	tr|E9PHY4|E9PHY4_HUMAN	37.05				4.54E+05	1	1	0	0	0	1	
3150	13135	tr|A0A0C4DH16|A0A0C4DH16_HUMAN	37.05				4.54E+05	1	1	0	0	0	1	
3150	13136	sp|Q9NYM9|BET1L_HUMAN	37.05				4.54E+05	1	1	0	0	0	1	
2843	8474	sp|Q92997|DVL3_HUMAN	36.93	2.10E+05		3.92E+05	3.34E+05	2	2	1	0	1	1	
2843	9540	tr|I3L2W9|I3L2W9_HUMAN	36.93			3.92E+05	3.34E+05	1	1	0	0	1	1	
2843	9551	tr|I3L2N2|I3L2N2_HUMAN	36.93			3.92E+05	3.34E+05	1	1	0	0	1	1	
2843	9552	sp|O14641|DVL2_HUMAN	36.93			3.92E+05	3.34E+05	1	1	0	0	1	1	
2843	11256	tr|I3L0M7|I3L0M7_HUMAN	36.93			3.92E+05	3.34E+05	1	1	0	0	1	1	
2843	13116	tr|A0A3B3ITC6|A0A3B3ITC6_HUMAN	36.93			3.92E+05	3.34E+05	1	1	0	0	1	1	
3180	10993	sp|Q9HBH5|RDH14_HUMAN	36.84				5.87E+05	2	2	0	0	0	1	
3485	9754	sp|Q9H7H0|MET17_HUMAN	36.77	4.91E+05			4.91E+05	2	2	1	0	0	1	
3178	10914	tr|Q5H9P2|Q5H9P2_HUMAN	36.74	9.28E+05	0	9.43E+05		2	2	1	1	1	0	
3178	10915	sp|P36383|CXG1_HUMAN	36.74	9.28E+05	0	9.43E+05		2	2	1	1	1	0	
3438	9614	sp|Q8WUY9|DEP1B_HUMAN	36.6			2.18E+05		1	1	0	0	1	0	Carbamid
3074	9127	sp|P27708|PYR1_HUMAN	36.58	1.02E+05				2	2	1	0	0	0	
3550	11433	sp|Q6DKK2|TTC19_HUMAN	36.53					1	1	0	0	0	0	
3550	13117	tr|K7ERR1|K7ERR1_HUMAN	36.53					1	1	0	0	0	0	
3550	13118	tr|K7EKH7|K7EKH7_HUMAN	36.53					1	1	0	0	0	0	
2171	9356	tr|J3KPF0|J3KPF0_HUMAN	36.51		2.05E+06	2.50E+06	3.03E+06	2	1	1	2	2	2	Deamidat
3400	9554	sp|P12273|PIP_HUMAN	36.47		3.64E+07	2.01E+05		2	2	0	1	1	0	
2640	13149	tr|E9PNC7|E9PNC7_HUMAN	36.47	5.45E+05	1.05E+06		1.38E+06	1	1	1	2	0	1	
2640	13150	tr|E9PQX9|E9PQX9_HUMAN	36.47	5.45E+05	1.05E+06		1.38E+06	1	1	1	2	0	1	
2640	13151	sp|Q14919|NC2A_HUMAN	36.47	5.45E+05	1.05E+06		1.38E+06	1	1	1	2	0	1	
2640	13152	tr|C9JCC6|C9JCC6_HUMAN	36.47	5.45E+05	1.05E+06		1.38E+06	1	1	1	2	0	1	
2914	9176	sp|Q13011|ECH1_HUMAN	36.41			5.48E+05	1.48E+05	2	2	0	0	1	1	
3211	11017	tr|A8MY26|A8MY26_HUMAN	36.38	7.44E+04				2	2	1	0	0	0	
3211	11018	tr|A8MTY9|A8MTY9_HUMAN	36.38	7.44E+04				2	2	1	0	0	0	
3211	11019	sp|O14972|DSCR3_HUMAN	36.38	7.44E+04				2	2	1	0	0	0	
3600	11822	sp|Q93062|RBPMS_HUMAN	36.31					1	1	0	0	0	0	
3600	11823	tr|B4E3T4|B4E3T4_HUMAN	36.31					1	1	0	0	0	0	
2737	9616	sp|Q9BXW9|FACD2_HUMAN	36.28	1.80E+05	2.04E+05		2.75E+04	2	2	1	1	0	1	Carbamid
2870	9696	sp|Q99549|MPP8_HUMAN	36.18	4.40E+05	4.21E+05	4.68E+05		1	1	1	1	1	0	
3673	13121	tr|E9PNM1|E9PNM1_HUMAN	36.1					1	1	0	0	0	0	
3673	13122	sp|P37268|FDFT_HUMAN	36.1					1	1	0	0	0	0	
3673	13123	tr|A0A1W2PQ47|A0A1W2PQ47_HUMAN	36.1					1	1	0	0	0	0	
2951	11154	sp|Q96NT0|CC115_HUMAN	36.08	2.32E+05			1.66E+05	2	2	1	0	0	1	
2951	13159	tr|B8ZZ99|B8ZZ99_HUMAN	36.08	2.32E+05			1.66E+05	1	1	1	0	0	1	
3443	9782	sp|P10586|PTPRF_HUMAN	36.05		2.07E+04			1	1	0	1	0	0	
3443	13127	tr|A2A437|A2A437_HUMAN	36.05		2.07E+04			1	1	0	1	0	0	
3104	8405	sp|Q6IQ49|SDE2_HUMAN	35.95	4.84E+04	1.46E+06			3	3	1	2	0	0	Carbamid
2782	13162	sp|P41220|RGS2_HUMAN	35.92		2.73E+05	3.49E+05	3.03E+05	1	1	0	1	1	1	Carbamid
3538	10847	sp|Q96JJ7|TMX3_HUMAN	35.88					2	2	0	0	0	0	
3597	11726	sp|P78312|F193A_HUMAN	35.71	1.04E+05				1	1	1	0	0	0	
3597	11727	tr|A0A1B0GVL4|A0A1B0GVL4_HUMAN	35.71	1.04E+05				1	1	1	0	0	0	
3597	13158	tr|E7EUR8|E7EUR8_HUMAN	35.71	1.04E+05				1	1	1	0	0	0	
2950	11074	sp|Q9H0T7|RAB17_HUMAN	35.68				6.74E+04	2	1	0	2	0	1	Carbamid
3479	9444	tr|E9PE82|E9PE82_HUMAN	35.66		3.37E+05			2	2	0	1	0	0	
3479	9445	sp|P16219|ACADS_HUMAN	35.66		3.37E+05			2	2	0	1	0	0	
3005	9748	tr|A0A0D9SG04|A0A0D9SG04_HUMAN	35.61				1.89E+05	1	1	0	0	0	1	
3005	9886	tr|A0A0X1KG75|A0A0X1KG75_HUMAN	35.61				1.89E+05	1	1	0	0	0	1	
3005	9887	sp|Q53SF7|COBL1_HUMAN	35.61				1.89E+05	1	1	0	0	0	1	
3040	9077	sp|Q8TDR0|MIPT3_HUMAN	35.53		4.32E+05		2.81E+05	2	2	0	1	0	1	
3505	11092	sp|Q86VS8|HOOK3_HUMAN	35.22			1.21E+05		1	1	0	0	1	0	
3505	13183	tr|H0YE69|H0YE69_HUMAN	35.22			1.21E+05		1	1	0	0	1	0	
3067	8250	tr|A6NMQ1|A6NMQ1_HUMAN	35.16	8.61E+04		6.18E+05		3	3	1	0	1	0	Carbamid
3430	9185	tr|E7EUN2|E7EUN2_HUMAN	35.12		1.31E+05	2.28E+05		2	2	0	1	1	0	
3430	9499	tr|A0A087X1U1|A0A087X1U1_HUMAN	35.12		1.31E+05	2.28E+05		2	2	0	1	1	0	
3430	9500	tr|F5GXM9|F5GXM9_HUMAN	35.12		1.31E+05	2.28E+05		2	2	0	1	1	0	
3430	9501	sp|Q9UPQ3|AGAP1_HUMAN	35.12		1.31E+05	2.28E+05		2	2	0	1	1	0	
3430	13184	tr|H7C0A7|H7C0A7_HUMAN	35.12			2.28E+05		1	1	0	0	1	0	
3219	13176	sp|O95571|ETHE1_HUMAN	35.07				1.48E+05	1	1	0	0	0	1	Carbamid
3219	13177	tr|M0QXB5|M0QXB5_HUMAN	35.07				1.48E+05	1	1	0	0	0	1	Carbamid
3057	8955	sp|Q03252|LMNB2_HUMAN	34.96					2	1	1	1	0	0	
3361	8724	sp|Q14534|ERG1_HUMAN	34.92	1.35E+05	1.79E+05			2	2	1	1	0	0	
3361	8758	tr|E7EVQ6|E7EVQ6_HUMAN	34.92	1.35E+05	1.79E+05			2	2	1	1	0	0	
3361	9507	tr|H0YBN7|H0YBN7_HUMAN	34.92	1.35E+05	1.79E+05			2	2	1	1	0	0	
3021	8534	sp|Q6UWP7|LCLT1_HUMAN	34.58			8.87E+05	5.86E+05	3	3	0	0	1	1	
2893	9862	tr|G5E9W3|G5E9W3_HUMAN	34.44		4.09E+05			1	1	0	1	0	0	
2893	9863	sp|Q9UKF6|CPSF3_HUMAN	34.44		4.09E+05			1	1	0	1	0	0	
3212	11101	tr|A0A087WZA9|A0A087WZA9_HUMAN	34.34	2.51E+05				2	2	1	0	0	0	
3212	11102	tr|A0A024R4K9|A0A024R4K9_HUMAN	34.34	2.51E+05				2	2	1	0	0	0	
3212	11103	sp|Q9BXJ8|T120A_HUMAN	34.34	2.51E+05				2	2	1	0	0	0	
3212	11104	tr|A0A087X266|A0A087X266_HUMAN	34.34	2.51E+05				2	2	1	0	0	0	
3363	9742	tr|A0A286YFD6|A0A286YFD6_HUMAN	34.26	8.93E+04				1	1	1	0	0	0	
3363	9743	sp|P31350|RIR2_HUMAN	34.26	8.93E+04				1	1	1	0	0	0	
3363	11915	tr|C9JXC1|C9JXC1_HUMAN	34.26	8.93E+04				1	1	1	0	0	0	
3363	11916	tr|A0A0C4DGZ6|A0A0C4DGZ6_HUMAN	34.26	8.93E+04				1	1	1	0	0	0	
3363	11917	sp|Q7LG56|RIR2B_HUMAN	34.26	8.93E+04				1	1	1	0	0	0	
3363	11918	tr|H0YAV1|H0YAV1_HUMAN	34.26	8.93E+04				1	1	1	0	0	0	
3532	9860	sp|Q9P2B7|CFA97_HUMAN	34.21			4.62E+05		1	1	0	0	1	0	
3532	11745	tr|A0A3B3IRJ5|A0A3B3IRJ5_HUMAN	34.21			4.62E+05		1	1	0	0	1	0	
3591	11535	sp|Q86XI2|CNDG2_HUMAN	34.04					1	1	0	0	0	0	
3591	13205	tr|H0Y6U5|H0Y6U5_HUMAN	34.04					1	1	0	0	0	0	
3674	13192	tr|E5RGR9|E5RGR9_HUMAN	34.04	2.19E+05				1	1	1	0	0	0	
3674	13199	tr|E5RHH4|E5RHH4_HUMAN	34.04	2.19E+05				1	1	1	0	0	0	
3674	13200	sp|Q969M3|YIPF5_HUMAN	34.04	2.19E+05				1	1	1	0	0	0	
3676	13202	sp|P00156|CYB_HUMAN	33.9					1	1	0	0	0	0	
3177	10831	sp|P11511|CP19A_HUMAN	33.88		6.47E+04			2	2	0	1	0	0	
3590	11492	tr|D6RBL0|D6RBL0_HUMAN	33.84			1.39E+06		1	1	0	0	1	0	
3529	8571	sp|Q9UBQ0|VPS29_HUMAN	33.84	1.34E+05	8.29E+05			3	3	1	1	0	0	
3529	8572	tr|F8VXU5|F8VXU5_HUMAN	33.84	1.34E+05	8.29E+05			3	3	1	1	0	0	
3529	13195	tr|Q05DG7|Q05DG7_HUMAN	33.84					1	1	0	0	0	0	
3331	9372	sp|Q7Z3T8|ZFY16_HUMAN	33.83			1.35E+05		1	1	0	0	1	0	
3547	11289	tr|A0A0G2JNZ5|A0A0G2JNZ5_HUMAN	33.81		7.70E+05			1	1	0	1	0	0	
3547	11290	tr|A0A0G2JNZ0|A0A0G2JNZ0_HUMAN	33.81		7.70E+05			1	1	0	1	0	0	
3547	11291	sp|P04062|GLCM_HUMAN	33.81		7.70E+05			1	1	0	1	0	0	
3547	11292	tr|A0A0G2JLB3|A0A0G2JLB3_HUMAN	33.81		7.70E+05			1	1	0	1	0	0	
3622	13211	sp|Q9H9T3|ELP3_HUMAN	33.77					1	1	0	0	0	0	
2783	13244	tr|A0A0B4J1T7|A0A0B4J1T7_HUMAN	33.66	1.37E+08	7.29E+07	2.36E+08	9.39E+07	1	1	1	1	1	1	
2823	8489	sp|A8MTT3|CEBOS_HUMAN	33.61	1.28E+06	9.08E+06	0	2.20E+06	3	3	1	3	1	1	
3220	13188	sp|Q9UBV8|PEF1_HUMAN	33.59		6.49E+04			1	1	0	2	0	0	
3362	9422	sp|O75179|ANR17_HUMAN	33.51	1.47E+06				2	2	2	0	0	0	Carbamid
3406	9888	sp|Q96RL7|VP13A_HUMAN	33.47					1	1	0	0	0	0	
3607	12013	tr|A0A024QZ42|A0A024QZ42_HUMAN	33.3					1	1	0	0	0	0	
3607	12014	sp|O75340|PDCD6_HUMAN	33.3					1	1	0	0	0	0	
3675	13197	sp|Q9Y371|SHLB1_HUMAN	33.29					1	1	0	0	0	0	
3675	13198	tr|A0A087WW40|A0A087WW40_HUMAN	33.29					1	1	0	0	0	0	
3598	11728	sp|Q92729|PTPRU_HUMAN	33.27				4.60E+05	1	1	0	0	0	1	
2772	13201	sp|Q9UHA4|LTOR3_HUMAN	33.26	5.45E+05			6.85E+05	1	1	1	0	0	1	
2974	13210	sp|O43617|TPPC3_HUMAN	33.1		7.98E+05			1	1	0	1	0	0	
2974	13213	tr|A0A087WWM0|A0A087WWM0_HUMAN	33.1		7.98E+05			1	1	0	1	0	0	
3533	10427	sp|P34947|GRK5_HUMAN	33.04			4.19E+05		1	1	0	0	1	0	
2539	9818	sp|P35658|NU214_HUMAN	32.82	4.23E+07	6.02E+07	4.71E+07	4.28E+07	2	1	1	1	1	2	
3460	11213	sp|Q76M96|CCD80_HUMAN	32.55			8.88E+05		1	1	0	0	1	0	

2924	11309	sp|Q5VZE5|NAA35_HUMAN	32.52		1.12E+05	1.40E+06		2	2	0	3	1	0	
3678	13241	sp|O75438|NDUB1_HUMAN	32.5					1	1	0	0	0	0	
3548	11348	sp|Q9H1D9|RPC6_HUMAN	32.34	3.56E+05				1	1	1	0	0	0	
3623	13291	tr|Q5T760|Q5T760_HUMAN	32.09	6.25E+04	1.16E+05			1	1	1	1	0	0	
3677	13222	tr|H0YLD0|H0YLD0_HUMAN	31.73		6.24E+05			1	1	0	1	0	0	Carbamid
3677	13223	tr|H0YLY9|H0YLY9_HUMAN	31.73		6.24E+05			1	1	0	1	0	0	Carbamid
3677	13224	sp|Q14442|PIGH_HUMAN	31.73		6.24E+05			1	1	0	1	0	0	Carbamid
3624	13303	tr|H3BSA6|H3BSA6_HUMAN	31.69		9.68E+04			1	1	0	1	0	0	
3624	13305	sp|Q9H773|DCTP1_HUMAN	31.69		9.68E+04			1	1	0	1	0	0	
3519	12097	tr|Q8TD49|Q8TD49_HUMAN	31.65		3.48E+04			1	1	0	1	0	0	
3519	12098	sp|Q9H1A3|METL9_HUMAN	31.65		3.48E+04			1	1	0	1	0	0	
3519	13218	tr|H3BQD2|H3BQD2_HUMAN	31.65		3.48E+04			1	1	0	1	0	0	
3519	13219	tr|H3BM54|H3BM54_HUMAN	31.65		3.48E+04			1	1	0	1	0	0	
3519	13220	tr|H3BN86|H3BN86_HUMAN	31.65		3.48E+04			1	1	0	1	0	0	
3425	8578	sp|Q12899|TRI26_HUMAN	31.57					3	3	0	0	0	0	
3425	10932	tr|A2AE48|A2AE48_HUMAN	31.57					2	2	0	0	0	0	
3425	13310	tr|A0A0G2JIW2|A0A0G2JIW2_HUMAN	31.57					1	1	0	0	0	0	
3613	12198	tr|H7C2T4|H7C2T4_HUMAN	31.47		2.56E+05			1	1	0	1	0	0	
3613	12199	sp|Q7Z698|SPRE2_HUMAN	31.47		2.56E+05			1	1	0	1	0	0	
3613	12200	tr|C9JG63|C9JG63_HUMAN	31.47		2.56E+05			1	1	0	1	0	0	
3452	10922	sp|Q9UNY4|TTF2_HUMAN	31.34					1	1	0	0	0	0	
3182	11253	tr|E9PDE8|E9PDE8_HUMAN	31.34		1.83E+05			1	1	0	1	0	0	
3182	11254	sp|O95757|HS74L_HUMAN	31.34		1.83E+05			1	1	0	1	0	0	
3002	8444	sp|Q93063|EXT2_HUMAN	31.29	1.76E+05	3.91E+04	3.87E+05		5	5	1	1	2	0	
3684	13366	tr|A8MZB2|A8MZB2_HUMAN	31.26			1.90E+05		1	1	0	0	1	0	
3684	13367	sp|P61599|NAA20_HUMAN	31.26			1.90E+05		1	1	0	0	1	0	
3589	11470	sp|Q3KQZ1|S2535_HUMAN	31.23				1.27E+05	1	1	0	0	0	1	
3614	12246	sp|Q9NNZ3|DNJC4_HUMAN	31.2				4.62E+05	1	1	0	0	0	1	
3614	12247	tr|J3KNJ8|J3KNJ8_HUMAN	31.2				4.62E+05	1	1	0	0	0	1	
3614	13324	tr|Q96FY5|Q96FY5_HUMAN	31.2				4.62E+05	1	1	0	0	0	1	
3431	9256	tr|H7C0V9|H7C0V9_HUMAN	31.19					1	1	0	0	0	0	
3431	9265	sp|P05067|A4_HUMAN	31.19					1	1	0	0	0	0	
3549	11389	sp|Q7Z4Q2|HEAT3_HUMAN	31.19					1	1	0	0	0	0	
3221	13292	tr|D6RJF2|D6RJF2_HUMAN	31.15			0		1	1	0	0	1	0	
3221	13300	tr|D6REB4|D6REB4_HUMAN	31.15			0		1	1	0	0	1	0	
3221	13306	sp|Q9H074|PAIP1_HUMAN	31.15			0		1	1	0	0	1	0	
3405	9865	sp|Q9BVQ7|SPA5L_HUMAN	31.04				4.42E+04	1	1	0	0	0	1	
3405	11694	tr|H0YCA5|H0YCA5_HUMAN	31.04				4.42E+04	1	1	0	0	0	1	
3681	13321	sp|Q9Y3B9|RRP15_HUMAN	31					1	1	0	0	0	0	
3434	9399	sp|P25054|APC_HUMAN	30.95		3.21E+04			1	1	0	1	0	0	
3190	12126	sp|Q86U44|MTA70_HUMAN	30.8	4.81E+05	3.39E+05			1	1	1	1	0	0	
3190	13307	tr|F5H6D8|F5H6D8_HUMAN	30.8	4.81E+05	3.39E+05			1	1	1	1	0	0	
3190	13308	tr|B4DTN4|B4DTN4_HUMAN	30.8	4.81E+05	3.39E+05			1	1	1	1	0	0	
3190	13309	tr|H0YFV6|H0YFV6_HUMAN	30.8	4.81E+05	3.39E+05			1	1	1	1	0	0	
2784	13350	sp|O14717|TRDMT_HUMAN	30.64	1.44E+06	1.73E+06	0	3.86E+06	1	1	1	1	1	1	
3594	11578	sp|Q9BVG4|PBDC1_HUMAN	30.61	3.87E+05				1	1	1	0	0	0	
3594	12022	tr|A6NDF3|A6NDF3_HUMAN	30.61	3.87E+05				1	1	1	0	0	0	
3679	13285	tr|D6RAR7|D6RAR7_HUMAN	30.61					1	1	0	0	0	0	Carbamid
3679	13286	tr|D6RJC6|D6RJC6_HUMAN	30.61					1	1	0	0	0	0	Carbamid
3679	13287	tr|A0A087WYP9|A0A087WYP9_HUMAN	30.61					1	1	0	0	0	0	Carbamid
3679	13288	tr|A6NFV8|A6NFV8_HUMAN	30.61					1	1	0	0	0	0	Carbamid
3679	13289	sp|Q96EV8|DTBP1_HUMAN	30.61					1	1	0	0	0	0	Carbamid
3222	13372	sp|Q96RF0|SNX18_HUMAN	30.51	9.13E+05				1	1	2	0	0	0	
3196	13330	sp|Q8NI37|PPTC7_HUMAN	30.35	5.58E+04	1.08E+05			1	1	1	1	0	0	Carbamid
3680	13293	tr|A0A2R8Y4P5|A0A2R8Y4P5_HUMAN	30.28					1	1	0	0	0	0	
3680	13294	tr|Q5SZ82|Q5SZ82_HUMAN	30.28					1	1	0	0	0	0	
3680	13295	tr|A0A087WXU0|A0A087WXU0_HUMAN	30.28					1	1	0	0	0	0	
3680	13296	tr|A0A2R8Y4J4|A0A2R8Y4J4_HUMAN	30.28					1	1	0	0	0	0	
3680	13297	sp|Q9NWS8|RMND1_HUMAN	30.28					1	1	0	0	0	0	
3446	9903	sp|Q8N6T7|SIR6_HUMAN	30.09	4.22E+05				2	2	1	0	0	0	
3446	10968	tr|M0R1F6|M0R1F6_HUMAN	30.09	4.22E+05				2	2	1	0	0	0	
3446	10969	tr|M0QZ09|M0QZ09_HUMAN	30.09	4.22E+05				2	2	1	0	0	0	
3446	12270	tr|M0R1N9|M0R1N9_HUMAN	30.09					1	1	0	0	0	0	
3446	13312	tr|M0QXA0|M0QXA0_HUMAN	30.09					1	1	0	0	0	0	
3682	13346	tr|E5RIR6|E5RIR6_HUMAN	30.08					1	1	0	0	0	0	Carbamid
3682	13347	tr|E7EVN3|E7EVN3_HUMAN	30.08					1	1	0	0	0	0	Carbamid
2644	9226	sp|Q96RW7|HMCN1_HUMAN	30.01					3	1	0	0	2	1	Deamidat
3115	9859	sp|P21796|VDAC1_HUMAN	30.01				3.11E+05	2	1	0	1	0	1	
3188	12102	tr|A0A087WUX6|A0A087WUX6_HUMAN	29.95	2.39E+05				1	1	1	0	0	0	Acetylatic
3188	12103	tr|A0A087WX59|A0A087WX59_HUMAN	29.95	2.39E+05				1	1	1	0	0	0	Acetylatic
3188	13392	sp|Q16186|ADRM1_HUMAN	29.95	2.39E+05				1	1	1	0	0	0	Acetylatic
3453	10964	tr|H7C1I0|H7C1I0_HUMAN	29.95		3.10E+05			2	2	0	1	0	0	
3453	10965	sp|Q96K37|S35E1_HUMAN	29.95		3.10E+05			2	2	0	1	0	0	
3151	12008	sp|P08236|BGLR_HUMAN	29.89	1.74E+05		1.37E+05	9.48E+04	1	1	1	0	1	1	
3151	13425	tr|F8WBK6|F8WBK6_HUMAN	29.89	1.74E+05		1.37E+05	9.48E+04	1	1	1	0	1	1	
3151	13426	tr|F2Z3L6|F2Z3L6_HUMAN	29.89	1.74E+05		1.37E+05	9.48E+04	1	1	1	0	1	1	
3239	8720	tr|G3V5X4|G3V5X4_HUMAN	29.81	6.45E+07	7.71E+07	4.93E+07	7.57E+07	2	2	1	1	1	1	Carbamid
3239	8721	tr|A0A0A0MRE3|A0A0A0MRE3_HUMAN	29.81	6.45E+07	7.71E+07	4.93E+07	7.57E+07	2	2	1	1	1	1	Carbamid
3239	8726	sp|Q8WXH0|SYNE2_HUMAN	29.81	6.45E+07	7.71E+07	4.93E+07	7.57E+07	2	2	1	1	1	1	Carbamid
3428	8789	tr|A0A2R8Y653|A0A2R8Y653_HUMAN	29.81				1.15E+05	2	2	0	0	0	1	
3428	8791	sp|Q9BZX2|UCK2_HUMAN	29.81				1.15E+05	2	2	0	0	0	1	
3068	8368	sp|Q8IUR0|TPPC5_HUMAN	29.77		2.33E+05		1.05E+05	3	3	0	1	0	1	
3068	11191	tr|M0QZQ8|M0QZQ8_HUMAN	29.77				1.05E+05	2	2	0	0	0	1	
3685	13458	sp|Q9Y2Q5|LTOR2_HUMAN	29.73			3.90E+05		1	1	0	0	1	0	Carbamid
3075	9294	sp|Q7L5Y9|MAEA_HUMAN	29.67		2.52E+05			1	1	0	1	0	0	
3075	9296	tr|E7ESC7|E7ESC7_HUMAN	29.67		2.52E+05			1	1	0	1	0	0	
3075	9733	tr|B4DQT1|B4DQT1_HUMAN	29.67		2.52E+05			1	1	0	1	0	0	
3075	9734	tr|B4DVN3|B4DVN3_HUMAN	29.67		2.52E+05			1	1	0	1	0	0	
3075	9843	tr|D6RE80|D6RE80_HUMAN	29.67		2.52E+05			1	1	0	1	0	0	
3224	13502	sp|Q96K12|FACR2_HUMAN	29.61					1	1	0	0	0	0	
3557	12088	tr|A0A0A0MT00|A0A0A0MT00_HUMAN	29.57		4.24E+04			1	1	0	1	0	0	
3557	13382	tr|E9PL11|E9PL11_HUMAN	29.57		4.24E+04			1	1	0	1	0	0	
3557	13383	tr|Q5T6K7|Q5T6K7_HUMAN	29.57		4.24E+04			1	1	0	1	0	0	
3557	13384	tr|E9PI99|E9PI99_HUMAN	29.57		4.24E+04			1	1	0	1	0	0	
3557	13385	tr|Q5T6K5|Q5T6K5_HUMAN	29.57		4.24E+04			1	1	0	1	0	0	
3557	13386	sp|Q13952|NFYC_HUMAN	29.57		4.24E+04			1	1	0	1	0	0	
3146	10790	tr|B8ZZG1|B8ZZG1_HUMAN	29.54		2.76E+05			1	1	0	1	0	0	
3146	10791	sp|Q9NZW5|MPP6_HUMAN	29.54		2.76E+05			1	1	0	1	0	0	
3146	13336	tr|C9J4Q3|C9J4Q3_HUMAN	29.54		2.76E+05			1	1	0	1	0	0	
3568	13470	sp|Q96LW7|CAR19_HUMAN	29.51			2.92E+05		1	1	0	0	1	0	
3183	11440	sp|Q9UK59|DBR1_HUMAN	29.43	2.35E+05			6.61E+05	1	1	1	0	0	1	
3012	9230	sp|Q5VU65|P210L_HUMAN	29.24	8.85E+06	8.95E+06		6.35E+06	2	2	1	1	0	2	
3683	13351	tr|B1AMT4|B1AMT4_HUMAN	29.2					1	1	0	0	0	0	
3683	13352	sp|Q8N3U4|STAG2_HUMAN	29.2					1	1	0	0	0	0	
3625	13399	tr|K7ER12|K7ER12_HUMAN	29.1	0				1	1	1	0	0	0	
3625	13400	tr|K7EQW1|K7EQW1_HUMAN	29.1	0				1	1	1	0	0	0	
3625	13401	tr|A0A087WUU5|A0A087WUU5_HUMAN	29.1	0				1	1	1	0	0	0	
3625	13402	tr|K7ELZ1|K7ELZ1_HUMAN	29.1	0				1	1	1	0	0	0	
3625	13403	sp|Q9UFG5|CS025_HUMAN	29.1	0				1	1	1	0	0	0	
3625	13404	tr|E7EP72|E7EP72_HUMAN	29.1	0				1	1	1	0	0	0	
3625	13405	tr|K7EK56|K7EK56_HUMAN	29.1	0				1	1	1	0	0	0	
3489	9900	tr|E9PPM8|E9PPM8_HUMAN	29.1		6.33E+04			1	1	0	1	0	0	
3489	9901	sp|Q9Y315|DEOC_HUMAN	29.1		6.33E+04			1	1	0	1	0	0	
3489	13389	tr|E9PMH9|E9PMH9_HUMAN	29.1		6.33E+04			1	1	0	1	0	0	
3489	13390	tr|G3V158|G3V158_HUMAN	29.1		6.33E+04			1	1	0	1	0	0	
3495	10720	sp|P11717|MPRI_HUMAN	28.89					1	1	0	0	0	0	

3343	9417	sp|Q9H4L7|SMRCD_HUMAN	28.85			7.81E+05		2	2	0	0	1	0	Carbamid
3155	13368	tr|K7ENF0|K7ENF0_HUMAN	28.8	1.97E+06				1	1	1	0	0	0	
2753	9143	sp|A1X283|SPD2B_HUMAN	28.49	1.22E+06	1.15E+06	1.32E+06	0	2	2	2	1	1	1	
2753	12146	tr|H0Y507|H0Y507_HUMAN	28.49	1.10E+06	1.15E+06	1.32E+06	0	1	1	1	1	1	1	
2753	12150	sp|Q5TCZ1|SPD2A_HUMAN	28.49	1.10E+06	1.15E+06	1.32E+06	0	1	1	1	1	1	1	
3456	11108	sp|O00442|RTCA_HUMAN	28.32				1.76E+05	1	1	0	0	0	1	
2739	9801	sp|O43292|GPAA1_HUMAN	28.25			1.37E+06	8.09E+05	2	2	0	0	2	1	
2739	12141	tr|E9PLG8|E9PLG8_HUMAN	28.25			4.03E+05	8.09E+05	1	1	0	0	1	1	
2739	12142	tr|E9PM11|E9PM11_HUMAN	28.25			4.03E+05	8.09E+05	1	1	0	0	1	1	
2739	12143	tr|E9PLV6|E9PLV6_HUMAN	28.25			4.03E+05	8.09E+05	1	1	0	0	1	1	
2739	12144	tr|E9PPZ9|E9PPZ9_HUMAN	28.25			4.03E+05	8.09E+05	1	1	0	0	1	1	
3480	9460	sp|Q13625|ASPP2_HUMAN	28.04	1.85E+05				2	2	1	0	0	0	
3686	13477	sp|P36405|ARL3_HUMAN	27.97	1.17E+05				1	1	1	0	0	0	
3439	9674	sp|Q9NWB7|IFT57_HUMAN	27.97					1	1	0	0	0	0	Acetylatic
3687	13510	sp|O95365|ZBT7A_HUMAN	27.83				1.21E+05	1	1	0	0	0	1	Carbamid
3486	9819	sp|Q460N5|PAR14_HUMAN	27.81					1	1	0	0	0	0	
2680	11975	sp|O60882|MMP20_HUMAN	27.75			5.89E+06	2.05E+06	1	1	0	0	1	1	
3329	8956	sp|P30622|CLIP1_HUMAN	27.63	8.91E+04				2	1	2	0	0	0	Deamidat
3571	13517	sp|Q9NQG6|MID51_HUMAN	27.59					1	1	0	0	0	0	Carbamid
3571	13518	tr|B0QY95|B0QY95_HUMAN	27.59					1	1	0	0	0	0	Carbamid
3376	9005	tr|H3BR35|H3BR35_HUMAN	27.5	1.32E+05				2	2	1	0	0	0	Carbamid
3376	9006	sp|P15170|ERF3A_HUMAN	27.5	1.32E+05				2	2	1	0	0	0	Carbamid
3197	13546	tr|D6RGK9|D6RGK9_HUMAN	27.48		1.70E+05	1.22E+05		1	1	0	1	1	0	
3197	13547	tr|H0Y9C1|H0Y9C1_HUMAN	27.48		1.70E+05	1.22E+05		1	1	0	1	1	0	
3197	13548	tr|H0Y9Z5|H0Y9Z5_HUMAN	27.48		1.70E+05	1.22E+05		1	1	0	1	1	0	
3197	13549	sp|Q96LI5|CNO6L_HUMAN	27.48		1.70E+05	1.22E+05		1	1	0	1	1	0	
3197	13550	sp|Q9ULM6|CNOT6_HUMAN	27.48		1.70E+05	1.22E+05		1	1	0	1	1	0	
3049	8277	tr|B1AH87|B1AH87_HUMAN	27.42	1.13E+05	1.08E+06	1.74E+06		4	4	1	1	1	0	
3049	8279	sp|P30536|TSPO_HUMAN	27.42	1.13E+05	1.08E+06	1.74E+06		4	4	1	1	1	0	
3441	9762	sp|Q969Q5|RAB24_HUMAN	27.4		5.98E+04			1	1	0	1	0	0	
3441	13465	tr|D6RFW3|D6RFW3_HUMAN	27.4		5.98E+04			1	1	0	1	0	0	
3411	10607	sp|Q8TBC3|SHKB1_HUMAN	27.37					1	1	0	0	0	0	
3477	8694	sp|O75146|HIP1R_HUMAN	27.35	2.53E+05				2	2	1	0	0	0	
2799	10477	tr|M0R127|M0R127_HUMAN	27.23			8.93E+06	9.77E+06	1	1	0	0	1	1	
2799	10827	sp|P21817|RYR1_HUMAN	27.23			8.93E+06	9.77E+06	1	1	0	0	1	1	
3449	10663	sp|Q1MSJ5|CSPP1_HUMAN	27.22	6.90E+04				1	1	1	0	0	0	
3401	9602	tr|B7Z2R7|B7Z2R7_HUMAN	26.99	9.67E+05			1.29E+06	2	2	1	0	0	1	
3401	9603	sp|Q5T8D3|ACBD5_HUMAN	26.99	9.67E+05			1.29E+06	2	2	1	0	0	1	
3688	13532	sp|O00481|BT3A1_HUMAN	26.98					1	1	0	0	0	0	
3615	12319	sp|O94953|KDM4B_HUMAN	26.87			1.34E+05		1	1	0	0	1	0	Carbamid
3615	12320	tr|A0A0C4DFL8|A0A0C4DFL8_HUMAN	26.87			1.34E+05		1	1	0	0	1	0	Carbamid
3615	12322	tr|F5GX28|F5GX28_HUMAN	26.87			1.34E+05		1	1	0	0	1	0	Carbamid
3445	9867	sp|O94876|TMCC1_HUMAN	26.81	3.58E+05				1	1	1	0	0	0	
3445	9905	tr|E9PC87|E9PC87_HUMAN	26.81	3.58E+05				1	1	1	0	0	0	
3556	12066	tr|K4DIB5|K4DIB5_HUMAN	26.73	1.03E+05				1	1	2	0	0	0	
3556	12067	tr|E9PSA8|E9PSA8_HUMAN	26.73	1.03E+05				1	1	2	0	0	0	
3556	12068	tr|E9PRH4|E9PRH4_HUMAN	26.73	1.03E+05				1	1	2	0	0	0	
3556	12069	tr|K4DIA9|K4DIA9_HUMAN	26.73	1.03E+05				1	1	2	0	0	0	
3556	12070	tr|E9PNN7|E9PNN7_HUMAN	26.73	1.03E+05				1	1	2	0	0	0	
3556	12071	tr|E9PNE7|E9PNE7_HUMAN	26.73	1.03E+05				1	1	2	0	0	0	
3556	12072	tr|G3V1B2|G3V1B2_HUMAN	26.73	1.03E+05				1	1	2	0	0	0	
3556	12074	sp|Q96H72|S39AD_HUMAN	26.73	1.03E+05				1	1	2	0	0	0	
3689	13544	tr|H3BM82|H3BM82_HUMAN	26.67	2.10E+05				1	1	1	0	0	0	Carbamid
3689	13545	sp|Q6FIF0|ZFAN6_HUMAN	26.67	2.10E+05				1	1	1	0	0	0	Carbamid
3690	13552	tr|C9J935|C9J935_HUMAN	26.61					1	1	0	0	0	0	
3690	13553	sp|Q8NE22|SETD9_HUMAN	26.61					1	1	0	0	0	0	
3500	10900	sp|P35030|TRY3_HUMAN	26.61			0		1	1	0	0	1	0	
3500	12040	tr|B1AN99|B1AN99_HUMAN	26.61			0		1	1	0	0	1	0	
3079	10981	sp|Q6PIF6|MYO7B_HUMAN	26.52					1	1	0	0	0	0	
3454	11054	tr|H6UMI1|H6UMI1_HUMAN	26.52					2	2	0	0	0	0	
3454	11056	sp|O95166|GBRAP_HUMAN	26.52					2	2	0	0	0	0	
3540	10948	sp|Q9BYD6|RM01_HUMAN	26.41					1	1	0	0	0	0	
3540	12263	tr|H0Y8N7|H0Y8N7_HUMAN	26.41					1	1	0	0	0	0	
3417	11067	tr|A0A0A0MRJ3|A0A0A0MRJ3_HUMAN	26.31	1.20E+05				1	1	1	0	0	0	Deamidat
3417	11069	sp|Q8NEY1|NAV1_HUMAN	26.31	1.20E+05				1	1	1	0	0	0	Deamidat
3506	11135	sp|Q9NXC5|MIO_HUMAN	26.2	1.41E+05				1	1	1	0	0	0	
3198	13604	sp|P63218|GBG5_HUMAN	26.18	1.06E+06				1	1	1	0	0	0	
3616	12331	sp|Q9NY27|PP4R2_HUMAN	26.16	1.45E+05				1	1	1	0	0	0	Carbamid
3692	13589	sp|Q7L8A9|VASH1_HUMAN	25.91					1	1	0	0	0	0	
3507	11287	tr|A0A087X1H5|A0A087X1H5_HUMAN	25.84	6.75E+04				1	1	1	0	0	0	Carbamid
3507	11288	sp|Q15057|ACAP2_HUMAN	25.84	6.75E+04				1	1	1	0	0	0	Carbamid
3507	11392	tr|A0A0U1RQT1|A0A0U1RQT1_HUMAN	25.84	6.75E+04				1	1	1	0	0	0	Carbamid
3507	13651	tr|H7C3Q8|H7C3Q8_HUMAN	25.84	6.75E+04				1	1	1	0	0	0	Carbamid
3461	11379	tr|A0A1W2PRF6|A0A1W2PRF6_HUMAN	25.53	1.41E+04	7.11E+05	1.61E+06		2	2	1	1	1	0	Deamidat
3461	11380	tr|A0A1W2PPX5|A0A1W2PPX5_HUMAN	25.53	1.41E+04	7.11E+05	1.61E+06		2	2	1	1	1	0	Deamidat
3461	11381	tr|A0A1W2PPU6|A0A1W2PPU6_HUMAN	25.53	1.41E+04	7.11E+05	1.61E+06		2	2	1	1	1	0	Deamidat
3461	11382	tr|A0A1W2PQR6|A0A1W2PQR6_HUMAN	25.53	1.41E+04	7.11E+05	1.61E+06		2	2	1	1	1	0	Deamidat
3461	11383	tr|A0A1W2PQB7|A0A1W2PQB7_HUMAN	25.53	1.41E+04	7.11E+05	1.61E+06		2	2	1	1	1	0	Deamidat
3461	11384	tr|A0A1W2PS43|A0A1W2PS43_HUMAN	25.53	1.41E+04	7.11E+05	1.61E+06		2	2	1	1	1	0	Deamidat
3461	11385	sp|Q14108|SCRB2_HUMAN	25.53	1.41E+04	7.11E+05	1.61E+06		2	2	1	1	1	0	Deamidat
3461	11386	tr|A0A1W2PRS1|A0A1W2PRS1_HUMAN	25.53	1.41E+04	7.11E+05	1.61E+06		2	2	1	1	1	0	Deamidat
3226	13632	sp|Q3SXM5|HSDL1_HUMAN	25.28		1.79E+05			1	1	0	1	0	0	
3484	9693	sp|Q9C026|TRIM9_HUMAN	25.18					2	2	0	0	0	0	Deamidat
3488	9837	sp|Q9NUG6|PDRG1_HUMAN	25.17					1	1	0	0	0	0	
3501	10912	tr|A0A0C4DFR1|A0A0C4DFR1_HUMAN	25.06	0				1	1	1	0	0	0	
3502	11004	sp|O14662|STX16_HUMAN	25.03					1	1	0	0	0	0	
3502	12352	tr|H3BU86|H3BU86_HUMAN	25.03					1	1	0	0	0	0	
3306	13622	sp|O43929|ORC4_HUMAN	24.99					1	1	0	0	0	0	
3536	10654	sp|Q9H0C8|ILKAP_HUMAN	24.81		5.05E+04	2.19E+05		2	2	0	1	1	0	
3536	13659	tr|E9PC05|E9PC05_HUMAN	24.81		5.05E+04			1	1	0	1	0	0	
3693	13630	tr|A0A140TA04|A0A140TA04_HUMAN	24.8					1	1	0	0	0	0	Carbamid
2494	11634	tr|A0A087WV45|A0A087WV45_HUMAN	24.69	4.15E+07	3.45E+07	2.60E+07	3.30E+07	1	1	1	1	1	1	
2494	11636	sp|P02766|TTHY_HUMAN	24.69	4.15E+07	3.45E+07	2.60E+07	3.30E+07	1	1	1	1	1	1	
2494	11641	tr|A0A087WT59|A0A087WT59_HUMAN	24.69	4.15E+07	3.45E+07	2.60E+07	3.30E+07	1	1	1	1	1	1	
3630	13684	sp|Q8WUS8|D42E1_HUMAN	24.49		1.23E+06			1	1	0	1	0	0	
3620	13163	sp|P56181|NDUV3_HUMAN	24.29		1.48E+06			1	1	0	1	0	0	
3380	10679	sp|O15061|SYNEM_HUMAN	24.25					1	1	0	0	0	0	
3380	11085	tr|A0A075B7B1|A0A075B7B1_HUMAN	24.25					1	1	0	0	0	0	
3380	12171	tr|H0YL34|H0YL34_HUMAN	24.25					1	1	0	0	0	0	
3307	8328	tr|K4DI95|K4DI95_HUMAN	24.12	1.05E+05				4	2	2	0	0	1	
3423	11690	tr|A0A0A0MRI1|A0A0A0MRI1_HUMAN	24.12		1.86E+05		2.67E+04	1	1	0	1	0	1	
3423	11691	sp|Q8IXQ4|GPAM1_HUMAN	24.12		1.86E+05		2.67E+04	1	1	0	1	0	1	
3493	10616	sp|O95835|LATS1_HUMAN	24.05					1	1	0	0	0	0	Carbamid
3696	13798	sp|Q8TC12|RDH11_HUMAN	23.96	5.43E+04				1	1	1	0	0	0	Carbamid
3697	13807	tr|D6REE2|D6REE2_HUMAN	23.83	8.12E+05				1	1	1	0	0	0	Carbamid
3697	13808	tr|J3KND6|J3KND6_HUMAN	23.83	8.12E+05				1	1	1	0	0	0	Carbamid
3697	13809	sp|Q8NBA8|DTWD2_HUMAN	23.83	8.12E+05				1	1	1	0	0	0	Carbamid
3245	9477	sp|Q8NF91|SYNE1_HUMAN	23.81			6.29E+05	1.24E+05	2	2	0	0	1	1	
3245	9479	tr|E7ENN3|E7ENN3_HUMAN	23.81			6.29E+05	1.24E+05	2	2	0	0	1	1	
3245	9792	tr|A0A0C4DG40|A0A0C4DG40_HUMAN	23.81			6.29E+05		1	1	0	0	1	0	
3702	13950	sp|Q8NEF3|CC112_HUMAN	23.79					1	1	0	0	0	0	
3511	11532	sp|P15104|GLNA_HUMAN	23.76					1	1	0	0	0	0	Carbamid
3511	13523	tr|A0A2R8YDT1|A0A2R8YDT1_HUMAN	23.76					1	1	0	0	0	0	Carbamid
3564	12348	sp|O43739|CYH3_HUMAN	23.48	4.88E+05			3.94E+05	1	1	1	0	0	1	
3565	12392	tr|G3V599|G3V599_HUMAN	23.36			5.59E+05	2.77E+05	1	1	0	0	1	1	
3636	13930	tr|A6NLH6|A6NLH6_HUMAN	23.36				0	1	1	0	0	0	1	

3636	13931	sp|Q9P003|CNIH4_HUMAN	23.36				0	1	1	0	0	0	1	
3510	11530	sp|Q86UX6|ST32C_HUMAN	23.25	1.77E+05				1	1	1	0	0	0	
3435	9517	sp|O95926|SYF2_HUMAN	23.15		4.84E+07			2	2	0	1	0	0	
3699	13887	sp|Q08752|PPID_HUMAN	23.08	5.83E+05				1	1	1	0	0	0	
3695	13753	tr|K7ELW9|K7ELW9_HUMAN	23.03					1	1	0	0	0	0	
3695	13754	sp|Q9NVP2|ASF1B_HUMAN	23.03					1	1	0	0	0	0	
3508	11422	tr|B1AKL4|B1AKL4_HUMAN	22.85		7.91E+04			1	1	0	1	0	0	
3508	11423	sp|Q9NRA8|4ET_HUMAN	22.85		7.91E+04			1	1	0	1	0	0	
3508	13928	tr|B0QYZ7|B0QYZ7_HUMAN	22.85		7.91E+04			1	1	0	1	0	0	
3415	10992	sp|Q7L1Q6|BZW1_HUMAN	22.77					1	1	0	0	0	0	
3429	8838	sp|Q6NZI2|CAVN1_HUMAN	22.69	1.57E+06		4.26E+05	3.98E+05	3	3	1	0	1	1	
3073	9037	sp|Q5TA45|INT11_HUMAN	22.68	3.00E+05	7.89E+04	1.30E+07		3	3	1	1	1	0	
3073	10683	tr|C9J979|C9J979_HUMAN	22.68	3.00E+05	7.89E+04			2	2	1	1	0	0	
3073	10685	tr|A0A087WYI0|A0A087WYI0_HUMAN	22.68	3.00E+05	7.89E+04			2	2	1	1	0	0	
3073	10686	tr|C9IYS7|C9IYS7_HUMAN	22.68	3.00E+05	7.89E+04			2	2	1	1	0	0	
3388	11313	tr|B7Z1U7|B7Z1U7_HUMAN	22.58				4.88E+05	1	1	0	0	0	1	
3388	11314	tr|F8VZG9|F8VZG9_HUMAN	22.58				4.88E+05	1	1	0	0	0	1	
3388	11315	tr|A0A286YEU2|A0A286YEU2_HUMAN	22.58				4.88E+05	1	1	0	0	0	1	
3388	11359	tr|S4R469|S4R469_HUMAN	22.58				4.88E+05	1	1	0	0	0	1	
3388	11360	tr|F8VR27|F8VR27_HUMAN	22.58				4.88E+05	1	1	0	0	0	1	
3388	11361	tr|F8VZY7|F8VZY7_HUMAN	22.58				4.88E+05	1	1	0	0	0	1	
3388	11362	tr|B0QYV1|B0QYV1_HUMAN	22.58				4.88E+05	1	1	0	0	0	1	
3388	11363	tr|B0QYY4|B0QYY4_HUMAN	22.58				4.88E+05	1	1	0	0	0	1	
3388	11364	tr|A0A087X2B1|A0A087X2B1_HUMAN	22.58				4.88E+05	1	1	0	0	0	1	
3388	11365	tr|A0A0G2JRA5|A0A0G2JRA5_HUMAN	22.58				4.88E+05	1	1	0	0	0	1	
3388	11366	sp|O43251|RFOX2_HUMAN	22.58				4.88E+05	1	1	0	0	0	1	
3388	11367	sp|Q9NWB1|RFOX1_HUMAN	22.58				4.88E+05	1	1	0	0	0	1	
3388	11368	tr|J3KNW3|J3KNW3_HUMAN	22.58				4.88E+05	1	1	0	0	0	1	
3388	11478	sp|A6NFN3|RFOX3_HUMAN	22.58				4.88E+05	1	1	0	0	0	1	
3388	11479	tr|A0A3B3ITL7|A0A3B3ITL7_HUMAN	22.58				4.88E+05	1	1	0	0	0	1	
3388	11480	tr|J3QQZ2|J3QQZ2_HUMAN	22.58				4.88E+05	1	1	0	0	0	1	
3388	11481	tr|J3QRF4|J3QRF4_HUMAN	22.58				4.88E+05	1	1	0	0	0	1	
3388	11643	tr|F8VRS4|F8VRS4_HUMAN	22.58				4.88E+05	1	1	0	0	0	1	
3388	11665	tr|A0A0G2JSB3|A0A0G2JSB3_HUMAN	22.58				4.88E+05	1	1	0	0	0	1	
3388	11666	tr|I3L1D4|I3L1D4_HUMAN	22.58				4.88E+05	1	1	0	0	0	1	
3388	11667	tr|B0QYY7|B0QYY7_HUMAN	22.58				4.88E+05	1	1	0	0	0	1	
3388	13992	tr|B0QYY6|B0QYY6_HUMAN	22.58				4.88E+05	1	1	0	0	0	1	
3621	13180	tr|H0Y696|H0Y696_HUMAN	22.55	3.66E+05				1	1	1	0	0	0	
3621	13317	sp|Q96Q42|ALS2_HUMAN	22.55	3.66E+05				1	1	1	0	0	0	
3628	13566	tr|E9PS41|E9PS41_HUMAN	22.39				4.90E+05	1	1	0	0	0	1	
3628	13568	sp|Q6RFH5|WDR74_HUMAN	22.39				4.90E+05	1	1	0	0	0	1	
3704	13981	sp|Q7LBR1|CHM1B_HUMAN	22.32					1	1	0	0	0	0	
3698	13870	tr|Q5T0D2|Q5T0D2_HUMAN	22.15					1	1	0	0	0	0	
3698	13871	sp|P30085|KCY_HUMAN	22.15					1	1	0	0	0	0	
3080	12359	sp|Q9NXH9|TRM1_HUMAN	22.04					1	1	0	0	0	0	
3080	13882	tr|K7EQQ8|K7EQQ8_HUMAN	22.04					1	1	0	0	0	0	
3462	11401	sp|Q8NFC6|BD1L1_HUMAN	22	3.62E+05				1	1	1	0	0	0	
3312	9817	tr|E7ESJ3|E7ESJ3_HUMAN	21.87		1.25E+05			1	1	0	1	0	0	
3457	11137	tr|A0A140T9V2|A0A140T9V2_HUMAN	21.83	1.45E+05				1	1	1	0	0	0	
3457	11144	tr|A0A0G2JK44|A0A0G2JK44_HUMAN	21.83	1.45E+05				1	1	1	0	0	0	
3457	11331	tr|A0A140T9X8|A0A140T9X8_HUMAN	21.83	1.45E+05				1	1	1	0	0	0	
3457	11332	tr|H0Y799|H0Y799_HUMAN	21.83	1.45E+05				1	1	1	0	0	0	
3457	11336	tr|E9PIQ3|E9PIQ3_HUMAN	21.83	1.45E+05				1	1	1	0	0	0	
3457	11338	tr|U3KQA6|U3KQA6_HUMAN	21.83	1.45E+05				1	1	1	0	0	0	
3457	11339	tr|A0A0G2JHL0|A0A0G2JHL0_HUMAN	21.83	1.45E+05				1	1	1	0	0	0	
3457	11340	tr|A0A140T9F7|A0A140T9F7_HUMAN	21.83	1.45E+05				1	1	1	0	0	0	
3457	11342	sp|P25440|BRD2_HUMAN	21.83	1.45E+05				1	1	1	0	0	0	
3457	11343	tr|A0A140T9E9|A0A140T9E9_HUMAN	21.83	1.45E+05				1	1	1	0	0	0	
3457	11344	tr|H0Y6K2|H0Y6K2_HUMAN	21.83	1.45E+05				1	1	1	0	0	0	
3457	12260	tr|E9PKQ9|E9PKQ9_HUMAN	21.83	1.45E+05				1	1	1	0	0	0	
2957	14093	sp|Q8N7C0|LRC52_HUMAN	21.7	3.24E+06	2.34E+06	7.50E+06	7.51E+06	1	1	1	1	1	1	
3409	10494	sp|O43422|P52K_HUMAN	21.62					1	1	0	0	0	0	Carbamid
3516	11910	sp|P14679|TYRO_HUMAN	21.51				0	1	1	0	0	0	1	Carbamid
3471	12047	sp|Q96JG6|VPS50_HUMAN	21.46					1	1	0	0	0	0	Carbamid
3351	10695	sp|Q9HC77|CENPJ_HUMAN	21.44	4.04E+04				1	1	1	0	0	0	
3351	10748	tr|F6VUX8|F6VUX8_HUMAN	21.44	4.04E+04				1	1	1	0	0	0	
3351	13412	tr|H0Y5L8|H0Y5L8_HUMAN	21.44	4.04E+04				1	1	1	0	0	0	
3566	13124	sp|Q6ZP82|CC141_HUMAN	21.4		3.01E+06			1	1	0	1	0	0	Deamidat
3566	13977	tr|E7ERF0|E7ERF0_HUMAN	21.4		3.01E+06			1	1	0	1	0	0	Deamidat
3566	14170	tr|C9JR62|C9JR62_HUMAN	21.4		3.01E+06			1	1	0	1	0	0	Deamidat
3567	13138	sp|Q02413|DSG1_HUMAN	21.4	6.48E+04				1	1	1	0	0	0	
3631	13720	sp|Q9NVI1|FANCI_HUMAN	21.39					1	1	0	0	0	0	
3386	11241	tr|A0A0X1KG73|A0A0X1KG73_HUMAN	21.06			1.73E+05	8.94E+04	1	1	0	0	1	1	
3386	11242	sp|Q15413|RYR3_HUMAN	21.06			1.73E+05	8.94E+04	1	1	0	0	1	1	
3386	11243	tr|A0A087X080|A0A087X080_HUMAN	21.06			1.73E+05	8.94E+04	1	1	0	0	1	1	
3386	11497	tr|A0A0U1RRH1|A0A0U1RRH1_HUMAN	21.06			1.73E+05	8.94E+04	1	1	0	0	1	1	
3413	10850	tr|U3KPY1|U3KPY1_HUMAN	21.03			1.19E+06	1.45E+05	2	2	0	0	1	1	Carbamid
3413	10852	tr|U3KQB0|U3KQB0_HUMAN	21.03			1.19E+06	1.45E+05	2	2	0	0	1	1	Carbamid
3413	10853	sp|P61218|RPAB2_HUMAN	21.03			1.19E+06	1.45E+05	2	2	0	0	1	1	Carbamid
3413	12156	tr|U3KQS8|U3KQS8_HUMAN	21.03			1.19E+06	1.45E+05	2	2	0	0	1	1	Carbamid
3413	12157	tr|F8WC47|F8WC47_HUMAN	21.03			1.19E+06	1.45E+05	2	2	0	0	1	1	Carbamid
3413	12158	tr|B0QYL8|B0QYL8_HUMAN	21.03			1.19E+06	1.45E+05	2	2	0	0	1	1	Carbamid
3413	12159	tr|B0QYL9|B0QYL9_HUMAN	21.03			1.19E+06	1.45E+05	2	2	0	0	1	1	Carbamid
3413	14228	tr|U3KPV3|U3KPV3_HUMAN	21.03				1.45E+05	1	1	0	0	0	1	Carbamid
3385	11168	tr|C9J8U3|C9J8U3_HUMAN	20.94			3.76E+05		1	1	0	0	1	0	
3385	11170	tr|C9JZ40|C9JZ40_HUMAN	20.94			3.76E+05		1	1	0	0	1	0	
3385	11174	sp|Q7Z2E3|APTX_HUMAN	20.94			3.76E+05		1	1	0	0	1	0	
3385	12234	tr|F8W6G5|F8W6G5_HUMAN	20.94			3.76E+05		1	1	0	0	1	0	
3385	13806	tr|A0A0A0MRW7|A0A0A0MRW7_HUMAN	20.94			3.76E+05		1	1	0	0	1	0	
3385	14389	tr|F8WBD6|F8WBD6_HUMAN	20.94			3.76E+05		1	1	0	0	1	0	
3385	14390	tr|E7EUY4|E7EUY4_HUMAN	20.94			3.76E+05		1	1	0	0	1	0	
3385	14391	tr|F8WBM3|F8WBM3_HUMAN	20.94			3.76E+05		1	1	0	0	1	0	
3385	14392	tr|Q6JV79|Q6JV79_HUMAN	20.94			3.76E+05		1	1	0	0	1	0	
3385	14393	tr|E7EVB7|E7EVB7_HUMAN	20.94			3.76E+05		1	1	0	0	1	0	
3638	13966	sp|Q96BR1|SGK3_HUMAN	20.92		9.41E+04			1	1	0	1	0	0	
3470	12021	sp|Q9BZL1|UBL5_HUMAN	20.61		1.38E+06		1.30E+06	2	2	0	1	0	1	Carbamid
3470	14150	tr|K7EQ43|K7EQ43_HUMAN	20.61		1.38E+06			1	1	0	1	0	0	Carbamid
3433	9360	sp|P50995|ANX11_HUMAN	20.52	6.34E+04	4.55E+04			2	2	1	1	0	0	Carbamid
3530	9770	sp|Q13443|ADAM9_HUMAN	20.44	1.45E+05				2	2	1	0	0	0	Carbamid
3530	13569	tr|F8WC54|F8WC54_HUMAN	20.44	1.45E+05				1	1	1	0	0	0	Carbamid
3530	13570	tr|A0AVL1|A0AVL1_HUMAN	20.44	1.45E+05				1	1	1	0	0	0	Carbamid
3509	11428	sp|O75367|H2AY_HUMAN	20.38	6.31E+04				1	1	1	0	0	0	
3509	13387	tr|B4DJC3|B4DJC3_HUMAN	20.38	6.31E+04				1	1	1	0	0	0	
3375	8756	sp|Q5HYI8|RABL3_HUMAN	20.38					3	3	0	0	0	0	Carbamid
3459	11169	tr|A0A1B0GTU3|A0A1B0GTU3_HUMAN	20.28					1	1	0	0	0	0	
3459	11171	sp|Q96L93|KI16B_HUMAN	20.28					1	1	0	0	0	0	
3459	13716	tr|H0Y534|H0Y534_HUMAN	20.28					1	1	0	0	0	0	
3345	9543	sp|Q15007|FL2D_HUMAN	20.25		2.60E+05	9.74E+05		2	2	0	1	1	0	
3637	13943	sp|Q8N8Q8|COX18_HUMAN	20.19			2.21E+05		1	1	0	0	1	0	
3637	13944	tr|B7ZL88|B7ZL88_HUMAN	20.19			2.21E+05		1	1	0	0	1	0	
3561	12223	sp|Q8WXE0|CSKI2_HUMAN	19.96	2.79E+06			1.71E+04	2	2	1	0	0	1	Carbamid
3653	14660	sp|O15055|PER2_HUMAN	19.92				5.66E+06	1	1	0	0	0	1	Deamidat
3572	13539	sp|Q96HB5|CC120_HUMAN	19.91	1.07E+06				1	1	1	0	0	0	
3297	9269	sp|Q92616|GCN1_HUMAN	19.91	3.01E+04				2	2	1	0	0	0	Oxidation
3357	11496	tr|A0A075B6E9|A0A075B6E9_HUMAN	19.9	6.80E+05	2.70E+05			1	1	1	1	0	0	
3292	9676	sp|P20908|CO5A1_HUMAN	19.89					1	1	0	0	0	0	

3518	12090	sp|Q9NTK5|OLA1_HUMAN	19.87					1	1	0	0	0	0	
3518	12091	tr|J3KQ32|J3KQ32_HUMAN	19.87					1	1	0	0	0	0	
3713	14520	sp|Q6SPF0|SAMD1_HUMAN	19.8	1.48E+05				1	1	1	0	0	0	
3442	9777	sp|Q9C0H5|RHG39_HUMAN	19.7			3.77E+05	2.13E+04	2	2	0	0	1	1	
3626	13439	tr|A0A3B3ITB8|A0A3B3ITB8_HUMAN	19.68					1	1	0	0	0	0	
3626	13440	tr|A0A3B3IRQ8|A0A3B3IRQ8_HUMAN	19.68					1	1	0	0	0	0	
3626	13441	tr|A0A3B3ITF6|A0A3B3ITF6_HUMAN	19.68					1	1	0	0	0	0	
3626	13442	tr|A0A3B3IRI2|A0A3B3IRI2_HUMAN	19.68					1	1	0	0	0	0	
3626	13443	sp|P17812|PYRG1_HUMAN	19.68					1	1	0	0	0	0	
3626	14265	tr|A0A3B3ITH9|A0A3B3ITH9_HUMAN	19.68					1	1	0	0	0	0	
3358	11540	sp|Q92796|DLG3_HUMAN	19.49		2.66E+06			1	1	0	1	0	0	
3358	11541	tr|Q5JUW8|Q5JUW8_HUMAN	19.49		2.66E+06			1	1	0	1	0	0	
3392	13130	sp|Q5T4B2|GT253_HUMAN	19.4			3.00E+06	1.73E+06	1	1	0	0	1	1	
3398	8645	sp|Q8TDC3|BRSK1_HUMAN	19.32	4.60E+05				3	2	1	1	0	0	Deamidat
3377	9725	sp|O76024|WFS1_HUMAN	19.3	5.51E+05	2.39E+05			2	2	2	1	0	0	
3520	12361	sp|Q9NNW5|WDR6_HUMAN	19.29		7.59E+04	6.15E+05		2	2	0	2	1	0	Carbamid
3520	12362	tr|A0A087X295|A0A087X295_HUMAN	19.29		7.59E+04	6.15E+05		2	2	0	2	1	0	Carbamid
3396	13746	tr|Q9BTG3|Q9BTG3_HUMAN	19.28			2.69E+07	2.08E+07	1	1	0	0	1	1	Deamidat
3829	11000	sp|Q99996|AKAP9_HUMAN	0					1	1	0	0	0	0	
3923	12184	tr|F2Z2U4|F2Z2U4_HUMAN	0					1	1	0	0	0	0	Carbamid
3936	9904	sp|Q9NSV4|DIAP3_HUMAN	0	1.17E+06				2	1	1	0	0	1	
4118	10769	tr|A0A3F2YNW8|A0A3F2YNW8_HUMAN	0		3.04E+07		2.47E+07	1	1	0	1	0	1	
4131	12216	sp|Q9NR09|BIRC6_HUMAN	0				1.97E+05	1	1	0	0	0	1	
4264	11537	sp|Q709C8|VP13C_HUMAN	0	5.58E+05				1	1	1	0	0	0	
4275	12041	sp|Q9NU22|MDN1_HUMAN	0					1	1	0	0	0	0	
4285	13281	sp|Q8NEV4|MYO3A_HUMAN	0		4.74E+05			1	1	0	1	0	0	
4489	10387	tr|A0A1B0GV47|A0A1B0GV47_HUMAN	0				7.64E+05	1	1	0	0	0	1	
4491	10777	sp|P46734|MP2K3_HUMAN	0	1.93E+05				1	1	1	0	0	0	
4523	13411	tr|C9JG08|C9JG08_HUMAN	0			3.73E+06		1	1	0	0	1	0	
4653	11760	sp|Q8IVF4|DYH10_HUMAN	0		5.46E+05			1	1	0	1	0	0	
4658	11971	tr|F5GYN0|F5GYN0_HUMAN	0					1	1	0	0	0	0	
4668	12315	sp|Q9UPN4|CP131_HUMAN	0					1	1	0	0	0	0	
4682	13448	sp|B2RTY4|MYO9A_HUMAN	0					1	1	0	0	0	0	
4827	11754	tr|B1AMJ5|B1AMJ5_HUMAN	0				0	1	1	0	0	0	1	
4840	12205	tr|F6VDE0|F6VDE0_HUMAN	0			1.00E+06		2	2	0	0	1	0	
4845	12292	tr|A0A2R8YH03|A0A2R8YH03_HUMAN	0		1.04E+06			1	1	0	1	0	0	
4859	13413	sp|Q68DQ2|CRBG3_HUMAN	0			1.37E+06	4.48E+06	1	1	0	0	1	1	
4959	14873	sp|Q9NTG1|PKDRE_HUMAN	0					1	1	0	0	0	0	
5053	9838	tr|A0A2R8Y5N2|A0A2R8Y5N2_HUMAN	0	5.58E+07		9.57E+07	5.46E+07	2	1	1	0	1	2	Deamidat
5097	13460	sp|A2RRP1|NBAS_HUMAN	0					1	1	0	0	0	0	
5379	11576	sp|Q9NS00|C1GLT_HUMAN	0	3.01E+05				1	1	1	0	0	0	
5388	11881	sp|Q2TAA5|ALG11_HUMAN	0					1	1	0	0	0	0	
5400	12280	sp|O95995|DRC4_HUMAN	0	7.60E+06				1	1	1	0	0	0	
5405	12384	sp|Q9BXM0|PRAX_HUMAN	0				7.73E+05	1	1	0	0	0	1	
5410	13280	sp|Q9UPA5|BSN_HUMAN	0	2.08E+07				1	1	1	0	0	0	
5426	13755	sp|Q5THJ4|VP13D_HUMAN	0		2.21E+05			1	1	0	1	0	0	
5430	13822	tr|A0A0A0MRF9|A0A0A0MRF9_HUMAN	0				5.46E+06	1	1	0	0	0	1	
5627	15674	tr|A0A0G2JMG8|A0A0G2JMG8_HUMAN	0					1	1	0	0	0	0	
5777	11570	sp|Q9P2J9|PDP2_HUMAN	0			1.89E+06		1	1	0	0	1	0	
5778	11606	tr|A0A3B3IRY8|A0A3B3IRY8_HUMAN	0	6.45E+05				1	1	1	0	0	0	
5780	11715	sp|Q92817|EVPL_HUMAN	0					1	1	0	0	0	0	
5781	11749	sp|P25189|MYP0_HUMAN	0		2.22E+05	5.49E+05		2	2	0	1	1	0	
5787	12077	sp|Q9H0E9|BRD8_HUMAN	0				5.12E+04	1	1	0	0	0	1	Carbamid
5831	13830	sp|Q9C091|GRB1L_HUMAN	0	1.36E+06			2.75E+05	1	1	1	0	0	1	
5846	14219	tr|A6PVH9|A6PVH9_HUMAN	0	0				1	1	1	0	0	0	
5913	14888	sp|Q9Y2L6|FRM4B_HUMAN	0	9.43E+05			8.77E+05	1	1	1	0	0	1	
5971	15299	tr|J3QR07|J3QR07_HUMAN	0				5.19E+05	1	1	0	0	0	1	
6317	11697	sp|O15066|KIF3B_HUMAN	0					1	1	0	0	0	0	
6323	12257	sp|Q9H8W3|F204A_HUMAN	0				2.33E+05	1	1	0	0	0	1	
6325	12312	sp|Q5T011|SZT2_HUMAN	0					1	1	0	0	0	0	
6326	12314	tr|A0A087X287|A0A087X287_HUMAN	0	2.10E+05				1	1	1	0	0	0	
6327	12339	sp|Q99698|LYST_HUMAN	0					1	1	0	0	0	0	
6331	12386	tr|B4DQU5|B4DQU5_HUMAN	0	3.68E+04				1	1	1	0	0	0	
6333	13320	sp|Q9UG01|IF172_HUMAN	0					1	1	0	0	0	0	Carbamid
6363	13855	tr|F8W8T1|F8W8T1_HUMAN	0					1	1	0	0	0	0	
6405	14399	sp|O43299|AP5Z1_HUMAN	0			1.38E+06	1.95E+06	1	1	0	0	1	2	Deamidat
6411	14436	sp|Q8IUC8|GLT13_HUMAN	0				3.01E+05	1	1	0	0	0	1	Deamidat
6490	15084	sp|Q7L9B9|EEPD1_HUMAN	0					1	1	0	0	0	0	
7096	12351	sp|P62683|GAK21_HUMAN	0		2.40E+05			1	1	0	1	0	0	
7100	13504	sp|P78357|CNTP1_HUMAN	0	1.14E+05				2	2	1	0	0	0	
7101	13648	sp|Q9HCM3|K1549_HUMAN	0	6.88E+05				1	1	1	0	0	0	
7102	13699	sp|O00750|P3C2B_HUMAN	0		4.14E+06			1	1	0	1	0	0	Deamidat
7108	13909	sp|P61956|SUMO2_HUMAN	0		1.42E+05			1	1	0	1	0	0	
7110	13965	sp|Q5FWF4|ZRAB3_HUMAN	0	3.86E+05				1	1	1	0	0	0	
7113	14095	sp|Q5TEU4|NDUF5_HUMAN	0	4.09E+05				1	1	1	0	0	0	
7150	14723	sp|O60477|BRNP1_HUMAN	0				4.14E+06	1	1	0	0	0	1	Deamidat
7168	14856	sp|Q9UMR2|DD19B_HUMAN	0	3.33E+05	1.84E+05			1	1	1	1	0	0	Carbamid
7170	14895	sp|H0YM25|GG6LV_HUMAN	0				4.32E+06	1	1	0	0	0	1	
7277	15824	sp|Q9NZI6|TF2L1_HUMAN	0			5.34E+05		1	1	0	0	1	0	
7334	16123	sp|Q9NQU5|PAK6_HUMAN	0			3.10E+06		1	1	0	0	1	0	Deamidat
8137	13751	tr|A0A0C4DFN8|A0A0C4DFN8_HUMAN	0					1	1	0	0	0	0	Carbamid
8139	13987	sp|Q04721|NOTC2_HUMAN	0					1	1	0	0	0	0	
8141	14045	sp|P31949|S10AB_HUMAN	0					1	1	0	0	0	0	
8145	14167	sp|Q9UKK3|PARP4_HUMAN	0		5.23E+04			1	1	0	1	0	0	Carbamid
8155	14524	sp|P27544|CERS1_HUMAN	0					1	1	0	0	0	0	Acetylatic
8167	14843	tr|Q17RL8|Q17RL8_HUMAN	0					1	1	0	0	0	0	
8176	14972	sp|P98082|DAB2_HUMAN	0	1.01E+06				1	1	1	0	0	0	
8178	15028	sp|Q9BVI0|PHF20_HUMAN	0				4.41E+06	1	1	0	0	0	1	
8195	15398	tr|A0A0G2JR66|A0A0G2JR66_HUMAN	0					1	1	0	0	0	0	
8214	15546	tr|A0A1W2PPB6|A0A1W2PPB6_HUMAN	0				4.94E+06	1	1	0	0	0	1	
8220	15650	sp|Q5MCW4|ZN569_HUMAN	0		6.94E+04			1	1	0	1	0	0	Carbamid
8228	15791	tr|B4DPR9|B4DPR9_HUMAN	0					1	1	0	0	0	0	
8234	15846	sp|Q149N8|SHPRH_HUMAN	0					1	1	0	0	0	0	
9842	14679	tr|J3KR44|J3KR44_HUMAN	0					1	1	0	0	0	0	
9845	14913	tr|F5H4U8|F5H4U8_HUMAN	0		2.89E+05			1	1	0	1	0	0	
9849	15010	sp|Q15776|ZKSC8_HUMAN	0	1.43E+05				1	1	1	0	0	0	Deamidat
9851	15030	tr|E9PRM7|E9PRM7_HUMAN	0					1	1	0	0	0	0	
9857	15239	sp|Q9Y6W5|WASF2_HUMAN	0		2.29E+05			1	1	0	1	0	0	
9873	15836	tr|A0A0D9SFG5|A0A0D9SFG5_HUMAN	0				1.95E+06	1	1	0	0	0	1	
9876	15852	tr|A0A0J9YVP4|A0A0J9YVP4_HUMAN	0		9.27E+05			1	1	0	1	0	0	
9904	16348	tr|K7EPQ7|K7EPQ7_HUMAN	0					1	1	0	0	0	0	
9905	16354	sp|Q5UCC4|EMC10_HUMAN	0					1	1	0	0	0	0	
9928	16594	sp|O95810|CAVN2_HUMAN	0			1.10E+05		1	1	0	0	1	0	
9932	16631	sp|Q9Y2V2|CHSP1_HUMAN	0			2.07E+05		1	1	0	0	1	0	

Table S2 _A3B-RSR pearson correlation

TCGA Tumor	P value	R	TCGA Normal	P value	R	GTEx	P value	R
ACC	1.60E-06	0.52				Adipose-Subcutaneous	0	0.53
BLCA	1.30E-08	0.28	BLCA	5.10E-05	0.79	Adipose-Visceral (Omentum)	2.70E-09	0.41
BRCA	0	0.37	BRCA	0	0.71	Adrenal Gland	0.0016	0.28
CESC	1.40E-05	0.25	CESC	0.51	0.69	Bladder	0.016	0.76
CHOL	0.28	0.19	CHOL	0.098	0.58	Cells-EBV-transformed lymphocytes	0.0061	0.26
COAD	4.60E-09	0.34	COAD	0.27	-0.18	Artery-Aorta	1.00E-05	0.5
DLBC	0.13	0.22				Artery-Coronary	1.70E-06	0.42
ESCA	0.00027	0.27	ESCA	0.0095	0.69	Artery-Tibial	1.30E-07	0.31
GBM	4.60E-05	0.31				Whole Blood	6.20E-15	0.41
HNSC	8.90E-16	0.34	HNSC	0.034	0.32	Cells-Leukemia cell line (CML)	0.12	0.19
KICH	0.0015	0.38	KICH	0.1	0.33	Brain-Amygdala	9.70E-05	0.45
KIRC	0	0.37	KIRC	3.40E-05	0.47	Brain-Anterior cingulate cortex (BA24)		
KIRP	0	0.52	KIRP	0.00048	0.58	Brain-Caudate (basal ganglia)	1.40E-05	0.4
LAML	1.60E-05	0.32				Brain-Cerebellar Hemisphere	0.16	0.14
LGG	0	0.57				Brain-Cerebellum	0.053	0.18
LIHC	6.90E-13	0.36	LIHC	1.60E-07	0.66	Brain-Cortex	0.051	0.19
LUAD	0	0.38	LUAD	0.00072	0.43	Brain-Frontal Cortex (BA9)	0.051	0.19
LUSC	1.30E-08	0.25	LUSC	0.00097	0.45	Brain-Hippocampus	0.011	0.28
MESO	0.003	0.31				Brain-Hypothalamus	4.90E-05	0.43
OV	4.50E-09	0.28				Brain-Nucleus accumbens (basal ganglia)		
PAAD	8.70E-12	0.48	PAAD	0.015	-0.99	Brain-Putamen (basal ganglia)	0.032	0.24
PCPG	1.30E-11	0.47	PCPG	0.15	0.97	Brain-Spinal cord (cervical c-1)	0.0071	0.34
PRAD	0	0.64	PRAD	4.30E-07	0.64	Brain-Substantia nigra	0.0058	0.36
READ	9.10E-06	0.44	READ	0.29	0.37	Breast-Mammary Tissue	0	0.59
SARC	0	0.5	SARC			Cervix-Ectocervix	0.012	0.91
SKCM	0	0.41	SKCM			Cervix-Endocervix	0.53	0.47
STAD	5.90E-11	0.32	STAD	0.0019	0.5	Colon-Sigmoid	0.019	0.2
TGCT	0.00042	0.3				Colon-Transverse	9.80E-05	0.3
THCA	1.10E-07	0.23	THCA	0.038	0.27	Esophagus-Gastroesophageal Junction	7.00E-12	0.54
THYM	0.17	0.13	THYM			Esophagus-Mucosa	0.011	0.15
UCEC	5.70E-07	0.37	UCEC	1.30E-05	0.91	Esophagus-Muscularis	5.90E-05	0.25
UCS	0.34	0.13				Fallopian Tube	0.87	0.1
UVM	1.40E-09	0.62				Heart - Atrial Appendage	0.00027	0.27
						Heart - Left Ventricle	0	0.54
						Kidney - Cortex	0.0024	0.55
						Liver	0	0.69
						Lung	2.20E-16	0.46
						Muscle - Skeletal	0	0.4
						Nerve - Tibial	1.30E-15	0.46
						Ovary	2.00E-05	0.44
						Pancreas	7.10E-05	0.3
						Pituitary	3.00E-04	0.34
						Prostate	3.80E-13	0.65
						Minor Salivary Gland	6.20E-07	0.61
						Cells - Transformed fibroblasts	0	0.84
						Skin - Not Sun Exposed (Suprapubic)	7.80E-14	0.46
						Skin - Sun Exposed (Lower leg)	2.20E-10	0.34
						Small Intestine - Terminal Ileum	1.20E-08	0.55
						Spleen	1.70E-06	0.46
						Stomach	5.00E-06	0.34
						Testis	9.90E-09	0.43
						Thyroid	0.014	0.15
						Uterus	0.0018	0.35
						Vagina	3.00E-05	0.44

Table S3_A3B-survival

TCGA Tumor	P value	R	TCGA Tumor	OS-HR	OS-p(HR)	-log10(p value)	DFS-HR	DFS-p(HR)	-log10(p value)
ACC	1.60E-06	0.52	ACC	8.2	3.70E-05	4.43	5.9	0.0021	2.677780705
BLCA	1.30E-08	0.28	BLCA	0.91	0.51	0.29	1	0.81	0.091514981
BRCA	0	0.37	BRCA	1.1	0.64	0.19	0.94	0.76	0.119186408
CESC	1.40E-05	0.25	CESC	0.46	0.0015	2.82	0.53	0.032	1.494850022
CHOL	0.28	0.19	CHOL	1.7	0.28	0.55	1.2	0.74	0.13076828
COAD	4.60E-09	0.34	COAD	1	0.93	0.03	1	0.89	0.050609993
DLBC	0.13	0.22	DLBC	0.61	0.5	0.30	0.47	0.25	0.602059991
ESCA	0.00027	0.27	ESCA	1.1	0.7	0.15	1.1	0.67	0.173925197
GBM	4.60E-05	0.31	GBM	1.3	0.18	0.74	1.4	0.15	0.823908741
HNSC	8.90E-16	0.34	HNSC	0.95	0.73	0.14	0.86	0.38	0.420216403
KICH	0.0015	0.38	KICH	2.8	0.21	0.68	1.6	0.49	0.30980392
KIRC	0	0.37	KIRC	1.4	0.13	0.89	1.1	0.71	0.148741651
KIRP	0	0.52	KIRP	2	0.1	1.00	2	0.017	1.769551079
LAML	1.60E-05	0.32	LAML	1.1	0.82	0.09	1	NAN	
LGG	0	0.57	LGG	2.8	7.80E-05	4.11	1.6	0.0018	2.744727495
LIHC	6.90E-13	0.36	LIHC	1.4	0.17	0.77	1.4	0.038	1.420216403
LUAD	0	0.38	LUAD	0.91	0.66	0.18	0.94	0.7	0.15490196
LUSC	1.30E-08	0.25	LUSC	0.78	0.21	0.68	0.89	0.51	0.292429824
MESO	0.003	0.31	MESO	1.5	0.28	0.55	1.3	0.35	0.455931956
OV	4.50E-09	0.28	OV	0.78	0.16	0.80	0.98	0.9	0.045757491
PAAD	8.70E-12	0.48	PAAD	1.7	0.092	1.04	1	0.91	0.040958608
PCPG	1.30E-11	0.47	PCPG	2.4	0.46	0.34	1.1	0.89	0.050609993
PRAD	0	0.64	PRAD	2.9	0.36	0.44	1.5	0.068	1.167491087
READ	9.10E-06	0.44	READ	0.68	0.56	0.25	1.2	0.66	0.180456064
SARC	0	0.5	SARC	1.4	0.26	0.59	1.4	0.079	1.102372909
SKCM	0	0.41	SKCM	1.3	0.25	0.60	1.1	0.3	0.522878745
STAD	5.90E-11	0.32	STAD	0.93	0.76	0.12	0.89	0.53	0.27572413
TGCT	0.00042	0.3	TGCT	1.3	0.85	0.07	1.9	0.09	1.045757491
THCA	1.10E-07	0.23	THCA	1.4	0.63	0.20	2.3	0.0085	2.070581074
THYM	0.17	0.13	THYM	1.7	0.53	0.28	1.2	0.7	0.15490196
UCEC	5.70E-07	0.37	UCEC	2.2	0.16	0.80	0.96	0.89	0.050609993
UCS	0.34	0.13	UCS	1.1	0.84	0.08	0.97	0.93	0.031517051
UVM	1.40E-09	0.62	UVM	5.3	0.034	1.47	11	0.0013	2.886056648

Table S4_A3B RNASEH1_survival

SampleID	OS	OS.time	APOBEC3B	RNASEHI	GroupID	Group
TCGA-VD-A8KB-01A	0	1354	1.1506387	3.988008	1	High_High
TCGA-VD-AA8O-01A	1	606	1.355927	4.239264	1	High_High
TCGA-V4-A9E8-01A	1	808	1.0179041	3.881435	1	High_High
TCGA-WC-A884-01A	0	12	1.2328884	3.921876	1	High_High
TCGA-VD-A8KJ-01A	0	1150	1.5097111	3.842699	1	High_High
TCGA-YZ-A982-01A	0	495	1.3780694	3.729894	1	High_High
TCGA-V4-A9EI-01A	1	389	2.1727154	4.234296	1	High_High
TCGA-V4-A9F1-01A	0	938	1.6936691	4.045396	1	High_High
TCGA-VD-A8K8-01A	1	1377	1.2846794	3.744909	1	High_High
TCGA-WC-AA9E-01A	0	838	2.5500601	4.427402	1	High_High
TCGA-WC-A885-01A	0	686	1.0238888	4.053269	1	High_High
TCGA-VD-AA8S-01B	0	427	1.1888236	4.007138	1	High_High
TCGA-VD-A8KK-01A	0	64	1.4020882	4.075775	1	High_High
TCGA-V4-A9E7-01A	1	415	1.9767357	4.57586	1	High_High
TCGA-V4-A9EQ-01A	1	455	1.129418	3.96656	1	High_High
TCGA-WC-A883-01A	1	241	1.5313211	4.518926	1	High_High
TCGA-V3-A9ZX-01A	0	470	1.7503208	3.90142	1	High_High
TCGA-V4-A9EX-01A	1	730	1.4783938	3.884951	1	High_High
TCGA-VD-A8KA-01B	0	1178	1.2736597	3.725124	1	High_High
TCGA-V4-A9F5-01A	0	203	1.0228491	3.933225	1	High_High
TCGA-V4-A9EH-01A	0	670	1.1127673	4.180117	1	High_High
TCGA-V4-A9ES-01A	0	1511	1.2149245	4.099736	1	High_High
TCGA-VD-A8KI-01A	1	1113	1.1486996	4.043807	1	High_High
TCGA-WC-AA9A-01A	1	453	1.7566483	3.97689	1	High_High
TCGA-V4-A9F8-01A	0	597	1.1966768	4.053554	1	High_High
TCGA-VD-AA8P-01A	0	86	1.2937307	3.224962	2	High_Low
TCGA-V4-A9F3-01A	1	873	1.5237773	3.520183	2	High_Low
TCGA-WC-A87U-01A	1	1581	1.19298	3.650565	2	High_Low
TCGA-YZ-A980-01A	0	1862	1.0865873	3.635727	2	High_Low
TCGA-VD-A8KM-01A	0	4	1.4391069	3.356509	2	High_Low
TCGA-V4-A9EM-01A	0	966	1.4020493	3.464816	2	High_Low
TCGA-VD-A8K9-01A	0	1424	1.1380272	3.522648	2	High_Low
TCGA-YZ-A983-01A	0	798	1.0702272	3.476179	2	High_Low
TCGA-VD-A8KD-01A	1	114	1.0618758	3.266591	2	High_Low
TCGA-VD-A8KN-01A	0	68	1.1510139	3.515006	2	High_Low
TCGA-V4-A9EE-01A	1	398	1.718788	3.65683	2	High_Low
TCGA-V4-A9EL-01A	0	982	1.7866991	3.580116	2	High_Low
TCGA-VD-A8KL-01A	0	638	1.0311529	3.315296	2	High_Low
TCGA-V4-A9EU-01A	0	709	0.9976413	3.013314	2	High_Low
TCGA-RZ-AB0B-01A	1	149	1.4638943	3.18581	2	High_Low
TCGA-YZ-A985-01A	0	1184	0.7884502	3.86828	3	Low_High
TCGA-V4-A9F2-01A	0	734	0.4681382	4.602443	3	Low_High
TCGA-WC-A880-01A	0	1258	0.6417034	3.735968	3	Low_High

TCGA-VD-AA8N-01A	0	44	0.9410859	3.909131	3	Low_High
TCGA-V4-A9E9-01A	0	1246	0.5315921	4.052236	3	Low_High
TCGA-VD-AA8M-01A	0	6	0.5941142	3.991651	3	Low_High
TCGA-V4-A9EF-01A	0	1165	0.6778241	3.748644	3	Low_High
TCGA-V4-A9EJ-01A	0	746	0.925692	3.722807	3	Low_High
TCGA-WC-A881-01A	0	1065	0.6274992	3.782684	3	Low_High
TCGA-V4-A9EC-01A	0	743	0.330553	3.91903	3	Low_High
TCGA-VD-A8KG-01A	0	19	0.4540167	4.072723	3	Low_High
TCGA-V4-A9E5-01A	0	2499	0.9830727	4.398308	3	Low_High
TCGA-V4-A9EZ-01A	0	599	0.5242937	4.241551	3	Low_High
TCGA-V4-A9ET-01A	0	1362	0.5936602	4.374229	3	Low_High
TCGA-V4-A9EA-01A	0	1348	0.1849963	4.499987	3	Low_High
TCGA-V4-A9EK-01A	0	1028	0.7481049	3.401302	4	Low_Low
TCGA-WC-A88A-01A	1	82	0.6856284	3.496084	4	Low_Low
TCGA-WC-A882-01A	0	1112	0.4316352	3.66159	4	Low_Low
TCGA-V4-A9ED-01A	0	1078	0.409653	3.63992	4	Low_Low
TCGA-WC-A87W-01A	0	1703	0.5326558	3.485062	4	Low_Low
TCGA-V4-A9EV-01A	1	1269	0.9112807	3.248084	4	Low_Low
TCGA-VD-AA8Q-01A	1	636	0.1298598	3.708374	4	Low_Low
TCGA-VD-A8KH-01A	0	1158	0.9501831	3.572309	4	Low_Low
TCGA-V4-A9F7-01A	0	1256	0.9470465	3.4446	4	Low_Low
TCGA-WC-A87Y-01A	1	1314	0.5240892	3.305233	4	Low_Low
TCGA-V3-A9ZY-01A	0	459	0.3689788	3.70507	4	Low_Low
TCGA-V4-A9F4-01A	0	634	0.9829706	3.436213	4	Low_Low
TCGA-YZ-A984-01A	1	1396	0.8781993	3.190489	4	Low_Low
TCGA-WC-A888-01A	1	575	0.4513828	3.70755	4	Low_Low
TCGA-VD-A8KO-01A	0	795	0.8412151	3.244965	4	Low_Low
TCGA-VD-AA8R-01A	0	6	0.8207525	2.815654	4	Low_Low
TCGA-VD-A8K7-01B	0	1459	0.1447914	2.88204	4	Low_Low
TCGA-VD-A8KE-01A	0	821	0.2903351	3.616951	4	Low_Low
TCGA-V4-A9EY-01A	0	837	0.2344881	3.494849	4	Low_Low
TCGA-VD-A8KF-01A	1	40	0.7789741	2.912602	4	Low_Low
TCGA-VD-AA8T-01A	0	49	0.4123517	2.825763	4	Low_Low
TCGA-V4-A9F0-01A	1	945	0.8624135	3.532682	4	Low_Low
TCGA-V4-A9EW-01A	0	1211	0.9738602	3.324996	4	Low_Low
TCGA-V4-A9EO-01A	0	773	0.4408638	3.152722	4	Low_Low
TCGA-WC-A87T-01A	0	2600	0.3598438	3.377438	4	Low_Low

Table S5_A3B RNASEH1_RSR SigScore

TCGA_ID	APOBEC3B	RNASEH1	RSR SigScore	Group_ID	Group
TCGA-V1-A9OQ-01	5.1639	7.6474	8.5135	1	high_high
TCGA-SU-A7E7-01	4.9787	7.6479	8.522405556	1	high_high
TCGA-KC-A7FE-01	4.8776	7.6483	8.592755556	1	high_high
TCGA-EJ-7315-01	6.633	7.6573	8.905338889	1	high_high
TCGA-KK-A8IL-01	5.6172	7.6581	8.795911111	1	high_high
TCGA-CH-5748-01	5.5047	7.6582	8.897611111	1	high_high
TCGA-TK-A8OK-01	5.1334	7.6618	8.647216667	1	high_high
TCGA-KC-A4BL-01	4.9641	7.6662	8.946394444	1	high_high
TCGA-ZG-A8QZ-01	5.654	7.6701	8.844233333	1	high_high
TCGA-HC-7077-01	5.3913	7.6702	8.6254	1	high_high
TCGA-G9-6348-01	5.4773	7.6702	8.786911111	1	high_high
TCGA-VP-A872-01	6.617	7.6784	8.944783333	1	high_high
TCGA-HC-A8D0-01	5.3485	7.6793	8.866827778	1	high_high
TCGA-CH-5743-01	5.9403	7.6795	8.736583333	1	high_high
TCGA-HC-A6AP-01	5.5142	7.6812	8.830894444	1	high_high
TCGA-EJ-7314-01	5.4392	7.6866	8.649533333	1	high_high
TCGA-YL-A8SI-01	5.2421	7.688	8.609033333	1	high_high
TCGA-J4-AATV-01	5.0491	7.6888	8.5694	1	high_high
TCGA-M7-A724-01	6.7181	7.692	9.316288889	1	high_high
TCGA-HC-A6AL-01	5.6359	7.6988	8.89355	1	high_high
TCGA-EJ-A65B-01	6.0177	7.6999	8.874627778	1	high_high
TCGA-KK-A59Z-01	5.251	7.7073	8.9028	1	high_high
TCGA-HC-8258-01	5.0556	7.7086	8.435022222	1	high_high
TCGA-HC-7817-01	6.3908	7.7119	9.068388889	1	high_high
TCGA-CH-5769-01	7.0139	7.7163	9.467472222	1	high_high
TCGA-V1-A9Z7-01	5.3684	7.7179	9.299794444	1	high_high
TCGA-CH-5792-01	5.3635	7.7201	8.909711111	1	high_high
TCGA-HI-7170-01	5.6112	7.7202	8.860166667	1	high_high
TCGA-EJ-5542-01	5.2118	7.7327	8.736944444	1	high_high
TCGA-G9-6343-01	5.4987	7.733	8.537622222	1	high_high
TCGA-CH-5769-11	7.1059	7.7398	9.45595	1	high_high
TCGA-EJ-5526-01	5.5826	7.7402	8.801255556	1	high_high
TCGA-CH-5794-01	5.9222	7.7451	8.934522222	1	high_high
TCGA-HC-7821-01	4.9639	7.7571	8.551261111	1	high_high
TCGA-EJ-7125-11	5.52	7.7589	8.4802	1	high_high
TCGA-EJ-5516-01	5.0966	7.7609	8.690822222	1	high_high
TCGA-ZG-A9L2-01	6.516	7.7723	9.212094444	1	high_high
TCGA-KC-A7F5-01	5.0815	7.775	9.023616667	1	high_high
TCGA-EJ-5531-01	5.7091	7.7767	9.022305556	1	high_high
TCGA-HC-7079-01	4.9954	7.7768	9.188366667	1	high_high

TCGA-CH-5738-01	5.2441	7.7774	8.644377778	1	high_high
TCGA-G9-6363-01	6.5204	7.7808	9.012922222	1	high_high
TCGA-J9-A52D-01	6.0392	7.7864	8.849316667	1	high_high
TCGA-EJ-A7NN-01	6.6904	7.7866	8.844088889	1	high_high
TCGA-CH-5739-01	4.8689	7.7943	8.556488889	1	high_high
TCGA-G9-A9S7-01	5.0101	7.797	8.882416667	1	high_high
TCGA-EJ-5509-01	5.9312	7.7991	8.89475	1	high_high
TCGA-J4-AATZ-01	4.9214	7.7993	8.614222222	1	high_high
TCGA-HC-7744-01	6.0815	7.7999	9.120588889	1	high_high
TCGA-KK-A8I7-01	5.7634	7.8005	9.065755556	1	high_high
TCGA-M7-A721-01	5.7427	7.8016	8.782977778	1	high_high
TCGA-HC-7213-01	6.6019	7.8092	9.615188889	1	high_high
TCGA-EJ-7327-01	5.0654	7.8141	8.83755	1	high_high
TCGA-G9-6499-11	4.8691	7.8231	8.884405556	1	high_high
TCGA-XQ-A8TA-01	6.9423	7.8273	10.29483889	1	high_high
TCGA-EJ-5521-01	5.4183	7.8316	8.783116667	1	high_high
TCGA-EJ-5501-01	5.719	7.8335	8.84165	1	high_high
TCGA-XK-AAJP-01	6.7586	7.8569	8.969711111	1	high_high
TCGA-HC-7736-01	5.1045	7.8625	8.850872222	1	high_high
TCGA-G9-7510-01	4.8825	7.8674	8.5918	1	high_high
TCGA-EJ-5532-01	5.4387	7.8818	9.014805556	1	high_high
TCGA-EJ-5519-01	5.8969	7.8826	8.914561111	1	high_high
TCGA-KK-A6DY-01	5.1621	7.8864	8.6222	1	high_high
TCGA-ZG-A9LU-01	5.1638	7.9026	9.142533333	1	high_high
TCGA-V1-A9OA-01	4.8392	7.9107	8.779605556	1	high_high
TCGA-HC-7209-01	5.4837	7.9177	8.791155556	1	high_high
TCGA-V1-A8WV-01	6.0931	7.9203	9.046655556	1	high_high
TCGA-EJ-A46F-01	5.4212	7.9216	8.958672222	1	high_high
TCGA-ZG-A9LS-01	4.8759	7.9218	8.650438889	1	high_high
TCGA-KK-A7B1-01	4.8995	7.9234	8.775911111	1	high_high
TCGA-YL-A8SL-01	7.6212	7.9408	9.339822222	1	high_high
TCGA-YL-A9WX-01	4.9302	7.944	8.520016667	1	high_high
TCGA-KK-A8I9-01	5.435	7.9495	8.961233333	1	high_high
TCGA-V1-A8MF-01	5.1418	7.9519	8.821955556	1	high_high
TCGA-HC-7081-01	5.0092	7.9522	8.738977778	1	high_high
TCGA-EJ-5507-01	6.36	7.9531	9.069716667	1	high_high
TCGA-EJ-7783-01	5.079	7.9606	8.771727778	1	high_high
TCGA-CH-5751-01	5.5924	7.9611	10.01152222	1	high_high
TCGA-EJ-7123-01	5.3398	7.9696	8.724622222	1	high_high
TCGA-YL-A9WJ-01	6.0431	7.9702	8.829772222	1	high_high
TCGA-VP-A87C-01	4.932	7.9707	9.151872222	1	high_high
TCGA-EJ-7782-01	5.2173	7.9741	8.817677778	1	high_high
TCGA-ZG-A9L4-01	5.1239	7.9765	9.120666667	1	high_high

TCGA-YL-A8HJ-01	5.2537	7.9797	8.829766667	1	high_high
TCGA-HC-A6AN-01	6.022	7.9802	8.857788889	1	high_high
TCGA-EJ-7794-01	5.0041	7.9809	8.841194444	1	high_high
TCGA-EJ-7782-11	5.4396	7.983	8.674572222	1	high_high
TCGA-EJ-5514-01	5.4517	7.9865	9.358244444	1	high_high
TCGA-YL-A9WL-01	5.3671	7.9871	8.83425	1	high_high
TCGA-V1-A8MJ-01	5.1963	7.9897	8.6093	1	high_high
TCGA-KK-A8IJ-01	5.0585	7.9899	8.464322222	1	high_high
TCGA-EJ-7331-01	5.2464	7.9932	8.595683333	1	high_high
TCGA-VP-AA1N-01	6.2585	7.9949	9.026361111	1	high_high
TCGA-EJ-5508-01	4.9287	7.9969	8.7404	1	high_high
TCGA-V1-A8WW-01	5.516	8.0033	8.882244444	1	high_high
TCGA-HC-A6AO-01	5.4145	8.0182	8.848194444	1	high_high
TCGA-HC-7230-01	5.0574	8.0189	8.609216667	1	high_high
TCGA-CH-5752-01	6.384	8.0252	9.294138889	1	high_high
TCGA-YL-A8HM-01	6.9743	8.0316	9.404161111	1	high_high
TCGA-EJ-5503-01	5.1167	8.035	8.453772222	1	high_high
TCGA-ZG-A9N3-01	7.883	8.0499	10.22139444	1	high_high
TCGA-HC-8216-01	4.8484	8.0508	8.982088889	1	high_high
TCGA-KK-A8IH-01	5.788	8.0529	8.508466667	1	high_high
TCGA-HC-7742-01	5.7822	8.065	9.105211111	1	high_high
TCGA-ZG-A9KY-01	6.4695	8.0687	9.704805556	1	high_high
TCGA-CH-5762-01	5.2115	8.0854	8.913494444	1	high_high
TCGA-EJ-7314-11	4.8707	8.0926	8.660588889	1	high_high
TCGA-4L-AA1F-01	5.8514	8.1154	9.252733333	1	high_high
TCGA-YL-A8SF-01	6.3716	8.117	9.294644444	1	high_high
TCGA-FC-7961-01	6.2104	8.1252	9.3446	1	high_high
TCGA-KK-A8ID-01	4.9859	8.1456	9.038655556	1	high_high
TCGA-EJ-5495-01	5.633	8.1498	9.074944444	1	high_high
TCGA-HC-7233-01	5.9423	8.1501	8.704011111	1	high_high
TCGA-EJ-7788-01	5.9463	8.1525	8.942161111	1	high_high
TCGA-2A-A8W3-01	5.5558	8.1599	9.052672222	1	high_high
TCGA-YL-A8SC-01	6.4832	8.1609	9.019638889	1	high_high
TCGA-V1-A9O9-01	5.5215	8.1657	8.863133333	1	high_high
TCGA-XQ-A8TB-01	5.0658	8.1684	8.683133333	1	high_high
TCGA-KK-A8IB-01	5.6461	8.1729	8.982122222	1	high_high
TCGA-EJ-7785-11	4.8804	8.1738	8.386905556	1	high_high
TCGA-HC-A9TH-01	6.2692	8.1832	9.198455556	1	high_high
TCGA-EJ-7785-01	5.2863	8.1837	8.677316667	1	high_high
TCGA-HC-8256-01	5.3437	8.1845	8.607022222	1	high_high
TCGA-XK-AAIV-01	7.6552	8.1865	9.503855556	1	high_high
TCGA-EJ-5522-01	5.299	8.2045	8.676388889	1	high_high
TCGA-EJ-5525-01	5.039	8.2127	9.254572222	1	high_high

TCGA-YL-A8SP-01	5.1379	8.252	8.793	1	high_high
TCGA-J9-A52E-01	5.7099	8.252	9.650033333	1	high_high
TCGA-EJ-7330-11	5.2382	8.2575	8.689266667	1	high_high
TCGA-KK-A8IK-01	5.7197	8.2588	9.1243	1	high_high
TCGA-YL-A9WY-01	6.159	8.2625	9.347133333	1	high_high
TCGA-YJ-A8SW-01	5.7406	8.2694	9.134116667	1	high_high
TCGA-CH-5761-01	7.9606	8.2736	9.861366667	1	high_high
TCGA-CH-5754-01	7.5298	8.2844	9.668077778	1	high_high
TCGA-YL-A9WH-01	6.8029	8.3002	9.412738889	1	high_high
TCGA-EJ-8469-01	6.4999	8.3012	9.276744444	1	high_high
TCGA-CH-5791-01	5.0897	8.3505	8.708061111	1	high_high
TCGA-EJ-7315-11	5.7908	8.3598	8.863105556	1	high_high
TCGA-EJ-5518-01	5.9711	8.3953	9.227672222	1	high_high
TCGA-CH-5788-01	6.1048	8.3975	9.7563	1	high_high
TCGA-EJ-7781-01	5.7883	8.4024	8.595933333	1	high_high
TCGA-V1-A9OL-01	6.3155	8.4052	9.489083333	1	high_high
TCGA-YL-A8S8-01	6.186	8.4104	9.221405556	1	high_high
TCGA-EJ-7789-01	5.967	8.4173	9.349977778	1	high_high
TCGA-EJ-5524-01	5.1718	8.4527	8.879355556	1	high_high
TCGA-XK-AAIW-01	7.9896	8.4731	9.832888889	1	high_high
TCGA-V1-A9O5-01	6.7758	8.4943	9.548983333	1	high_high
TCGA-KK-A8IC-01	5.9678	8.5058	8.856294444	1	high_high
TCGA-KK-A7B4-01	6.2609	8.6526	9.367733333	1	high_high
TCGA-V1-A9O5-06	7.1312	8.6677	9.867172222	1	high_high
TCGA-KK-A6E1-01	6.3896	9.1318	9.270761111	1	high_high
TCGA-XJ-A83F-01	5.3723	6.5442	8.669177778	2	high_low
TCGA-ZG-A9LM-01	5.5554	6.5825	8.560716667	2	high_low
TCGA-G9-7525-01	4.8722	6.6438	8.852044444	2	high_low
TCGA-G9-6369-01	5.253	6.7565	8.676788889	2	high_low
TCGA-2A-A8VX-01	5.4702	6.7658	9.067238889	2	high_low
TCGA-V1-A8MM-01	5.0822	6.774	8.773488889	2	high_low
TCGA-M7-A722-01	6.0271	6.8116	9.043338889	2	high_low
TCGA-EJ-A6RA-01	4.9843	6.8714	8.613566667	2	high_low
TCGA-J4-A67Q-01	4.8615	6.9113	8.540461111	2	high_low
TCGA-G9-A9S4-01	5.8787	6.9488	9.2143	2	high_low
TCGA-HC-A6HY-01	4.9379	6.9599	8.564572222	2	high_low
TCGA-HC-A76W-01	4.9413	7.0143	8.78465	2	high_low
TCGA-J9-A52C-01	4.8313	7.0423	8.492538889	2	high_low
TCGA-G9-6356-11	5.5104	7.0433	8.469338889	2	high_low
TCGA-KK-A6E7-01	5.3862	7.0533	9.174594444	2	high_low
TCGA-KK-A59Y-01	5.1078	7.0611	8.5422	2	high_low
TCGA-EJ-A46G-01	5.5099	7.1081	9.271027778	2	high_low
TCGA-ZG-A9L5-01	5.3608	7.1092	8.964966667	2	high_low

TCGA-V1-A9OX-01	5.4681	7.112	8.661405556	2	high_low
TCGA-ZG-A9M4-01	4.9496	7.1231	8.532888889	2	high_low
TCGA-QU-A6IM-01	5.2031	7.1244	8.449227778	2	high_low
TCGA-H9-A6BY-01	5.3627	7.1759	8.91995	2	high_low
TCGA-VN-A88N-01	5.8798	7.1768	8.768316667	2	high_low
TCGA-ZG-A9NI-01	6.1321	7.2123	9.196922222	2	high_low
TCGA-G9-6356-01	5.2346	7.2162	8.800377778	2	high_low
TCGA-EJ-A7NM-01	5.71	7.2212	9.1175	2	high_low
TCGA-EJ-8474-01	5.5451	7.2229	8.737933333	2	high_low
TCGA-J9-A8CN-01	5.0375	7.2304	8.517416667	2	high_low
TCGA-ZG-A8QX-01	5.2266	7.2379	8.630383333	2	high_low
TCGA-XJ-A83G-01	5.2398	7.2443	8.807727778	2	high_low
TCGA-J4-A67L-01	5.6407	7.2479	8.817672222	2	high_low
TCGA-VP-A876-01	6.2102	7.2678	8.9854	2	high_low
TCGA-KC-A4BV-01	5.6074	7.2749	8.77755	2	high_low
TCGA-KK-A5A1-01	4.941	7.2765	9.461433333	2	high_low
TCGA-YL-A8SH-01	5.7627	7.2792	8.867872222	2	high_low
TCGA-KK-A6E0-01	4.8995	7.2952	9.032211111	2	high_low
TCGA-CH-5750-01	5.1903	7.3165	9.303066667	2	high_low
TCGA-J9-A8CL-01	5.9658	7.3186	9.334605556	2	high_low
TCGA-EJ-A65D-01	6.9693	7.3221	9.102766667	2	high_low
TCGA-G9-6363-11	6.8869	7.3257	9.042055556	2	high_low
TCGA-ZG-A8QY-01	5.1854	7.3279	9.060833333	2	high_low
TCGA-XK-AAJU-01	4.8568	7.3341	8.311044444	2	high_low
TCGA-EJ-A65E-01	5.8157	7.336	8.949105556	2	high_low
TCGA-EJ-A6RC-01	5.6421	7.3406	8.75005	2	high_low
TCGA-KK-A7AQ-01	5.2004	7.3419	9.069116667	2	high_low
TCGA-YL-A9WI-01	6.5641	7.3472	9.491533333	2	high_low
TCGA-HC-A6AS-01	5.7017	7.3555	8.600577778	2	high_low
TCGA-V1-A9Z8-01	5.8794	7.3557	9.195605556	2	high_low
TCGA-G9-6336-01	5.3384	7.3611	8.537544444	2	high_low
TCGA-V1-A8WL-01	4.8995	7.3616	8.44205	2	high_low
TCGA-G9-7523-01	5.4356	7.3672	8.551477778	2	high_low
TCGA-HC-A76X-01	4.943	7.3705	8.782438889	2	high_low
TCGA-J9-A52B-01	5.74	7.3726	9.028233333	2	high_low
TCGA-G9-6370-01	5.0211	7.3731	8.460777778	2	high_low
TCGA-EJ-A7NF-01	5.3328	7.3748	8.5773	2	high_low
TCGA-G9-6496-01	5.9557	7.377	9.273205556	2	high_low
TCGA-V1-A9Z9-01	5.4492	7.3783	8.636472222	2	high_low
TCGA-EJ-A46I-01	4.9762	7.3952	8.447138889	2	high_low
TCGA-KK-A6E6-01	5.5523	7.4004	8.661477778	2	high_low
TCGA-G9-6365-01	5.3735	7.4038	8.857127778	2	high_low
TCGA-Y6-A8TL-01	4.9163	7.4043	8.632772222	2	high_low

TCGA-EJ-5494-01	5.1279	7.413	8.863633333	2	high_low
TCGA-EJ-5505-01	5.1555	7.4209	8.566088889	2	high_low
TCGA-V1-A8MU-01	5.4656	7.4211	8.810538889	2	high_low
TCGA-VP-A878-01	5.4018	7.4232	8.73875	2	high_low
TCGA-KK-A59X-01	6.7341	7.4321	9.161127778	2	high_low
TCGA-EJ-8472-01	6.2962	7.4356	8.928133333	2	high_low
TCGA-ZG-A9L9-01	6.5633	7.4364	9.236538889	2	high_low
TCGA-EJ-5517-01	5.0357	7.4418	8.593194444	2	high_low
TCGA-HC-7752-11	5.0169	7.4451	8.69815	2	high_low
TCGA-TP-A8TV-01	5.4533	7.4492	8.649372222	2	high_low
TCGA-EJ-7318-01	5.647	7.4502	8.75225	2	high_low
TCGA-J4-A6G1-01	5.7732	7.4516	9.391255556	2	high_low
TCGA-J9-A8CM-01	5.2238	7.4609	9.042161111	2	high_low
TCGA-EJ-5504-01	5.6088	7.4616	8.805472222	2	high_low
TCGA-J4-A83J-01	6.2128	7.47	8.848905556	2	high_low
TCGA-ZG-A9LN-01	7.0244	7.4789	9.430188889	2	high_low
TCGA-KC-A7F3-01	5.1693	7.4954	8.624461111	2	high_low
TCGA-EJ-A8FS-01	5.0832	7.4969	8.639672222	2	high_low
TCGA-XK-AAK1-01	5.4227	7.4994	8.37955	2	high_low
TCGA-FC-A5OB-01	5.1251	7.5061	8.574188889	2	high_low
TCGA-KK-A7AZ-01	5.2373	7.5094	8.492144444	2	high_low
TCGA-HC-8261-01	5.5775	7.5131	8.753111111	2	high_low
TCGA-ZG-A9L0-01	6.412	7.5164	9.249172222	2	high_low
TCGA-EJ-5499-01	6.2668	7.5169	9.304733333	2	high_low
TCGA-KK-A59V-01	7.2501	7.5224	9.804422222	2	high_low
TCGA-HC-7737-01	5.5816	7.5231	8.632777778	2	high_low
TCGA-HC-7750-01	5.7931	7.5343	8.622694444	2	high_low
TCGA-FC-A6HD-01	6.4563	7.5427	9.1033	2	high_low
TCGA-EJ-AB27-01	5.3222	7.5429	8.702444444	2	high_low
TCGA-EJ-A65M-01	5.8762	7.5493	9.100938889	2	high_low
TCGA-EJ-7218-01	5.551	7.5546	8.468472222	2	high_low
TCGA-VP-A87H-01	5.4794	7.5559	8.93205	2	high_low
TCGA-CH-5744-01	5.6239	7.5582	8.687422222	2	high_low
TCGA-X4-A8KQ-01	5.3553	7.5643	8.833677778	2	high_low
TCGA-G9-A9S0-01	5.9505	7.5687	9.220222222	2	high_low
TCGA-KK-A7B0-01	6.2348	7.5801	8.779188889	2	high_low
TCGA-HC-7738-01	5.6747	7.5819	8.82305	2	high_low
TCGA-VN-A88O-01	5.325	7.5837	8.554805556	2	high_low
TCGA-EJ-7793-01	4.8965	7.5838	8.422111111	2	high_low
TCGA-KK-A7AP-01	4.8407	7.5846	8.481727778	2	high_low
TCGA-EJ-7115-01	4.8646	7.5864	8.695822222	2	high_low
TCGA-EJ-7125-01	6.1148	7.5868	8.842355556	2	high_low
TCGA-XJ-A9DI-01	4.8431	7.5901	8.817466667	2	high_low

TCGA-ZG-A9LZ-01	5.2992	7.5915	8.957627778	2	high_low
TCGA-KK-A8IF-01	6.2175	7.5961	8.622388889	2	high_low
TCGA-HC-A631-01	6.6519	7.5974	9.0722	2	high_low
TCGA-J4-A6G3-01	6.7637	7.598	9.354794444	2	high_low
TCGA-YL-A8HO-01	5.9763	7.5986	9.145677778	2	high_low
TCGA-CH-5767-11	5.4132	7.6006	8.906377778	2	high_low
TCGA-HC-8266-01	5.8349	7.6024	8.875894444	2	high_low
TCGA-EJ-8470-01	6.0414	7.6029	9.040044444	2	high_low
TCGA-ZG-A9MC-01	6.2206	7.6088	8.693155556	2	high_low
TCGA-YL-A8SO-01	5.7918	7.6162	9.150244444	2	high_low
TCGA-HC-8262-01	4.9039	7.6172	8.71215	2	high_low
TCGA-KK-A6E4-01	4.9512	7.6183	8.813838889	2	high_low
TCGA-ZG-A8QW-01	5.445	7.6193	9.347183333	2	high_low
TCGA-ZG-A9LB-01	5.6989	7.6325	9.509016667	2	high_low
TCGA-V1-A8WN-01	4.8502	7.6351	8.399105556	2	high_low
TCGA-J4-A67K-01	5.1853	7.6381	8.473666667	2	high_low
TCGA-XK-AAJR-01	5.7065	7.6382	8.770761111	2	high_low
TCGA-G9-6329-01	5.3169	7.6408	8.862022222	2	high_low
TCGA-EJ-5510-01	5.085	7.6433	8.540427778	2	high_low
TCGA-CH-5761-11	4.9645	7.6453	8.353605556	2	high_low
TCGA-YL-A8SJ-01	3.5438	7.6575	9.067361111	3	low_high
TCGA-EJ-A8FO-01	4.6643	7.6598	8.485838889	3	low_high
TCGA-J4-A6M7-01	3.8536	7.6616	8.601894444	3	low_high
TCGA-J4-A67T-01	3.1923	7.6639	8.343922222	3	low_high
TCGA-J4-8200-01	4.7077	7.6718	8.529133333	3	low_high
TCGA-CH-5771-01	4.2528	7.6813	8.701227778	3	low_high
TCGA-YL-A8SK-01	4.7076	7.683	8.621055556	3	low_high
TCGA-G9-6377-01	4.5767	7.684	8.628566667	3	low_high
TCGA-HC-7210-01	3.1818	7.6871	8.514322222	3	low_high
TCGA-EJ-7328-01	3.4073	7.6873	8.643338889	3	low_high
TCGA-EJ-5506-01	3.5726	7.6896	8.2988	3	low_high
TCGA-KK-A6E8-01	4.4006	7.7056	8.6435	3	low_high
TCGA-G9-6366-01	3.7512	7.7061	8.474616667	3	low_high
TCGA-KK-A8I5-01	3.7384	7.7093	8.492438889	3	low_high
TCGA-EJ-5496-01	4.4642	7.7097	8.485227778	3	low_high
TCGA-EJ-5515-01	3.6226	7.7108	8.464338889	3	low_high
TCGA-VN-A88P-01	4.666	7.7155	8.358966667	3	low_high
TCGA-VP-A87E-01	4.5716	7.7181	8.460944444	3	low_high
TCGA-CH-5746-01	4.0666	7.7201	8.754461111	3	low_high
TCGA-ZG-A9L6-01	4.3599	7.7276	8.544438889	3	low_high
TCGA-V1-A8X3-01	4.8183	7.7321	8.6059	3	low_high
TCGA-EJ-7791-01	4.5758	7.7325	8.8854	3	low_high
TCGA-TP-A8TT-01	4.809	7.7389	8.388211111	3	low_high

TCGA-YL-A8SR-01	3.6594	7.7415	8.595394444	3	low_high
TCGA-HC-8262-11	4.1279	7.7583	8.580961111	3	low_high
TCGA-VP-A879-01	3.5276	7.7594	8.08955	3	low_high
TCGA-HC-7819-11	4.186	7.765	8.329955556	3	low_high
TCGA-CH-5764-01	4.4722	7.7671	8.647994444	3	low_high
TCGA-J4-A83K-01	4.063	7.7672	8.508177778	3	low_high
TCGA-YL-A8SQ-01	4.2159	7.772	8.885938889	3	low_high
TCGA-Y6-A9XI-01	4.1825	7.7725	8.638727778	3	low_high
TCGA-KC-A7FA-01	3.9401	7.7794	8.518372222	3	low_high
TCGA-KK-A6E2-01	4.4457	7.7847	8.639333333	3	low_high
TCGA-KC-A4BN-01	4.6732	7.7919	8.395705556	3	low_high
TCGA-HC-7740-11	3.3105	7.796	8.204933333	3	low_high
TCGA-J4-8198-01	2.708	7.7984	8.390583333	3	low_high
TCGA-2A-AAYU-01	4.7543	7.8102	8.407922222	3	low_high
TCGA-EJ-7792-01	4.6486	7.8122	8.581233333	3	low_high
TCGA-EJ-7784-01	4.4101	7.8138	8.844616667	3	low_high
TCGA-HC-7231-01	1.4907	7.8145	8.555044444	3	low_high
TCGA-EJ-7786-01	4.7908	7.8145	8.387194444	3	low_high
TCGA-YL-A9WK-01	4.0086	7.838	8.816072222	3	low_high
TCGA-KK-A8IG-01	2.1533	7.8397	8.532016667	3	low_high
TCGA-EJ-5527-01	4.5872	7.8439	8.784488889	3	low_high
TCGA-EJ-7797-01	4.6679	7.8468	8.493883333	3	low_high
TCGA-CH-5768-01	4.6525	7.8509	8.953205556	3	low_high
TCGA-EJ-7317-11	3.0829	7.8533	8.107572222	3	low_high
TCGA-2A-A8W1-01	4.1315	7.8574	8.580855556	3	low_high
TCGA-EJ-7789-11	4.4657	7.8625	8.135261111	3	low_high
TCGA-EJ-7321-11	4.316	7.8717	8.175533333	3	low_high
TCGA-EJ-5498-01	4.4529	7.8744	8.555705556	3	low_high
TCGA-HC-7819-01	4.732	7.8767	8.600177778	3	low_high
TCGA-EJ-5502-01	4.0433	7.8784	8.58255	3	low_high
TCGA-CH-5789-01	3.6955	7.8796	8.317816667	3	low_high
TCGA-EJ-A46H-01	4.8147	7.8899	8.351244444	3	low_high
TCGA-EJ-7792-11	4.7498	7.8968	8.374005556	3	low_high
TCGA-KK-A7AW-01	4.3861	7.9009	8.56945	3	low_high
TCGA-YL-A8S9-01	4.6492	7.9049	8.758633333	3	low_high
TCGA-VP-A87K-01	4.4609	7.906	8.648972222	3	low_high
TCGA-HC-7232-01	3.4906	7.9079	8.300638889	3	low_high
TCGA-HC-7818-01	3.8611	7.9085	8.43545	3	low_high
TCGA-HC-8260-01	3.3407	7.9087	8.439288889	3	low_high
TCGA-HC-7742-11	3.8185	7.9114	8.323427778	3	low_high
TCGA-EJ-A8FU-01	4.5218	7.9134	8.392594444	3	low_high
TCGA-G9-6384-01	4.7664	7.9151	8.606894444	3	low_high
TCGA-EJ-7784-11	4.7041	7.9167	8.304377778	3	low_high

TCGA-EJ-8468-01	4.1515	7.9194	8.291927778	3	low_high
TCGA-EJ-7115-11	4.5704	7.9286	8.465022222	3	low_high
TCGA-FC-7708-01	4.1458	7.929	8.440033333	3	low_high
TCGA-KK-A8I6-01	4.1615	7.9345	8.748116667	3	low_high
TCGA-HC-A48F-01	4.2673	7.9365	8.366588889	3	low_high
TCGA-KC-A4BR-01	3.0231	7.9384	8.546211111	3	low_high
TCGA-HC-7737-11	3.2173	7.9405	8.079622222	3	low_high
TCGA-CH-5766-01	4.4404	7.945	8.681838889	3	low_high
TCGA-EJ-A8FO-11	4.3774	7.9477	8.62985	3	low_high
TCGA-KK-A8II-01	4.7648	7.9505	8.384155556	3	low_high
TCGA-V1-A9ZI-01	4.3683	7.954	8.864561111	3	low_high
TCGA-CH-5765-01	3.1206	7.9543	8.554077778	3	low_high
TCGA-V1-A8WS-01	3.602	7.9573	8.723183333	3	low_high
TCGA-EJ-7797-11	4.3704	7.9583	8.395661111	3	low_high
TCGA-KK-A8IA-01	3.7365	7.9621	8.945944444	3	low_high
TCGA-ZG-A9LY-01	4.2949	7.963	8.430188889	3	low_high
TCGA-VP-A87D-01	4.4263	7.9758	8.443205556	3	low_high
TCGA-EJ-5497-01	4.4499	7.9779	8.446988889	3	low_high
TCGA-KK-A8IM-01	4.7179	7.98	8.698188889	3	low_high
TCGA-HC-7738-11	3.5795	8.001	8.141277778	3	low_high
TCGA-CH-5753-01	3.6993	8.0053	8.6516	3	low_high
TCGA-VP-A87J-01	4.6411	8.0077	8.272355556	3	low_high
TCGA-EJ-7330-01	4.5045	8.0162	8.645472222	3	low_high
TCGA-VN-A88Q-01	3.0098	8.0189	8.3459	3	low_high
TCGA-EJ-5511-01	4.2798	8.0283	8.406827778	3	low_high
TCGA-HC-7211-01	4.2912	8.0382	8.749411111	3	low_high
TCGA-YL-A8HL-01	4.7278	8.0386	8.553733333	3	low_high
TCGA-J4-A83I-01	4.5147	8.0397	8.3788	3	low_high
TCGA-HC-7748-01	2.6531	8.054	8.537672222	3	low_high
TCGA-V1-A9ZG-01	3.3975	8.0703	8.770905556	3	low_high
TCGA-HC-7747-11	4.1095	8.0797	7.975972222	3	low_high
TCGA-VN-A88K-01	0.8767	8.1003	8.750605556	3	low_high
TCGA-YL-A8SA-01	3.7292	8.1006	8.133738889	3	low_high
TCGA-EJ-7331-11	4.3093	8.1017	8.447988889	3	low_high
TCGA-EJ-7793-11	4.4532	8.1109	8.466655556	3	low_high
TCGA-V1-A9ZK-01	4.738	8.1237	8.619766667	3	low_high
TCGA-HC-7211-11	3.1497	8.1407	8.171816667	3	low_high
TCGA-HC-A4ZV-01	4.022	8.1484	9.677855556	3	low_high
TCGA-EJ-5530-01	3.5036	8.1492	8.624344444	3	low_high
TCGA-EJ-7786-11	4.5406	8.1514	8.527616667	3	low_high
TCGA-HC-7745-11	3.6124	8.1582	8.094255556	3	low_high
TCGA-EJ-7794-11	4.7176	8.161	8.6779	3	low_high
TCGA-VN-A88R-01	4.3424	8.1814	8.900033333	3	low_high

TCGA-EJ-A65J-01	4.2797	8.1989	8.197938889	3	low_high
TCGA-MG-AAMC-01	2.479	8.2147	8.168672222	3	low_high
TCGA-2A-A8VT-01	4.8179	8.2283	8.619172222	3	low_high
TCGA-EJ-7328-11	3.2454	8.2324	8.473011111	3	low_high
TCGA-V1-A9O7-01	4.4576	8.2395	8.961361111	3	low_high
TCGA-YL-A8HK-01	4.3969	8.2413	8.977016667	3	low_high
TCGA-HC-8258-11	3.3223	8.2538	7.912644444	3	low_high
TCGA-HC-7212-01	4.5976	8.3057	8.687983333	3	low_high
TCGA-KC-A7FD-01	4.7853	8.3249	8.639094444	3	low_high
TCGA-G9-7521-01	4.1816	8.3383	8.742283333	3	low_high
TCGA-EJ-7783-11	4.5238	8.3552	8.341466667	3	low_high
TCGA-EJ-7781-11	4.7449	8.4912	8.476477778	3	low_high
TCGA-M7-A725-01	1.2674	8.5329	9.086594444	3	low_high
TCGA-EJ-7123-11	3.4421	8.6777	8.287033333	3	low_high
TCGA-FC-A4JI-01	0.4526	9.0311	10.00931111	3	low_high
TCGA-X4-A8KS-01	3.3361	6.3143	8.706561111	4	low_low
TCGA-V1-A9OT-01	2.6597	6.4502	8.627488889	4	low_low
TCGA-HC-7752-01	0	6.701	8.086055556	4	low_low
TCGA-KK-A6E3-01	3.7696	6.7219	8.523044444	4	low_low
TCGA-XJ-A9DQ-01	4.0785	6.7221	8.448244444	4	low_low
TCGA-G9-6362-11	3.8905	6.7458	8.846855556	4	low_low
TCGA-HC-A6HX-01	4.2661	6.7731	8.7181	4	low_low
TCGA-FC-A8O0-01	4.2669	6.7818	8.30055	4	low_low
TCGA-EJ-A65G-01	3.6018	6.8536	8.290566667	4	low_low
TCGA-HC-A6AQ-01	4.6448	6.8592	8.30465	4	low_low
TCGA-G9-6362-01	3.6752	6.8677	8.414883333	4	low_low
TCGA-HI-7169-01	2.4365	6.9087	8.349416667	4	low_low
TCGA-EJ-AB20-01	4.0259	6.9147	8.873455556	4	low_low
TCGA-QU-A6IP-01	4.829	6.9161	8.57675	4	low_low
TCGA-G9-6348-11	3.9138	6.9371	8.575172222	4	low_low
TCGA-G9-6496-11	4.0818	6.9573	8.348238889	4	low_low
TCGA-XJ-A9DX-01	3.6111	6.9589	8.583161111	4	low_low
TCGA-WW-A8ZI-01	3.207	6.9602	8.507794444	4	low_low
TCGA-J4-A67M-01	4.3427	6.9716	8.353438889	4	low_low
TCGA-2A-A8VL-01	3.994	6.9846	8.227461111	4	low_low
TCGA-G9-6378-01	3.9836	6.9916	8.525994444	4	low_low
TCGA-EJ-A46E-01	4.6709	6.9991	8.415405556	4	low_low
TCGA-G9-6342-11	4.2203	7.0133	8.490922222	4	low_low
TCGA-J4-A67R-01	4.8134	7.0144	8.532822222	4	low_low
TCGA-M7-A723-01	3.949	7.0147	8.493505556	4	low_low
TCGA-G9-6333-01	2.955	7.0149	8.5908	4	low_low
TCGA-CH-5741-01	4.5596	7.0542	8.421755556	4	low_low
TCGA-CH-5740-01	4.6757	7.062	8.877566667	4	low_low

TCGA-QU-A6IO-01	4.3535	7.0811	8.4184	4	low_low
TCGA-2A-AAYO-01	4.0186	7.085	8.74385	4	low_low
TCGA-2A-A8VO-01	4.5218	7.0936	8.537972222	4	low_low
TCGA-FC-A66V-01	4.384	7.1033	8.100194444	4	low_low
TCGA-HC-8265-01	4.6634	7.1097	8.803161111	4	low_low
TCGA-G9-6347-01	4.4738	7.1182	8.520494444	4	low_low
TCGA-G9-6365-11	4.4411	7.1217	8.470461111	4	low_low
TCGA-G9-6498-01	4.5487	7.1283	8.810516667	4	low_low
TCGA-G9-6333-11	2.4454	7.1299	8.31355	4	low_low
TCGA-G9-6367-01	4.0015	7.1301	8.28	4	low_low
TCGA-G9-6373-01	4.3846	7.1448	8.601772222	4	low_low
TCGA-HC-7080-01	4.1907	7.1457	8.542166667	4	low_low
TCGA-J4-A67O-01	3.7848	7.1458	8.560272222	4	low_low
TCGA-G9-6371-01	3.9355	7.1531	8.532238889	4	low_low
TCGA-CH-5772-01	4.532	7.1548	8.699294444	4	low_low
TCGA-EJ-A46B-01	2.7161	7.1549	8.190083333	4	low_low
TCGA-HC-7075-01	4.3785	7.1583	8.340677778	4	low_low
TCGA-KK-A7B3-01	4.8194	7.163	8.542583333	4	low_low
TCGA-G9-6332-01	2.897	7.1666	8.076488889	4	low_low
TCGA-G9-6385-01	0.4371	7.1695	8.477555556	4	low_low
TCGA-XK-AAJA-01	4.389	7.1731	8.755394444	4	low_low
TCGA-G9-6351-01	4.1366	7.186	8.252711111	4	low_low
TCGA-HC-8213-01	3.38	7.1861	8.123205556	4	low_low
TCGA-EJ-7325-01	3.5408	7.21	8.432083333	4	low_low
TCGA-CH-5737-01	4.4933	7.2179	8.534972222	4	low_low
TCGA-CH-5745-01	4.7826	7.2236	8.123855556	4	low_low
TCGA-HC-7747-01	4.6656	7.2265	8.5895	4	low_low
TCGA-G9-6351-11	4.0735	7.2371	8.2728	4	low_low
TCGA-G9-6379-01	4.4186	7.2454	8.396538889	4	low_low
TCGA-V1-A8MG-01	3.9714	7.2467	8.761538889	4	low_low
TCGA-G9-7519-01	4.3427	7.2513	8.544372222	4	low_low
TCGA-QU-A6IL-01	4.3198	7.2551	8.452588889	4	low_low
TCGA-EJ-7312-01	4.6096	7.2591	8.575877778	4	low_low
TCGA-M7-A71Y-01	3.8243	7.2598	8.409661111	4	low_low
TCGA-XA-A8JR-01	4.322	7.2727	8.464266667	4	low_low
TCGA-V1-A9ZR-01	3.9194	7.2731	8.370733333	4	low_low
TCGA-G9-6338-01	3.5436	7.2766	8.551027778	4	low_low
TCGA-G9-6353-01	3.5053	7.2884	8.3393	4	low_low
TCGA-VN-A88I-01	4.2773	7.2923	8.542394444	4	low_low
TCGA-KK-A7B2-01	4.0084	7.2994	8.718205556	4	low_low
TCGA-VN-A88M-01	3.2864	7.3147	8.442516667	4	low_low
TCGA-J4-A67S-01	3.5111	7.3151	8.442205556	4	low_low
TCGA-VN-A943-01	4.4329	7.318	8.01325	4	low_low

TCGA-V1-A8MK-01	3.974	7.3204	8.486177778	4	low_low
TCGA-J4-A83N-01	4.2466	7.3205	8.652244444	4	low_low
TCGA-HC-7749-01	4.5705	7.3351	8.481733333	4	low_low
TCGA-J4-AAU2-01	4.6401	7.3493	8.5464	4	low_low
TCGA-KC-A7F6-01	4.0868	7.3512	8.859794444	4	low_low
TCGA-EJ-A8FN-01	3.2874	7.3519	8.525627778	4	low_low
TCGA-HI-7171-01	4.4753	7.3586	9.126283333	4	low_low
TCGA-V1-A8ML-01	4.2889	7.3656	8.614338889	4	low_low
TCGA-J4-A83M-01	4.2262	7.3817	8.173811111	4	low_low
TCGA-G9-6342-01	3.9572	7.3871	8.7963	4	low_low
TCGA-M7-A71Z-01	4.3385	7.388	8.484555556	4	low_low
TCGA-ZG-A9L1-01	2.9107	7.3916	8.848338889	4	low_low
TCGA-2A-A8VV-01	3.1701	7.393	8.581383333	4	low_low
TCGA-KK-A6E5-01	3.5033	7.3938	8.603411111	4	low_low
TCGA-KK-A7AU-01	4.7486	7.4041	8.528927778	4	low_low
TCGA-HI-7168-01	4.2805	7.4086	9.17535	4	low_low
TCGA-2A-AAYF-01	3.6876	7.4088	8.237883333	4	low_low
TCGA-J9-A8CK-01	4.6892	7.4094	8.933566667	4	low_low
TCGA-J4-A67N-01	4.3254	7.412	8.296833333	4	low_low
TCGA-G9-6354-01	3.5526	7.4185	8.130266667	4	low_low
TCGA-G9-6361-01	3.9153	7.4185	8.601905556	4	low_low
TCGA-HC-8259-11	3.5796	7.4191	8.239672222	4	low_low
TCGA-J4-A83J-11	4.4391	7.4202	8.598583333	4	low_low
TCGA-G9-6384-11	3.8036	7.4205	8.216533333	4	low_low
TCGA-G9-6364-01	4.5605	7.4227	8.385633333	4	low_low
TCGA-VN-A88L-01	4.4867	7.4296	8.694438889	4	low_low
TCGA-VP-A87B-01	3.7338	7.4321	8.776238889	4	low_low
TCGA-HC-7078-01	4.7155	7.4346	8.777277778	4	low_low
TCGA-XJ-A83H-01	4.3124	7.4426	8.785866667	4	low_low
TCGA-KK-A7AV-01	4.7429	7.4479	8.618944444	4	low_low
TCGA-ZG-A9ND-01	2.9849	7.448	8.782127778	4	low_low
TCGA-CH-5768-11	2.3905	7.451	8.194272222	4	low_low
TCGA-HC-8260-11	3.2065	7.4525	8.05335	4	low_low
TCGA-XK-AAJ3-01	4.1732	7.4528	8.793944444	4	low_low
TCGA-HC-8259-01	4.563	7.4538	8.458283333	4	low_low
TCGA-V1-A9OF-01	3.3635	7.4542	8.551005556	4	low_low
TCGA-HC-A8CY-01	4.8233	7.4614	8.316655556	4	low_low
TCGA-HC-7740-01	4.3818	7.4684	8.366905556	4	low_low
TCGA-G9-6499-01	3.77	7.4727	8.530255556	4	low_low
TCGA-EJ-A65F-01	4.7569	7.4734	8.639244444	4	low_low
TCGA-XJ-A9DK-01	4.476	7.4767	8.716761111	4	low_low
TCGA-EJ-A46D-01	4.2529	7.4864	8.357227778	4	low_low
TCGA-J9-A8CP-01	4.7077	7.4932	8.66345	4	low_low

TCGA-EJ-A7NJ-01	3.5466	7.4947	8.2649	4	low_low
TCGA-V1-A9OY-01	4.1183	7.4997	8.788194444	4	low_low
TCGA-HC-7820-01	2.6795	7.5024	8.106877778	4	low_low
TCGA-EJ-5512-01	2.8545	7.5102	8.268566667	4	low_low
TCGA-HC-A9TE-01	4.3753	7.5116	8.439005556	4	low_low
TCGA-G9-7522-01	2.8728	7.5125	8.240933333	4	low_low
TCGA-XK-AAIR-01	4.8059	7.52	8.388994444	4	low_low
TCGA-EJ-A7NK-01	4.4922	7.522	8.397605556	4	low_low
TCGA-EJ-A8FP-01	3.7614	7.5243	8.279705556	4	low_low
TCGA-EJ-7317-01	3.7311	7.5269	8.515038889	4	low_low
TCGA-J4-A83L-01	3.8563	7.5276	8.403788889	4	low_low
TCGA-V1-A9OH-01	4.1172	7.5324	8.8175	4	low_low
TCGA-KK-A7AY-01	4.5178	7.536	8.542583333	4	low_low
TCGA-H9-7775-01	4.2155	7.5396	8.606272222	4	low_low
TCGA-CH-5763-01	4.6936	7.5406	8.488333333	4	low_low
TCGA-CH-5767-01	4.4339	7.5491	8.65025	4	low_low
TCGA-HC-8264-01	4.1973	7.5544	8.096938889	4	low_low
TCGA-HC-8257-01	4.4292	7.5601	8.733338889	4	low_low
TCGA-HC-A632-01	3.2205	7.5603	8.895072222	4	low_low
TCGA-XK-AAJT-01	3.8122	7.5642	8.642922222	4	low_low
TCGA-G9-6494-01	4.2427	7.568	8.590155556	4	low_low
TCGA-KK-A8I4-01	3.3316	7.5746	8.921294444	4	low_low
TCGA-G9-7509-01	3.3112	7.5838	8.336905556	4	low_low
TCGA-M7-A720-01	4.6178	7.5859	8.238727778	4	low_low
TCGA-HC-7745-01	3.9056	7.5921	8.451927778	4	low_low
TCGA-CH-5790-01	2.5206	7.5927	8.057661111	4	low_low
TCGA-EJ-A7NG-01	4.7194	7.5947	8.396133333	4	low_low
TCGA-YL-A8SB-01	4.4304	7.5971	8.898566667	4	low_low
TCGA-EJ-7327-11	3.6362	7.6054	8.191266667	4	low_low
TCGA-EJ-A7NH-01	3.5517	7.6081	8.489177778	4	low_low
TCGA-G9-6339-01	4.4022	7.61	8.371672222	4	low_low
TCGA-EJ-7321-01	4.6773	7.6178	8.669533333	4	low_low
TCGA-QU-A6IN-01	3.0193	7.6241	8.38265	4	low_low
TCGA-VP-A875-01	4.0859	7.6273	8.505111111	4	low_low
TCGA-H9-A6BX-01	3.6169	7.6343	8.743805556	4	low_low
TCGA-KK-A8I8-01	4.2504	7.6392	8.755272222	4	low_low
TCGA-HC-A8D1-01	3.0849	7.641	8.442127778	4	low_low

Table S6 The clinical features of melanoma patient cohorts in IHC assay.

Characteristics	Adjacent tissue (N=5)	Nevi (N=14)	Melanoma (N=67)
Gender			
Female	2	8	23
Male	3	6	44
Age			
≤60	4	8	43
> 60	1	6	24
7th AJCC T stage			
1	2	-	2
2	0	-	26
3	2	-	31
4	1	-	1
unknown	0	-	7

Table S7 Primers used for RT-PCR.

Gene	Forward sequence	Reverse sequence
APOBEC3B	CGCCAGACCTACTTGTGCTAT	CATTTGCAGCGCCTCCTTAT
GAPDH	AGGTCGGTGTGAACGGATTTG	TGTAGACCATGTAGTTGAGGTCA

Table S8 Primers used for cloning.

Name	Sequence (5' – 3')
hAPOBEC3B MluI Fwd	AAGACACCGGCGGCCACGCGTGCCACCATGAATCCACAGATCAGAAATC
hAPOBEC3B XhoI Rev	GTCATCCTTGTAATCCTCGAGGTTTCCCTGATTCTGGAGAA
hRNASEH1 BamHI Fwd	GATCTGGCCCGGGCGGGATCCATGAGCTGGCTTCTGTTCCT
linker hRNASEH1 Rev	CTCCACCGCCGCTTCCGCCTCCGTCTTCCGATTGTTTAGCTC
linker EGFP Fwd	AGGCGGAAGCGGCGGTGGAGGATCAATGGTGAGCAAGGGCGAGGAG
EGFP XhoI Rev	GTCATCCTTGTAATCCTCGAGCTACTTGTACAGCTCGTCCATGC
CDD1 Rev	ATTCTCGAGTCTGAGAATCTCCTTTAG
CDD2 Fwd	ATTACGCGTATGTACCTGATGGATCCA

Table S9 Replication stress response signature gene set.

KRAS	CCND2	NPM1	MYC	CCND1	MDM2
STAT5A	HRAS	CCNE1	CCND3	NRAS	DEK
CCNE2	CDC6	CDCA5	AURKA	MYBL2	E2F1
